# Cobalt-catalyzed alkyne silylamidation unlocks *Z*-selective unnatural dehydropeptides synthesis

**DOI:** 10.1093/nsr/nwag011

**Published:** 2026-01-09

**Authors:** Jixin Wang, Ting Zeng, Kaixin Chen, Zexu Chen, Long Lin, Wenhua Yu, Jianlin Yao, Hong Yi, Baosheng Wei, Jie Li

**Affiliations:** State Key Laboratory of Bioinspired Interfacial Materials Science, College of Chemistry, Chemical Engineering and Materials Science, Soochow University, Suzhou 215123, China; State Key Laboratory of Bioinspired Interfacial Materials Science, College of Chemistry, Chemical Engineering and Materials Science, Soochow University, Suzhou 215123, China; College of Chemistry and Molecular Sciences, The Institute for Advanced Studies (IAS), Wuhan University, Wuhan 430072, China; State Key Laboratory of Bioinspired Interfacial Materials Science, College of Chemistry, Chemical Engineering and Materials Science, Soochow University, Suzhou 215123, China; State Key Laboratory of Bioinspired Interfacial Materials Science, College of Chemistry, Chemical Engineering and Materials Science, Soochow University, Suzhou 215123, China; College of Chemistry and Chemical Engineering, Central South University, Changsha 410083, China; State Key Laboratory of Bioinspired Interfacial Materials Science, College of Chemistry, Chemical Engineering and Materials Science, Soochow University, Suzhou 215123, China; College of Chemistry and Molecular Sciences, The Institute for Advanced Studies (IAS), Wuhan University, Wuhan 430072, China; College of Chemistry and Chemical Engineering, Central South University, Changsha 410083, China; State Key Laboratory of Bioinspired Interfacial Materials Science, College of Chemistry, Chemical Engineering and Materials Science, Soochow University, Suzhou 215123, China; Suzhou Key Laboratory of Pathogen Bioscience and Anti-infective Medicine, Soochow University, Suzhou 215123, China; MOE Key Laboratory of Geriatric Diseases and Immunology, Soochow University, Suzhou 215123, China

**Keywords:** dehydropeptides, stereoselectivity, alkynes, silylamidation, cobalt catalysis

## Abstract

Due to their unique conformational properties and activities, dehydropeptides play a pivotal role in the fields of biological and medicinal chemistry. Yet, the synthesis of unnatural dehydropeptides still suffers from cumbersome steps and less generality, in particular, with rather limited structural diversity. Herein, a modular cobalt-catalyzed 1,2-silylamidation of non-conjugated alkynes with dioxazolones and silylzinc pivalates is disclosed, thus affording structurally diverse non-canonical sila-dehydropeptides with complete control of regio- and stereoselectivity. Notably, the reaction enables efficient peptide ligation between peptide-containing dioxazolones and peptide-containing alkynes in a *Z*/*E*-stereoselective and diastereoretentive manner. Moreover, broad substrate scope, outstanding functional group compatibility, as well as facile late-stage diversifications of pharmaceutically active molecules substantiate the synthetic value of this method.

## Teaser

Regio- and stereoselective cobalt-catalyzed silylative peptide-ligation of non-conjugated alkynes with dioxazolones and silylzinc pivalates unlocks Z-selective unnatural sila-dehydropeptides synthesis.

## INTRODUCTION

**Figure 1.**
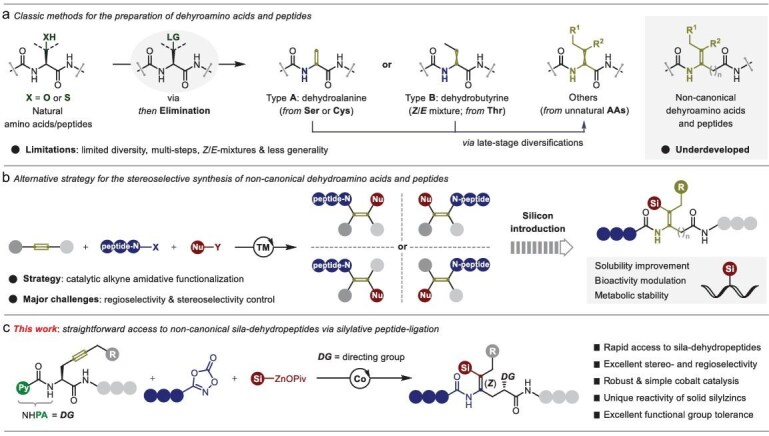
Background and project synopsis. (a) Classic methods for the preparation of dehydroamino acids and peptides. (b) Alternative strategy for the stereoselective synthesis of non-canonical dehydroamino acids and peptides. (c) Our approach to accessing non-canonical sila-dehydropeptides through regio- and stereoselective silylative peptide-ligation.

Dehydroamino acids, serving as nonproteinogenic building blocks, participate in a structurally diverse array of dehydropeptides with unique conformational properties [1] and activities [2], which have been afforded significant recent attention in the fields of biological, pharmaceutical and synthetic chemistry. Driven by the great potential of these unnatural amino acids for modulating the bioactivities of naturally occurring peptides or other bio-molecules, many efforts toward the efficient synthesis of dehydroamino acids have been made during the past decades [3,4]. Among them, dehydroalanine (Dha) and dehydrobutyrine (Dhb) as the most common residues, which can be facilely prepared from serine (Ser), cysteine (Cys) and threonine (Thr) residues via elimination processes [5–8], have found numerous applications in post-translationally modified peptides [9,10]. Beyond that, although cumbersome steps, less generality and *Z*/*E*-stereoselectivity control still represent significant drawbacks, late-stage diversifications of Dha- or Dhb-containing peptides, such as Heck-type arylation [11,12], C−H amidation [13] and oxidative phosphorylation [14], provide the stage to further expand the library of dehydroamino acids in peptide chemistry. In contrast to the common construction of conjugated *α,β*-dehydropeptides, the ability to efficiently prepare non-canonical dehydroamino acids and peptides in a stereoselective manner still remains a problematic scenario (Fig. 1a).

Given that tri- or tetrasubstituted alkene motifs are the essential backbones in dehydroamino acids and peptides, we believe that catalytic difunctionalization of alkynes should provide an attractive platform to construct such scaffolds. Thus far, 1,2-difunctionalization of terminal alkynes has been thoroughly studied, while catalytic 1,2-difunctionalization of unsymmetrical internal alkynes toward acyclic tetrasubstituted alkene synthesis still remains a crucial challenge, particularly when done in a regio- and *Z*/*E*-stereoselective manner [15,16]. Representative methods were largely limited to the use of electronically [17–27] or sterically [28] biased alkynes. Although hydroamidation of terminal alkynes [29,30] and intramolecular alkyne cyclized hydroamidation [31,32] have been described, catalytic regio- and stereoselective intermolecular hydrofunctionalization of unsymmetrical internal alkynes largely relied on the chelation-assistance of directing auxiliaries [33–36]. In addition, a palladium-catalyzed regio- and stereoselective 1,2-oxyhalogenation of non-conjugated alkynes has been only recently facilitated by a bidentate directing auxiliary [37]. Despite these major advances, extension to the synthesis of dehydropeptides through regio- and *Z*/*E*-stereoselective amidative functionalization of unsymmetrical alkynes has unfortunately thus far proven elusive (Fig. 1b, left). Driven by dramatic effects in the modulation of bioactivity, cell penetration, and metabolic stability, the development of synthetic methods for efficient incorporation of sila-functionalities into bioactive molecules has recently received significant attention in medicinal chemistry and biology settings [38–40]. As such, this void terrain offers new opportunities for chemical innovation by developing a practical catalysis that operates efficient alkyne silylative amidation to afford sila-dehydropeptides, which is therefore in high demand (Fig. 1b, right).

To achieve this goal, we hypothesized that a mild basic/nucleophilic silicon-based reagent with high reactivity and satisfactory functional group compatibility, particularly the peptide bonds, is highly required. Recently, we have demonstrated a new type of bench-stable and solid silylzinc reagent by replacing traditional halide anions with pivalate-coordination, which showed a distinct advantage of reacting well in transition metal-catalyzed silylative functionalization transformations [41–43]. These unique paradigms of anion-coordination in silylzinc reagents encouraged us to devise an efficient methodology to the synthesis of non-canonical dehydropeptides through cobalt-catalyzed silylative peptide-ligation of non-conjugated alkynes with dioxazolones and solid silylzinc pivalates. Key to our success is the chelation-assistance of a bidentate picolinamide (PA) auxiliary [33,44–49], which stimulates the catalytic activity of cobalt catalysts and steers 1,2-regioselectivity and *Z*/*E*-stereoselectivity control. Indeed, this approach allows us to rapidly and reliably incorporate silicon moieties and amino acids, peptides, or drug-like molecules into *L*-propargylglycine derivatives, thereby providing a straightforward access to sila-dehydropeptides with excellent functional group compatibility under mild reaction conditions (Fig. 1c).

## RESULTS

**Figure 2.**
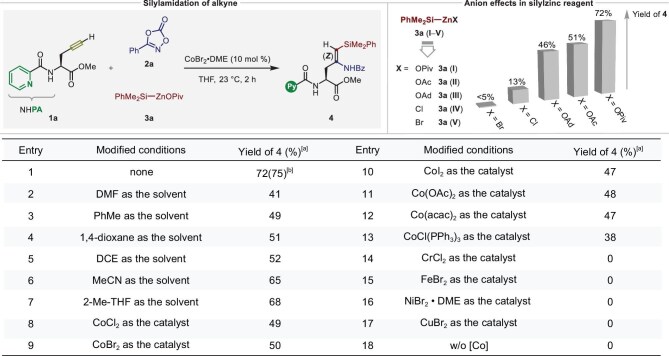
Optimization for regio- and stereoselective silylamidation of alkyne. Reaction conditions: **1a** (0.1 mmol, 1.0 equiv), **2a** (0.2 mmol, 2.0 equiv), **3a** (0.2 mmol, 2.0 equiv), [Co] (10 mol %), solvent (1.0 mL), @ 23°C, 2 h. [a] Isolated yields. [b] The yield of **4** was determined by ¹H-NMR analysis with CH_2_Br_2_ as the internal standard.

We began our studies by exploring the three-component silylamidation of *L*-propargylglycine derivative (**1a**) with dioxazolone (**2a**) and solid silylzinc pivalate (**3a**). As shown in Fig. 2, following a screening campaign investigating a variety of representative solvents (entries 1‒7) and typical cobalt complexes (entries 8‒13), we identified tetrahydrofuran (THF) as the reaction medium of choice and CoBr_2_•DME as the ideal metal source, furnishing the 1,2-*syn*-silylamidated product **4** in a 72% yield with complete control of *Z*-selectivity. However, switching from cobalt salts to other base-metal catalysts such as CrCl_2_, FeBr_2_, NiBr_2_•DME, or CuBr_2_ gave seriously negative results (entries 14‒17). Control experiments without a cobalt source showed the crucial role of the catalyst in this transformation (entry 18). Remarkably, anion-coordination in silylzinc reagents showed unique paradigms to tune their reactivity in this three-component reaction. Indeed, the more electron-rich carboxylate anion–supported silylzinc reagents displayed significantly superior reactivity than the halide-supported reagents in this alkyne silylamidation transformation, which highlighted the crucial importance of anion-effects in organozinc reagents [50–52].

**Figure 3.**
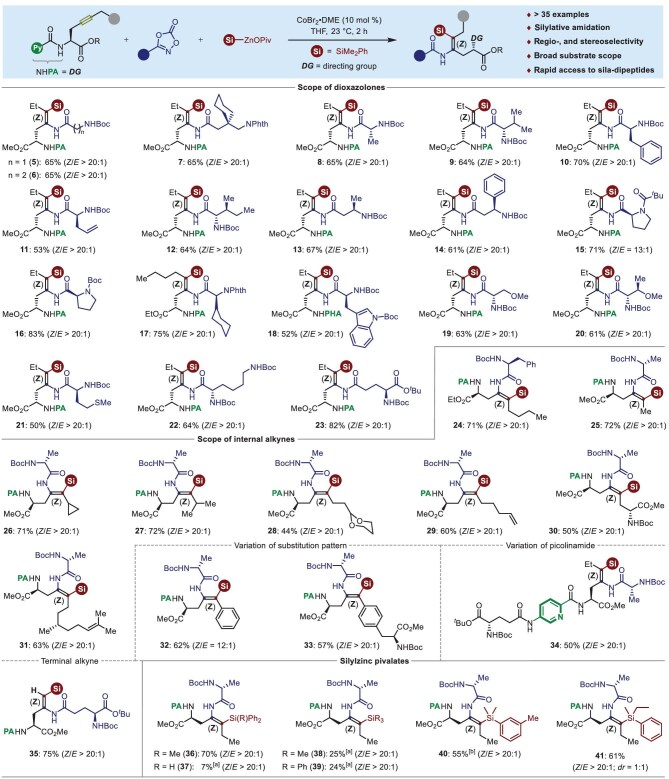
Substrate scope for cobalt-catalyzed stereoselective alkyne silylamidation. Reaction conditions: alkynes (**1**, 0.15 mmol, 1.0 equiv), dioxazolones (**2**, 2.0 equiv), silylzinc pivalates (**3**, 2.0 equiv), CoBr_2_•DME (10 mol %), THF (1.5 mL), @ 23°C, 2 h. [a] MeCN instead of THF as the solvent, 2 h. [b] Under 40°C for 2 h.

With the optimized reaction conditions in hand, we began to evaluate the substrate scope of the cobalt-catalyzed stereoselective silylamidation of internal alkynes with dioxazolones and silylzinc pivalates (Fig. 3, top). First, the dioxazolones derived from non-chiral amino acids, such as glycine (**5**), *β*-glycine (**6**) and *γ*-amino acid (**7**) proceeded smoothly to furnish the stereodefined sila-dehydrodipeptides with complete control of *Z*-selectivity. Thereafter, we turned our attention to investigating the generality of the reaction for the simultaneous control of both *Z*/*E*-stereoselectivity and diastereoselectivity by using the chiral amino acid-derived dioxazolones as the coupling partners. It is noteworthy that a variety of dioxazolones derived from alanine (**8**), valine (**9**), phenylalanine (**10**), allylglycine (**11**), isoleucine (**12**), *β*-alanine (**13**), *β*-phenylglycine (**14**), proline (**15**–**16**), cyclohexylglycine (**17**), tryptophan (**18**), serine (**19**), threonine (**20**), methionine (**21**), lysine (**22**) and glutamic acid (**23**) were identified to be viable substrates, thus uniquely affording the *Z*-selective sila-dehydrodipeptides in 50%‒83% yields with complete diastereoselectivity retention. Encouraged by these results, we next examined the substrate scope of alkynes with alanine-based dioxazolone (Fig. 3, middle). Various internal alkynes derived from *L*-propargylglycine are competent substrates for the in-sequence installation of silyl and amidyl motifs across the triple bonds in a regio- and stereoselective manifold, thereby delivering the desired silicon-containing dehydrodipeptides **24**‒**34** in moderate to good yields. As shown, the reaction was found to be compatible in the presence of sterically hindered alkyl substrates (**24**‒**31**), and functional groups such as strained cyclopropane (**26**), acetal (**28**), and both terminal (**29**) and internal olefins (**31**). Interestingly, the internally functionalized alkyne bearing two *L*-alanine motifs across the triple bond of potential regioselectivity for difunctionalization, uniquely underwent picolinamide- assisted 1,2-*syn*-silylamidation to afford the *δ*-sila-*γ*-amidated product **30** in a 50% yield, again with excellent *Z*-stereoselectivity and diastereoselectivity. Electronically biased alkynes possessing different aromatic moieties posed no problems when undergoing the chelation-assisted 1,2-silylamidation process, furnishing the stereodefined non-canonical dehydropeptides **32**‒**33** as the sole products in 57%‒62% yields. Gratifyingly, internal alkynes containing glutamic acid and propargylglycine with an aminopicolinic acid linker was also proven to be a viable substrate, leading to the desired product **34** in a 50% yield. Likewise, terminal alkyne smoothly underwent the *syn*-silylamidation to afford the corresponding dipeptide **35**. As shown for **37**‒**39**, the reaction was found to be very sensitive to the electronic variations of silylzinc reagents. Ph_2_HSi−ZnOPiv, Me_3_Si−ZnOPiv and Ph_3_Si−ZnOPiv only gave silylated dehydropeptides in rather modest yields using anhydrous acetonitrile as the reaction medium. However, MePh_2_Si−ZnOPiv (**36**), as well as (3-MePh)Me_2_Si−ZnOPiv (**40**) and PhEt(Me)Si−ZnOPiv (**41**) were identified as suitable nucleophilic partners to incorporate with alanine-derived dioxazolone into the *L*-propargylglycine scaffold, yielding the silylamidated products in 55%−70% yields (Fig. 3, bottom).

**Figure 4.**
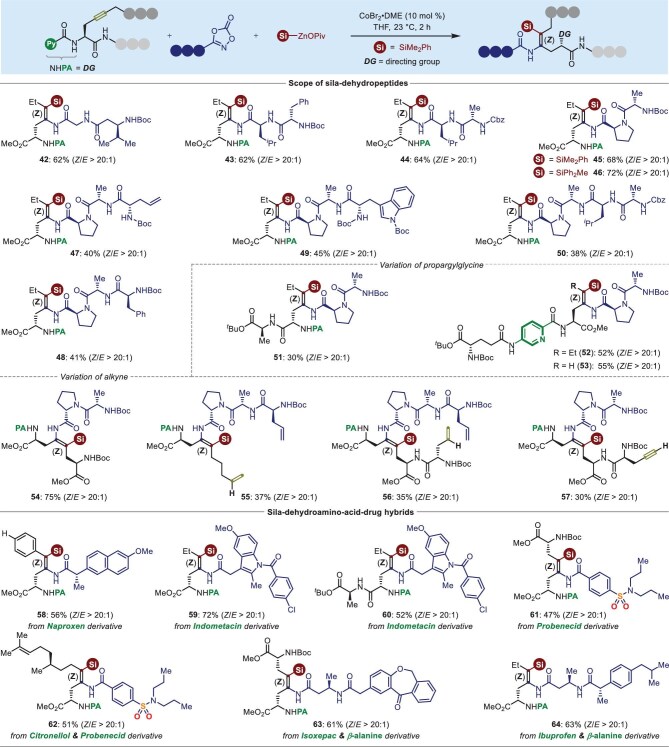
Peptide ligation through cobalt-catalyzed 1,2-*syn*-silylamidation for the assembly of sila-dehydropeptide hybrids. Reaction conditions: alkynes (**1**, 0.15 mmol, 1.0 equiv), dioxazolones (**2**, 2.0 equiv), silylzinc pivalates (**3**, 2.0 equiv), CoBr_2_•DME (10 mol %), THF (1.5 mL), @ 23°C, 2 h. Note: all the products were obtained with complete diastereoselectivity unless otherwise noted.

Encouraged by the versatility of cobalt-catalyzed regio- and stereoselective alkyne silylamidation, we applied this protocol to the direct chemical ligation of picolinamide-masked propargylglycine derivatives with the more challenging substrates of C-terminal peptide-dioxazolones (Fig. 4, top). As shown for **42**‒**46**, various dipeptide-dioxazolones, along with Boc-, and Cbz-protected *N*-termini were found to be perfectly tolerated under identical reaction conditions, giving silicon-containing dehydrotripeptides in good yields. Notably, our protocol could be further extended to the straightforward synthesis of silicon-containing dehydro-tetrapeptides **47**‒**49** and pentapeptide **50** using tri- and tetrapeptide-dioxazolones as nitrene precursors. Importantly, internal alkynes bearing a peptide regardless of the peptidic amino acid variation and ligation sequence, successfully incorporated dipeptide-dioxazolone and silicon moiety into a triple bond. The desired unnatural dehydropeptides **51**‒**53** were obtained in 30%‒55% yields. It is noteworthy that *L*-propargylglycines possessing olefins (**55**), amino acids (**54**) or peptides (**56,57**) on the opposite side of the triple bond could be coupled with various peptide-dioxazolones to give the sila-dehydropeptides **54**‒**57** with complete control of regio- and stereoselectivity, while the other terminal double and triple bonds remained untouched under these conditions. Furthermore, we demonstrated the synthetic utilization of our method to bio-conjugation with various dioxazolones derived from naproxen (**58**), indomethacin (**59**‒**60**) and probenecid (**61**‒**62**), the corresponding sila-dehydroamino acid-drug hybrids were obtained in 47%‒72% yields. Importantly, this selective alkyne silylamidation allowed for the bio-conjugation to form peptide-drug hybrids. Indeed, hybrid conjugates **63**‒**64** combined sila-dehydrodipeptide with isoxepac or ibuprofen were modularly assembled in 61%‒63% yields. These results should prove the robustness of our method toward modular synthesis of sila-drugs (Fig. 4, bottom).

**Figure 5.**
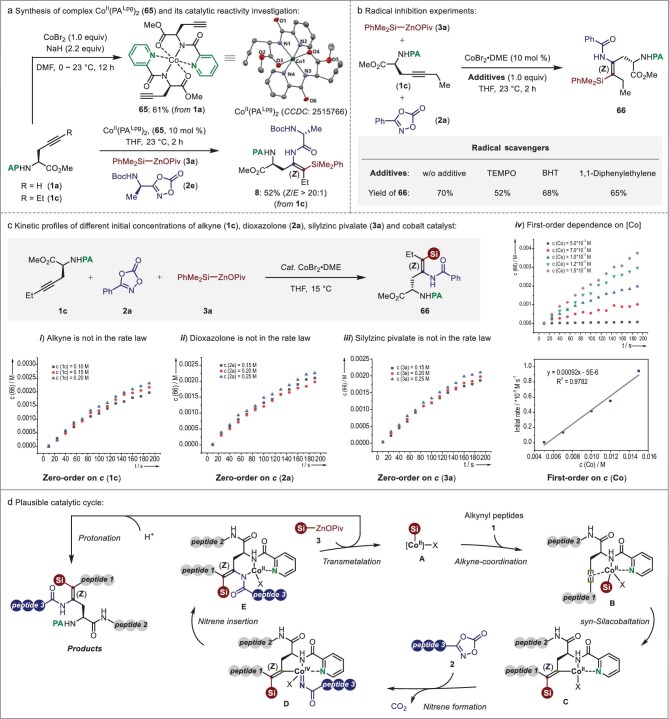
Mechanistic studies. (a) Synthesis of complex Co^II^(PA^Lpg^)_2_ (**65**) and its catalytic reactivity investigation. (b) Control experiments with radical scavengers. (c) Kinetic profiles of cobalt-catalyzed three-component silylamidation monitored by *in situ* IR spectroscopy. (d) Plausible catalytic cycle.

Following evaluation of the substrate scope, we thereafter turned our attention to gathering evidence about the reaction mechanism of alkyne silylamidation (Fig. 5). Initially, in order to investigate the chelation-assistance of bidentate picolinamide (PA) with cobalt catalyst, the Co^II^-complex **65** was obtained in a 61% yield upon the addition of NaH and CoBr_2_ to the PA-masked *L*-propargylglycine derivative (**1a**). Further X-ray diffraction analysis clarified the structure of **65**. Importantly, Co^II^(PA^Lpg^)_2_ was found to be adequate as the catalyst for the envisioned alkyne silylamidation reaction, affording the sila-dehydropeptide **8** in a 52% yield with excellent regio- and *Z*-stereoselectivity (Fig. 5a). Moreover, a range of alkynes bearing different bidentate directing groups, such as 8-aminoquinoline, internal-triazole, or methyl-blocked picolinamide, were proven to be unsuitable substrates, failing to occur in this three-component coupling reaction (see Fig. S2). These results should highlight the critical chelation-assistance of PA-directing groups. In addition, this cobalt-catalyzed silylamidation of alkynes occurred smoothly even in the presence of stoichiometric quantities of 2,2,6,6-tetramethyl-1-piperinedinyloxy (TEMPO), butylated hydroxytoluene (BHT) or 1,1-diphenylethene, affording the desired silylamidated product **66** in 52%‒68% yields. These observations demonstrate that a radical pathway could be excluded as the mechanism (Fig. 5b). Moreover, further kinetic studies were also conducted in THF at 15°C. It is worth noting that variations in concentration of alkyne, dioxazolone and silylzinc pivalate showed only negligible change in initial rates (Fig. 5c, i−iii). In sharp contrast, plots of the initial rates for alkyne silylamidation versus concentrations of cobalt catalyst gave linear curves (Fig. 5c, iv). These results indicated a first order rate dependence on the catalyst and zero order on the concentration of starting materials.

By joining our mechanistic studies and previous insights [31,32], a plausible mechanism for this cobalt-catalyzed stereoselective alkyne silylamidation is proposed in Fig. 5d. Initially, the *in situ* formed silacobalt-species **A** via a transmetalation reaction between Co^II^-complex and silylzinc pivalate (**3**) selective coordinates to the bidentate picolinamide (PA) auxiliary of alkyne (**1**) led to intermediate **B**. Subsequent intramolecular *syn*-silacobaltation of **B** affords the 5,5-bicyclic complex **C**, which can bind to dioxazolone (**2**) and furnish the key intermediate of Co^IV^-nitrene **D** by releasing CO_2_. Thereafter, the highly electrophilic nature of the Co^IV^-nitrenoid moiety allows for facile nitrene insertion with the nucleophilic alkenyl center to form intermediate **E**. Finally, silacobalt-species **A** is regenerated by another silylzinc pivalate liberating the desired product.

**Figure 6.**
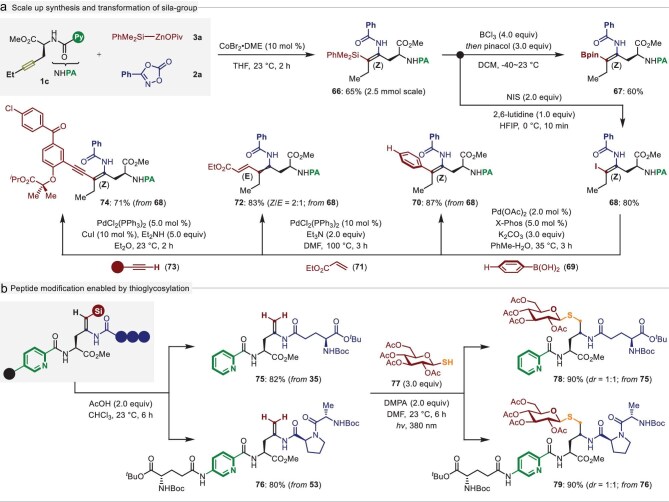
Synthetic utilizations. (a) Scale up synthesis and diverse conversions of alkenyl silianes. (b) Peptide modification through facile thioglycosylation. Note: all the products were obtained with complete *Z*-stereoselectivity unless otherwise noted and the diastereoselectivities were determined by ^1^H NMR spectroscopy.

Finally, we sought to demonstrate the later synthetic utility of this cobalt-catalyzed stereoselective silylamidation protocol (Fig. 6). First, a scale-up transformation was conducted and afforded the desired product **66** in a 65% yield. It is worth noting that the resulting compound **66** could be easily subjected to facile desilylative borylation in the presence of BCl_3_ and pinacol, furnishing the stereodefined tetrasubstituted alkenyl borate **67** in a 60% yield in a stereoretentive fashion. On the other hand, upon treatment of the silylamidated compound **66** with *N*-iodosuccinimide (NIS) and 2,6-lutidine, a selective alkenyl Csp^2^‒Si bond iodization occurred to afford the *Z*-selective product **68** in an 80% yield. With this alkenyl iodide in hand, classic cross-coupling reactions such as Suzuki-, Heck-, and Sonogashira-couplings were smoothly conducted, thereby leading to the arylated (**70**) and alkynylated (**74**) dehydroamino acids in good yields and excellent *Z*/*E*-stereoselectivity, while the Heck-type conjugated diene **72** was obtained with a 2:1 *Z*/*E*-ratio (Fig. 6a).

In addition, following a facile desilylation process, the tri-substituted sila-dehydropeptides **35** and **53** were transformed to the terminal alkene-containing dehydropeptides **75**‒**76** in high yields, which can further serve as a versatile functional group at later synthetic stages. To this end, the resulting products were subjected as bioorthogonal handles to ligate a thioglucoside **77** motif through a thiol-ene click reaction [53], thus affording thioether-linked glucoside-peptide hybrids **78**‒**79** in excellent yields (Fig. 6b). As such, these results should prove the synthetic value of our method and leave ample space for the synthesis of unnatural peptides with structural diversity.

## CONCLUSION

In conclusion, we have disclosed a practical cobalt-catalyzed unprecedented alkyne silylamidation with readily accessible amino acids or peptides derived from dioxazolones and solid silylzinc pivalates, thus providing straightforward access to the synthesis of sila-dehydropeptides with complete control of regioselectivity and *Z/E*-stereoselectivity. A broad array of functionalized alkynyl amides, dioxazolones and silylzinc pivalates are compatible under both simple and mild reaction conditions. Moreover, the scope encompasses late-stage modification of various pharmaceuticals and downstream transformations of the resulting products to make structurally diverse unnatural peptides that substantiate the great potential of our current method in synthetic and biological chemistry. Further development of the related applications with unnatural sila-dehydropeptides is currently ongoing in our laboratory and will be reported in due course.

## METHODS

### General procedure for the cobalt-catalyzed alkyne silylamidation

**Condition A**: In a nitrogen-filled glovebox, alkyne **1** (1.0 equiv.), dioxazolone **2** (2.0 equiv.), CoBr₂•DME (10 mol %) and anhydrous THF (0.5 mL) were added to an oven-dried 10-mL scintillation vial equipped with a Teflon-coated magnetic stir bar. The vial was sealed with a screw-top septum cap and removed from the glovebox. A solution of silylzinc pivalate **3** (2.0 equiv.) in anhydrous THF (1.0 mL) or THF (0.5 mL) was then added dropwise via a syringe to the reaction mixture at 23°C under argon atmosphere, with stirring, for 2 h. The mixture was then diluted with DCM (4 mL) and quenched with saturated aqueous NaHCO_3_ solution (3 mL). The resulting mixture was extracted with DCM (10 mL × 3). The combined organic layers were dried over Na_2_SO_4_, filtered, and concentrated under reduced pressure. The crude product was purified by column chromatography on silica gel to afford the desired silylamidation product.

**Condition B**: In a nitrogen-filled glovebox, alkyne **1** (1.0 equiv.), dioxazolone **2** (2.0 equiv.), CoBr₂•DME (10 mol %) and anhydrous MeCN (0.5 mL) were added to an oven-dried 10-mL scintillation vial equipped with a Teflon-coated magnetic stir bar. The vial was sealed with a screw-top septum cap and removed from the glovebox. A solution of silylzinc pivalate **3** (2.0 equiv.) in anhydrous MeCN (1.0 mL) or MeCN (0.5 mL) was then added dropwise via a syringe to the reaction mixture at 23°C under argon atmosphere, with stirring, for 2 h. The mixture was then diluted with DCM (4 mL) and quenched with saturated aqueous NaHCO_3_ solution (3 mL). The resulting mixture was extracted with DCM (10 mL × 3). The combined organic layers were dried over Na_2_SO_4_, filtered, and concentrated under reduced pressure. The crude product was purified by column chromatography on silica gel to afford the desired silylamidation product.

## Supplementary Material

nwag011_Supplemental_Files

## Data Availability

The authors declare that all other data supporting the findings of this study, including experimental procedures and compound characterization, are available within the article and the Supplementary Information. Source data are provided with this paper. The X-ray crystallographic data for structure **65** used in this study are available in the joint Cambridge Crystallographic Data Centre (CCDC 2515766) and Fachinformationszentrum Karlsruhe Access Structures service https://www.ccdc.cam.ac.uk/structures/. All data are available from the corresponding author upon request.
