# Supplementary material for: Cobalt-catalyzed alkyne silylamidation unlocks Z-selective unnatural dehydropeptides synthesis
Source: Natl Sci Rev. 2026 Jan 9;13(5):nwag011. doi: 10.1093/nsr/nwag011 (PMC12954855; doi:10.1093/nsr/nwag011)
Supplement: nwag011_Supplemental_Files [file nwag011_supplemental_files.zip › Supporting_Information.pdf]

Supporting Information

Cobalt-Catalyzed Alkyne Silylamidation Unlocks *Z*-Selective Unnatural Dehydropeptides Synthesis

Jixin Wang,[1‡] Ting Zeng,[1‡] Kaixin Chen,[2] Zexu Chen,[1] Jianlin Yao,[1]* Long Lin,[1] Hong Yi,[2]* Baosheng Wei,[3]* and Jie Li[1,4,5]*

[1] State Key Laboratory of Bioinspired Interfacial Materials Science, College of Chemistry, Chemical Engineering and Materials Science, Soochow University, Suzhou, China

[2] College of Chemistry and Molecular Sciences, The Institute for Advanced Studies (IAS), Wuhan University, Wuhan, China

[3] College of Chemistry and Chemical Engineering, Central South University, Changsha 410083, China

[4] Suzhou Key Laboratory of Pathogen Bioscience and Anti-infective Medicine, Soochow University, Suzhou, China

[5] MOE Key Laboratory of Geriatric Diseases and Immunology, Soochow University, Suzhou, China

E-mails: jlyao@suda.edu.cn; hong.yi@whu.edu.cn; bswei@csu.edu.cn; jjackli@suda.edu.cn

Contents

General Remarks······S2
Optimization Studies······S3
Additional Experiments······S5
Preparation of Alkynes······S13
Preparation of Dioxazolones······S30
Preparation and Stability Study of Silylzinc Reagents······S42
General Procedure for Co-Catalyzed Silylamidation······S47
Characterization Data of Products **4−64**······S49
Gram-Scale Experiments and Synthetic Applications······S95
X-Ray Crystallography Data of **65**······S107
References······S109
NMR Spectra······S111

General Remarks

Unless otherwise indicated, all reactions were carried out with magnetic stirring and in flame-dried glassware under argon. Other chemicals were obtained from commercial sources and were used without further purification. Superdry solvents, THF, DMF, DMA, MeCN, PhMe and DCE were purchased form commercial sources. Yields refer to isolated compounds, estimated to be > 95% pure as determined by ^{1}H-NMR and LC-analysis. Reactions were monitored by High Performance Liquid Chromatography (HPLC) or thin layer chromatography (TLC). TLC were performed using aluminum plates covered with SiO_2 (Merck 60, F-254) and visualized by UV detection. ^{1}H-NMR spectra were recorded on a BRUKER AVANCE III HD (400 MHz and 600 MHz) spectrometer. Chemical shifts are reported in ppm from TMS with the solvent resonance as internal standard ($CDCl_3$: δ 7.26). The following abbreviations (or combinations thereof) were used to explain multiplicities: *br* = broad, *s* = singlet, *d* = doublet, *dd* = doublet of doublet, *t* = triplet, *q* = quartet, *hept* = heptet, and *m* = multiplet. ^{13}C-NMR spectra were recorded on a BRUKER AVANCE III HD (101 MHz and 151 MHz) spectrometer with complete proton decoupling. Chemical shifts are reported in ppm from TMS with the solvent resonance as the internal standard ($CDCl_3$: δ 77.160). Mass spectrometry and high-resolution mass spectrometry (HRMS) recordings use electrospray ionization (ESI) unless otherwise noted. For the React IR kinetic experiments, the reaction spectra were recorded using an IC 10 and IC 15 from Mettler-Toledo AutoChem. Data manipulation was carried out using the IC IR software, version 4.2. High-Performance Liquid Chromatography (HPLC) were recorded on an Dionex UltiMate 3000 U3000 Flow Rate Range: 200-10,000 u L/min.

Optimization Studies

Table S1. Optimization for co-catalyzed silylamidation of alkyne **1a**[a]

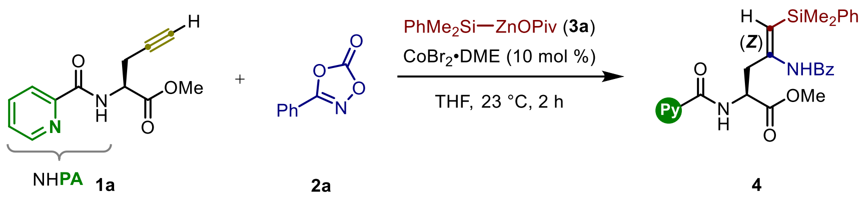

Entry	Modified conditions	Yield (%)[a]
**1**	**none**	**72/75[b]**
2	DMF as the solvent	41
3	PhMe as the solvent	49
4	1,4-dioxane as the solvent	51
5	DCE as the solvent	52
6	MeCN as the solvent	65
7	2-Me-THF as the solvent	68
8	$CoCl_2$ as the catalyst	49
9	$CoBr_2$ as the catalyst	50
10	CoI_2 as the catalyst	47
11	$Co(OAc)_2$ as the catalyst	48
12	$Co(acac)_2$ as the catalyst	47
13	$CoCl(PPh_3)_3$ as the catalyst	38
14	$CrCl_2$ as the catalyst	0
15	$FeBr_2$ as the catalyst	0
16	$NiBr_2$•DME as the catalyst	0
17	$CuBr_2$ as the catalyst	0
18	w/o [Co]	0
19	At 50 °C	57
20	At 0 °C	60

Reaction conditions: **1a** (0.1 mmol, 1.0 equiv.), **2a** (0.2 mmol, 2.0 equiv.), **3a** (0.2 mmol, 2.0 equiv.), [Co] (10 mol %), solvent (1.0 mL), @ 23 °C, 2 h. [a] Isolated yields. [b] The product yield was determined by ^{1}H-NMR spectroscopy with CH_2Br_2 as the internal standard.

Anion-effects in silylzinc reagents

Procedure for Anion-effects in silylzinc reagents: In a nitrogen-filled glovebox, alkyne **1a** (23.2 mg, 0.1 mmol, 1.0 equiv.), dioxazolone **2a** (32.6 mg, 0.2 mmol, 2.0 equiv.), $CoBr_2$•DME (3.1 mg, 10 mol %), and anhydrous THF (0.5 mL) were added to an oven-dried 10-mL scintillation vial equipped with a Teflon-coated magnetic stir bar. The vial

was sealed with a screw-top septum cap and removed from the glovebox. A solution of $Me_2PhSi-ZnX$ (X = OPiv, OAc, OAd, Cl, Br) (0.2 mmol, 2.0 equiv.) in anhydrous THF (0.5 mL) was then added dropwise via syringe to the reaction mixture at 23 °C under argon atmosphere with stirring. The reaction was stirred under argon atmosphere at 23 °C for 2 h. The mixture was then diluted with DCM (4 mL) and quenched with saturated aq. $NaHCO_3$ solution (3 mL). The resulting mixture was extracted with DCM (10 mL × 3). The combined organic layers were dried over Na_2SO_4, filtered, and concentrated under reduced pressure. The crude product was purified by column chromatography (petroleum ether/EtOAc = 2:1) on silica gel to afford the desired silylamidation product **4**.

Table S2. Anion-effects in silylzinc reagent.

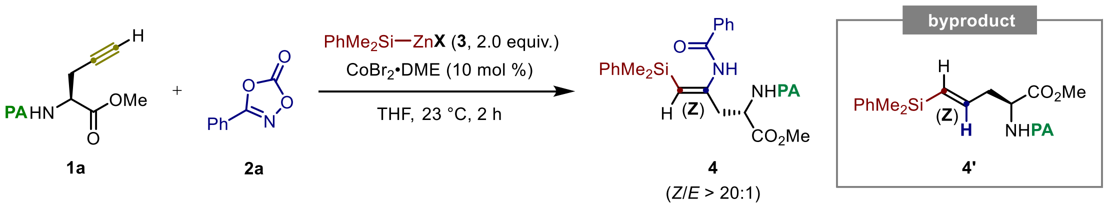

Entry	X	Yield		
		**4** (%)[a]	**4'** (%)[a]	**1a**[a]
1	OPiv	72 (75)[b]	8	trace
2	OAc	51	24	9
3	OAd	46	28	8
4	Cl	13	71	trace
5	Br	trace	46	47

Reaction conditions: **1a** (0.1 mmol, 1.0 equiv), **2a** (0.2 mmol, 2.0 equiv), **3** (0.2 mmol, 2.0 equiv), $CoBr_2$•DME (10 mol %), THF (1.0 mL), @ 23 °C, 2 h. [a] Isolated yields. [b] The yield of **4** was determined by ^{1}H-NMR analysis with CH_2Br_2 as the internal standard.

Additional Experiments

a) Synthesis and catalytic activity study of $Co^{II}(PA^{Lpg})_2$

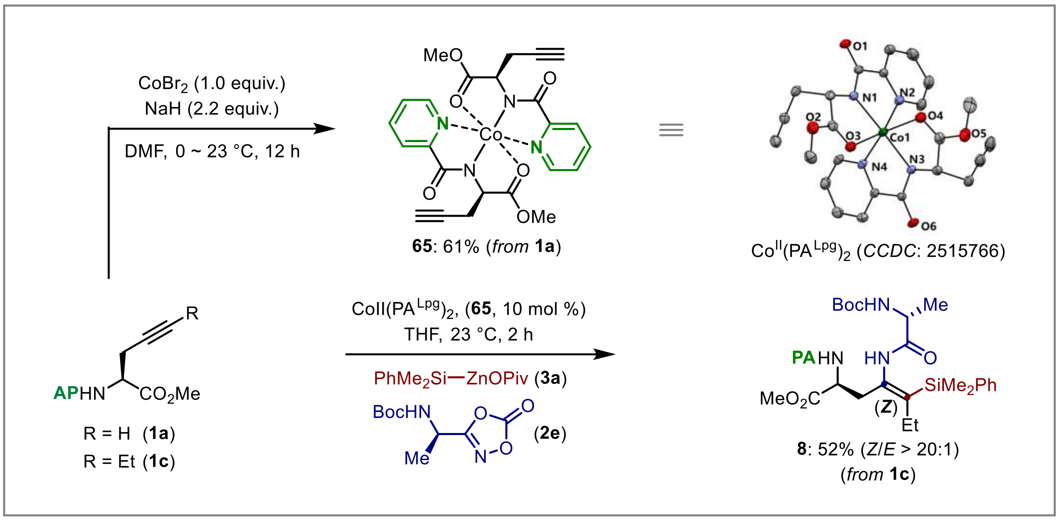

Figure S1. Synthesis and catalytic activity study of $Co^{II}(PA^{Lpg})_2$.

Synthesis of complex $Co^{II}(PA^{Lpg})_2$： A flame-dried 25 mL Schlenk tube equipped with a magnetic stir bar was charged with **1a** (232 mg, 1.0 mmol, 2.0 equiv.). The tube was evacuated and backfilled with N_2. Dry DMF (10 mL) was added via syringe. NaH (44 mg, 1.1 mmol, 2.2 equiv., 60% dispersion in mineral oil) was added at 0 °C, and the mixture was stirred at this temperature for 1 h. To the resulting homogeneous pale yellow solution, $CoBr_2$ (110 mg, 0.5 mmol, 1.0 equiv.) was added in one portion. The reaction mixture was then allowed to stir under N_2 at 23 °C for 12 h. The resulting dark green solution was concentrated to dryness under reduced pressure to afford a slate green solid. The solid was triturated with dry CH_2Cl_2, and the extract was filtered through a plug of Celite®. The filtrate was layered with dry hexane, yielding the product as green needle-shaped crystals **65** (158 mg, 61%). HR-MS (ESI) m/z calcd for $C_{24}H_{22}CoN_4O_6$: 521.0871, found: 521.0876.

Catalytic Activity Studies of $Co^{II}(PA^{Lpg})_2$: In a nitrogen-filled glovebox, alkyne **1c** (26.0 mg, 0.1 mmol, 1.0 equiv.), dioxazolone **2e** (46 mg, 0.2 mmol, 2.0 equiv.), **$Co^{II}(PA^{Lpg})_2$** (5.2 mg, 10 mol %) and anhydrous THF (0.5 mL) were added to an oven-

dried 10-mL scintillation vial equipped with a Teflon-coated magnetic stir bar. The vial was sealed with a screw-top septum cap and removed from the glovebox. A solution of silylzinc pivalate **3a** (0.2 mmol, 2.0 equiv.) in anhydrous THF (0.5 mL) was then added dropwise via syringe to the reaction mixture at 23 °C under argon atmosphere with stirring. The reaction was stirred under argon atmosphere at 23 °C for 2 h. The mixture was then diluted with DCM (4 mL) and quenched with saturated aq. $NaHCO_3$ solution (3 mL). The resulting mixture was extracted with DCM (10 mL × 3). The combined organic layers were dried over Na_2SO_4, filtered, and concentrated under reduced pressure. The crude product was purified by column chromatography (petroleum ether/EtOAc = 2:1) on silica gel to afford the desired silylamidation product **8** (30.3 mg, 52%).

b) Chelation-assistance of directing groups

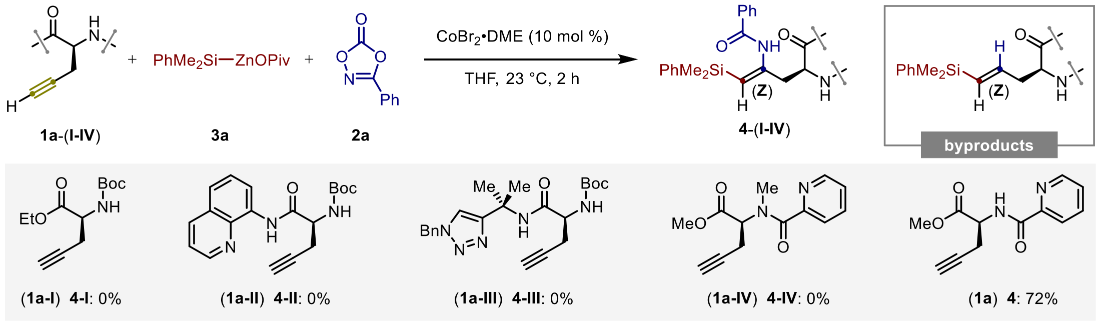

Figure S2. Chelation-assistance of directing groups.

Procedures for chelation-assistance of directing groups: In a nitrogen-filled glovebox, alkyne **1a** (23.2 mg, 0.1 mmol, 1.0 equiv.) or **1a-I** (24.1 mg, 0.1 mmol, 1.0 equiv.) or **1a-II** (33.9 mg, 0.1 mmol, 1.0 equiv.) or **1a-III** (41.1 mg, 0.1 mmol, 1.0 equiv.), or **1a-IV** (24.6 mg, 0.1 mmol, 1.0 equiv.), dioxazolone **2a** (32.6 mg, 0.2 mmol, 1.0 equiv.), $CoBr_2$•DME (3.1 mg, 10 mol %), and anhydrous THF (0.5 mL) were added to an oven-dried 10-mL scintillation vial equipped with a Teflon-coated magnetic stir bar. The vial was sealed with a screw-top septum cap and removed from the glovebox. A solution of silylzinc pivalate **3a** (0.2 mmol, 2.0 equiv.) in anhydrous THF (0.5 mL) was then added dropwise via syringe to the reaction mixture at 23 °C under argon

atmosphere with stirring. The reaction was stirred under argon atmosphere at 23 °C for 2 h. The mixture was then diluted with DCM (4 mL) and quenched with saturated aq. $NaHCO_3$ solution (3 mL). The resulting mixture was extracted with DCM (10 mL × 3). The combined organic layers were dried over Na_2SO_4, filtered, and concentrated under reduced pressure. The crude product was purified by column chromatography on silica gel to afford the desired silylamidation product. The experimental results indicate that the three-component silylamidation reaction did not proceed with alkynes **1a**-(**I**–**IV**), and hydrosilylation of the alkyne predominated as the major side reaction. In contrast, picolinamide-masked propargylglycine derivative **1a** occurred cobalt-catalyzed 1,2-*syn*-silylamidation to afford the desired product **4** in 72% yield. These results should highlight the unique chelation-assistance of PA-directing group.

c) Radical inhibition experiments

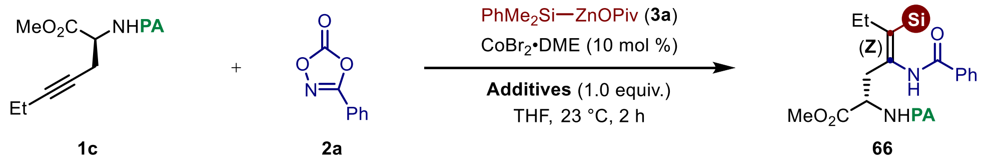

Additives:	w/o additive	TEMPO	BHT	1,1-Diphenylethylene
Yield of **66**:	70%	52%	68%	65%

Figure S3. Radical inhibition experiments.

Procedures for radical inhibition experiments: In a nitrogen-filled glovebox, alkyne **1c** (26.0 mg, 0.1 mmol, 1.0 equiv.), dioxazolone **2a** (32.6 mg, 0.2 mmol, 2.0 equiv.), $CoBr_2$•DME (3.1 mg, 10 mol %), anhydrous THF (0.5 mL), and 2,2,6,6-tetramethylpiperidinooxy (TEMPO) (15.6 mg, 0.1 mmol, 1.0 equiv.) or butylated hydroxytoluene (BHT) (22.0 mg, 0.1 mmol, 1.0 equiv.) or 1,1-diphenylethylene (18.0 mg, 0.1 mmol, 1.0 equiv.) were added to an oven-dried 10-mL scintillation vial equipped with a Teflon-coated magnetic stir bar. The vial was sealed with a screw-top septum cap and removed from the glovebox. A solution of silylzinc pivalate **3a** (0.2

mmol, 2.0 equiv.) in anhydrous THF (0.5 mL) was then added dropwise via syringe to the reaction mixture at 23 °C under argon atmosphere with stirring. The reaction was stirred under argon atmosphere at 23 °C for 2 h. The mixture was then diluted with DCM (4 mL) and quenched with saturated aq. $NaHCO_3$ solution (3 mL). The resulting mixture was extracted with DCM (10 mL × 3). The combined organic layers were dried over Na_2SO_4, filtered, and concentrated under reduced pressure. The crude product was purified by column chromatography (petroleum ether/EtOAc = 2:1) on silica gel to afford the desired silylamidation product **66**. The control reaction without additive provided the highest yield (36.0 mg, 70%), while TEMPO showed the most significant inhibitory effect (27.0 mg, 52% yield). The use of BHT as additive afforded the product in 63% yield (35.0 mg). The use of 1,1-diphenylethylene as additive afforded the product in 65% yield (33.5 mg).

d) Kinetic profiles

Note: Due to the reaction rate is too fast to collect data under room temperature, performing the kinetic experiments under a lower reaction temperature to slow down the reaction rate is necessary. In this context, all of the kinetics experiments were performed at 15 ºC.

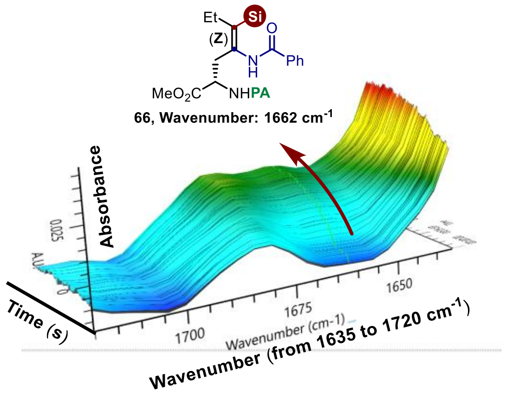

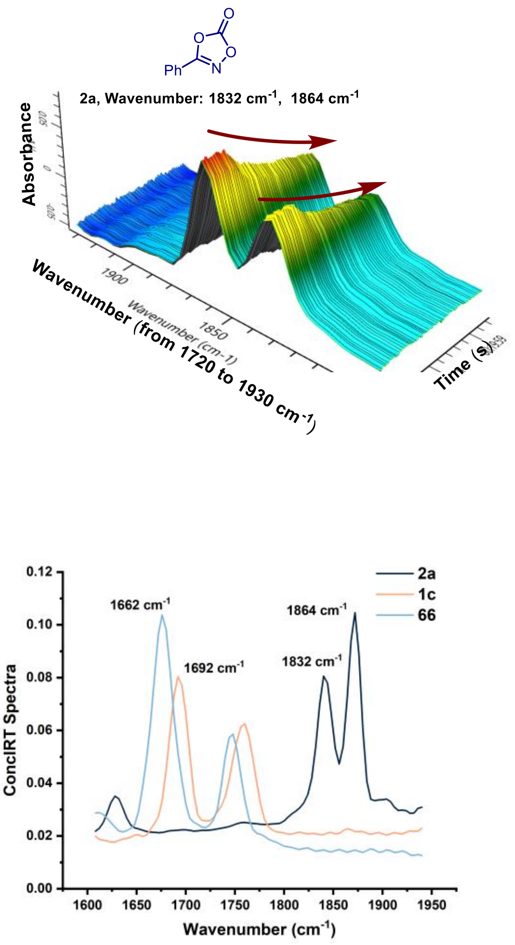

Figure S4. Local 3D in-situ IR spectrum (1600 cm^{-1} to 1900 cm^{-1}).

Procedure for Kinetic profiles: ConcIRT Spectrum of dioxazolone **2a** with peak at 1832, 1864 wavenumber cm^{-1} and the product **66**with peak at 1662 wavenumber cm^{-1} were used for the generation of the kinetic profiles.

i) Kinetic profiles of different initial concentrations of alkyne **1c**

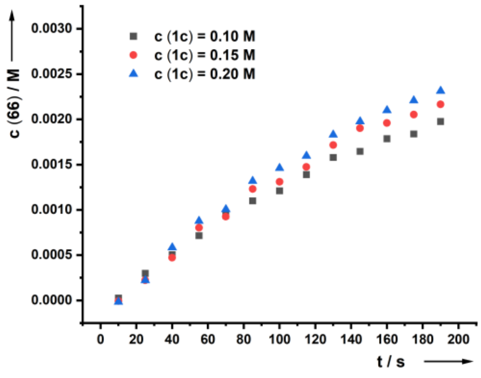

Figure S5. Kinetic profiles of different initial concentrations of alkyne **1c**.

Procedure for Kinetic profiles of different initial concentrations of alkyne 1c: In an oven-dried three-necked reactor was charged with alkyne **1c** (from 0.1 mmol to 0.2 mmol) and dioxazolone **2a** (2.0 equiv.). After evacuated and backfilled with nitrogen three times, anhydrous THF (0.5 mL) was added. Then the silylzinc pivalate **3a** (2.0 equiv.) in anhydrous THF (0.5 mL) was added via a syringe at 25 °C. Afterwards, we used cooling bath to keep the reaction temperature at 15 °C and $CoBr_2$•DME (10 mol %) in anhydrous THF (20 μL) were added at 15 °C. After 30 minutes, the reaction quenched by EtOH. The yield was identified by HPLC analysis with biphenyl as an internal standard after IR tests (every 5 seconds for one scan). All of these reactions exhibit zero-order kinetic behavior and the initial rate were obtained as the slopes of the straight lines.

ii) Kinetic profiles of different initial concentrations of dioxazolone **2a**

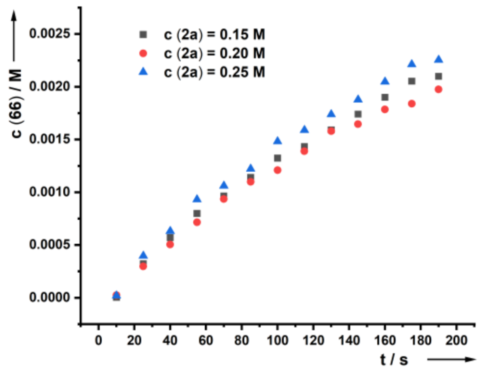

Figure S6. Kinetic profiles of different initial concentrations of dioxazolone **2a**.

Procedure for Kinetic profiles of different initial concentrations of dioxazolone 2a: In an oven-dried three-necked reactor was charged with alkyne **1c** (0.1 mmol) and dioxazolone **2a** (from 0.15 mmol to 0.25 mmol). After evacuated and backfilled with nitrogen three times, anhydrous THF (0.5 mL) was added. Then the silylzinc pivalate **3a** (2.0 equiv.) in anhydrous THF (0.5 mL) was added via a syringe at 25 °C. Afterwards, we used cooling bath to keep the reaction temperature at 15 °C and $CoBr_2$•DME (10 mol %) in anhydrous THF (20 μL) were added at 15 °C. After 30 minutes, the reaction quenched by EtOH. The yield was identified by HPLC analysis with biphenyl as an

internal standard after IR tests (every 5 seconds for one scan). All of these reactions exhibit zero-order kinetic behavior and the initial rate were obtained as the slopes of the straight lines.

iii) Kinetic profiles of different initial concentrations of silylzinc pivalate **3a**

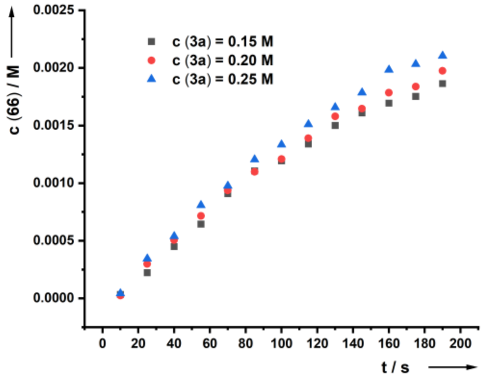

Figure S7. Kinetic profiles of different initial concentrations of silylzinc pivalate **3a**.

Procedure for Kinetic profiles of different initial concentrations of silylzinc pivalate 3a: In an oven-dried three-necked reactor was charged with alkyne **1c** (0.1 mmol) and dioxazolone **2a** (0.2 equiv.). After evacuated and backfilled with nitrogen three times, anhydrous THF (0.5 mL) was added. Then the silylzinc pivalate **3a** (from 0.15 mmol to 0.25 mmol) in anhydrous THF (0.5 mL) was added via a syringe at 25 °C. Afterwards, we used cooling bath to keep the reaction temperature at 15 °C and $CoBr_2$•DME (10 mol %) in anhydrous THF (20 μL) were added at 15 °C. After 30 minutes, the reaction quenched by EtOH. The yield was identified by HPLC analysis with biphenyl as an internal standard after IR tests (every 5 seconds for one scan). All of these reactions exhibit zero-order kinetic behavior and the initial rate were obtained as the slopes of the straight lines.

iv) Kinetic profiles of different initial concentrations of cobalt dibromo(1,2-dimethoxyethane)

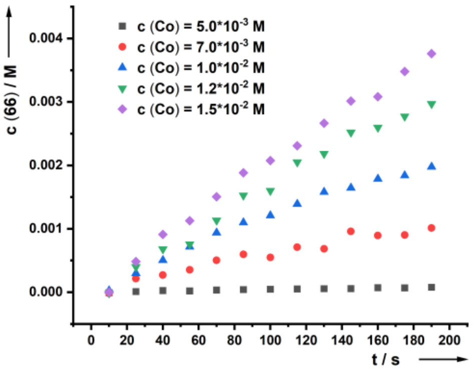

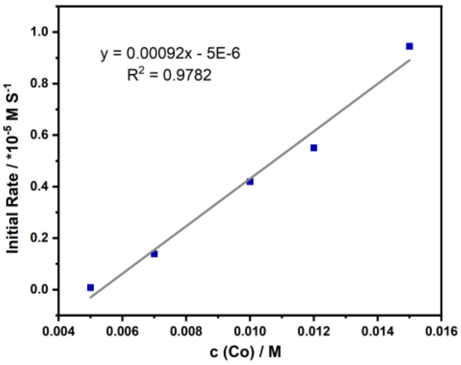

Figure S8. Kinetic profiles of different initial concentrations of cobalt dibromo(1,2-dimethoxyethane) loadings.

Procedure for Kinetic profiles of different initial concentrations of cobalt dibromo(1,2-dimethoxyethane): In an oven-dried three-necked reactor was charged with alkyne **1c** (0.1 mmol) and dioxazolone **2a** (0.2 equiv.). After evacuated and backfilled with nitrogen three times, anhydrous THF (0.5 mL) was added. Then the silylzinc pivalate **3a** (0.2 equiv.) in anhydrous THF (0.5 mL) was added via a syringe at 25 °C. Afterwards, we used cooling bath to keep the reaction temperature at 15 °C and $CoBr_2$•DME (from 5 mol % to 15 mol %) in anhydrous THF (20 μL) were added at 15 °C. After 30 minutes, the reaction quenched by EtOH. The yield was identified by HPLC analysis with biphenyl as an internal standard after IR tests (every 5 seconds for one scan). Plotting kobs vs catalyst loading displayed a linear relationship in **[Co]** catalyst, which suggested a first-order kinetic dependence in **[Co]**.

Preparation of Alkyne Substrates

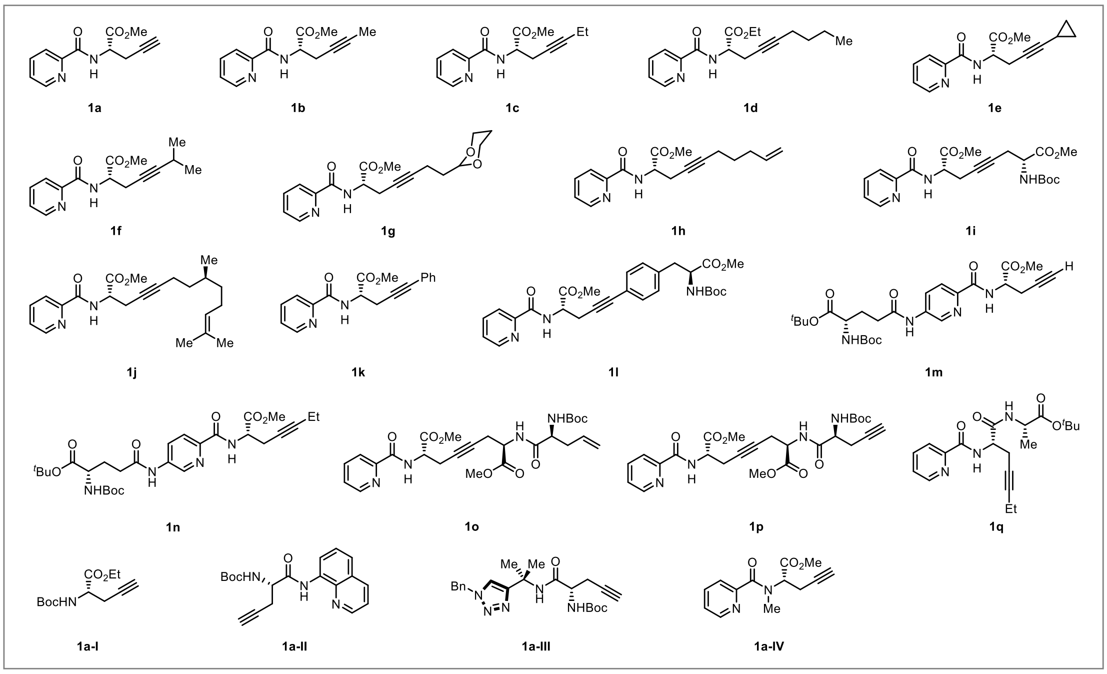

Figure S9. List of alkynes.

Typical procedure 1 (TP1) for the preparation of alkyne substrates 1a-1h, 1j, 1m-1n

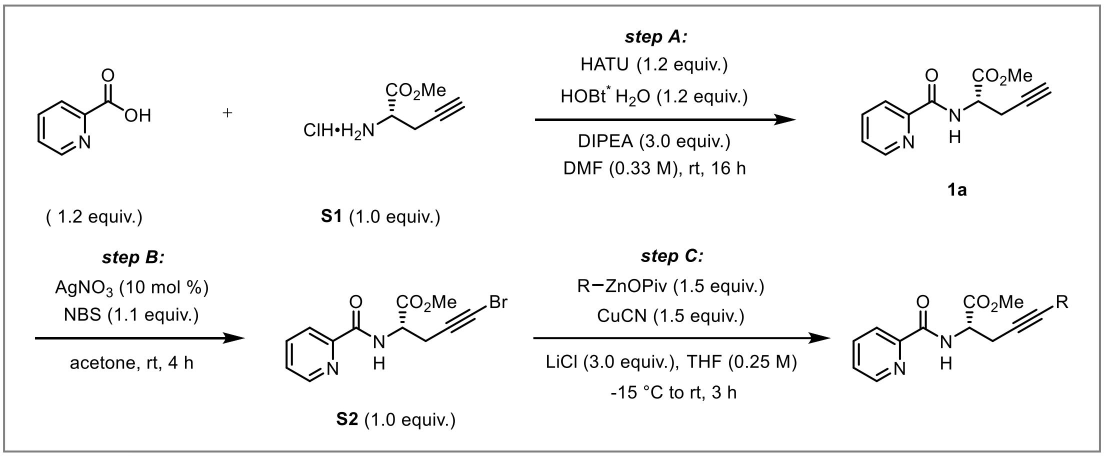

Figure S10. Typical procedure 1 (**TP1**) for the preparation of alkyne substrates **1a-1h**, **1j** and **1m**.

Amide coupling (step A):[1]

To a 100 mL Schlenk flask was added a mixture of picolinic acid (5.91 g, 48 mmol, 1.2 equiv.), HOBt hydrate (5.4 g, 40 mmol, 1.2 equiv.), and HATU (18.2 g, 48 mmol, 1.2 equiv.). The flask was evacuated and backfilled with argon for 3 times. The mixture was then dissolved in DMF (120 mL, 0.33 M) and stirred at room temperature until solution became homogeneous. Methyl (*S*)-2-aminopent-4-ynoate hydrochloride salt **S1** (6.54 g, 40 mmol, 1.0 equiv.) was added to the reaction mixture, followed by addition of DIPEA (15.5 g, 120 mmol, 3.0 equiv.). The mixture was stirred at room temperature overnight and then partitioned into a separatory funnel with EtOAc and water. The aqueous layer was extracted with EtOAc (100 mL × 3) and the combined organic layers were washed with saturated aqueous $NaHCO_3$ solution (50 mL × 1), water (100 mL × 3), and brine (50 mL × 1), then dried over anhydrous Na_2SO_4. The solution was then filtered and solvent was removed under reduced pressure via rotary evaporator. The crude product was purified by column chromatography (petroleum ether/EtOAc 4:1) on silica gel to yield the desired product **1a** (7.9 g, 85%) as a yellow solid.

Bromination of terminal alkyne (step B):

To a solution of alkyne (4.64 g, 20 mmol, 1.0 equiv.) in acetone (100 mL, 0.2 M), NBS (3.92 g, 22 mmol, 1.1 equiv.) and $AgNO_3$ (340 mg, 2 mmol, 10 mol %) were added. The resulting mixture was stirred under argon at room temperature for 4 h. After this period, the reaction mixture was filtered through a silica gel pad. The filtrate was concentrated under reduced pressure using a rotary evaporator and purified by flash column chromatography (petroleum ether/EtOAc = 5:1) on silica gel to afford the desired product **S2** (4.6 g, 74%) as a yellow solid

Negishi coupling (step C):

CuCN (1.5 equiv.) and LiCl (3.0 equiv.) were heated to 150 °C for 2 h and cooled to room temperature. Addition of THF (0.5 M) formed a soluble CuCN•2LiCl complex within 5 min. After cooling the Cu-complex to -15 °C, the organozinc pivalate reagent was added dropwise over 20 min. Then a solution of bromoacetylene **S2** (1.0 equiv.) in

dry THF (1.0 M) was added to the mixture. The mixture was stirred at room temperature for 3 h. After this period, saturated aqueous solution of $NaHCO_3$ was added and the mixture was filtered through a pad of silica. The aqueous phase was extracted with EtOAc (× 3). The combined organic phases were washed with brine, dried over anhydrous Na_2SO_4, and concentrated with a rotary evaporator. The crude mixture was purified by silica gel column chromatography (petroleum ether/EtOAc) to give propargylglycine-derived internal alkynes.

Typical procedure 2 (TP2) for the preparation of alkyne substrates 1i [2]

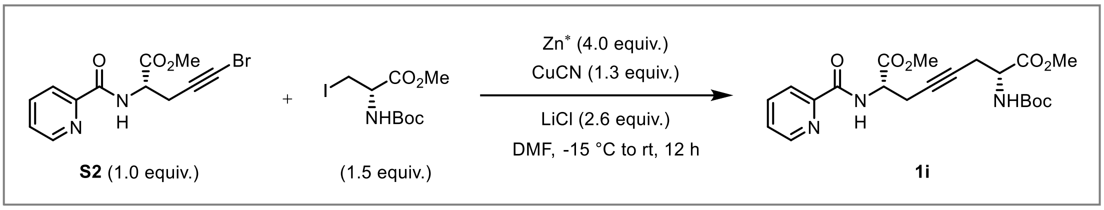

Figure S11. Typical procedure 2 (**TP2**) for the preparation of alkyne substrates **1i**.

Zinc dust (915 mg, 14 mmol, 4.0 equiv.) was weighed into a 100 mL flask, which was repeatedly evacuated (with heating using a heat gun) and flushed with argon. Dry DMF (5 mL) and 1,2-dibromoethane (100 μL, 1.1 mmol) were added and the flask was heated at 80°C for 40 min. The reaction mixture was allowed to cool to room temperature, trimethylsilyl chloride (40 μL, 0.35 mmol) was added and the resulting mixture was stirred vigorously for a further 30 min under argon. Then a solution of methyl (*S*)-2-[(*tert*-butoxycarbonyl)amino]-3-iodopropanoate (1.73 g, 5.3 mmol, 1.5 equiv.) in DMF (2 mL) was added and stirred at room temperature for 3 h more after which stirring was ceased to settle the zinc. CuCN (410 mg, 4.55 mmol, 1.3 equiv.) and LiCl (386 mg, 9.1mmol, 2.6 equiv.) were heated to 150 °C for 2 h and cooled to room temperature. Addition of DMF (8 mL) formed a soluble CuCN•2LiCl complex within 5 min. After cooling the Cu-complex to -15 °C, the organozinc reagent was added dropwise followed by the methyl (*S*)-5-bromo-2-(picolinamido)pent-4-ynoate (1.09 g, 3.5 mmol). The mixture was allowed to stir overnight at room temperature. After this period, saturated

aqueous solution of NH_4Cl was added and the mixture was filtered through a pad of silica. The aqueous layer was extracted with EtOAc (50 mL × 3) and the combined organic layers were washed with water (100 mL × 3), and brine (50 mL × 1), then dried over anhydrous Na_2SO_4. The solution was then filtered and solvent was removed under reduced pressure via rotary evaporator. The crude product was purified by column chromatography (petroleum ether/EtOAc = 2:1) on silica gel to yield the desired product **1i** (759 mg, 50 %) as a yellow oil.

Typical procedure 3 (TP3) for the preparation of alkyne substrates 1k-1l

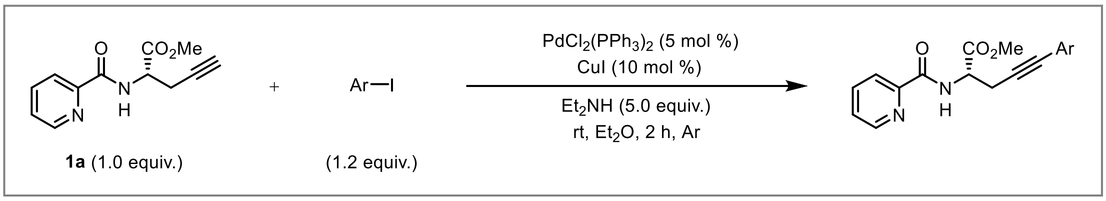

Figure S12. Typical procedure 3 (**TP3**) for the preparation of alkyne substrates **1k-1l**.

Sonogashira coupling:[3]

To a solution of the methyl (*S*)-2-(picolinamido)pent-4-ynoate **S2** (1.0 equiv.), aryl iodide (1.2 equiv.) and Et_2NH (5.0 equiv.) in Et_2O (0.1 M), CuI (10 mol %) and $PdCl_2(PPh_3)_2$ (5 mol %) were added. The mixture was stirred at room temperature under argon for 2 h. The reaction mixture was poured into a saturated aqueous solution of NH_4Cl. After phase separation, the aqueous layer was extracted with Et_2O (× 1). The combined organic layers were washed with brine, dried over Na_2SO_4 and concentrated in vacuo. The crude mixture was purified by silica gel column chromatography (petroleum ether/EtOAc) to give aryl alkynes.

Typical procedure 4 (TP4) for the preparation of alkyne substrates 1o-1p

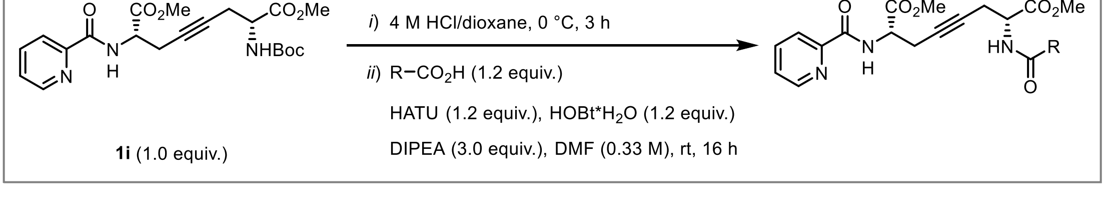

Figure S13. Typical procedure 4 (**TP4**) for the preparation of alkyne substrates **1o-1p**.

Boc-deprotection:

Compound **1i** (1.73 g, 4 mmol, 1.0 equiv.) was dissolved in 4 M HCl/dioxane (40 mL, 40 mmol, 10 equiv.) at 0 °C and stirred for 3 h. After completion, the solvent was evaporated to give a semi-oil residue. The resulting residue was dried in vacuo to afford the desired product (1.4 g, 95%) as a white hygroscopic solid without further purification

Amide coupling:

To a 100 mL Schlenk flask was added a mixture of amino acid (1.2 equiv.), HOBt hydrate (1.2 equiv.), and HATU (1.2 equiv.). The flask was evacuated and backfilled with argon for 3 times. The mixture was then dissolved in DMF (0.33 M) and stirred at room temperature until solution became homogeneous. The amino acid hydrochloride salt obtained from the previous step (1.0 equiv.) was added to the reaction mixture, followed by addition of DIPEA (3.0 equiv.). The mixture was stirred at room temperature overnight and then partitioned into a separatory funnel with EtOAc and water. The aqueous layer was extracted with EtOAc (× 3) and the combined organic layers were washed with saturated aqueous $NaHCO_3$ solution (x 1), water (× 3), and brine (× 1), then dried over anhydrous Na_2SO_4. The solution was then filtered and solvent was removed under reduced pressure via rotary evaporator. The crude product was purified by column chromatography (petroleum ether/EtOAc) on silica gel to yield the desired product.

Typical procedure 5 (TP5) for the preparation of alkyne substrates 1q

Hydrolysis of methyl ester: [4]

To a solution of alkyne **1c** (520 mg, 2 mmol, 1.0 equiv.) in dry MeCN (10 mL, 0.2 M), H_2O (0.2 mL, 2 vol %), Et_3N (834 mL, 6 mmol, 3.0 equiv.) and LiBr (1.74 g, 20 mmol, 10 equiv.) were added subsequently. The resulting mixture was stirred at room temperature for 2 h. H_2O and DCM were added. Then 1 M HCl was added to the aqueous solution to adjust the pH to 4, the aqueous layer was extracted with DCM (20 mL × 3). The combined organic layers were washed with brine, dried over anhydrous

Na_2SO_4, filtered, and concentrated under vacuum to afford the (*S*)-2-(picolinamido)hept-4-ynoic acid (468 mg, 95%) as a yellow solid without further purification.

Amide coupling:

To a 100 mL Schlenk flask was added a mixture of (*S*)-2-(picolinamido)hept-4-ynoic acid (1.0 equiv.), HOBt hydrate (1.0 equiv.), and HATU (1.2 equiv.). The flask was evacuated and backfilled with argon for 3 times. The mixture was then dissolved in DMF (0.33 M) and stirred at room temperature until solution became homogeneous. The H-Ala-O^{t}Bu•HCl (1.2 equiv.) was added to the reaction mixture, followed by addition of DIPEA (3.0 equiv.). The mixture was stirred at room temperature overnight and then partitioned into a separatory funnel with EtOAc and water. The aqueous layer was extracted with EtOAc (× 3) and the combined organic layers were washed with saturated aqueous $NaHCO_3$ solution (× 1), water (× 3), and brine (× 1), then dried over anhydrous Na_2SO_4. The solution was then filtered and solvent was removed under reduced pressure via rotary evaporator. The crude product was purified by column chromatography (petroleum ether/EtOAc) on silica gel to yield the desired product.

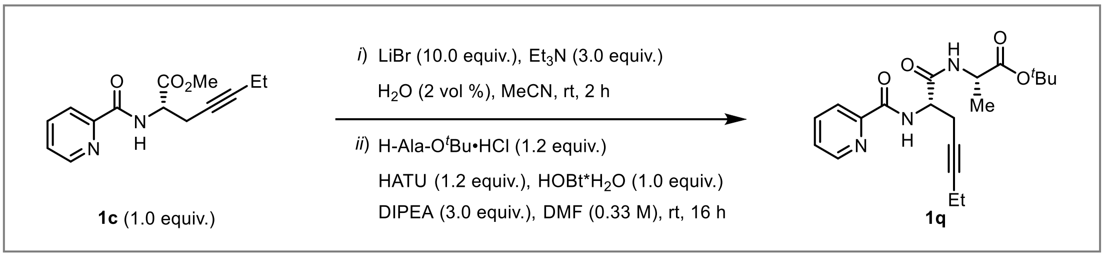

Figure S14. Typical procedure 5 (**TP5**) for the preparation of alkyne substrates **1q**.

Typical procedure 6 (TP6) for the preparation of alkyne substrates 1a-II, 1a-III

Amide coupling:

To a 100 mL Schlenk flask was added a mixture of (*S*)-2-[(*tert*-butoxycarbonyl)amino]pent-4-ynoic acid (1.0 equiv.), HOBt hydrate (1.0 equiv.), and HATU (1.2 equiv.). The flask was evacuated and backfilled with argon for 3 times. The mixture was then dissolved in DMF (0.33 M) and stirred at room temperature until

solution became homogeneous. R−NH_2 (1.2 equiv.) was added to the reaction mixture, followed by addition of DIPEA (3.0 equiv.). The mixture was stirred at room temperature overnight and then partitioned into a separatory funnel with EtOAc and water. The aqueous layer was extracted with EtOAc (× 3) and the combined organic layers were washed with saturated aqueous $NaHCO_3$ solution (× 1), water (× 3), and brine (× 1), then dried over anhydrous Na_2SO_4. The solution was then filtered and solvent was removed under reduced pressure via rotary evaporator. The crude product was purified by column chromatography (petroleum ether/EtOAc) on silica gel to yield the desired product.

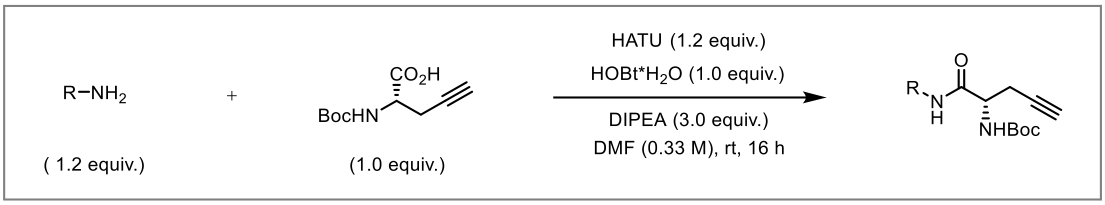

Figure S15. Typical procedure 6 (**TP6**) for the preparation of alkyne substrates **1a-II** and **1a-III**.

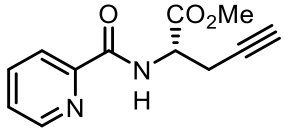

Methyl (*S*)-2-(picolinamido)pent-4-ynoate (1a)

^{1}H NMR (400 MHz, $CDCl_3$) δ = 8.71 (d, J = 8.0 Hz, 1H), 8.57 – 8.46 (m, 1H), 8.09 (dt, J = 7.8, 1.0 Hz, 1H), 7.77 (td, J = 7.7, 1.7 Hz, 1H), 7.43 – 7.33 (m, 1H), 4.88 (dt, J = 8.4, 5.1 Hz, 1H), 3.74 (s, 3H), 2.85 – 2.77 (m, 2H), 2.04 (t, J = 2.7 Hz, 1H). ^{13}C NMR (101 MHz, $CDCl_3$) δ = 170.6, 164.1, 149.1, 148.4, 137.3, 126.5, 122.3, 78.4, 71.7, 52.8, 50.8, 22.5. HR-MS (ESI) m/z calcd for $C_{12}H_{12}N_2NaO_3$ [M+Na$^+$]: 255.0740, found: 255.0743.

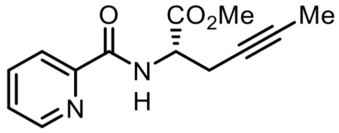

The general procedure **TP1** was followed on 5 mmol scale and purification by column chromatography (petroleum ether/EtOAc 5:1) yielded **1b** (763 mg, 62%, yield of step C) as a yellow oil. ^{1}H NMR (400 MHz, $CDCl_3$) δ = 8.71 (d, J = 7.8 Hz, 1H), 8.60 (d, J = 4.2 Hz, 1H), 8.16 (d, J = 7.8 Hz, 1H), 7.83 (td, J = 7.7, 1.7 Hz, 1H), 7.48 – 7.39 (m, 1H), 4.94 – 4.84 (m, 1H), 3.79 (s, 3H), 2.89 – 2.70 (m, 2H), 1.77 (t, J = 2.5 Hz, 3H). ^{13}C NMR (101 MHz, $CDCl_3$) δ = 171.1, 164.2, 149.4, 148.4, 137.3, 126.5, 122.4, 79.4, 73.0, 52.7, 51.3, 22.9, 3.6. HR-MS (ESI) m/z calcd for $C_{13}H_{14}N_2NaO_3$ [M+Na$^+$]: 269.0897, found: 269.0896.

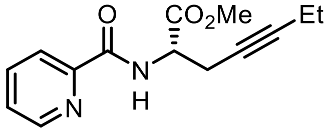

Methyl (*S*)-2-(picolinamido)hept-4-ynoate (1c)

The general procedure **TP1** was followed on 30.8 mmol scale and purification by column chromatography (petroleum ether/EtOAc 5:1) yielded **1c** (5.2 g, 63%, yield of step C) as a yellow oil. ^{1}H NMR (400 MHz, $CDCl_3$) δ = 8.75 (d, J = 7.9 Hz, 1H), 8.61 (dd, J = 4.0, 0.7 Hz, 1H), 8.18 (d, J = 7.8 Hz, 1H), 7.96 – 7.77 (m, 1H), 7.54 – 7.40 (m, 1H), 4.98 – 4.81 (m, 1H), 3.81 (s, 3H), 2.93 – 2.72 (m, 2H), 2.25 – 2.06 (m, 2H), 1.11 (t, J = 7.5 Hz, 3H). ^{13}C NMR (101 MHz, $CDCl_3$) δ = 171.6, 169.4, 164.3, 149.2, 148.3, 137.2, 126.4, 122.3, 85.5, 81.9, 74.1, 52.1, 49.0, 27.9, 22.7, 18.6, 13.9, 12.3. HR-MS (ESI) m/z calcd for $C_{14}H_{16}N_2NaO_3$ [M+Na$^+$]: 283.1053, found: 283.1055.

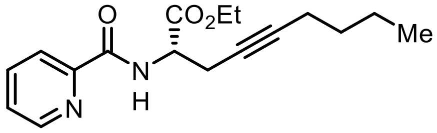

Ethyl (*S*)-2-(picolinamido)non-4-ynoate (1d)

The general procedure **TP1** was followed on 3 mmol scale and purification by column chromatography (petroleum ether/EtOAc 5:1) yielded **1d** (544 mg, 60%, yield of step C) as a yellow oil. ^{1}H NMR (400 MHz, $CDCl_3$) δ = 8.76 (d, J = 8.0 Hz, 1H), 8.60 (d, J = 4.1 Hz, 1H), 8.18 (d, J = 7.8 Hz, 1H), 7.91 – 7.79 (m, 1H), 7.48 – 7.40 (m, 1H), 4.95 – 4.79 (m, 1H), 4.33 – 4.20 (m, 2H), 2.95 – 2.75 (m, 2H), 2.24 – 2.10 (m, 2H), 1.49 – 1.36 (m, 4H), 1.31 (t, J = 7.1 Hz, 3H), 0.91 – 0.83 (m, 3H). ^{13}C NMR (151 MHz, $CDCl_3$)

δ = 170.6, 164.1, 149.5, 148.4, 137.3, 126.4, 122.3, 84.1, 74.0, 61.7, 51.4, 30.9, 23.1, 21.8, 18.4, 14.3, 13.7. HR-MS (ESI) m/z calcd for $C_{17}H_{22}N_2NaO_3$ [M+Na$^+$]: 325.1523, found: 325.1520.

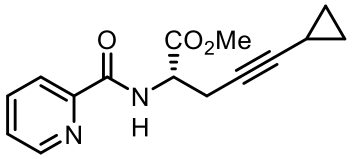

Methyl (*S*)-5-cyclopropyl-2-(picolinamido)pent-4-ynoate (1e)

The general procedure **TP1** was followed on 5 mmol scale and purification by column chromatography (petroleum ether/EtOAc 6:1) yielded **1e** (926 mg, 68%, yield of step C) as a yellow oil. ^{1}H NMR (400 MHz, $CDCl_3$) δ = 8.70 (s, 1H), 8.59 (s, 1H), 8.15 (d, *J* = 3.7 Hz, 1H), 7.83 (dd, *J* = 5.5, 3.8 Hz, 1H), 7.43 (d, *J* = 1.7 Hz, 1H), 4.85 (d, *J* = 3.6 Hz, 1H), 3.78 (dd, *J* = 5.8, 4.1 Hz, 3H), 2.78 (dd, *J* = 5.6, 3.6 Hz, 2H), 1.16 (s, 1H), 0.67 (dd, *J* = 21.2, 17.4 Hz, 4H). ^{13}C NMR (101 MHz, $CDCl_3$) δ = 171.1, 164.1, 149.4, 148.4, 137.3, 126.5, 122.4, 87.4, 69.1, 52.7, 51.3, 23.1, 8.3, 8.2, -0.3. HR-MS (ESI) m/z calcd for $C_{15}H_{16}N_2NaO_3$ [M+Na$^+$]: 295.1053, found: 295.1056.

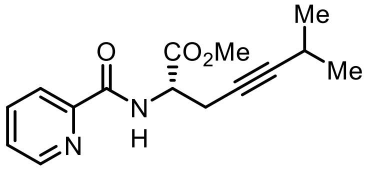

Methyl (*S*)-6-methyl-2-(picolinamido)hept-4-ynoate (1f)

The general procedure **TP1** was followed on 2.5 mmol scale and purification by column chromatography (petroleum ether/EtOAc 6:1) yielded **1f** (440 mg, 64%, yield of step C) as a yellow oil. ^{1}H NMR (400 MHz, $CDCl_3$) δ = 8.75 (d, *J* = 8.1 Hz, 1H), 8.58 (d, *J* = 4.3 Hz, 1H), 8.16 (d, *J* = 7.8 Hz, 1H), 7.96 – 7.76 (m, 1H), 7.54 – 7.34 (m, 1H), 4.96 – 4.82 (m, 1H), 3.78 (s, 3H), 2.90 – 2.72 (m, 2H), 2.56 – 2.43 (m, 1H), 1.12 (d, *J* = 6.9 Hz, 6H). ^{13}C NMR (101 MHz, $CDCl_3$) δ = 171.0, 164.0, 149.3, 148.3, 137.2, 126.4, 122.2, 90.0, 73.1, 52.5, 51.2, 23.1, 22.9, 20.5. HR-MS (ESI) m/z calcd for $C_{15}H_{18}N_2NaO_3$ [M+Na$^+$]: 297.1210, found: 297.1212.

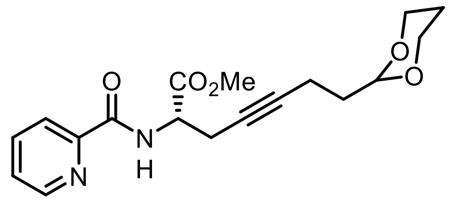

Methyl (*S*)-7-(1,3-dioxan-2-yl)-2-(picolinamido)hept-4-ynoate (1g)

The general procedure **TP1** was followed on 2.5 mmol scale and purification by column chromatography (petroleum ether/EtOAc 3:2) yielded **1g** (558 mg, 64%, yield of step C) as a yellow oil. ^{1}H NMR (400 MHz, $CDCl_3$) δ = 8.73 (d, J = 8.2 Hz, 1H), 8.58 (d, J = 4.1 Hz, 1H), 8.14 (d, J = 7.8 Hz, 1H), 7.87 – 7.73 (m, 1H), 7.55 – 7.38 (m, 1H), 4.87 (dt, J = 8.6, 5.0 Hz, 1H), 4.61 (t, J = 5.3 Hz, 1H), 4.15 – 3.98 (m, 2H), 3.82 – 3.67 (m, 5H), 2.91 – 2.72 (m, 2H), 2.30 – 2.16 (m, 2H), 2.07 – 1.96 (m, 1H), 1.79 – 1.65 (m, 2H), 1.28 (dd, J = 12.3, 1.1 Hz, 1H). ^{13}C NMR (101 MHz, $CDCl_3$) δ = 171.0, 164.1, 149.3, 148.4, 137.3, 126.4, 122.3, 100.7, 83.1, 74.2, 66.8, 52.6, 51.2, 34.1, 25.8, 22.9, 13.6. HR-MS (ESI) m/z calcd for $C_{18}H_{22}N_2NaO_5$ [M+Na$^+$]: 369.1421, found: 369.1423.

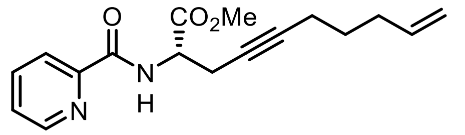

Methyl (*S*)-2-(picolinamido)dec-9-en-4-ynoate (1h)

The general procedure **TP1** was followed on 5 mmol scale and purification by column chromatography (petroleum ether/EtOAc 6:1) yielded **1h** (930 mg, 62%, yield of step C) as a yellow oil. ^{1}H NMR (400 MHz, $CDCl_3$) δ = 8.74 (d, J = 7.5 Hz, 1H), 8.55 (s, 1H), 8.13 (d, J = 7.7 Hz, 1H), 7.81 (t, J = 7.6 Hz, 1H), 7.40 (s, 1H), 5.72 (dq, J = 11.8, 7.5 Hz, 1H), 5.05 – 4.82 (m, 3H), 3.76 (s, 3H), 2.92 – 2.71 (m, 2H), 2.10 (dd, J = 13.8, 6.8 Hz, 4H), 1.59 – 1.46 (m, 2H). ^{13}C NMR (101 MHz, $CDCl_3$) δ = 171.0, 164.0, 149.3, 148.3, 137.9, 137.2, 126.4, 122.2, 115.1, 83.7, 74.2, 52.6, 51.2, 32.6, 27.9, 22.9, 18.0. HR-MS (ESI) m/z calcd for $C_{17}H_{20}N_2NaO_3$ [M+Na$^+$]: 323.1366, found: 323.1362.

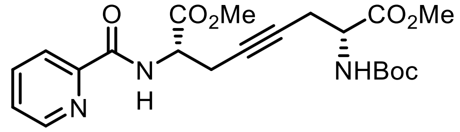

Dimethyl (2*R*,7*S*)-2-[(*tert*-butoxycarbonyl)amino]-7-(picolinamido)oct-4-ynedioate (1i)

The general procedure **TP2** was followed on 3.5 mmol scale and purification by column chromatography (petroleum ether/EtOAc 2:1) yielded **1i** (759 mg, 50 %) as a yellow oil. ^{1}H NMR (400 MHz, $CDCl_3$) δ = 8.69 (d, J = 8.1 Hz, 1H), 8.62 (d, J = 4.3 Hz, 1H), 8.15 (d, J = 7.8 Hz, 1H), 7.90 – 7.78 (m, 1H), 7.50 – 7.39 (m, 1H), 5.72 (d, J = 8.3 Hz, 1H), 4.99 – 4.81 (m, 1H), 4.58 – 4.35 (m, 1H), 3.80 (s, 3H), 3.70 (s, 3H), 2.88 – 2.61 (m, 4H), 1.40 (s, 9H). ^{13}C NMR (101 MHz, $CDCl_3$) δ = 171.1, 170.9, 164.1, 155.2, 149.3, 148.4, 137.3, 126.5, 122.4, 79.9, 78.1, 77.6, 52.8, 52.5, 52.2, 51.1, 28.3, 23.2, 23.0. HR-MS (ESI) m/z calcd for $C_{21}H_{27}N_3NaO_7$ [M+Na$^+$]: 456.1741, found: 456.1744.

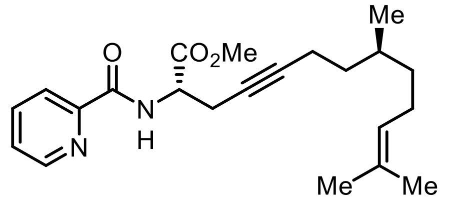

Methyl (2*S*,8*S*)-8,12-dimethyl-2-(picolinamido)tridec-11-en-4-ynoate (1j)

The general procedure **TP1** was followed on 3 mmol scale and purification by column chromatography (petroleum ether/EtOAc 6:1) yielded **1a** (660 mg, 60%, yield of step C) as a yellow oil. ^{1}H NMR (400 MHz, $CDCl_3$) δ = 8.73 (d, J = 8.2 Hz, 1H), 8.63 – 8.52 (m, 1H), 8.16 (d, J = 7.8 Hz, 1H), 7.90 – 7.78 (m, 1H), 7.50 – 7.39 (m, 1H), 5.15 – 5.00 (m, 1H), 4.97 – 4.82 (m, 1H), 3.78 (s, 3H), 2.96 – 2.71 (m, 2H), 2.26 – 2.07 (m, 2H), 1.98 – 1.84 (m, 2H), 1.64 (s, 3H), 1.55 (s, 3H), 1.54 – 1.44 (m, 2H), 1.32 – 1.22 (m, 2H), 1.18 – 1.05 (m, 1H), 0.83 (d, J = 6.5 Hz, 3H). ^{13}C NMR (101 MHz, $CDCl_3$) δ = 171.1, 164.1, 149.4, 148.3, 137.2, 131.1, 126.4, 124.8, 122.3, 84.3, 73.8, 52.6, 51.3, 36.8, 35.9, 31.6, 25.7, 25.4, 23.0, 19.1, 17.6, 16.5. HR-MS (ESI) m/z calcd for $C_{22}H_{30}N_2NaO_3$ [M+Na$^+$]: 393.2149, found: 393.2146.

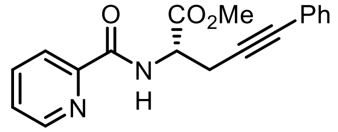

Methyl (*S*)-5-phenyl-2-(picolinamido)pent-4-ynoate (1k)

The general procedure **TP3** was followed on 5 mmol scale and purification by column chromatography (petroleum ether/EtOAc 4:1) yielded **1k** (1.31 g, 85%) as a yellow oil. ^{1}H NMR (400 MHz, $CDCl_3$) δ = 8.85 (d, J = 8.1 Hz, 1H), 8.65 – 8.56 (m, 1H), 8.19 (dt, J = 7.8, 1.0 Hz, 1H), 7.90 – 7.79 (m, 1H), 7.50 – 7.42 (m, 1H), 7.42 – 7.35 (m, 2H), 7.31 – 7.26 (m, 3H), 5.08 – 4.99 (m, 1H), 3.84 (s, 3H), 3.19 – 3.03 (m, 2H). ^{13}C NMR (101 MHz, $CDCl_3$) δ = 170.9, 164.2, 149.2, 148.4, 137.3, 131.7, 128.2, 128.1, 126.5, 123.0, 122.3, 83.8, 52.8, 51.2, 23.6. HR-MS (ESI) m/z calcd for $C_{18}H_{16}N_2NaO_3$ [M+Na$^+$]: 331.1053, found: 331.1051.

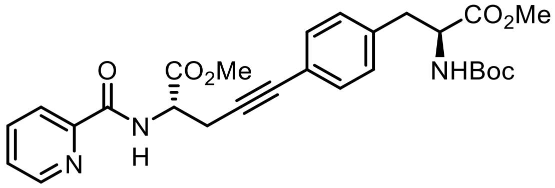

Methyl (*S*)-5-{{4-{(*S*)-2-[(*tert*-butoxycarbonyl)amino]-3-methoxy-3-oxopropyl}phenyl}}-2-(picolinamido)pent-4-ynoate (1l)

The general procedure **TP3** was followed on 5 mmol scale and purification by column chromatography (petroleum ether/EtOAc 5:2) yielded **1l** (2.42 g, 95%) as a yellow oil. ^{1}H NMR (400 MHz, $CDCl_3$) δ = 8.82 (d, J = 8.3 Hz, 1H), 8.58 (d, J = 4.6 Hz, 1H), 8.16 (d, J = 7.8 Hz, 1H), 7.83 (td, J = 7.7, 1.6 Hz, 1H), 7.42 (dd, J = 6.8, 4.9 Hz, 1H), 7.30 (d, J = 8.1 Hz, 2H), 7.03 (d, J = 8.0 Hz, 2H), 5.00 (dt, J = 8.7, 5.1 Hz, 2H), 4.54 (d, J = 7.4 Hz, 1H), 3.81 (s, 3H), 3.68 (s, 3H), 3.18 – 2.94 (m, 4H), 1.39 (s, 9H). ^{13}C NMR (151 MHz, $CDCl_3$) δ = 172.2, 170.9, 164.2, 155.1, 149.3, 148.4, 137.3, 136.3, 131.9, 129.2, 126.5, 122.3, 121.8, 84.0, 83.6, 80.0, 54.3, 52.8, 52.3, 51.2, 38.2, 28.3, 23.7. HR-MS (ESI) m/z calcd for $C_{27}H_{31}N_3NaO_7$ [M+Na$^+$]: 532.2054, found: 532.2052.

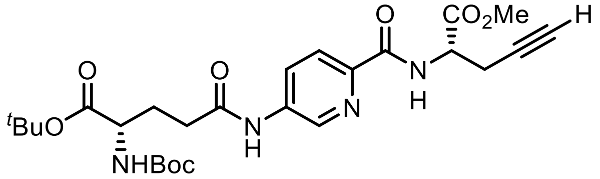

Methyl (*S*)-2-{5-[(S)-5-(*tert*-butoxy)-4-[(*tert*-butoxycarbonyl)amino]-5-oxopentanamido]picolinamido}pent-4-ynoate (1m)

The general procedure **TP1** step A was followed. Purification by column chromatography (petroleum ether/EtOAc 1:1) yielded **1m** as a yellow solid. ^{1}H NMR (400 MHz, $CDCl_3$) δ = 9.79 (s, 1H), 8.75 (s, 1H), 8.64 (d, J = 8.3 Hz, 1H), 8.34 (d, J = 7.1 Hz, 1H), 8.13 (d, J = 8.5 Hz, 1H), 5.48 (d, J = 7.6 Hz, 1H), 4.95 (dt, J = 8.5, 5.0 Hz, 1H), 4.20 (t, J = 7.4 Hz, 1H), 3.82 (s, 3H), 2.96 – 2.83 (m, 2H), 2.51 (t, J = 6.4 Hz, 2H), 2.30-2.27 (m, 1H), 2.08 (t, J = 2.6 Hz, 1H), 1.90-1.84 (m, 1H), 1.49 (s, 9H), 1.46 (s, 9H). ^{13}C NMR (101 MHz, $CDCl_3$) δ = 171.5, 171.0, 164.1, 144.1, 139.7, 126.8, 123.0, 83.2, 81.1, 78.6, 77.5, 77.2, 76.8, 71.8, 53.2, 52.9, 51.0, 34.3, 28.4, 28.1, 22.7. HR-MS (ESI) m/z calcd for $C_{26}H_{37}N_4O_8$ [M+H$^+$]: 533.2606, found: 533.2602.

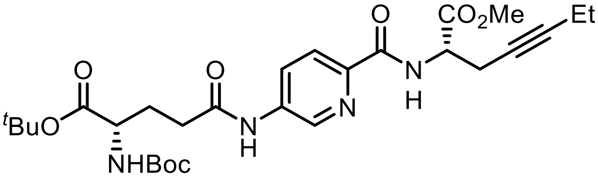

Methyl (*S*)-2-{{5-{(*S*)-5-(*tert*-butoxy)-4-[(*tert*-butoxycarbonyl)amino]-5-oxopentanamido}picolinamido}}hept-4-ynoate (1n)

The general procedure **TP1** and **TP5** was followed. Purification by column chromatography (petroleum ether/EtOAc 2:1) yielded **1n** as a white solid. ^{1}H NMR (400 MHz, $CDCl_3$) δ = 9.65 (s, 1H), 8.67 (s, 1H), 8.57 (d, J = 8.0 Hz, 1H), 8.23 (d, J = 7.9 Hz, 1H), 8.03 (d, J = 8.0 Hz, 1H), 5.51 (d, J = 7.8 Hz, 1H), 4.90 – 4.76 (m, 1H), 4.13 (s, 1H), 3.74 (s, 3H), 2.85 – 2.70 (m, 2H), 2.45 (d, J = 6.0 Hz, 2H), 2.21 (d, J = 6.7 Hz, 1H), 2.09 (d, J = 7.3 Hz, 2H), 1.88 (s, 1H), 1.39 (s, 18H), 1.05 (t, J = 7.4 Hz, 3H). ^{13}C NMR (101 MHz, $CDCl_3$) δ = 171.4, 171.3, 171.1, 164.0, 156.6, 143.9, 139.5, 138.1, 126.6, 122.7, 85.6, 82.7, 80.5, 73.1, 53.3, 52.6, 51.2, 33.8, 29.8, 28.3, 27.9, 22.8, 14.0, 12.3. HR-MS (ESI) m/z calcd for $C_{28}H_{40}N_4NaO_8$ [M+Na$^+$]: 583.2738, found: 583.2735.

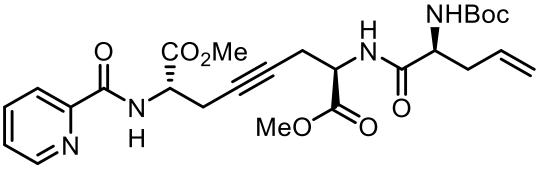

Methyl (*S*)-2-(picolinamido)pent-4-ynoate (1o)

The general procedure **TP4** was followed on 2 mmol scale and purification by column chromatography (petroleum ether/EtOAc 2:1) yielded **1o** (867 mg, 78%, 2 steps) as a yellow oil. ^{1}H NMR (600 MHz, $CDCl_3$) δ = 8.67 (d, J = 8.5 Hz, 1H), 8.59 (dd, J = 2.8, 1.8 Hz, 1H), 8.17 (d, J = 7.2 Hz, 1H), 7.82 (t, J = 7.7 Hz, 1H), 7.50 – 7.37 (m, 1H), 7.20 (s, 1H), 5.87 – 5.68 (m, 1H), 5.39 (d, J = 6.2 Hz, 1H), 5.09 (dd, J = 21.7, 13.6 Hz, 2H), 4.98 – 4.89 (m, 1H), 4.66 (dd, J = 7.8, 4.2 Hz, 1H), 4.39 (s, 1H), 3.80 (d, J = 2.1 Hz, 3H), 3.68 (s, 3H), 2.80 – 2.68 (m, 3H), 2.68 – 2.55 (m, 2H), 2.53 – 2.41 (m, 1H), 1.37 (s, 9H). ^{13}C NMR (151 MHz, $CDCl_3$) δ = 171.5, 171.1, 170.6, 164.3, 155.5, 149.2, 148.4, 137.4, 133.2, 126.6, 122.5, 118.6, 79.7, 78.0, 77.8, 53.7, 52.9, 52.6, 51.2, 50.8, 37.3, 28.3, 23.2, 22.5. HR-MS (ESI) m/z calcd for $C_{26}H_{34}N_4NaO_8$ [M+Na$^+$]: 553.2269, found: 553.2265.

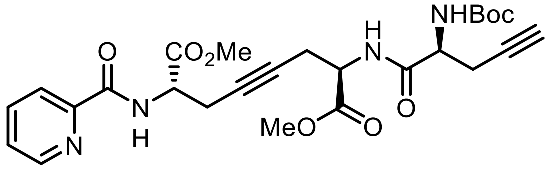

Dimethyl (2*R*,7*S*)-2-{(*S*)-2-[(*tert*-butoxycarbonyl)amino]pent-4-ynamido}-7-(picolinamido)oct-4-ynedioate (1p)

The general procedure **TP4** was followed on 1.15 mmol scale and purification by column chromatography (petroleum ether/EtOAc 3:2) yielded **1p** (539 mg, 85%, 2 steps) as a white solid. ^{1}H NMR (400 MHz, $CDCl_3$) δ = 8.67 (d, J = 8.5 Hz, 1H), 8.62 (d, J = 4.3 Hz, 1H), 8.18 (d, J = 7.8 Hz, 1H), 7.88 – 7.80 (m, 1H), 7.48 – 7.41 (m, 1H), 7.33 (d, J = 7.2 Hz, 1H), 5.70 (d, J = 7.6 Hz, 1H), 5.01 – 4.87 (m, 1H), 4.76 – 4.65 (m, 1H), 4.52 (d, J = 6.2 Hz, 1H), 3.81 (s, 3H), 3.70 (s, 3H), 2.80 – 2.65 (m, 6H), 2.07 (t, J = 2.4 Hz, 1H), 1.40 (s, 9H). ^{13}C NMR (151 MHz, $CDCl_3$) δ = 171.1, 170.4, 170.2, 164.3, 155.4, 149.2, 148.4, 137.5, 126.6, 122.6, 80.1, 79.4, 78.0, 77.7, 71.5, 53.0, 52.8, 52.7, 51.2, 51.0, 28.3, 23.3, 23.0, 22.6. HR-MS (ESI) m/z calcd for $C_{26}H_{32}N_4NaO_8$ [M+Na$^+$]: 551.2112, found: 551.2115.

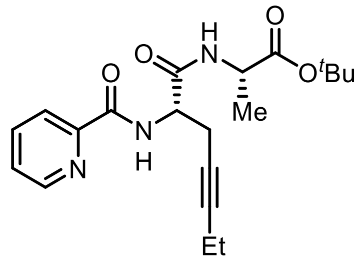

tert-Butyl [(_S_)-2-(picolinamido)hept-4-ynoyl]-_L_-alaninate (1q)

The general procedure **TP5** was followed on 1.5 mmol scale and purification by column chromatography (petroleum ether/EtOAc 2:1) yielded **1q** (426 mg, 76%, 2 steps) as a yellow oil. ^{1}H NMR (400 MHz, $CDCl_3$) δ = 8.74 (d, J = 8.0 Hz, 1H), 8.56 (d, J = 4.6 Hz, 1H), 8.15 (d, J = 7.8 Hz, 1H), 7.89 – 7.75 (m, 1H), 7.46 – 7.36 (m, 1H), 7.02 (d, J = 6.8 Hz, 1H), 4.70 (td, J = 7.6, 5.3 Hz, 1H), 4.44 (p, J = 7.1 Hz, 1H), 2.95 – 2.81 (m, 1H), 2.71 – 2.52 (m, 1H), 2.17 – 2.10 (m, 2H), 1.41 (s, 9H), 1.36 (d, J = 7.1 Hz, 3H), 1.07 (t, J = 7.5 Hz, 3H). ^{13}C NMR (101 MHz, $CDCl_3$) δ = 171.7, 169.5, 164.4, 149.3, 148.4, 137.3, 126.5, 122.3, 85.6, 82.0, 74.2, 52.2, 49.1, 27.9, 22.8, 18.6, 14.0, 12.4. HR-MS (ESI) m/z calcd for $C_{20}H_{27}N_3NaO_4$ [M+Na$^+$]: 396.1894, found: 396.1897.

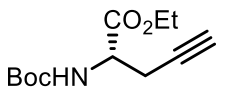

Ethyl (_S_)-2-[(_tert_-butoxycarbonyl)amino]pent-4-ynoate (1a-I)

Following a modified literature procedure,[5] _N_-(tert-Butoxycarbonyl)-L-propargylglycine (1.06 g, 5 mmol, 1.0 equiv.), 1-(3'-dimethylaminopropyl)-3-ethylcarbodiimide hydrochloride (1.05 g, 5.5 mmol, 1.1 equiv.), 4-dimethylaminopyridine (122 mg, 1.0 mmol, 0.2 equiv.) and ethanol (1.15 g, 25 mmol, 5.0 equiv.) were dissolved in dry DCM (25 mL) and stirred at room temperature for 3 h. Purification by column chromatography (petroleum ether/EtOAc 10:1) yielded **1a-I** (809 mg, 67%) as a colorless oil. ^{1}H NMR (400 MHz, $CDCl_3$) δ = 5.35 (d, J = 8.0 Hz, 1H), 4.39 – 4.27 (m, 1H), 4.18 – 4.09 (m, 2H), 2.68 – 2.57 (m, 2H), 1.96 (t, J = 2.5 Hz, 1H), 1.35 (s, 9H), 1.19 (t, J = 7.1 Hz, 3H). ^{13}C NMR (101 MHz, $CDCl_3$) δ 170.5, 155.0, 79.8, 78.5, 71.5, 61.5, 51.9, 28.1, 22.6, 14.0. HR-MS (ESI) m/z calcd for $C_{12}H_{19}NNaO_4$ [M+Na$^+$]: 264.1206, found: 264.1209.

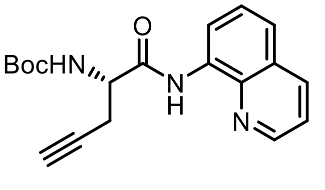

***tert*-Butyl (*S*)-[1-oxo-1-(quinolin-8-ylamino)pent-4-yn-2-yl]carbamate (1a-II)**

The general procedure **TP6** was followed on 6 mmol scale and purification by column chromatography (petroleum ether/EtOAc 10:1) yielded **1a-II** (1.57 g, 77%) as a white solid. ^{1}H NMR (400 MHz, $CDCl_3$) δ = 10.54 (s, 1H), 8.85 – 8.72 (m, 2H), 8.14 (dd, J = 8.3, 1.6 Hz, 1H), 7.61 – 7.47 (m, 2H), 7.44 (dd, J = 8.3, 4.2 Hz, 1H), 5.57 (s, 1H), 4.64 (s, 1H), 3.00 (d, J = 15.9 Hz, 1H), 2.86 – 2.70 (m, 1H), 2.09 (t, J = 2.6 Hz, 1H), 1.51 (s, 9H). ^{13}C NMR (101 MHz, $CDCl_3$) δ = 168.7, 155.4, 148.4, 138.7, 136.3, 134.0, 128.0, 127.3, 122.1, 121.7, 116.7, 80.7, 79.2, 72.1, 54.0, 28.4, 22.6. HR-MS (ESI) m/z calcd for $C_{19}H_{21}N_3NaO_3$ [M+Na$^+$]: 362.1475, found: 362.1472.

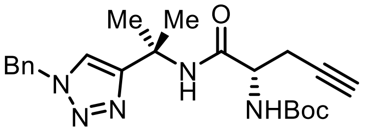

***tert*-Butyl (*S*)-{{1-{[2-(1-benzyl-1*H*-1,2,3-triazol-4-yl)propan-2-yl]amino}-1-oxopent-4-yn-2-yl}}carbamate (1a-III)**

The general procedure **TP6** was followed on 4 mmol scale and purification by column chromatography (petroleum ether/EtOAc 2:1) yielded **1a-III** (1.15 g, 70%) as a yellow solid. ^{1}H NMR (400 MHz, $CDCl_3$) δ = 7.42 (s, 1H), 7.34 (t, J = 6.0 Hz, 3H), 7.27 – 7.22 (m, 2H), 6.87 (s, 1H), 5.47 (s, 2H), 5.30 (s, 1H), 4.19 (s, 1H), 2.76 – 2.64 (m, 1H), 2.57 – 2.45 (m, 1H), 1.94 (t, J = 2.5 Hz, 1H), 1.72 (d, J = 4.1 Hz, 6H), 1.42 (s, 9H). ^{13}C NMR (101 MHz, $CDCl_3$) δ = 169.2, 153.4, 134.7, 129.0, 128.6, 128.0, 120.3, 80.4, 79.5, 71.6, 54.1, 53.3, 51.8, 28.2, 28.0, 27.8, 22.4. HR-MS (ESI) m/z calcd for $C_{22}H_{29}N_5NaO_3$ [M+Na$^+$]: 434.2163, found: 434.2161.

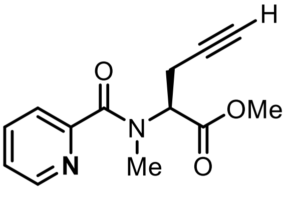

Methyl (*S*)-2-(*N*-methylpicolinamido)pent-4-ynoate (1a-IV)

A solution of **1a** (116 mg, 0.5 mmol, 1.0 equiv.) in DMF (10 mL) was cooled to 0 °C, then MeI (142 mg, 1.0 mmol, 2.0 equiv) and KOH (112 mg, 2.0 mmol, 4.0 equiv) were added. The mixture was stirred at room temperature for 12 h and then partitioned into a separatory funnel with EtOAc and water. The aqueous layer was extracted with EtOAc (10 mL x 3) and the combined organic layers were washed with water (10 mL × 3), and brine (10 mL × 1), then dried over anhydrous Na_2SO_4. The solution was then filtered and solvent was removed under reduced pressure via rotary evaporator. The crude residue was was purified by column chromatography (petroleum ether/EtOAc 2:1) yielded **1a-IV** (99 mg, 80%) as a yellow oil. ^{1}H NMR (400 MHz, $CDCl_3$) δ = 8.63 (d, J = 4.8 Hz, 1H), 8.55 (d, J = 4.8 Hz, 0.68 H), 7.93 – 7.73 (m, 2.47 H), 7.67 (d, J = 7.8 Hz, 1H), 7.42 – 7.30 (m, 1.77 H), 5.32 (dd, J = 9.1, 5.8 Hz, 0.71 H), 5.12 (t, J = 7.4 Hz, 1H), 3.79 (d, J = 1.6 Hz, 5H), 3.19 (s, 3H), 3.11 (s, 2H), 2.97 (dd, J = 7.4, 2.6 Hz, 2H), 2.90 – 2.73 (m, 1.52 H), 2.07 (t, J = 2.6 Hz, 1H), 2.01 (t, J = 2.6 Hz, 0.64 H). ^{13}C NMR (101 MHz, $CDCl_3$) δ = 170.3, 170, 169.3, 153.9, 153.6, 148.5, 148.1, 137.2, 137.0, 125.0, 124.9, 124.7, 124.0, 79.9, 79.1, 71.7, 70.9, 59.9, 57.6, 52.8, 52.7, 35.8, 30.3, 20.4, 19.1. HR-MS (ESI) m/z calcd for $C_{13}H_{15}N_2O_3$ [M+H$^+$]: 247.1077, found: 247.1072.

Preparation of Dioxazolones

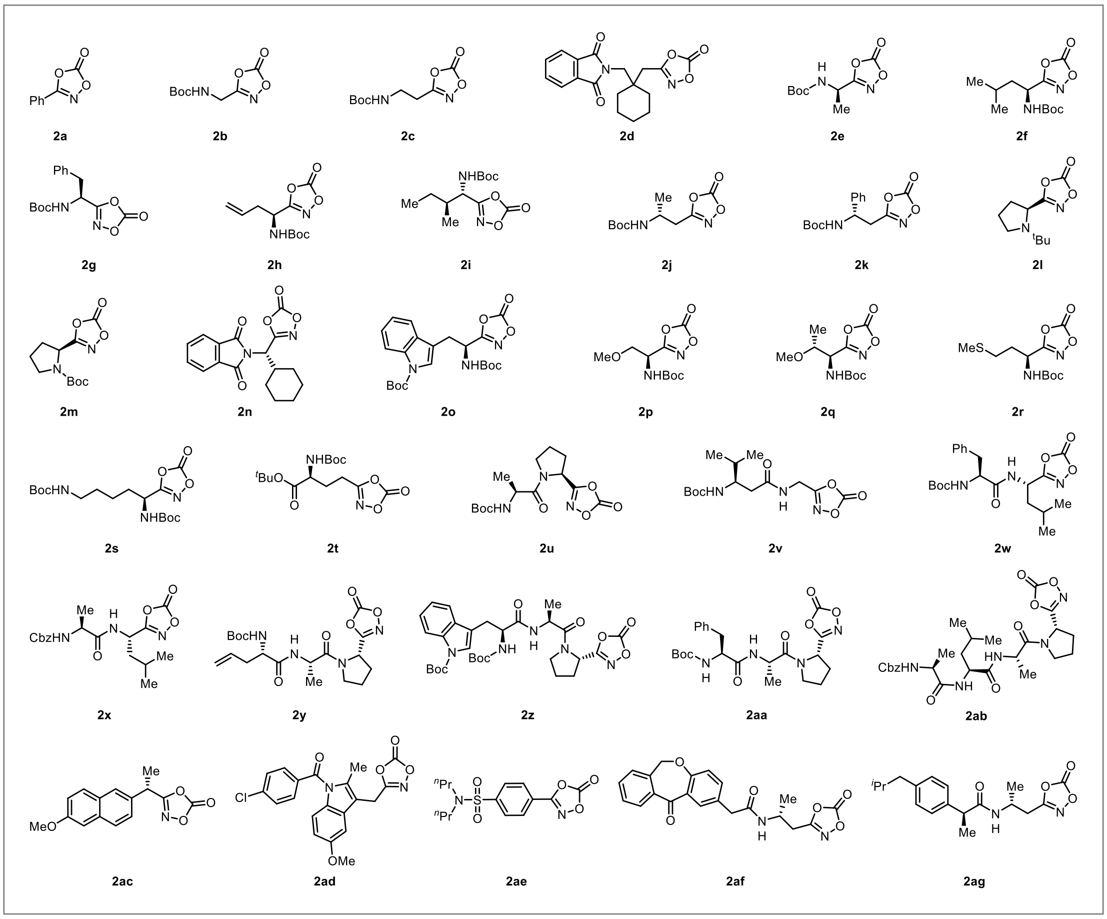

Figure S16. List of dioxazolones.

Dioxazolones substrates were prepared according to literatures.[1, 6-10]

Typical procedure 7 (TP7) for the preparation of dioxazolones substrates 2a-2d, 2j-2l, 2n, 2ac-2ae.

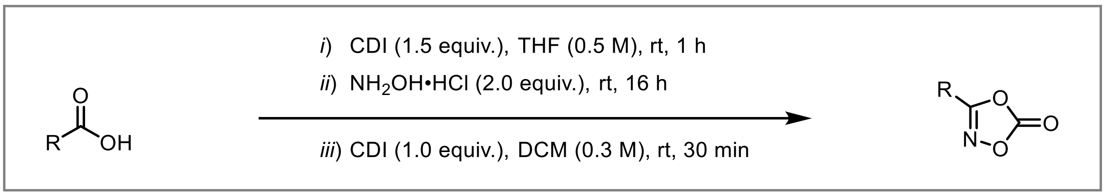

Figure S17. Typical procedure 7 (**TP7**) for the preparation of dioxazolones substrates **2a-2d, 2j-2l, 2n, 2ac-2ae**.

1,1'-Carbonyldiimidazole (CDI, 15 mmol, 1.5 equiv.) was added to a solution of carboxylic acid (10 mmol, 1.0 equiv.) in dry THF (30 mL). The reaction mixture was stirred at room temperature for 1 h. Powdered hydroxylamine hydrochloride (20 mmol, 2.0 equiv.) was added. The resulting mixture was stirred overnight. The mixture was diluted with 5% aq. $KHSO_4$ (20 mL) and extracted with EtOAc (30 mL × 2). The combined organic phase was washed with brine (50 mL) and dried over Na_2SO_4. The extract was filtered and concentrated under reduced pressure and the crude mixture was used directly in the next reaction without further purification. The crude mixture was dissolved in DCM (30 mL) and the CDI (10 mmol, 1.0 equiv.) was added to the solution of mixture at room temperature. After stirring for 30 minutes, the reaction mixture was quenched with 1 M HCl (20 mL), extracted with DCM (50 mL × 3), and dried over anhydrous Na_2SO_4. The extract was filtered and concentrated under reduced pressure and the crude mixture was purified by silica gel column chromatography to obtain the dioxazolones.

Typical procedure 8 (TP8) for the preparation of dioxazolones substrates 2e-2i, 2m, 2o-2ab, 2af-2ag.

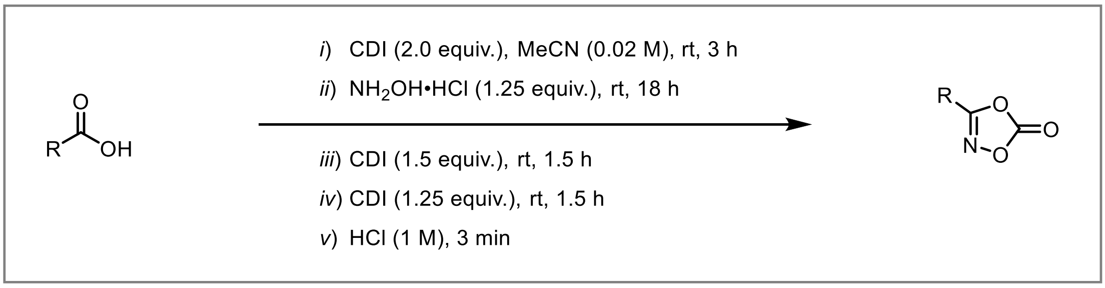

Figure S18. Typical procedure 8 (**TP8**) for the preparation of dioxazolones substrates **2e-2i, 2m, 2o-2ab** and **2af-2ag**.

To a flame-dried Schlenk tube equipped with stir bar was added carboxylic acid (1.0 equiv.),[1] followed by addition of dry MeCN (0.02 M), under an atmosphere of Ar. To the reaction mixture was added CDI (2.0 equiv.) in one portion and the mixture was stirred at room temperature for 3 h. Hydroxylamine hydrochloride (1.25 equiv.) was added in one portion to the reaction mixture and stirred 18 h. A second portion of CDI

(1.5 equiv.) was added to the reaction mixture and stirred for 1.5 h. A third portion of CDI (1.25 equiv.) was added to the reaction mixture and stirred for 1.5 h. The reaction was quenched with 1 M HCl solution, and allowed to stir at room temperature for 3 min. The resulting mixture was partitioned into a separatory funnel with EtOAc and water. The aqueous layer was extracted with EtOAc (× 3) and the combined organic layers were washed with brine (× 2), then dried over anhydrous Na_2SO_4. The extract was then filtered and concentrated under reduced pressure and the crude mixture was purified by silica gel column chromatography to obtain the dioxazolones.

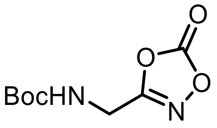

***tert*-Butyl [(5-oxo-1,4,2-dioxazol-3-yl)methyl]carbamate (2b)**

The general procedure **TP8** was followed using the corresponding carboxylic acid (10 mmol, 1.0 equiv.). Purification by column chromatography (petroleum ether/EtOAc = 5:1) yielded **2b** as a white solid. ^{1}H NMR (400 MHz, $CDCl_3$) δ = 5.12 (s, 1H), 4.33 (d, J = 4.6 Hz, 2H), 1.45 (s, 9H). ^{13}C NMR (101 MHz, $CDCl_3$) δ = 164.0, 155.2, 153.7, 81.5, 35.3, 28.2. HR-MS (ESI) m/z calcd for $C_8H_{12}N_2NaO_5$ [M+Na$^+$]: 239.0638, found: 239.0635.

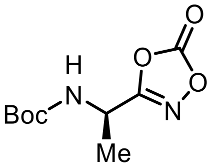

***tert*-Butyl (*R*)-[1-(5-oxo-1,4,2-dioxazol-3-yl)ethyl]carbamate (2e)**

The general procedure **TP8** was followed using the corresponding carboxylic acid (10 mmol, 1.0 equiv.). Purification by column chromatography (petroleum ether/EtOAc = 8:1) yielded **2e** as a white solid. ^{1}H NMR (400 MHz, $CDCl_3$) δ = 4.99 (s, 1H), 4.84 (s, 1H), 1.53 (d, J = 7.1 Hz, 3H), 1.44 (s, 9H). ^{13}C NMR (101 MHz, $CDCl_3$) δ = 166.9, 154.5, 153.8, 81.3, 42.7, 28.3, 17.7. HR-MS (ESI) m/z calcd for $C_9H_{14}N_2NaO_5$ [M+Na$^+$]: 253.0795, found: 253.0797.

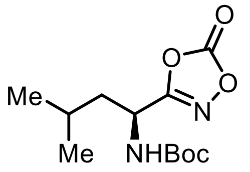

***tert*-Butyl (*S*)-[3-methyl-1-(5-oxo-1,4,2-dioxazol-3-yl)butyl]carbamate (2f)**

The general procedure **TP8** was followed using the corresponding carboxylic acid (10 mmol, 1.0 equiv.). Purification by column chromatography (petroleum ether/EtOAc = 8:1) yielded **2f** as a white solid. ^{1}H NMR (400 MHz, $CDCl_3$) δ = 4.97 (s, 1H), 4.78 (d, J = 4.9 Hz, 1H), 1.68 (dt, J = 13.0, 6.1 Hz, 3H), 1.42 (s, 9H), 0.95 (t, J = 6.5 Hz, 6H). ^{13}C NMR (101 MHz, $CDCl_3$) δ = 166.6, 154.8, 153.8, 81.1, 45.3, 40.4, 28.2, 24.5, 22.5, 21.6. HR-MS (ESI) m/z calcd for $C_{12}H_{20}N_2NaO_5$ [M+Na$^+$]: 295.1264, found: 295.1261.

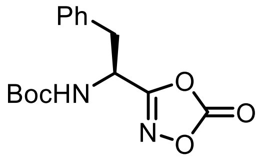

***tert*-Butyl (*S*)-[1-(5-oxo-1,4,2-dioxazol-3-yl)-2-phenylethyl]carbamate (2g)**

The general procedure **TP8** was followed using the corresponding carboxylic acid (20 mmol, 1.0 equiv.). Purification by column chromatography (petroleum ether/EtOAc = 5:1) yielded **2g** as a white solid. ^{1}H NMR (400 MHz, $CDCl_3$) δ = 7.33 (dd, J = 11.9, 7.0 Hz, 3H), 7.17 (d, J = 6.7 Hz, 2H), 5.04 (s, 1H), 4.81 (s, 1H), 3.24 – 3.07 (m, 2H), 1.41 (s, 9H). HR-MS (ESI) m/z calcd for $C_{15}H_{18}N_2NaO_5$ [M+Na$^+$]: 329.1108, found: 329.1108.

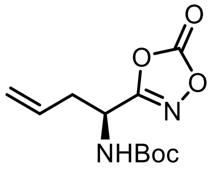

***tert*-Butyl (*S*)-[1-(5-oxo-1,4,2-dioxazol-3-yl)but-3-en-1-yl]carbamate (2h)**

The general procedure **TP8** was followed using the corresponding carboxylic acid (20 mmol, 1.0 equiv.). Purification by column chromatography (petroleum ether/EtOAc = 10:1) yielded **2h** as a white solid. ^{1}H NMR (400 MHz, $CDCl_3$) δ = 5.88 – 5.61 (m, 1H), 5.32 – 5.18 (m, 2H), 5.16 – 4.74 (m, 2H), 2.72 – 2.27 (m, 2H), 1.44 (s, 9H). ^{13}C NMR

(101 MHz, $CDCl_3$) δ = 165.9, 154.6, 153.7, 130.4, 121.2, 81.4, 46.4, 35.9, 28.3. HR-MS (ESI) m/z calcd for $C_{11}H_{16}N_2NaO_5$ [M+Na$^+$]: 279.0951, found: 279.0952.

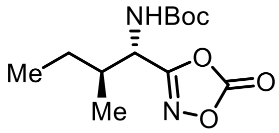

***tert*-Butyl [(1*S*,2*S*)-2-methyl-1-(5-oxo-1,4,2-dioxazol-3-yl)butyl]carbamate (2i)**

The general procedure **TP8** was followed using the corresponding carboxylic acid (10 mmol, 1.0 equiv.). Purification by column chromatography (petroleum ether/EtOAc = 20:1) yielded **2i** as a white solid. ^{1}H NMR (400 MHz, $CDCl_3$) δ = 4.89 (s, 1H), 4.69 (s, 1H), 1.95 – 1.79 (m, 1H), 1.53 (dd, J = 18.7, 5.2 Hz, 1H), 1.45 (s, 9H), 1.29 – 1.18 (m, 1H), 1.05 – 0.89 (m, 6H). ^{13}C NMR (101 MHz, $CDCl_3$) δ = 165.8, 154.9, 153.8, 81.2, 51.5, 37.1, 28.3, 24.9, 15.4, 11.2. HR-MS (ESI) m/z calcd for $C_{12}H_{20}N_2NaO_5$ [M+Na$^+$]: 295.1264, found: 295.1268.

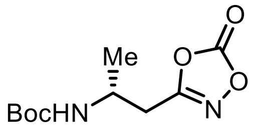

***tert*-Butyl (*R*)-[1-(5-oxo-1,4,2-dioxazol-3-yl)propan-2-yl]carbamate (2j)**

The general procedure **TP7** was followed using the corresponding carboxylic acid (10 mmol, 1.0 equiv.). Purification by column chromatography (petroleum ether/EtOAc = 5:1) yielded **2j** as a white solid. ^{1}H NMR (400 MHz, $CDCl_3$) δ = 4.71 (d, J = 5.1 Hz, 1H), 4.17 – 3.93 (m, 1H), 2.80 (dd, J = 15.0, 4.7 Hz, 1H), 2.69 (dd, J = 15.0, 7.5 Hz, 1H), 1.39 (s, 9H), 1.27 (d, J = 6.9 Hz, 3H). ^{13}C NMR (101 MHz, $CDCl_3$) δ = 164.5, 155.1, 154.1, 80.0, 43.7, 32.5, 28.2, 20.2. HR-MS (ESI) m/z calcd for $C_{10}H_{16}N_2NaO_5$ [M+Na$^+$]: 267.0951, found: 267.0950.

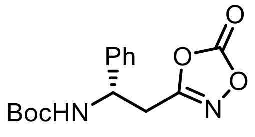

***tert*-Butyl (*S*)-[2-(5-oxo-1,4,2-dioxazol-3-yl)-1-phenylethyl]carbamate (2k)**

The general procedure **TP7** was followed using the corresponding carboxylic acid (4 mmol, 1.0 equiv.). Purification by column chromatography (petroleum ether/EtOAc =

7:1) yielded **2k** as a white solid. ^{1}H NMR (400 MHz, $CDCl_3$) δ = 7.39 – 7.12 (m, 5H), 5.00 (s, 2H), 3.09 (dd, J = 14.6, 7.9 Hz, 1H), 2.98 (dd, J = 14.8, 3.8 Hz, 1H), 1.34 (s, 9H). ^{13}C NMR (101 MHz, $CDCl_3$) δ = 164.2, 155.1, 154.0, 138.8, 129.3, 128.8, 126.3, 80.6, 51.8, 32.5, 28.3. HR-MS (ESI) m/z calcd for $C_{15}H_{18}N_2NaO_5$ [M+Na$^+$]: 329.1108, found: 329.1106.

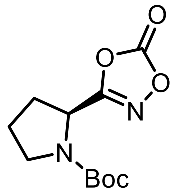

***tert*-Butyl (*S*)-2-(5-oxo-1,4,2-dioxazol-3-yl)pyrrolidine-1-carboxylate (2m)**

The general procedure **TP7** was followed using the corresponding carboxylic acid (10 mmol, 1.0 equiv.). Purification by column chromatography (petroleum ether/EtOAc = 8:1) yielded **2m** as a colorless oil. ^{1}H NMR (400 MHz, $CDCl_3$) δ = 4.70 (dd, J = 33.6, 5.1 Hz, 1H), 3.55 – 3.27 (m, 2H), 2.37 – 1.87 (m, 4H), 1.37 (d, J = 13.3 Hz, 9H). ^{13}C NMR (101 MHz, $CDCl_3$) δ = 166.7, 166.5, 153.9, 153.8, 153.7, 152.9, 81.0, 80.7, 52.0, 46.4, 46.1, 30.4, 29.6, 28.1, 24.0, 23.3. HR-MS (ESI) m/z calcd for $C_{11}H_{16}N_2NaO_5$ [M+Na$^+$]: 279.0951, found: 279.0954.

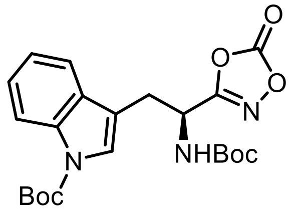

***tert*-Butyl (*S*)-3-{2-[(*tert*-butoxycarbonyl)amino]-2-(5-oxo-1,4,2-dioxazol-3-yl)ethyl}-1*H*-indole-1-carboxylate (2o)**

The general procedure **TP8** was followed using the corresponding carboxylic acid (10 mmol, 1.0 equiv.). Purification by column chromatography (petroleum ether/EtOAc = 10:1) yielded **2o** as a white solid. ^{1}H NMR (400 MHz, $CDCl_3$) δ = 8.10 (s, 1H), 7.46 (dd, J = 17.4, 12.0 Hz, 2H), 7.33 – 7.20 (m, 2H), 5.11 (d, J = 15.6 Hz, 2H), 3.24 (s, 2H), 1.63 (s, 9H), 1.37 (s, 9H). ^{13}C NMR (101 MHz, $CDCl_3$) δ = 165.8, 154.6, 153.6, 149.4, 135.4, 129.8, 124.9, 124.6, 123.0, 118.2, 115.5, 113.3, 84.1, 81.2, 60.4, 28.1, 27.3. HR-MS (ESI) m/z calcd for $C_{22}H_{27}N_3NaO_7$ [M+Na$^+$]: 468.1741, found: 468.1745.

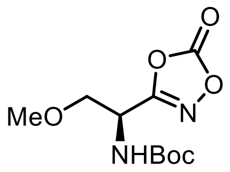

***tert*-Butyl (*S*)-[2-methoxy-1-(5-oxo-1,4,2-dioxazol-3-yl)ethyl]carbamate (2p)**

The general procedure **TP8** was followed using the corresponding carboxylic acid (10 mmol, 1.0 equiv.). Purification by column chromatography (petroleum ether/EtOAc = 8:1) yielded **2p** as a colorless oil. ^{1}H NMR (400 MHz, $CDCl_3$) δ = 5.49 (t, J = 7.5 Hz, 1H), 4.91 (d, J = 3.2 Hz, 1H), 3.72 (d, J = 9.5 Hz, 1H), 3.62 (dd, J = 9.8, 4.1 Hz, 1H), 3.34 (d, J = 1.5 Hz, 3H), 1.40 (d, J = 1.5 Hz, 9H). ^{13}C NMR (101 MHz, $CDCl_3$) δ = 165.0, 154.8, 153.8, 81.1, 70.5, 59.3, 47.3, 28.1. HR-MS (ESI) m/z calcd for $C_{10}H_{16}N_2NaO_6$ [M+Na$^+$]: 283.0901, found: 283.0903.

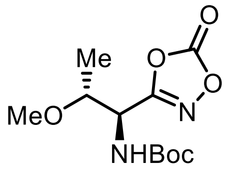

***tert*-Butyl [(1*S*,2*R*)-2-methoxy-1-(5-oxo-1,4,2-dioxazol-3-yl)propyl]carbamate (2q)**

The general procedure **TP8** was followed using the corresponding carboxylic acid (10 mmol, 1.0 equiv.). Purification by column chromatography (petroleum ether/EtOAc = 10:1) yielded **2q** as a colorless oil. ^{1}H NMR (400 MHz, $CDCl_3$) δ = 5.21 (d, J = 8.9 Hz, 1H), 4.76 (d, J = 9.3 Hz, 1H), 3.80 (dd, J = 6.0, 1.8 Hz, 1H), 3.30 (s, 3H), 1.44 (s, 9H), 1.26 (d, J = 6.2 Hz, 3H). ^{13}C NMR (101 MHz, $CDCl_3$) δ = 165.6, 155.4, 153.9, 81.1, 74.8, 56.8, 51.9, 28.2, 14.9. HR-MS (ESI) m/z calcd for $C_{11}H_{18}N_2NaO_6$ [M+Na$^+$]: 297.1057, found: 297.1055.

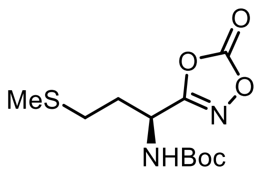

***tert*-Butyl (*S*)-[3-(methylthio)-1-(5-oxo-1,4,2-dioxazol-3-yl)propyl]carbamate (2r)**

The general procedure **TP8** was followed using the corresponding carboxylic acid (10 mmol, 1.0 equiv.). Purification by column chromatography (petroleum ether/EtOAc =

8:1) yielded **2r** as a colorless oil. ^{1}H NMR (400 MHz, $CDCl_3$) δ = 5.00 (d, J = 45.6 Hz, 2H), 2.71 – 2.49 (m, 2H), 2.28 – 1.97 (m, 5H), 1.45 (s, 9H). ^{13}C NMR (101 MHz, $CDCl_3$) δ = 166.0, 154.7, 153.7, 81.4, 46.0, 30.7, 29.6, 28.3, 15.5. HR-MS (ESI) m/z calcd for $C_{11}H_{18}N_2NaO_5S$ [M+Na$^+$]: 313.0829, found: 313.0831.

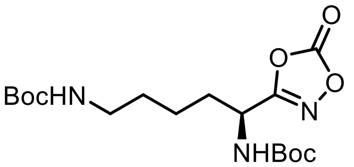

Di-*tert*-butyl [1-(5-oxo-1,4,2-dioxazol-3-yl)pentane-1,5-diyl](*S*)-dicarbamate (2s)

The general procedure **TP8** was followed using the corresponding carboxylic acid (10 mmol, 1.0 equiv.). Purification by column chromatography (petroleum ether/EtOAc = 5:1) yielded **2s** as a white solid. ^{1}H NMR (400 MHz, $CDCl_3$) δ = 5.18 (s, 1H), 4.71 (s, 1H), 4.62 (s, 1H), 3.20 – 3.07 (m, 2H), 1.86 (s, 2H), 1.61 – 1.48 (m, 5H), 1.44 (s, 18H). ^{13}C NMR (101 MHz, $CDCl_3$) δ = 166.4, 156.5, 153.8, 79.5, 53.7, 47.0, 39.4, 30.9, 29.7, 28.5, 28.3, 22.0. HR-MS (ESI) m/z calcd for $C_{17}H_{29}N_3NaO_7$ [M+Na$^+$]: 410.1898, found: 410.1899.

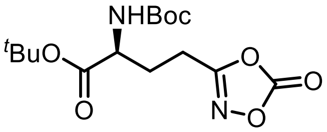

***tert*-Butyl (*S*)-2-[(*tert*-butoxycarbonyl)amino]-4-(5-oxo-1,4,2-dioxazol-3-yl)butanoate (2t)**

The general procedure **TP8** was followed using the corresponding carboxylic acid (20 mmol, 1.0 equiv.). Purification by column chromatography (petroleum ether/EtOAc = 7:1) yielded **2t** as a white solid. ^{1}H NMR (400 MHz, $CDCl_3$) δ = 5.16 (s, 1H), 4.27 (s, 1H), 2.86 – 2.61 (m, 2H), 2.38 – 2.22 (m, 1H), 2.06 – 1.89 (m, 1H), 1.48 (s, 9H), 1.44 (s, 9H). ^{13}C NMR (101 MHz, $CDCl_3$) δ = 170.4, 166.0, 155.5, 154.0, 83.2, 80.4, 53.0, 28.3, 28.0, 21.4. HR-MS (ESI) m/z calcd for $C_{15}H_{24}N_2NaO_7$ [M+Na$^+$]: 367.1476, found: 367.1479.

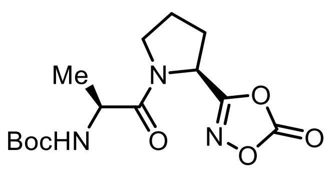

***tert*-Butyl {(*S*)-1-oxo-1-[(*S*)-2-(5-oxo-1,4,2-dioxazol-3-yl)pyrrolidin-1-yl]propan-2-yl}carbamate (2u)**

The general procedure **TP8** was followed using the corresponding carboxylic acid (20 mmol, 1.0 equiv.). Purification by column chromatography (petroleum ether/EtOAc = 3:1) yielded **2u** as a white solid. ^{1}H NMR (400 MHz, CDCl$_3$) δ = 5.27 (d, J = 7.9 Hz, 1H), 5.06 (dd, J = 8.0, 2.9 Hz, 1H), 4.49 – 4.38 (m, 1H), 3.82 – 3.71 (m, 1H), 3.67 – 3.61 (m, 1H), 2.35 – 2.26 (m, 1H), 2.15 (dd, J = 11.5, 5.9 Hz, 3H), 1.41 (s, 9H), 1.32 (d, J = 7.0 Hz, 3H). ^{13}C NMR (101 MHz, CDCl$_3$) δ = 172.8, 165.8, 155.3, 153.7, 80.0, 52.0, 47.9, 46.7, 28.9, 28.4, 24.7, 18.3. HR-MS (ESI) m/z calcd for $C_{14}H_{21}N_3NaO_6$ [M+Na$^+$]: 350.1323, found: 350.1320.

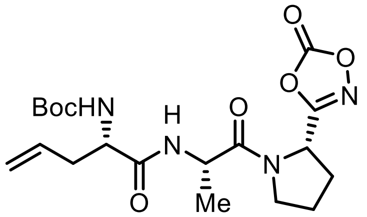

***tert*-Butyl {{{(*S*)-1-oxo-1-{{{(*S*)-1-oxo-1-[(*S*)-2-(5-oxo-1,4,2-dioxazol-3-yl)pyrrolidin-1-yl]propan-2-yl}amino}}pent-4-en-2-yl}}}carbamate (2y)**

The general procedure **TP8** was followed using the corresponding carboxylic acid (10 mmol, 1.0 equiv.). Purification by column chromatography (petroleum ether/EtOAc = 1:1) yielded **2y** as a white solid. ^{1}H NMR (400 MHz, CDCl$_3$) δ = 7.23 (s, 1H), 5.75 – 5.61 (m, 1H), 5.21 – 5.00 (m, 4H), 4.70 (p, J = 6.9 Hz, 1H), 4.19 (d, J = 4.4 Hz, 1H), 3.77 (dd, J = 15.6, 7.3 Hz, 1H), 3.70 – 3.59 (m, 1H), 2.52 – 2.40 (m, 2H), 2.37 – 2.26 (m, 1H), 2.22 – 2.06 (m, 3H), 1.41 (s, 9H), 1.34 (d, J = 6.9 Hz, 3H). ^{13}C NMR (101 MHz, CDCl$_3$) δ = 172.0, 165.6, 155.6, 153.7, 132.8, 119.2, 80.3, 53.7, 52.0, 46.9, 46.8, 36.9, 28.8, 28.3, 24.6, 17.9. HR-MS (ESI) m/z calcd for $C_{19}H_{28}N_4NaO_7$ [M+Na$^+$]: 447.1850, found: 447.1847.

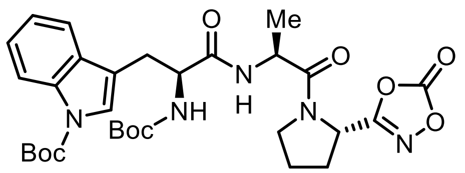

tert-Butyl 3-{{{(_S_)-2-[(_tert_-butoxycarbonyl)amino]-3-oxo-3-{{{(_S_)-1-oxo-1-[(S)-2-(5-oxo-1,4,2-dioxazol-3-yl)pyrrolidin-1-yl]propan-2-yl}amino}}propyl}}}-1_H_-indole-1-carboxylate (2z)

The general procedure **TP8** was followed using the corresponding carboxylic acid (10 mmol, 1.0 equiv.). Purification by column chromatography (petroleum ether/EtOAc = 3:2) yielded **2z** as a white solid. ^{1}H NMR (400 MHz, $CDCl_3$) δ = 8.09 (d, J = 6.9 Hz, 1H), 7.52 (d, J = 7.7 Hz, 1H), 7.46 (s, 1H), 7.25 (s, 1H), 7.19 (t, J = 7.4 Hz, 1H), 6.85 (s, 1H), 5.31 (d, J = 7.7 Hz, 1H), 4.97 (dd, J = 8.0, 2.6 Hz, 1H), 4.44 (s, 2H), 3.52 (s, 2H), 3.27 – 3.15 (m, 1H), 3.10 (dd, J = 13.7, 7.0 Hz, 1H), 2.35 – 2.23 (m, 1H), 2.15 – 2.06 (m, 3H), 1.66 (d, J = 5.2 Hz, 9H), 1.42 (s, 9H), 1.23 (d, J = 6.8 Hz, 3H). ^{13}C NMR (101 MHz, $CDCl_3$) δ = 171.4, 170.7, 165.6, 155.3, 153.7, 149.6, 135.4, 130.5, 124.4, 124.3, 122.4, 119.1, 115.4, 115.2, 83.8, 80.3, 54.8, 51.8, 46.8, 46.5, 28.8, 28.3, 28.2, 24.6, 18.0. HR-MS (ESI) m/z calcd for $C_{30}H_{39}N_5NaO_9$ [M+Na$^+$]: 636.2640, found: 636.2643.

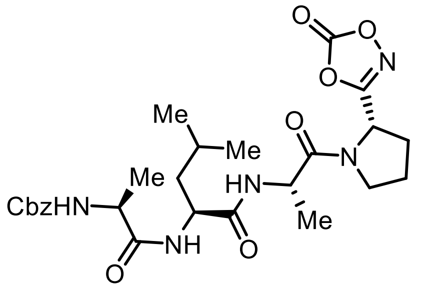

Benzyl {{{{(_S_)-1-{{{{{{(_S_)-4-methyl-1-oxo-1-{{{(_S_)-1-oxo-1-[(_S_)-2-(5-oxo-1,4,2-dioxazol-3-yl)pyrrolidin-1-yl]propan-2-yl}amino}}pentan-2-yl}}}amino}}}}-1-oxopropan-2-yl}}}}}carbamate (2aa)

The general procedure **TP8** was followed using the corresponding carboxylic acid (3 mmol, 1.0 equiv.). Purification by column chromatography (petroleum ether/EtOAc = 1:3) yielded **2aa** as a white solid. ^{1}H NMR (400 MHz, $CDCl_3$) δ = 7.44 (d, J = 7.5 Hz,

1H), 7.38 – 7.27 (m, 5H), 6.93 (d, J = 8.0 Hz, 1H), 5.74 (d, J = 7.6 Hz, 1H), 5.21 – 4.97 (m, 3H), 4.81 – 4.66 (m, 1H), 4.59 (dd, J = 13.5, 8.4 Hz, 1H), 4.43 – 4.19 (m, 1H), 3.77 (dd, J = 16.4, 7.4 Hz, 1H), 3.71 – 3.53 (m, 1H), 2.26 (dt, J = 19.4, 7.3 Hz, 1H), 2.13 (dd, J = 10.2, 5.3 Hz, 3H), 1.65 – 1.46 (m, 3H), 1.34 (t, J = 6.4 Hz, 6H), 0.87 (t, J = 6.4 Hz, 6H). ^{13}C NMR (101 MHz, $CDCl_3$) δ = 172.6, 172.0, 165.6, 156.0, 153.8, 136.4, 128.5, 128.1, 128.0, 66.8, 52.0, 51.5, 50.3, 46.7, 46.6, 41.7, 28.6, 24.7, 24.6, 22.8, 22.2, 19.0, 17.7. HR-MS (ESI) m/z calcd for $C_{26}H_{35}N_5NaO_8$ [M+Na$^+$]: 568.2378, found: 568.2375.

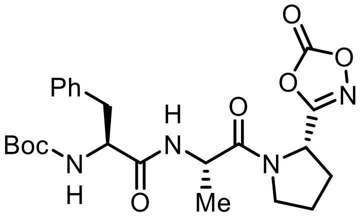

***tert*-Butyl {{{(*S*)-1-oxo-1-{{{(*S*)-1-oxo-1-[(*S*)-2-(5-oxo-1,4,2-dioxazol-3-yl)pyrrolidin-1-yl]propan-2-yl}amino}}-3-phenylpropan-2-yl}}}carbamate (2ab)**

The general procedure **TP8** was followed using the corresponding carboxylic acid (10 mmol, 1.0 equiv.). Purification by column chromatography (petroleum ether/EtOAc = 3:1) yielded **2ab** as a white solid. ^{1}H NMR (400 MHz, $CDCl_3$) δ = 7.23 (dd, J = 19.2, 11.6 Hz, 6H), 5.12 (s, 1H), 5.04 (d, J = 5.9 Hz, 1H), 4.75 – 4.62 (m, 1H), 4.45 (s, 1H), 3.68 (d, J = 29.3 Hz, 2H), 3.19 – 2.91 (m, 2H), 2.29 (s, 1H), 2.15 (s, 3H), 1.40 – 1.29 (m, 12H). ^{13}C NMR (101 MHz, $CDCl_3$) δ = 171.8, 171.1, 165.6, 155.4, 153.7, 136.5, 129.3, 128.5, 126.9, 80.2, 55.3, 51.9, 46.8, 46.7, 38.2, 28.8, 28.2, 24.6, 17.8. HR-MS (ESI) m/z calcd for $C_{23}H_{30}N_4NaO_7$ [M+Na$^+$]: 497.2007, found: 497.2004.

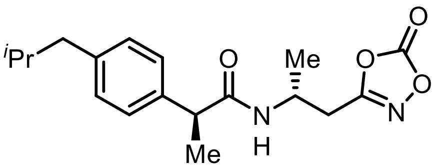

(*S*)-2-(4-Isobutylphenyl)-*N*-[(*R*)-1-(5-oxo-1,4,2-dioxazol-3-yl)propan-2-yl]propanamide (2ag)

The general procedure **TP8** was followed using the corresponding carboxylic acid (4 mmol, 1.0 equiv.). Purification by column chromatography (petroleum ether/EtOAc =

3:1) yielded **2ag** as a white solid. ^{1}H NMR (400 MHz, $CDCl_3$) δ = 7.14 (dd, J = 23.3, 7.5 Hz, 4H), 5.68 (d, J = 6.9 Hz, 1H), 4.38 – 4.22 (m, 1H), 3.50 (d, J = 7.0 Hz, 1H), 2.86 (dd, J = 15.1, 4.7 Hz, 1H), 2.71 (dd, J = 15.1, 6.9 Hz, 1H), 2.45 (d, J = 6.9 Hz, 2H), 1.85 (dt, J = 13.1, 6.5 Hz, 1H), 1.47 (d, J = 7.0 Hz, 3H), 1.19 (d, J = 6.7 Hz, 3H), 0.89 (d, J = 6.4 Hz, 6H). ^{13}C NMR (101 MHz, $CDCl_3$) δ = 174.7, 164.4, 154.0, 140.9, 138.0, 129.6, 127.2, 46.5, 45.0, 42.5, 31.5, 30.2, 22.4, 19.7, 18.4. HR-MS (ESI) m/z calcd for $C_{18}H_{24}N_2NaO_4$ [M+Na$^+$]: 355.1628, found: 355.1630.

Preparation and Stability Study of Silylzinc Reagents

a) Preparation of arylated silylzinc reagents 3a-3g, 3a(II)-3a(V)

Me_2PhSi—ZnOPiv	$MePh_2Si$—ZnOPiv	Ph_3Si—ZnOPiv	Me_3Si—ZnOPiv	Ph_2HSi—ZnOPiv
**3a**	**3b**	**3c**	**3d**	**3e**
Et(Me)PhSi—ZnOPiv	$Me_2(3\text{-}Me\text{-}C_6H_4)Si$—ZnOPiv	Me_2PhSi—ZnOAc	Me_2PhSi—ZnOAd	Me_2PhSi—ZnCl
**3f**	**3g**	**3a** (**II**)	**3a** (**III**)	**3a** (**IV**)
Me_2PhSi—ZnBr				
**3a** (**V**)				

Figure S19. List of silylzinc reagents.

Note: *These silylzinc pivalates exhibited excellent solubility in polar solvents, such as DMA, DMF, 1,4-dioxane, THF, Et_2O, and 2-Me-THF, as well as the nonpolar solvent of PhMe.*

Preparation of $Zn(OPiv)_2$

Pivalic acid (20.4 g, 22.6 mL, 200 mmol) was placed in a dry and argon-flushed 500 mL three-necked round bottom flask, equipped with a magnetic stirring bar, a septum and a pressure equalizer, and was dissolved in dry THF (120 mL). The mixture was cooled to 0 °C, and a solution of Et_2Zn (13.0 g, 10.8 mL, 105 mmol) in dry hexane (120 mL) was added over a period of 30 min under vigorous stirring. Then, the ice-bath was removed and stirring was continued at 25 °C for one additional hour at which point bubbling has ceased (a thick slurry was formed). The solvent was removed in vacuo and the solid residue was dried for at least 4 h longer. $Zn(OPiv)_2$ was obtained in quantitative yield, as a puffy amorphous white solid.

Preparation of $Zn(OAd)_2$

1-adamantaneacetic acid (1.94 g, 10 mmol) was placed in a dry and nitrogen flushed 100 mL three-necked roundbottom flask, equipped with a magnetic stirring bar, a septum and a pressure equalizer, and was dissolved in dry THF (5.0 mL). The mixture was cooled to 0 °C, and a solution of Et_2Zn (5.2 mL, 5.2 mmol, 1.0 M in heptane) was

added over a period of 30 minutes under vigorous stirring. Then, the ice-bath was removed and stirring was continued at 25 °C for one additional hour at which point bubbling has ceased (a thick slurry was formed). The solvent was removed in vacuo and the solid residue was dried for at least 4 hours longer. $Zn(OAd)_2$ was yielded (1.8 g, 80%) as a puffy amorphous white solid.

Typical procedure 9 (TP9) for the preparation of arylated silylzinc reagents

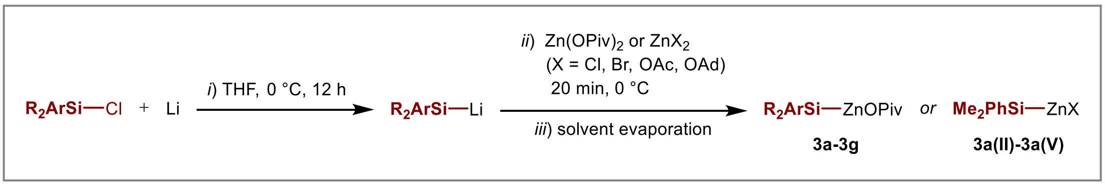

Figure S20. Typical procedure 9 (**TP9**) for the preparation of arylated silylzinc reagents.

To a flame-dried 25 mL schlenk tube equipped with a magnetic stirring bar, lithium clippings (208 mg, 30 mmol, 3.0 equiv.) was added. After evacuated and backfilled with argon three times. Dry THF (10 mL) was added via a syringe. Then chlorosilane (10 mmol, 1.0 equiv.) was added at 0 °C and the mixture was stirred at same temperature for 12 h. The silyllithium was titrated against diphenylacetic acid according to Kofron's method (routinely formed as a ~ 0.75 M solution).[11] Next, this solution was added via syringe into a flame-dried 25 mL schlenk tube equipped with a magnetic stirring and ZnX_2 (X = OPiv, Cl, Br, OAc, OAd) (1 M solution in THF, 1.2 equiv. with respect to titrated silyllithium) at 0 °C, and the reaction mixture was stirred at 0 °C for 20 min. The $R_2ArSi–ZnX$ (X = OPiv, Cl, Br, OAc, OAd) (routinely formed as a ~ 0.36 M solution) was titrated using Knochel's method.[12]

Photographic depiction reaction setup according to the Typical procedure 9

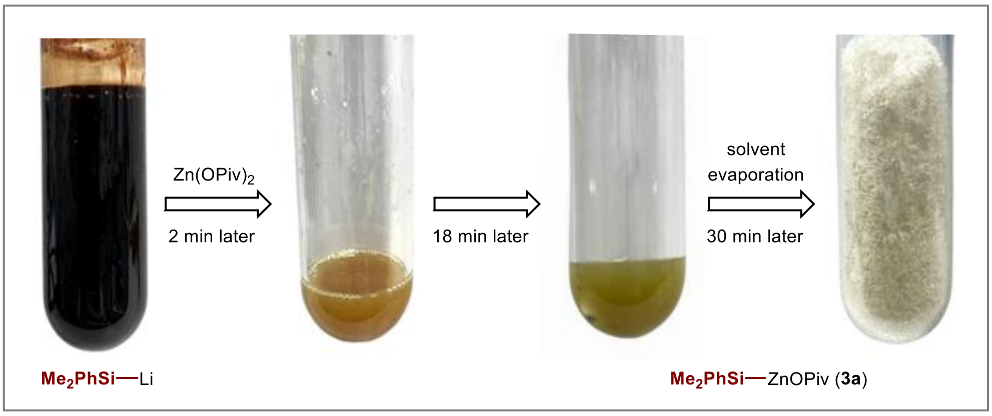

Figure S21. Synthetic scheme for solid $PhMe_2Si–ZnOPiv$.

Typical procedure 10 (TP10) for the preparation of trimethylsilylzinc reagents

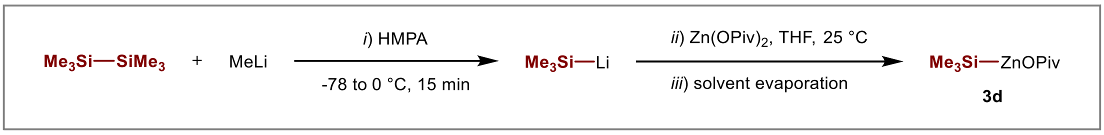

Figure S22. Typical procedure 10 (**TP10**) for the preparation of trimethylsilylzinc reagents.

A 50-mL two-necked round-bottomed flask equipped with a magnetic stirring bar and was charged with hexamethyldisilane (1.00 mL, 4.88 mmol) and HMPA (2.00 mL). The reaction mixture was cooled to -78 °C. To the white solid was added to MeLi (1.6 M in Et_2O, 2.5 mL, 4.0 mmol) and dry THF (8.0 mL) at -78 °C, which was warmed to 0 °C. After stirring at 0 °C for 15 min, the trimethylsilyllithium was titrated against diphenylacetic acid according to Kofron's method (routinely formed as a ~ 0.29 M solution).[11, 13] Next, this solution was added via syringe into a 50 mL two-necked round-bottomed flask equipped with a magnetic stirring and $Zn(OPiv)_2$ (1.2 equiv. with respect to titrated silyllithium) at 0 °C, then the reaction mixture was stirred at 25 °C for 15 min to afford a solution of the trimethylsilylzinc reagent. The colorless silylzinc solution (routinely formed as a ~ 0.25 M solution) was titrated using Knochel's method.[12]

b) Stability study of solid silylzinc reagents

Titration of silylzinc reagents using iodine[12]

Accurately weighted aliquots (100 mg) of solid silylzinc pivalates were dissolved in dry THF, so that the total volume of the solution was 1.0 mL. The formed solution was added a standard solution of iodine (0.3 M, in dry THF) until the complete appearance of the dark brown color of iodine. Thus, the concentration of the active species (in mmol/g) was determined.

Stability studies of solid silylzinc pivalates under nitrogen

To evaluate the stability of silylzinc reagents under nitrogen, accurately weighed aliquots of the solid material were placed in Schlenk-flasks at 25 °C. After an extended period of time under nitrogen at the given temperature the solid material was dissolved in THF and the resulting solution was titrated against iodine, according to the procedure described above and the measured concentration was compared to the one before the storage. The stability of silylzinc pivalates is presented in **Figure S23**. Notably, the reactivity of these silylzincs only slightly reduces after 30 days when it is stored under nitrogen atmosphere at room temperature, the values of their half-life ($t_{1/2}$) are more than 117 days.

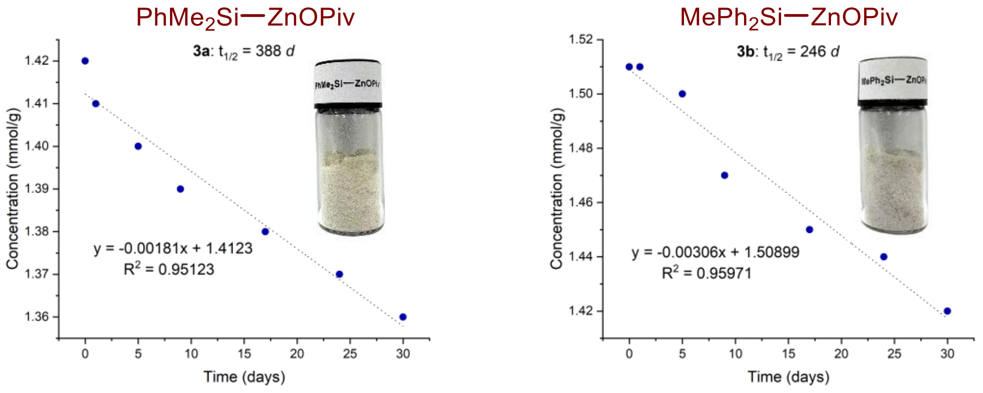

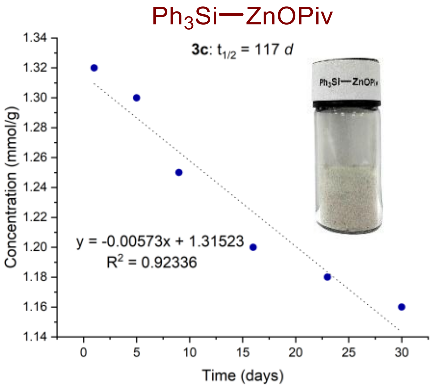

Figure S23. Stability of solid silylzinc pivalates (**3a**–**3c**) under N_2 atmosphere.

Stability studies of PhMe$_2$Si–ZnOPiv under air

To evaluate the stability of silylzinc reagents under air, accurately weighed aliquots of the solid **PhMe$_2$Si–ZnOPiv** (**3a**) were placed in Schlenk-flasks at 25 °C. After an extended period of time under air at the given temperature the solid material was dissolved in THF and the resulting solution was titrated against iodine, according to the procedure described above and the measured concentration was compared to the one before the storage. The stability of the **3a** is presented in **Figure S24**. The half-life of PhMe$_2$Si–ZnOPiv in air at room temperature is 89 hours.

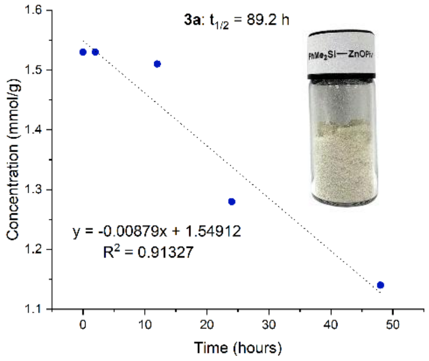

Figure S24. Stability of solid silylzinc pivalates (**3a**) under air.

General Procedure for Co-Catalyzed Silylamidation

Typical procedure 11 (TP11) for the Co-catalyzed silylamidation

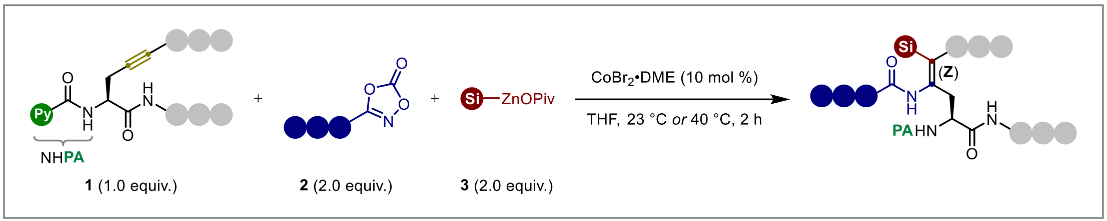

Figure S25. Typical procedure 11 (**TP11**) for the co-catalyzed silylamidation.

In a nitrogen-filled glovebox, alkyne **1** (1.0 equiv.), dioxazolone **2** (2.0 equiv.), $CoBr_2$•DME (10 mol %), and anhydrous THF (0.5 mL) were added to an oven-dried 10-mL scintillation vial equipped with a Teflon-coated magnetic stir bar. The vial was sealed with a screw-top septum cap and removed from the glovebox. A solution of silylzinc pivalate **3** (2.0 equiv.) in anhydrous THF (1.0 mL) or THF (0.5 mL) was then added dropwise via syringe to the reaction mixture at 23 °C or 40 °C under argon atmosphere with stirring. The reaction was stirred under argon atmosphere at 23 °C or 40 °C for 2 h. The mixture was then diluted with DCM (4 mL) and quenched with saturated aq. $NaHCO_3$ solution (3 mL). The resulting mixture was extracted with DCM (10 mL × 3). The combined organic layers were dried over Na_2SO_4, filtered, and concentrated under reduced pressure. The crude product was purified by column chromatography on silica gel to afford the desired silylamidation product.

Typical procedure 12 (TP12) for the Co-catalyzed silylamidation

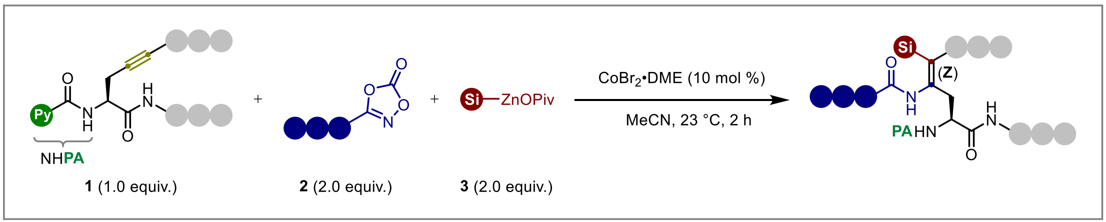

Figure S26. Typical procedure 12 (**TP12**) for the co-catalyzed silylamidation.

In a nitrogen-filled glovebox, alkyne **1** (1.0 equiv.), dioxazolone **2** (2.0 equiv.), $CoBr_2$•DME (10 mol %), and anhydrous MeCN (0.5 mL) were added to an oven-dried 10-mL scintillation vial equipped with a Teflon-coated magnetic stir bar. The vial was sealed with a screw-top septum cap and removed from the glovebox. A solution of silylzinc pivalate **3** (2.0 equiv.) in anhydrous MeCN (1.0 mL) or MeCN (0.5 mL) was then added dropwise via syringe to the reaction mixture at 23 °C under argon atmosphere with stirring. The reaction was stirred under argon atmosphere at 23 °C for 2 h. The mixture was then diluted with DCM (4 mL) and quenched with saturated aq. $NaHCO_3$ solution (3 mL). The resulting mixture was extracted with DCM (10 mL × 3). The combined organic layers were dried over Na_2SO_4, filtered, and concentrated under reduced pressure. The crude product was purified by column chromatography on silica gel to afford the desired silylamidation product.

Characterization Data of Products 4−64

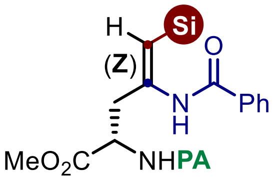

Methyl (*S*,*Z*)-4-benzamido-5-[dimethyl(phenyl)silyl]-2-(picolinamido)pent-4-enoate (4)

The general procedure **TP11** was followed using **1a** (23.2 mg, 0.1 mmol, 1.0 equiv.), **2a** (32.6 mg, 0.2 mmol, 2.0 equiv.), **3a** (0.5 mL, 0.2 mmol, 2.0 equiv.) and THF (0.5 mL) for 2 h. Purification by column chromatography (petroleum ether/EtOAc = 4:1) yielded **4** (35.0 mg, 72%) as a colorless oil. ^{1}H NMR (400 MHz, $CDCl_3$) δ = ^{1}H NMR (400 MHz, $CDCl_3$) δ 8.67 (d, *J* = 8.4 Hz, 1H), 8.54 (d, *J* = 4.3 Hz, 1H), 8.18 (d, *J* = 7.8 Hz, 1H), 7.86 (td, *J* = 7.7, 1.5 Hz, 1H), 7.48 – 7.38 (m, 5H), 7.34 (t, *J* = 7.4 Hz, 1H), 7.24 (dd, *J* = 12.2, 4.9 Hz, 4H), 7.18 (d, *J* = 7.3 Hz, 2H), 5.26 (s, 1H), 4.98 (dd, *J* = 8.8, 4.1 Hz, 1H), 3.69 (s, 3H), 3.59 (dd, *J* = 13.8, 4.8 Hz, 1H), 3.12 (dd, *J* = 13.8, 9.2 Hz, 1H), 0.26 (s, 3H), 0.25 (s, 3H). ^{13}C NMR (101 MHz, $CDCl_3$) δ = 172.2, 165.5, 164.2, 149.5, 148.3, 147.4, 137.4, 137.3, 134.1, 133.6, 131.7, 129.7, 128.6, 128.5, 127.1, 126.5, 122.4, 119.3, 52.6, 51.2, 39.9, -2.0, -2.1. HR-MS (ESI) m/z calcd for $C_{27}H_{29}N_3NaO_4Si$ [M+Na^{+}]: 510.1820, found: 510.1827.

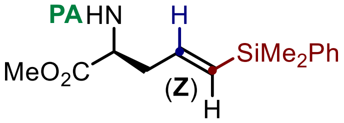

Methyl (*S*,*E*)-5-(dimethyl(phenyl)silyl)-2-(picolinamido)pent-4-enoate (4')

^{1}H NMR (400 MHz, $CDCl_3$) δ = 8.62 – 8.41 (m, 2H), 8.17 (d, *J* = 7.8 Hz, 1H), 7.84 (td, *J* = 7.7, 1.7 Hz, 1H), 7.52 – 7.40 (m, 3H), 7.33 – 7.25 (m, 3H), 6.16 – 5.86 (m, 2H), 4.88 (ddd, *J* = 8.2, 6.8, 5.8 Hz, 1H), 3.73 (s, 3H), 2.89 – 2.66 (m, 2H), 0.36 – 0.22 (m, 6H). ^{13}C NMR (101 MHz, $CDCl_3$) δ = 172.1, 164.1, 149.5, 148.4, 141.9, 138.5, 137.4, 133.9, 133.9, 129.0, 127.8, 126.5, 122.4, 52.5, 51.7, 39.8, -2.6. HR-MS (ESI) m/z calcd for $C_{20}H_{25}N_2O_3Si$ [M+H^{+}]: 369.1629, found: 369.1633.

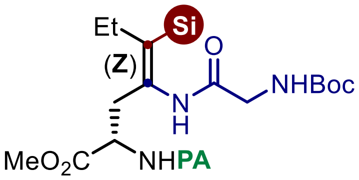

Methyl (*S*,*Z*)-4-{2-[(*tert*-butoxycarbonyl)amino]acetamido}-5-[dimethyl(phenyl)silyl]-2-(picolinamido)hept-4-enoate (5)

The general procedure **TP11** was followed using **1c** (26.0 mg, 0.1 mmol, 1.0 equiv.), **2b** (43.2 mg, 0.2 mmol, 2.0 equiv.), **3a** (0.5 mL, 0.2 mmol, 2.0 equiv.) and THF (0.5 mL) for 2 h. Purification by column chromatography (petroleum ether/EtOAc = 1:1) yielded **5** (37.0 mg, 65%) as a colorless oil. ^{1}H NMR (600 MHz, $CDCl_3$) δ = 8.59 (d, *J* = 4.7 Hz, 1H), 8.56 (d, *J* = 8.9 Hz, 1H), 8.13 (d, *J* = 7.9 Hz, 1H), 7.88 (td, *J* = 7.7, 1.6 Hz, 1H), 7.47 (ddd, *J* = 7.5, 4.8, 1.1 Hz, 1H), 7.39 (d, *J* = 6.7 Hz, 2H), 7.30 (t, *J* = 7.4 Hz, 1H), 7.22 (t, *J* = 7.4 Hz, 2H), 7.17 (s, 1H), 4.88 (td, *J* = 9.6, 4.7 Hz, 2H), 3.75 (s, 3H), 3.70 – 3.65 (m, 1H), 3.48 (s, 1H), 3.16 (dd, *J* = 14.4, 4.8 Hz, 1H), 2.99 (dd, *J* = 14.3, 10.2 Hz, 1H), 2.21 – 2.14 (m, 2H), 1.43 (s, 9H), 0.91 (t, *J* = 7.5 Hz, 3H), 0.30 (s, 3H), 0.27 (s, 3H). ^{13}C NMR (151 MHz, $CDCl_3$) δ = 172.3, 168.5, 164.0, 155.6, 149.2, 148.4, 139.4, 139.3, 138.7, 137.4, 133.4, 129.2, 128.2, 126.5, 122.4, 79.8, 52.6, 50.4, 44.0, 33.3, 28.3, 24.0, 14.8, -1.8, -1.9. HR-MS (ESI) m/z calcd for $C_{29}H_{40}N_4NaO_6Si$ [M+Na$^+$]: 591.2609, found: 591.2602.

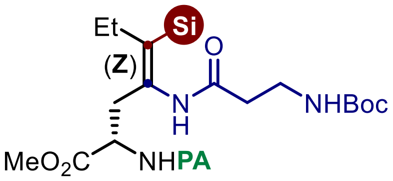

Methyl (*S*,*Z*)-4-{3-[(*tert*-butoxycarbonyl)amino]propanamido}-5-[dimethyl(phenyl)silyl]-2-(picolinamido)hept-4-enoate (6)

The general procedure **TP11** was followed using **1c** (26.0 mg, 0.1 mmol, 1.0 equiv.), **2c** (46.0 mg, 0.2 mmol, 2.0 equiv.), **3a** (0.5 mL, 0.2 mmol, 2.0 equiv.) and THF (0.5 mL) for 2 h. Purification by column chromatography (petroleum ether/EtOAc = 1:1) yielded **6** (38.0 mg, 65%) as a colorless oil. ^{1}H NMR (400 MHz, $CDCl_3$) δ = 8.57 (t, *J* = 7.7 Hz, 2H), 8.13 (d, *J* = 7.8 Hz, 1H), 7.88 (t, *J* = 7.7 Hz, 1H), 7.51 – 7.44 (m, 1H),

7.43 – 7.36 (m, 2H), 7.31 (dd, J = 10.6, 4.1 Hz, 1H), 7.26 (dd, J = 11.2, 4.0 Hz, 2H), 6.63 (s, 1H), 5.40 (s, 1H), 4.87 (td, J = 9.2, 5.5 Hz, 1H), 3.74 (s, 3H), 3.32 – 3.13 (m, 3H), 3.00 (dd, J = 14.0, 9.9 Hz, 1H), 2.19 (tq, J = 13.4, 6.6 Hz, 2H), 2.03 (dt, J = 19.1, 5.4 Hz, 1H), 1.93 (dt, J = 12.3, 5.6 Hz, 1H), 1.42 (s, 9H), 0.94 (t, J = 7.4 Hz, 3H), 0.29 (d, J = 8.2 Hz, 6H). ^{13}C NMR (101 MHz, $CDCl_3$) δ = 172.3, 170.9, 164.0, 156.0, 149.2, 148.4, 140.0, 138.6, 138.2, 137.4, 133.4, 129.4, 128.3, 126.6, 122.4, 79.0, 52.6, 50.6, 36.4, 35.9, 33.0, 28.5, 23.9, 15.0, -1.9, -1.9. HR-MS (ESI) m/z calcd for $C_{30}H_{42}N_4NaO_6Si$ [M+Na$^+$]: 605.2766, found: 605.2762.

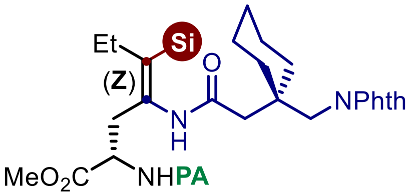

Methyl (*S*,*Z*)-5-[dimethyl(phenyl)silyl]-4-{{2-{1-[(1,3-dioxoisoindolin-2-yl)methyl]cyclohexyl}acetamido}}-2-(picolinamido)hept-4-enoate (7)

The general procedure **TP11** was followed using **1c** (26.0 mg, 0.1 mmol, 1.0 equiv.), **2d** (68.5 mg, 0.2 mmol, 2.0 equiv.), **3a** (0.5 mL, 0.2 mmol, 2.0 equiv.) and THF (0.5 mL) for 2 h. Purification by column chromatography (petroleum ether/EtOAc = 1.5:1) yielded **7** (45.0 mg, 65%) as a colorless oil. ^{1}H NMR (400 MHz, $CDCl_3$) δ = 8.64 (d, J = 8.8 Hz, 1H), 8.37 (d, J = 4.2 Hz, 1H), 8.12 (d, J = 7.8 Hz, 1H), 7.86 – 7.75 (m, 3H), 7.67 (dd, J = 5.4, 3.0 Hz, 2H), 7.40 (d, J = 7.2 Hz, 2H), 7.35 (dd, J = 6.8, 5.1 Hz, 1H), 7.20 – 7.07 (m, 3H), 6.98 (s, 1H), 5.02 (td, J = 10.2, 5.2 Hz, 1H), 3.88 – 3.67 (m, 5H), 3.23 (dd, J = 14.1, 5.1 Hz, 1H), 3.05 (dd, J = 13.9, 10.8 Hz, 1H), 2.24 (tt, J = 13.8, 6.8 Hz, 2H), 1.87 (q, J = 15.1 Hz, 2H), 1.74 – 1.63 (m, 2H), 1.61 – 1.55 (m, 2H), 1.53 – 1.46 (m, 2H), 1.35 – 1.31 (m, 2H), 1.30 – 1.26 (m, 2H), 0.99 (t, J = 6.9 Hz, 3H), 0.32 (s, 6H). ^{13}C NMR (101 MHz, $CDCl_3$) δ = 172.4, 170.3, 169.4, 164.1, 149.6, 148.2, 140.8, 139.1, 138.3, 137.2, 133.9, 133.5, 132.1, 128.9, 128.0, 126.3, 123.3, 122.4, 52.6, 51.2, 46.3, 38.4, 34.1, 33.3, 33.1, 25.9, 24.1, 21.6, 21.6, 15.0, -1.6, -1.7. HR-MS (ESI) m/z calcd for $C_{39}H_{46}N_4NaO_6Si$ [M+Na$^+$]: 717.3079, found: 717.3075.

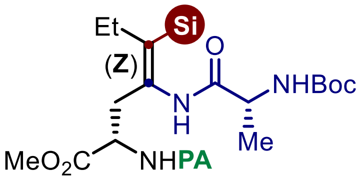

Methyl (*S*,*Z*)-4-{(*R*)-2-[(*tert*-butoxycarbonyl)amino]propanamido}-5-[dimethyl(phenyl)silyl]-2-(picolinamido)hept-4-enoate (8)

The general procedure **TP11** was followed using **1c** (26.0 mg, 0.1 mmol, 1.0 equiv.), **2e** (46.0 mg, 0.2 mmol, 2.0 equiv.), **3a** (0.5 mL, 0.2 mmol, 2.0 equiv.) and THF (0.5 mL) for 2 h. Purification by column chromatography (petroleum ether/EtOAc = 2:1) yielded **8** (38.0 mg, 65%) as a colorless oil. ^{1}H NMR (400 MHz, $CDCl_3$) δ = 8.62 – 8.57 (m, 1H), 8.54 (d, *J* = 9.1 Hz, 1H), 8.16 (d, *J* = 7.8 Hz, 1H), 7.87 (td, *J* = 7.7, 1.7 Hz, 1H), 7.47 (ddd, *J* = 7.6, 4.8, 1.2 Hz, 1H), 7.42 – 7.35 (m, 2H), 7.29 – 7.25 (m, 1H), 7.18 (dd, *J* = 13.3, 5.8 Hz, 3H), 5.04 (d, *J* = 5.5 Hz, 1H), 4.92 (td, *J* = 9.4, 5.7 Hz, 1H), 3.91 (p, *J* = 7.1 Hz, 1H), 3.74 (s, 3H), 3.25 (dd, *J* = 14.4, 5.3 Hz, 1H), 3.02 (dd, *J* = 14.4, 10.1 Hz, 1H), 2.19 (q, *J* = 7.5 Hz, 2H), 1.42 (s, 9H), 1.14 (d, *J* = 7.0 Hz, 3H), 0.92 (t, *J* = 7.5 Hz, 3H), 0.29 (s, 6H). ^{13}C NMR (101 MHz, $CDCl_3$) δ = 172.4, 171.7, 164.1, 155.2, 149.2, 148.4, 139.9, 138.5, 137.8, 137.4, 133.5, 129.3, 128.2, 126.6, 122.5, 79.7, 52.6, 50.4, 32.7, 28.4, 24.0, 18.2, 14.9, -1.6, -1.6. HR-MS (ESI) m/z calcd for $C_{30}H_{42}N_4NaO_6Si$ [M+Na$^+$]: 605.2766, found: 605.2763.

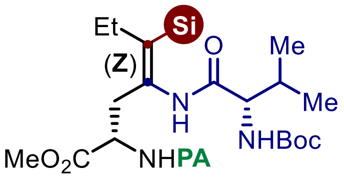

Methyl (*S*,*Z*)-4-{(*S*)-2-[(*tert*-butoxycarbonyl)amino]-3-methylbutanamido}-5-[dimethyl(phenyl)silyl]-2-(picolinamido)hept-4-enoate (9)

The general procedure **TP11** was followed using **1c** (26.0 mg, 0.1 mmol, 1.0 equiv.), **2f** (51.7 mg, 0.2 mmol, 2.0 equiv.), **3a** (0.5 mL, 0.2 mmol, 2.0 equiv.) and THF (0.5 mL) for 2 h. Purification by column chromatography (petroleum ether/EtOAc = 2:1) yielded **9** (39.0 mg, 64%) as a colorless oil. ^{1}H NMR (400 MHz, $CDCl_3$) δ = 8.58 (t, *J* = 5.8 Hz, 2H), 8.13 (d, *J* = 7.8 Hz, 1H), 7.88 (td, *J* = 7.7, 1.6 Hz, 1H), 7.52 – 7.43 (m,

1H), 7.37 (d, J = 6.7 Hz, 2H), 7.33 – 7.27 (m, 1H), 7.22 (t, J = 7.2 Hz, 2H), 7.12 (s, 1H), 4.96 (d, J = 9.0 Hz, 1H), 4.85 (ddd, J = 11.1, 9.0, 4.4 Hz, 1H), 3.77 (dd, J = 9.2, 5.0 Hz, 1H), 3.74 (s, 3H), 3.12 (dd, J = 14.1, 4.2 Hz, 1H), 2.92 (dd, J = 14.0, 11.2 Hz, 1H), 2.24 – 2.07 (m, 2H), 1.96 (td, J = 13.0, 6.6 Hz, 1H), 1.43 (s, 9H), 0.91 – 0.85 (m, 6H), 0.79 (d, J = 6.8 Hz, 3H), 0.29 (s, 3H), 0.27 (s, 3H). ^{13}C NMR (101 MHz, $CDCl_3$) δ = 172.3, 170.8, 164.1, 155.7, 149.2, 148.4, 139.5, 139.2, 138.3, 137.4, 133.6, 129.3, 128.2, 126.5, 122.4, 79.4, 59.3, 52.7, 50.6, 33.5, 31.4, 28.4, 24.1, 19.6, 17.2, 14.9, -1.6, -2.2. HR-MS (ESI) m/z calcd for $C_{32}H_{46}N_4NaO_6Si$ [M+Na$^+$]: 633.3079, found: 633.3080.

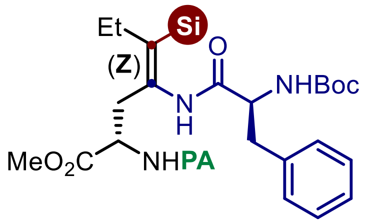

Methyl (*S*,*Z*)-4-{(*S*)-2-[(*tert*-butoxycarbonyl)amino]-3-phenylpropanamido}-5-[dimethyl(phenyl)silyl]-2-(picolinamido)hept-4-enoate (10)

The general procedure **TP11** was followed using **1c** (26.0 mg, 0.1 mmol, 1.0 equiv.), **2g** (61.2 mg, 0.2 mmol, 2.0 equiv.), **3a** (0.5 mL, 0.2 mmol, 2.0 equiv.) and THF (0.5 mL) for 2 h. Purification by column chromatography (petroleum ether/EtOAc = 2:1) yielded **10** (46.0 mg, 70%) as a colorless oil. ^{1}H NMR (400 MHz, $CDCl_3$) δ = 8.55 (d, J = 4.2 Hz, 1H), 8.47 (d, J = 8.4 Hz, 1H), 8.14 (d, J = 7.8 Hz, 1H), 7.87 (td, J = 7.7, 1.6 Hz, 1H), 7.44 (dd, J = 6.5, 4.9 Hz, 1H), 7.36 (d, J = 6.3 Hz, 3H), 7.30 (d, J = 7.3 Hz, 1H), 7.24 (dd, J = 13.7, 6.2 Hz, 4H), 7.17 (d, J = 7.3 Hz, 1H), 7.12 (d, J = 7.2 Hz, 2H), 4.68 (s, 1H), 4.50 (d, J = 6.8 Hz, 1H), 4.27 – 4.04 (m, 1H), 3.76 (s, 3H), 3.17 – 3.01 (m, 2H), 3.02 – 2.91 (m, 1H), 2.72 – 2.51 (m, 1H), 2.17 (qd, J = 13.6, 7.1 Hz, 2H), 1.34 (s, 9H), 0.90 (t, J = 7.5 Hz, 3H), 0.27 (s, 3H), 0.25 (s, 3H). ^{13}C NMR (101 MHz, $CDCl_3$) δ = 172.3, 170.4, 164.0, 155.2, 149.4, 148.4, 139.8, 138.9, 138.7, 137.3, 137.0, 133.6, 129.4, 129.3, 128.6, 128.2, 126.7, 126.4, 122.4, 79.8, 55.7, 52.6, 50.7, 38.1, 33.2, 28.3, 24.1, 14.9, -1.6, -1.7. HR-MS (ESI) m/z calcd for $C_{36}H_{46}N_4NaO_6Si$ [M+Na$^+$]: 681.3079, found: 681.3076.

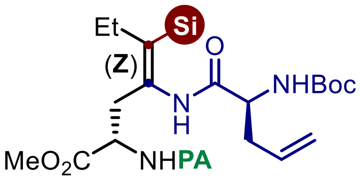

Methyl (*S*,*Z*)-4-{(*S*)-2-[(*tert*-butoxycarbonyl)amino]pent-4-enamido}-5-[dimethyl(phenyl)silyl]-2-(picolinamido)hept-4-enoate (11)

The general procedure **TP11** was followed using **1c** (39.0 mg, 0.15 mmol, 1.0 equiv.), **2h** (76.8 mg, 0.3 mmol, 2.0 equiv.), **3a** (1.0 mL, 0.3 mmol, 2.0 equiv.) and THF (0.5 mL) for 2 h. Purification by column chromatography (petroleum ether/EtOAc = 2:1) yielded **11** (48.4 mg, 53%) as a colorless oil. ^{1}H NMR (400 MHz, $CDCl_3$) δ = 8.67 – 8.54 (m, 2H), 8.14 (d, J = 7.7 Hz, 1H), 7.89 (td, J = 7.7, 1.3 Hz, 1H), 7.47 (dd, J = 7.3, 4.8 Hz, 1H), 7.43 – 7.34 (m, 3H), 7.31 (t, J = 7.2 Hz, 1H), 7.24 (t, J = 7.3 Hz, 2H), 5.65 (td, J = 17.1, 7.1 Hz, 1H), 5.08 (d, J = 5.8 Hz, 1H), 5.05 (s, 1H), 4.88 (td, J = 10.8, 4.5 Hz, 1H), 4.67 (d, J = 7.2 Hz, 1H), 3.92 (d, J = 5.3 Hz, 1H), 3.76 (s, 3H), 3.11 (dd, J = 14.1, 4.4 Hz, 1H), 2.94 (dd, J = 13.9, 11.1 Hz, 1H), 2.55 – 2.40 (m, 1H), 2.29 – 2.06 (m, 3H), 1.43 (s, 9H), 0.89 (t, J = 7.4 Hz, 3H), 0.29 (s, 3H), 0.28 (s, 3H). ^{13}C NMR (101 MHz, $CDCl_3$) δ = 172.4, 170.4, 164.0, 149.3, 148.4, 139.7, 138.6, 137.4, 133.6, 133.3, 129.2, 128.2, 126.5, 122.4, 118.8, 79.8, 53.9, 52.7, 50.6, 36.9, 33.4, 28.4, 24.1, 14.9, -1.6, -1.8. HR-MS (ESI) m/z calcd for $C_{32}H_{44}N_4NaO_6Si$ [M+Na$^+$]: 631.2922, found: 631.2927.

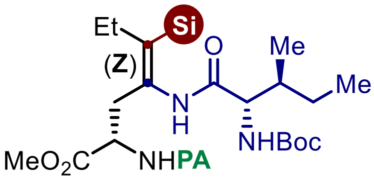

Methyl (*S*,*Z*)-4-{(2*S*,3*S*)-2-[(*tert*-butoxycarbonyl)amino]-3-methylpentanamido}-5-[dimethyl(phenyl)silyl]-2-(picolinamido)hept-4-enoate (12)

The general procedure **TP11** was followed using **1c** (26.0 mg, 0.1 mmol, 1.0 equiv.), **2i** (54.5 mg, 0.2 mmol, 2.0 equiv.), **3a** (0.5 mL, 0.2 mmol, 2.0 equiv.) and THF (0.5 mL) for 2 h. Purification by column chromatography (petroleum ether/EtOAc = 3:1) yielded **12** (40.0 mg, 64%) as a colorless oil. ^{1}H NMR (400 MHz, $CDCl_3$) δ = 8.63 –

8.54 (m, 2H), 8.15 (d, *J* = 7.7 Hz, 1H), 7.88 (td, *J* = 7.7, 1.7 Hz, 1H), 7.46 (ddd, *J* = 7.6, 4.8, 1.2 Hz, 1H), 7.42 (s, 1H), 7.41 (d, *J* = 1.3 Hz, 1H), 7.36 – 7.28 (m, 2H), 7.24 (t, *J* = 7.2 Hz, 2H), 4.85 (ddd, *J* = 10.6, 8.9, 4.8 Hz, 1H), 4.43 (d, *J* = 7.9 Hz, 1H), 4.02 – 3.82 (m, 1H), 3.76 (s, 3H), 3.14 (dd, *J* = 14.0, 4.5 Hz, 1H), 2.97 (dd, *J* = 14.0, 10.7 Hz, 1H), 2.18 (qd, *J* = 13.6, 7.1 Hz, 2H), 1.62 – 1.44 (m, 3H), 1.42 (s, 9H), 0.91 – 0.82 (m, 9H), 0.30 (s, 3H), 0.28 (s, 3H). ^{13}C NMR (101 MHz, $CDCl_3$) δ = 172.4, 171.8, 163.9, 155.4, 149.4, 148.4, 140.0, 138.8, 138.7, 137.3, 133.7, 129.2, 128.2, 126.4, 122.4, 79.7, 53.2, 52.6, 50.7, 41.5, 33.3, 28.4, 24.8, 24.0, 23.2, 21.7, 14.9, -1.5, -1.9. HR-MS (ESI) m/z calcd for $C_{33}H_{48}N_4NaO_6Si$ [M+Na$^+$]: 647.3235, found: 647.3237.

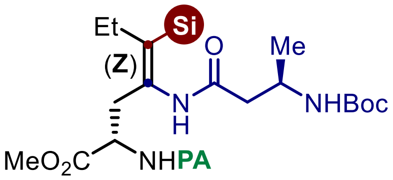

Methyl (*S*,*Z*)-4-{(*R*)-3-[(*tert*-butoxycarbonyl)amino]butanamido}-5-[dimethyl(phenyl)silyl]-2-(picolinamido)hept-4-enoate (13)

The general procedure **TP11** was followed using **1c** (26.0 mg, 0.1 mmol, 1.0 equiv.), **2j** (48.8 mg, 0.2 mmol, 2.0 equiv.), **3a** (0.5 mL, 0.2 mmol, 2.0 equiv.) and THF (0.5 mL) for 2 h. Purification by column chromatography (petroleum ether/EtOAc = 2:1) yielded **13** (40.0 mg, 67%) as a colorless oil. ^{1}H NMR (400 MHz, $CDCl_3$) δ = 8.56 (dt, *J* = 13.0, 4.9 Hz, 2H), 8.14 (d, *J* = 7.8 Hz, 1H), 7.87 (td, *J* = 7.7, 1.7 Hz, 1H), 7.46 (ddd, *J* = 7.6, 4.8, 1.2 Hz, 1H), 7.37 (dd, *J* = 7.9, 1.3 Hz, 2H), 7.28 (dd, *J* = 10.5, 4.3 Hz, 1H), 7.20 (t, *J* = 7.2 Hz, 2H), 6.67 (s, 1H), 5.53 (s, 1H), 4.89 (td, *J* = 10.1, 5.0 Hz, 1H), 3.85 (td, *J* = 12.2, 5.4 Hz, 1H), 3.74 (s, 3H), 3.21 (dd, *J* = 14.2, 5.0 Hz, 1H), 2.95 (dd, *J* = 14.1, 10.3 Hz, 1H), 2.39 – 2.10 (m, 2H), 1.97 – 1.75 (m, 2H), 1.42 (s, 9H), 1.15 (d, *J* = 6.7 Hz, 3H), 0.93 (t, *J* = 7.4 Hz, 3H), 0.29 (s, 3H), 0.26 (s, 3H). ^{13}C NMR (101 MHz, $CDCl_3$) δ = 172.3, 170.1, 164.0, 155.3, 149.2, 148.4, 140.1, 138.6, 137.7, 137.4, 133.4, 129.3, 128.3, 126.5, 122.5, 79.0, 52.6, 50.6, 43.8, 41.8, 33.0, 28.5, 23.9, 14.9, -1.8, -1.9. HR-MS (ESI) m/z calcd for $C_{31}H_{44}N_4NaO_6Si$ [M+Na$^+$]: 619.2922, found: 619.2925.

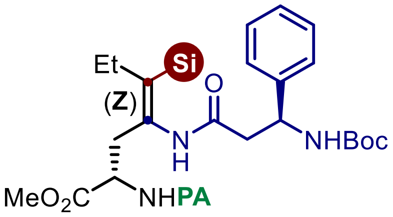

Methyl (*S*,*Z*)-4-{(*S*)-3-[(*tert*-butoxycarbonyl)amino]-3-phenylpropanamido}-5-[dimethyl(phenyl)silyl]-2-(picolinamido)hept-4-enoate (14)

The general procedure **TP11** was followed using **1c** (26.0 mg, 0.1 mmol, 1.0 equiv.), **2k** (61.3 mg, 0.2 mmol, 2.0 equiv.), **3a** (0.5 mL, 0.2 mmol, 2.0 equiv.) and THF (0.5 mL) for 2 h. Purification by column chromatography (petroleum ether/EtOAc = 2:1) yielded **14** (40.0 mg, 61%) as a colorless oil.^{1}H NMR (400 MHz, $CDCl_3$) δ = 8.35 (d, J = 4.3 Hz, 1H), 8.28 (d, J = 9.0 Hz, 1H), 7.89 (d, J = 7.8 Hz, 1H), 7.67 (td, J = 7.7, 1.7 Hz, 1H), 7.25 (ddd, J = 7.6, 4.8, 1.1 Hz, 1H), 7.11 – 7.04 (m, 7H), 6.98 (dd, J = 9.9, 4.7 Hz, 3H), 6.56 (s, 1H), 6.29 (s, 1H), 4.73 (s, 1H), 4.23 (s, 1H), 3.53 (s, 3H), 2.75 (d, J = 13.8 Hz, 1H), 2.62 (dd, J = 14.1, 10.9 Hz, 1H), 2.05 (dd, J = 27.1, 8.2 Hz, 2H), 1.95 – 1.79 (m, 2H), 1.18 (s, 9H), 0.66 (t, J = 7.5 Hz, 3H), 0.00 (s, 3H), -0.07 (s, 3H). ^{13}C NMR (101 MHz, $CDCl_3$) δ = 172.2, 169.8, 164.1, 155.3, 149.2, 148.4, 141.9, 139.7, 139.6, 138.8, 137.4, 133.4, 129.2, 128.6, 128.2, 127.0, 126.6, 126.1, 122.4, 79.2, 52.6, 51.5, 50.2, 41.9, 33.1, 28.4, 27.0, 23.9, 14.8, -2.0. HR-MS (ESI) m/z calcd for $C_{36}H_{46}N_4NaO_6Si$ [M+Na$^+$]: 681.3079, found: 681.3076.

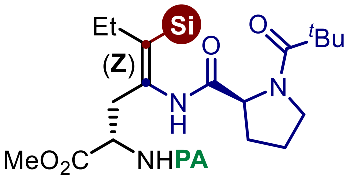

Methyl (*S*,*Z*)-5-[dimethyl(phenyl)silyl]-2-(picolinamido)-4-[(S)-1-pivaloylpyrrolidine-2-carboxamido]hept-4-enoate (15)

The general procedure **TP11** was followed using **1c** (26.0 mg, 0.1 mmol, 1.0 equiv.), **2l** (48.0 mg, 0.2 mmol, 2.0 equiv.), **3a** (0.5 mL, 0.2 mmol, 2.0 equiv.) and THF (0.5 mL) for 2 h. Purification by column chromatography (petroleum ether/EtOAc = 2:1) yielded **15** (42.0 mg, 71%, *Z/E* = 13:1) as a colorless oil. ^{1}H NMR (600 MHz, $CDCl_3$) δ = 8.61 – 8.54 (m, 2H), 8.12 (d, J = 7.8 Hz, 1H), 7.86 (td, J = 7.7, 1.5 Hz, 1H), 7.59 (s,

1H), 7.46 – 7.41 (m, 3H), 7.27 (t, *J* = 7.3 Hz, 1H), 7.22 (t, *J* = 7.3 Hz, 2H), 4.97 (ddd, *J* = 10.2, 8.9, 5.3 Hz, 1H), 4.30 (dd, *J* = 7.6, 5.6 Hz, 1H), 3.75 (s, 3H), 3.66 (dt, *J* = 10.1, 6.4 Hz, 1H), 3.39 (dt, *J* = 9.8, 6.5 Hz, 1H), 3.20 (dd, *J* = 14.0, 5.3 Hz, 1H), 2.94 (dd, *J* = 14.0, 10.5 Hz, 1H), 2.17 (dq, *J* = 15.0, 7.5 Hz, 1H), 2.08 (dq, *J* = 14.7, 7.4 Hz, 1H), 1.86 – 1.77 (m, 2H), 1.76 – 1.62 (m, 2H), 1.24 (s, 9H), 0.84 (t, *J* = 7.5 Hz, 3H), 0.35 (s, 3H), 0.29 (s, 3H). ^{13}C NMR (151 MHz, $CDCl_3$) δ = 177.8, 172.9, 171.7, 163.8, 149.7, 148.3, 140.8, 139.1, 138.6, 137.2, 133.8, 128.8, 127.8, 126.3, 122.2, 62.8, 52.5, 50.4, 48.3, 39.1, 33.5, 27.4, 27.2, 25.9, 24.1, 14.9, -1.1, -1.6. HR-MS (ESI) m/z calcd for $C_{32}H_{44}N_4NaO_5Si$ [M+Na$^+$]: 615.2973, found: 615.2979.

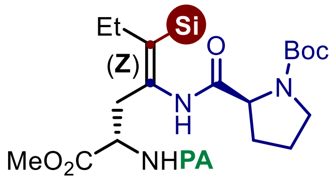

***tert*-Butyl (*R*)-2-{{{(*S*,*Z*)-3-[dimethyl(phenyl)silyl]-7-methoxy-7-oxo-6-(picolinamido)hept-3-en-4-yl}carbamoyl}}pyrrolidine-1-carboxylate (16)**

The general procedure **TP11** was followed using **1c** (26.0 mg, 0.1 mmol, 1.0 equiv.), **2m** (51.3 mg, 0.2 mmol, 2.0 equiv.), **3a** (0.5 mL, 0.2 mmol, 2.0 equiv.) and THF (0.5 mL) for 2 h. Purification by column chromatography (petroleum ether/EtOAc = 1.5:1) yielded **16** (50.5 mg, 83%) as a colorless oil. ^{1}H NMR (400 MHz, $CDCl_3$) δ = 8.60 (dd, *J* = 28.0, 13.1 Hz, 2H), 8.17 (d, *J* = 6.7 Hz, 1H), 7.86 (td, *J* = 7.7, 1.6 Hz, 1H), 7.49 – 7.36 (m, 3H), 7.32 – 7.26 (m, 1H), 7.21 (dd, *J* = 11.2, 4.4 Hz, 2H), 4.97 (s, 1H), 4.07 (dd, *J* = 8.6, 3.5 Hz, 1H), 3.76 (s, 3H), 3.38 – 2.83 (m, 4H), 2.33 – 2.05 (m, 2H), 2.01 – 1.66 (m, 3H), 1.67 – 1.45 (m, 2H), 1.40 (s, 9H), 0.90 – 0.82 (m, 3H), 0.30 (s, 6H). ^{13}C NMR (101 MHz, $CDCl_3$) δ = 172.7, 172.2, 164.0, 155.4, 149.7, 148.3, 140.7, 138.6, 137.3, 133.7, 128.8, 127.9, 126.3, 122.3, 80.1, 61.2, 52.6, 50.7, 47.1, 33.3, 29.1, 28.4, 24.4, 24.2, 14.9, -1.2, -1.3. HR-MS (ESI) m/z calcd for $C_{32}H_{44}N_4NaO_6Si$ [M+Na$^+$]: 631.2922, found: 631.2925.

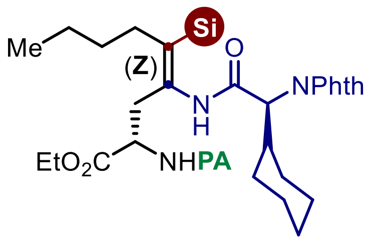

Ethyl (*S*,*Z*)-4-[(*S*)-2-cyclohexyl-2-(1,3-dioxoisoindolin-2-yl)acetamido]-5-[dimethyl(phenyl)silyl]-2-(picolinamido)non-4-enoate (17)

The general procedure **TP11** was followed using **1d** (30.2 mg, 0.1 mmol, 1.0 equiv.), **2n** (65.7 mg, 0.2 mmol, 2.0 equiv.), **3a** (0.5 mL, 0.2 mmol, 2.0 equiv.) and THF (0.5 mL) for 2 h. Purification by column chromatography (petroleum ether/EtOAc = 3:1) yielded **17** (54.0 mg, 75%) as a colorless oil. ^{1}H NMR (400 MHz, $CDCl_3$) δ = 8.60 – 8.53 (m, 2H), 8.10 (d, *J* = 7.8 Hz, 1H), 7.86 (td, *J* = 7.7, 1.4 Hz, 1H), 7.78 (dt, *J* = 7.3, 3.7 Hz, 2H), 7.71 (dd, *J* = 5.4, 3.1 Hz, 2H), 7.66 (s, 1H), 7.45 (dd, *J* = 7.1, 5.0 Hz, 1H), 7.28 (d, *J* = 6.8 Hz, 2H), 7.10 (t, *J* = 7.3 Hz, 1H), 7.03 (t, *J* = 7.2 Hz, 2H), 4.95 (td, *J* = 10.7, 4.7 Hz, 1H), 4.27 – 4.14 (m, 3H), 3.19 (dd, *J* = 14.1, 4.7 Hz, 1H), 2.91 (dd, *J* = 14.0, 10.9 Hz, 1H), 2.48 (q, *J* = 11.0 Hz, 1H), 2.10 – 2.01 (m, 2H), 1.86 (d, *J* = 12.9 Hz, 1H), 1.63 (dd, *J* = 25.3, 12.3 Hz, 4H), 1.50 (d, *J* = 12.0 Hz, 1H), 1.27 (dd, *J* = 15.1, 8.0 Hz, 6H), 1.20 – 1.10 (m, 5H), 0.75 (t, *J* = 6.9 Hz, 3H), 0.25 (s, 3H), 0.21 (s, 3H). ^{13}C NMR (101 MHz, $CDCl_3$) δ = 172.1, 168.0, 167.6, 164.1, 149.4, 148.4, 140.2, 138.4, 138.3, 137.3, 133.9, 133.5, 131.9, 128.8, 127.8, 126.4, 123.5, 122.4, 61.7, 60.5, 50.7, 35.9, 33.7, 32.5, 31.2, 31.1, 29.6, 26.1, 25.6, 22.9, 14.2, 13.9, -1.3, -1.5. HR-MS (ESI) m/z calcd for $C_{41}H_{50}N_4NaO_6Si$ [M+Na$^+$]: 745.3392, found: 745.3399.

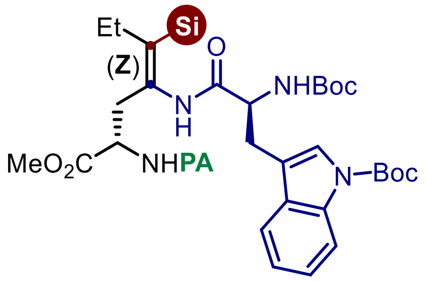

***tert*-Butyl 3-{{{(*S*)-2-[(*tert*-butoxycarbonyl)amino]-3-{{{(*S*,*Z*)-3-[dimethyl(phenyl)silyl]-7-methoxy-7-oxo-6-(picolinamido)hept-3-en-4-yl}amino}}-3-oxopropyl}}}-1H-indole-1-carboxylate (18)**

The general procedure **TP11** was followed using **1c** (39.0 mg, 0.15 mmol, 1.0 equiv.), **2o** (133.5 mg, 0.3 mmol, 2.0 equiv.), **3a** (1.0 mL, 0.3 mmol, 2.0 equiv.) and THF (0.5 mL) for 2 h. Purification by column chromatography (petroleum ether/EtOAc = 2.5:1) yielded **18** (62.2 mg, 52%) as a white solid. ^{1}H NMR (400 MHz, $CDCl_3$) δ = 8.53 (d, J = 4.3 Hz, 2H), 8.12 (d, J = 7.6 Hz, 2H), 7.86 (td, J = 7.7, 1.4 Hz, 1H), 7.51 (d, J = 7.7 Hz, 1H), 7.43 (s, 3H), 7.33 – 7.22 (m, 5H), 7.15 (t, J = 6.8 Hz, 2H), 4.78 (s, 1H), 4.52 (d, J = 7.7 Hz, 1H), 4.25 (s, 1H), 3.71 (s, 3H), 3.11 (t, J = 12.5 Hz, 2H), 3.02 – 2.86 (m, 1H), 2.77 (dd, J = 13.9, 7.9 Hz, 1H), 2.19 (dd, J = 16.2, 8.6 Hz, 2H), 1.64 (s, 9H), 1.37 (s, 9H), 0.91 (t, J = 7.2 Hz, 3H), 0.24 (s, 3H), 0.22 (s, 3H). ^{13}C NMR (101 MHz, $CDCl_3$) δ = 172.3, 170.4, 163.9, 155.3, 149.7, 149.4, 148.4, 139.8, 139.1, 138.7, 137.3, 135.6, 133.4, 130.6, 129.2, 128.2, 126.4, 124.5, 124.1, 122.6, 122.4, 119.3, 116.0, 115.2, 83.5, 79.8, 54.5, 52.6, 50.6, 33.4, 28.2, 27.5, 24.0, 14.9, -1.7, -1.9. HR-MS (ESI) m/z calcd for $C_{43}H_{55}N_5NaO_8Si$ [M+Na$^+$]: 820.3712, found: 820.3714.

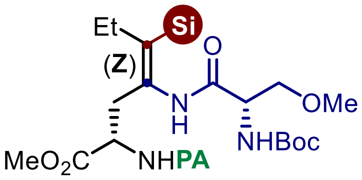

Methyl (*S*,*Z*)-4-{(*S*)-2-[(*tert*-butoxycarbonyl)amino]-3-methoxypropanamido}-5-[dimethyl(phenyl)silyl]-2-(picolinamido)hept-4-enoate (19)

The general procedure **TP11** was followed using **1c** (26.0 mg, 0.1 mmol, 1.0 equiv.), **2p** (52.0 mg, 0.2 mmol, 2.0 equiv.), **3a** (0.5 mL, 0.2 mmol, 2.0 equiv.) and THF (0.5 mL) for 2 h. Purification by column chromatography (petroleum ether/EtOAc = 2:1) yielded **19** (38.6 mg, 63%) as a colorless oil. ^{1}H NMR (400 MHz, $CDCl_3$) δ = 8.60 (d, J = 4.3 Hz, 1H), 8.53 (d, J = 8.7 Hz, 1H), 8.14 (d, J = 7.8 Hz, 1H), 7.88 (td, J = 7.7, 1.7 Hz, 1H), 7.59 (s, 1H), 7.46 (ddd, J = 7.6, 4.8, 1.2 Hz, 1H), 7.41 – 7.32 (m, 2H), 7.29 – 7.25 (m, 1H), 7.18 (t, J = 7.2 Hz, 2H), 5.31 (d, J = 6.3 Hz, 1H), 4.91 (ddd, J = 11.1, 8.9, 4.4 Hz, 1H), 4.02 (s, 1H), 3.75 (s, 3H), 3.66 (dd, J = 9.2, 3.9 Hz, 1H), 3.39 (dd, J = 9.2, 5.5 Hz, 1H), 3.24 (s, 3H), 3.13 (dd, J = 14.2, 4.4 Hz, 1H), 2.91 (dd, J = 14.1, 11.2 Hz, 1H), 2.29 – 2.10 (m, 2H), 1.42 (s, 9H), 0.89 (t, J = 7.4 Hz, 3H), 0.31 (s, 3H), 0.28 (s, 3H). ^{13}C NMR (101 MHz, $CDCl_3$) δ = 172.5, 169.6, 164.1, 155.4, 149.5, 148.5, 140.2,

139.7, 138.7, 137.4, 133.6, 129.1, 128.0, 126.4, 122.5, 79.9, 72.4, 58.9, 54.2, 52.6, 50.7, 33.2, 28.4, 24.2, 14.9, -1.5, -1.9. HR-MS (ESI) m/z calcd for $C_{31}H_{44}N_4NaO_7Si$ [M+Na$^+$]: 635.2871, found: 635.2870.

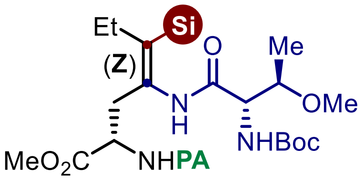

Methyl (*S*,*Z*)-4-{(2*S*,3*R*)-2-[(*tert*-butoxycarbonyl)amino]-3-methoxybutanamido}-5-[dimethyl(phenyl)silyl]-2-(picolinamido)hept-4-enoate (20)

The general procedure **TP11** was followed using **1c** (26.0 mg, 0.1 mmol, 1.0 equiv.), **2q** (54.9 mg, 0.2 mmol, 2.0 equiv.), **3a** (0.5 mL, 0.2 mmol, 2.0 equiv.) and THF (0.5 mL) for 2 h. Purification by column chromatography (petroleum ether/EtOAc = 2:1) yielded **20** (38.0 mg, 61%) as a colorless oil. ^{1}H NMR (400 MHz, $CDCl_3$) δ = 8.60 (d, J = 4.2 Hz, 1H), 8.52 (d, J = 8.5 Hz, 1H), 8.16 (d, J = 7.8 Hz, 1H), 7.88 (td, J = 7.7, 1.6 Hz, 1H), 7.63 (s, 1H), 7.50 – 7.42 (m, 1H), 7.38 (d, J = 6.7 Hz, 2H), 7.25 (d, J = 7.2 Hz, 1H), 7.19 (t, J = 7.1 Hz, 2H), 5.37 (d, J = 7.8 Hz, 1H), 4.93 (ddd, J = 11.1, 8.8, 4.7 Hz, 1H), 3.94 (dd, J = 10.6, 8.2 Hz, 1H), 3.89 (dd, J = 6.2, 2.8 Hz, 1H), 3.74 (s, 3H), 3.25 (s, 3H), 3.07 (dd, J = 14.1, 4.7 Hz, 1H), 2.99 – 2.83 (m, 1H), 2.30 – 2.07 (m, 2H), 1.42 (s, 9H), 1.10 (d, J = 6.3 Hz, 3H), 0.91 (t, J = 7.3 Hz, 3H), 0.31 (s, 3H), 0.29 (s, 3H). ^{13}C NMR (101 MHz, $CDCl_3$) δ = 172.4, 169.6, 164.2, 155.7, 149.6, 148.4, 140.3, 139.7, 138.7, 137.3, 133.5, 129.0, 128.0, 126.3, 122.5, 79.7, 76.1, 57.9, 56.5, 52.6, 51.1, 33.5, 28.4, 24.2, 14.9, 14.6, -1.5, -2.0. HR-MS (ESI) m/z calcd for $C_{32}H_{46}N_4NaO_7Si$ [M+Na$^+$]: 649.3028, found: 649.3025.

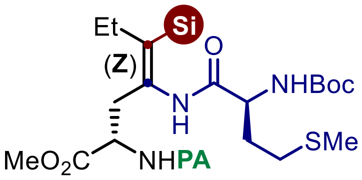

Methyl (*S*,*Z*)-4-{(*S*)-2-[(*tert*-butoxycarbonyl)amino]-4-(methylthio)butanamido}-5-[dimethyl(phenyl)silyl]-2-(picolinamido)hept-4-enoate (21)

The general procedure **TP11** was followed using **1c** (26.0 mg, 0.1 mmol, 1.0 equiv.), **2r** (58.1 mg, 0.2 mmol, 2.0 equiv.), **3a** (0.5 mL, 0.2 mmol, 2.0 equiv.) and THF (0.5 mL) for 2 h. Purification by column chromatography (petroleum ether/EtOAc = 2:1) yielded **21** (32.0 mg, 50%) as a colorless oil. ^{1}H NMR (400 MHz, $CDCl_3$) δ = 8.64 – 8.55 (m, 2H), 8.13 (d, J = 7.8 Hz, 1H), 7.88 (td, J = 7.7, 1.7 Hz, 1H), 7.47 (ddd, J = 7.6, 4.8, 1.1 Hz, 1H), 7.42 – 7.38 (m, 2H), 7.37 – 7.29 (m, 2H), 7.24 (dd, J = 11.5, 4.7 Hz, 2H), 4.85 (ddd, J = 10.7, 8.9, 4.6 Hz, 1H), 4.76 (d, J = 7.1 Hz, 1H), 3.97 (dt, J = 12.3, 6.1 Hz, 1H), 3.75 (s, 3H), 3.11 (dd, J = 14.2, 4.6 Hz, 1H), 2.94 (dd, J = 14.1, 10.8 Hz, 1H), 2.44 (t, J = 7.6 Hz, 2H), 2.18 (ddd, J = 24.2, 12.9, 6.5 Hz, 2H), 2.08 – 1.99 (m, 4H), 1.65 – 1.57 (m, 1H), 1.44 (s, 9H), 0.90 (t, J = 7.5 Hz, 3H), 0.29 (s, 3H), 0.28 (s, 3H). ^{13}C NMR (101 MHz, $CDCl_3$) δ = 172.3, 170.5, 164.0, 155.3, 149.3, 148.5, 139.6, 139.5, 138.6, 137.4, 133.6, 129.3, 128.2, 126.5, 122.4, 79.9, 53.7, 52.7, 50.6, 33.5, 32.2, 30.1, 28.4, 24.1, 15.4, 14.9, -1.6, -1.9. HR-MS (ESI) m/z calcd for $C_{32}H_{46}N_4NaO_6SSi$ [M+Na$^+$]: 665.2800, found: 665.2802.

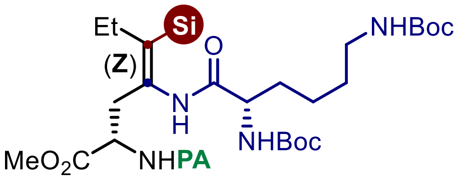

Methyl (*S*,*Z*)-4-{(*S*)-2,6-bis[(*tert*-butoxycarbonyl)amino]hexanamido}-5-[dimethyl(phenyl)silyl]-2-(picolinamido)hept-4-enoate (22)

The general procedure **TP11** was followed using **1c** (26.0 mg, 0.1 mmol, 1.0 equiv.), **2s** (77.5 mg, 0.2 mmol, 2.0 equiv.), **3a** (0.5 mL, 0.2 mmol, 2.0 equiv.) and THF (0.5 mL) for 2 h. Purification by column chromatography (petroleum ether/EtOAc = 2:1) yielded **22** (47.1 mg, 64%) as a colorless oil. ^{1}H NMR (400 MHz, $CDCl_3$) δ = 8.65 – 8.51 (m, 2H), 8.15 (d, J = 7.7 Hz, 1H), 7.95 – 7.83 (m, 1H), 7.48 (dd, J = 6.6, 5.0 Hz, 1H), 7.38 (d, J = 6.9 Hz, 2H), 7.32 (t, J = 7.2 Hz, 1H), 7.25 (dd, J = 13.9, 6.6 Hz, 2H), 7.16 (s, 1H), 5.03 – 4.66 (m, 3H), 3.76 (s, 4H), 3.16 (dd, J = 14.1, 4.2 Hz, 1H), 3.07 (s, 2H), 2.94 (dd, J = 13.9, 11.2 Hz, 1H), 2.18 (qd, J = 13.6, 7.2 Hz, 2H), 1.75 – 1.63 (m, 1H), 1.44 (s, 9H), 1.41 (s, 9H), 1.39 – 1.18 (m, 5H), 0.90 (t, J = 7.5 Hz, 3H), 0.29 (s, 3H), 0.26 (s, 3H). ^{13}C NMR (101 MHz, $CDCl_3$) δ = 172.3, 170.9, 164.0, 156.1, 155.4,

149.2, 148.4, 139.6, 138.9, 138.5, 137.4, 133.6, 129.3, 128.3, 126.5, 122.4, 79.7, 78.9, 54.2, 52.7, 50.5, 40.3, 33.3, 32.5, 29.4, 28.5, 28.4, 24.0, 22.7, 14.9, -1.6, -2.1. HR-MS (ESI) m/z calcd for $C_{38}H_{57}N_5NaO_8Si$ [M+Na$^+$]: 762.3869, found: 762.3865.

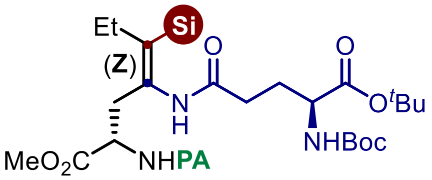

Methyl (*S*,*Z*)-4-{(*S*)-5-(*tert*-butoxy)-4-[(*tert*-butoxycarbonyl)amino]-5-oxopentanamido}-5-[dimethyl(phenyl)silyl]-2-(picolinamido)hept-4-enoate (23)

The general procedure **TP11** was followed using **1c** (26.0 mg, 0.1 mmol, 1.0 equiv.), **2t** (68.9 mg, 0.2 mmol, 2.0 equiv.), **3a** (0.5 mL, 0.2 mmol, 2.0 equiv.) and THF (0.5 mL) for 2 h. Purification by column chromatography (petroleum ether/EtOAc = 1.5:1) yielded **23** (57.3 mg, 82%) as a colorless oil. ^{1}H NMR (400 MHz, $CDCl_3$) δ = 8.58 (d, J = 4.3 Hz, 1H), 8.53 (d, J = 8.5 Hz, 1H), 8.13 (d, J = 7.8 Hz, 1H), 7.87 (dd, J = 10.9, 4.5 Hz, 1H), 7.47 (dd, J = 7.0, 5.3 Hz, 1H), 7.38 (d, J = 7.0 Hz, 2H), 7.29 (dd, J = 12.8, 5.5 Hz, 1H), 7.21 (t, J = 7.2 Hz, 2H), 6.65 (s, 1H), 5.12 (d, J = 7.6 Hz, 1H), 4.86 (td, J = 9.6, 5.1 Hz, 1H), 4.05 (s, 1H), 3.73 (s, 3H), 3.37 – 3.11 (m, 1H), 2.98 (dd, J = 14.2, 10.1 Hz, 1H), 2.19 (dt, J = 8.1, 5.5 Hz, 2H), 2.00 – 1.92 (m, 1H), 1.90 – 1.82 (m, 2H), 1.78 – 1.69 (m, 1H), 1.46 (s, 9H), 1.45 (s, 9H), 0.94 (t, J = 7.5 Hz, 3H), 0.30 (s, 3H), 0.28 (s, 3H). ^{13}C NMR (101 MHz, $CDCl_3$) δ = 172.3, 171.5, 171.0, 164.1, 155.6, 149.3, 148.4, 140.1, 138.7, 138.3, 137.4, 133.4, 129.3, 128.3, 126.5, 122.4, 82.0, 79.7, 53.7, 52.6, 50.6, 33.0, 32.2, 28.4, 28.1, 28.0, 23.9, 14.9, -1.8, -1.9. HR-MS (ESI) m/z calcd for $C_{36}H_{52}N_4NaO_8Si$ [M+Na$^+$]: 719.3447, found: 719.3449.

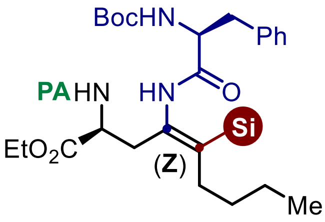

Ethyl (*S*,*Z*)-4-{(*S*)-2-[(*tert*-butoxycarbonyl)amino]-3-phenylpropanamido}-5-[dimethyl(phenyl)silyl]-2-(picolinamido)non-4-enoate (24)

The general procedure **TP11** was followed using **1c** (26.0 mg, 0.1 mmol, 1.0 equiv.), **2g** (61.2 mg, 0.2 mmol, 2.0 equiv.), **3a** (0.5 mL, 0.2 mmol, 2.0 equiv.) and THF (0.5 mL) for 2 h. Purification by column chromatography (petroleum ether/EtOAc = 4:1) yielded **24** (50.0 mg, 71%) as a colorless oil. ^{1}H NMR (400 MHz, $CDCl_3$) δ = 8.56 (d, J = 4.2 Hz, 1H), 8.47 (d, J = 8.5 Hz, 1H), 8.14 (d, J = 7.7 Hz, 1H), 7.88 (dd, J = 10.9, 4.5 Hz, 1H), 7.45 (d, J = 7.7 Hz, 2H), 7.35 (d, J = 6.9 Hz, 2H), 7.32 – 7.22 (m, 5H), 7.15 (dd, J = 16.2, 7.4 Hz, 3H), 4.65 (s, 1H), 4.54 (d, J = 7.6 Hz, 1H), 4.22 (dt, J = 16.7, 8.3 Hz, 3H), 3.06 (dd, J = 13.7, 5.3 Hz, 2H), 2.98 – 2.86 (m, 1H), 2.76 – 2.52 (m, 1H), 2.10 (s, 2H), 1.37 – 1.20 (m, 16H), 0.80 (t, J = 6.8 Hz, 3H), 0.26 (s, 3H), 0.25 (s, 3H). ^{13}C NMR (101 MHz, $CDCl_3$) δ = 171.9, 170.4, 164.0, 148.4, 139.9, 138.8, 138.2, 137.3, 137.0, 133.6, 129.4, 129.2, 128.6, 128.1, 126.7, 126.3, 122.4, 79.7, 61.8, 50.7, 38.2, 33.6, 32.6, 31.0, 28.3, 23.0, 14.3, 13.9, -1.6, -1.7. HR-MS (ESI) m/z calcd for $C_{39}H_{52}N_4NaO_6Si$ [M+Na$^+$]: 723.3548, found: 723.3553.

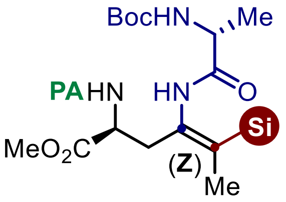

Methyl (*S*,*Z*)-4-{(*R*)-2-[(*tert*-butoxycarbonyl)amino]propanamido}-5-[dimethyl(phenyl)silyl]-2-(picolinamido)hex-4-enoate (25)

The general procedure **TP11** was followed using **1b** (24.6 mg, 0.1 mmol, 1.0 equiv.), **2e** (46.0 mg, 0.2 mmol, 2.0 equiv.), **3a** (0.5 mL, 0.2 mmol, 2.0 equiv.) and THF (0.5 mL) for 2 h. Purification by column chromatography (petroleum ether/EtOAc = 2:1) yielded **25** (41.0 mg, 72%) as a colorless oil. ^{1}H NMR (400 MHz, $CDCl_3$) δ = 8.58 (d, J = 4.3 Hz, 1H), 8.54 (d, J = 8.9 Hz, 1H), 8.16 (d, J = 7.8 Hz, 1H), 7.87 (td, J = 7.7, 1.6 Hz, 1H), 7.50 – 7.45 (m, 1H), 7.41 – 7.36 (m, 2H), 7.30 – 7.27 (m, 1H), 7.24 – 7.15 (m, 3H), 4.96 (td, J = 9.3, 5.5 Hz, 2H), 3.97 – 3.87 (m, 1H), 3.74 (s, 3H), 3.20 (dd, J = 14.3, 5.2 Hz, 1H), 3.04 (dd, J = 14.3, 9.6 Hz, 1H), 1.76 (s, 3H), 1.43 (s, 9H), 1.16 (d, J = 7.0 Hz, 3H), 0.29 (s, 3H), 0.27 (s, 3H). ^{13}C NMR (101 MHz, $CDCl_3$) δ = 172.2, 171.6, 164.0, 155.1, 149.2, 148.3, 139.3, 138.2, 137.3, 133.3, 133.0, 129.2, 128.1, 126.4, 122.4,

79.7, 52.5, 50.4, 33.0, 28.2, 17.4, -2.0, -2.3. HR-MS (ESI) m/z calcd for $C_{29}H_{40}N_4NaO_6Si$ [M+Na^+]: 591.2609, found: 591.2603.

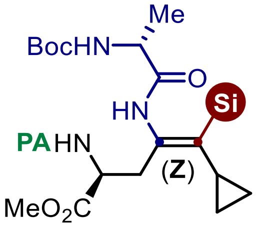

Methyl (*S*,*Z*)-4-{(*R*)-2-[(*tert*-butoxycarbonyl)amino]propanamido}-5-cyclopropyl-5-[dimethyl(phenyl)silyl]-2-(picolinamido)pent-4-enoate (26)

The general procedure **TP11** was followed using **1e** (27.2 mg, 0.1 mmol, 1.0 equiv.), **2e** (46.0 mg, 0.2 mmol, 2.0 equiv.), **3a** (0.5 mL, 0.2 mmol, 2.0 equiv.) and THF (0.5 mL) for 2 h. Purification by column chromatography (petroleum ether/EtOAc = 2:1) yielded **26** (42.0 mg, 71%) as a colorless oil. ^{1}H NMR (400 MHz, $CDCl_3$) δ = 8.55 (dd, J = 13.1, 6.4 Hz, 2H), 8.18 (d, J = 7.8 Hz, 1H), 7.88 (t, J = 7.6 Hz, 1H), 7.49 – 7.45 (m, 1H), 7.41 (d, J = 6.9 Hz, 2H), 7.26 (dd, J = 13.0, 5.8 Hz, 1H), 7.19 (t, J = 7.0 Hz, 2H), 7.02 (s, 1H), 5.00 – 4.80 (m, 2H), 3.85 – 3.77 (m, 1H), 3.75 (s, 3H), 3.31 (dd, J = 14.5, 5.0 Hz, 1H), 3.14 – 3.04 (m, 1H), 2.56 (s, 1H), 1.75 – 1.59 (m, 4H), 1.43 (s, 9H), 1.08 (d, J = 7.0 Hz, 3H), 0.36 (s, 6H). ^{13}C NMR (101 MHz, $CDCl_3$) δ = 172.2, 171.4, 164.0, 155.1, 149.3, 148.3, 141.8, 139.8, 139.5, 137.3, 133.1, 129.1, 128.3, 126.5, 122.4, 79.7, 52.6, 50.8, 50.3, 42.5, 31.7, 31.6, 28.4, 26.7, 25.9, 1.5, 0.7. HR-MS (ESI) m/z calcd for $C_{31}H_{42}N_4NaO_6Si$ [M+Na^+]: 617.2766, found: 617.2760.

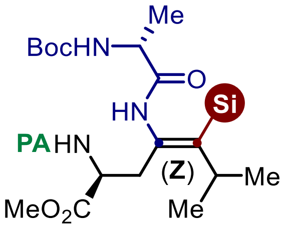

Methyl (*S*,*Z*)-4-{(*R*)-2-[(*tert*-butoxycarbonyl)amino]propanamido}-5-[dimethyl(phenyl)silyl]-6-methyl-2-(picolinamido)hept-4-enoate (27)

The general procedure **TP11** was followed using **1f** (27.4 mg, 0.1 mmol, 1.0 equiv.), **2e** (46.0 mg, 0.2 mmol, 2.0 equiv.), **3a** (0.5 mL, 0.2 mmol, 2.0 equiv.) and THF (0.5 mL) for 2 h. Purification by column chromatography (petroleum ether/EtOAc = 2:1)

yielded **27** (43.0 mg, 72%) as a colorless oil. ^{1}H NMR (400 MHz, $CDCl_3$) δ = 8.53 (dd, J = 14.6, 6.7 Hz, 2H), 8.16 (d, J = 7.8 Hz, 1H), 7.87 (td, J = 7.7, 1.5 Hz, 1H), 7.52 – 7.43 (m, 1H), 7.40 (d, J = 6.8 Hz, 2H), 7.23 (t, J = 7.4 Hz, 1H), 7.15 (t, J = 7.2 Hz, 2H), 7.01 (s, 1H), 5.03 – 4.77 (m, 2H), 3.84 – 3.75 (m, 1H), 3.73 (s, 3H), 3.27 (dd, J = 14.5, 5.5 Hz, 1H), 3.11 – 2.88 (m, 2H), 1.41 (s, 9H), 1.14 (d, J = 7.0 Hz, 3H), 1.06 (dd, J = 6.9, 4.4 Hz, 6H), 0.33 (d, J = 1.6 Hz, 6H). ^{13}C NMR (101 MHz, $CDCl_3$) δ = 172.2, 171.4, 164.1, 155.1, 149.2, 148.4, 143.0, 139.7, 139.0, 137.4, 133.1, 129.1, 128.3, 126.6, 122.5, 79.7, 52.6, 50.8, 50.3, 32.9, 31.1, 28.4, 22.2, 22.2, 18.1, 1.2, 0.4. HR-MS (ESI) m/z calcd for $C_{31}H_{44}N_4NaO_6Si$ [M+Na$^+$]: 619.2922, found: 619.2929.

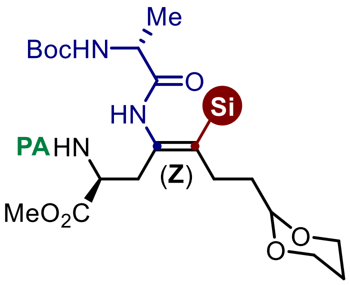

Methyl (*S*,*Z*)-4-{(*R*)-2-[(*tert*-butoxycarbonyl)amino]propanamido}-5-[dimethyl(phenyl)silyl]-7-(1,3-dioxan-2-yl)-2-(picolinamido)hept-4-enoate (28)

The general procedure **TP11** was followed using **1g** (51.9 mg, 0.15 mmol, 1.0 equiv.), **2e** (69.0 mg, 0.3 mmol, 2.0 equiv.), **3a** (1.0 mL, 0.3 mmol, 2.0 equiv.) and THF (0.5 mL) for 2 h. Purification by column chromatography (petroleum ether/EtOAc = 1.5:1) yielded **28** (44.2 mg, 44%) as a colorless oil. ^{1}H NMR (400 MHz, $CDCl_3$) δ = 8.60 (d, J = 4.7 Hz, 1H), 8.55 (d, J = 9.2 Hz, 1H), 8.14 (d, J = 7.8 Hz, 1H), 7.87 (t, J = 7.7 Hz, 1H), 7.49 – 7.44 (m, 1H), 7.34 (d, J = 7.1 Hz, 2H), 7.23 (d, J = 7.5 Hz, 1H), 7.18 (s, 1H), 7.12 (t, J = 7.3 Hz, 2H), 5.09 (d, J = 7.3 Hz, 1H), 4.92 (dd, J = 14.4, 9.4 Hz, 1H), 4.45 (t, J = 4.9 Hz, 1H), 4.08 (dd, J = 11.3, 4.6 Hz, 2H), 3.95 – 3.88 (m, 1H), 3.73 (s, 3H), 3.26 (dd, J = 14.5, 5.0 Hz, 1H), 3.07 (dd, J = 14.4, 10.3 Hz, 1H), 2.36 – 2.23 (m, 2H), 2.10 – 2.00 (m, 1H), 1.90 – 1.80 (m, 1H), 1.76 – 1.63 (m, 1H), 1.59 – 1.50 (m, 3H), 1.41 (s, 9H), 1.14 (d, J = 7.0 Hz, 3H), 0.28 (d, J = 3.3 Hz, 6H). ^{13}C NMR (101 MHz, $CDCl_3$) δ = 172.3, 171.7, 164.2, 149.2, 148.5, 140.9, 138.3, 137.4, 135.6, 133.4, 129.3, 128.2, 126.6, 122.5, 101.4, 79.7, 66.9, 52.6, 50.2, 35.2, 32.5, 28.4, 25.8, 25.2, -

1.6, -1.6. HR-MS (ESI) m/z calcd for $C_{34}H_{48}N_4NaO_8Si$ [M+Na$^+$]: 691.3134, found: 691.3126.

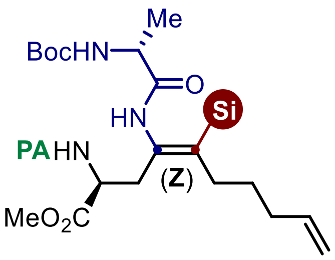

Methyl (*S*,*Z*)-4-{(*R*)-2-[(*tert*-butoxycarbonyl)amino]propanamido}-5-[dimethyl(phenyl)silyl]-2-(picolinamido)deca-4,9-dienoate (29)

The general procedure **TP11** was followed using **1h** (30.0 mg, 0.1 mmol, 1.0 equiv.), **2e** (46.0 mg, 0.2 mmol, 2.0 equiv.), **3a** (0.5 mL, 0.2 mmol, 2.0 equiv.) and THF (0.5 mL) for 2 h. Purification by column chromatography (petroleum ether/EtOAc = 3:1) yielded **29** (37.4 mg, 60%) as a colorless oil. ^{1}H NMR (400 MHz, $CDCl_3$) δ = 8.62 – 8.56 (m, 1H), 8.54 (d, J = 9.1 Hz, 1H), 8.16 (d, J = 7.8 Hz, 1H), 7.88 (td, J = 7.7, 1.7 Hz, 1H), 7.48 (ddd, J = 7.6, 4.8, 1.2 Hz, 1H), 7.40 – 7.35 (m, 2H), 7.29 – 7.24 (m, 2H), 7.17 (t, J = 7.3 Hz, 2H), 5.79 – 5.64 (m, 1H), 5.05 (s, 1H), 4.95 (dd, J = 20.0, 4.9 Hz, 3H), 4.00 – 3.88 (m, 1H), 3.75 (s, 3H), 3.24 (dd, J = 14.4, 5.1 Hz, 1H), 3.00 (dd, J = 14.4, 10.1 Hz, 1H), 2.25 – 2.08 (m, 2H), 1.99 (q, J = 7.2 Hz, 2H), 1.42 (s, 9H), 1.32 (dd, J = 14.9, 7.4 Hz, 2H), 1.14 (d, J = 7.0 Hz, 3H), 0.28 (d, J = 1.0 Hz, 6H). ^{13}C NMR (101 MHz, $CDCl_3$) δ = 172.4, 171.7, 164.1, 155.2, 149.2, 148.4, 140.3, 138.4, 138.2, 137.4, 133.5, 129.3, 128.2, 128.0, 126.6, 122.5, 115.0, 79.7, 52.6, 50.4, 33.9, 32.9, 30.6, 29.6, 28.4, 18.2, -1.6, -1.6. HR-MS (ESI) m/z calcd for $C_{33}H_{46}N_4NaO_6Si$ [M+Na$^+$]: 645.3079, found: 645.3086.

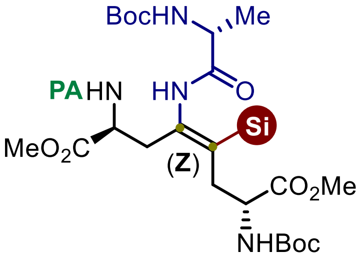

Dimethyl (2*R*,7*S*,*Z*)-2-[(*tert*-butoxycarbonyl)amino]-5-{(*R*)-2-[(*tert*-butoxycarbonyl)amino]propanamido}-4-[dimethyl(phenyl)silyl]-7-(picolinamido)oct-4-enedioate (30)

The general procedure **TP11** was followed using **1i** (43.4 mg, 0.1 mmol, 1.0 equiv.), **2e** (46.0 mg, 0.2 mmol, 2.0 equiv.), **3a** (0.5 mL, 0.2 mmol, 2.0 equiv.) and THF (0.5 mL) for 2 h. Purification by column chromatography (petroleum ether/EtOAc = 1.5:1) yielded **30** (57.0 mg, 50%) as a colorless oil. ^{1}H NMR (400 MHz, $CDCl_3$) δ = 8.52 (d, J = 4.4 Hz, 1H), 8.43 (d, J = 8.7 Hz, 1H), 8.07 (d, J = 7.8 Hz, 1H), 7.79 (td, J = 7.7, 1.1 Hz, 1H), 7.40 (dd, J = 6.9, 5.1 Hz, 1H), 7.36 (d, J = 7.0 Hz, 2H), 7.20 (d, J = 8.2 Hz, 1H), 7.11 (t, J = 7.1 Hz, 2H), 7.03 (s, 1H), 5.13 (d, J = 8.3 Hz, 1H), 4.90 (d, J = 6.0 Hz, 1H), 4.79 (dd, J = 14.6, 8.7 Hz, 1H), 4.22 (dd, J = 13.7, 7.1 Hz, 1H), 3.81 – 3.74 (m, 1H), 3.66 (d, J = 4.5 Hz, 6H), 3.23 (dd, J = 14.4, 5.0 Hz, 1H), 3.01 (dd, J = 14.6, 9.2 Hz, 1H), 2.60 (t, J = 10.4 Hz, 2H), 1.35 (s, 9H), 1.34 (s, 9H), 1.04 (d, J = 7.0 Hz, 3H), 0.29 (d, J = 3.1 Hz, 6H). ^{13}C NMR (101 MHz, $CDCl_3$) δ = 172.8, 172.0, 171.4, 164.2, 155.1, 149.2, 148.4, 144.0, 138.0, 137.4, 133.4, 129.6, 128.4, 126.6, 122.5, 80.0, 79.7, 53.6, 52.6, 52.4, 50.4, 34.1, 32.5, 28.3, 28.3, 18.2, -1.7, -2.1. HR-MS (ESI) m/z calcd for $C_{37}H_{53}N_5NaO_{10}Si$ [M+Na$^+$]: 778.3454, found: 778.3459.

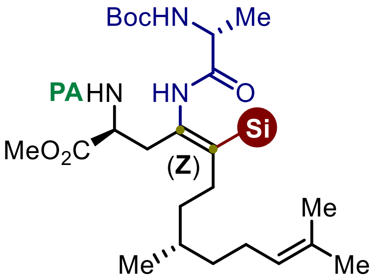

Methyl (2*S*,8*S*,*Z*)-4-{(*R*)-2-[(*tert*-butoxycarbonyl)amino]propanamido}-5-[dimethyl(phenyl)silyl]-8,12-dimethyl-2-(picolinamido)trideca-4,11-dienoate (31)

The general procedure **TP11** was followed using **1j** (37.0 mg, 0.1 mmol, 1.0 equiv.), **2e** (46.0 mg, 0.2 mmol, 2.0 equiv.), **3a** (0.5 mL, 0.2 mmol, 2.0 equiv.) and THF (0.5 mL) for 2 h. Purification by column chromatography (petroleum ether/EtOAc = 3:1) yielded **31** (44.0 mg, 63%) as a colorless oil. ^{1}H NMR (400 MHz, $CDCl_3$) δ = 8.59 (d, J = 4.1 Hz, 1H), 8.55 (d, J = 9.1 Hz, 1H), 8.16 (d, J = 7.8 Hz, 1H), 7.88 (td, J = 7.7, 1.6 Hz,

1H), 7.47 (ddd, *J* = 7.5, 4.8, 1.1 Hz, 1H), 7.38 (d, *J* = 6.8 Hz, 2H), 7.29 – 7.24 (m, 2H), 7.17 (t, *J* = 7.3 Hz, 2H), 5.07 (dd, *J* = 9.2, 3.5 Hz, 2H), 4.93 (dd, *J* = 13.7, 9.4 Hz, 1H), 3.94 (p, *J* = 7.0 Hz, 1H), 3.75 (s, 3H), 3.23 (dd, *J* = 14.4, 4.9 Hz, 1H), 2.98 (dd, *J* = 14.3, 10.2 Hz, 1H), 2.11 (dq, *J* = 12.9, 7.9 Hz, 2H), 2.00 – 1.86 (m, 2H), 1.69 (s, 3H), 1.60 (s, 3H), 1.42 (s, 9H), 1.40 – 1.18 (m, 4H), 1.15 (d, *J* = 7.0 Hz, 3H), 1.07 – 1.02 (m, 1H), 0.79 (d, *J* = 6.5 Hz, 3H), 0.29 (s, 6H). ^{13}C NMR (101 MHz, $CDCl_3$) δ = 172.3, 171.7, 164.1, 155.2, 149.2, 148.4, 139.8, 138.4, 137.4, 137.0, 133.5, 131.2, 129.2, 128.1, 126.6, 124.9, 122.5, 79.7, 52.6, 50.4, 37.6, 36.9, 33.0, 32.8, 28.8, 28.4, 25.8, 25.6, 19.4, 18.2, 17.8, -1.6, -1.6. HR-MS (ESI) m/z calcd for $C_{38}H_{56}N_4NaO_6Si$ [M+Na$^+$]: 715.3861, found: 715.3853.

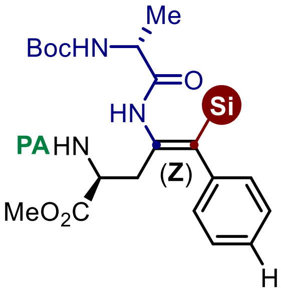

Methyl (*S*,*Z*)-4-{(*R*)-2-[(*tert*-butoxycarbonyl)amino]propanamido}-5-[dimethyl(phenyl)silyl]-5-phenyl-2-(picolinamido)pent-4-enoate (32)

The general procedure **TP11** was followed using **1k** (30.8 mg, 0.1 mmol, 1.0 equiv.), **2e** (46.0 mg, 0.2 mmol, 2.0 equiv.), **3a** (0.5 mL, 0.2 mmol, 2.0 equiv.) and THF (0.5 mL) for 2 h. Purification by column chromatography (petroleum ether/EtOAc = 2:1) yielded **32** (39.0 mg, 62%) as a colorless oil. ^{1}H NMR (400 MHz, $CDCl_3$) δ = 8.67 (d, *J* = 4.1 Hz, 1H), 8.38 (d, *J* = 9.6 Hz, 1H), 8.20 (d, *J* = 7.8 Hz, 1H), 7.91 (td, *J* = 7.7, 1.6 Hz, 1H), 7.52 (ddd, *J* = 7.6, 4.8, 1.1 Hz, 1H), 7.44 – 7.40 (m, 3H), 7.30 – 7.25 (m, 3H), 7.22 – 7.18 (m, 1H), 7.14 (t, *J* = 7.4 Hz, 2H), 7.01 (d, *J* = 7.2 Hz, 1H), 6.85 (d, *J* = 6.2 Hz, 1H), 5.18 (s, 1H), 4.95 – 4.85 (m, 1H), 4.00 – 3.89 (m, 1H), 3.65 (s, 3H), 3.28 (dd, *J* = 14.4, 4.7 Hz, 1H), 2.70 (dd, *J* = 14.3, 11.2 Hz, 1H), 1.43 (s, 9H), 1.19 (d, *J* = 7.0 Hz, 3H), 0.16 (s, 3H), 0.03 (s, 3H). ^{13}C NMR (101 MHz, $CDCl_3$) δ = 172.0, 171.7, 164.1, 155.3, 149.3, 148.5, 142.0, 141.1, 137.5, 137.5, 133.6, 129.5, 128.6, 128.6, 128.3,

127.8, 126.7, 126.1, 122.6, 79.8, 52.5, 50.6, 50.0, 34.1, 28.4, 18.2, -1.7, -1.8. HR-MS (ESI) m/z calcd for $C_{34}H_{42}N_4NaO_6Si$ [M+Na$^+$]: 653.2766, found: 653.2773.

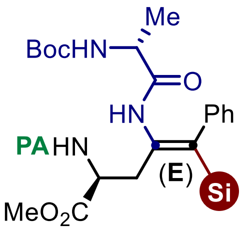

Methyl (*S,E*)-4-{(*R*)-2-[(*tert*-butoxycarbonyl)amino]propanamido}-5-[dimethyl(phenyl)silyl]-5-phenyl-2-(picolinamido)pent-4-enoate (32-1)

The general procedure **TP11** was followed using **1k** (30.8 mg, 0.1 mmol, 1.0 equiv.), **2e** (46.0 mg, 0.2 mmol, 2.0 equiv.), **3a** (0.5 mL, 0.2 mmol, 2.0 equiv.) and THF (0.5 mL) for 2 h. Purification by column chromatography (petroleum ether/EtOAc = 2:1) yielded **32-1** (3.0 mg, 5%) as a colorless oil. ^{1}H NMR (400 MHz, $CDCl_3$) δ = 8.60 (dd, J = 4.0, 0.8 Hz, 1H), 8.40 (d, J = 7.8 Hz, 1H), 8.33 (s, 1H), 8.19 (d, J = 7.8 Hz, 1H), 7.88 (td, J = 7.7, 1.7 Hz, 1H), 7.46 (ddd, J = 7.6, 4.8, 1.1 Hz, 1H), 7.42 – 7.38 (m, 2H), 7.28 – 7.26 (m, 3H), 7.22 – 7.17 (m, 1H), 7.13 (t, J = 7.5 Hz, 2H), 7.06 (d, J = 7.0 Hz, 2H), 5.38 (s, 1H), 4.96 – 4.81 (m, 1H), 4.31 (s, 1H), 3.72 (s, 3H), 2.77 (ddd, J = 23.4, 14.4, 7.9 Hz, 2H), 1.43 (d, J = 2.2 Hz, 3H), 1.38 (s, 9H), 0.15 (s, 3H), -0.04 (s, 3H). ^{13}C NMR (101 MHz, $CDCl_3$) δ = 172.5, 164.4, 150.0, 148.5, 145.9, 139.8, 139.1, 137.7, 134.0, 129.6, 128.9, 128.5, 127.9, 127.2, 122.7, 100.1, 80.2, 52.6, 52.1, 35.4, 28.4, 18.5, -0.9. HR-MS (ESI) m/z calcd for $C_{34}H_{42}N_4NaO_6Si$ [M+Na$^+$]: 653.2766, found: 653.2770.

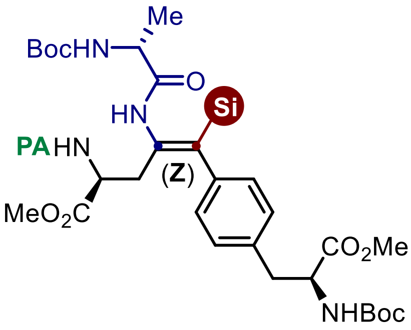

Methyl (*S*,*Z*)-5-{{4-{(*S*)-2-[(tert-butoxycarbonyl)amino]-3-methoxy-3-oxopropyl}phenyl}}-4-{(*R*)-2-[(tert-butoxycarbonyl)amino]propanamido}-5-[dimethyl(phenyl)silyl]-2-(picolinamido)pent-4-enoate (33)

The general procedure **TP11** was followed using **1l** (51.0 mg, 0.1 mmol, 1.0 equiv.), **2e** (46.0 mg, 0.2 mmol, 2.0 equiv.), **3a** (0.5 mL, 0.2 mmol, 2.0 equiv.) and THF (0.5 mL) for 2 h. Purification by column chromatography (petroleum ether/EtOAc = 1.5:1) yielded **33** (47.4 mg, 57%) as a colorless oil. ^{1}H NMR (400 MHz, $CDCl_3$) δ = 8.67 (d, J = 4.3 Hz, 1H), 8.37 (d, J = 9.6 Hz, 1H), 8.20 (d, J = 7.8 Hz, 1H), 7.92 (t, J = 7.7 Hz, 1H), 7.56 – 7.50 (m, 1H), 7.44 (s, 1H), 7.40 (d, J = 7.2 Hz, 2H), 7.29 – 7.26 (m, 1H), 7.15 (t, J = 7.3 Hz, 2H), 7.06 (d, J = 7.6 Hz, 2H), 6.94 (d, J = 6.9 Hz, 1H), 6.76 (d, J = 6.5 Hz, 1H), 5.17 (s, 1H), 5.05 (d, J = 7.8 Hz, 1H), 4.89 (t, J = 9.8 Hz, 1H), 4.59 (dd, J = 13.3, 6.7 Hz, 1H), 4.03 – 3.86 (m, 1H), 3.67 (s, 3H), 3.66 (s, 3H), 3.25 (dd, J = 14.3, 3.8 Hz, 1H), 3.11 – 2.98 (m, 2H), 2.74 – 2.63 (m, 1H), 1.43 (s, 18H), 1.19 (d, J = 6.9 Hz, 3H), 0.15 (s, 3H), 0.04 (s, 3H). ^{13}C NMR (101 MHz, $CDCl_3$) δ = 172.5, 171.9, 171.7, 164.1, 155.3, 155.1, 149.2, 148.5, 142.0, 139.8, 137.5, 133.9, 133.6, 133.1, 129.5, 128.8, 128.3, 128.0, 126.7, 122.6, 80.0, 79.8, 54.6, 52.5, 52.2, 50.6, 50.0, 38.3, 34.2, 28.4, 18.2, -1.8, -1.9. HR-MS (ESI) m/z calcd for $C_{43}H_{57}N_5NaO_{10}Si$ [M+Na$^+$]: 854.3767, found: 854.3757.

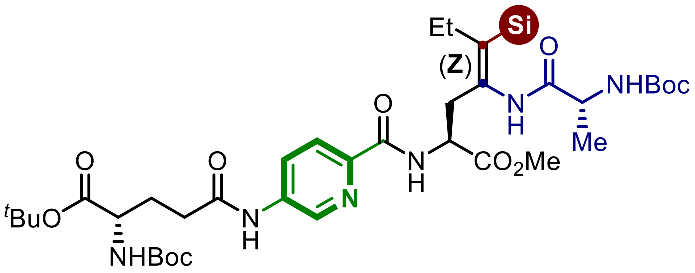

Methyl (*S*,*Z*)-2-{{5-{(*S*)-5-(*tert*-butoxy)-4-[(*tert*-butoxycarbonyl)amino]-5-oxopentanamido}picolinamido}}-4-{(*R*)-2-[(*tert*-butoxycarbonyl)amino]propanamido}-5-[dimethyl(phenyl)silyl]hept-4-enoate (34)

The general procedure **TP11** was followed using **1n** (57.4 mg, 0.1 mmol, 1.0 equiv.), **2e** (46.0 mg, 0.2 mmol, 2.0 equiv.), **3a** (0.5 mL, 0.2 mmol, 2.0 equiv.) and THF (0.5 mL) for 2 h. Purification by column chromatography (petroleum ether/EtOAc = 1:1)

yielded **34** (44.1 mg, 50%) as a colorless oil. ^{1}H NMR (400 MHz, $CDCl_3$) δ = 9.86 (s, 1H), 8.73 (s, 1H), 8.40 (dd, J = 15.2, 8.6 Hz, 2H), 8.11 (d, J = 8.5 Hz, 1H), 7.39 (d, J = 6.7 Hz, 2H), 7.31 – 7.27 (m, 2H), 7.20 (t, J = 7.2 Hz, 2H), 5.49 (d, J = 7.6 Hz, 1H), 5.09 (s, 1H), 4.91 (s, 1H), 4.22 (t, J = 7.6 Hz, 1H), 4.02 – 3.88 (m, 1H), 3.74 (s, 3H), 3.22 (dd, J = 14.5, 5.1 Hz, 1H), 2.98 (dd, J = 14.4, 10.3 Hz, 1H), 2.57 – 2.49 (m, 2H), 2.36 – 2.26 (m, 1H), 2.17 (q, J = 7.7 Hz, 2H), 1.87 – 1.82 (m, 1H), 1.50 (s, 9H), 1.46 (s, 9H), 1.42 (s, 9H), 1.15 (d, J = 7.0 Hz, 3H), 0.90 (t, J = 7.6 Hz, 3H), 0.29 (s, 6H). ^{13}C NMR (101 MHz, $CDCl_3$) δ = 172.5, 171.8, 171.5, 171.0, 164.0, 157.2, 155.2, 144.1, 139.9, 139.5, 138.5, 138.2, 133.5, 129.3, 128.2, 126.7, 123.1, 83.3, 81.2, 79.7, 53.1, 52.6, 50.4, 50.3, 34.3, 32.8, 31.2, 28.4, 28.4, 28.1, 24.0, 14.9, 14.2, -1.5, -1.6. HR-MS (ESI) m/z calcd for $C_{44}H_{66}N_6NaO_{11}Si$ [M+Na$^+$]: 905.4451, found: 905.4461.

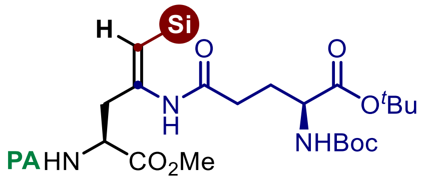

Methyl (*S*,*Z*)-4-{(S)-5-(*tert*-butoxy)-4-[(*tert*-butoxycarbonyl)amino]-5-oxopentanamido}-5-[dimethyl(phenyl)silyl]-2-(picolinamido)pent-4-enoate (35)

The general procedure **TP11** was followed using **1a** (34.8 mg, 0.15 mmol, 1.0 equiv.), **2t** (103.3 mg, 0.3 mmol, 2.0 equiv.), **3a** (1.0 mL, 0.3 mmol, 2.0 equiv.) and THF (0.5 mL) for 2 h. Purification by column chromatography (petroleum ether/EtOAc = 2:1) yielded **35** (75 mg, 75%) as a colorless oil. ^{1}H NMR (400 MHz, $CDCl_3$) δ = 8.58 (dd, J = 16.6, 6.2 Hz, 2H), 8.16 (d, J = 7.8 Hz, 1H), 7.87 (t, J = 7.4 Hz, 1H), 7.53 – 7.41 (m, 3H), 7.38 (t, J = 7.3 Hz, 1H), 7.34 – 7.26 (m, 2H), 6.79 (s, 1H), 5.08 (d, J = 8.0 Hz, 1H), 5.04 (s, 1H), 4.90 (td, J = 9.0, 5.0 Hz, 1H), 4.02 (s, 1H), 3.74 (s, 3H), 3.54 – 3.34 (m, 1H), 2.99 – 2.78 (m, 1H), 1.94 (t, J = 10.4 Hz, 1H), 1.82 – 1.61 (m, 3H), 1.46 (s, 18H), 0.26 (d, J = 20.3 Hz, 6H). ^{13}C NMR (101 MHz, $CDCl_3$) δ = 172.2, 171.5, 170.6, 164.2, 155.7, 149.5, 148.4, 146.8, 137.7, 137.4, 134.0, 129.9, 128.6, 126.5, 122.4, 117.4, 82.1, 79.8, 53.6, 52.5, 51.1, 40.1, 32.6, 28.4, 28.1, 22.7, -2.1, -2.3. HR-MS (ESI) m/z calcd for $C_{34}H_{49}N_4O_8Si$ [M+H$^+$]: 669.3314, found: 669.3318.

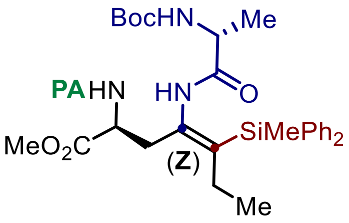

Methyl (*S*,*Z*)-4-{(*R*)-2-[(*tert*-butoxycarbonyl)amino]propanamido}-5-(methyldiphenylsilyl)-2-(picolinamido)hept-4-enoate (36)

The general procedure **TP11** was followed using **1c** (39.0 mg, 0.15 mmol, 1.0 equiv.), **2e** (69.0 mg, 0.3 mmol, 2.0 equiv.), **3b** (1.0 mL, 0.3 mmol, 2.0 equiv.) and THF (0.5 mL) for 2 h. Purification by column chromatography (petroleum ether/EtOAc = 2.5:1) yielded **36** (67.6 mg, 70%) as a colorless oil. ^{1}H NMR (600 MHz, $CDCl_3$) δ = 8.60 (dd, *J* = 4.9, 4.2 Hz, 2H), 8.19 (d, *J* = 7.8 Hz, 1H), 7.89 (td, *J* = 7.7, 1.6 Hz, 1H), 7.48 (ddd, *J* = 7.5, 4.8, 1.1 Hz, 1H), 7.40 (dt, *J* = 8.1, 4.1 Hz, 4H), 7.31 (td, *J* = 7.4, 1.3 Hz, 2H), 7.20 (t, *J* = 6.9 Hz, 4H), 7.06 (s, 1H), 4.93 (dd, *J* = 14.5, 9.1 Hz, 1H), 4.87 (s, 1H), 3.73 (s, 3H), 3.64 – 3.56 (m, 1H), 3.38 (dd, *J* = 14.6, 5.6 Hz, 1H), 3.16 (dd, *J* = 14.6, 9.8 Hz, 1H), 2.18 (q, *J* = 7.4 Hz, 2H), 1.36 (s, 9H), 0.93 (d, *J* = 7.0 Hz, 3H), 0.84 (t, *J* = 7.5 Hz, 3H), 0.58 (s, 3H). ^{13}C NMR (101 MHz, $CDCl_3$) δ = 172.3, 171.4, 164.1, 155.0, 149.3, 148.4, 141.3, 137.4, 135.8, 135.7, 134.6, 134.5, 129.6, 128.2, 128.2, 126.6, 122.5, 79.5, 52.6, 50.5, 50.2, 32.3, 28.3, 24.1, 14.8, -2.5. HR-MS (ESI) m/z calcd for $C_{35}H_{44}N_4NaO_6Si$ [M+Na$^+$]: 667.2922, found: 667.2926.

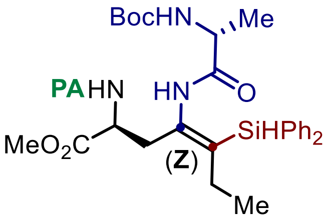

Methyl (*S*,*Z*)-4-{(*R*)-2-[(*tert*-butoxycarbonyl)amino]propanamido}-5-(diphenylsilyl)-2-(picolinamido)hept-4-enoate (37)

The general procedure **TP12** was followed using **1c** (39.0 mg, 0.15 mmol, 1.0 equiv.), **2e** (69.0 mg, 0.3 mmol, 2.0 equiv.), **3e** (1.0 mL, 0.3 mmol, 2.0 equiv.) and MeCN (0.5 mL) for 2 h. Purification by column chromatography (petroleum ether/EtOAc = 1.5:1) yielded **37** (7.0 mg, 7%) as a colorless oil. ^{1}H NMR (600 MHz, $CDCl_3$) δ = 8.67 (d, *J* =

8.8 Hz, 1H), 8.59 (d, J = 4.3 Hz, 1H), 8.25 (s, 1H), 8.20 (d, J = 7.8 Hz, 1H), 7.90 (td, J = 7.7, 1.6 Hz, 1H), 7.56 (d, J = 7.1 Hz, 2H), 7.48 (dd, J = 6.6, 4.8 Hz, 1H), 7.43 (d, J = 7.1 Hz, 2H), 7.34 (dt, J = 20.3, 7.4 Hz, 3H), 7.28 (t, J = 7.4 Hz, 2H), 7.20 (t, J = 7.6 Hz, 2H), 5.01 (td, J = 9.1, 5.5 Hz, 1H), 4.96 (s, 1H), 3.88 – 3.78 (m, 1H), 3.76 (s, 3H), 3.28 (d, J = 13.7 Hz, 1H), 3.16 (dd, J = 14.2, 9.5 Hz, 1H), 2.07 (dq, J = 14.9, 7.5 Hz, 1H), 1.99 (dd, J = 13.9, 7.3 Hz, 1H), 1.35 (s, 9H), 1.06 (d, J = 7.0 Hz, 3H), 0.68 (t, J = 7.5 Hz, 3H). ^{13}C NMR (101 MHz, $CDCl_3$) δ = 172.3, 172.1, 164.5, 155.6, 149.3, 148.5, 137.5, 135.4, 135.3, 134.7, 134.7, 130.0, 129.9, 128.0, 127.9, 126.6, 122.6, 80.3, 55.4, 52.7, 50.9, 33.3, 28.4, 28.3, 23.5, 14.6. HR-MS (ESI) m/z calcd for $C_{34}H_{43}N_4O_6Si$ [M+H$^+$]: 631.2946, found: 631.2941.

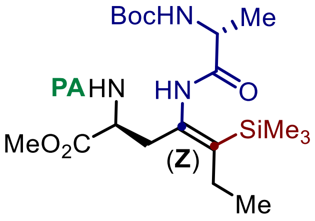

Methyl (*S*,*Z*)-4-{(*S*)-2-[(*tert*-butoxycarbonyl)amino]propanamido}-2-(picolinamido)-5-(trimethylsilyl)hept-4-enoate (38)

The general procedure **TP12** was followed using **1c** (39.0 mg, 0.15 mmol, 1.0 equiv.), **2e** (69.0 mg, 0.3 mmol, 2.0 equiv.), **3d** (1.0 mL, 0.3 mmol, 2.0 equiv.) and MeCN (0.5 mL) for 2 h. Purification by column chromatography (petroleum ether/EtOAc = 2.5:1) yielded **38** (19.3 mg, 25%) as a colorless oil. ^{1}H NMR (600 MHz, $CDCl_3$) δ = 8.59 (t, J = 7.2 Hz, 2H), 8.13 (d, J = 7.8 Hz, 1H), 7.90 – 7.81 (m, 2H), 7.47 – 7.40 (m, 1H), 5.39 (s, 1H), 4.99 (s, 1H), 4.39 – 4.28 (m, 1H), 3.78 (s, 3H), 3.09 (dd, J = 14.3, 4.3 Hz, 1H), 2.92 – 2.81 (m, 1H), 2.05 (dt, J = 7.8, 5.2 Hz, 2H), 1.50 – 1.44 (m, 12H), 0.79 (t, J = 7.5 Hz, 3H), 0.00 (s, 9H). ^{13}C NMR (151 MHz, $CDCl_3$) δ = 172.5, 172.5, 164.3, 155.3, 148.4, 142.6, 138.1, 137.4, 126.6, 122.4, 79.9, 52.7, 50.6, 50.3, 33.5, 28.4, 24.0, 18.6, 14.7, -0.3. HR-MS (ESI) m/z calcd for $C_{25}H_{40}N_4NaO_6Si$ [M+Na$^+$]: 543.2609, found: 543.2603.

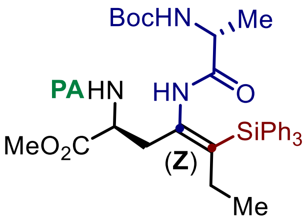

Methyl (*S*,*Z*)-4-{(*S*)-2-[(*tert*-butoxycarbonyl)amino]propanamido}-2-(picolinamido)-5-(triphenylsilyl)hept-4-enoate (39)

The general procedure **TP12** was followed using **1c** (39.0 mg, 0.15 mmol, 1.0 equiv.), **2e** (69.0 mg, 0.3 mmol, 2.0 equiv.), **3c** (1.0 mL, 0.3 mmol, 2.0 equiv.) and MeCN (0.5 mL) for 2 h. Purification by column chromatography (petroleum ether/EtOAc = 2.5:1) yielded **39** (25.0 mg, 24%) as a white solid. ^{1}H NMR (400 MHz, $CDCl_3$) δ = 8.63 (t, J = 5.7 Hz, 2H), 8.23 (d, J = 7.8 Hz, 1H), 7.91 (td, J = 7.7, 1.6 Hz, 1H), 7.53 – 7.48 (m, 7H), 7.37 (t, J = 7.4 Hz, 3H), 7.25 (dd, J = 10.2, 4.6 Hz, 6H), 6.93 (s, 1H), 4.93 (td, J = 9.1, 6.0 Hz, 1H), 4.63 (d, J = 7.8 Hz, 1H), 3.76 (s, 3H), 3.56 (dd, J = 14.8, 5.9 Hz, 1H), 3.38 – 3.26 (m, 2H), 2.23 – 2.08 (m, 2H), 1.31 (s, 9H), 0.78 (t, J = 7.4 Hz, 3H), 0.70 (d, J = 7.0 Hz, 3H). ^{13}C NMR (101 MHz, $CDCl_3$) δ = 172.3, 171.2, 164.2, 154.8, 149.4, 148.5, 142.5, 137.4, 135.8, 133.5, 129.9, 128.3, 126.6, 122.6, 79.4, 52.6, 50.7, 50.1, 32.0, 28.4, 28.3, 24.2, 14.7. HR-MS (ESI) m/z calcd for $C_{40}H_{46}N_4NaO_6Si$ [M+Na$^+$]: 729.3079, found: 729.3072.

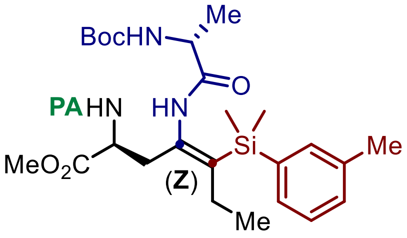

Methyl (*S*,*Z*)-4-{(*R*)-2-[(*tert*-butoxycarbonyl)amino]propanamido}-5-[dimethyl(*m*-tolyl)silyl]-2-(picolinamido)hept-4-enoate (40)

The general procedure **TP11** was followed using **1c** (26.0 mg, 0.1 mmol, 1.0 equiv.), **2e** (46.0 mg, 0.3 mmol, 2.0 equiv.), **3g** (0.5 mL, 0.2 mmol, 2.0 equiv.) and THF (0.5 mL), at 40 °C for 2 h. Purification by column chromatography (petroleum ether/EtOAc = 2:1) yielded **40** (33.0 mg, 55%) as a colorless oil. ^{1}H NMR (400 MHz, $CDCl_3$) δ = 8.56 (dd, J = 13.8, 6.7 Hz, 2H), 8.16 (d, J = 7.8 Hz, 1H), 7.86 (td, J = 7.7, 1.6 Hz, 1H),

7.46 (ddd, J = 7.5, 4.8, 1.0 Hz, 1H), 7.26 (s, 1H), 7.19 (d, J = 7.3 Hz, 2H), 7.14 – 7.00 (m, 2H), 5.18 – 4.77 (m, 2H), 4.03 – 3.82 (m, 1H), 3.77 (d, J = 24.9 Hz, 3H), 3.24 (dd, J = 14.4, 5.5 Hz, 1H), 3.04 (dd, J = 14.3, 9.5 Hz, 1H), 2.28 (s, 3H), 2.18 (q, J = 7.5 Hz, 2H), 1.42 (s, 9H), 1.11 (d, J = 7.0 Hz, 3H), 0.92 (t, J = 7.5 Hz, 3H), 0.28 (d, J = 4.5 Hz, 6H). ^{13}C NMR (101 MHz, $CDCl_3$) δ = 172.4, 171.6, 164.9, 155.2, 149.3, 148.5, 139.8, 138.4, 137.8, 137.4, 134.2, 130.5, 130.3, 128.2, 126.6, 122.5, 79.7, 52.6, 50.6, 38.9, 32.7, 28.4, 24.1, 22.8, 21.5, 15.0, -1.5, -1.7. HR-MS (ESI) m/z calcd for $C_{31}H_{44}N_4NaO_6Si$ [M+Na$^+$]: 619.2922, found: 619.2928.

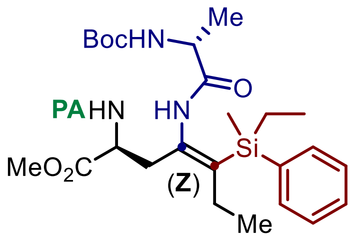

(*dr* = 1:1)

Methyl (2*S*,*Z*)-4-{(*R*)-2-[(*tert*-butoxycarbonyl)amino]propanamido}-5-[ethyl(methyl)(phenyl)silyl]-2-(picolinamido)hept-4-enoate (41)

The general procedure **TP11** was followed using **1c** (26.0 mg, 0.1 mmol, 1.0 equiv.), **2e** (46.0 mg, 0.3 mmol, 2.0 equiv.), **3f** (0.5 mL, 0.2 mmol, 2.02 equiv.) and THF (0.5 mL) for 2 h. Purification by column chromatography (petroleum ether/EtOAc = 2:1) yielded **41** (35.6 mg, 61%) as a colorless oil. ^{1}H NMR (400 MHz, $CDCl_3$) δ = 8.58 (d, J = 4.7 Hz, 1H), 8.56 – 8.44 (m, 1H), 8.16 (d, J = 7.8 Hz, 1H), 7.87 (td, J = 7.7, 1.5 Hz, 1H), 7.46 (dd, J = 7.1, 5.2 Hz, 1H), 7.38 (d, J = 7.1 Hz, 2H), 7.26 (dd, J = 10.4, 4.3 Hz, 1H), 7.22 – 7.01 (m, 3H), 5.09 (dd, J = 107.9, 64.6 Hz, 2H), 4.04 – 3.79 (m, 1H), 3.74 (s, 3H), 3.29 (dd, J = 14.5, 5.3 Hz, 1H), 3.05 (ddd, J = 14.4, 10.0, 2.1 Hz, 1H), 2.39 – 2.08 (m, 2H), 1.42 (d, J = 5.4 Hz, 9H), 1.12 (dd, J = 6.9, 4.6 Hz, 3H), 1.01 – 0.91 (m, 3H), 0.90 – 0.73 (m, 5H), 0.28 (d, J = 2.4 Hz, 3H). ^{13}C NMR (101 MHz, $CDCl_3$) δ = 172.4, 171.6, 164.1, 155.2, 149.3, 148.4, 140.3, 140.2, 137.6, 137.4, 133.8, 133.8, 129.3, 128.2, 126.6, 122.5, 79.68, 52.6, 50.4, 32.5, 28.4, 28.4, 24.0, 23.9, 18.2, 15.1, 15.0, 7.5, 7.5, 6.1, 6.1, -4.1. HR-MS (ESI) m/z calcd for $C_{31}H_{44}N_4NaO_6Si$ [M+Na$^+$]: 619.2922, found: 619.2927.

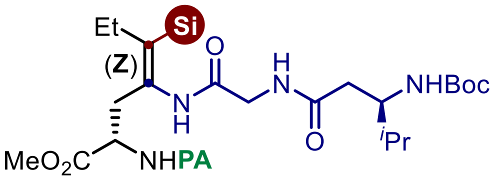

Methyl (6*R*,15*S*,*Z*)-13-{1-[dimethyl(phenyl)silyl]propylidene}-6-isopropyl-2,2-dimethyl-4,8,11-trioxo-15-(picolinamido)-3-oxa-5,9,12-triazahexadecan-16-oate (42)

The general procedure **TP11** was followed using **1c** (26.0 mg, 0.1 mmol, 1.0 equiv.), **2v** (65.8 mg, 0.2 mmol, 2.0 equiv.), **3a** (0.5 mL, 0.2 mmol, 2.0 equiv.) and THF (0.5 mL) for 2 h. Purification by column chromatography (petroleum ether/EtOAc = 1:1) yielded **42** (42.2 mg, 62%) as a colorless oil. ^{1}H NMR (400 MHz, $CDCl_3$) δ = 8.61 (s, 2H), 8.07 (d, J = 7.7 Hz, 1H), 7.90 (t, J = 7.7 Hz, 1H), 7.51 – 7.46 (m, 1H), 7.34 (d, J = 7.2 Hz, 2H), 7.28 (d, J = 7.1 Hz, 1H), 7.19 (dd, J = 13.8, 6.4 Hz, 3H), 6.34 (s, 1H), 5.27 (d, J = 8.7 Hz, 1H), 4.85 (t, J = 7.6 Hz, 1H), 3.75 (s, 3H), 3.61 (dd, J = 17.9, 12.2 Hz, 3H), 3.10 (d, J = 13.7 Hz, 1H), 2.96 – 2.85 (m, 1H), 2.30 (q, J = 15.6 Hz, 2H), 2.17 (q, J = 6.9 Hz, 2H), 1.83 (dd, J = 12.4, 6.2 Hz, 1H), 1.40 (s, 9H), 0.91 (d, J = 7.0 Hz, 9H), 0.29 (s, 3H), 0.22 (s, 3H). ^{13}C NMR (101 MHz, $CDCl_3$) δ = 172.3, 171.6, 168.1, 164.1, 156.1, 149.0, 148.5, 140.2, 139.2, 139.1, 137.4, 133.5, 129.1, 128.1, 126.7, 122.3, 79.2, 53.2, 52.7, 50.1, 42.9, 38.3, 33.6, 31.9, 28.4, 24.0, 19.4, 18.8, 14.8, -1.7, -2.2. HR-MS (ESI) m/z calcd for $C_{35}H_{51}N_5NaO_7Si$ [M+Na$^+$]: 704.3450, found: 704.3460.

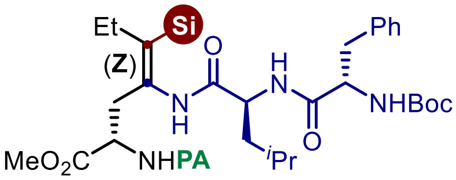

Methyl (6*S*,9*S*,14*S*,*Z*)-6-benzyl-12-{1-[dimethyl(phenyl)silyl]propylidene}-9-isobutyl-2,2-dimethyl-4,7,10-trioxo-14-(picolinamido)-3-oxa-5,8,11-triazapentadecan-15-oate (43)

The general procedure **TP11** was followed using **1c** (26.0 mg, 0.1 mmol, 1.0 equiv.), **2w** (83.9 mg, 0.2 mmol, 2.0 equiv.), **3a** (0.5 mL, 0.2 mmol, 2.0 equiv.) and THF (0.5 mL) for 2 h. Purification by column chromatography (petroleum ether/EtOAc = 1:1)

yielded **43** (48.0 mg, 62%) as a colorless oil. ^{1}H NMR (600 MHz, $CDCl_3$) δ = 8.59 (t, J = 5.8 Hz, 2H), 8.12 (d, J = 7.7 Hz, 1H), 7.85 (t, J = 7.4 Hz, 1H), 7.45 (dd, J = 7.3, 5.0 Hz, 1H), 7.38 (d, J = 7.1 Hz, 3H), 7.32 (t, J = 7.3 Hz, 1H), 7.25 (dd, J = 12.1, 5.0 Hz, 4H), 7.20 (t, J = 5.6 Hz, 3H), 6.40 (d, J = 5.4 Hz, 1H), 4.90 (s, 1H), 4.85 (td, J = 10.7, 4.4 Hz, 1H), 4.42 (s, 1H), 4.18 (dd, J = 12.7, 5.5 Hz, 1H), 3.76 (s, 3H), 3.18 – 3.08 (m, 2H), 3.00 (dd, J = 14.2, 8.4 Hz, 1H), 2.88 (dd, J = 13.9, 11.1 Hz, 1H), 2.19 – 2.07 (m, 2H), 1.43 (dd, J = 12.0, 3.4 Hz, 3H), 1.39 (s, 9H), 0.86 (t, J = 7.4 Hz, 3H), 0.82 (d, J = 6.1 Hz, 3H), 0.80 (d, J = 6.0 Hz, 3H), 0.28 (s, 3H), 0.26 (s, 3H). ^{13}C NMR (151 MHz, $CDCl_3$) δ = 172.5, 171.6, 171.1, 164.0, 155.6, 149.2, 148.5, 140.1, 139.7, 138.7, 137.3, 133.6, 129.4, 129.2, 128.5, 128.2, 126.7, 126.5, 122.4, 80.1, 55.5, 52.7, 52.1, 50.3, 40.9, 37.0, 33.7, 28.3, 24.6, 24.1, 23.2, 21.4, 14.8, -1.4, -1.8. HR-MS (ESI) m/z calcd for $C_{42}H_{57}N_5NaO_7Si$ [M+Na$^+$]: 794.3919, found: 794.3915.

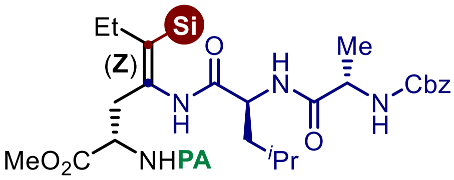

Methyl (5*S*,8*S*,13*S*,*Z*)-11-{1-[dimethyl(phenyl)silyl]propylidene}-8-isobutyl-5-methyl-3,6,9-trioxo-1-phenyl-13-(picolinamido)-2-oxa-4,7,10-triazatetradecan-14-oate (44)

The general procedure **TP11** was followed using **1c** (26.0 mg, 0.1 mmol, 1.0 equiv.), **2x** (75.4 mg, 0.2 mmol, 2.0 equiv.), **3a** (0.5 mL, 0.2 mmol, 2.0 equiv.) and THF (0.5 mL) for 2 h. Purification by column chromatography (petroleum ether/EtOAc = 1.5:1) yielded **44** (46.4 mg, 64%) as a colorless oil. ^{1}H NMR (400 MHz, $CDCl_3$) δ = 8.58 (t, J = 6.2 Hz, 2H), 8.10 (d, J = 7.8 Hz, 1H), 7.85 (t, J = 7.7 Hz, 1H), 7.48 – 7.43 (m, 1H), 7.35 (t, J = 8.9 Hz, 8H), 7.29 – 7.20 (m, 3H), 6.21 (d, J = 7.2 Hz, 1H), 5.38 (d, J = 7.0 Hz, 1H), 5.10 (s, 2H), 4.82 (td, J = 9.9, 4.6 Hz, 1H), 4.20 (dt, J = 14.3, 6.9 Hz, 2H), 3.75 (s, 3H), 3.68 (t, J = 11.3 Hz, 1H), 3.11 (dd, J = 14.1, 4.5 Hz, 1H), 2.90 (dd, J = 14.0, 10.8 Hz, 1H), 2.22 – 2.09 (m, 2H), 1.48 (dd, J = 14.0, 7.3 Hz, 2H), 1.35 (d, J = 7.0 Hz, 3H), 0.88 – 0.84 (m, 6H), 0.81 (d, J = 5.7 Hz, 3H), 0.27 (d, J = 14.5 Hz, 6H). ^{13}C NMR (101 MHz, $CDCl_3$) δ = 172.5, 172.2, 171.1, 164.0, 149.2, 148.5, 140.2, 139.6,

138.8, 137.4, 136.3, 133.6, 129.2, 128.6, 128.3, 128.2, 128.1, 126.5, 122.4, 67.0, 52.7, 52.1, 50.6, 50.3, 41.0, 33.7, 24.9, 24.1, 23.2, 21.6, 14.9, -1.4, -2.0. HR-MS (ESI) m/z calcd for $C_{39}H_{51}N_5NaO_7Si$ [M+Na$^+$]: 752.3450, found: 752.3457.

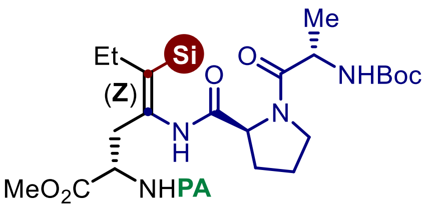

Methyl (*S*,*Z*)-4-{(*S*)-1-[(*tert*-butoxycarbonyl)-L-alanyl]pyrrolidine-2-carboxamido}-5-[dimethyl(phenyl)silyl]-2-(picolinamido)hept-4-enoate (45)

The general procedure **TP11** was followed using **1c** (26.0 mg, 0.1 mmol, 1.0 equiv.), **2u** (65.4 mg, 0.2 mmol, 2.0 equiv.), **3a** (0.5 mL, 0.2 mmol, 2.0 equiv.) and THF (0.5 mL) for 2 h. Purification by column chromatography (petroleum ether/EtOAc = 1.5:1 to 1:1) yielded **45** (46.0 mg, 68%) as a colorless oil. ^{1}H NMR (400 MHz, $CDCl_3$) δ = 8.63 – 8.52 (m, 2H), 8.13 (d, J = 7.6 Hz, 1H), 7.88 (t, J = 7.6 Hz, 1H), 7.50 – 7.39 (m, 4H), 7.36 – 7.25 (m, 2H), 7.22 (t, J = 7.0 Hz, 2H), 5.43 (d, J = 7.9 Hz, 1H), 4.84 (dd, J = 14.8, 7.2 Hz, 1H), 4.52 – 4.42 (m, 1H), 4.18 (s, 1H), 3.76 (s, 3H), 3.58 (s, 1H), 3.43 (s, 1H), 3.19 (dd, J = 14.1, 4.8 Hz, 1H), 3.01 – 2.91 (m, 1H), 2.24 – 2.13 (m, 2H), 1.93 – 1.83 (m, 3H), 1.44 (s, 9H), 1.34 (d, J = 6.7 Hz, 3H), 0.90 (t, J = 6.5 Hz, 3H), 0.40 (s, 3H), 0.31 (s, 3H). ^{13}C NMR (101 MHz, $CDCl_3$) δ = 172.5, 172.4, 170.4, 163.8, 155.1, 149.3, 148.3, 140.0, 138.7, 138.5, 137.2, 133.6, 128.9, 127.8, 126.3, 122.2, 79.4, 60.4, 52.4, 50.3, 47.8, 47.1, 33.1, 28.3, 28.1, 24.8, 24.0, 18.5, 14.8, -1.3, -1.9. HR-MS (ESI) m/z calcd for $C_{35}H_{49}N_5NaO_7Si$ [M+Na$^+$]: 702.3293, found: 702.3298.

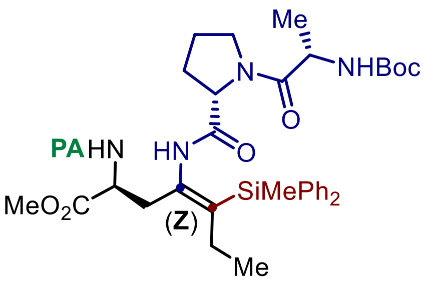

Methyl (*S*,*Z*)-4-{(*S*)-1-[(*tert*-butoxycarbonyl)-L-alanyl]pyrrolidine-2-carboxamido}-5-(methyldiphenylsilyl)-2-(picolinamido)hept-4-enoate (46)

The general procedure **TP11** was followed using **1c** (39.0 mg, 0.15 mmol, 1.0 equiv.), **2u** (98.0 mg, 0.3 mmol, 2.0 equiv.), **3b** (1.0 mL, 0.3 mmol, 2.0 equiv.) and THF (0.5 mL) for 2 h. Purification by column chromatography (petroleum ether/EtOAc = 1:1) yielded **46** (80.1 mg, 72%) as a colorless oil. ^{1}H NMR (600 MHz, $CDCl_3$) δ = 8.62 – 8.52 (m, 2H), 8.17 (d, J = 7.8 Hz, 1H), 7.88 (t, J = 7.7 Hz, 1H), 7.46 (dd, J = 7.3, 5.0 Hz, 1H), 7.41 (q, J = 9.2 Hz, 4H), 7.31 (dd, J = 12.2, 7.2 Hz, 2H), 7.27 (s, 1H), 7.23 (dd, J = 11.8, 7.3 Hz, 4H), 5.36 (d, J = 8.3 Hz, 1H), 4.85 (dd, J = 14.8, 9.2 Hz, 1H), 4.47 – 4.33 (m, 1H), 3.92 (d, J = 7.6 Hz, 1H), 3.76 (s, 3H), 3.46 (dd, J = 15.9, 6.9 Hz, 1H), 3.32 (dd, J = 14.2, 5.6 Hz, 1H), 3.25 (dd, J = 9.6, 5.9 Hz, 1H), 3.04 (dd, J = 14.2, 9.9 Hz, 1H), 2.17 – 2.05 (m, 2H), 1.75 – 1.65 (m, 3H), 1.59 (dd, J = 11.3, 5.0 Hz, 1H), 1.41 (s, 9H), 1.27 (d, J = 6.8 Hz, 3H), 0.75 (t, J = 7.4 Hz, 3H), 0.64 (s, 3H). ^{13}C NMR (151 MHz, $CDCl_3$) δ = 172.5, 170.5, 164.0, 155.2, 149.7, 148.4, 141.8, 137.3, 136.4, 136.3, 136.1, 134.9, 134.8, 129.4, 129.3, 128.1, 127.9, 126.4, 122.4, 79.5, 60.6, 52.6, 50.6, 47.9, 47.1, 33.0, 28.4, 28.1, 24.8, 24.3, 18.5, 14.7, -2.6. HR-MS (ESI) m/z calcd for $C_{40}H_{51}N_5NaO_7Si$ [M+Na$^+$]: 764.3450, found: 764.3459.

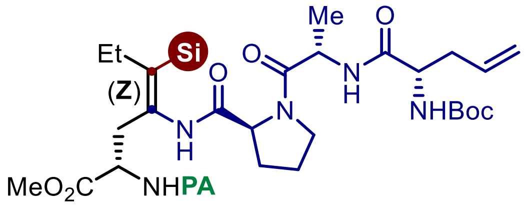

Methyl (*S*,*Z*)-4-{{{(*S*)-1-{{{(*S*)-2-[(*tert*-butoxycarbonyl)amino]pent-4-enoyl}-L-alanyl}}pyrrolidine-2-carboxamido}}}-5-[dimethyl(phenyl)silyl]-2-(picolinamido)hept-4-enoate (47)

The general procedure **TP11** was followed using **1c** (26.0 mg, 0.1 mmol, 1.0 equiv.), **2y** (84.8 mg, 0.2 mmol, 2.0 equiv.), **3a** (0.5 mL, 0.2 mmol, 2.0 equiv.) and THF (0.5 mL) for 2 h. Purification by column chromatography (petroleum ether/EtOAc = 1:3) yielded **47** (31.0 mg, 40%) as a colorless oil. ^{1}H NMR (400 MHz, $CDCl_3$) δ = 8.57 (d, J = 4.4 Hz, 1H), 8.54 (d, J = 8.7 Hz, 1H), 8.12 (d, J = 7.8 Hz, 1H), 7.88 (t, J = 7.1 Hz, 1H), 7.46 (dd, J = 6.9, 5.2 Hz, 1H), 7.42 (d, J = 6.9 Hz, 2H), 7.35 (s, 1H), 7.32 – 7.26 (m, 2H), 7.19 (t, J = 7.3 Hz, 2H), 6.98 (d, J = 7.3 Hz, 1H), 5.78 – 5.67 (m, 1H), 5.12

(dd, J = 13.2, 7.3 Hz, 2H), 5.02 (s, 1H), 4.90 – 4.81 (m, 1H), 4.72 – 4.65 (m, 1H), 4.13 (d, J = 26.1 Hz, 2H), 3.75 (s, 3H), 3.54 (d, J = 8.4 Hz, 1H), 3.40 (s, 1H), 3.19 (dd, J = 14.2, 5.6 Hz, 1H), 2.94 (dd, J = 14.1, 10.1 Hz, 1H), 2.49 (dd, J = 13.1, 6.5 Hz, 2H), 2.17 (dt, J = 13.9, 6.3 Hz, 2H), 1.86 (d, J = 4.4 Hz, 3H), 1.42 (s, 9H), 1.32 (d, J = 6.6 Hz, 3H), 0.91 (t, J = 7.5 Hz, 3H), 0.38 (s, 3H), 0.29 (s, 3H). ^{13}C NMR (101 MHz, $CDCl_3$) δ = 172.6, 171.6, 170.6, 170.5, 164.0, 155.4, 149.4, 148.4, 140.1, 138.9, 138.7, 137.4, 133.7, 133.1, 129.1, 128.0, 126.5, 122.4, 119.1, 80.1, 60.7, 53.9, 52.6, 50.4, 47.2, 47.0, 37.0, 34.7, 33.1, 28.4, 25.0, 24.1, 18.1, 15.0, -1.2, -1.9. HR-MS (ESI) m/z calcd for $C_{40}H_{56}N_6NaO_8Si$ [M+Na$^+$]: 799.3821, found: 799.3827.

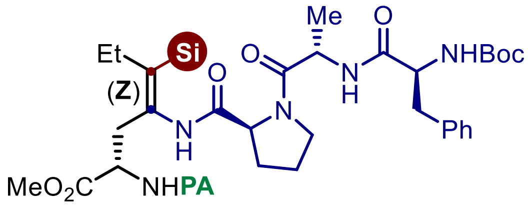

Methyl (*S*,*Z*)-4-{(*S*)-1-[(*tert*-butoxycarbonyl)-L-phenylalanyl-L-alanyl]pyrrolidine-2-carboxamido}-5-[dimethyl(phenyl)silyl]-2-(picolinamido)hept-4-enoate (48)

The general procedure **TP11** was followed using **1c** (39.0 mg, 0.15 mmol, 1.0 equiv.), **2aa** (142.0 mg, 0.3 mmol, 2.0 equiv.), **3a** (1.0 mL, 0.3 mmol, 2.0 equiv.) and THF (0.5 mL) for 2 h. Purification by column chromatography (petroleum ether/EtOAc = 1:2) yielded **48** (50.1 mg, 41%) as a colorless oil. ^{1}H NMR (400 MHz, $CDCl_3$) δ = 8.64 – 8.48 (m, 2H), 8.11 (d, J = 7.8 Hz, 1H), 7.87 (t, J = 7.7 Hz, 1H), 7.49 – 7.40 (m, 3H), 7.37 (s, 1H), 7.27 (dd, J = 11.2, 6.0 Hz, 3H), 7.19 (t, J = 7.8 Hz, 5H), 6.90 (d, J = 6.7 Hz, 1H), 5.01 (d, J = 7.4 Hz, 1H), 4.85 (dd, J = 15.2, 9.0 Hz, 1H), 4.71 – 4.62 (m, 1H), 4.40 (d, J = 5.0 Hz, 1H), 4.09 (s, 1H), 3.72 (s, 3H), 3.49 (s, 1H), 3.38 (s, 1H), 3.18 (dd, J = 14.1, 5.5 Hz, 1H), 3.09 (dd, J = 13.3, 5.8 Hz, 2H), 2.98 – 2.90 (m, 1H), 2.34 – 2.06 (m, 3H), 1.84 (d, J = 5.6 Hz, 3H), 1.37 (s, 9H), 1.28 (d, J = 6.1 Hz, 3H), 0.90 (t, J = 7.3 Hz, 3H), 0.38 (s, 3H), 0.28 (s, 3H). ^{13}C NMR (101 MHz, $CDCl_3$) δ = 172.5, 171.5, 170.4, 163.9, 155.2, 149.3, 148.3, 140.0, 138.8, 138.5, 137.3, 136.6, 133.7, 129.4, 129.0, 128.5, 127.9, 126.8, 126.4, 122.3, 80.0, 60.6, 55.5, 52.5, 50.2, 47.1, 46.9, 38.3, 33.0, 28.2, 24.9,

24.0, 17.9, 14.9, -1.2, -1.9. HR-MS (ESI) m/z calcd for $C_{44}H_{58}N_6NaO_8Si$ [M+Na$^+$]: 849.3978, found: 849.3969.

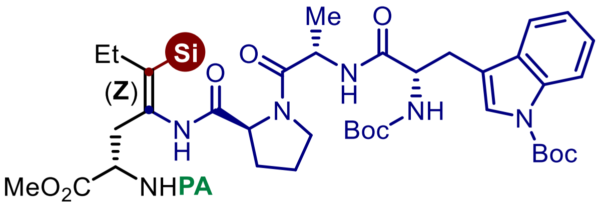

***tert*-Butyl 3-{{{{{{(*S*)-2-[(*tert*-butoxycarbonyl)amino]-3-{{{{{{{{{(*S*)-1-{{{(*S*)-2-{{{(*S*,*Z*)-3-[dimethyl(phenyl)silyl]-7-methoxy-7-oxo-6-(picolinamido)hept-3-en-4-yl}carbamoyl}}pyrrolidin-1-yl}}}-1-oxopropan-2-yl}}}}amino}}}}}-3-oxopropyl}}}}}}-1*H*-indole-1-carboxylate (49)**

The general procedure **TP11** was followed using **1c** (39.0 mg, 0.15 mmol, 1.0 equiv.), **2z** (184.0 mg, 0.3 mmol, 2.0 equiv.), **3a** (1.0 mL, 0.3 mmol, 2.0 equiv.) and THF (0.5 mL) for 2 h. Purification by column chromatography (petroleum ether/EtOAc = 1:1) yielded **49** (64.7 mg, 45%) as a colorless oil. ^{1}H NMR (400 MHz, $CDCl_3$) δ = 8.55 (d, J = 4.5 Hz, 1H), 8.52 (d, J = 8.8 Hz, 1H), 8.08 (d, J = 7.8 Hz, 2H), 7.91 – 7.82 (m, 1H), 7.53 (d, J = 7.8 Hz, 1H), 7.45 (s, 2H), 7.42 – 7.39 (m, 2H), 7.32 (s, 1H), 7.27 (dd, J = 9.0, 5.5 Hz, 2H), 7.19 (t, J = 7.3 Hz, 3H), 6.89 (d, J = 7.4 Hz, 1H), 5.14 (d, J = 7.2 Hz, 1H), 4.81 (td, J = 9.4, 6.0 Hz, 1H), 4.57 – 4.49 (m, 1H), 4.44 (d, J = 5.9 Hz, 1H), 4.06 (s, 1H), 3.70 (s, 3H), 3.41 – 3.28 (m, 2H), 3.15 (dd, J = 14.3, 5.8 Hz, 3H), 2.93 (dd, J = 14.1, 10.0 Hz, 1H), 2.22 – 2.10 (m, 2H), 1.84 (s, 4H), 1.65 (s, 9H), 1.40 (s, 9H), 1.24 (d, J = 6.7 Hz, 3H), 0.89 (t, J = 7.4 Hz, 3H), 0.36 (s, 3H), 0.27 (s, 3H). ^{13}C NMR (101 MHz, $CDCl_3$) δ = 172.5, 171.4, 170.4, 163.9, 155.3, 149.6, 149.4, 148.3, 140.1, 138.8, 138.5, 137.3, 135.5, 133.7, 130.7, 129.0, 128.0, 126.4, 124.4, 124.3, 122.4, 122.3, 119.1, 115.5, 115.2, 83.6, 80.1, 60.6, 54.8, 52.5, 50.3, 47.1, 47.0, 33.1, 28.3, 28.2, 28.2, 24.9, 24.0, 18.0, 14.9, -1.2, -1.9. HR-MS (ESI) m/z calcd for $C_{51}H_{67}N_7NaO_{10}Si$ [M+Na$^+$] 988.4611, found: 988.4622.

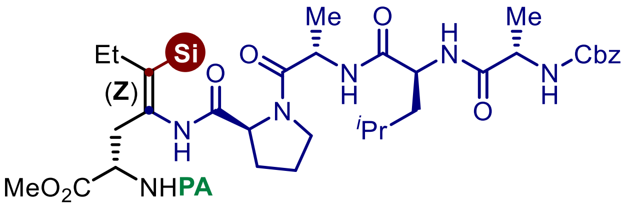

Methyl (*S*,*Z*)-4-{{(*S*)-1-{[(benzyloxy)carbonyl]-L-alanyl-L-leucyl-L-alanyl}pyrrolidine-2-carboxamido}}-5-[dimethyl(phenyl)silyl]-2-(picolinamido)hept-4-enoate (50)

The general procedure **TP11** was followed using **1c** (26.0 mg, 0.1 mmol, 1.0 equiv.), **2ab** (109 mg, 0.2 mmol, 2.0 equiv.), **3a** (0.5 mL, 0.2 mmol, 2.0 equiv.) and THF (0.5 mL) for 2 h. Purification by column chromatography (petroleum ether/EtOAc = 1:8) yielded **50** (34.4 mg, 38%) as a colorless oil. ^{1}H NMR (400 MHz, $CDCl_3$) δ = 8.59 – 8.51 (m, 2H), 8.09 (d, J = 7.8 Hz, 1H), 7.86 (t, J = 7.7 Hz, 1H), 7.47 – 7.40 (m, 3H), 7.33 (s, 6H), 7.25 – 7.15 (m, 3H), 7.09 (d, J = 7.3 Hz, 1H), 6.68 (d, J = 7.7 Hz, 1H), 5.43 (d, J = 6.9 Hz, 1H), 5.09 (s, 2H), 4.93 (dd, J = 15.3, 8.7 Hz, 1H), 4.63 – 4.55 (m, 1H), 4.50 – 4.43 (m, 1H), 4.30 – 4.22 (m, 1H), 4.09 (s, 1H), 3.74 (s, 3H), 3.55 – 3.46 (m, 1H), 3.35 – 3.26 (m, 1H), 3.19 (dd, J = 14.2, 6.2 Hz, 1H), 2.93 (dd, J = 14.0, 10.0 Hz, 1H), 2.19 (dd, J = 13.9, 6.8 Hz, 2H), 1.85 (dd, J = 17.4, 6.1 Hz, 4H), 1.67 – 1.60 (m, 2H), 1.37 (d, J = 6.9 Hz, 3H), 1.21 (d, J = 6.5 Hz, 4H), 0.97 – 0.88 (m, 9H), 0.36 (s, 3H), 0.28 (s, 3H). ^{13}C NMR (101 MHz, $CDCl_3$) δ = 172.5, 172.3, 171.6, 171.1, 170.5, 164.1, 156.0, 149.4, 148.4, 140.1, 139.0, 138.9, 137.4, 136.3, 133.6, 129.1, 128.6, 128.2, 128.1, 128.1, 126.5, 122.3, 67.0, 61.0, 52.6, 52.0, 50.6, 50.3, 47.2, 47.0, 41.6, 32.8, 29.8, 29.4, 28.3, 25.2, 24.8, 24.1, 23.1, 21.8, 18.0, 15.0, -1.2, -1.8. HR-MS (ESI) m/z calcd for $C_{47}H_{63}N_7NaO_9Si$ [M+Na$^+$]: 920.4349, found: 920.4342.

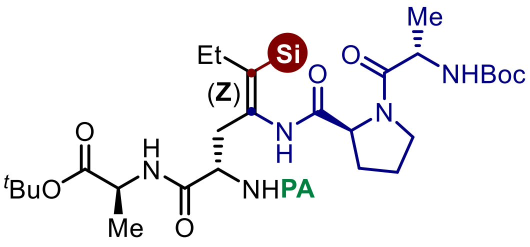

tert-Butyl {{(S,Z)-4-{(S)-1-[(tert-butoxycarbonyl)-L-alanyl]pyrrolidine-2-carboxamido}-5-[dimethyl(phenyl)silyl]-2-(picolinamido)hept-4-enoyl}}-L-alaninate (51)

The general procedure **TP11** was followed using **1q** (56.0 mg, 0.15 mmol, 1.0 equiv.), **2u** (98.0 mg, 0.3 mmol, 2.0 equiv.), **3a** (1.0 mL, 0.3 mmol, 2.0 equiv.) and THF (0.5 mL) for 2 h. Purification by column chromatography (petroleum ether/EtOAc = 1:1) yielded **51** (35.6 mg, 30%) as a colorless oil. ^{1}H NMR (600 MHz, $CDCl_3$) δ = 8.65 (d, J = 9.1 Hz, 1H), 8.55 (d, J = 4.6 Hz, 1H), 8.13 (d, J = 7.8 Hz, 1H), 7.96 (d, J = 7.4 Hz, 1H), 7.83 (t, J = 7.2 Hz, 1H), 7.64 (s, 1H), 7.45 (d, J = 6.0 Hz, 2H), 7.41 (dd, J = 7.3, 5.0 Hz, 1H), 7.29 (dd, J = 12.4, 5.7 Hz, 3H), 5.29 (d, J = 8.5 Hz, 1H), 4.79 – 4.68 (m, 1H), 4.51 – 4.43 (m, 2H), 4.22 (dd, J = 8.5, 5.3 Hz, 1H), 3.62 (dd, J = 16.3, 6.9 Hz, 1H), 3.41 (dd, J = 15.6, 6.9 Hz, 1H), 3.06 – 2.86 (m, 2H), 2.13 (q, J = 7.3 Hz, 2H), 1.99 – 1.85 (m, 2H), 1.75 (dt, J = 8.9, 6.1 Hz, 2H), 1.46 (s, 9H), 1.43 (s, 3H), 1.40 (s, 9H), 1.35 (d, J = 6.9 Hz, 3H), 0.81 (t, J = 7.5 Hz, 3H), 0.33 (s, 3H), 0.25 (s, 3H). ^{13}C NMR (151 MHz, $CDCl_3$) δ = 173.1, 172.1, 171.3, 170.8, 163.7, 155.3, 149.7, 148.4, 140.7, 139.2, 139.1, 137.1, 133.8, 128.8, 127.9, 126.2, 122.2, 81.9, 79.6, 61.9, 50.7, 48.8, 48.1, 47.4, 34.9, 28.5, 28.4, 28.0, 25.0, 24.2, 18.0, 17.9, 14.6, -0.8, -1.2. HR-MS (ESI) m/z calcd for $C_{41}H_{60}N_6NaO_8Si$ [M+Na$^+$]: 815.4134, found: 815.4140.

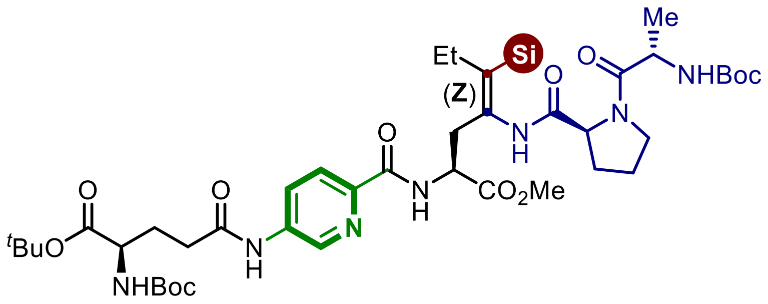

Methyl (S,Z)-2-{{5-{(S)-5-(tert-butoxy)-4-[(tert-butoxycarbonyl)amino]-5-oxopentanamido}picolinamido}}-4-{(S)-1-[(tert-butoxycarbonyl)-L-alanyl]pyrrolidine-2-carboxamido}-5-[dimethyl(phenyl)silyl]hept-4-enoate (52)

The general procedure **TP11** was followed using **1n** (57.4 mg, 0.1 mmol, 1.0 equiv.), **2u** (65.4 mg, 0.2 mmol, 2.0 equiv.), **3a** (0.5 mL, 0.2 mmol, 2.0 equiv.) and THF (0.5 mL) for 2 h. Purification by column chromatography (petroleum ether/EtOAc = 1:2)

yielded **52** (51.0 mg, 52%) as a colorless oil. ^{1}H NMR (600 MHz, $CDCl_3$) δ = 9.79 (s, 1H), 8.70 (s, 1H), 8.38 (d, J = 8.7 Hz, 1H), 8.33 (d, J = 7.9 Hz, 1H), 8.04 (d, J = 8.4 Hz, 1H), 7.47 (s, 1H), 7.41 (d, J = 7.1 Hz, 2H), 7.28 (t, J = 7.4 Hz, 1H), 7.21 (t, J = 7.3 Hz, 2H), 5.47 (dd, J = 16.8, 8.0 Hz, 2H), 4.81 (dd, J = 14.7, 9.2 Hz, 1H), 4.51 – 4.40 (m, 1H), 4.18 (s, 2H), 3.72 (s, 3H), 3.59 – 3.51 (m, 1H), 3.40 (dd, J = 9.1, 5.5 Hz, 1H), 3.12 (dd, J = 14.1, 5.4 Hz, 1H), 2.89 (dd, J = 13.8, 10.4 Hz, 1H), 2.53 – 2.47 (m, 2H), 2.29 – 2.24 (m, 1H), 2.19 – 2.06 (m, 3H), 1.88 – 1.80 (m, 4H), 1.47 (s, 9H), 1.44 (s, 9H), 1.40 (s, 9H), 1.31 (d, J = 6.8 Hz, 3H), 0.85 (t, J = 7.4 Hz, 3H), 0.36 (s, 3H), 0.27 (s, 3H). ^{13}C NMR (151 MHz, $CDCl_3$) δ = 172.6, 171.5, 171.0, 170.8, 163.7, 157.0, 155.2, 144.2, 140.1, 139.5, 139.0, 138.8, 138.1, 133.7, 129.0, 128.0, 126.7, 122.8, 83.1, 81.0, 79.5, 60.7, 53.2, 52.6, 50.3, 47.9, 47.1, 34.2, 33.4, 30.8, 28.4, 28.4, 28.3, 28.0, 24.9, 24.1, 18.5, 14.9, -1.2, -1.8. HR-MS (ESI) m/z calcd for $C_{49}H_{73}N_7NaO_{12}Si$ [M+Na$^+$]: 1002.4979, found: 1002.4984.

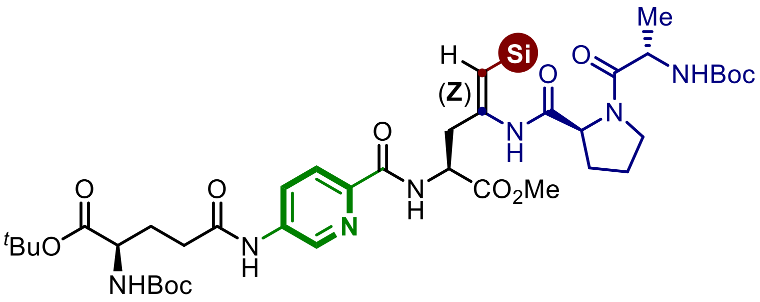

Methyl (*S*,*Z*)-2-{5-{(*S*)-5-(*tert*-butoxy)-4-[(*tert*-butoxycarbonyl)amino]-5-oxopentanamido}picolinamido}-4-{(*S*)-1-[(*tert*-butoxycarbonyl)-*L*-alanyl]pyrrolidine-2-carboxamido}-5-[dimethyl(phenyl)silyl]pent-4-enoate (53)

The general procedure **TP11** was followed using **1m** (80.0 mg, 0.15 mmol, 1.0 equiv.), **2u** (90.0 mg, 0.3 mmol, 2.0 equiv.), **3a** (1.0 mL, 0.3 mmol, 2.0 equiv.) and THF (0.5 mL) for 2 h. Purification by column chromatography (petroleum ether/EtOAc = 1:2) yielded **53** (78.5 mg, 55%) as a colorless oil. ^{1}H NMR (400 MHz, $CDCl_3$) δ = 9.77 (s, 1H), 8.75 (s, 1H), 8.41 – 8.29 (m, 2H), 8.09 (d, J = 8.5 Hz, 1H), 7.62 (s, 1H), 7.45 (d, J = 6.8 Hz, 2H), 7.31 (t, J = 7.3 Hz, 1H), 7.25 (t, J = 7.2 Hz, 2H), 5.50 (d, J = 7.5 Hz, 1H), 5.39 (d, J = 8.3 Hz, 1H), 5.16 (s, 1H), 4.82 (td, J = 9.1, 5.6 Hz, 1H), 4.52 – 4.38 (m, 1H), 4.21 (s, 1H), 4.05 (d, J = 5.7 Hz, 1H), 3.72 (s, 3H), 3.58 (dd, J = 17.8, 8.6 Hz,

1H), 3.51 – 3.39 (m, 2H), 2.78 (dd, *J* = 13.6, 10.2 Hz, 1H), 2.52 (t, *J* = 6.2 Hz, 2H), 2.32 – 2.24 (m, 1H), 2.00 – 1.79 (m, 5H), 1.46 (t, *J* = 10.7 Hz, 27H), 1.30 (d, *J* = 6.8 Hz, 3H), 0.30 (d, *J* = 32.0 Hz, 6H). ^{13}C NMR (101 MHz, $CDCl_3$) δ = 172.7, 172.4, 171.5, 171.1, 170.0, 164.0, 157.1, 155.3, 146.5, 144.3, 139.5, 138.1, 134.0, 129.4, 128.2, 126.7, 122.9, 119.8, 83.2, 81.0, 79.6, 60.6, 53.2, 52.5, 50.7, 47.9, 47.2, 40.9, 34.2, 30.9, 28.5, 28.4, 28.1, 25.0, 18.5, -1.66, -2.2. HR-MS (ESI) m/z calcd for $C_{47}H_{69}N_7NaO_{12}Si$ [M+Na$^+$]: 974.4666, found: 974.4659.

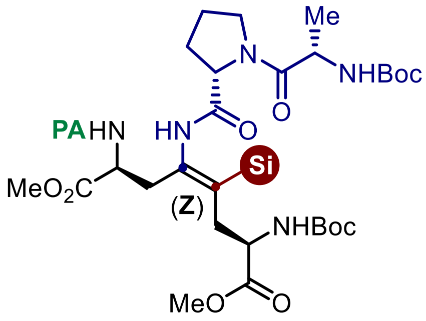

Dimethyl (2*S*,7*R*,*Z*)-4-{(*S*)-1-[(*tert*-butoxycarbonyl)-*L*-alanyl]pyrrolidine-2-carboxamido}-7-[(*tert*-butoxycarbonyl)amino]-5-[dimethyl(phenyl)silyl]-2-(picolinamido)oct-4-enedioate (54)

The general procedure **TP11** was followed using **1i** (65.0 mg, 0.15 mmol, 1.0 equiv.), **2u** (98.0 mg, 0.3 mmol, 2.0 equiv.), **3a** (1.0 mL, 0.3 mmol, 2.0 equiv.) and THF (0.5 mL) for 2 h. Purification by column chromatography (petroleum ether/EtOAc = 1:1.5) yielded **54** (96.0 mg, 75%) as a colorless oil. ^{1}H NMR (400 MHz, $CDCl_3$) δ = 8.57 (d, *J* = 4.5 Hz, 1H), 8.51 (d, *J* = 8.6 Hz, 1H), 8.11 (d, *J* = 7.8 Hz, 1H), 7.86 (t, *J* = 7.5 Hz, 1H), 7.47 (dd, *J* = 13.7, 8.8 Hz, 4H), 7.28 (t, *J* = 7.2 Hz, 1H), 7.20 (t, *J* = 7.2 Hz, 2H), 5.41 (d, *J* = 8.2 Hz, 1H), 5.16 (d, *J* = 7.8 Hz, 1H), 4.75 (dd, *J* = 15.4, 7.9 Hz, 1H), 4.55 – 4.38 (m, 1H), 4.26 (d, *J* = 7.0 Hz, 1H), 4.04 (d, *J* = 5.8 Hz, 1H), 3.74 (s, 3H), 3.73 (s, 3H), 3.61 – 3.53 (m, 1H), 3.49 (s, 1H), 3.21 (dd, *J* = 14.2, 6.4 Hz, 1H), 2.99 (dd, *J* = 14.3, 8.7 Hz, 1H), 2.72 – 2.56 (m, 2H), 1.95 – 1.71 (m, 4H), 1.43 (s, 18H), 1.31 (d, *J* = 6.8 Hz, 3H), 0.47 (s, 3H), 0.38 (s, 3H). ^{13}C NMR (101 MHz, $CDCl_3$) δ = 172.9, 172.6, 172.1, 170.1, 164.0, 155.1, 149.4, 148.3, 144.2, 138.3, 137.3, 133.7, 131.0, 129.2, 128.1, 126.4, 122.4, 80.0, 79.5, 60.3, 53.5, 52.6, 52.4, 50.5, 47.8, 47.2, 34.7, 34.0, 28.4, 28.3,

25.3, 25.0, 18.6, -1.2, -2.1. HR-MS (ESI) m/z calcd for $C_{42}H_{60}N_6NaO_{11}Si$ [M+Na$^+$]: 875.3982, found: 875.3973.

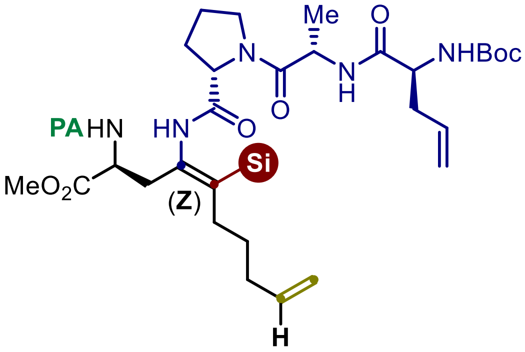

Methyl (*S*,*Z*)-4-{{{(*S*)-1-{{{(*S*)-2-[(*tert*-butoxycarbonyl)amino]pent-4-enoyl}-*L*-alanyl}}pyrrolidine-2-carboxamido}}}-5-[dimethyl(phenyl)silyl]-2-(picolinamido)deca-4,9-dienoate (55)

The general procedure **TP11** was followed using **1h** (30.0 mg, 0.1 mmol, 1.0 equiv.), **2y** (84.9 mg, 0.2 mmol, 2.0 equiv.), **3a** (0.5 mL, 0.2 mmol, 2.0 equiv.) and THF (0.5 mL) for 2 h. Purification by column chromatography (petroleum ether/EtOAc = 1:2) yielded **55** (30.0 mg, 37%) as a colorless oil. ^{1}H NMR (400 MHz, $CDCl_3$) δ = 8.58 (d, J = 4.5 Hz, 1H), 8.53 (d, J = 8.6 Hz, 1H), 8.12 (d, J = 7.7 Hz, 1H), 7.88 (t, J = 7.6 Hz, 1H), 7.50 – 7.45 (m, 1H), 7.42 – 7.37 (m, 3H), 7.32 – 7.26 (m, 2H), 7.19 (t, J = 7.3 Hz, 2H), 6.97 (d, J = 6.4 Hz, 1H), 5.70 (dt, J = 17.0, 9.6 Hz, 2H), 5.16 – 5.10 (m, 2H), 5.02 (d, J = 7.4 Hz, 1H), 4.89 (dt, J = 14.8, 12.4 Hz, 3H), 4.68 (dd, J = 13.9, 6.8 Hz, 1H), 4.14 (d, J = 18.2 Hz, 2H), 3.76 (s, 3H), 3.54 (d, J = 8.4 Hz, 1H), 3.40 (s, 1H), 3.18 (dd, J = 14.0, 5.5 Hz, 1H), 2.97 – 2.86 (m, 1H), 2.49 (dd, J = 13.2, 6.6 Hz, 2H), 2.11 (s, 2H), 1.98 (dd, J = 13.7, 6.9 Hz, 2H), 1.85 (d, J = 4.6 Hz, 3H), 1.42 (s, 9H), 1.32 (d, J = 6.5 Hz, 5H), 0.38 (s, 3H), 0.28 (s, 3H). ^{13}C NMR (101 MHz, $CDCl_3$) δ = 172.5, 171.7, 170.6, 170.5, 164.0, 155.5, 149.4, 148.4, 140.5, 138.8, 138.3, 137.4, 137.2, 133.8, 133.1, 129.1, 128.0, 126.5, 122.4, 119.1, 114.9, 80.2, 60.7, 53.9, 52.6, 50.3, 47.2, 47.0, 37.0, 34.0, 33.3, 30.7, 29.7, 28.4, 25.0, 18.1, -1.1, -1.9. HR-MS (ESI) m/z calcd for $C_{43}H_{60}N_6NaO_8Si$ [M+Na$^+$]: 839.4134, found: 839.4137.

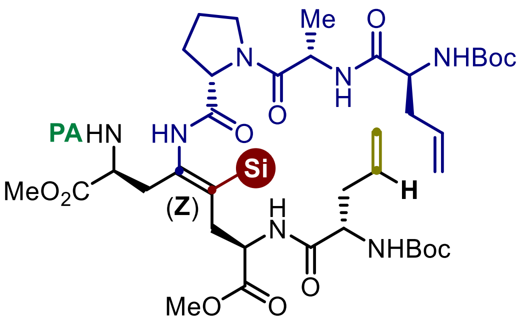

Dimethyl (2*R*,7*S*,*Z*)-2-{(*S*)-2-[(*tert*-butoxycarbonyl)amino]pent-4-enamido}-5-{{{(*S*)-1-{{{(*S*)-2-[(*tert*-butoxycarbonyl)amino]pent-4-enoyl}-*L*-alanyl}}pyrrolidine-2-carboxamido}}}-4-[dimethyl(phenyl)silyl]-7-(picolinamido)oct-4-enedioate (56)

The general procedure **TP11** was followed using **1o** (53.0 mg, 0.1 mmol, 1.0 equiv.), **2y** (84.9 mg, 0.2 mmol, 2.0 equiv.), **3a** (0.5 mL, 0.2 mmol, 2.0 equiv.) and THF (0.5 mL) for 2 h. Purification by column chromatography (petroleum ether/EtOAc = 1:1.5) yielded **56** (36.6 mg, 35%) as a colorless oil. ^{1}H NMR (600 MHz, $CDCl_3$) δ = 8.58 (d, *J* = 4.4 Hz, 2H), 8.13 (d, *J* = 7.4 Hz, 1H), 7.94 – 7.86 (m, 1H), 7.63 (s, 1H), 7.49 (dd, *J* = 7.1, 4.8 Hz, 1H), 7.32 (d, *J* = 7.2 Hz, 2H), 7.28 (s, 1H), 7.18 (t, *J* = 7.4 Hz, 2H), 7.01 (s, 1H), 6.75 (s, 1H), 5.99 (s, 1H), 5.82 – 5.69 (m, 2H), 5.16 – 5.07 (m, 4H), 5.03 (s, 1H), 4.89 (dd, *J* = 14.5, 8.5 Hz, 1H), 4.76 – 4.69 (m, 1H), 4.41 (s, 1H), 4.31 (s, 1H), 4.23 (s, 1H), 4.18 (s, 1H), 3.78 (s, 3H), 3.67 (s, 3H), 3.62 (s, 1H), 3.49 (s, 1H), 3.06 (s, 1H), 2.97 – 2.87 (m, 1H), 2.71 – 2.61 (m, 2H), 2.55 – 2.45 (m, 3H), 2.36 – 2.29 (m, 1H), 2.08 (s, 2H), 1.96 (s, 2H), 1.45 (s, 9H), 1.42 (s, 9H), 1.33 (d, *J* = 6.7 Hz, 3H), 0.38 (s, 3H), 0.32 (s, 3H). ^{13}C NMR (151 MHz, $CDCl_3$) δ = 172.0, 171.8, 171.6, 171.1, 170.7, 164.3, 156.0, 155.4, 149.1, 148.5, 143.4, 138.0, 137.5, 135.2, 133.9, 133.7, 133.0, 129.2, 128.1, 126.7, 122.5, 119.1, 118.4, 80.1, 79.5, 60.6, 53.9, 53.8, 52.9, 52.5, 51.4, 50.4, 47.3, 46.9, 38.7, 37.0, 36.8, 34.1, 33.5, 28.3, 25.0, 18.0, -1.7, -1.8. HR-MS (ESI) m/z calcd for $C_{52}H_{74}N_8NaO_{13}Si$ [M+Na$^+$]: 1069.5037, found: 1069.5042.

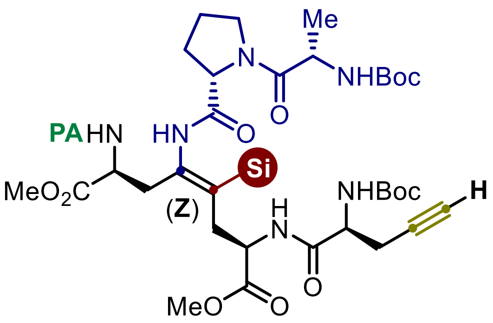

Dimethyl (2*S*,7*R*,*Z*)-4-{(*S*)-1-[(*tert*-butoxycarbonyl)-*L*-alanyl]pyrrolidine-2-carboxamido}-7-{(*S*)-2-[(*tert*-butoxycarbonyl)amino]pent-4-ynamido}-5-[dimethyl(phenyl)silyl]-2-(picolinamido)oct-4-enedioate (57)

The general procedure **TP11** was followed using **1p** (52.8 mg, 0.1 mmol, 1.0 equiv.), **2u** (65.4 mg, 0.2 mmol, 2.0 equiv.), **3a** (0.5 mL, 0.2 mmol, 2.0 equiv.) and THF (0.5 mL) for 2 h. Purification by column chromatography (petroleum ether/EtOAc = 1:3) yielded **57** (28.4 mg, 30%) as a colorless oil. ^{1}H NMR (600 MHz, $CDCl_3$) δ = 10.04 (d, J = 9.9 Hz, 1H), 8.71 (d, J = 9.2 Hz, 1H), 8.60 (s, 1H), 8.11 (d, J = 7.2 Hz, 1H), 7.87 (s, 1H), 7.63 (s, 2H), 7.49 (s, 1H), 7.28 (d, J = 8.6 Hz, 2H), 7.21 (s, 2H), 6.28 (d, J = 9.4 Hz, 1H), 6.04 (d, J = 6.8 Hz, 1H), 5.55 (d, J = 6.6 Hz, 1H), 5.02 (s, 1H), 4.95 (s, 1H), 4.67 (t, J = 9.6 Hz, 1H), 4.54 (s, 1H), 4.22 (s, 1H), 4.12 (s, 1H), 3.75 (s, 3H), 3.70 (s, 4H), 3.18 (t, J = 12.9 Hz, 1H), 2.88 (t, J = 12.3 Hz, 1H), 2.57 (d, J = 4.9 Hz, 2H), 2.34 (d, J = 13.1 Hz, 1H), 2.15 (d, J = 15.2 Hz, 2H), 2.02 (s, 1H), 1.91 (s, 2H), 1.44 (s, 21H), 0.47 (d, J = 8.2 Hz, 6H). ^{13}C NMR (151 MHz, $CDCl_3$) δ = 174.4, 171.5, 171.3, 170.8, 169.8, 164.5, 155.3, 154.6, 148.6, 144.4, 142.3, 137.5, 136.7, 134.6, 129.2, 127.7, 126.8, 122.9, 117.9, 79.9, 79.4, 59.6, 56.0, 53.1, 53.0, 52.5, 50.9, 48.0, 47.3, 40.1, 39.1, 36.5, 29.9, 28.5, 28.4, 28.4, 24.8, 18.8, -1.6. HR-MS (ESI) m/z calcd for $C_{47}H_{65}N_7NaO_{12}Si$ [M+Na$^+$]: 970.4353, found: 970.4357.

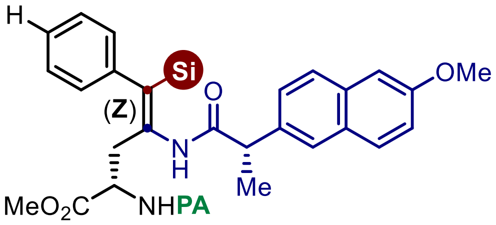

Methyl (*S*,*Z*)-5-[dimethyl(phenyl)silyl]-4-[(*S*)-2-(6-methoxynaphthalen-2-yl)propanamido]-5-phenyl-2-(picolinamido)pent-4-enoate (58)

The general procedure **TP11** was followed using **1k** (43.8 mg, 0.15 mmol, 1.0 equiv.), **2ac** (81.4 mg, 0.3 mmol, 2.0 equiv.), **3a** (1.0 mL, 0.3 mmol, 2.0 equiv.) and THF (0.5 mL) for 2 h. Purification by column chromatography (petroleum ether/EtOAc = 3:1) yielded **58** (56.0 mg, 56%) as a yellow oil. ^{1}H NMR (600 MHz, $CDCl_3$) δ = 8.61 – 8.56 (m, 1H), 8.25 (d, J = 9.1 Hz, 1H), 8.12 (d, J = 7.8 Hz, 1H), 7.87 (td, J = 7.7, 1.7 Hz, 1H), 7.64 (d, J = 9.0 Hz, 1H), 7.58 (d, J = 8.5 Hz, 1H), 7.44 (ddd, J = 7.6, 4.7, 1.0 Hz, 2H), 7.31 – 7.28 (m, 2H), 7.26 (dd, J = 8.4, 1.6 Hz, 1H), 7.23 (t, J = 3.7 Hz, 2H), 7.20 – 7.12 (m, 2H), 7.11 – 7.07 (m, 3H), 7.03 (d, J = 2.4 Hz, 1H), 7.01 (s, 1H), 6.98 (d, J = 7.2 Hz, 1H), 6.68 (d, J = 6.8 Hz, 1H), 4.52 (ddd, J = 11.1, 9.2, 4.3 Hz, 1H), 3.88 (s, 3H), 3.46 (s, 3H), 3.27 (q, J = 7.1 Hz, 1H), 3.05 (dd, J = 14.0, 4.3 Hz, 1H), 2.63 (dd, J = 13.9, 11.2 Hz, 1H), 1.41 (d, J = 7.1 Hz, 3H), 0.00 (s, 3H), -0.19 (s, 3H). ^{13}C NMR (151 MHz, $CDCl_3$) δ = 173.5, 172.0, 164.0, 157.6, 149.4, 148.4, 142.4, 141.1, 138.4, 137.7, 137.4, 136.0, 133.8, 133.65, 129.6, 129.3, 129.1, 128.6, 128.2, 127.9, 127.5, 126.5, 126.5, 126.1, 126.1, 122.5, 118.8, 105.7, 55.4, 52.4, 50.2, 47.4, 34.3, 18.2, -1.9, -2.1. HR-MS (ESI) m/z calcd for $C_{40}H_{41}N_3NaO_5Si$ [M+Na$^+$]: 694.2708, found: 694.2715.

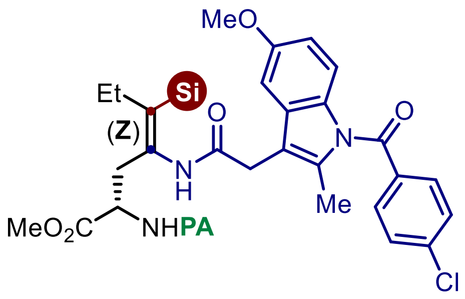

Methyl (*S*,*Z*)-4-{2-[1-(4-chlorobenzoyl)-5-methoxy-2-methyl-1*H*-indol-3-yl]acetamido}-5-[dimethyl(phenyl)silyl]-2-(picolinamido)hept-4-enoate (59)

The general procedure **TP11** was followed using **1c** (26.0 mg, 0.1 mmol, 1.0 equiv.), **2ad** (79.8 mg, 0.2 mmol, 2.0 equiv.), **3a** (0.5 mL, 0.2 mmol, 2.0 equiv.) and THF (0.5 mL) for 2 h. Purification by column chromatography (petroleum ether/EtOAc = 2:1) yielded **59** (54.0 mg, 72%) as a yellow oil. ^{1}H NMR (400 MHz, $CDCl_3$) δ = 8.55 (d, J = 4.2 Hz, 1H), 8.45 (d, J = 8.9 Hz, 1H), 7.95 (d, J = 7.8 Hz, 1H), 7.82 (t, J = 7.7 Hz, 1H), 7.73 (d, J = 8.2 Hz, 2H), 7.47 – 7.41 (m, 1H), 7.33 (d, J = 7.1 Hz, 3H), 7.26 (t, J = 6.6 Hz, 3H), 7.16 (t, J = 7.5 Hz, 2H), 6.97 (d, J = 2.0 Hz, 1H), 6.76 (d, J = 9.0 Hz,

1H), 6.60 (dd, J = 9.0, 1.9 Hz, 1H), 4.57 – 4.15 (m, 1H), 3.81 (s, 3H), 3.70 (s, 3H), 3.41 (q, J = 16.4 Hz, 2H), 2.97 (dd, J = 13.9, 4.0 Hz, 1H), 2.90 – 2.72 (m, 1H), 2.31 (s, 3H), 2.03 (q, J = 7.1 Hz, 2H), 0.72 (t, J = 7.4 Hz, 3H), 0.21 (s, 3H), 0.18 (s, 3H). ^{13}C NMR (101 MHz, $CDCl_3$) δ = 172.3, 170.3, 168.4, 163.9, 156.2, 149.0, 148.5, 141.3, 139.8, 139.1, 138.6, 137.3, 136.4, 134.1, 133.5, 131.5, 131.1, 130.6, 129.1, 128.9, 127.8, 126.6, 122.2, 115.2, 113.0, 112.2, 100.6, 55.6, 52.7, 49.9, 34.4, 32.8, 24.2, 14.6, 13.3, -1.6, -1.6. HR-MS (ESI) m/z calcd for $C_{41}H_{43}ClN_4NaO_6Si$ [M+Na$^+$]: 773.2533, found: 773.2537.

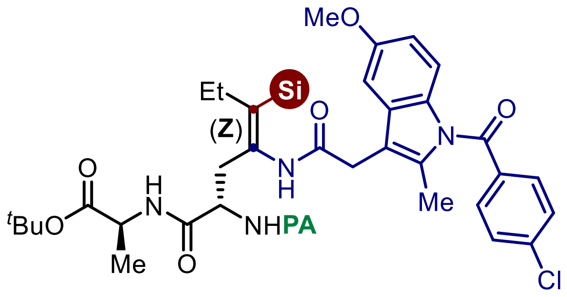

tert-Butyl {{(S,Z)-4-{2-[1-(4-chlorobenzoyl)-5-methoxy-2-methyl-1H-indol-3-yl]acetamido}-5-[dimethyl(phenyl)silyl]-2-(picolinamido)hept-4-enoyl}}-L-alaninate (60)

The general procedure **TP11** was followed using **1q** (37.4 mg, 0.1 mmol, 1.0 equiv.), **2ad** (79.8 mg, 0.2 mmol, 2.0 equiv.), **3a** (0.5 mL, 0.2 mmol, 2.0 equiv.) and THF (0.5 mL) for 2 h. Purification by column chromatography (petroleum ether/EtOAc = 2:1) yielded **60** (45.0 mg, 52%) as a yellow oil. ^{1}H NMR (400 MHz, $CDCl_3$) δ = 8.66 (d, J = 8.6 Hz, 1H), 8.56 (d, J = 4.1 Hz, 1H), 7.99 (d, J = 7.8 Hz, 1H), 7.82 (td, J = 7.7, 1.7 Hz, 1H), 7.70 (d, J = 8.7 Hz, 2H), 7.45 – 7.41 (m, 1H), 7.37 (d, J = 7.7 Hz, 2H), 7.31 – 7.24 (m, 4H), 7.17 (t, J = 7.2 Hz, 2H), 7.06 (d, J = 2.4 Hz, 1H), 6.83 (d, J = 9.0 Hz, 1H), 6.62 (dd, J = 9.0, 2.5 Hz, 1H), 6.32 (d, J = 7.5 Hz, 1H), 4.39 (p, J = 7.2 Hz, 1H), 4.28 – 4.14 (m, 1H), 3.81 (s, 3H), 3.38 (dd, J = 33.6, 16.2 Hz, 2H), 2.94 (dd, J = 14.3, 4.9 Hz, 1H), 2.82 (dd, J = 14.1, 10.2 Hz, 1H), 2.27 (s, 3H), 2.07 (q, J = 7.4 Hz, 2H), 1.46 (s, 9H), 1.32 (d, J = 7.2 Hz, 3H), 0.76 (t, J = 7.5 Hz, 3H), 0.23 (s, 3H), 0.22 (s, 3H). ^{13}C NMR (101 MHz, $CDCl_3$) δ = 171.3, 170.4, 170.2, 168.4, 163.8, 156.0, 149.4, 148.6, 140.0, 140.0, 139.1, 138.8, 137.2, 136.6, 134.1, 133.5, 131.4, 131.2, 130.8, 129.1, 129.0,

127.9, 126.4, 122.0, 115.4, 113.0, 112.3, 101.0, 81.8, 56.0, 50.7, 48.9, 35.6, 32.8, 28.1, 24.2, 18.1, 14.6, 13.4, -1.4, -1.7. HR-MS (ESI) m/z calcd for $C_{47}H_{54}ClN_5NaO_7Si$ [M+Na$^+$]: 886.3373, found: 886.3369.

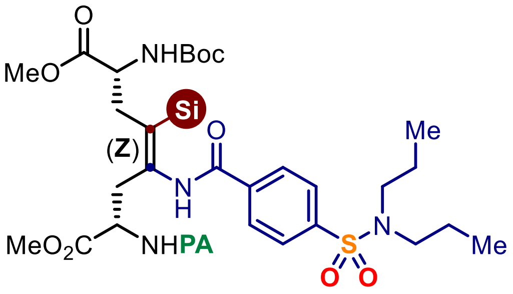

Dimethyl (2*R*,7*S*,*Z*)-2-[(*tert*-butoxycarbonyl)amino]-4-[dimethyl(phenyl)silyl]-5-[4-(*N*,*N*-dipropylsulfamoyl)benzamido]-7-(picolinamido)oct-4-enedioate (61)

The general procedure **TP11** was followed using **1i** (43.4 mg, 0.1 mmol, 1.0 equiv.), **2ae** (65.2 mg, 0.2 mmol, 2.0 equiv.), **3a** (0.5 mL, 0.2 mmol, 2.0 equiv.) and THF (0.5 mL) for 2 h. Purification by column chromatography (petroleum ether/EtOAc = 2.5:1 to 2:1) yielded **61** (40.2 mg, 47%) as a colorless oil. ^{1}H NMR (400 MHz, $CDCl_3$) δ = 8.56 (d, J = 12.9 Hz, 2H), 8.09 (d, J = 7.6 Hz, 1H), 7.88 (t, J = 7.1 Hz, 1H), 7.65 (d, J = 7.7 Hz, 2H), 7.48 (s, 2H), 7.37 (d, J = 6.8 Hz, 3H), 7.28 (s, 2H), 7.20 (t, J = 7.1 Hz, 2H), 5.34 (d, J = 8.8 Hz, 1H), 4.93 (dd, J = 12.8, 8.3 Hz, 1H), 4.36 (d, J = 6.9 Hz, 1H), 3.81 (s, 3H), 3.67 (s, 3H), 3.39 (d, J = 12.7 Hz, 1H), 3.19 (dd, J = 14.5, 8.9 Hz, 1H), 3.09 (t, J = 7.4 Hz, 4H), 2.83 – 2.68 (m, 2H), 1.56 (d, J = 7.5 Hz, 4H), 1.47 (s, 9H), 0.88 (d, J = 7.2 Hz, 6H), 0.37 (s, 3H), 0.32 (s, 3H). ^{13}C NMR (101 MHz, $CDCl_3$) δ = 172.8, 172.0, 164.2, 164.1, 149.0, 148.3, 144.1, 143.0, 138.0, 137.5, 136.6, 133.5, 129.7, 128.7, 127.8, 127.0, 126.7, 122.5, 80.2, 53.7, 52.8, 52.5, 50.8, 50.0, 34.3, 32.6, 28.4, 22.0, 11.3, -1.9, -2.4. HR-MS (ESI) m/z calcd for $C_{42}H_{57}N_5NaO_{10}SSi$ [M+Na$^+$]: 874.3488, found: 874.3492.

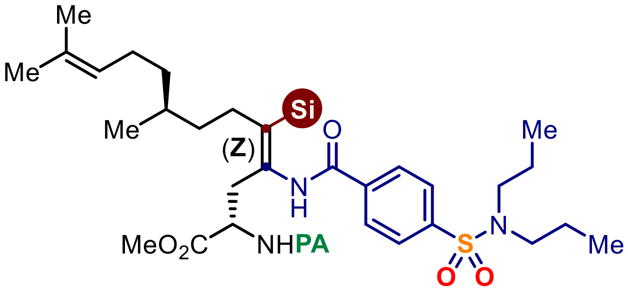

Methyl (2*S*,8*S*,*Z*)-5-[dimethyl(phenyl)silyl]-4-[4-(*N*,*N*-dipropylsulfamoyl)benzamido]-8,12-dimethyl-2-(picolinamido)trideca-4,11-dienoate (62)

The general procedure **TP11** was followed using **1j** (37.0 mg, 0.1 mmol, 1.0 equiv.), **2ae** (65.2 mg, 0.2 mmol, 2.0 equiv.), **3a** (0.5 mL, 0.2 mmol, 2.0 equiv.) and THF (0.5 mL) for 2 h. Purification by column chromatography (petroleum ether/EtOAc = 1:1) yielded **62** (40.0 mg, 51%) as a colorless oil. ^{1}H NMR (400 MHz, $CDCl_3$) δ = 8.63 (d, J = 8.9 Hz, 1H), 8.55 (s, 1H), 8.15 (d, J = 7.7 Hz, 1H), 7.91 (t, J = 7.6 Hz, 1H), 7.71 (d, J = 8.0 Hz, 2H), 7.63 (s, 1H), 7.48 (d, J = 6.8 Hz, 3H), 7.29 (d, J = 8.6 Hz, 3H), 7.15 (t, J = 7.2 Hz, 2H), 5.10 (t, J = 6.5 Hz, 1H), 4.94 (t, J = 9.5 Hz, 1H), 3.71 (s, 3H), 3.30 (d, J = 13.4 Hz, 1H), 3.07 (dd, J = 19.1, 10.7 Hz, 5H), 2.20 (s, 2H), 2.03 – 1.89 (m, 2H), 1.71 (s, 3H), 1.62 (s, 3H), 1.56 (dd, J = 14.3, 7.2 Hz, 4H), 1.35 (dd, J = 30.5, 13.3 Hz, 3H), 1.14 – 1.04 (m, 2H), 0.88 (dd, J = 15.9, 7.9 Hz, 9H), 0.26 (s, 3H), 0.23 (s, 3H). ^{13}C NMR (101 MHz, $CDCl_3$) δ = 172.2, 164.3, 164.2, 149.1, 148.4, 142.8, 139.7, 139.1, 138.4, 137.4, 137.0, 133.5, 131.3, 129.3, 128.4, 127.9, 127.1, 126.7, 124.9, 122.4, 52.7, 50.6, 50.1, 37.8, 36.9, 33.1, 28.9, 25.8, 25.7, 22.1, 19.5, 17.8, 11.3, -1.8, -2.0. HR-MS (ESI) m/z calcd for $C_{43}H_{60}N_4NaO_6SSi$ [M+Na$^+$]: 811.3895, found: 811.3889.

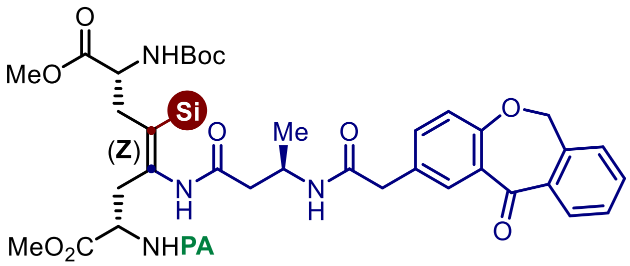

Dimethyl (2*R*,7*S*,*Z*)-2-[(*tert*-butoxycarbonyl)amino]-4-[dimethyl(phenyl)silyl]-5-{(*R*)-3-[2-(11-oxo-6,11-dihydrodibenzo[*b*,*e*]oxepin-2-yl)acetamido]butanamido}-7-(picolinamido)oct-4-enedioate (63)

The general procedure **TP11** was followed using **1i** (43.4 mg, 0.1 mmol, 1.0 equiv.), **2af** (78.9 mg, 0.2 mmol, 2.0 equiv.), **3a** (0.5 mL, 0.2 mmol, 2.0 equiv.) and THF (0.5 mL) for 2 h. Purification by column chromatography (petroleum ether/EtOAc = 1:2) yielded **63** (56.0 mg, 61%) as a colorless oil. ^{1}H NMR (600 MHz, $CDCl_3$) δ = 8.57 (d,

J = 4.6 Hz, 1H), 8.52 (d, J = 9.1 Hz, 1H), 8.11 (d, J = 7.8 Hz, 1H), 8.08 (d, J = 2.1 Hz, 1H), 7.89 (dd, J = 10.6, 4.5 Hz, 2H), 7.55 (t, J = 7.4 Hz, 1H), 7.50 – 7.44 (m, 3H), 7.41 (d, J = 7.1 Hz, 2H), 7.36 (d, J = 7.4 Hz, 1H), 7.30 (t, J = 7.4 Hz, 1H), 7.21 (t, J = 7.4 Hz, 2H), 7.01 (d, J = 8.4 Hz, 1H), 6.89 (d, J = 8.2 Hz, 1H), 6.59 (s, 1H), 5.20 (d, J = 8.4 Hz, 1H), 5.17 (s, 2H), 4.85 (td, J = 9.1, 5.8 Hz, 1H), 4.31 (d, J = 7.7 Hz, 1H), 4.16 – 4.10 (m, 1H), 3.75 (s, 3H), 3.73 (s, 3H), 3.50 (q, J = 15.2 Hz, 2H), 3.30 (dd, J = 14.3, 4.9 Hz, 1H), 3.03 (dd, J = 14.5, 9.2 Hz, 1H), 2.66 (d, J = 6.9 Hz, 2H), 1.86 – 1.81 (m, 2H), 1.43 (s, 9H), 1.11 (d, J = 6.8 Hz, 3H), 0.39 (s, 3H), 0.33 (s, 3H). ^{13}C NMR (151 MHz, $CDCl_3$) δ = 190.9, 172.8, 171.9, 170.0, 169.7, 164.1, 160.4, 155.2, 149.1, 148.5, 144.0, 140.5, 137.5, 136.4, 135.7, 133.4, 133.1, 132.8, 132.4, 129.8, 129.7, 129.6, 129.3, 129.3, 128.6, 128.0, 127.9, 126.7, 125.2, 122.5, 121.2, 80.2, 73.7, 53.6, 52.8, 52.5, 50.7, 42.6, 42.4, 41.3, 34.2, 28.4, 28.3, 19.8, -2.1, -2.1. HR-MS (ESI) m/z calcd for $C_{49}H_{57}N_5NaO_{11}Si$ [M+Na$^+$]: 942.3716, found: 942.3722.

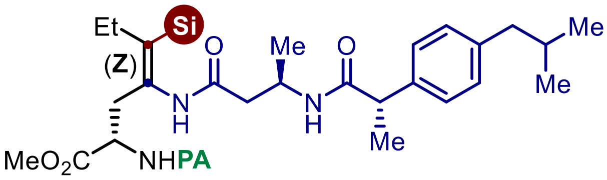

Methyl (*S*,*Z*)-5-[dimethyl(phenyl)silyl]-4-{(*R*)-3-[(*S*)-2-(4-isobutylphenyl)propanamido]butanamido}-2-(picolinamido)hept-4-enoate (64)

The general procedure **TP11** was followed using **1c** (39.0 mg, 0.15 mmol, 1.0 equiv.), **2ag** (99.6 mg, 0.3 mmol, 2.0 equiv.), **3a** (1.0 mL, 0.3 mmol, 2.0 equiv.) and THF (0.5 mL) for 2 h. Purification by column chromatography (petroleum ether/EtOAc = 1.5:1 to 1:1) yielded **64** (64.3 mg, 63%) as a colorless oil. ^{1}H NMR (400 MHz, $CDCl_3$) δ = 8.57 (dd, J = 11.6, 6.7 Hz, 2H), 8.13 (d, J = 7.8 Hz, 1H), 7.88 (td, J = 7.7, 1.5 Hz, 1H), 7.50 – 7.44 (m, 1H), 7.41 – 7.33 (m, 2H), 7.31 – 7.27 (m, 1H), 7.24 – 7.16 (m, 4H), 7.07 (d, J = 8.0 Hz, 2H), 6.70 (s, 1H), 6.62 (d, J = 8.3 Hz, 1H), 4.86 (td, J = 9.7, 5.0 Hz, 1H), 4.24 – 4.08 (m, 1H), 3.75 (s, 3H), 3.52 (q, J = 7.1 Hz, 1H), 3.19 (dd, J = 14.3, 5.0 Hz, 1H), 2.96 (dd, J = 14.2, 10.2 Hz, 1H), 2.43 (d, J = 7.1 Hz, 2H), 2.25 – 2.08 (m, 3H), 1.89 – 1.78 (m, 2H), 1.45 (d, J = 7.1 Hz, 3H), 1.03 (d, J = 6.8 Hz, 3H), 0.94 (t, J = 7.3 Hz, 3H), 0.89 (s, 3H), 0.88 (s, 3H), 0.30 (s, 3H), 0.28 (s, 3H). ^{13}C NMR (101 MHz,

$CDCl_3$) δ = 173.7, 172.2, 170.2, 164.0, 149.1, 148.4, 140.3, 139.9, 139.0, 138.4, 138.2, 137.4, 133.4, 129.4, 129.3, 128.3, 127.2, 126.6, 122.4, 52.6, 50.6, 46.5, 45.1, 42.3, 41.1, 32.9, 30.2, 23.9, 22.4, 19.8, 18.7, 14.9, -1.8, -1.9. HR-MS (ESI) m/z calcd for $C_{39}H_{52}N_4NaO_5Si$ [M+Na$^+$]: 707.3599, found: 707.3590.

Gram-Scale Experiments and Synthetic Applications

a) Scale up synthesis and transformation of sila-group

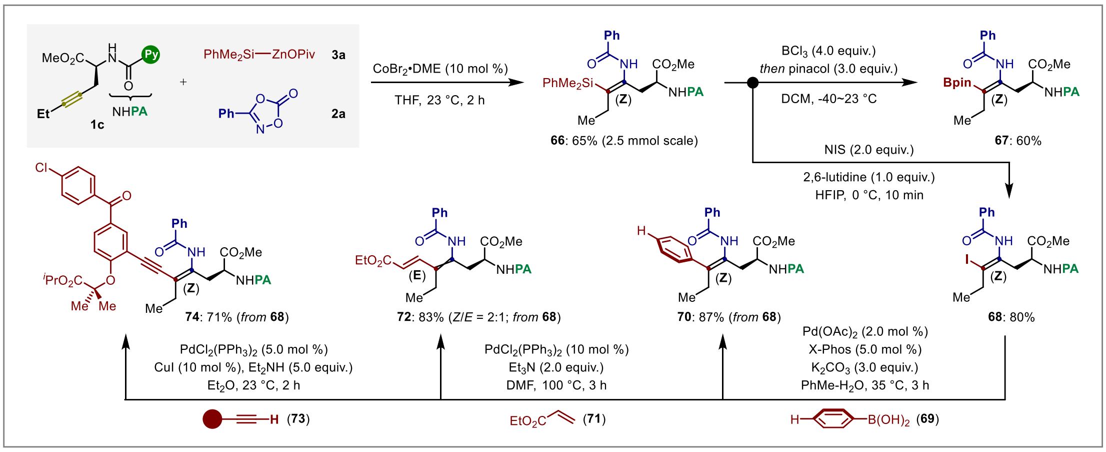

Figure S27. Scale up synthesis and transformation of sila-group.

i) Gram-scale experiment

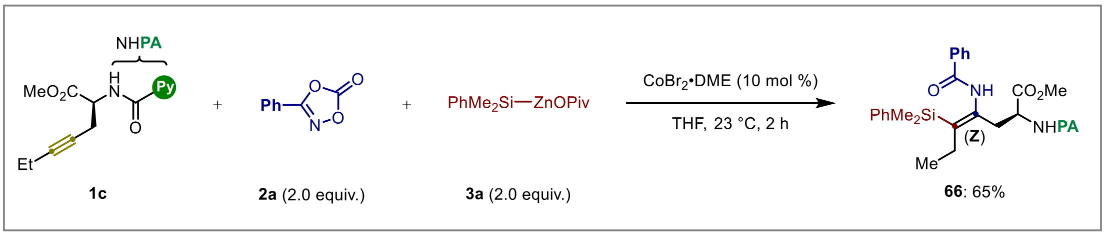

Figure S28. Co-catalyzed silylamidation on gram scale.

In a nitrogen-filled glovebox, **1c** (651 mg, 2.5 mmol, 1.0 equiv.), **2a** (815 mg, 5.0 mmol, 2.0 equiv.), $CoBr_2$•DME (77 mg, 0.25 mmol, 10 mol %), and anhydrous THF (5.0 mL) were added to an oven-dried 100-mL Schlenk tube equipped with a Teflon-coated magnetic stir bar. The Schlenk tube was sealed with a rubber stopper and removed from the glovebox. A solution of silylzinc pivalate **3a** (5.0 mmol, 2.0 equiv.) in anhydrous THF (15.0 mL) was then added dropwise via syringe to the reaction mixture at 23 °C under argon atmosphere with stirring. The reaction was stirred under argon atmosphere at 23 °C for 2 h. The mixture was then diluted with DCM (10 mL) and quenched with saturated aq. $NaHCO_3$ solution (10 mL). The resulting mixture was extracted with DCM (20 mL × 3). The combined organic layers were dried over Na_2SO_4, filtered, and

concentrated under reduced pressure. The crude product was purified by column chromatography (petroleum ether/EtOAc = 3:1) on silica gel to afford the desired silylamidation product **66** (838 mg, 65%) as a colorless oil.

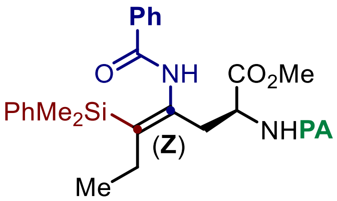

Methyl (*S*,*Z*)-4-benzamido-5-[dimethyl(phenyl)silyl]-2-(picolinamido)hept-4-enoate (66)

^{1}H NMR (400 MHz, $CDCl_3$) δ = 8.63 (d, *J* = 8.8 Hz, 1H), 8.53 (d, *J* = 4.1 Hz, 1H), 8.16 (d, *J* = 7.8 Hz, 1H), 7.87 (td, *J* = 7.7, 1.5 Hz, 1H), 7.50 – 7.41 (m, 3H), 7.39 – 7.32 (m, 4H), 7.31 – 7.25 (m, 3H), 7.16 (t, *J* = 7.4 Hz, 2H), 4.94 (td, *J* = 9.3, 4.9 Hz, 1H), 3.65 (s, 3H), 3.34 (dd, *J* = 14.3, 4.8 Hz, 1H), 3.14 (dd, *J* = 14.2, 9.6 Hz, 1H), 2.26 (dd, *J* = 14.4, 7.0 Hz, 2H), 0.98 (t, *J* = 7.5 Hz, 3H), 0.26 (d, *J* = 5.4 Hz, 6H). ^{13}C NMR (101 MHz, $CDCl_3$) δ = 172.4, 165.8, 164.1, 149.2, 148.3, 140.3, 139.4, 138.5, 137.3, 133.6, 133.5, 131.6, 129.2, 128.4, 128.3, 127.2, 126.5, 122.4, 52.6, 50.7, 32.9, 24.1, 15.0, -1.9, -2.0. HR-MS (ESI) m/z calcd for $C_{29}H_{33}N_3NaO_4Si$ $[M+Na^+]$: 538.2133, found: 538.2136.

ii) Desilylative borylation of compound **66**

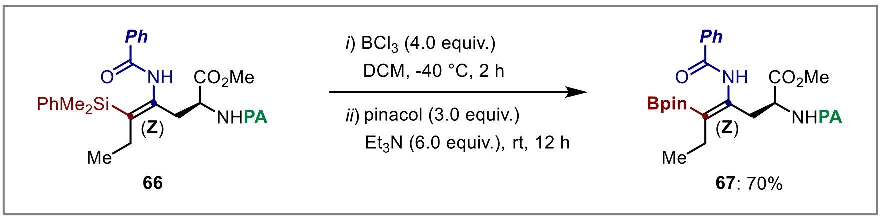

Figure S29. Desilylative borylation of **66**.

Following a modified literature procedure,[14] to a solution of **66** (67 mg, 0.13 mmol, 1.0 equiv.) in anhydrous DCM (0.5 mL) was added a solution of BCl_3 (0.52 mmol, 4.0 equiv., 1.0 M in hexane) at -40 ℃. After stirring the mixture at -40 ℃ for 2 h, a solution of pinacol (46 mg, 0.39 mmol, 3.0 equiv.) and Et_3N (78.9 mg, 0.78 mmol, 6.0 equiv.) in anhydrous DCM (0.5 mL) was added to the mixture. After stirring the mixture at

room temperature for 12 h, saturated aq $NaHCO_3$ (ca. 2 mL) was added to the mixture. Organic phase was separated and aqueous phase was extracted by DCM (10 mL × 1). The combined organic layers were dried over Na_2SO_4, filtered, and concentrated under reduced pressure. The crude product was purified by column chromatography (petroleum ether/EtOAc = 1:1.5) on silica gel to afford the desired product **67** (39.5 mg, 60%) as a white solid. ^{1}H NMR (400 MHz, $CDCl_3$) δ = 10.54 (s, 1H), 9.00 (d, J = 7.2 Hz, 1H), 8.53 (d, J = 4.6 Hz, 1H), 8.15 (d, J = 7.8 Hz, 1H), 8.07 – 7.99 (m, 2H), 7.83 (td, J = 7.7, 1.7 Hz, 1H), 7.54 (t, J = 7.3 Hz, 1H), 7.49 – 7.39 (m, 3H), 4.76 (dd, J = 12.6, 6.5 Hz, 1H), 3.79 (s, 3H), 3.41 (dd, J = 14.8, 6.5 Hz, 1H), 3.09 – 2.97 (m, 1H), 2.31 – 2.15 (m, 2H), 1.29 (s, 6H), 1.29 (s, 6H), 1.06 (t, J = 7.5 Hz, 3H). ^{13}C NMR (101 MHz, $CDCl_3$) δ = 172.3, 168.7, 164.9, 149.0, 148.5, 137.3, 136.0, 132.5, 132.2, 128.7, 127.6, 126.7, 122.3, 81.5, 52.9, 52.2, 32.1, 25.9, 22.0, 15.5. ^{11}B NMR (128 MHz, $CDCl_3$): δ 17.52. HR-MS (ESI) m/z calcd for $C_{27}H_{34}BN_3NaO_6$ [M+Na$^+$]: 530.2433, found: 530.2436.

iii) Iododesilylation of compound **66**

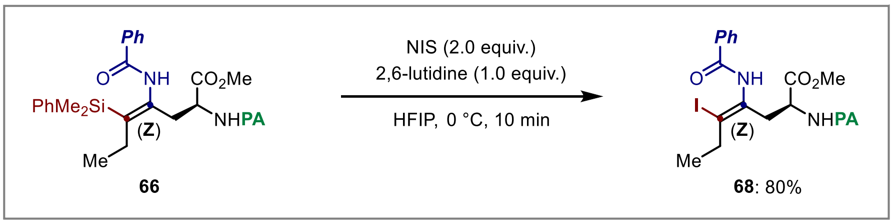

Figure S30. Desilylative iodization of compound **66**.

Following a modified literature procedure,[15] N-Iodosuccinimide (225 mg, 1.0 mmol, 2.0 equiv.) was added to a solution of substrate **66** (258 mg, 0.5 mmol, 1.0 equiv.) and 2,6-lutidine (54 mg, 0.5 mmol, 1.0 equiv.) in hexafluoroisopropanol (HFIP) (3.0 mL) at 0 ℃ and stirred for 10 min. EtOAc (15 mL) and H_2O (5 mL) were added to the reaction mixture. The phases were separated and the aqueous phase was extracted with EtOAc (15 mL × 3). The combined organic phases were washed with saturated aqueous sodium thiosulfate (15 mL), H_2O (10 mL), brine, dried over Na_2SO_4, filtered, and concentrated under reduced pressure. The crude product was purified by column chromatography (petroleum ether/EtOAc = 2.5:1) on silica gel to afford the desired

product **68** (202 mg, 80%) as a yellow solid. ^{1}H NMR (400 MHz, $CDCl_3$) δ = 8.71 (d, J = 8.5 Hz, 1H), 8.53 (d, J = 4.6 Hz, 1H), 8.15 (d, J = 7.8 Hz, 1H), 8.00 – 7.88 (m, 2H), 7.84 (td, J = 7.7, 1.6 Hz, 1H), 7.79 (s, 1H), 7.58 – 7.52 (m, 1H), 7.51 – 7.32 (m, 3H), 5.02 – 4.96 (m, 1H), 3.72 (s, 3H), 3.45 (dd, J = 14.5, 4.7 Hz, 1H), 3.31 (dd, J = 14.4, 9.2 Hz, 1H), 2.67 – 2.46 (m, 2H), 0.99 (t, J = 7.3 Hz, 3H). ^{13}C NMR (101 MHz, $CDCl_3$) δ = 171.9, 165.8, 164.5, 149.2, 148.4, 137.5, 134.0, 132.1, 128.9, 127.5, 126.6, 122.4, 108.3, 52.9, 51.1, 33.1, 32.2, 15.0. HR-MS (ESI) m/z calcd for $C_{21}H_{23}IN_3O_4$ [M+H$^+$]: 508.0728, found: 508.0731.

iv) Suzuki cross-coupling reaction of compound **68**

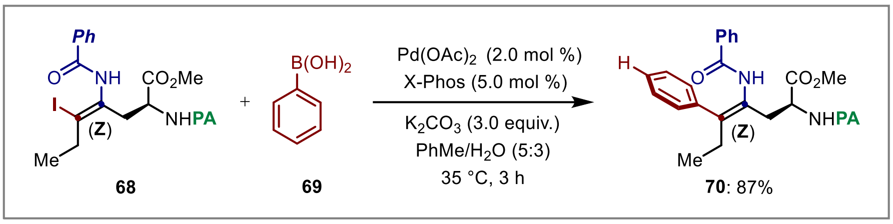

Figure S31. Suzuki cross-coupling reaction of compound **68**.

Following a modified literature procedure,[16] to an oven-dried 10-mL scintillation vial equipped with a spin vane triangular-shaped Teflon stirbar were added $Pd(OAc)_2$ (0.5 mg, 2.0 mol %), X-phos (2.4 mg, 5.0 mol %), **68** (50.7 mg, 0.1 mmol, 1.0 equiv.), **69** (24.4 mg, 0.2 mmol, 2.0 equiv.) and K_2CO_3 (41.5 mg, 0.3 mmol, 3.0 equiv.). To the vial were added argon-bubbled toluene/water = 5:3 (0.68 mL) under atmospheric condition and then sealed. The reaction mixture was vigorously stirred at 35 °C for 3 h. The reaction mixture was diluted with EtOAc and washed with brine. The organic layer was dried over Na_2SO_4, filtered, and concentrated under reduced pressure. The crude product was purified by column chromatography (petroleum ether/EtOAc = 2.5:1) on silica gel to afford the desired product **70** (40 mg, 87%) as a yellow oil. ^{1}H NMR (400 MHz, $CDCl_3$) δ = 8.80 (d, J = 8.7 Hz, 1H), 8.57 (d, J = 4.7 Hz, 1H), 8.20 (d, J = 7.8 Hz, 1H), 7.88 (td, J = 7.7, 1.6 Hz, 1H), 7.59 – 7.50 (m, 2H), 7.43 (ddd, J = 18.4, 8.7, 4.1 Hz, 3H), 7.32 (t, J = 7.6 Hz, 2H), 7.28 – 7.22 (m, 2H), 7.18 (dd, J = 8.6, 6.1 Hz, 1H), 7.10 – 6.97 (m, 2H), 5.06 (td, J = 9.2, 4.6 Hz, 1H), 3.74 (s, 3H), 3.43 (dd, J = 14.2, 4.6 Hz, 1H), 3.25 (dd, J = 14.2, 9.5 Hz, 1H), 2.52 – 2.27 (m, 2H), 0.80 (t, J = 7.5 Hz, 3H).

^{13}C NMR (101 MHz, $CDCl_3$) δ = 172.4, 166.4, 164.5, 149.4, 148.5, 140.7, 139.8, 137.58, 134.58, 131.58, 128.6, 128.1, 127.2, 126.6, 122.3, 52.7, 51.1, 33.1, 26.4, 12.8. HR-MS (ESI) m/z calcd for $C_{27}H_{28}N_3O_4$ [M+H$^+$]: 458.2074, found: 458.2077.

v) Heck cross-coupling reaction of compound **68**

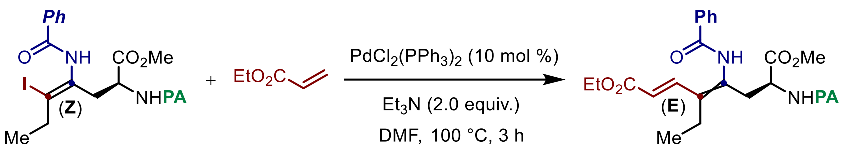

Figure S32. Heck cross-coupling reaction of compound **68**.

Following a modified literature procedure,[17] ethyl acrylate **71** (20.0 mg, 0.2 mmol, 2.5 equiv.) was added to a stirred mixture of **68** (40.6 mg, 0.08 mmol, 1.0 equiv.), $PdCl_2(PPh_3)_2$ (5.6 mg, 10 mol %, 0.008 mmol), NEt_3 (16.2 mg, 0.16 mmol, 2.0 equiv.) in DMF (1 mL) and stirred at 100 °C under argon for 3 h. The mixture was quenched with sat. aq. NaCl (10 mL) The aqueous layer was extracted with EtOAc (15 mL × 3). The combined organic layers were washed with brine, dried over anhydrous Na_2SO_4, filtered, and concentrated under reduced pressure. The crude product was purified by column chromatography (petroleum ether/EtOAc = 2:1) on silica gel to afford the desired product **72** (32 mg, 83%) as a yellow oil. ^1H NMR (400 MHz, $CDCl_3$) δ = 8.76 (d, J = 8.8 Hz, 1H), 8.53 (dd, J = 18.5, 4.6 Hz, 1H), 8.30 (s, 1H), 8.10 (dd, J = 17.5, 7.8 Hz, 1H), 8.05 – 7.93 (m, 2H), 7.82 (dtd, J = 13.4, 7.7, 1.6 Hz, 1H), 7.64 – 7.40 (m, 5H), 5.84 (t, J = 16.1 Hz, 1H), 5.13 – 5.00 (m, 1H), 4.16 (dq, J = 25.4, 7.1 Hz, 2H), 3.75 (t, J = 5.7 Hz, 3H), 3.30 (ddd, J = 22.1, 15.0, 7.1 Hz, 1H), 3.08 (dd, J = 14.4, 10.4 Hz, 1H), 2.42 – 2.17 (m, 2H), 1.30 (t, J = 7.1 Hz, 1H), 1.22 (t, J = 7.1 Hz, 2H), 0.97 (t, J = 7.5 Hz, 2H), 0.86 (d, J = 4.5 Hz, 1H). ^{13}C NMR (101 MHz, $CDCl_3$) δ = 172.0, 171.7, 167.3, 167.1, 166.5, 166.2, 164.7, 148.9, 148.5, 139.85, 137.55, 137.35, 136.9, 136.09, 135.8, 135.3, 134.1, 133.8, 132.1, 128.9, 127.6, 126.7, 122.5, 122.2, 119.6, 119.4, 60.5, 52.9, 51.1, 50.8, 35.08, 33.7, 21.1, 14.4, 13.6, 12.4. HR-MS (ESI) m/z calcd for $C_{26}H_{30}N_3O_6$ [M+H$^+$]: 480.2129, found: 480.2134.

vi) Sonogashira cross-coupling reaction of compound **68**

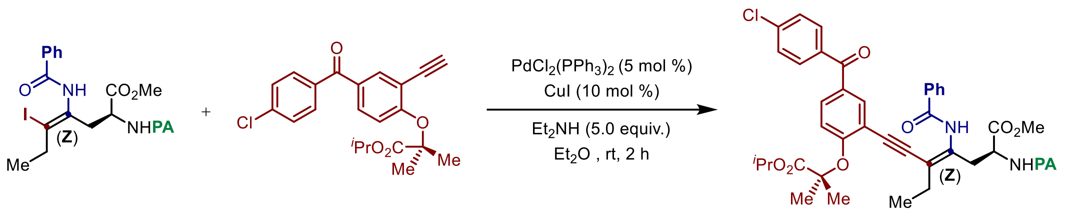

Figure S33. Sonogashira cross-coupling reaction of compound **68**.

Compound **73** was prepared according to literature procedures.[18] Purification by column chromatography (petroleum ether/EtOAc = 20:1) yielded **73** as a yellow solid. ^{1}H NMR (400 MHz, $CDCl_3$) δ = 7.91 (d, J = 2.2 Hz, 1H), 7.70 (dd, J = 8.6, 1.8 Hz, 3H), 7.46 (d, J = 8.6 Hz, 2H), 6.79 (d, J = 8.7 Hz, 1H), 5.10 (dt, J = 12.5, 6.3 Hz, 1H), 3.27 (s, 1H), 1.70 (s, 6H), 1.22 (d, J = 6.3 Hz, 6H). ^{13}C NMR (101 MHz, $CDCl_3$) δ = 193.6, 173.0, 160.8, 138.8, 136.6, 136.0, 131.6, 131.3, 130.3, 128.8, 115.88, 114.4, 82.1, 80.8, 79.3, 69.6, 25.4, 21.7. HR-MS (ESI) m/z calcd for $C_{22}H_{22}ClO_4$ [M+H$^+$]: 385.1201, found: 385.1205.

The sonogashira cross-coupling reaction of compound **68** was performed following a modified literature procedure,[3] to a solution of the **68** (40.6 mg, 0.08 mmol, 1.0 equiv.), **73** (36.8 mg, 0.096 mmol, 1.2 equiv.) and Et_2NH (29.3 mg, 0.4 mmol. 5 equiv.) in Et_2O (1 mL), CuI (10 mol %) and the $PdCl_2(PPh_3)_2$ (2.8 mg, 0.004 mmol, 5 mol %) were added. The mixture was stirred at room temperature under argon for 2 h. The reaction mixture was poured into a saturated aqueous solution of NH_4Cl. After phase separation, the aqueous layer was extracted with Et_2O (10 mL × 2). The combined organic layers were washed with brine, dried over Na_2SO_4, filtered, and concentrated in vacuo. The crude product was purified by column chromatography (petroleum ether/EtOAc = 2:1) on silica gel to afford the desired product **74** (43.4 mg, 71%) as a yellow oil. ^{1}H NMR (400 MHz, $CDCl_3$) δ = 8.84 (d, J = 8.6 Hz, 1H), 8.49 (s, 1H), 8.45 (d, J = 4.6 Hz, 1H), 8.14 (d, J = 7.8 Hz, 1H), 7.91 – 7.84 (m, 2H), 7.80 (td, J = 7.7, 1.6 Hz, 1H), 7.76 (d, J = 2.2 Hz, 1H), 7.67 (d, J = 8.5 Hz, 2H), 7.62 (dd, J = 8.7, 2.2 Hz, 1H), 7.47 (t, J = 7.4 Hz, 1H), 7.45 – 7.30 (m, 5H), 6.72 (d, J = 8.7 Hz, 1H), 5.12 – 4.97 (m, 2H), 3.74 (d, J

= 4.0 Hz, 3H), 3.65 (d, *J* = 7.2 Hz, 2H), 2.38 (q, *J* = 7.4 Hz, 2H), 1.47 (d, *J* = 5.0 Hz, 6H), 1.23 – 1.12 (m, 9H). ^{13}C NMR (101 MHz, $CDCl_3$) δ = 193.5, 172.8, 172.0, 165.70 (s), 164.5, 159.8, 149.4, 148.3, 138.9, 137.9, 137.3, 136.0, 135.1, 134.7, 132.1, 131.3, 130.3, 128.8, 127.4, 126.4, 122.4, 115.5, 115.1, 113.3, 94.4, 91.2, 80.6, 69.6, 52.8, 51.8, 30.5, 25.2, 23.6, 21.6, 13.7. HR-MS (ESI) m/z calcd for $C_{43}H_{43}ClN_3O_8$ [M+H$^+$]: 764.2733, found: 764.2729.

b) Peptide modification enabled by thioglycosylation

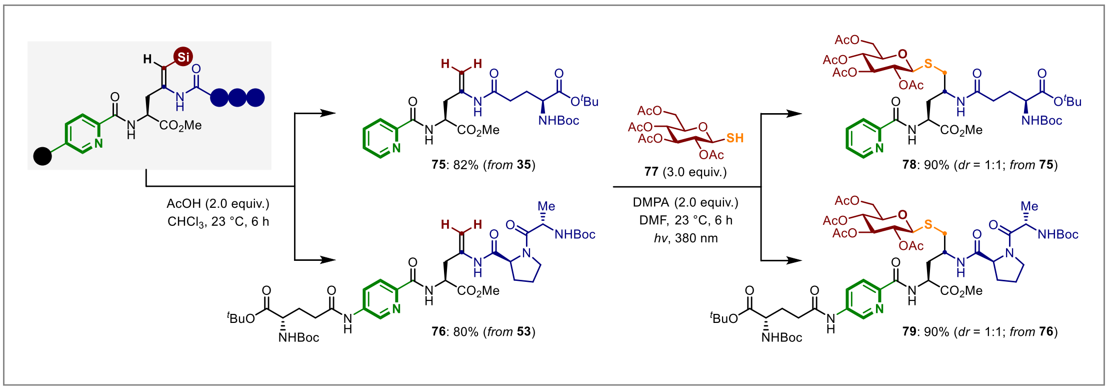

Figure S34. Peptide modification enabled by thioglycosylation.

i) Protodesilylation of compound **35**

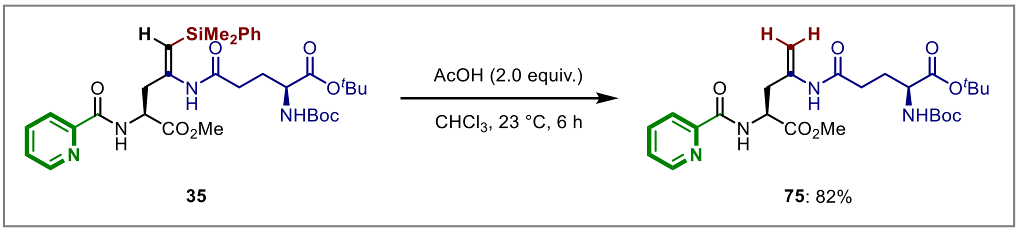

Figure S35. Protodesilylation of compound **35**.

To a 25 mL round-bottom flask were added **35** (196.1 mg, 0.3 mmol, 1.0 equiv.) and $CHCl_3$ (3 mL). The resulting solution was stirred at room temperature. AcOH (36.3 mg, 0.6 mmol, 2.0 equiv.) was added and the reaction was stirred at room temperature for 6 h. Upon completion, the reaction mixture was concentrated directly. The crude product was purified by column chromatography (petroleum ether/EtOAc = 1.5:1) on silica gel to afford the desired product **75** (132 mg, 82%) as a colorless oil. ^1H NMR (400 MHz, $CDCl_3$) δ = 8.69 (d, J = 7.1 Hz, 1H), 8.59 (d, J = 4.2 Hz, 1H), 8.27 (s, 1H), 8.16 (d, J = 7.8 Hz, 1H), 7.85 (dd, J = 9.0, 5.0 Hz, 1H), 7.46 (dd, J = 6.9, 5.3 Hz, 1H), 5.63 (s, 1H), 5.35 (d, J = 7.6 Hz, 1H), 4.92 (d, J = 6.5 Hz, 1H), 4.60 (s, 1H), 4.19 (s, 1H), 3.79 (s, 3H), 2.88 (dd, J = 14.4, 5.4 Hz, 1H), 2.67 (dd, J = 14.4, 6.7 Hz, 1H), 2.36 (t, J = 7.1 Hz, 2H), 2.23 (dd, J = 8.4, 5.4 Hz, 1H), 1.94 – 1.84 (m, 1H), 1.45 (d, J = 7.0 Hz, 18H). ^{13}C NMR (101 MHz, $CDCl_3$) δ = 172.1, 171.6, 164.6, 156.2, 148.5, 137.5, 137.2, 126.7,

122.5, 102.7, 82.4, 80.2, 53.6, 52.8, 51.3, 39.4, 33.9, 29.5, 28.4, 28.1. HR-MS (ESI) m/z calcd for $C_{26}H_{39}N_4O_8$ [M+H$^+$]: 535.2762, found: 535.2766.

Compound **76** was synthesized following the protodesilylation procedure of compound **35**. **53** (143 mg, 0.15 mmol, 1.0 equiv.), AcOH (18.0 mg, 0.3 mmol, 2.0 equiv.) and $CHCl_3$ (1.5 mL) for 6 h. Purification by column chromatography (petroleum ether/EtOAc = 1:4) yielded **76** (98 mg, 80%) as a white solid. ^{1}H NMR (400 MHz, $CDCl_3$) δ = 9.78 (s, 1H), 8.86 (s, 1H), 8.74 (s, 1H), 8.45 (d, J = 7.8 Hz, 1H), 8.28 (d, J = 7.8 Hz, 1H), 8.07 (d, J = 8.4 Hz, 1H), 5.51 (d, J = 9.2 Hz, 3H), 4.83 (d, J = 6.2 Hz, 1H), 4.59 (d, J = 10.4 Hz, 2H), 4.50 (dd, J = 14.5, 7.1 Hz, 1H), 4.19 (s, 1H), 3.76 (s, 3H), 3.71 – 3.58 (m, 2H), 2.84 (dt, J = 9.5, 4.8 Hz, 1H), 2.59 (dd, J = 14.2, 7.9 Hz, 1H), 2.50 (d, J = 5.8 Hz, 2H), 2.35 – 2.14 (m, 4H), 1.98 – 1.88 (m, 2H), 1.46 (t, J = 10.3 Hz, 27H), 1.32 (d, J = 6.9 Hz, 3H). ^{13}C NMR (101 MHz, $CDCl_3$) δ = 173.4, 172.1, 171.6, 171.2, 170.0, 164.3, 157.0, 155.4, 144.0, 139.6, 138.3, 137.0, 126.8, 122.92 (s), 103.1, 83.1, 81.0, 79.8, 60.9, 53.3, 52.7, 51.0, 48.0, 47.5, 38.9, 34.2, 28.5, 28.1, 25.2, 22.8, 18.3. HR-MS (ESI) m/z calcd for $C_{39}H_{60}N_7O_{12}$ [M+H$^+$]: 818.4294, found: 818.4300.

ii) Thiol-ene reaction of compound **75** with thiosugar **77**

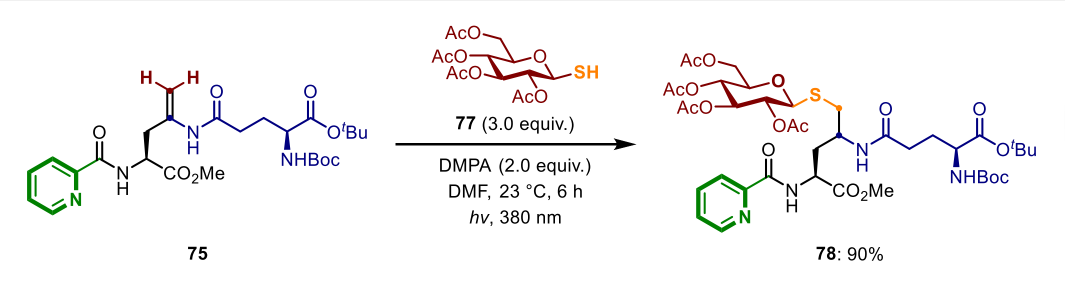

Figure S36. Thiol-ene reaction of compound **75** with thiosugar **77**.

Following a modified literature procedure,[19] in a nitrogen-filled glovebox, dehydropeptide **75** (53.5 mg, 0.1 mmol, 1.0 equiv.), 1-thio-*β-D* glucose tetraacetate **77** (109.3 mg, 0.3 mmol, 3.0 equiv.), 2,2 dimethoxy-2-phenylacetophenone (DMPA, 51.3 mg, 0.2 mmol, 2.0 equiv.) and degassed DMF (1.0 mL) were added to an oven-dried 10-mL scintillation vial equipped with a Teflon-coated magnetic stir bar. The vial was

sealed with a screw-top septum cap and removed from the glovebox. The vial was placed 5 cm from a UV LED Kessil lamp (380 nm, 50 w) and stirred at 1000 rpm with cooling fans for 6 h. Upon completion, the mixture was quenched with sat. aq. NaCl (10 mL) The aqueous layer was extracted with EtOAc (15 mL × 3). The combined organic layers were washed with brine, dried over anhydrous Na_2SO_4, filtered, and concentrated under reduced pressure. The crude product was purified by column chromatography (petroleum ether/EtOAc = 1:2) on silica gel to afford the desired product **78** (81.0 mg, 90%) as a white solid and as a mixture of two inseparable diastereomers (*dr* = 1:1). ^{1}H NMR (400 MHz, $CDCl_3$) δ = 8.85 (d, *J* = 7.9 Hz, 1H), 8.63 (dd, *J* = 11.6, 3.9 Hz, 1H), 8.16 (t, *J* = 7.2 Hz, 1H), 7.86 (t, *J* = 7.3 Hz, 1H), 7.55 – 7.42 (m, 1H), 6.76 (dd, *J* = 55.6, 7.1 Hz, 1H), 5.41 – 5.25 (m, 1H), 5.21 (dd, *J* = 17.3, 9.1 Hz, 1H), 5.07 (td, *J* = 9.8, 2.3 Hz, 1H), 4.98 (td, *J* = 9.8, 2.5 Hz, 1H), 4.81 (s, 1H), 4.57 (dd, *J* = 10.0, 4.3 Hz, 1H), 4.26 (dd, *J* = 12.3, 4.7 Hz, 1H), 4.13 (m, 3H), 3.83 – 3.63 (m, 4H), 2.93 (m, 2H), 2.30 (t, *J* = 7.5 Hz, 2H), 2.19 – 1.98 (m, 16H), 1.46 (d, *J* = 12.1 Hz, 18H). ^{13}C NMR (101 MHz, $CDCl_3$) δ = 172.3, 172.1, 172.0, 171.5, 170.8, 170.2, 169.6, 164.6, 155.9, 149.29, 148.6, 137.5, 126.7, 122.5, 84.4, 83.5, 82.25, 78.0, 76.1, 73.9, 70.0, 68.2, 62.0, 53.7, 52.8, 49.8, 47.2, 35.1, 32.7, 29.0, 28.4, 28.1, 20.8. HR-MS (ESI) m/z calcd for $C_{40}H_{59}N_4O_{17}S$ [M+H$^+$]: 899.3590, found: 899.3593.

Compound **79** was synthesized following the thiol-ene reaction of compound **75** with thiosugar **77**. **76** (70.0 mg, 0.085 mmol, 1.0 equiv.), 1-thio-*β-D* glucose tetraacetate **77** (93.0 mg, 0.255 mmol, 3.0 equiv.), 2,2 dimethoxy-2-phenylacetophenone (DMPA, 43.6 mg, 0.17 mmol, 2.0 equiv.) and degassed DMF (1.0 mL) for 6 h. Purification by column chromatography (EtOAc) yielded **79** (90.0 mg, 90%) as a white solid and as a mixture of two inseparable diastereomers (*dr* = 1:1). ^{1}H NMR (400 MHz, $CDCl_3$) δ = 9.72 (s, 1H), 8.66 (m, 2H), 8.38 – 8.23 (m, 1H), 8.06 (dd, *J* = 17.7, 8.4 Hz, 1H), 7.48 (d, *J* = 7.5 Hz, 1H), 7.27 (d, *J* = 7.9 Hz, 1H), 5.56 – 5.23 (m, 3H), 5.15 – 5.07 (m, 1H), 5.02 – 4.92 (m, 1H), 4.87 – 4.46 (m, 4H), 4.34 – 4.16 (m, 3H), 4.11 (d, *J* = 11.9 Hz, 1H), 4.05 – 3.89 (m, 1H), 3.71 (m, 5H), 3.16 – 3.01 (m, 1H), 2.70 (m, 1H), 2.51 (s, 2H), 2.24 (d, *J* = 19.4 Hz, 4H), 2.10 – 1.93 (m, 16H), 1.46 (t, *J* = 10.2 Hz, 27H), 1.36 (d, *J* = 15.3 Hz,

3H). ^{13}C NMR (101 MHz, $CDCl_3$) δ = 173.5, 172.7, 172.4, 172.2, 171.5, 171.1, 170.9, 170.2, 169.7, 169.6, 169.5, 164.3, 157.0, 155.3, 143.9, 139.7, 138.2, 126.8, 122.9, 84.4, 83.1, 80.9, 79.7, 75.8, 73.9, 70.4, 70.1, 68.5, 68.4, 62.2, 61.9, 60.3, 53.0, 52.7, 49.6, 47.9, 47.3, 47.0, 36.5, 35.3, 34.2, 30.8, 28.4, 28.0, 27.3, 25.1, 20.8, 20.7, 20.6, 18.6, 18.3. HR-MS (ESI) m/z calcd for $C_{53}H_{80}N_7O_{21}S$ [M+H$^+$]: 1182 5122, found: 1182 5120.

c) Removal of PA-directing group

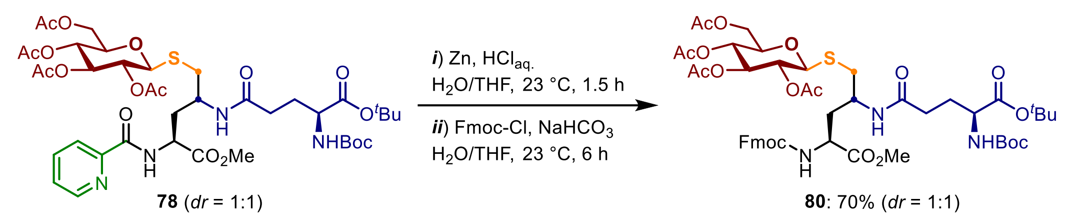

Figure S37. Removal of PA-directing group.

Following a modified literature procedure,[20] To a stirred solution of **78** (45.0 mg, 0.05 mmol, 1.0 equiv.) in THF/H_2O (2:1, 0.75 mL) at 23 °C was added aqueous HCl (1.5 M, 0.5 mL) over 3 min, then zinc powder (49.0 mg, 0.75 mmol, 15.0 equiv.) was added. The mixture was stirred for 1.5 h at 23 °C. Afterward, $NaHCO_3$ was added to adjust the pH to 7~8, and Fmoc-Cl (77.6 mg, 0.15 mmol, 6.0 equiv.) was added susequently. The reaction mixture was stirred for 6 h and quenched with water. The mixture was extracted with EA (3 × 5 mL). The combined organic layers were washed with brine, dried over anhydrous Na_2SO_4, and concentrated in vacuo. The crude product was purified by column chromatography (petroleum ether/EtOAc = 1:1) on silica gel to afford the desired product **80** (35.6 mg, 70%) as a white solid and as a mixture of two inseparable diastereomers (*dr* = 1:1). ^{1}H NMR (400 MHz, $CDCl_3$) δ = ^{1}H NMR (400 MHz, $CDCl_3$) δ = 7.76 (d, *J* = 7.5 Hz, 2H), 7.64 (dd, *J* = 13.5, 6.9 Hz, 2H), 7.40 (t, *J* = 7.5 Hz, 2H), 7.32 (t, *J* = 7.4 Hz, 2H), 6.50 (s, 0.77 H), 6.17 (d, *J* = 8.7 Hz, 1H), 5.88 (s, 0.33H), 5.36 – 5.14 (m, 2H), 5.12 – 4.95 (m, 2H), 4.55 – 4.02 (m, 9H), 3.74 (d, *J* = 6.0 Hz, 4H), 3.11 – 2.57 (m, 2H), 2.36 – 2.12 (m, 4H), 2.10 – 1.98 (m, 13H), 1.87 (d, *J* = 6.6 Hz, 1H), 1.45 (d, *J* = 13.8 Hz, 18H). ^{13}C NMR (101 MHz, $CDCl_3$) δ = 172.5, 171.4,

170.9, 170.8, 170.2, 170.2, 169.8, 169.7, 169.6, 169.5, 156.4, 144.0, 141.4, 127.8, 127.3, 125.4, 120.1, 84.0, 83.5, 82.5, 82.4, 80.1, 76.4, 76.2, 73.8, 70.1, 70.0, 68.3, 68.2, 67.4, 67.2, 62.1, 61.9, 53.6, 52.7, 51.5, 47.3, 46.3, 350, 34.8, 32.6, 28.5, 28.1, 28.1, 27.4, 27.1, 20.9, 20.7. HR-MS (ESI) m/z calcd for $C_{49}H_{66}N_3O_{18}S$ [M+H$^+$]: 1016.4057, found: 1016.4034.

X-Ray Crystallography Data

Experimental Summary for Crystal of 65

Single crystals suitable for X-ray diffraction experiment were obtained by vapor diffusion of hexanes into a saturated solution of **65** in DCM (CCDC 2515766).

Table S3. Details for X-ray data collection and structure refinement for compound **65**.

Empirical formula	$C_{8.31}H_{7.39}Co_{0.31}N_{1.23}O_{2.47}$
Formula weight	182.16
Temperature [K]	220.0(2)
Crystal system	monoclinic
Space group (number)	$P2_1/c$ (14)
a [Å]	10.6101(11)
b [Å]	12.6819(18)
c [Å]	21.670(3)
α [°]	90
β [°]	90.686(12)
γ [°]	90
Volume [Å^3]	2915.6(7)
Z	13
ρ_{calc} [gcm^{-3}]	1.349
μ [mm^{-1}]	0.642
F(000)	1221
Crystal size [mm^3]	0.35×0.2×0.1
Crystal colour	clear light green
Crystal shape	block
Radiation	Mo K_α (λ=0.71073 Å)
2θ range [°]	3.72 to 61.08 (0.70 Å)

Index ranges	$-14 \le h \le 15$<br>$-15 \le k \le 18$<br>$-18 \le l \le 30$
Reflections collected	13666
Independent reflections	7391<br>R_{int} = 0.0587<br>R_{sigma} = 0.1323
Completeness to θ = 25.242°	98.5 %
Data / Restraints / Parameters	7391/0/318
Absorption correction<br>T_{min}/T_{max} (method)	0.80920/1.00000<br>(multi-scan)
Goodness-of-fit on F^2	0.876
Final R indexes<br>[$I \geq 2\sigma(I)$]	R_1 = 0.0662<br>wR_2 = 0.1457
Final R indexes<br>[all data]	R_1 = 0.1477<br>wR_2 = 0.1934
Largest peak/hole [eÅ^{-3}]	0.35/−0.46

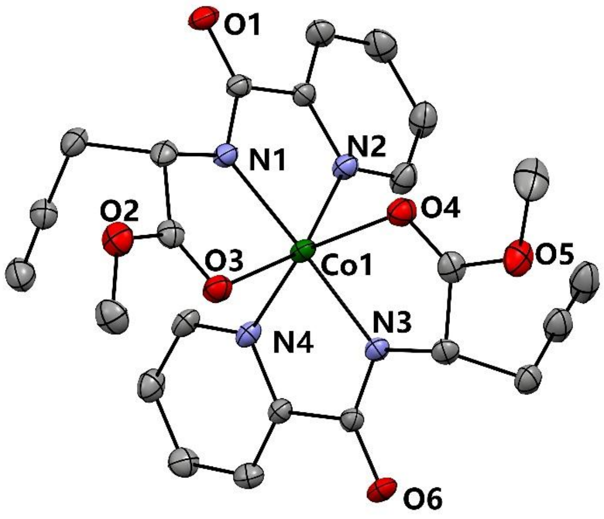

Figure S38. ORTEP plots for molecular structures of **65** with the probability at 50% Level.

References

[1] Lamartina, C. W.; Chartier, C. A.; Lee, S.; Shah, N. H.; Rovis, T. Modular Synthesis of Unnatural Peptides via Rh(III)-Catalyzed Diastereoselective Three-Component Carboamidation Reaction. *J. Am. Chem. Soc.* **2023**, *145*, 1129-1135.

[2] Ijsselstijn, M.; Kaiser, J.; van Delft, F. L.; Schoemaker, H. E.; Rutjes, F. Synthesis of Novel Acetylene-Containing Amino Acids. *Amino Acids* **2003**, *24*, 263-266.

[3] van Esseveldt, B. C. J.; Vervoort, P. W. H.; van Delft, F. L.; Rutjes, F. P. J. T.; Novel Approach to 5-Substituted Proline Derivatives Using a Silver-Catalyzed Cyclization as the Key Step. *J. Org. Chem.* **2005**, *70*, 1791-1795.

[4] Mattsson, S.; Dahlstrom, M.; Karlsson, S. A Mild Hydrolysis of Esters Mediated by Lithium Salts. *Tetrahedron Lett.* **2007**, *48*, 2497-2499.

[5] Kobayashi, T.; Yasuno, T.; Takahashi, K.; Nakamura, S.; Mashino, T.; Ohe, T. Novel Pyridinium-Type Fullerene Derivatives as Multitargeting Inhibitors of HIV-1 Reverse Transcriptase, HIV-1 Protease, And HCV NS5B Polymerase. *Bioorg. Med. Chem. Lett.* **2021**, *49*, 128267-128272.

[6] Rovis, T.; Lei, H.; Ir-Catalyzed Intermolecular Branch-Selective Allylic C−H Amidation of Unactivated Terminal Olefins. *J. Am. Chem. Soc.* **2019**, *141*, 2268-2273.

[7] Wang, H.; Jung, H.; Song, F.; Zhu, S.; Bai, Z.; Chen, D.; He, G.; Chang, S.; Chen, G. Nitrene-Mediated Intermolecular N−N Coupling for Efficient Synthesis of Hydrazides. *Nat. Chem.* **2021**, *13*, 378-385.

[8] Knecht, T.; Mondal, S.; Ye, J.-H.; Das, M.; Glorius, F. Intermolecular, Branch-Selective, and Redox-Neutral Cp*Ir^{III}-Catalyzed Allylic C−H Amidation. *Angew. Chem., Int. Ed.* **2019**, *58*, 7117-7121.

[9] Hong, S. Y.; Park, Y.; Hwang, Y.; Kim, Y. B.; Baik, M.-H.; Chang, S. Selective Formation of *γ*-Lactams via C−H Amidation Enabled by Tailored Iridium Catalysts. *Science* **2018**, *359*, 1016-1021.

[10] Du, B.; Ouyang, Y.; Chen, Q.; Yu, W.-Y. Thioether-Directed NiH-Catalyzed Remote *γ*-C(sp^3)−H Hydroamidation of Alkenes by 1,4,2-Dioxazol-5-ones. *J. Am. Chem. Soc.* **2021**, *143*, 14962-14968.

[11] Kofron, W. G.; Baclawski, L. M. Convenient Method for Estimation of Alkyllithium Concentrations. *J. Org. Chem.* **1976**, *41*, 1879-1880.

[12] Krasovskiy, A.; Knochel, P. Convenient Titration Method for Organometallic Zinc, Magnesium, and Lanthanide Reagents. *Synthesis* **2006**, *2006*, 890-891.

[13] Azuma, H.; Okano, K.; Tokuyama, H. Synthesis of Acylsilanes by Palladium-Catalyzed Cross-Coupling Reaction of Thiol Esters and Silylzinc Chlorides. *Chem. Lett.* **2011**, *40*, 959-961.

[14] Perrone, S.; Knochel, P. Highly Diastereoselective Preparation of (*E*)-Alkenylsilanes Bearing an *α*-Chiral Center. *Org. Lett.* **2007**, *9*, 1041-1044.

[15] Herrmann, A. T.; Martinez, S. R.; Zakarian, A. A Concise Asymmetric Total Synthesis of (+)-Brevisamide. *Org. Lett.* **2011**, *13*, 3636-3639.

[16] Hong, S. Y.; Son, J.; Kim, D.; Chang, S. Ir(III)-Catalyzed Stereoselective Haloamidation of Alkynes Enabled by Ligand Participation. *J. Am. Chem. Soc.* **2018**, *140*, 12359-12363.

[17] Shindo, M.; Matsumoto, K.; Mori, S.; Shishido, K. The First General Method for *Z*-Selective Olefination of Acylsilanes via Ynolate Anions Providing Multisubstituted Alkenes. *J. Am. Chem. Soc.* **2002**, *124*, 6840-6841.

[18] Kanemoto, K.; Yoshimura, K.; Ono, K.; Ding, W.; Ito, S.; Yoshikai, N. Amino- and Alkoxybenziodoxoles: Facile Preparation and Use as Arynophiles. *Chem. Eur. J.* ***2024**, 30,* e202400894.

[19] Gu, X.; Zhang, Y.-A.; Zhang, S.; Wang, L.; Ye, X.; Occhialini, G.; Barbour, J.; Pentelute, B. L.; Wendlandt, A. E. Synthesis of Non-Canonical Amino Acids Through Dehydrogenative Tayloring. *Nature* **2024**, *634*, 352-358.

[20] Li, B.; Li, X.; Han, B.; Chen, Z.; Zhang, X.; He, G.; Chen, G. Construction of Natural-Product-Like Cyclophane-Braced Peptide Macrocycles via sp^3C−H Arylation. *J. Am. Chem. Soc.* **2019**, *141*, 9401−9407.

NMR Spectra

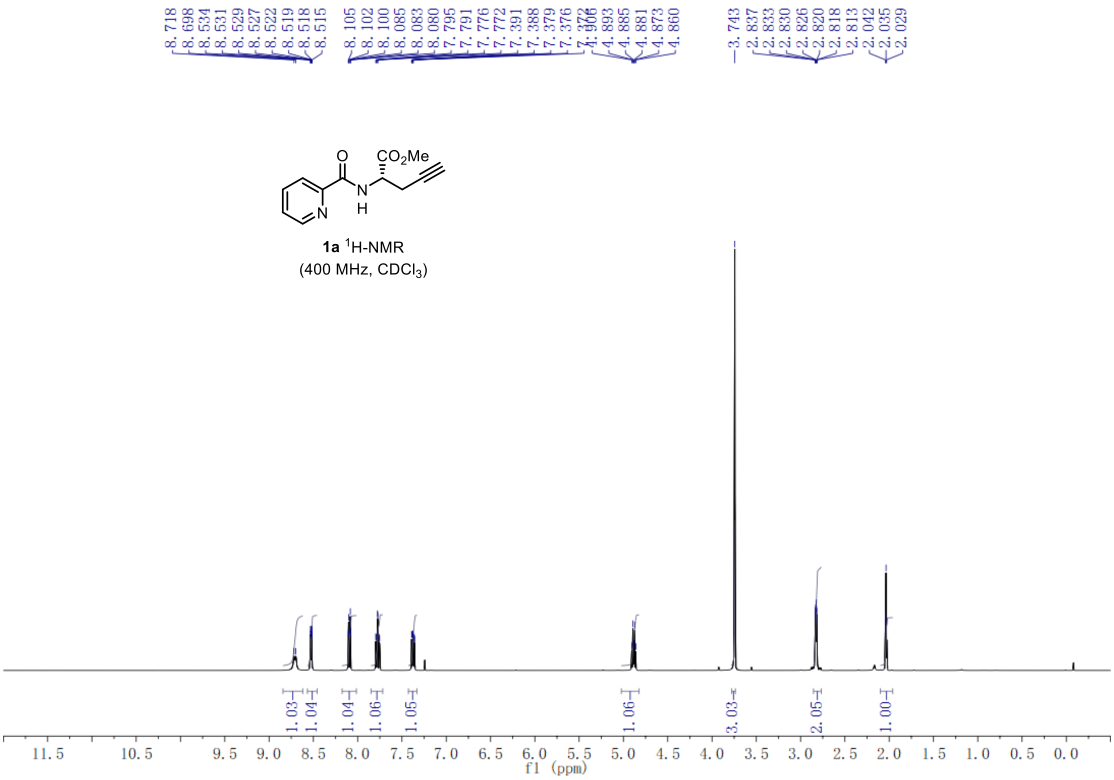
8.718
8.698
8.534
8.531
8.529
8.527
8.522
8.519
8.518
8.515
8.105
8.102
8.100
8.085
8.083
8.080
7.795
7.791
7.776
7.772
7.391
7.388
7.379
7.376
7.372
4.906
4.893
4.885
4.881
4.873
4.860
3.743
2.837
2.833
2.830
2.826
2.820
2.818
2.813
2.042
2.035
2.029
O
CO_2Me
N
H
N
1a ^{1}H-NMR
(400 MHz, $CDCl_3$)
1.03
1.04
1.04
1.06
1.05
1.06
3.03
2.05
1.00
11.5 10.5 9.5 9.0 8.5 8.0 7.5 7.0 6.5 6.0 5.5 5.0 4.5 4.0 3.5 3.0 2.5 2.0 1.5 1.0 0.5 0.0
f1 (ppm)

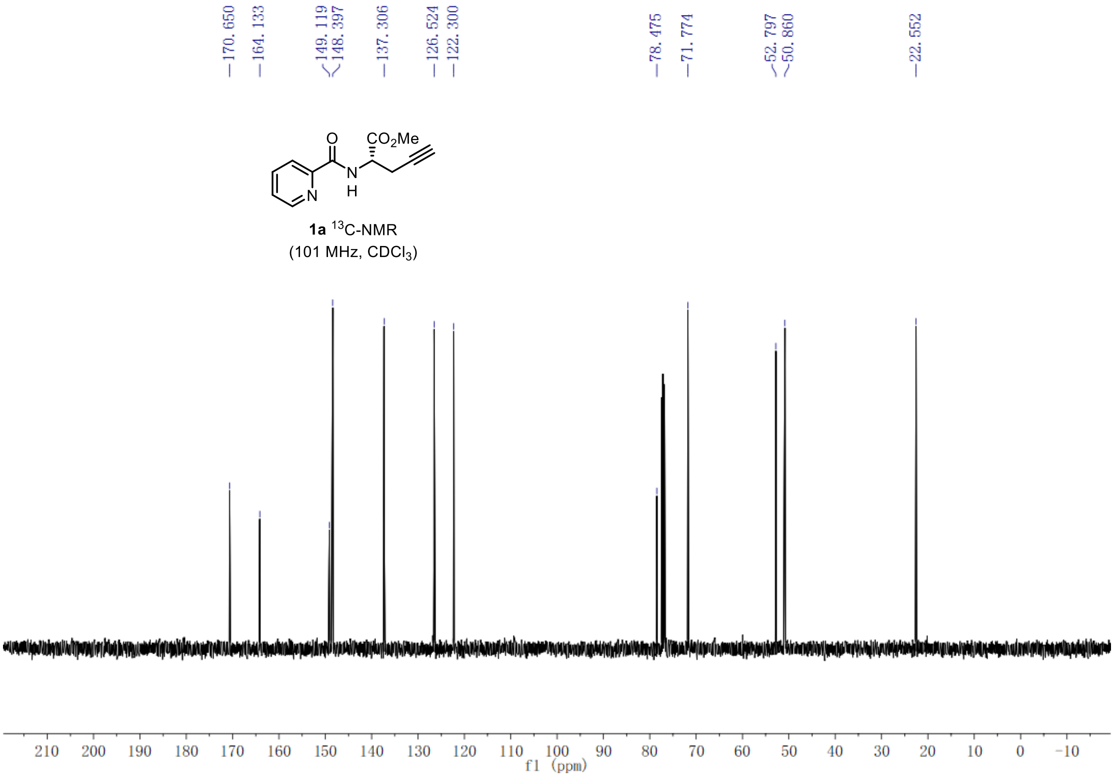
170.650
164.133
149.119
148.397
137.306
126.524
122.300
78.475
71.774
52.797
50.860
22.552
O
CO_2Me
N
H
N
1a ^{13}C-NMR
(101 MHz, $CDCl_3$)
210 200 190 180 170 160 150 140 130 120 110 100 90 80 70 60 50 40 30 20 10 0 −10
f1 (ppm)

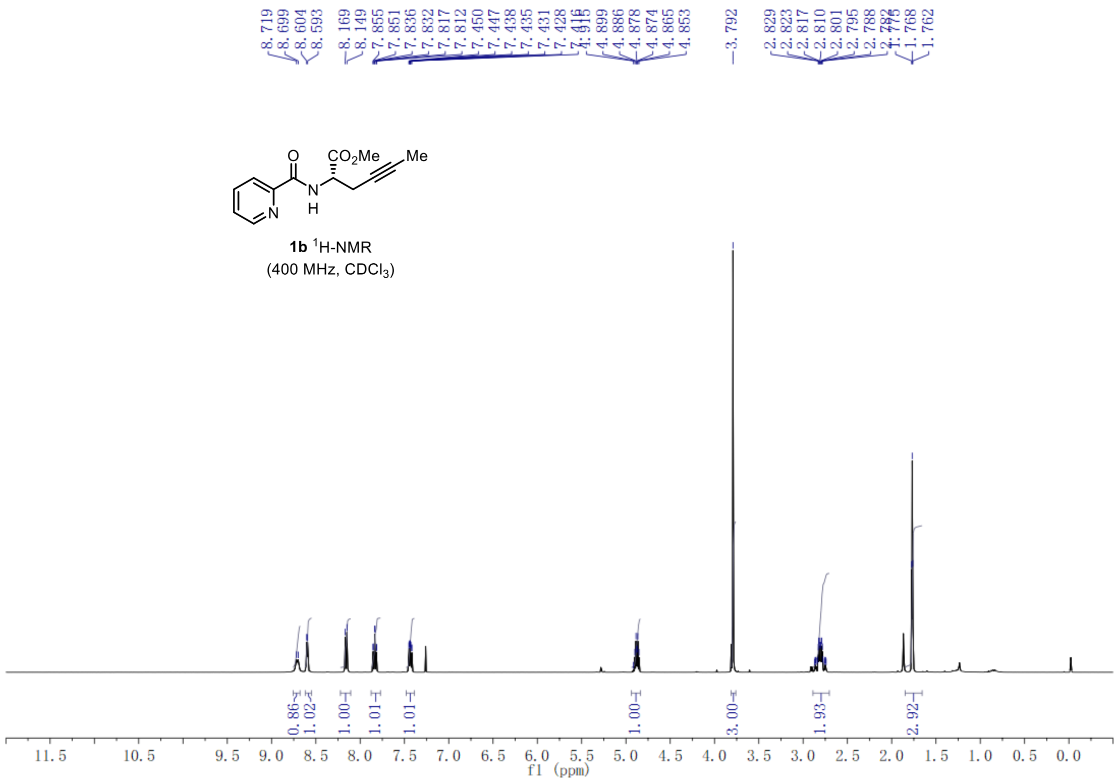

1b ¹H-NMR
(400 MHz, CDCl3)

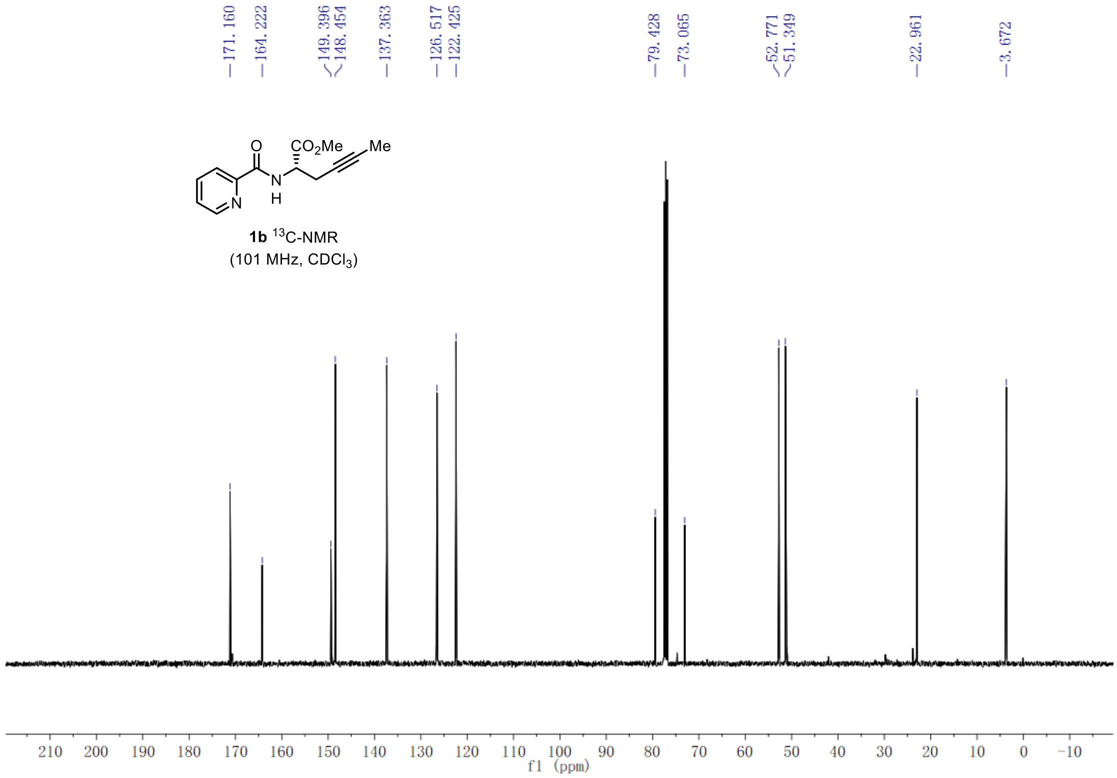

1b ¹³C-NMR
(101 MHz, CDCl3)

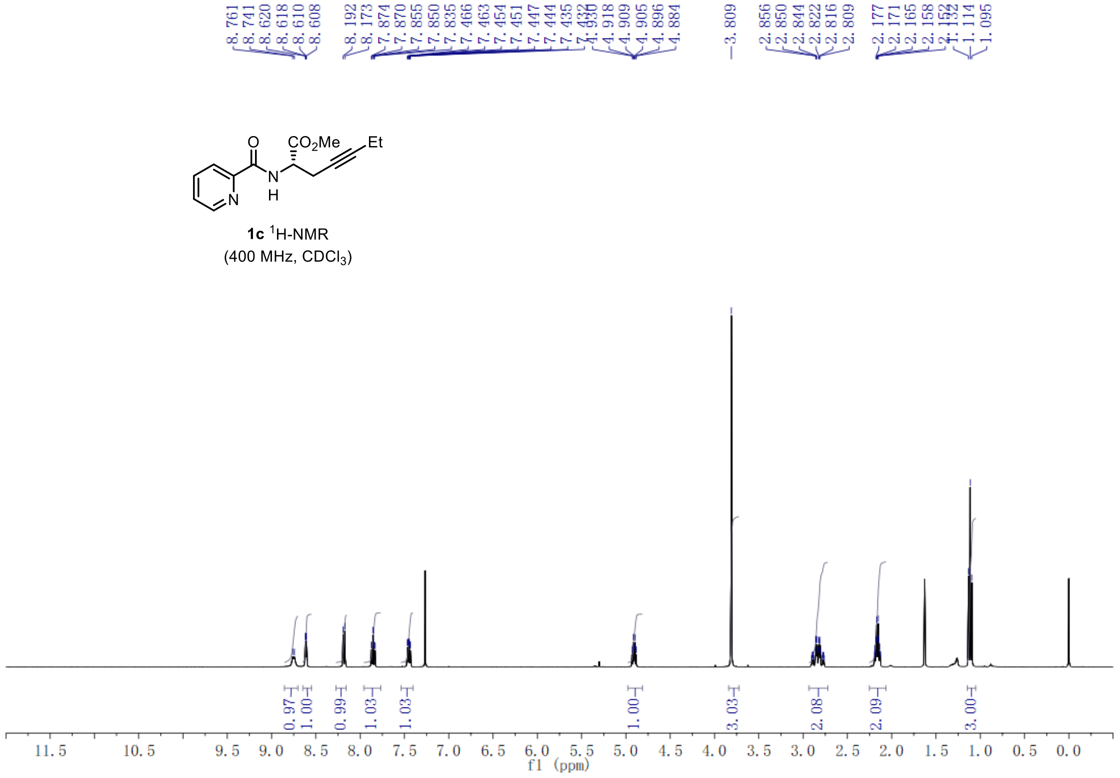
8.761
8.741
8.620
8.618
8.610
8.608
8.192
8.173
7.874
7.870
7.855
7.850
7.835
7.466
7.463
7.454
7.451
7.447
7.444
7.435
4.918
4.909
4.905
4.896
4.884
3.809
2.856
2.850
2.844
2.822
2.816
2.809
2.177
2.171
2.165
2.158
1.114
1.095
O
CO2Me
Et
N
H
N
1c 1H-NMR
(400 MHz, CDCl3)
0.97
1.00
0.99
1.03
1.03
1.00
3.03
2.08
2.09
3.00
f1 (ppm)

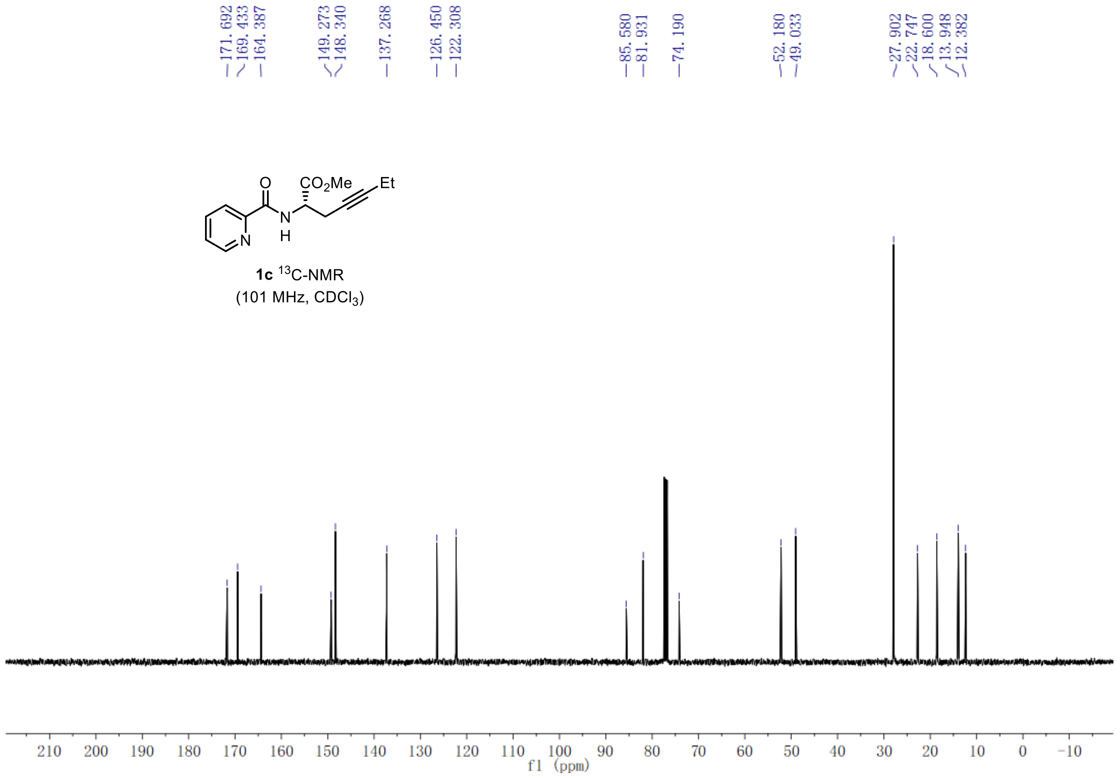
171.692
169.433
164.387
149.273
148.340
137.268
126.450
122.308
85.580
81.931
74.190
52.180
49.033
27.902
22.747
18.600
13.948
12.382
O
CO2Me
Et
N
H
N
1c 13C-NMR
(101 MHz, CDCl3)
f1 (ppm)

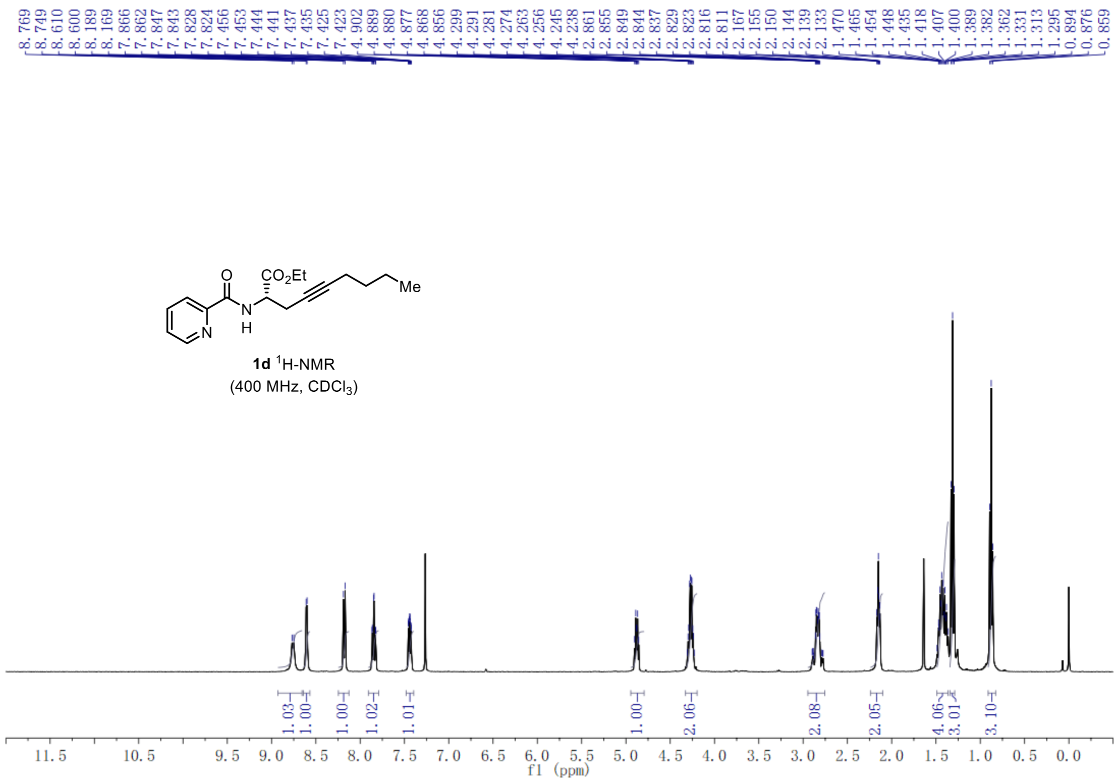
1d ¹H-NMR
(400 MHz, CDCl3)
f1 (ppm)

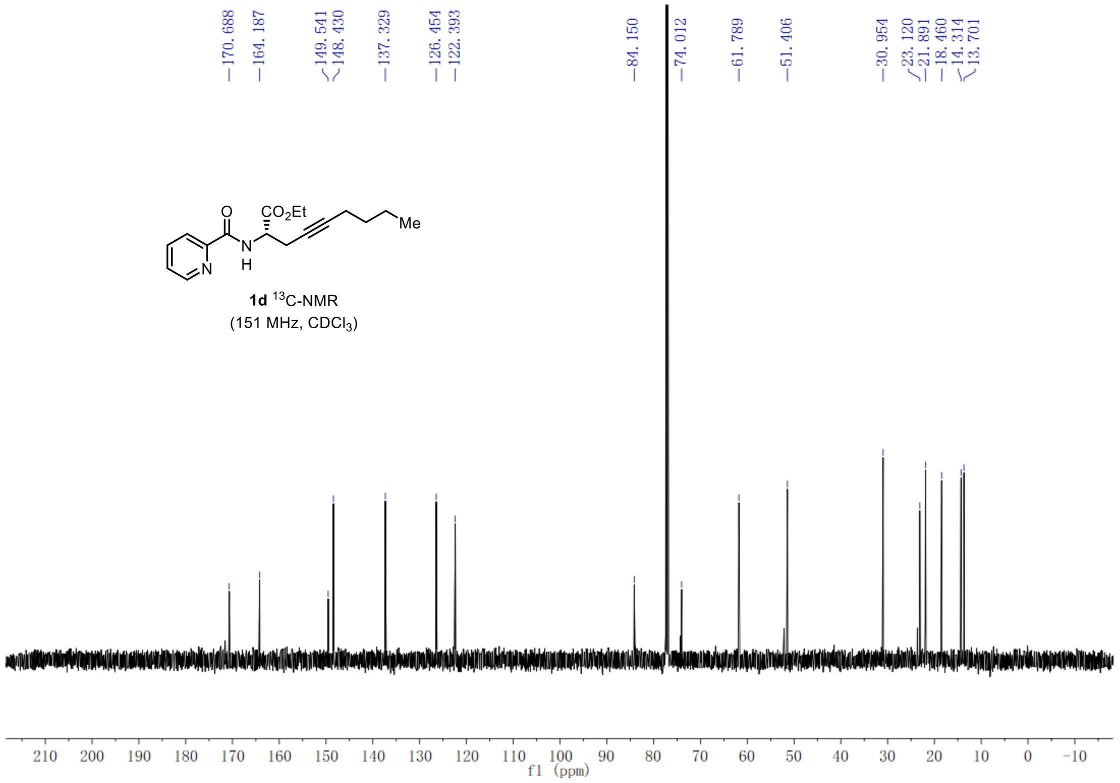
1d ¹³C-NMR
(151 MHz, CDCl3)
f1 (ppm)

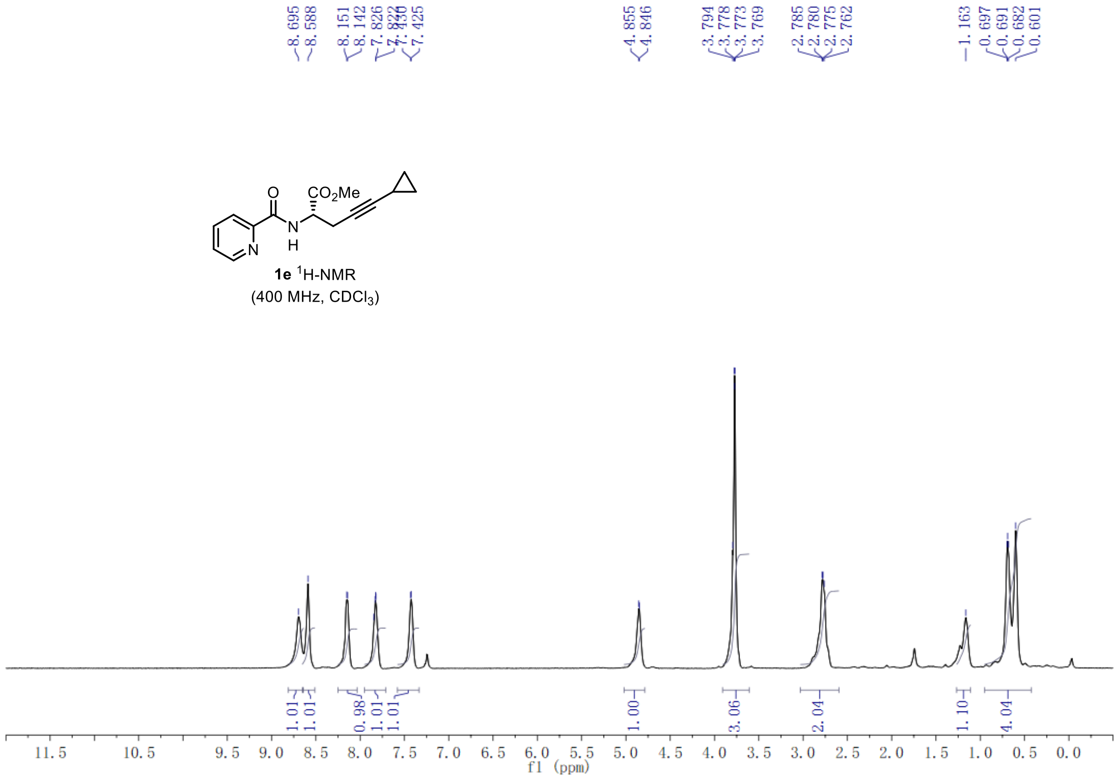

8.695
8.588
8.151
8.142
7.826
7.822
7.430
7.425
4.855
4.846
3.794
3.778
3.773
3.769
2.785
2.780
2.775
2.762
1.163
0.697
0.691
0.682
0.601
O
CO2Me
N
H
N
1e 1H-NMR
(400 MHz, CDCl3)
1.01
1.01
0.98
1.01
1.01
1.00
3.06
2.04
1.10
4.04
f1 (ppm)

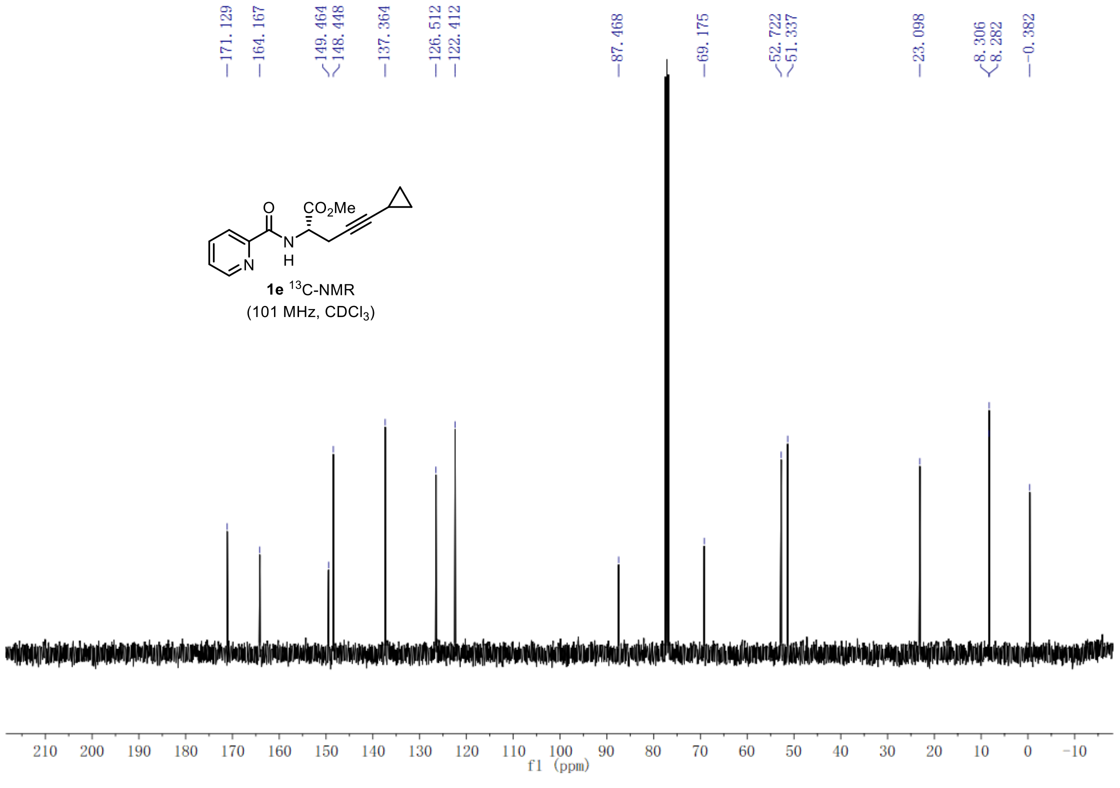

171.129
164.167
149.464
148.448
137.364
126.512
122.412
87.468
69.175
52.722
51.337
23.098
8.306
8.282
0.382
O
CO2Me
N
H
N
1e 13C-NMR
(101 MHz, CDCl3)
f1 (ppm)

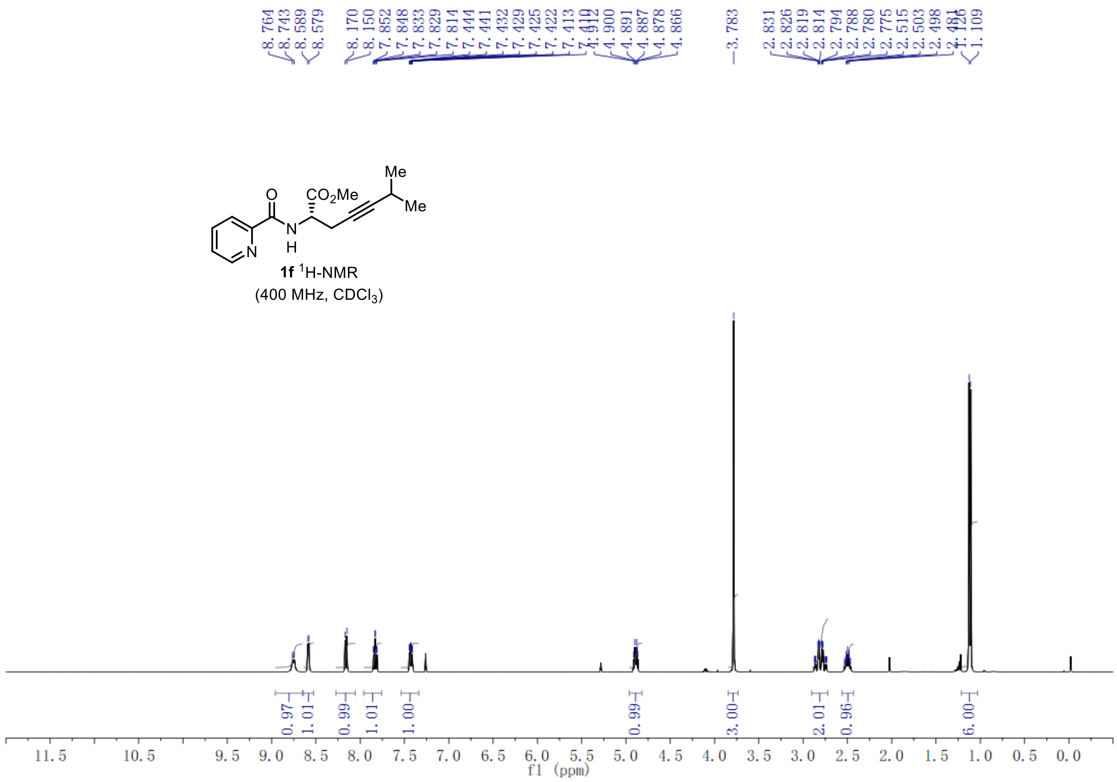

1f ^{1}H-NMR
(400 MHz, $CDCl_3$)

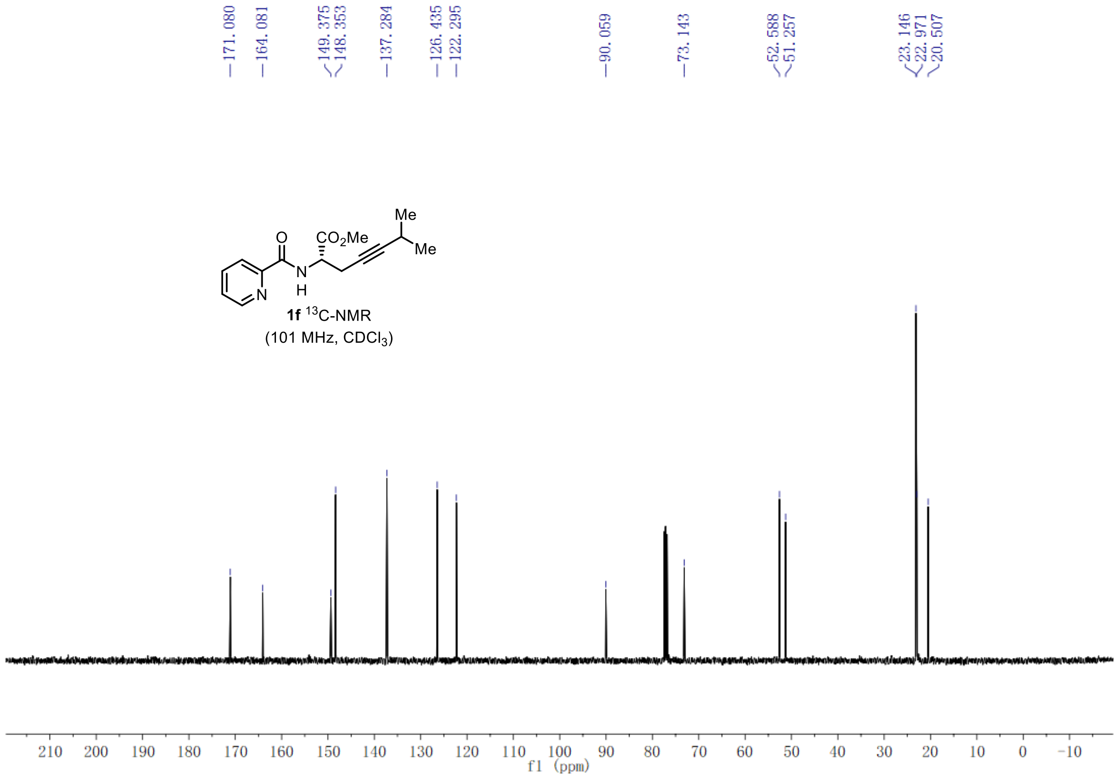

1f ^{13}C-NMR
(101 MHz, $CDCl_3$)

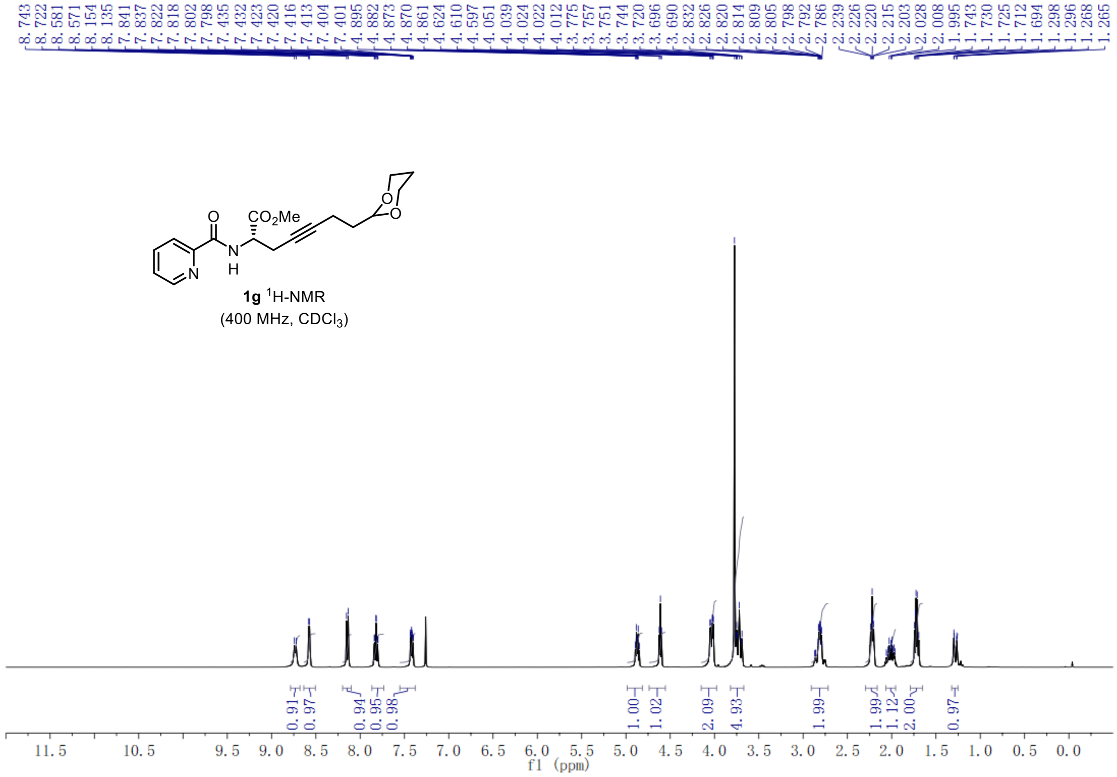
1g ^{1}H-NMR
(400 MHz, CDCl$_3$)
f1 (ppm)

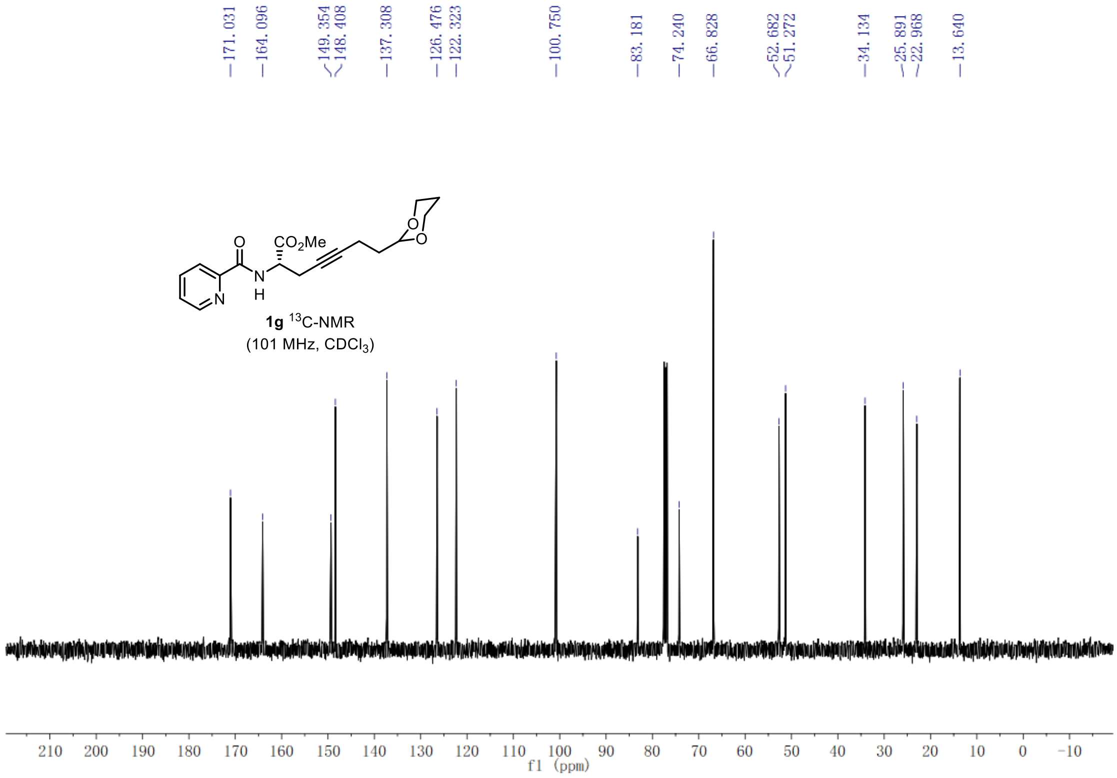
1g ^{13}C-NMR
(101 MHz, CDCl$_3$)
171.031
164.096
149.354
148.408
137.308
126.476
122.323
100.750
83.181
74.240
66.828
52.682
51.272
34.134
25.891
22.968
13.640
f1 (ppm)

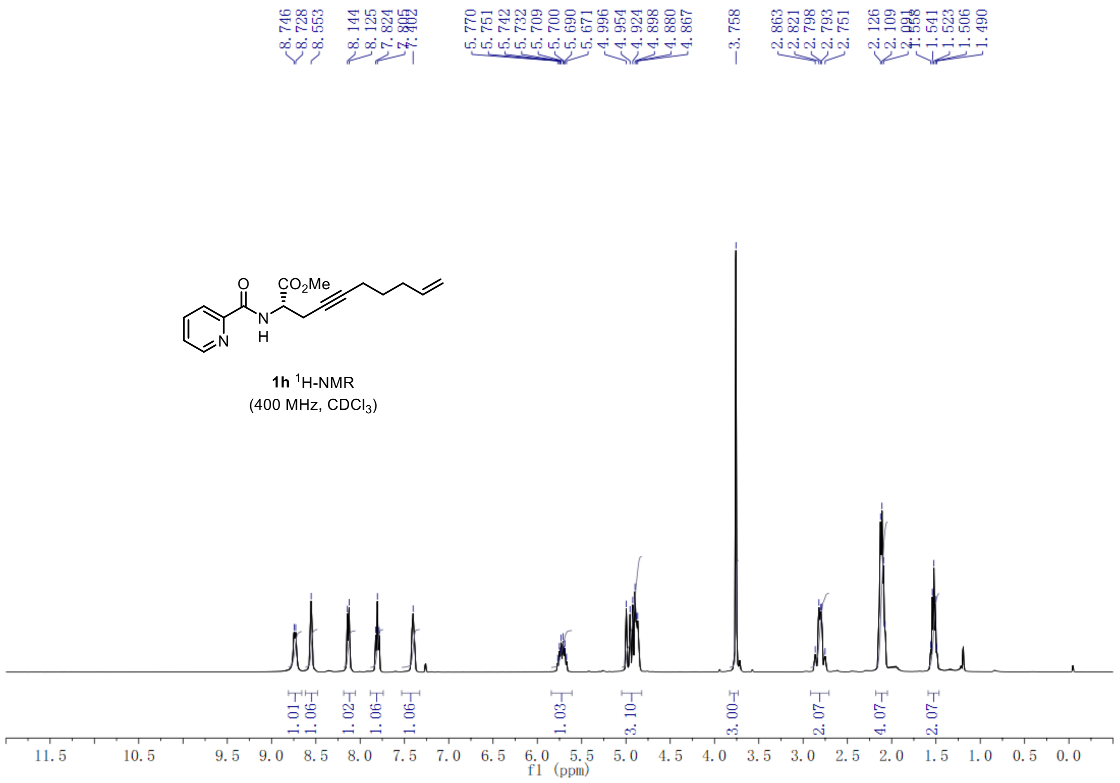

1h ^{1}H-NMR
(400 MHz, $CDCl_3$)

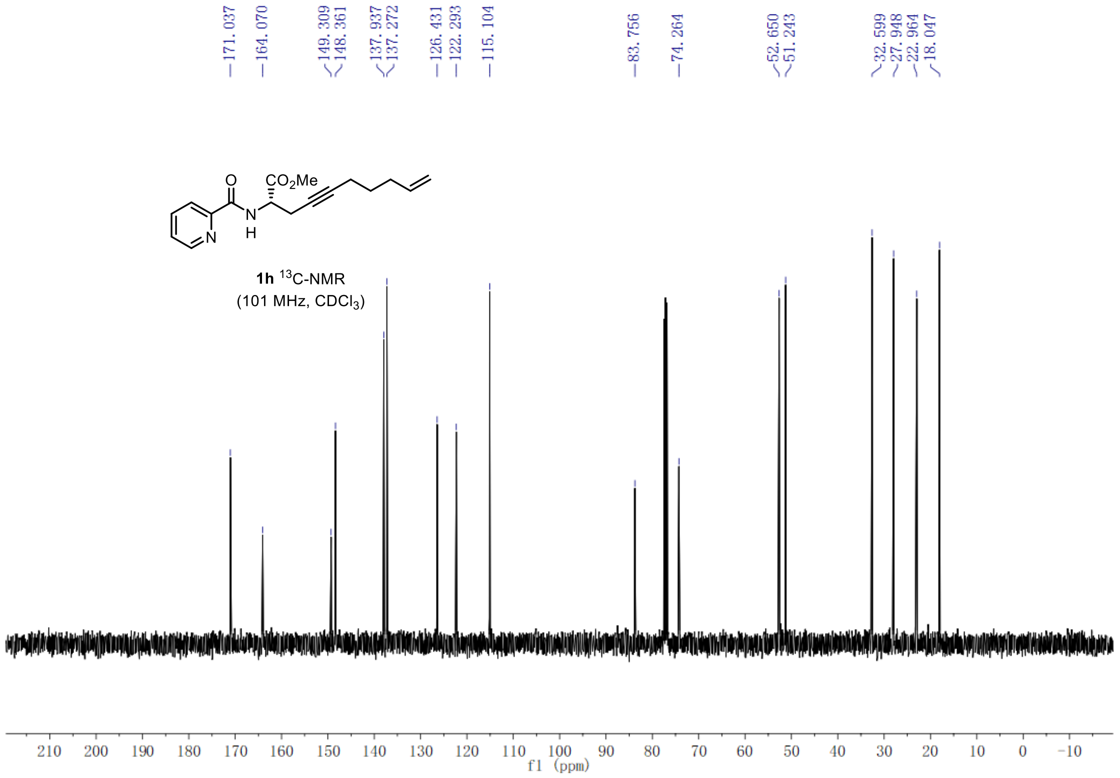

1h ^{13}C-NMR
(101 MHz, $CDCl_3$)

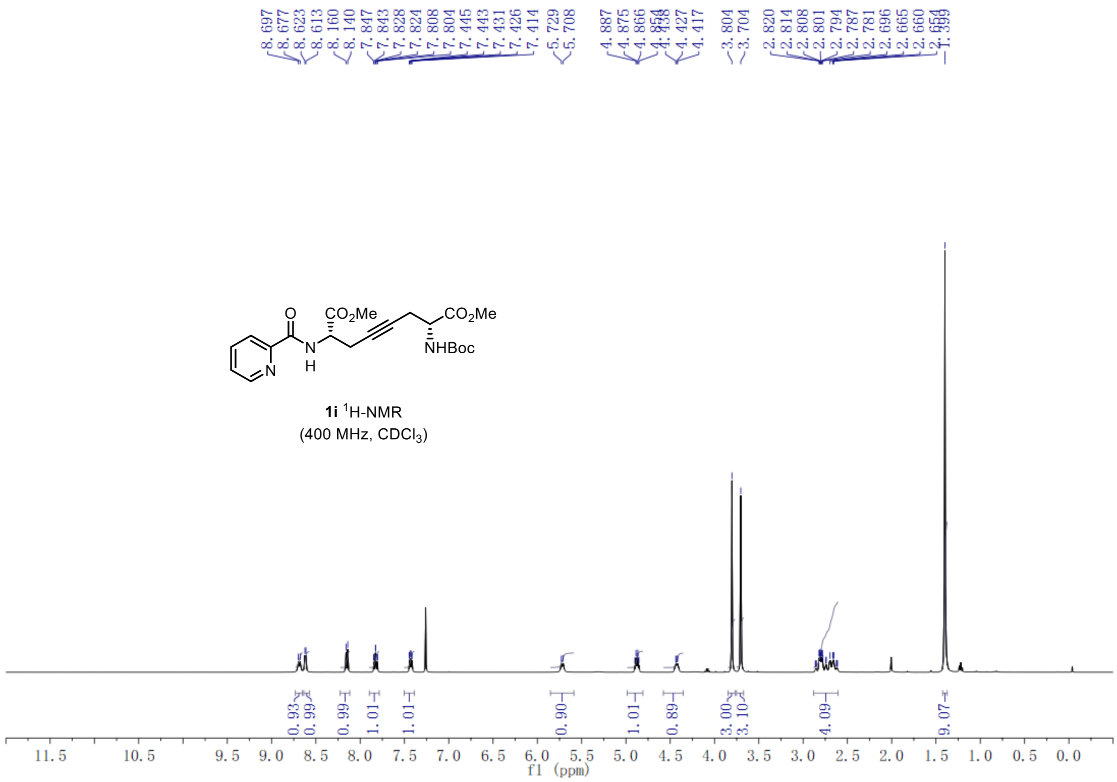
8.697
8.677
8.623
8.613
8.160
8.140
7.847
7.843
7.828
7.824
7.808
7.804
7.445
7.443
7.431
7.426
7.414
5.729
5.708
4.887
4.875
4.866
4.427
4.417
3.804
3.704
2.820
2.814
2.808
2.801
2.794
2.787
2.781
2.696
2.665
2.660
1i ^{1}H-NMR
(400 MHz, $CDCl_3$)
0.93
0.99
0.99
1.01
1.01
0.90
1.01
0.89
3.00
3.10
4.09
9.07
f1 (ppm)

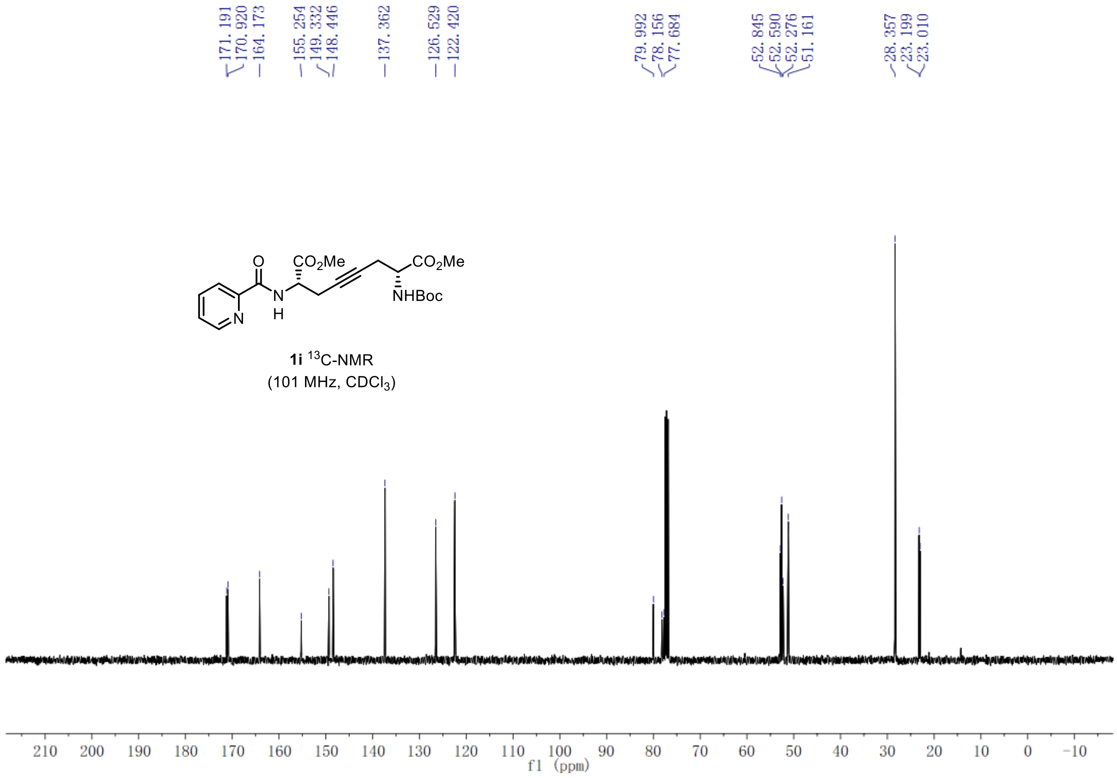
171.191
170.920
164.173
155.254
149.332
148.446
137.362
126.529
122.420
79.992
78.156
77.684
52.845
52.590
52.276
51.161
28.357
23.199
23.010
1i ^{13}C-NMR
(101 MHz, $CDCl_3$)
f1 (ppm)

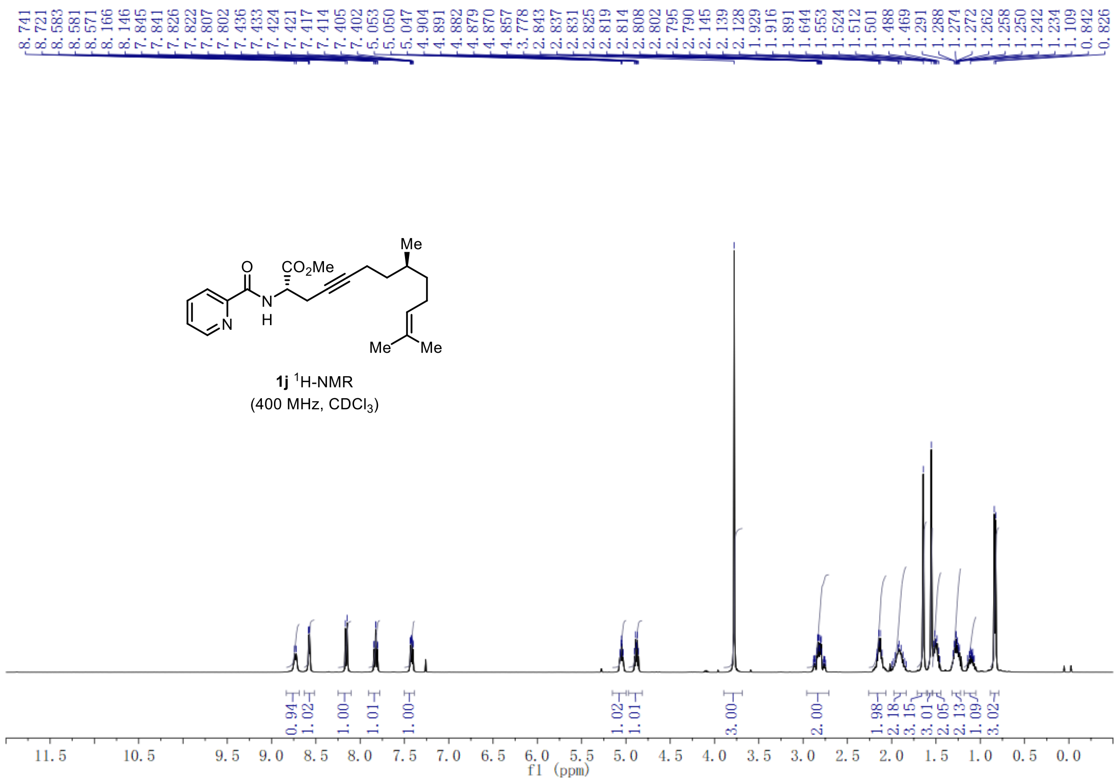
8.741 8.721 8.583 8.581 8.571 8.166 8.146 7.845 7.841 7.826 7.822 7.807 7.802 7.436 7.433 7.424 7.421 7.417 7.414 7.405 7.402 5.053 5.050 5.047 4.904 4.891 4.882 4.879 4.870 4.857 3.778 2.843 2.837 2.831 2.825 2.819 2.814 2.808 2.802 2.795 2.790 2.145 2.139 2.128 1.929 1.916 1.891 1.644 1.553 1.524 1.512 1.501 1.488 1.469 1.291 1.288 1.274 1.272 1.262 1.258 1.250 1.242 1.234 1.109 0.842 0.826
O
CO2Me
Me
N
H
N
Me
Me
1j 1H-NMR
(400 MHz, CDCl3)
0.94 1.02 1.00 1.01 1.00 1.02 1.01 3.00 2.00 1.98 2.18 3.15 3.01 2.05 2.13 1.09 3.02
11.5 10.5 9.5 9.0 8.5 8.0 7.5 7.0 6.5 6.0 5.5 5.0 4.5 4.0 3.5 3.0 2.5 2.0 1.5 1.0 0.5 0.0
f1 (ppm)

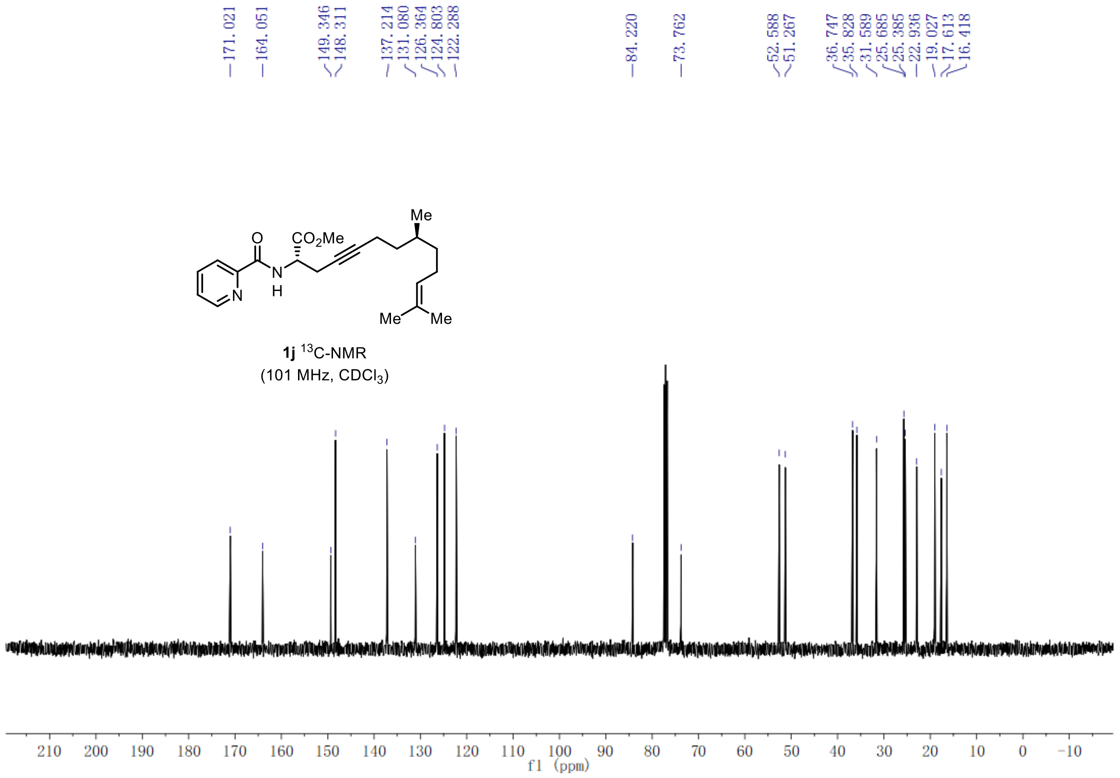
171.021 164.051 149.346 148.311 137.214 131.080 126.364 124.803 122.288 84.220 73.762 52.588 51.267 36.747 35.828 31.589 25.685 25.385 22.936 19.027 17.613 16.418
O
CO2Me
Me
N
H
N
Me
Me
1j 13C-NMR
(101 MHz, CDCl3)
210 200 190 180 170 160 150 140 130 120 110 100 90 80 70 60 50 40 30 20 10 0 -10
f1 (ppm)

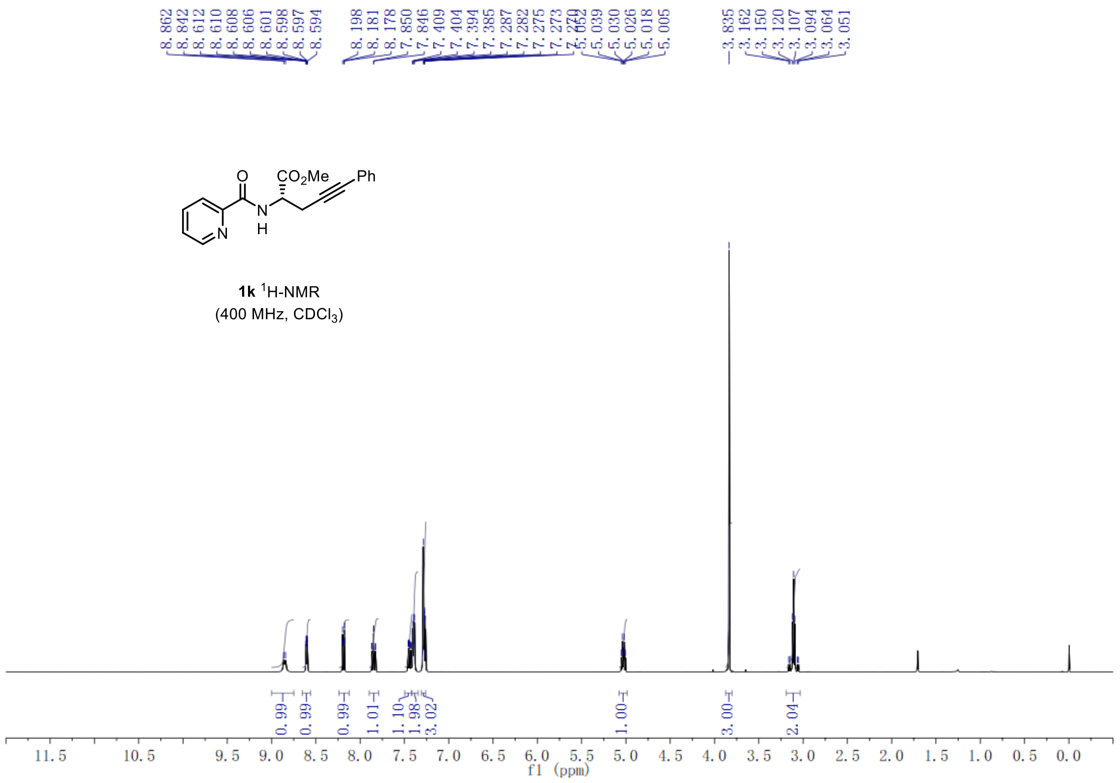
8.862
8.842
8.612
8.610
8.608
8.606
8.601
8.598
8.597
8.594
8.198
8.181
8.178
7.850
7.846
7.409
7.404
7.394
7.385
7.287
7.282
7.275
7.273
5.039
5.030
5.026
5.018
5.005
3.835
3.162
3.150
3.120
3.107
3.094
3.064
3.051
O
CO2Me
Ph
N
H
N
1k 1H-NMR
(400 MHz, CDCl3)
0.99
0.99
0.99
1.01
1.10
1.98
3.02
1.00
3.00
2.04
f1 (ppm)

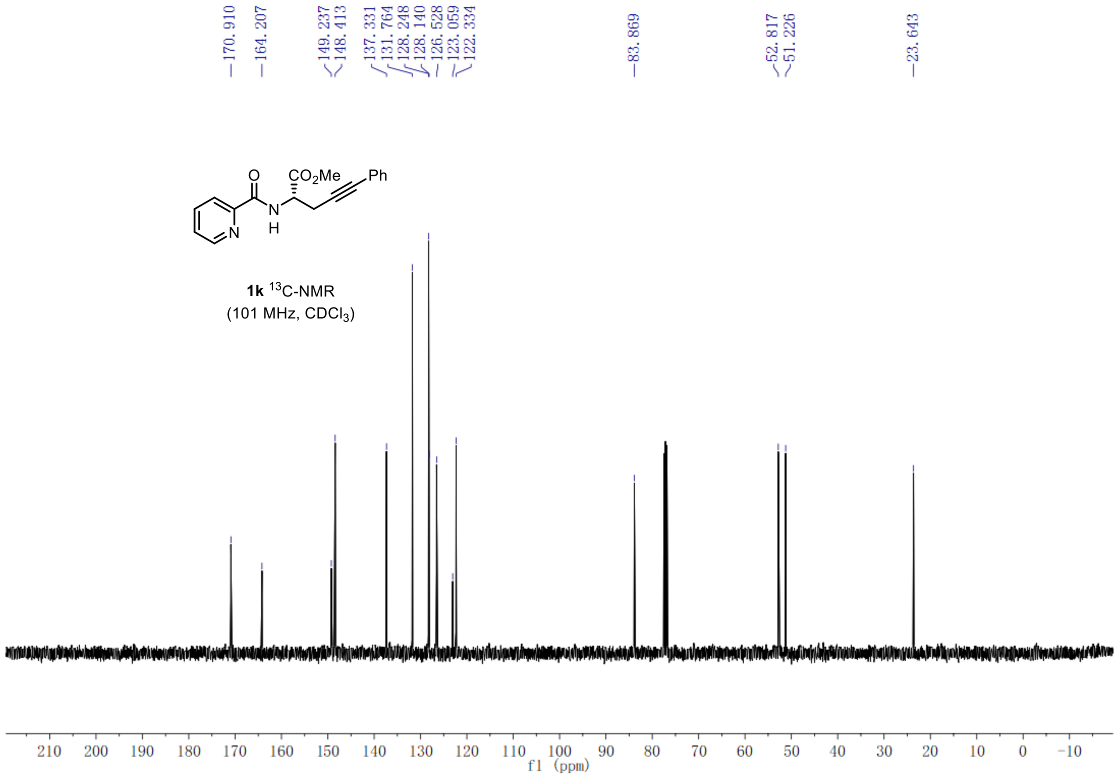
170.910
164.207
149.237
148.413
137.331
131.764
128.248
128.140
126.528
123.059
122.334
83.869
52.817
51.226
23.643
O
CO2Me
Ph
N
H
N
1k 13C-NMR
(101 MHz, CDCl3)
f1 (ppm)

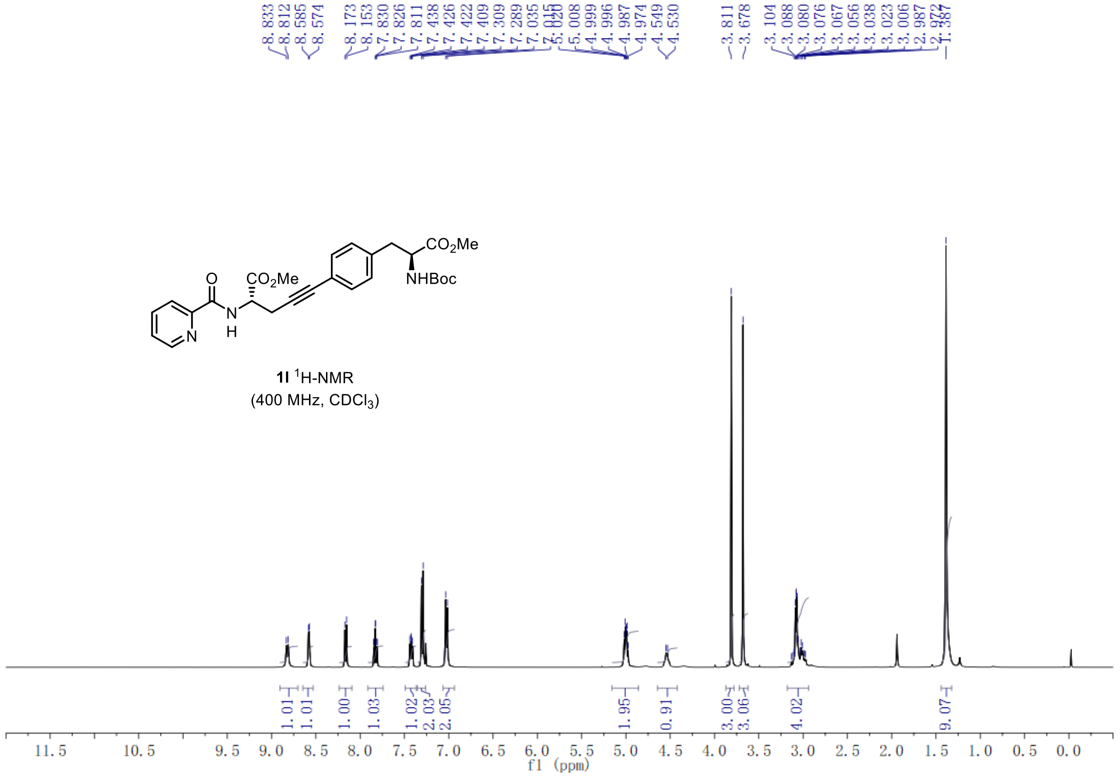

1l ^{1}H-NMR
(400 MHz, $CDCl_3$)

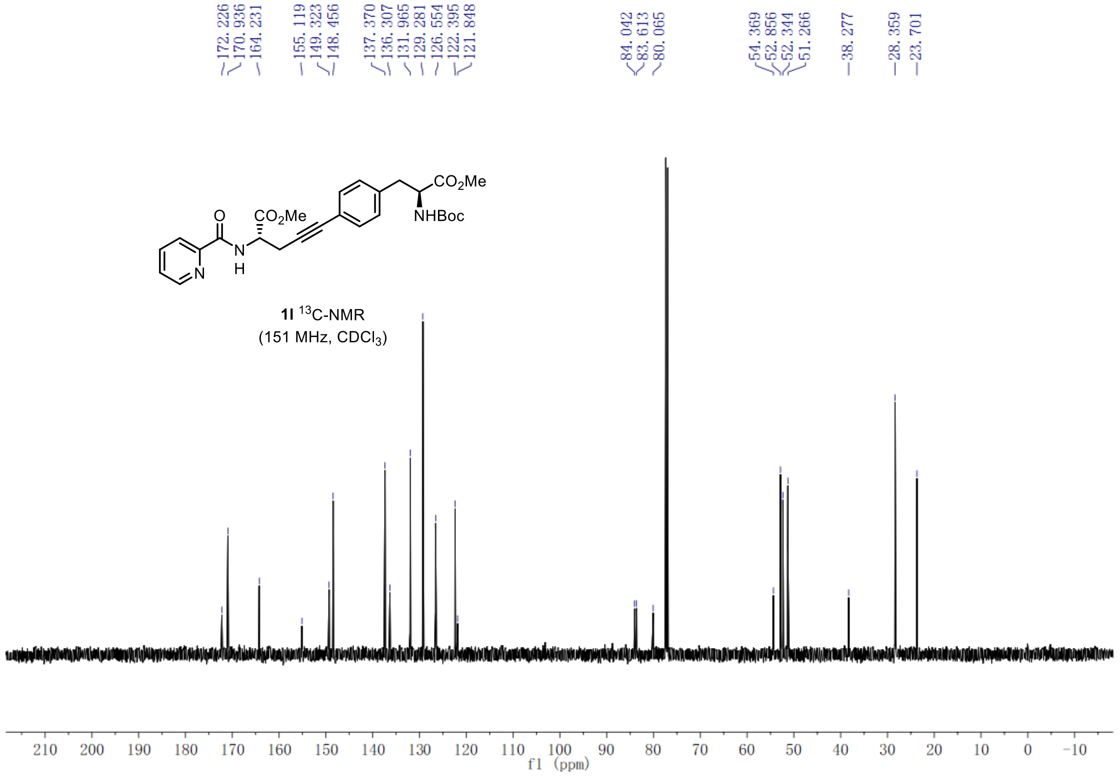

1l ^{13}C-NMR
(151 MHz, $CDCl_3$)

1m ^{1}H-NMR
(400 MHz, $CDCl_3$)

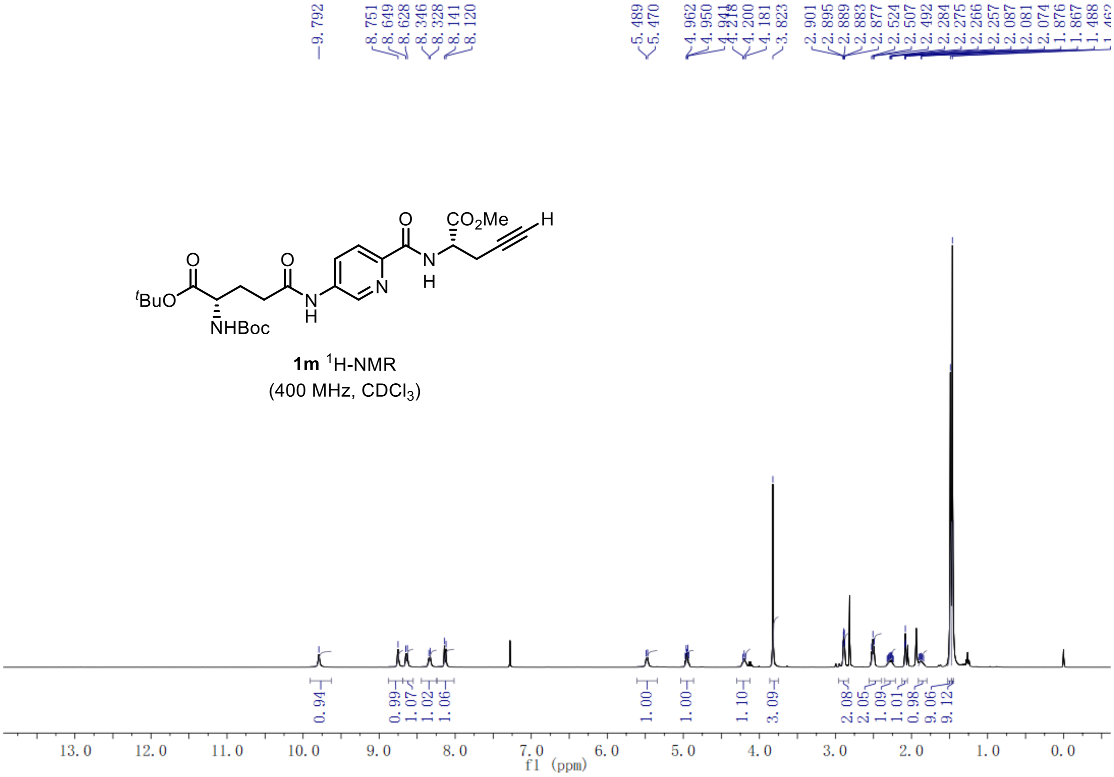

1m ^{13}C-NMR
(101 MHz, $CDCl_3$)

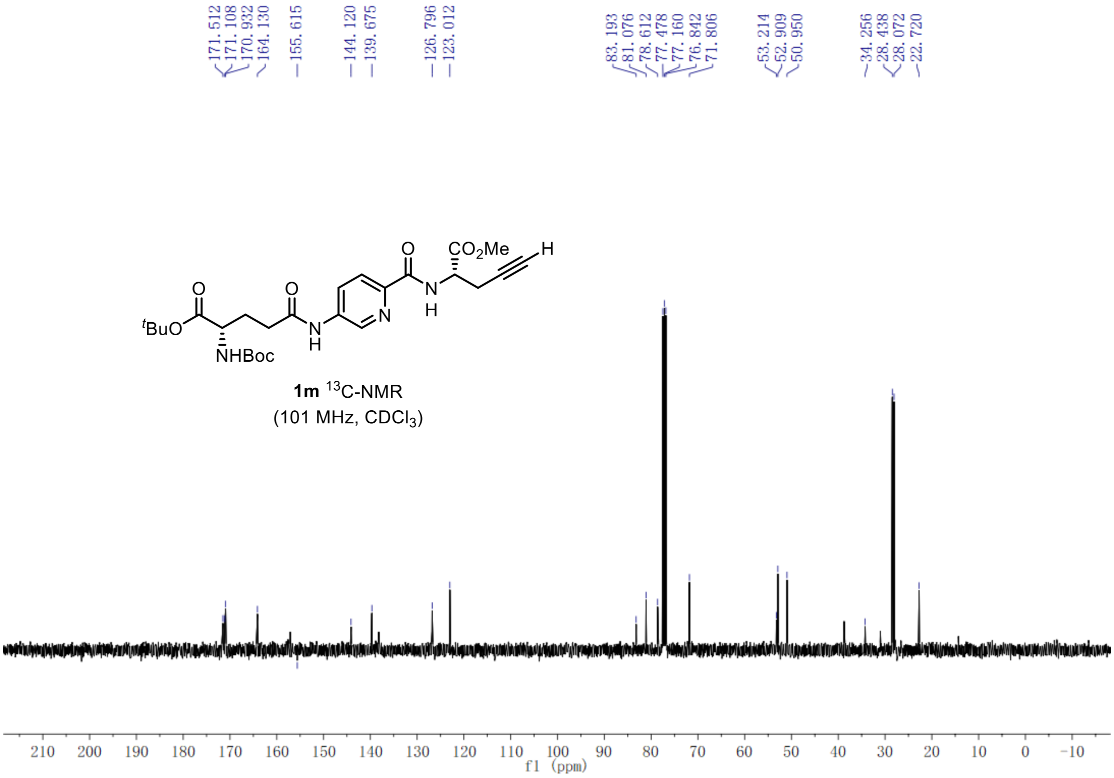

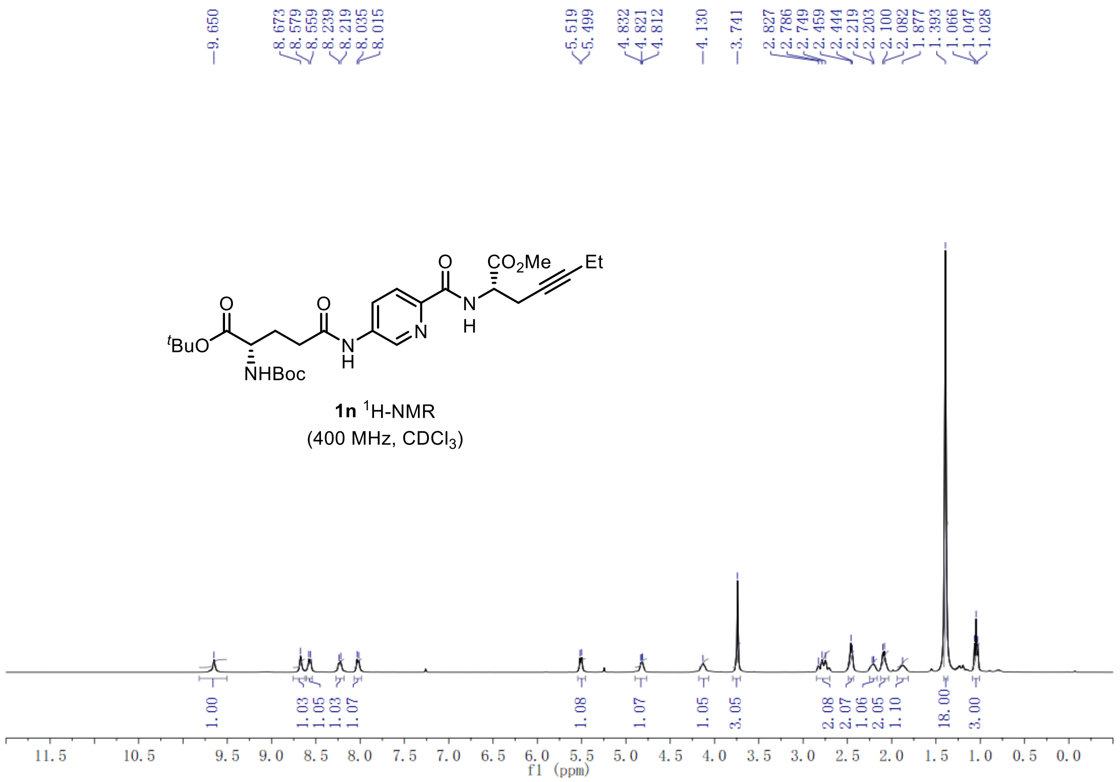

1n ^{1}H-NMR
(400 MHz, $CDCl_3$)

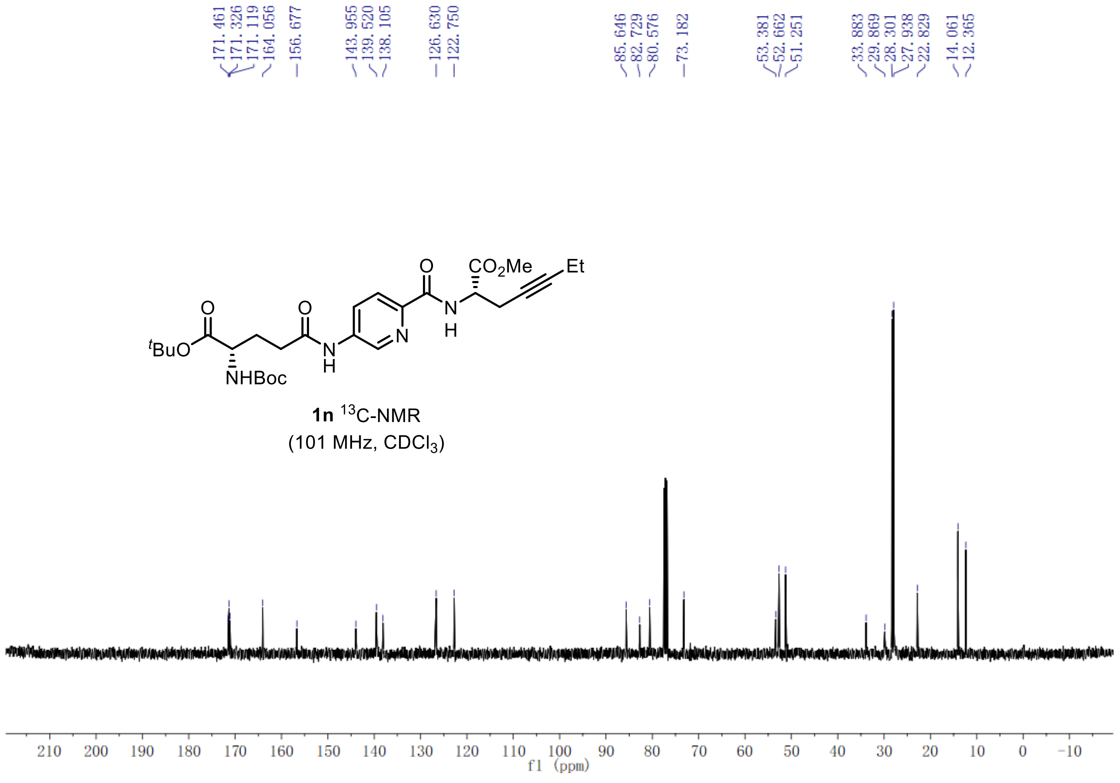

1n ^{13}C-NMR
(101 MHz, $CDCl_3$)

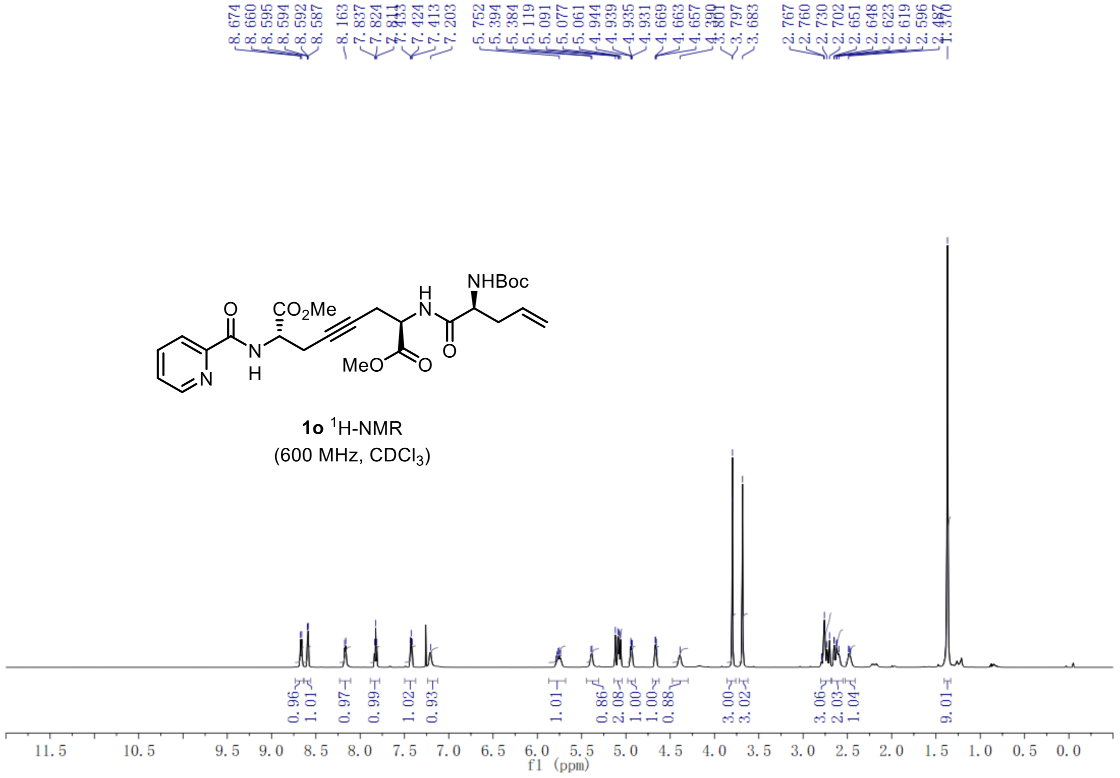

8.674
8.660
8.595
8.594
8.592
8.587
8.163
7.837
7.824
7.424
7.413
7.203
5.752
5.394
5.384
5.119
5.091
5.077
5.061
4.944
4.939
4.935
4.931
4.669
4.663
4.657
3.797
3.683
2.767
2.760
2.730
2.702
2.651
2.648
2.623
2.619
2.596
1.370
NHBoc
CO_2Me
O
N
H
N
H
N
MeO
O
O
1o ^{1}H-NMR
(600 MHz, $CDCl_3$)
0.96
1.01
0.97
0.99
1.02
0.93
1.01
0.86
2.08
1.00
1.00
0.88
3.00
3.02
3.06
2.03
1.04
9.01
f1 (ppm)

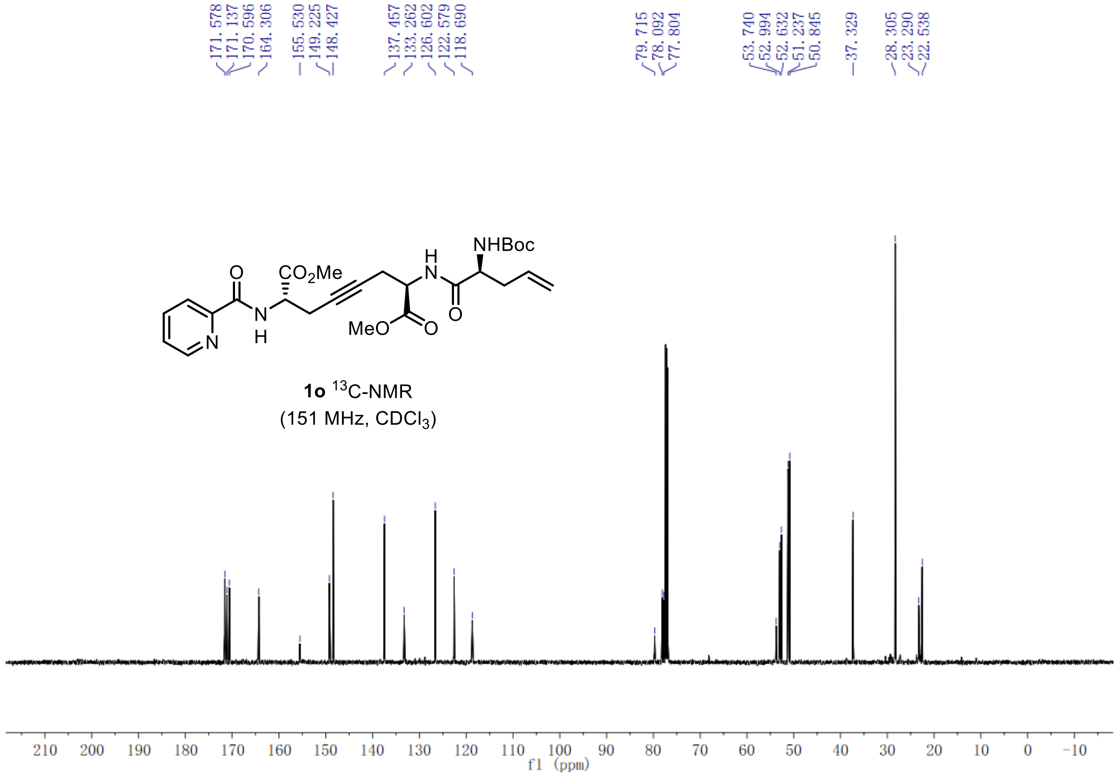

171.578
171.137
170.596
164.306
155.530
149.225
148.427
137.457
133.262
126.602
122.579
118.690
79.715
78.092
77.804
53.740
52.994
52.632
51.237
50.845
37.329
28.305
23.290
22.538
NHBoc
CO_2Me
O
N
H
N
H
N
MeO
O
O
1o ^{13}C-NMR
(151 MHz, $CDCl_3$)
f1 (ppm)

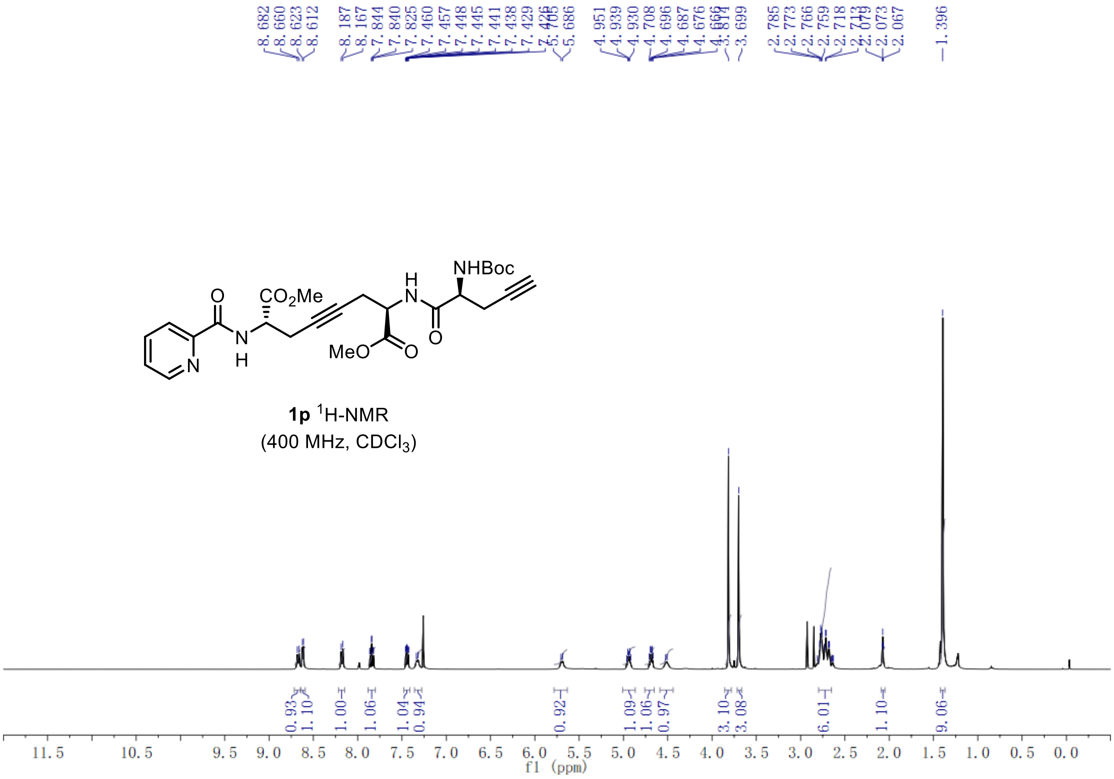
8.682
8.660
8.623
8.612
8.187
8.167
7.844
7.840
7.825
7.460
7.457
7.448
7.445
7.441
7.438
7.429
5.686
4.951
4.939
4.930
4.708
4.696
4.687
4.676
3.699
2.785
2.773
2.766
2.759
2.718
2.073
2.067
1.396
NHBoc
CO2Me
H
N
O
N
H
N
MeO
O
O
1p 1H-NMR
(400 MHz, CDCl3)
0.93
1.10
1.00
1.06
1.04
0.94
0.92
1.09
1.06
0.97
3.10
3.08
6.01
1.10
9.06
11.5 10.5 9.5 9.0 8.5 8.0 7.5 7.0 6.5 6.0 5.5 5.0 4.5 4.0 3.5 3.0 2.5 2.0 1.5 1.0 0.5 0.0
f1 (ppm)

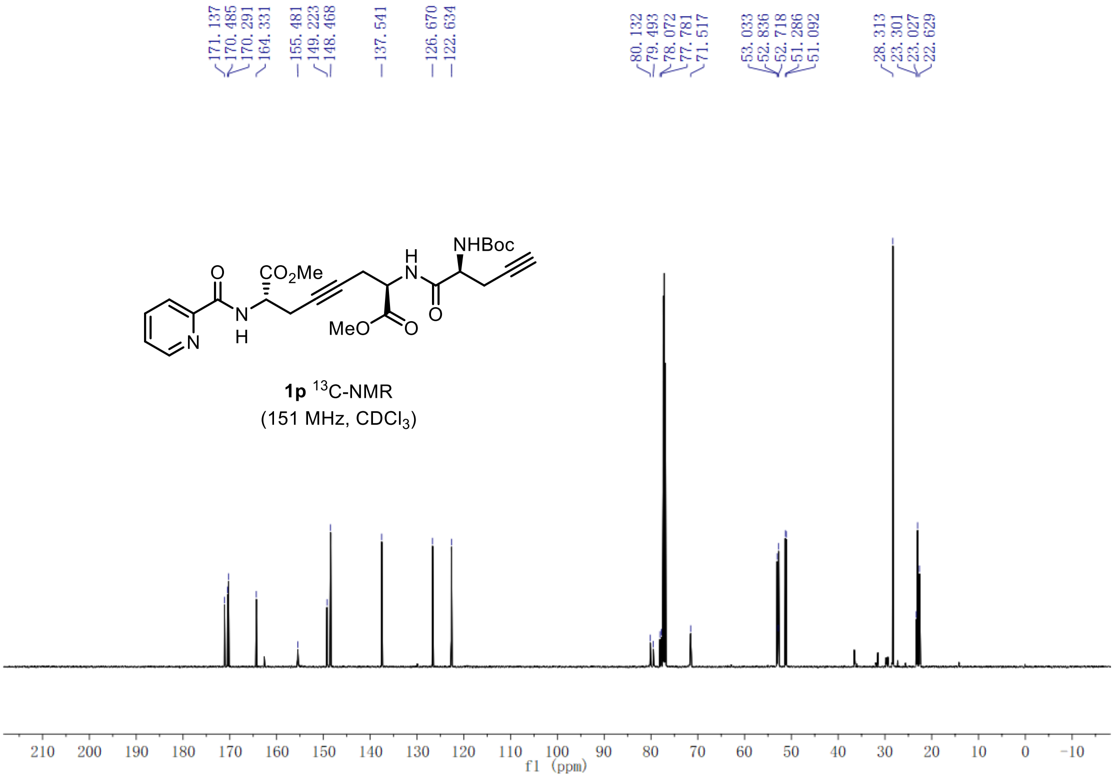
171.137
170.485
170.291
164.331
155.481
149.223
148.468
137.541
126.670
122.634
80.132
79.493
78.072
77.781
71.517
53.033
52.836
52.718
51.286
51.092
28.313
23.301
23.027
22.629
NHBoc
CO2Me
H
N
O
N
H
N
MeO
O
O
1p 13C-NMR
(151 MHz, CDCl3)
210 200 190 180 170 160 150 140 130 120 110 100 90 80 70 60 50 40 30 20 10 0 -10
f1 (ppm)

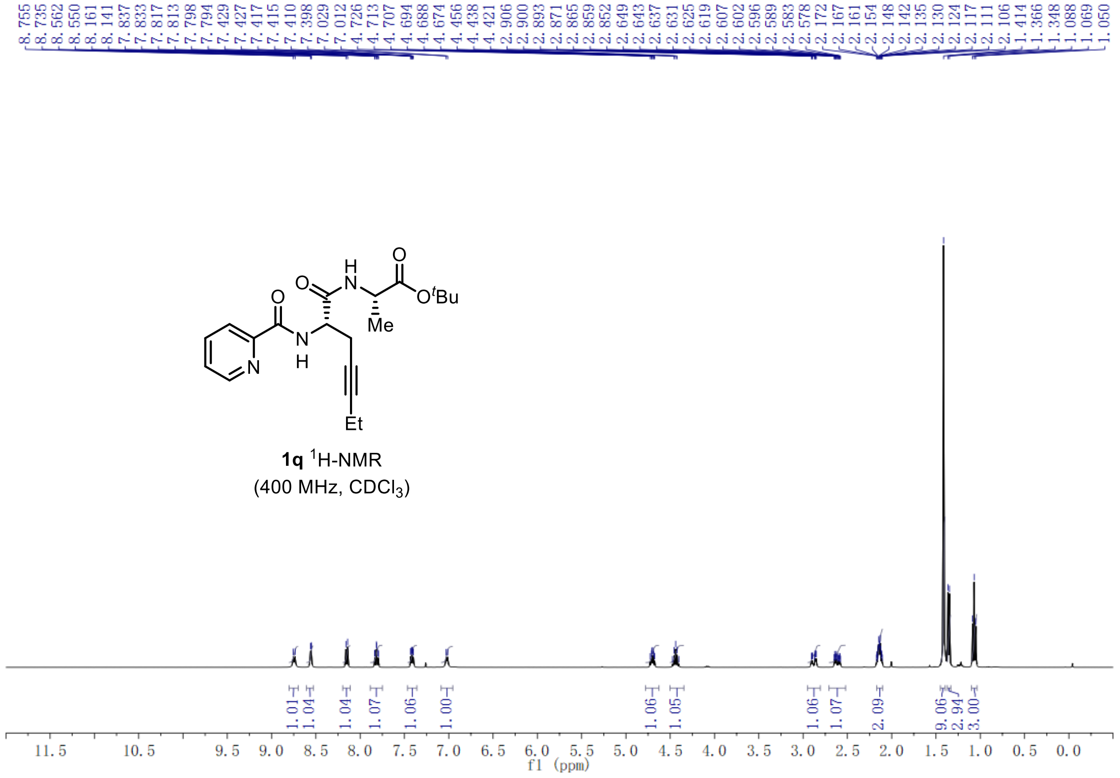
8.755 8.735 8.562 8.550 8.161 8.141 7.837 7.833 7.817 7.813 7.798 7.794 7.429 7.427 7.417 7.415 7.410 7.398 7.029 7.012 4.726 4.713 4.707 4.694 4.688 4.674 4.456 4.438 4.421 2.906 2.900 2.893 2.871 2.865 2.859 2.852 2.649 2.643 2.637 2.631 2.625 2.619 2.607 2.602 2.596 2.589 2.583 2.578 2.172 2.167 2.161 2.154 2.148 2.142 2.135 2.130 2.124 2.117 2.111 2.106 1.414 1.366 1.348 1.088 1.069 1.050
O
H
N
O
O
OtBu
N
H
Me
N
Et
1q 1H-NMR
(400 MHz, CDCl3)
1.01 1.04 1.04 1.07 1.06 1.00 1.06 1.05 1.06 1.07 2.09 9.06 2.94 3.00
11.5 10.5 9.5 9.0 8.5 8.0 7.5 7.0 6.5 6.0 5.5 5.0 4.5 4.0 3.5 3.0 2.5 2.0 1.5 1.0 0.5 0.0
f1 (ppm)

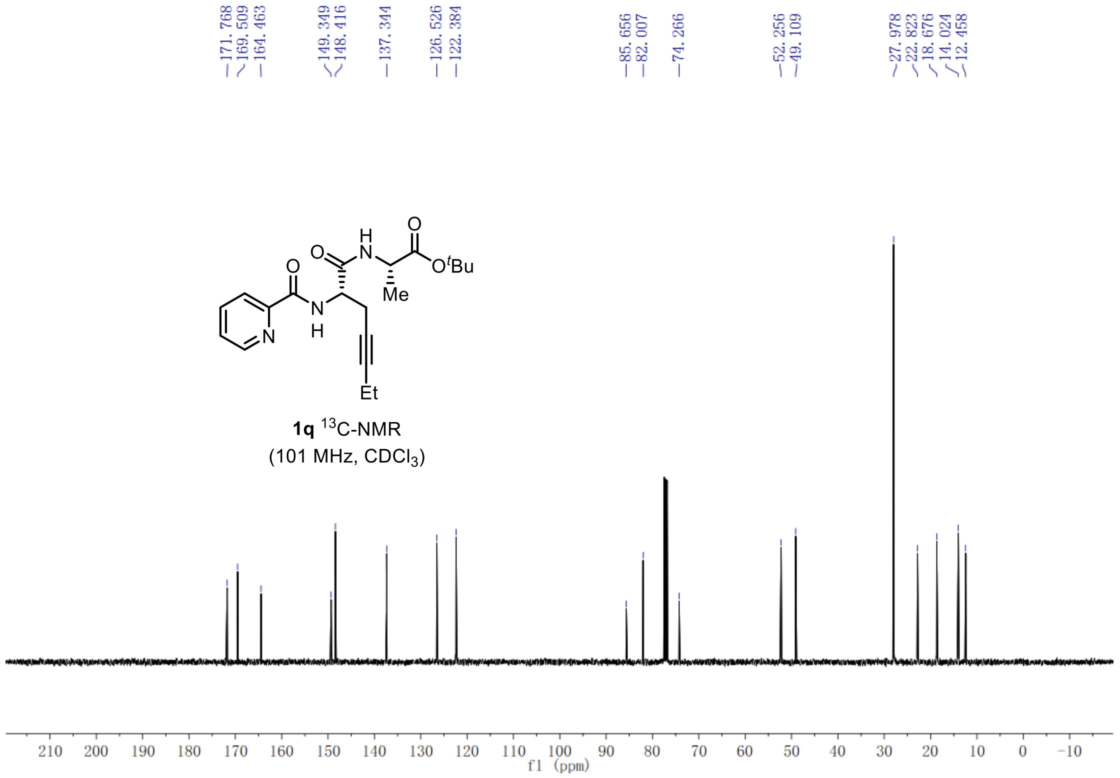
171.768 169.509 164.463 149.349 148.416 137.344 126.526 122.384 85.656 82.007 74.266 52.256 49.109 27.978 22.823 18.676 14.024 12.458
O
H
N
O
O
OtBu
N
H
Me
N
Et
1q 13C-NMR
(101 MHz, CDCl3)
210 200 190 180 170 160 150 140 130 120 110 100 90 80 70 60 50 40 30 20 10 0 -10
f1 (ppm)

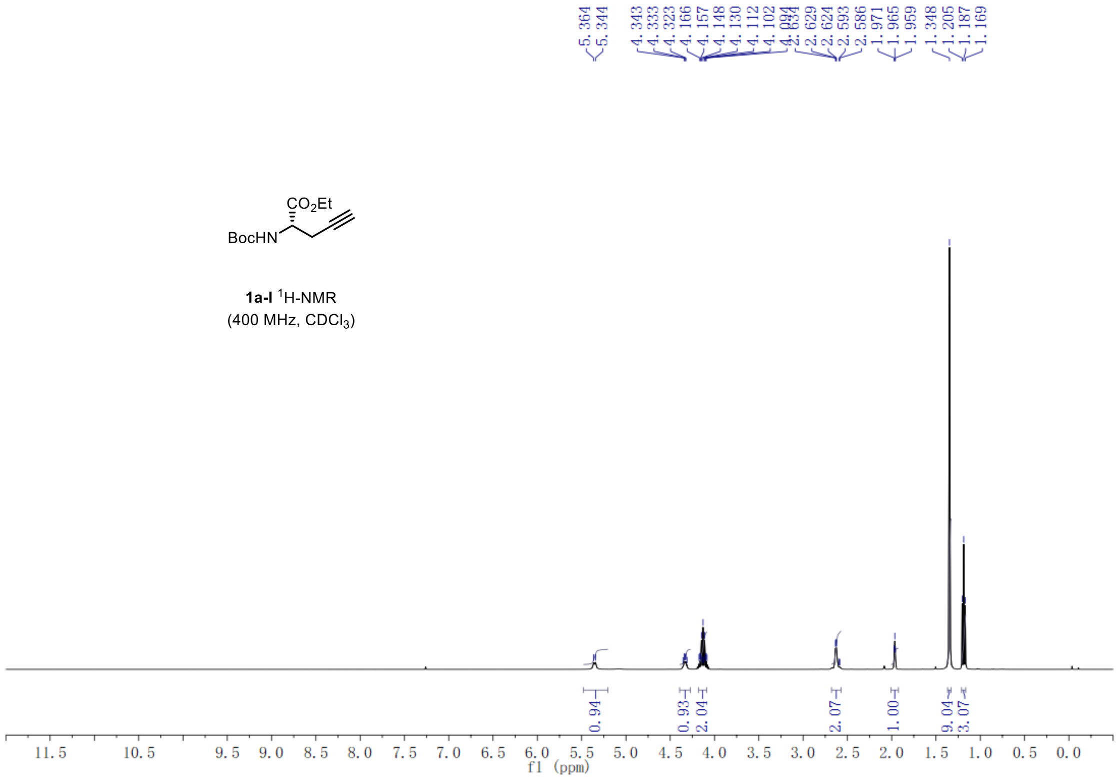
5.364
5.344
4.343
4.333
4.323
4.166
4.157
4.148
4.130
4.112
4.102
2.629
2.624
2.593
2.586
1.971
1.965
1.959
1.348
1.205
1.187
1.169
CO2Et
BocHN
1a-l 1H-NMR
(400 MHz, CDCl3)
0.94
0.93
2.04
2.07
1.00
9.04
3.07
f1 (ppm)

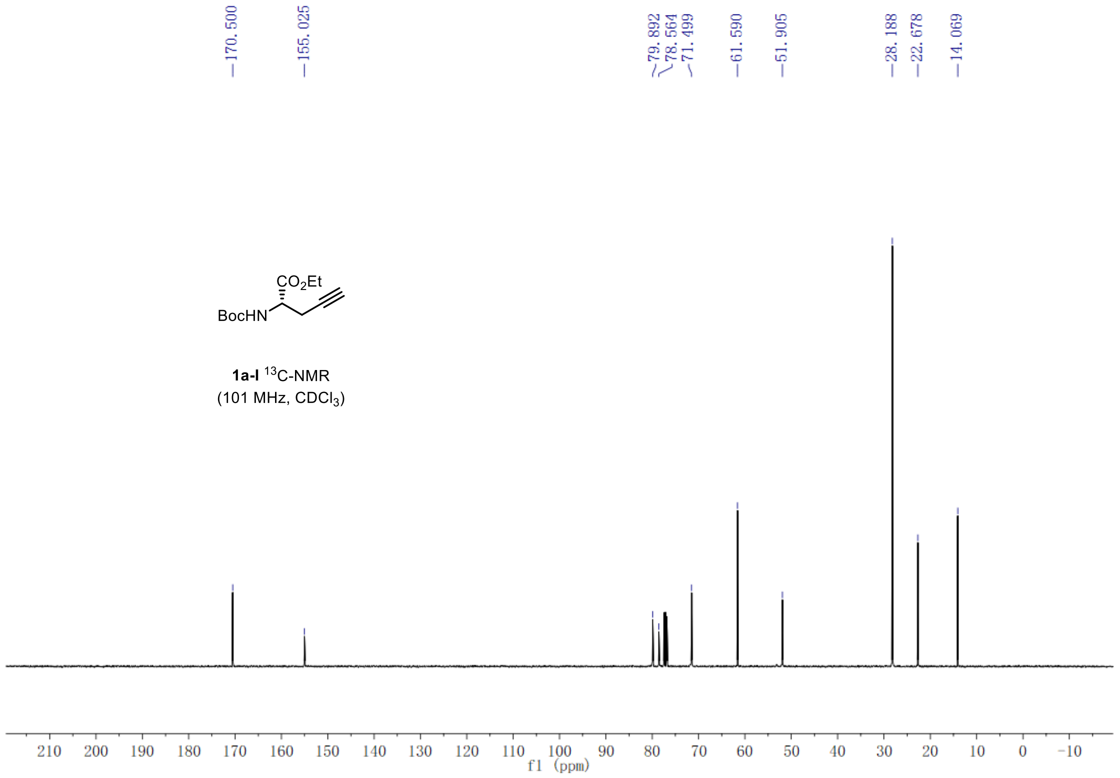
170.500
155.025
79.892
78.564
71.499
61.590
51.905
28.188
22.678
14.069
CO2Et
BocHN
1a-l 13C-NMR
(101 MHz, CDCl3)
f1 (ppm)

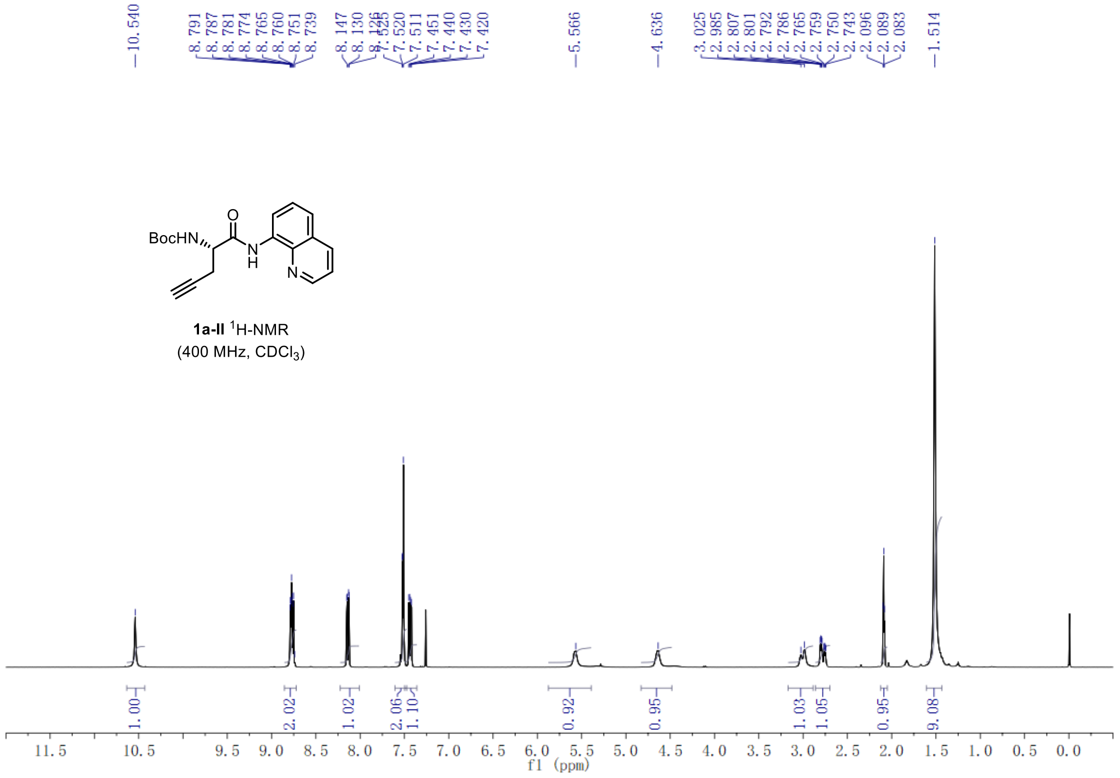
10.540
8.791
8.787
8.781
8.774
8.765
8.760
8.751
8.739
8.147
8.130
8.126
7.525
7.520
7.511
7.451
7.440
7.430
7.420
5.566
4.636
3.025
2.985
2.807
2.801
2.792
2.786
2.765
2.759
2.750
2.743
2.096
2.089
2.083
1.514
BocHN
O
N
H
N
1a-II ^{1}H-NMR
(400 MHz, $CDCl_3$)
1.00
2.02
1.02
2.06
1.10
0.92
0.95
1.03
1.05
0.95
9.08
11.5
10.5
9.5
9.0
8.5
8.0
7.5
7.0
6.5
6.0
5.5
5.0
4.5
4.0
3.5
3.0
2.5
2.0
1.5
1.0
0.5
0.0
f1 (ppm)

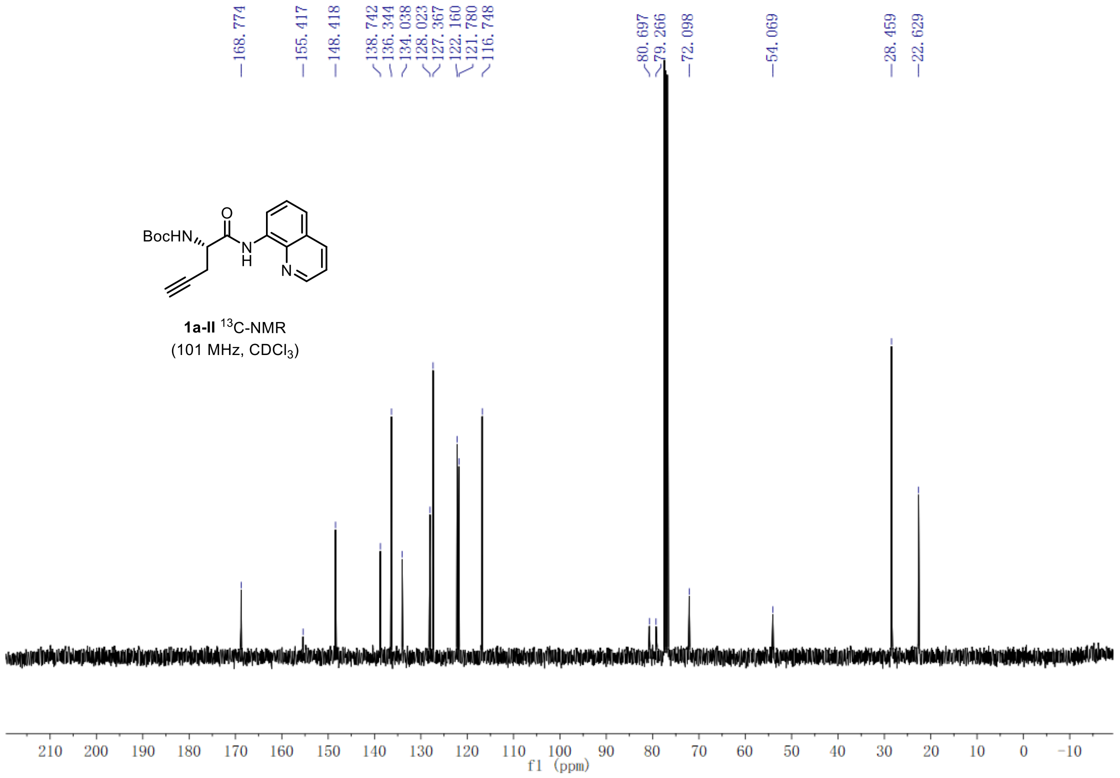
168.774
155.417
148.418
138.742
136.344
134.038
128.023
127.367
122.160
121.780
116.748
80.697
79.266
72.098
54.069
28.459
22.629
BocHN
O
N
H
N
1a-II ^{13}C-NMR
(101 MHz, $CDCl_3$)
210
200
190
180
170
160
150
140
130
120
110
100
90
80
70
60
50
40
30
20
10
0
-10
f1 (ppm)

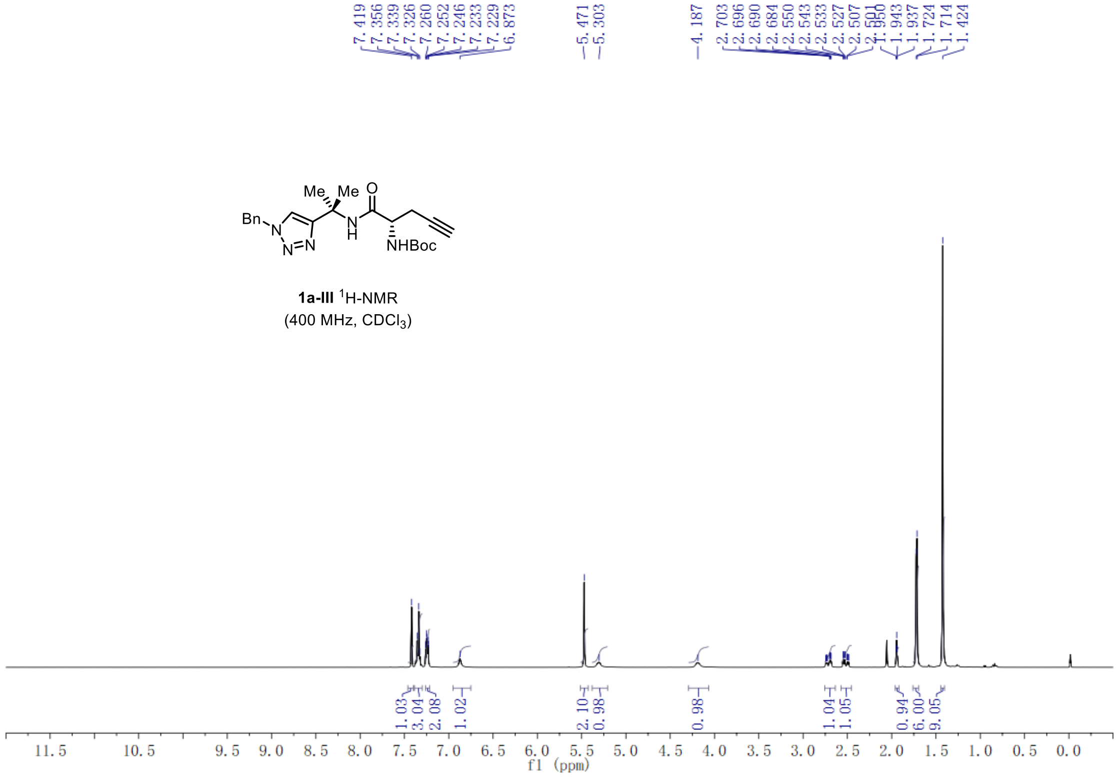
1a-III 1H-NMR
(400 MHz, CDCl3)
Bn
Me Me O
N
H
NHBoc
f1 (ppm)

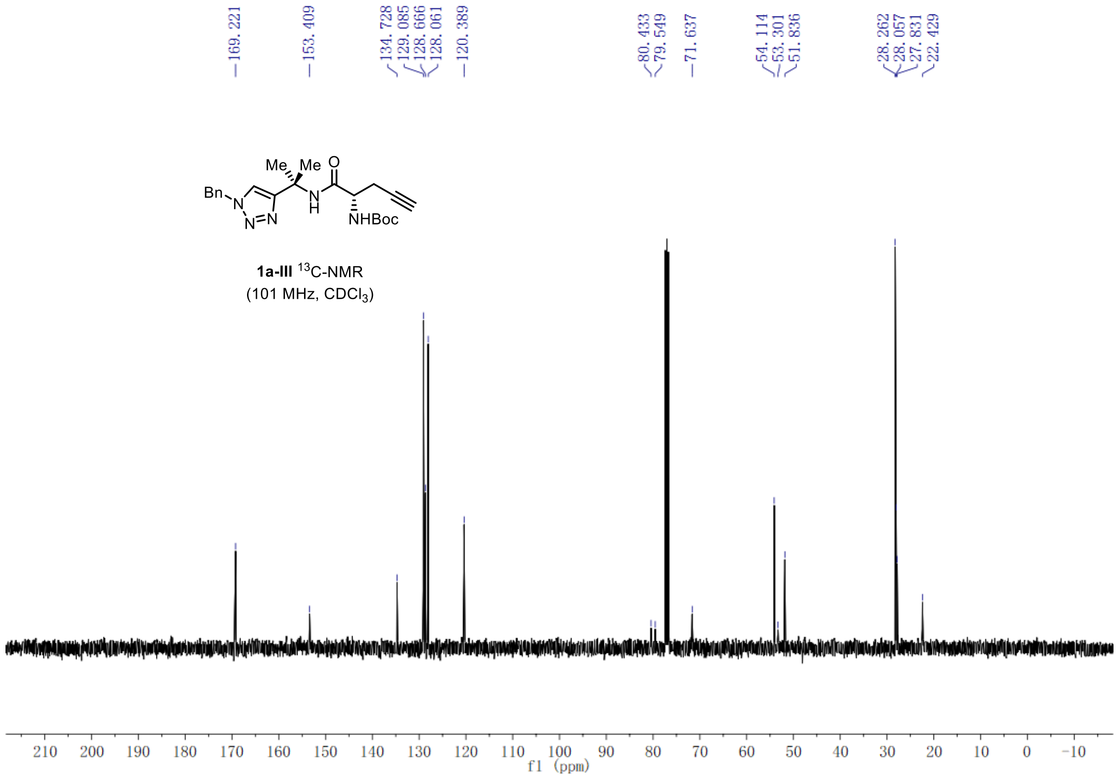
1a-III 13C-NMR
(101 MHz, CDCl3)
Bn
Me Me O
N
H
NHBoc
f1 (ppm)

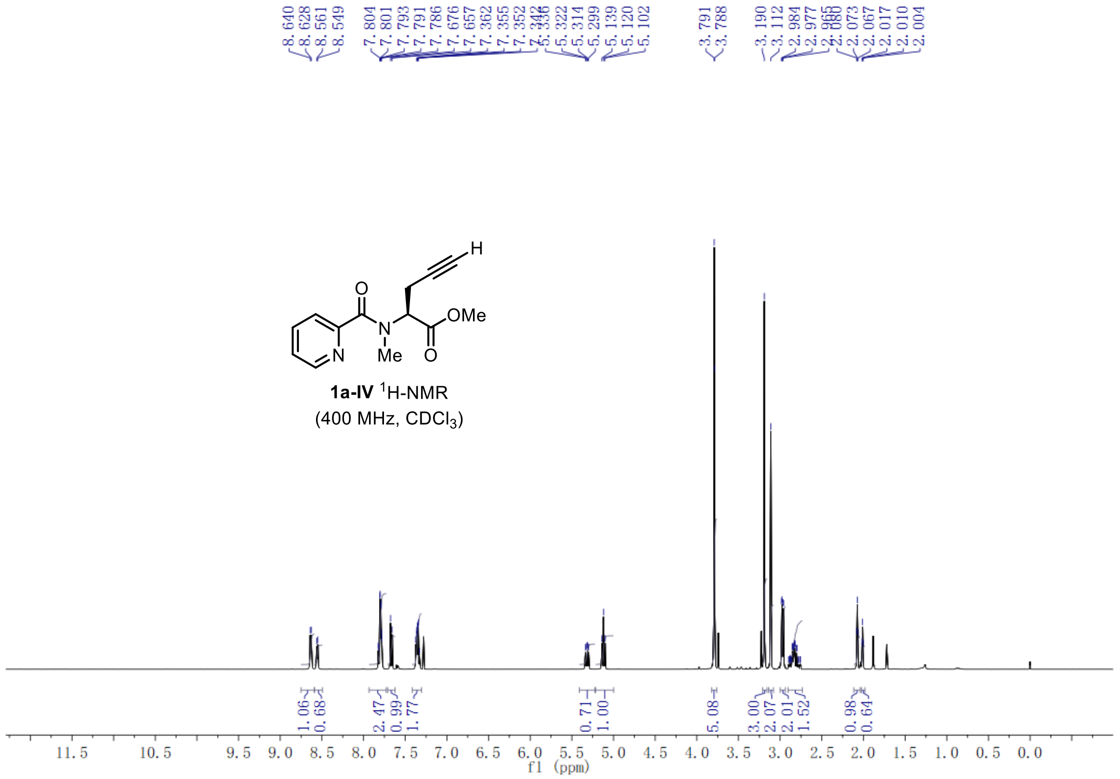
8.640
8.628
8.561
8.549
7.804
7.801
7.793
7.791
7.786
7.676
7.657
7.362
7.355
7.352
5.322
5.314
5.299
5.139
5.120
5.102
3.791
3.788
3.190
3.112
2.984
2.977
2.073
2.067
2.017
2.010
2.004
H
O
N
OMe
N
Me
O
1a-IV 1H-NMR
(400 MHz, CDCl3)
1.06
0.68
2.47
0.99
1.77
0.71
1.00
5.08
3.00
2.07
2.01
1.52
0.98
0.64
11.5
10.5
9.5
9.0
8.5
8.0
7.5
7.0
6.5
6.0
5.5
5.0
4.5
4.0
3.5
3.0
2.5
2.0
1.5
1.0
0.5
0.0
f1 (ppm)

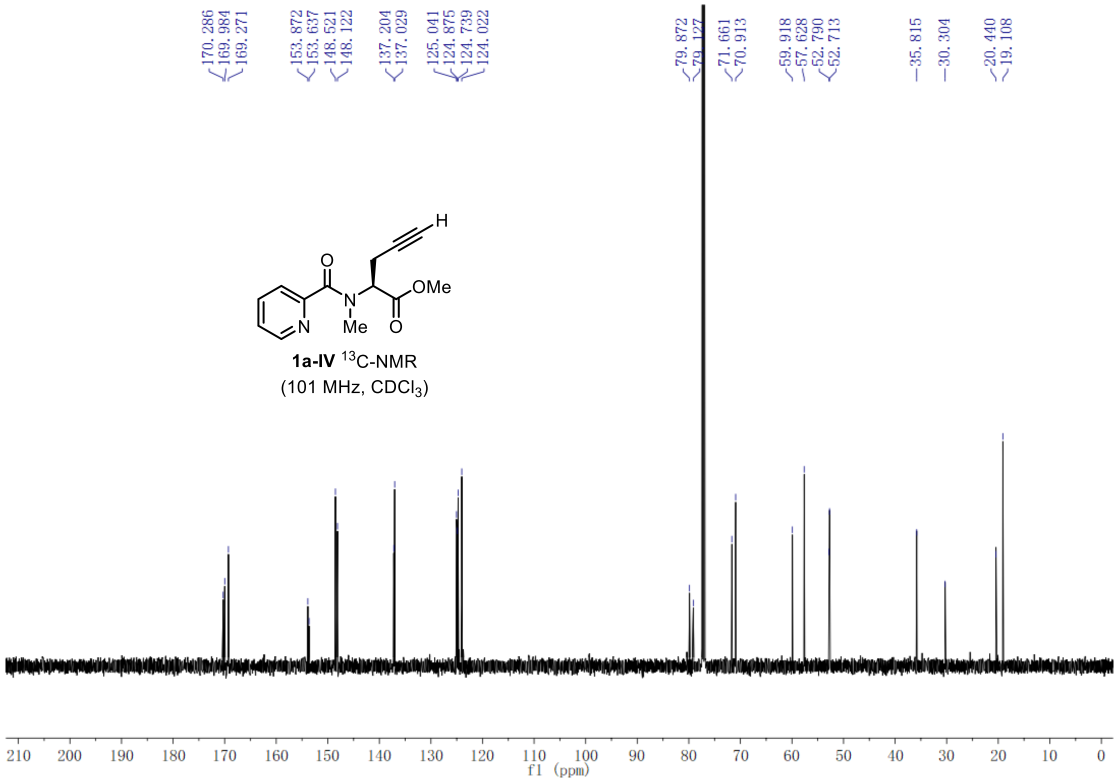
170.286
169.984
169.271
153.872
153.637
148.521
148.122
137.204
137.029
125.041
124.875
124.739
124.022
79.872
71.661
70.913
59.918
57.628
52.790
52.713
35.815
30.304
20.440
19.108
H
O
N
OMe
N
Me
O
1a-IV 13C-NMR
(101 MHz, CDCl3)
210
200
190
180
170
160
150
140
130
120
110
100
90
80
70
60
50
40
30
20
10
0
f1 (ppm)

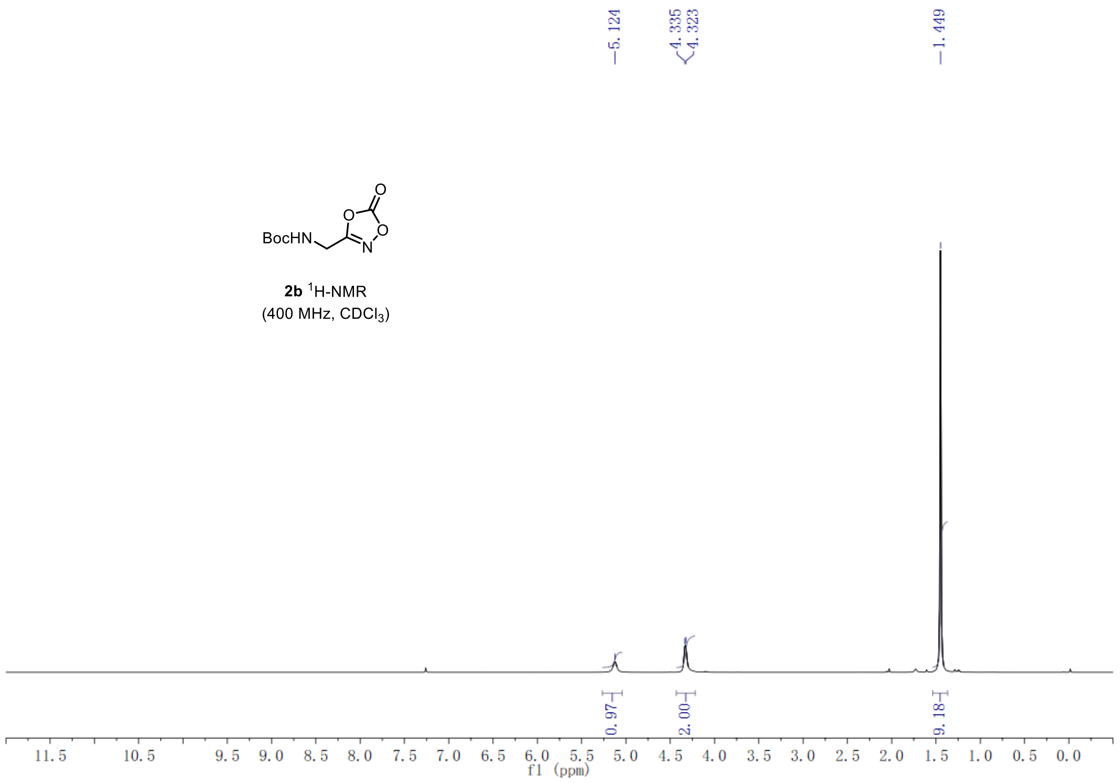

5.124
4.335
4.323
1.449
BocHN
O
O
O
N
2b ¹H-NMR
(400 MHz, CDCl3)
0.97
2.00
9.18
f1 (ppm)

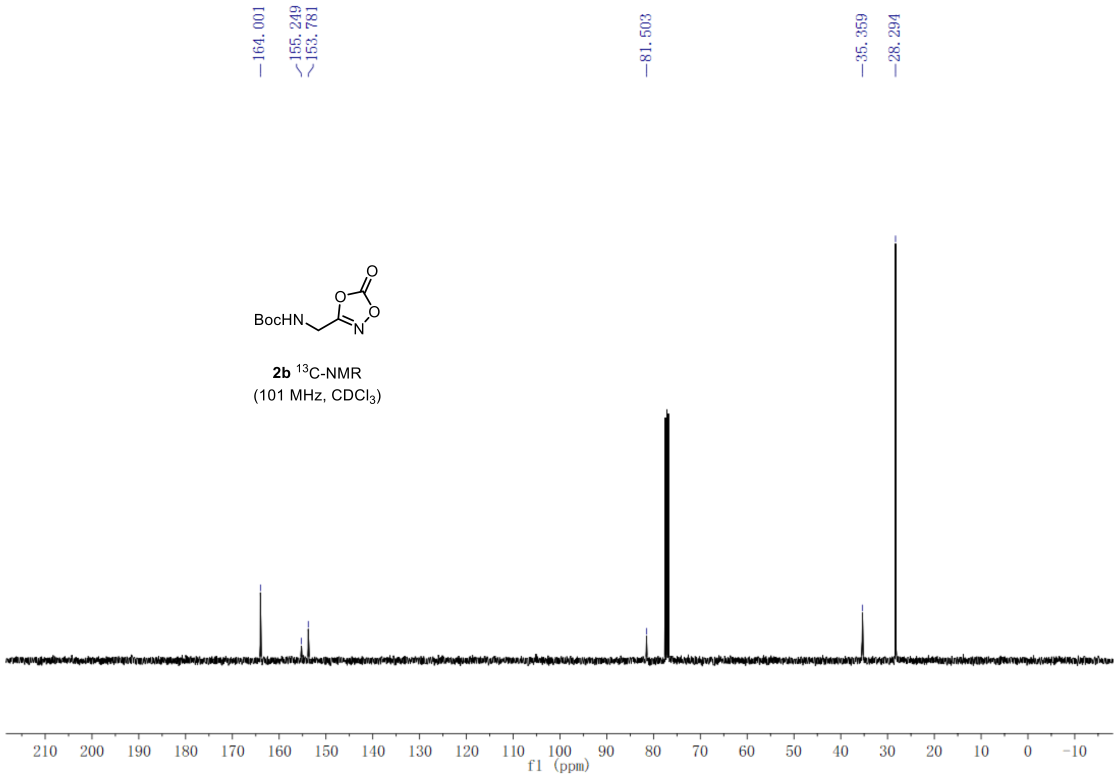

164.001
155.249
153.781
81.503
35.359
28.294
BocHN
O
O
O
N
2b ¹³C-NMR
(101 MHz, CDCl3)
f1 (ppm)

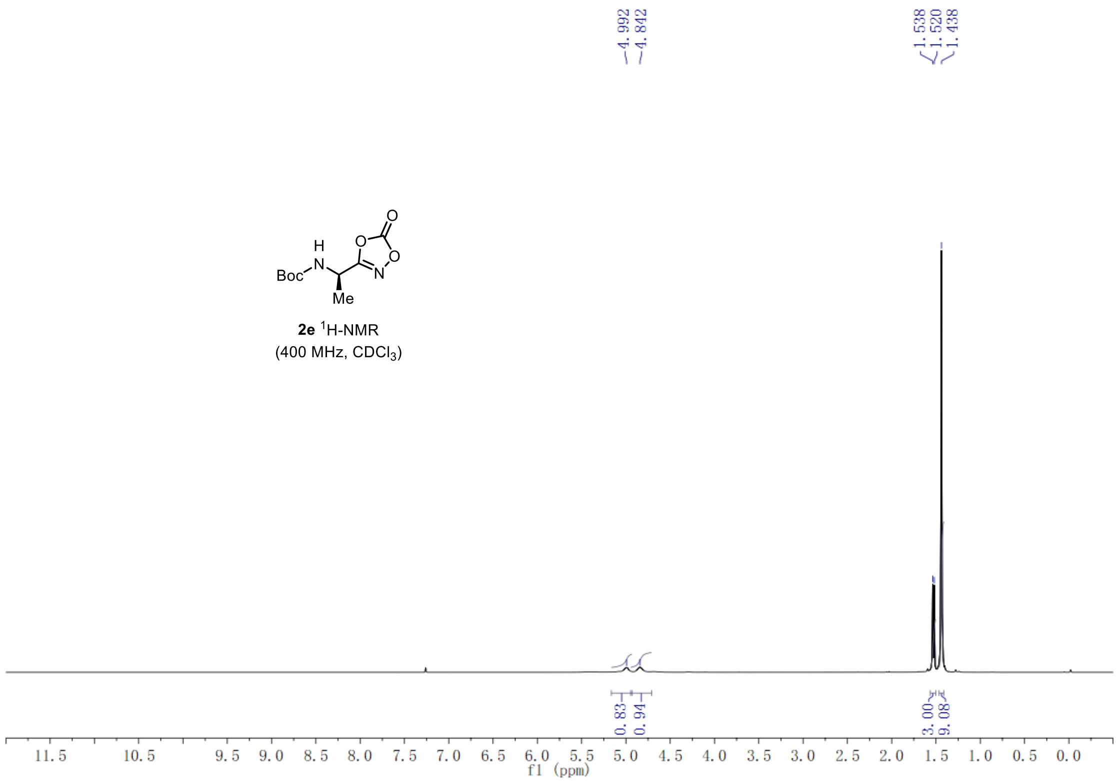
4.992
4.842
1.538
1.520
1.438
H
N
Boc
O
O
O
N
Me
2e 1H-NMR
(400 MHz, CDCl3)
0.83
0.94
3.00
9.08
11.5 10.5 9.5 9.0 8.5 8.0 7.5 7.0 6.5 6.0 5.5 5.0 4.5 4.0 3.5 3.0 2.5 2.0 1.5 1.0 0.5 0.0
f1 (ppm)

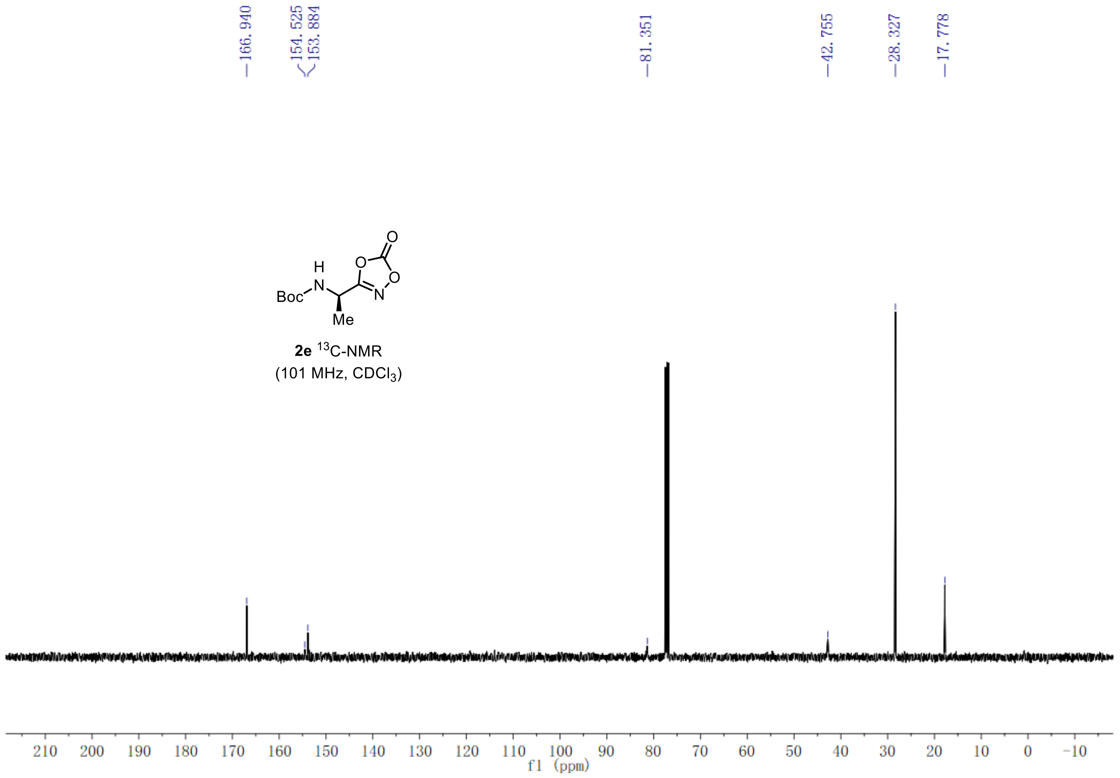
166.940
154.525
153.884
81.351
42.755
28.327
17.778
H
N
Boc
O
O
O
N
Me
2e 13C-NMR
(101 MHz, CDCl3)
210 200 190 180 170 160 150 140 130 120 110 100 90 80 70 60 50 40 30 20 10 0 -10
f1 (ppm)

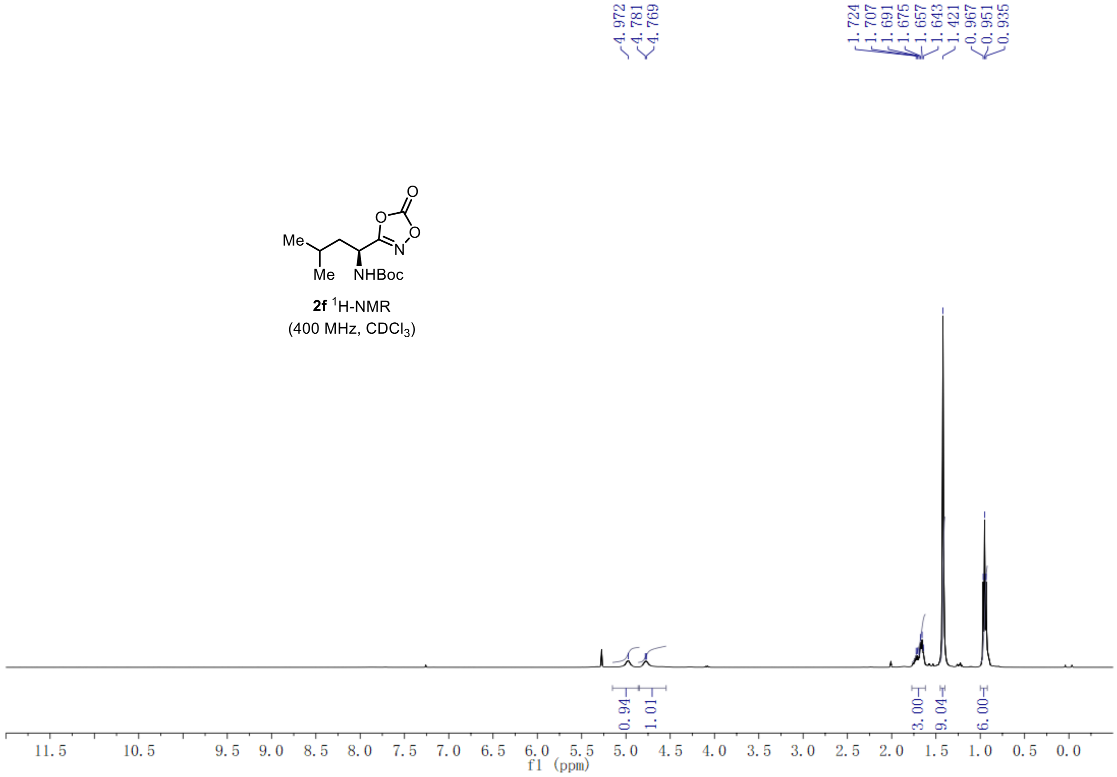
4.972
4.781
4.769
1.724
1.707
1.691
1.675
1.657
1.643
1.421
0.967
0.951
0.935
Me
Me
NHBoc
O
O
O
N
2f ^{1}H-NMR
(400 MHz, $CDCl_3$)
0.94
1.01
3.00
9.04
6.00
11.5 10.5 9.5 9.0 8.5 8.0 7.5 7.0 6.5 6.0 5.5 5.0 4.5 4.0 3.5 3.0 2.5 2.0 1.5 1.0 0.5 0.0
f1 (ppm)

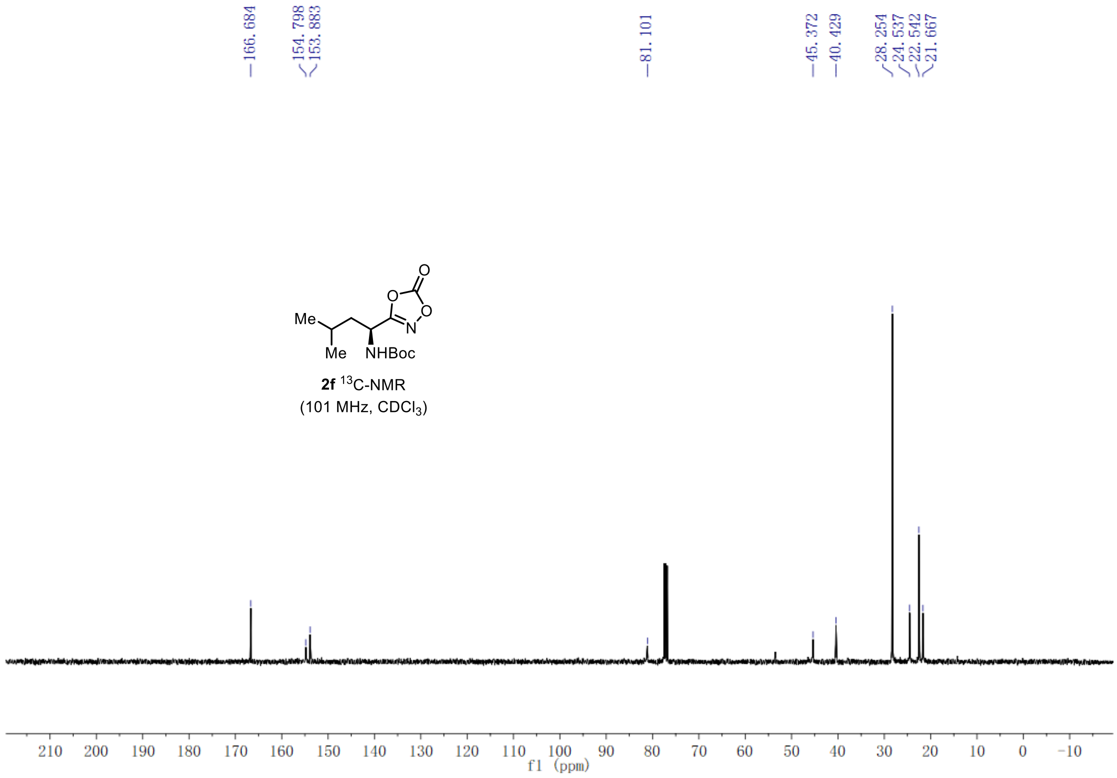
166.684
154.798
153.883
81.101
45.372
40.429
28.254
24.537
22.542
21.667
Me
Me
NHBoc
O
O
O
N
2f ^{13}C-NMR
(101 MHz, $CDCl_3$)
210 200 190 180 170 160 150 140 130 120 110 100 90 80 70 60 50 40 30 20 10 0 -10
f1 (ppm)

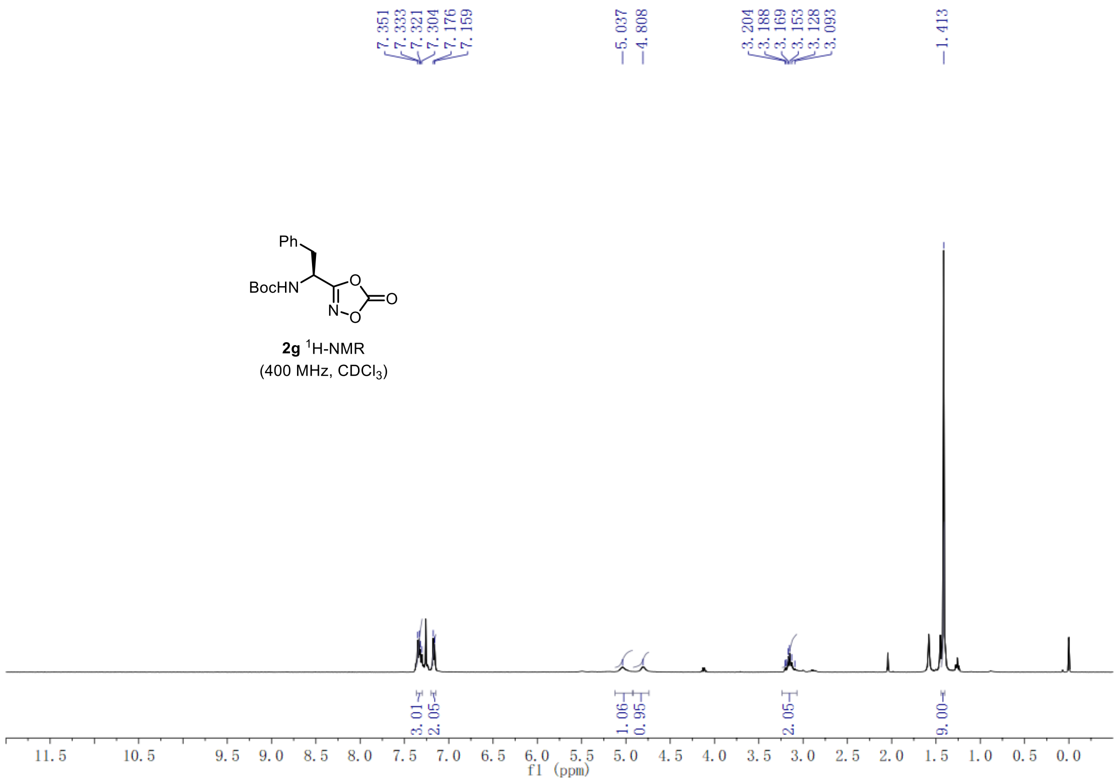

Ph
BocHN
O
N
O
O
2g ¹H-NMR
(400 MHz, CDCl3)
7.351
7.333
7.321
7.304
7.176
7.159
5.037
4.808
3.204
3.188
3.169
3.153
3.128
3.093
1.413
3.01
2.05
1.06
0.95
2.05
9.00
11.5
10.5
9.5
9.0
8.5
8.0
7.5
7.0
6.5
6.0
5.5
5.0
4.5
4.0
3.5
3.0
2.5
2.0
1.5
1.0
0.5
0.0
f1 (ppm)

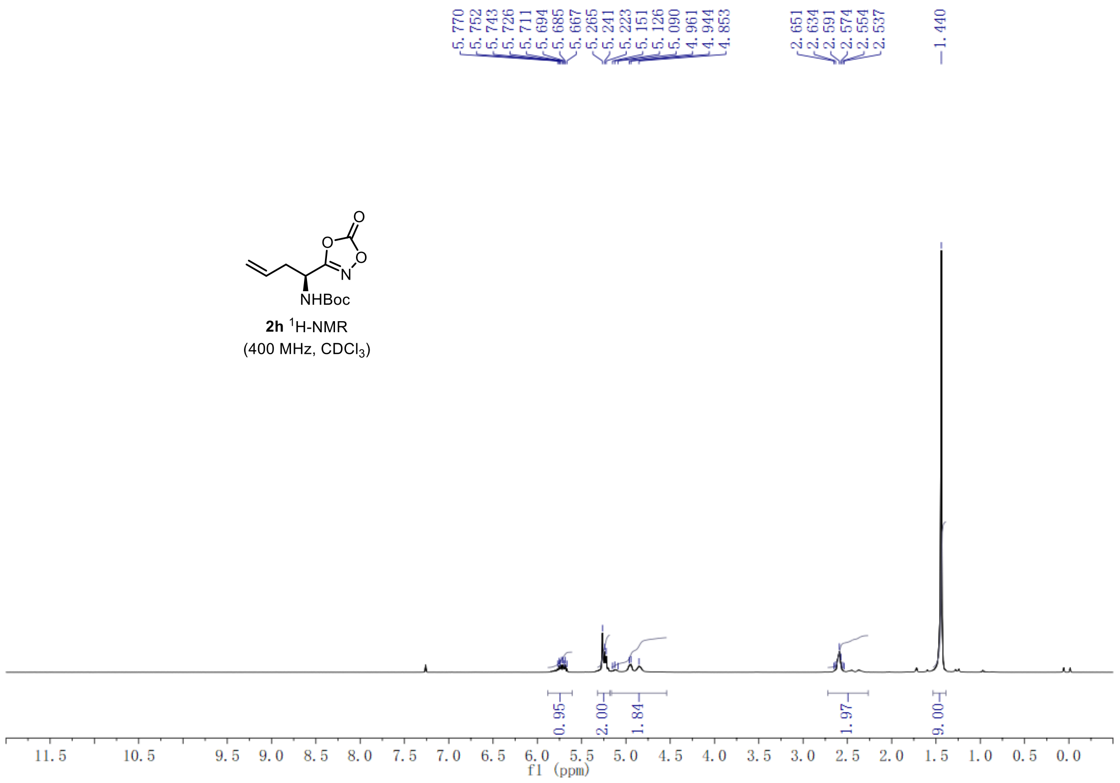

5.770
5.752
5.743
5.726
5.711
5.694
5.685
5.667
5.265
5.241
5.223
5.151
5.126
5.090
4.961
4.944
4.853
2.651
2.634
2.591
2.574
2.554
2.537
1.440
O
O
O
N
NHBoc
2h ¹H-NMR
(400 MHz, CDCl₃)
0.95
2.00
1.84
1.97
9.00
11.5
10.5
9.5
9.0
8.5
8.0
7.5
7.0
6.5
6.0
5.5
5.0
4.5
4.0
3.5
3.0
2.5
2.0
1.5
1.0
0.5
0.0
f1 (ppm)

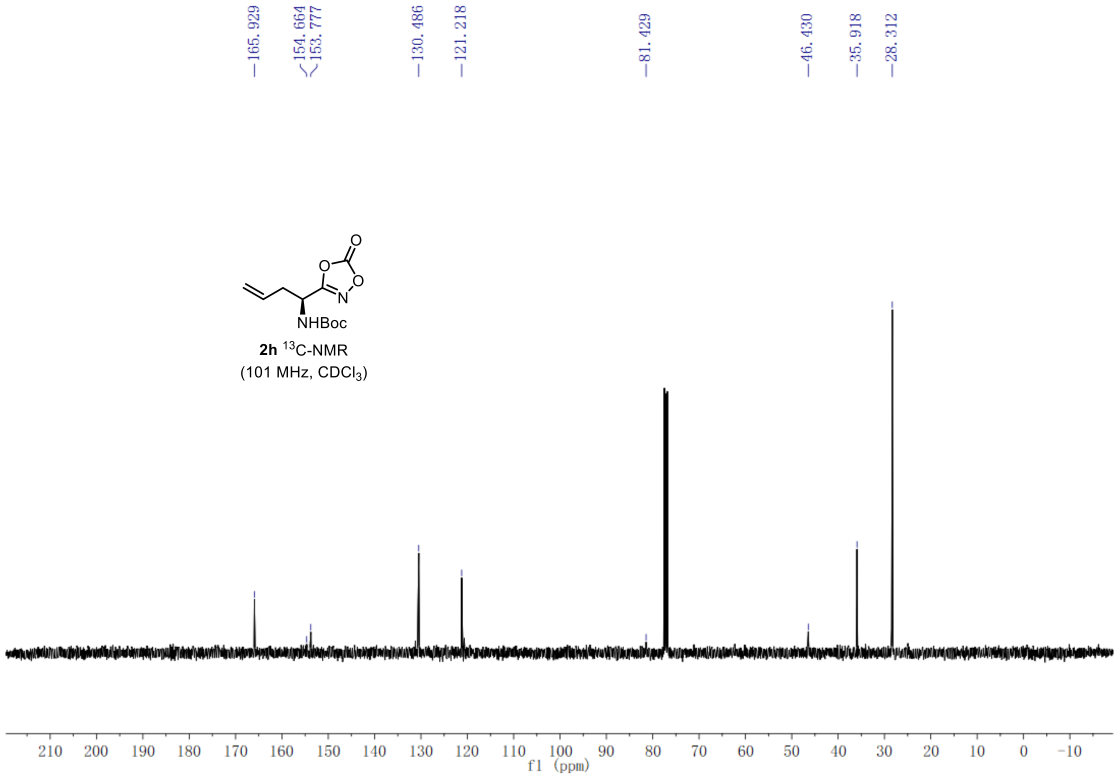

165.929
154.664
153.777
130.486
121.218
81.429
46.430
35.918
28.312
O
O
O
N
NHBoc
2h ¹³C-NMR
(101 MHz, CDCl₃)
210
200
190
180
170
160
150
140
130
120
110
100
90
80
70
60
50
40
30
20
10
0
-10
f1 (ppm)

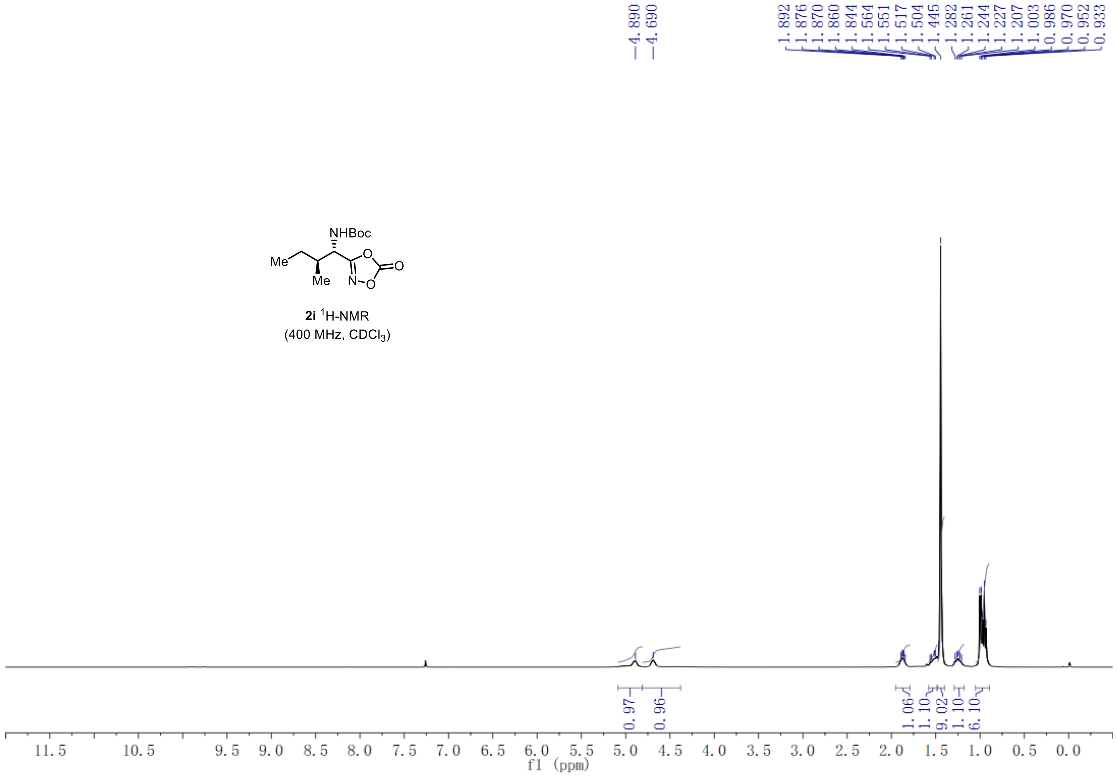
4.890
4.690
1.892
1.876
1.870
1.860
1.844
1.564
1.551
1.517
1.504
1.445
1.282
1.261
1.244
1.227
1.207
1.003
0.986
0.970
0.952
0.933
NHBoc
Me
Me
O
N
O
O
2i ¹H-NMR
(400 MHz, CDCl3)
0.97
0.96
1.06
1.10
9.02
1.10
6.10
11.5
10.5
9.5
9.0
8.5
8.0
7.5
7.0
6.5
6.0
5.5
5.0
4.5
4.0
3.5
3.0
2.5
2.0
1.5
1.0
0.5
0.0
f1 (ppm)

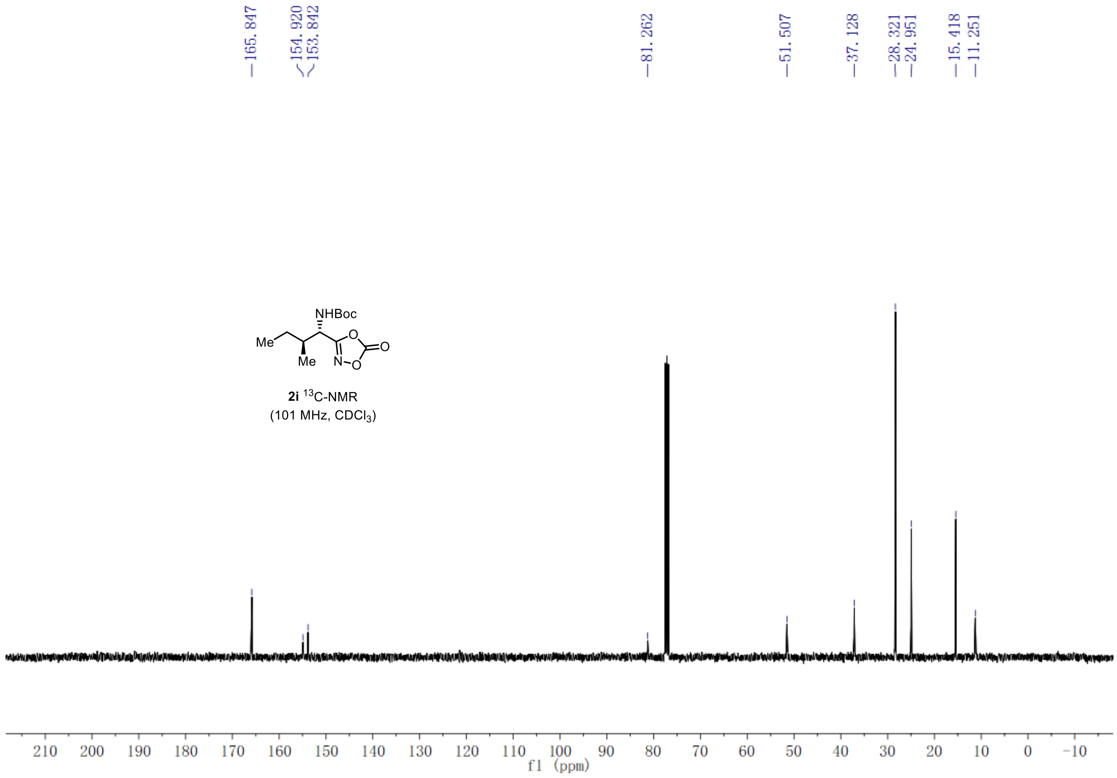
165.847
154.920
153.842
81.262
51.507
37.128
28.321
24.951
15.418
11.251
NHBoc
Me
Me
O
N
O
O
2i ¹³C-NMR
(101 MHz, CDCl3)
210
200
190
180
170
160
150
140
130
120
110
100
90
80
70
60
50
40
30
20
10
0
-10
f1 (ppm)

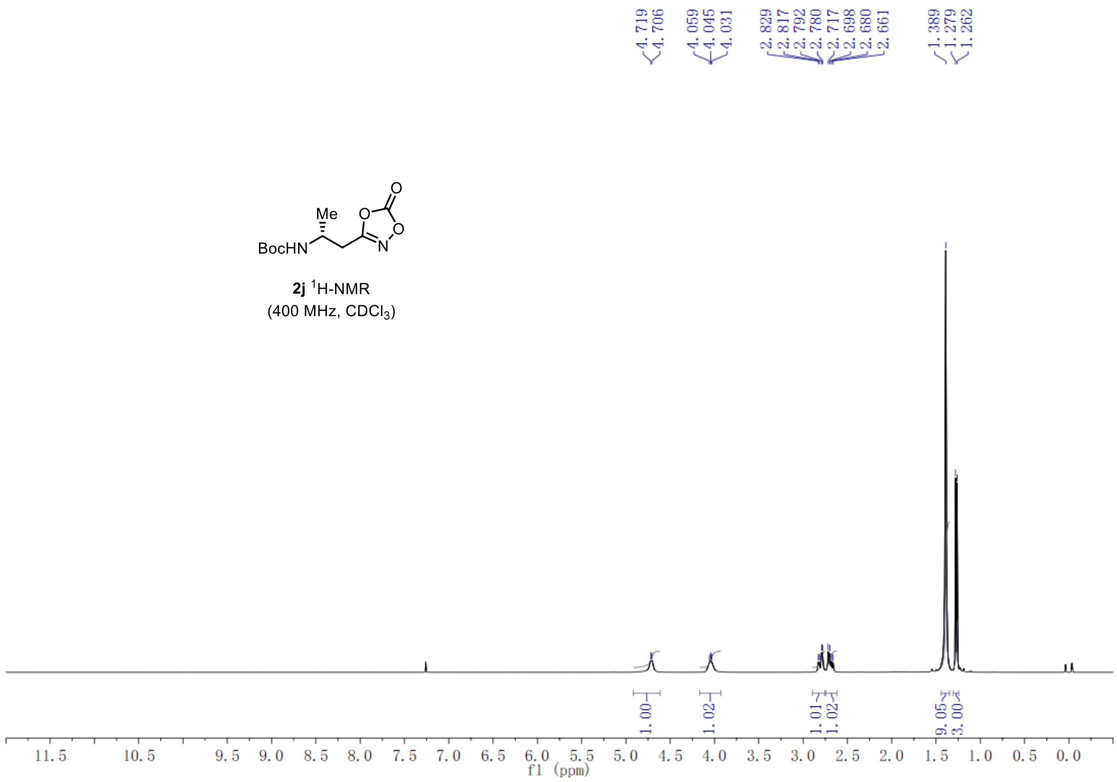

2j ¹H-NMR
(400 MHz, $CDCl_3$)

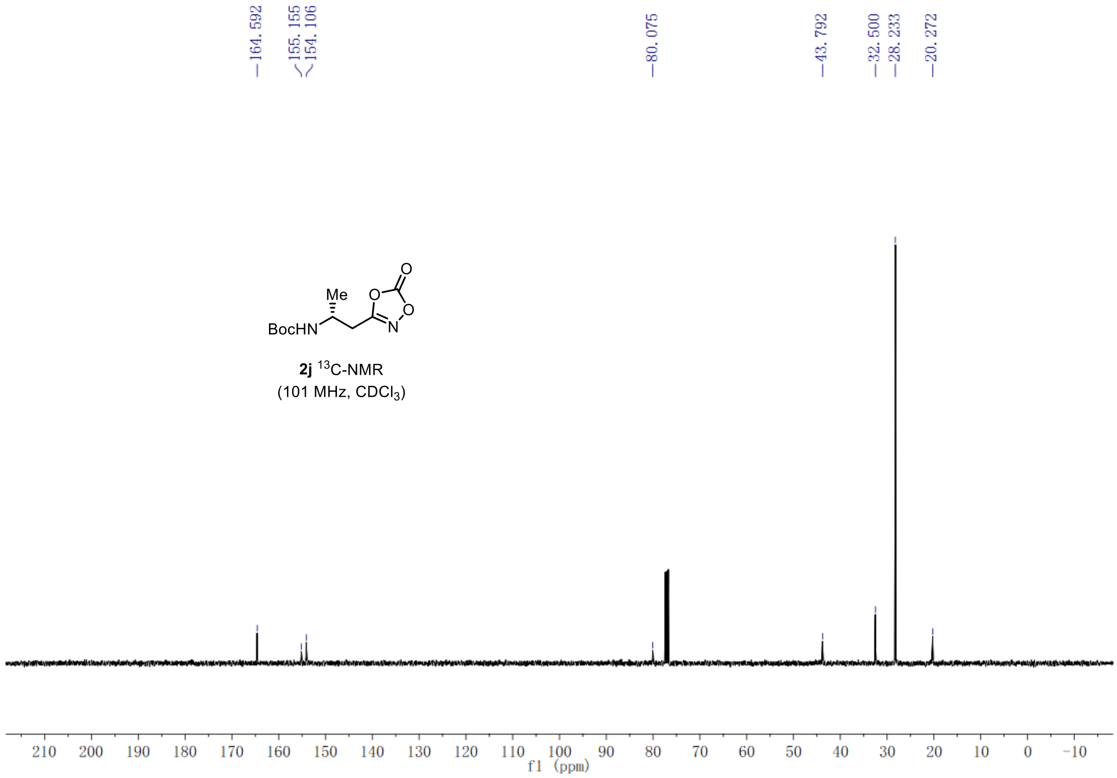

2j ¹³C-NMR
(101 MHz, $CDCl_3$)

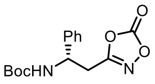

2k ^{1}H-NMR
(400 MHz, $CDCl_3$)

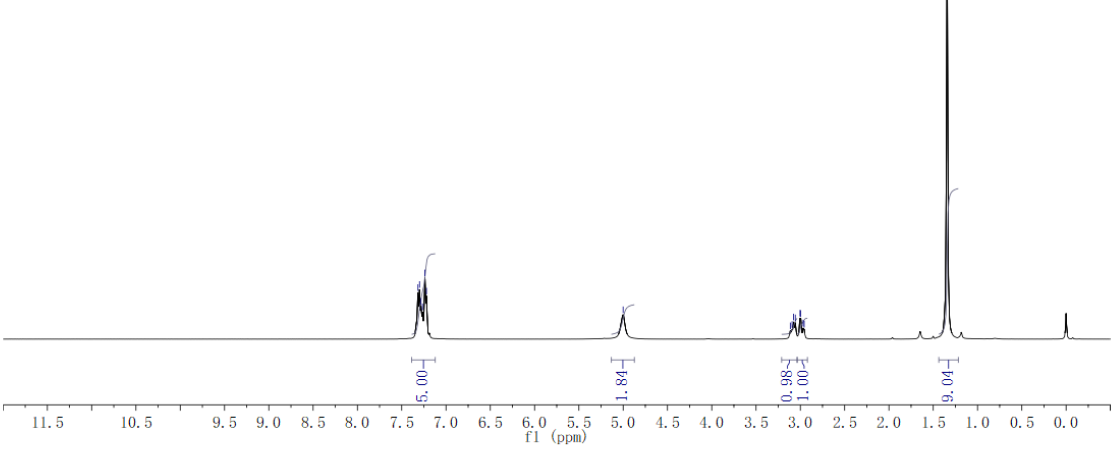

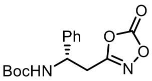

2k ^{13}C-NMR
(101 MHz, $CDCl_3$)

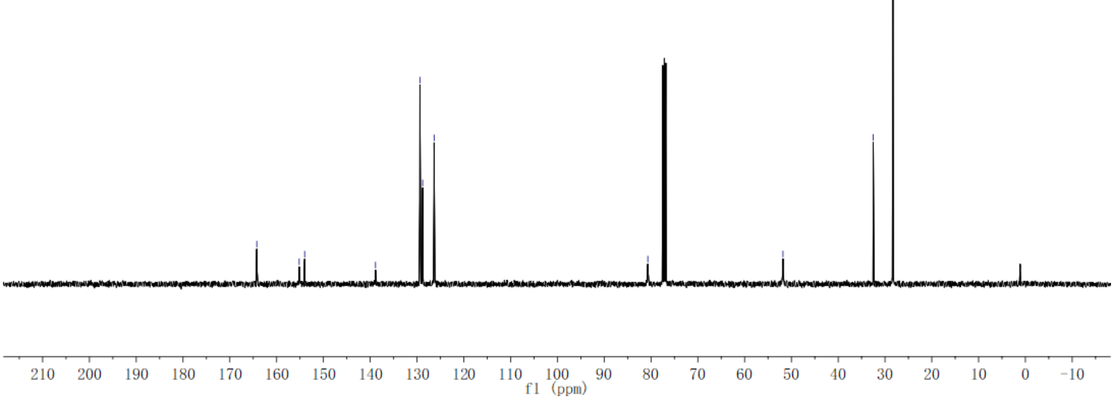

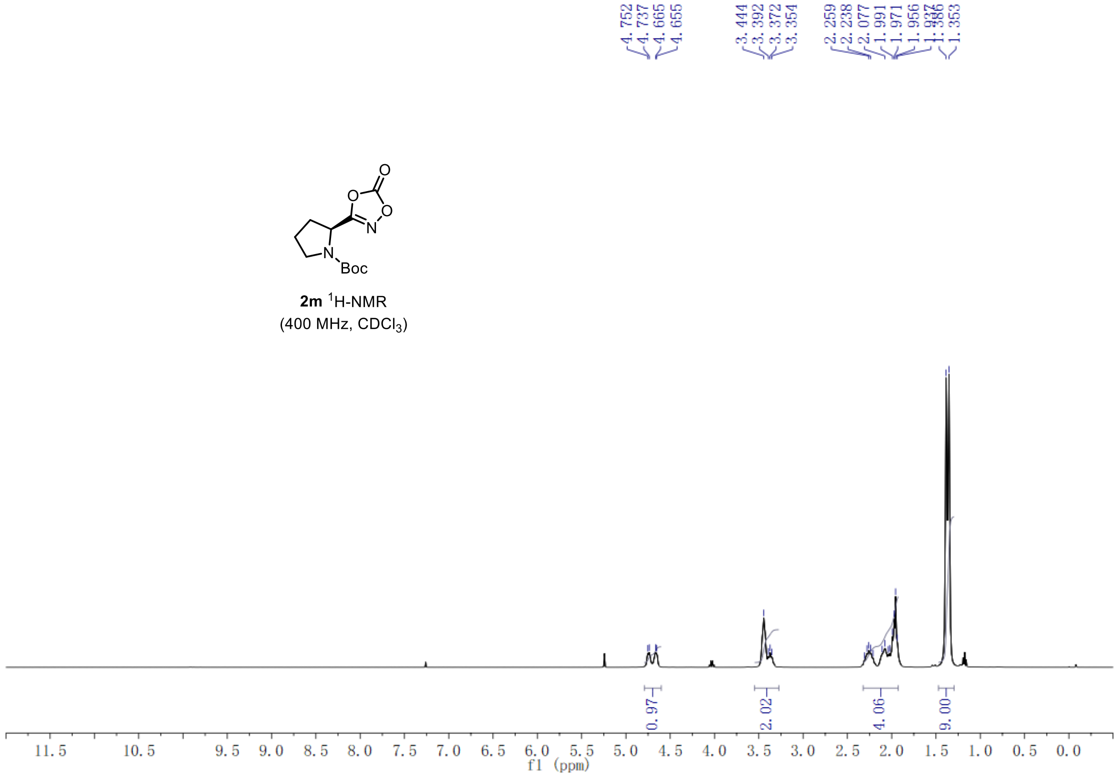

2m ^{1}H-NMR
(400 MHz, $CDCl_3$)

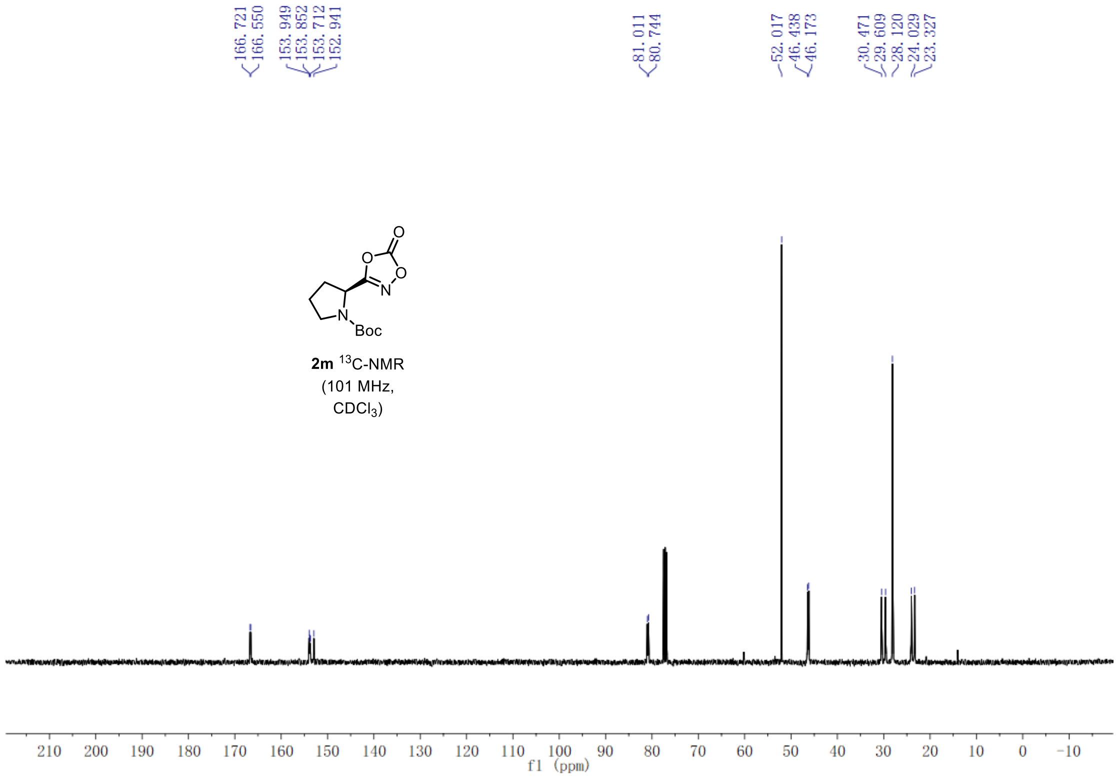

2m ^{13}C-NMR
(101 MHz,
$CDCl_3$)

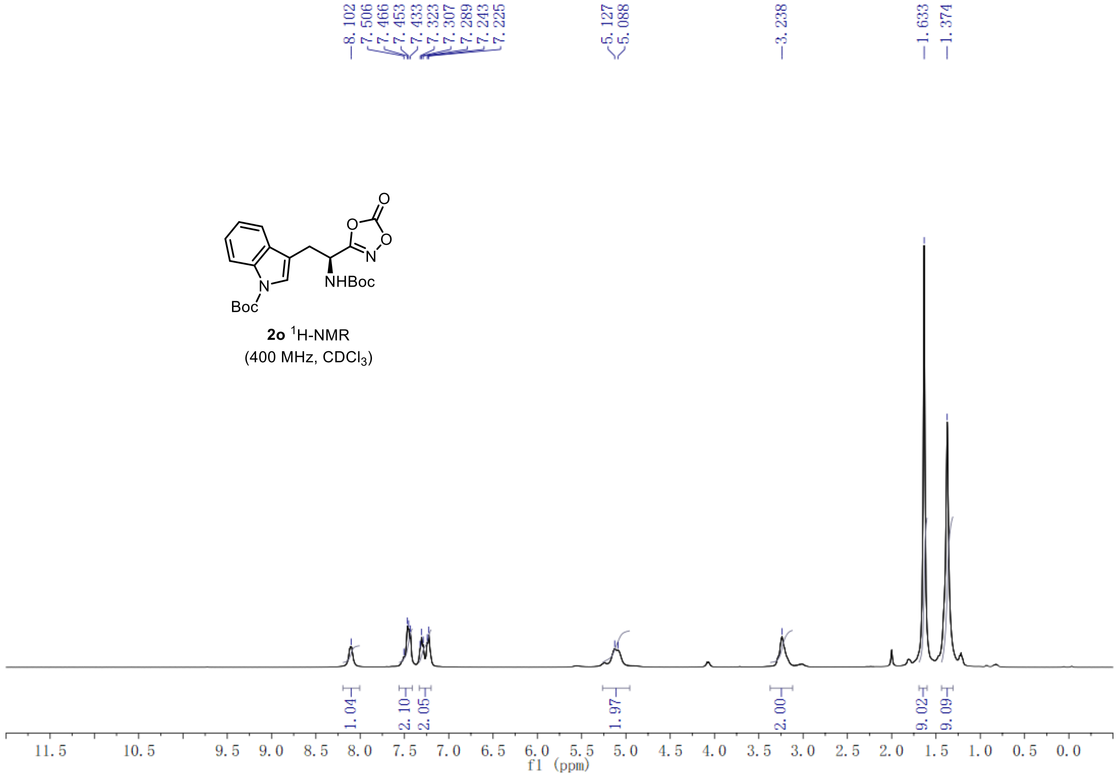
8.102
7.506
7.466
7.453
7.433
7.323
7.307
7.289
7.243
7.225
5.127
5.088
3.238
1.633
1.374
NHBoc
Boc
2o 1H-NMR
(400 MHz, CDCl3)
1.04
2.10
2.05
1.97
2.00
9.02
9.09
f1 (ppm)

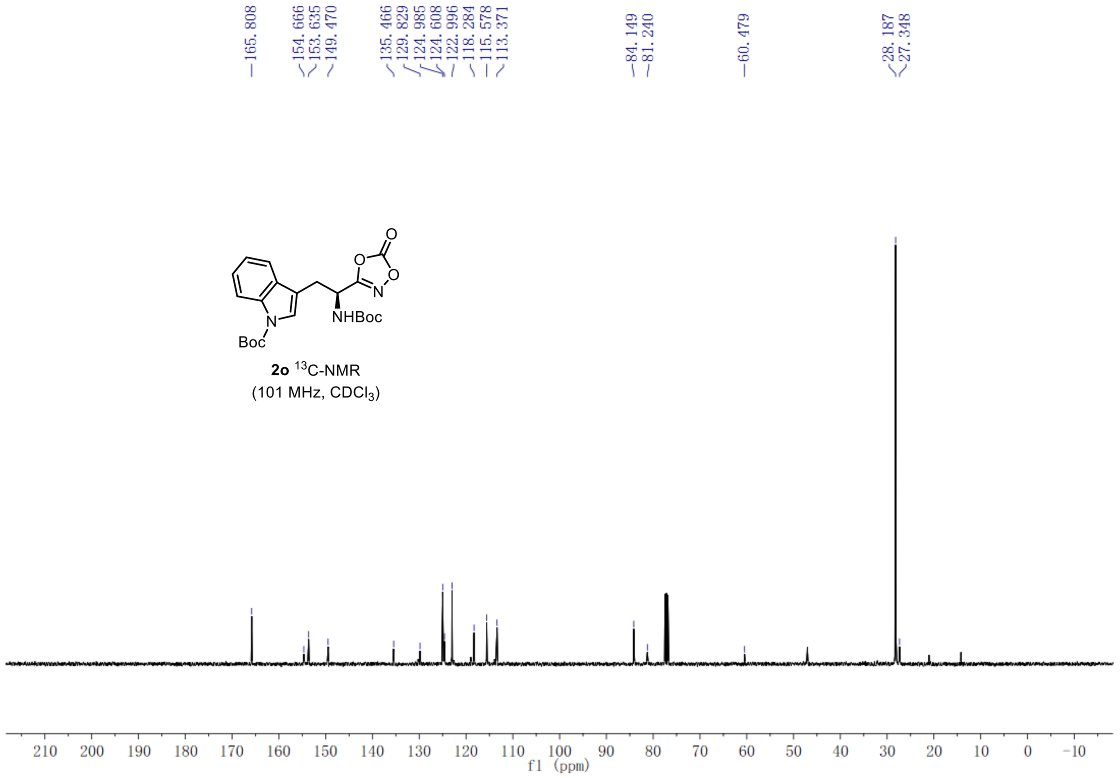
165.808
154.666
153.635
149.470
135.466
129.829
124.985
124.608
122.996
118.284
115.578
113.371
84.149
81.240
60.479
28.187
27.348
NHBoc
Boc
2o 13C-NMR
(101 MHz, CDCl3)
f1 (ppm)

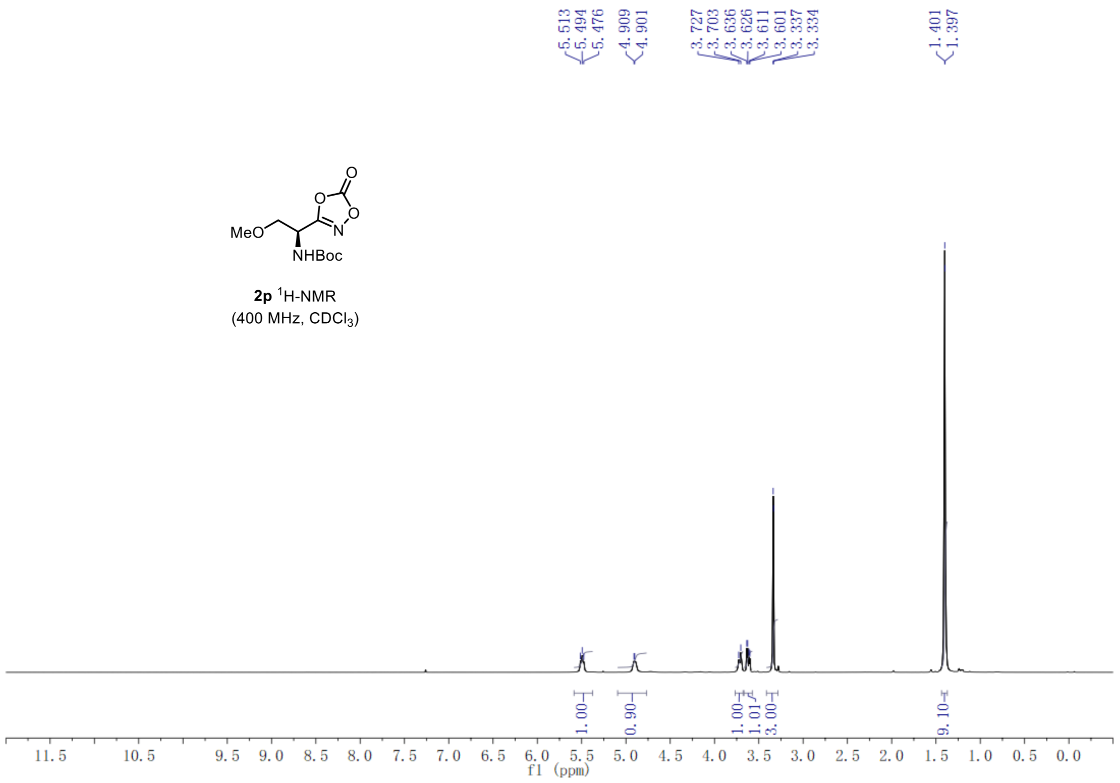

2p ^{1}H-NMR
(400 MHz, $CDCl_3$)

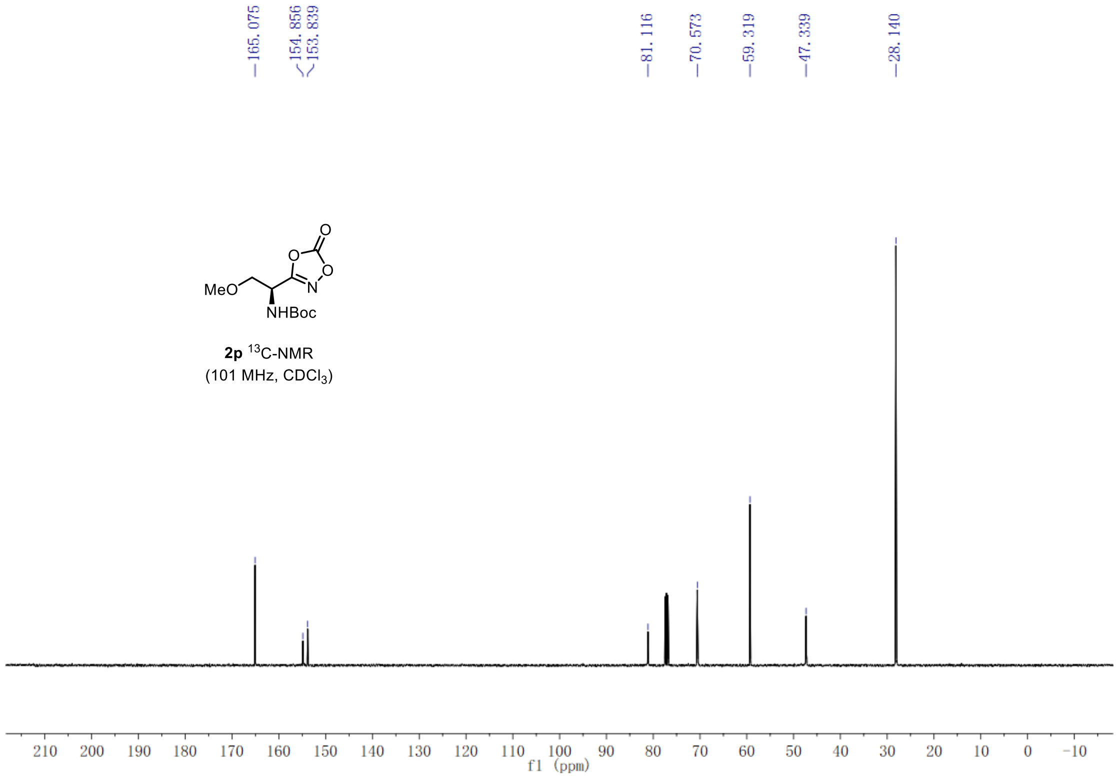

2p ^{13}C-NMR
(101 MHz, $CDCl_3$)

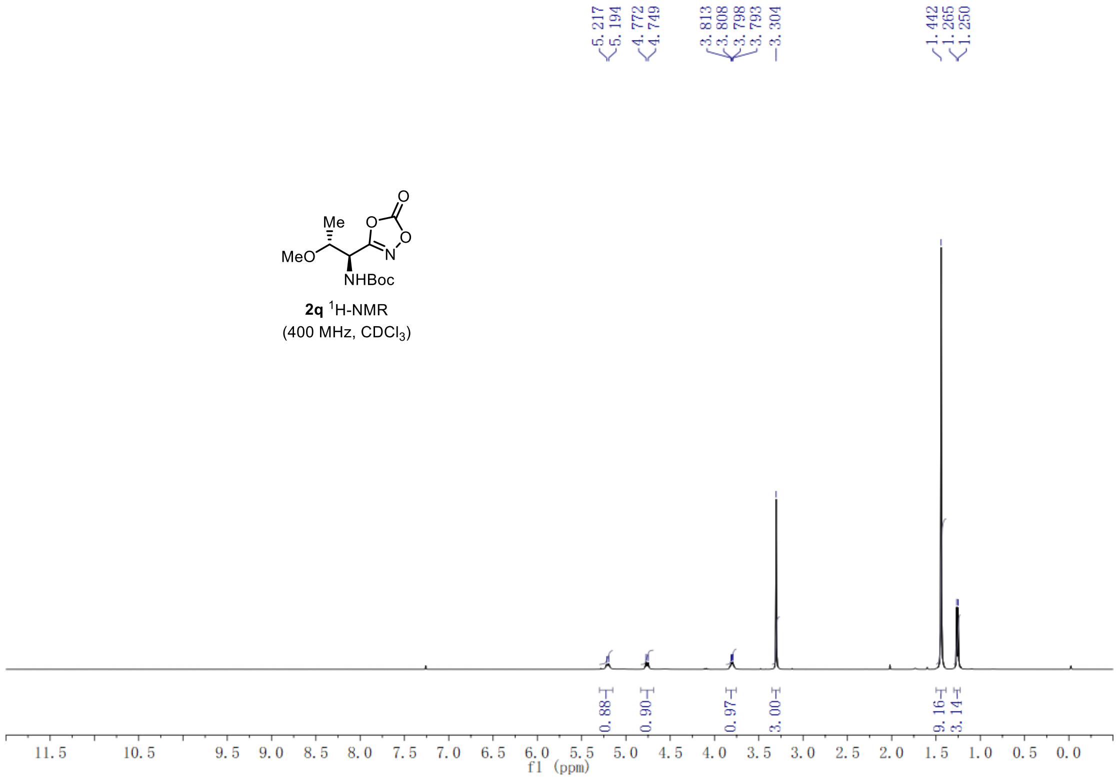
5.217
5.194
4.772
4.749
3.813
3.808
3.798
3.793
3.304
1.442
1.265
1.250
Me
MeO
O
O
O
N
NHBoc
2q ^{1}H-NMR
(400 MHz, $CDCl_3$)
0.88
0.90
0.97
3.00
9.16
3.14
f1 (ppm)

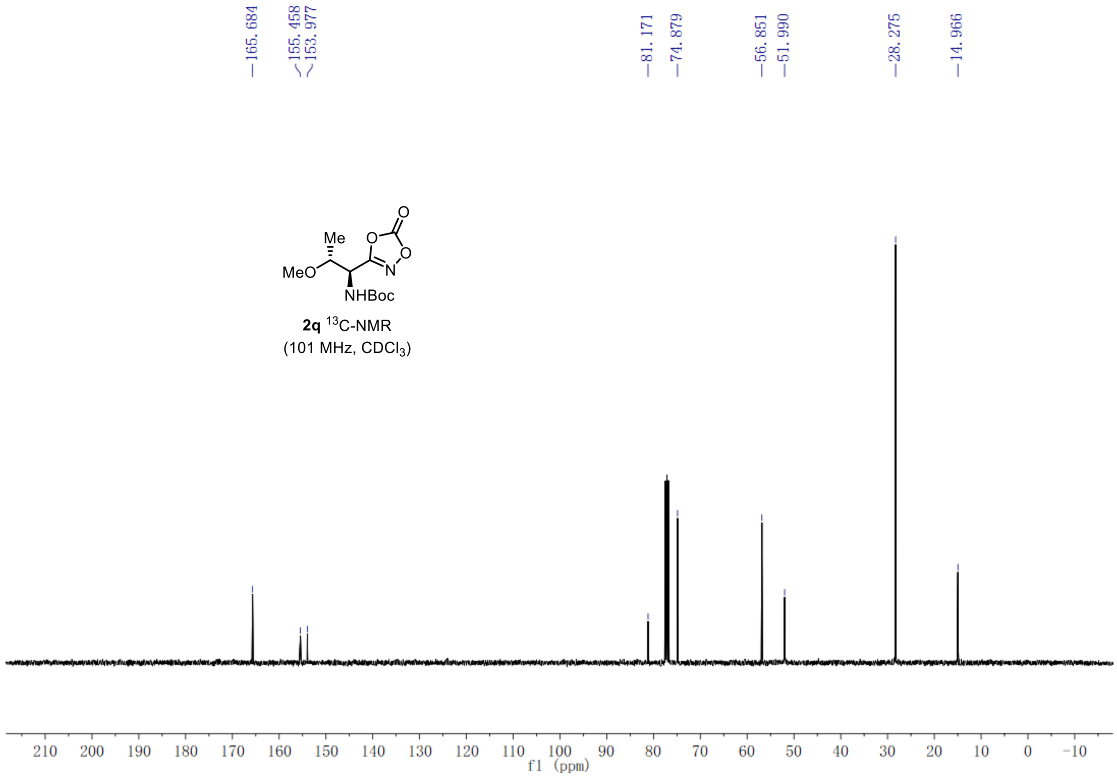
165.684
155.458
153.977
81.171
74.879
56.851
51.990
28.275
14.966
Me
MeO
O
O
O
N
NHBoc
2q ^{13}C-NMR
(101 MHz, $CDCl_3$)
f1 (ppm)

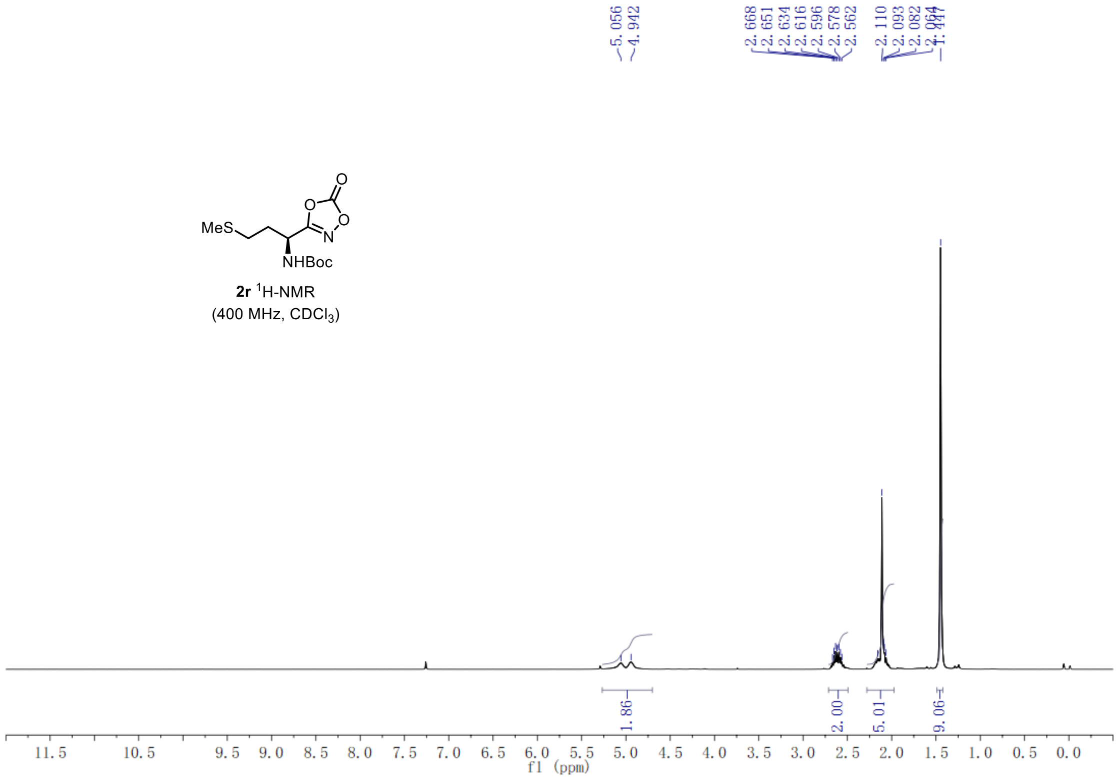
5.056
4.942
2.668
2.651
2.634
2.616
2.596
2.578
2.562
2.110
2.093
2.082
MeS
NHBoc
2r 1H-NMR
(400 MHz, CDCl3)
1.86
2.00
5.01
9.06
f1 (ppm)

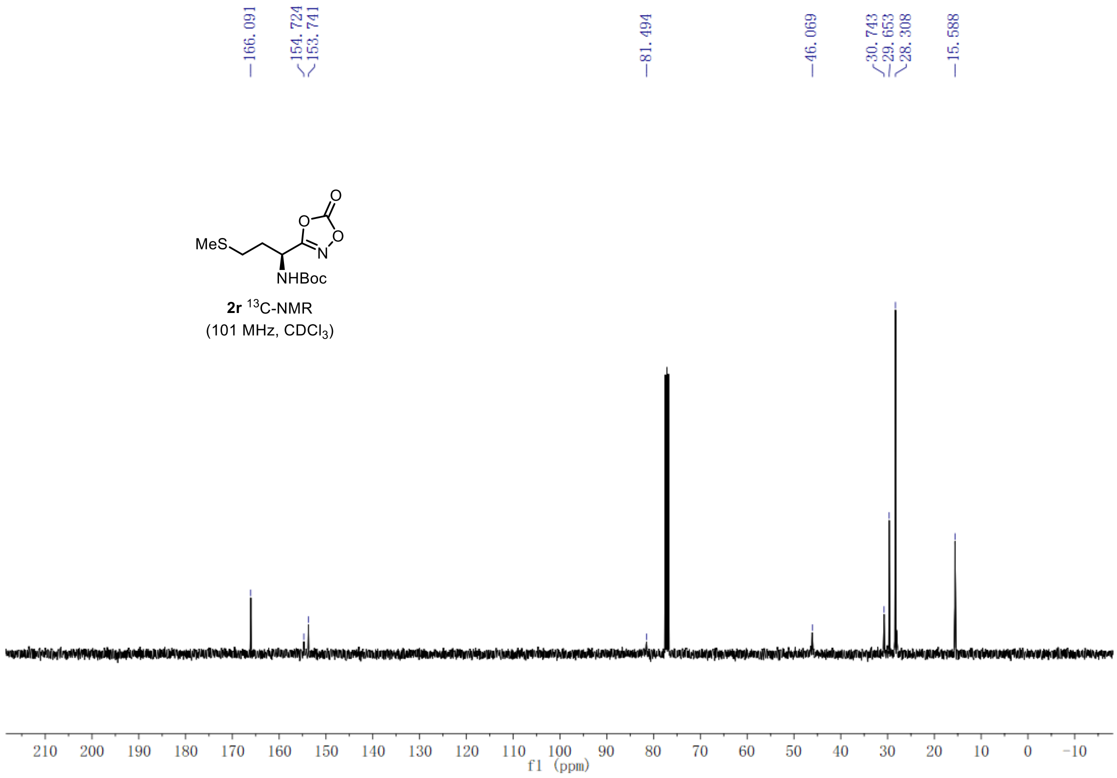
166.091
154.724
153.741
81.494
46.069
30.743
29.653
28.308
15.588
MeS
NHBoc
2r 13C-NMR
(101 MHz, CDCl3)
f1 (ppm)

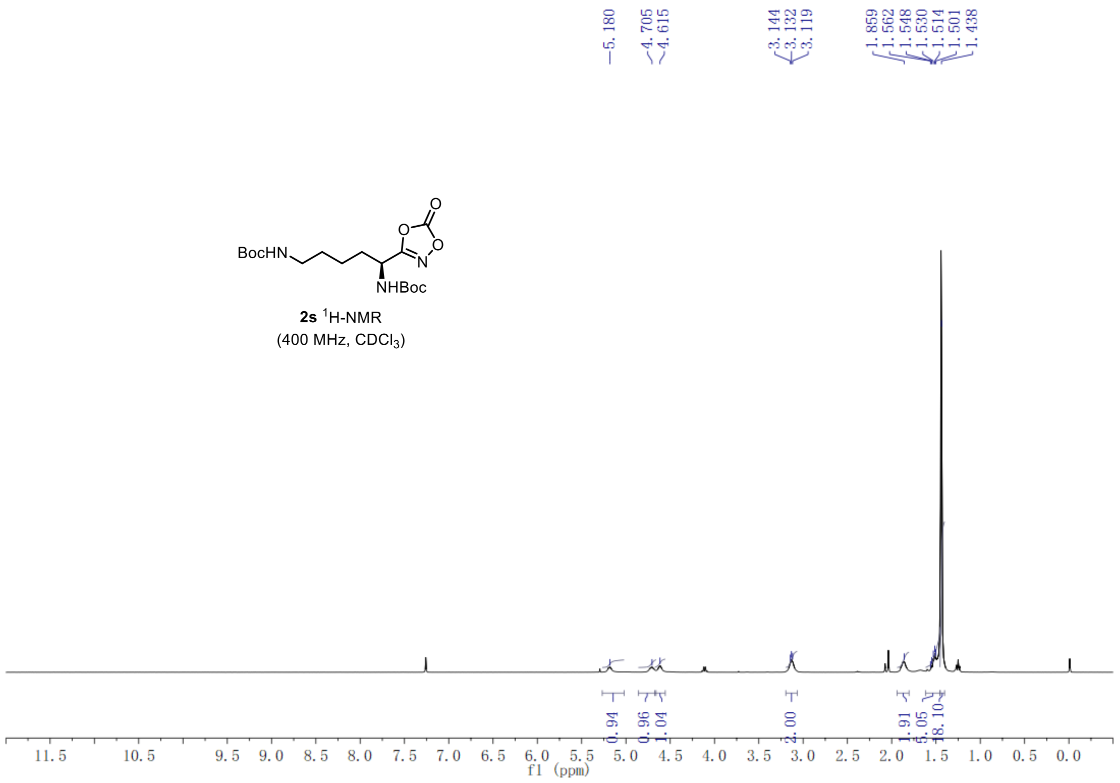

5.180
4.705
4.615
3.144
3.132
3.119
1.859
1.562
1.548
1.530
1.514
1.501
1.438
BocHN
NHBoc
O
O
O
N
2s ¹H-NMR
(400 MHz, CDCl3)
0.94
0.96
1.04
2.00
1.91
5.05
18.10
11.5
10.5
9.5
9.0
8.5
8.0
7.5
7.0
6.5
6.0
5.5
5.0
4.5
4.0
3.5
3.0
2.5
2.0
1.5
1.0
0.5
0.0
f1 (ppm)

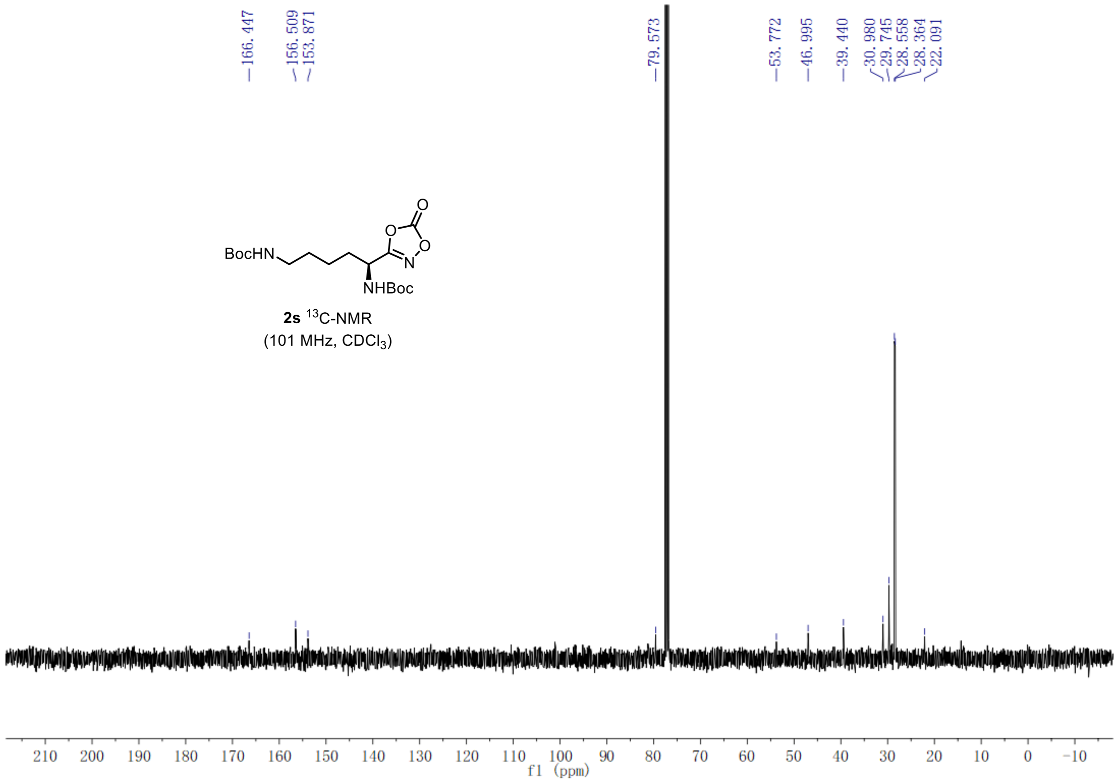

166.447
156.509
153.871
79.573
53.772
46.995
39.440
30.980
29.745
28.558
28.364
22.091
BocHN
NHBoc
O
O
O
N
2s ¹³C-NMR
(101 MHz, CDCl3)
210
200
190
180
170
160
150
140
130
120
110
100
90
80
70
60
50
40
30
20
10
0
-10
f1 (ppm)

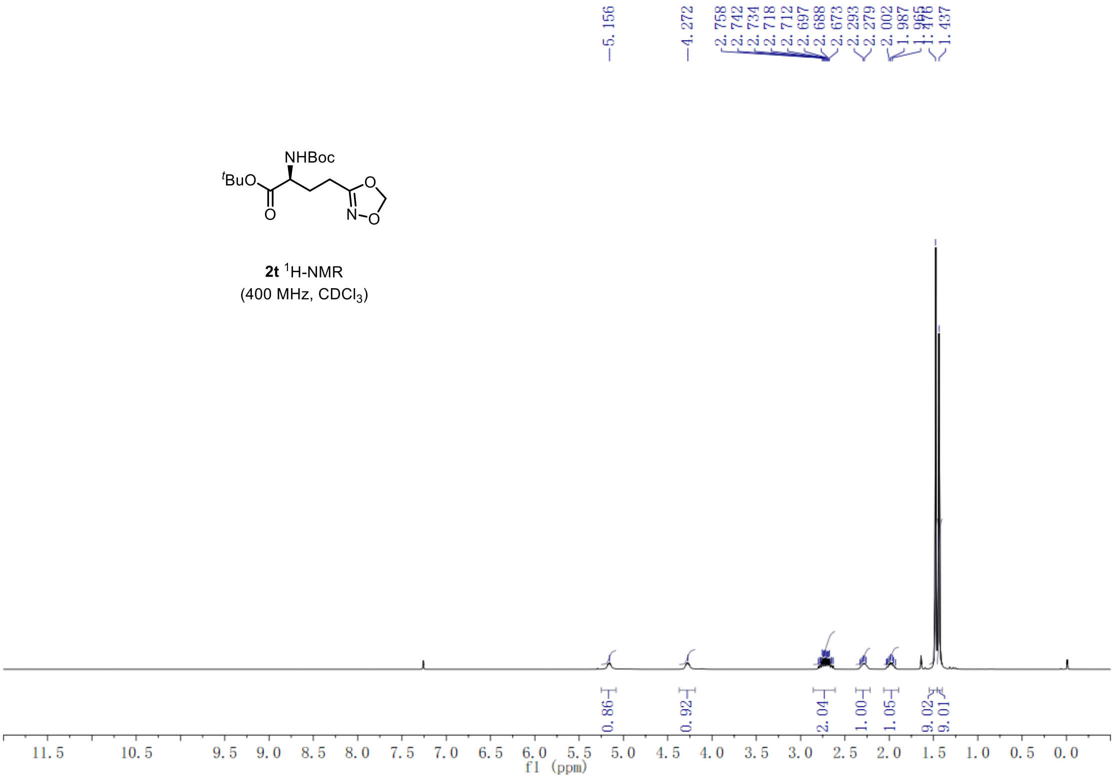
5.156
4.272
2.758
2.742
2.734
2.718
2.712
2.697
2.688
2.673
2.293
2.279
2.002
1.987
1.437
NHBoc
tBuO
O
O
N-O
2t 1H-NMR
(400 MHz, CDCl3)
0.86
0.92
2.04
1.00
1.05
9.02
9.01
11.5 10.5 9.5 9.0 8.5 8.0 7.5 7.0 6.5 6.0 5.5 5.0 4.5 4.0 3.5 3.0 2.5 2.0 1.5 1.0 0.5 0.0
f1 (ppm)

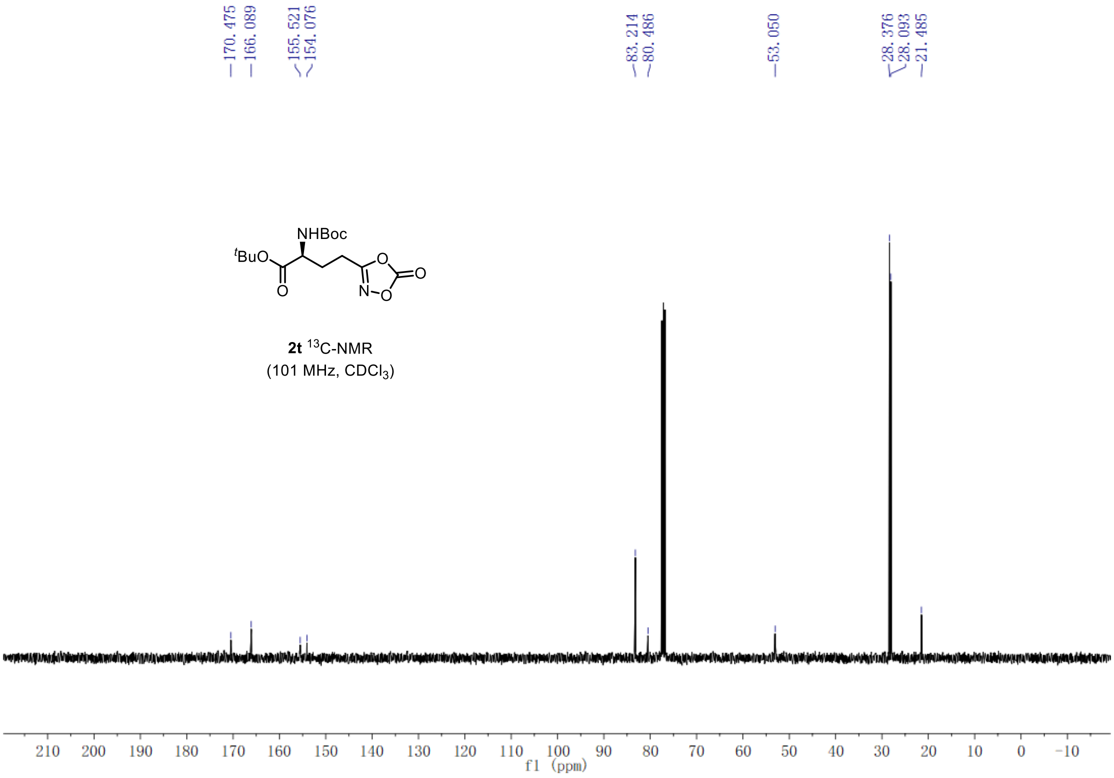
170.475
166.089
155.521
154.076
83.214
80.486
53.050
28.376
28.093
21.485
NHBoc
tBuO
O
O
O
N-O
2t 13C-NMR
(101 MHz, CDCl3)
210 200 190 180 170 160 150 140 130 120 110 100 90 80 70 60 50 40 30 20 10 0 -10
f1 (ppm)

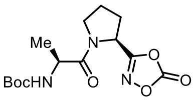

2u ^{1}H-NMR
(400 MHz, $CDCl_3$)

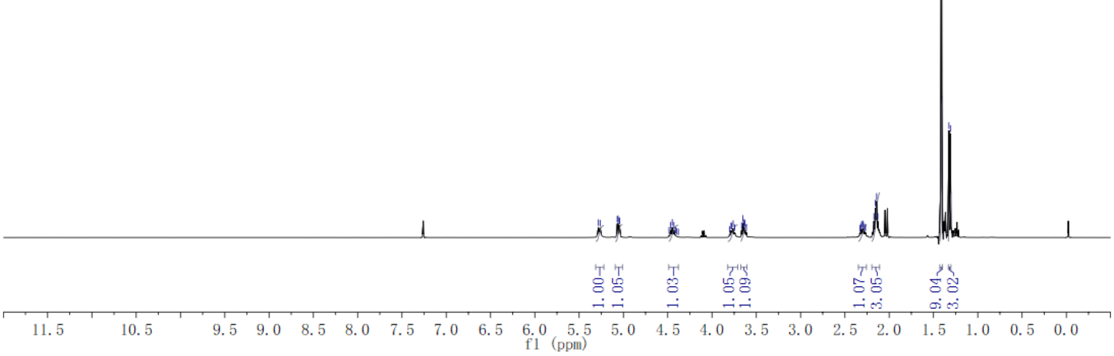

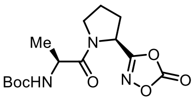

2u ^{13}C-NMR
(101 MHz, $CDCl_3$)

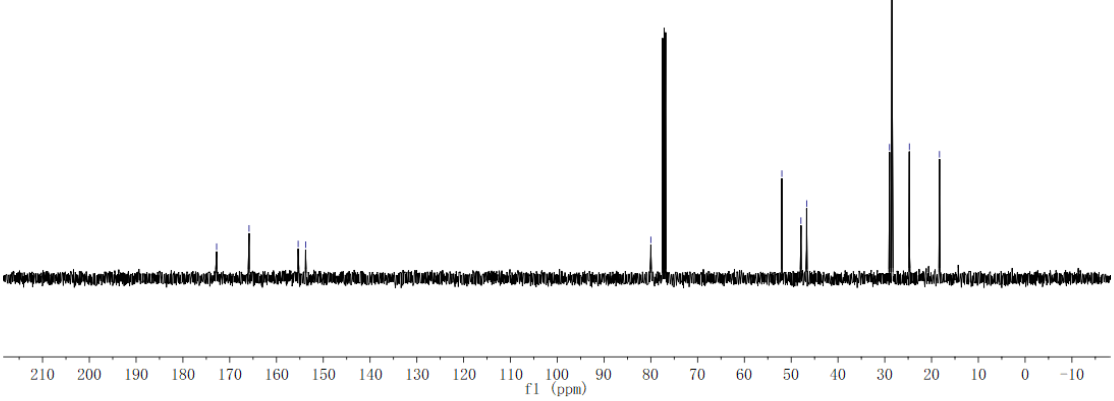

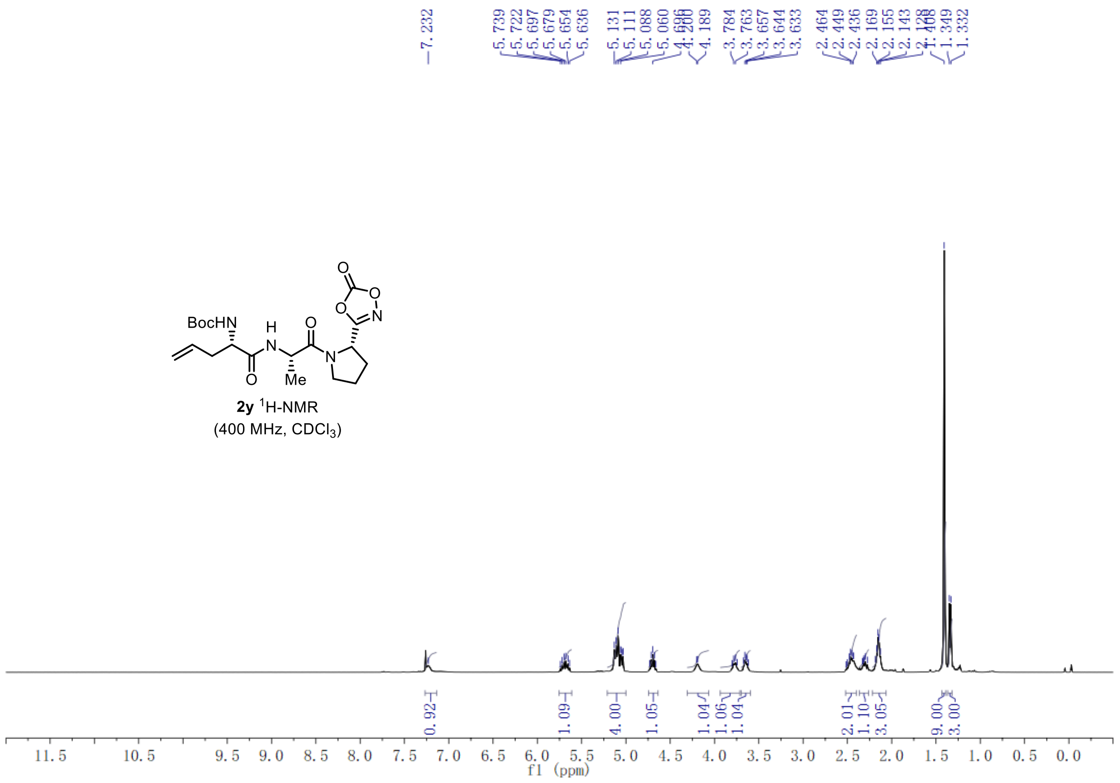
BocHN
H
N
O
Me
2y ¹H-NMR
(400 MHz, CDCl₃)
f1 (ppm)

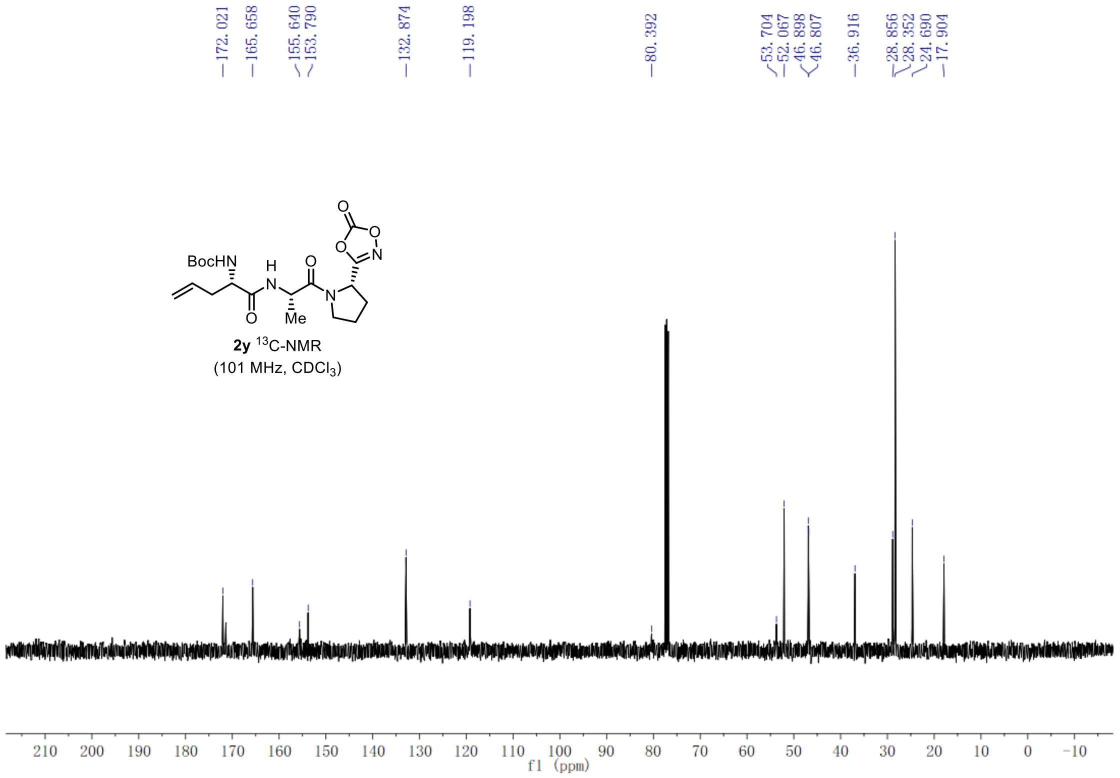
BocHN
H
N
O
Me
2y ¹³C-NMR
(101 MHz, CDCl₃)
f1 (ppm)

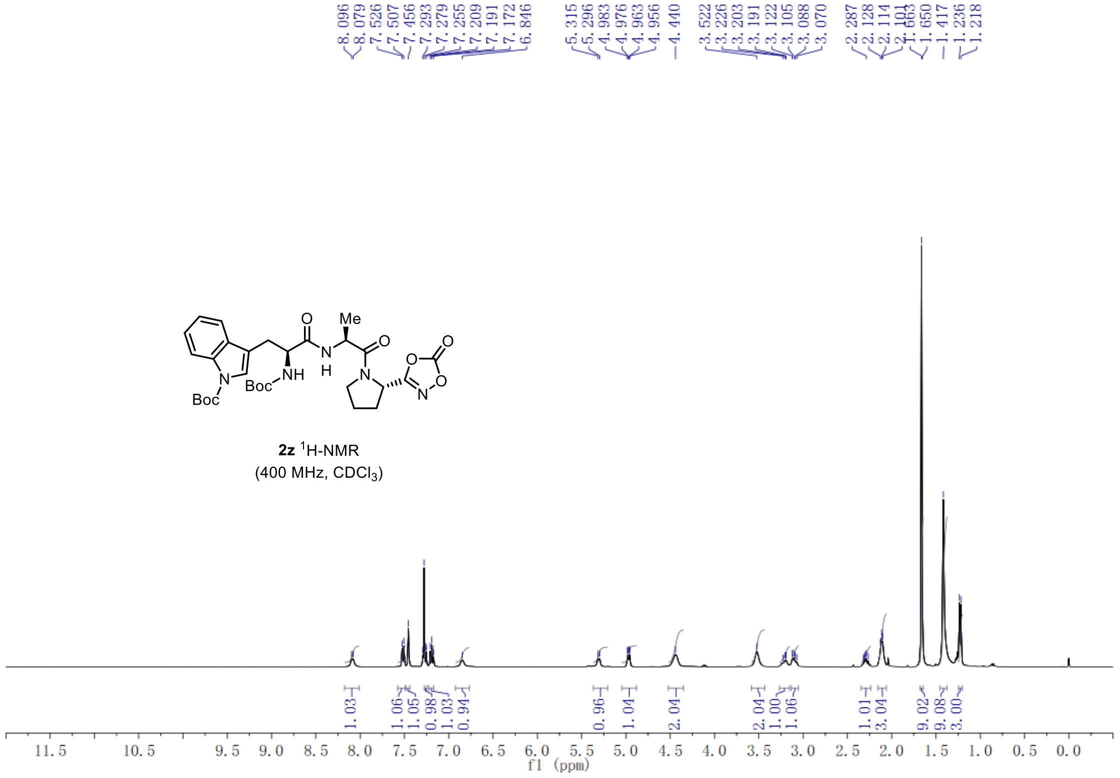

2z ^{1}H-NMR
(400 MHz, $CDCl_3$)

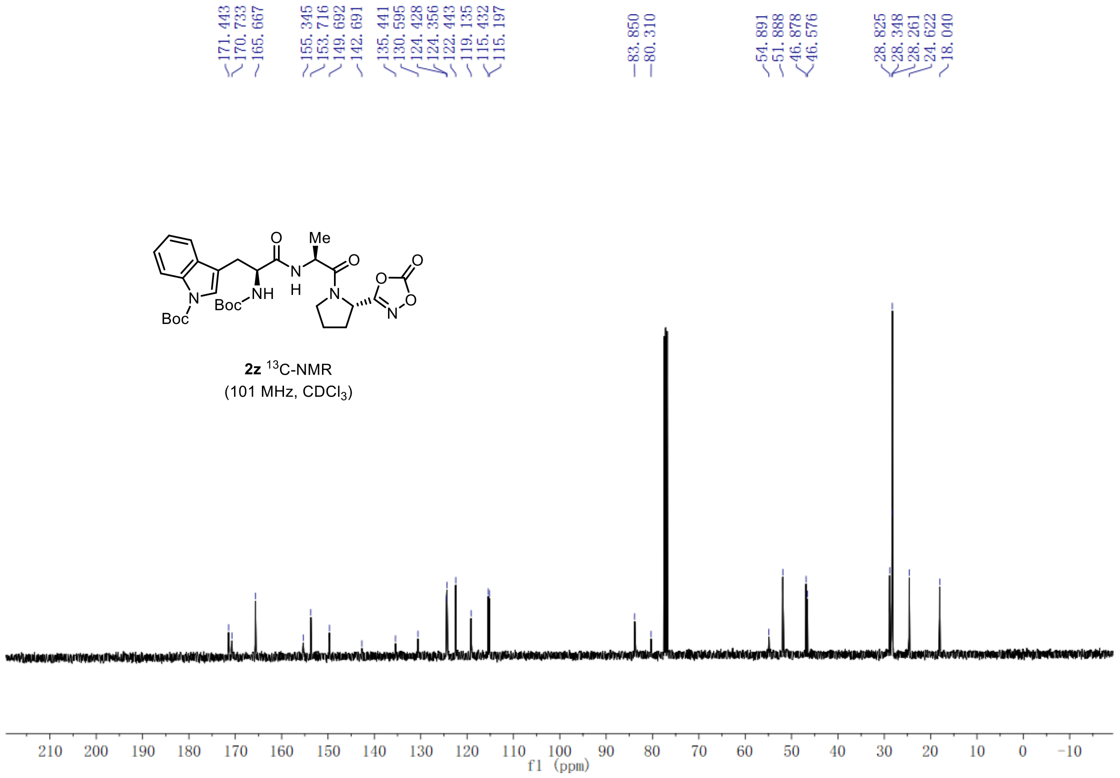

2z ^{13}C-NMR
(101 MHz, $CDCl_3$)

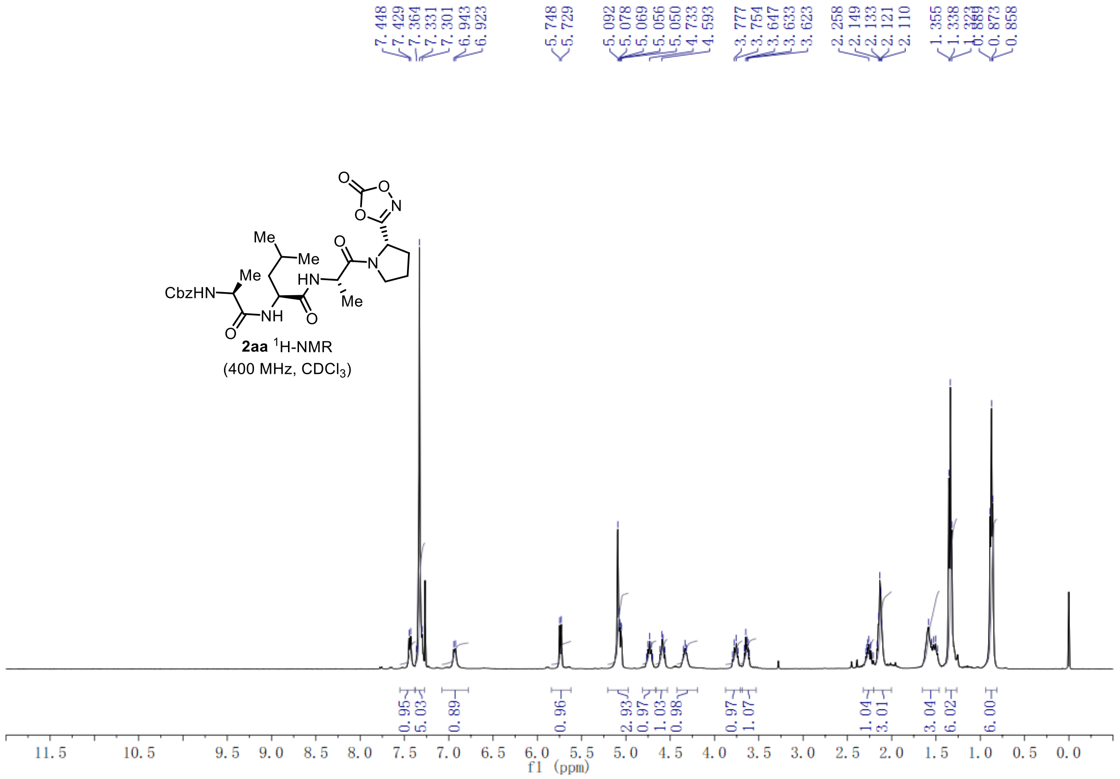
2aa ¹H-NMR
(400 MHz, CDCl3)
f1 (ppm)

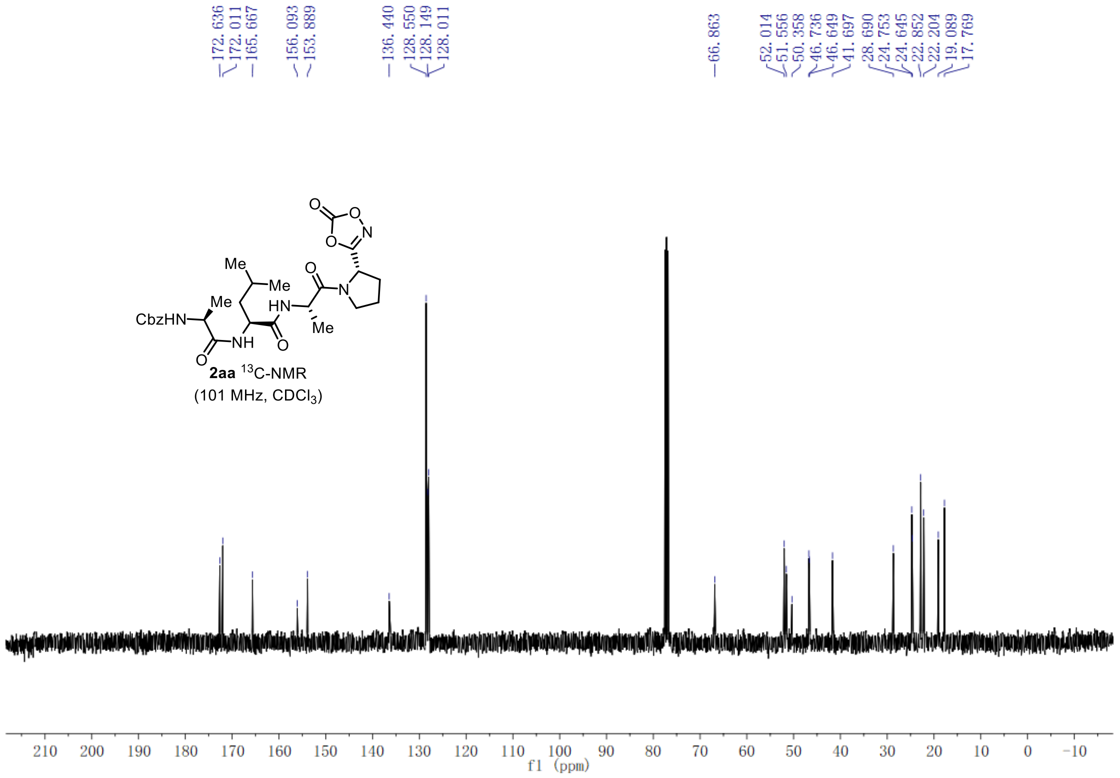
2aa ¹³C-NMR
(101 MHz, CDCl3)
f1 (ppm)

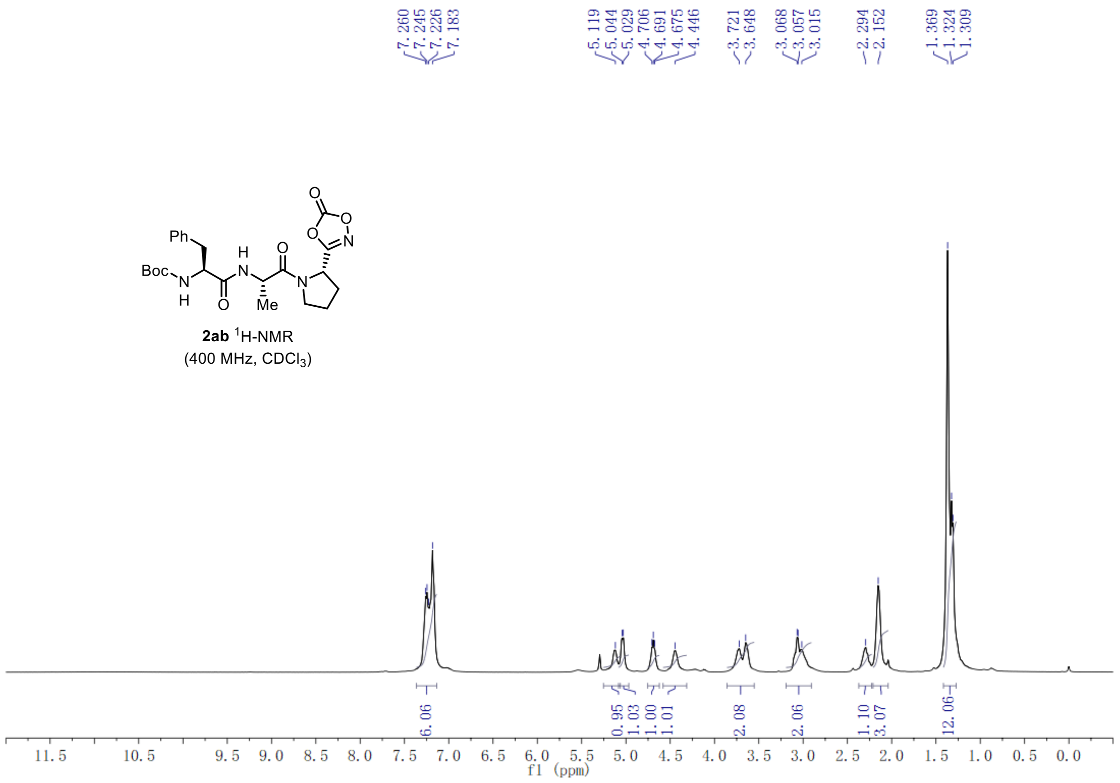
7.260
7.245
7.226
7.183
5.119
5.044
5.029
4.706
4.691
4.675
4.446
3.721
3.648
3.068
3.057
3.015
2.294
2.152
1.369
1.324
1.309
Ph
H
O
Boc
N
H
O
N
H
O
Me
N
O
O
N
2ab ¹H-NMR
(400 MHz, CDCl3)
6.06
0.95
1.03
1.00
1.01
2.08
2.06
1.10
3.07
12.06
f1 (ppm)

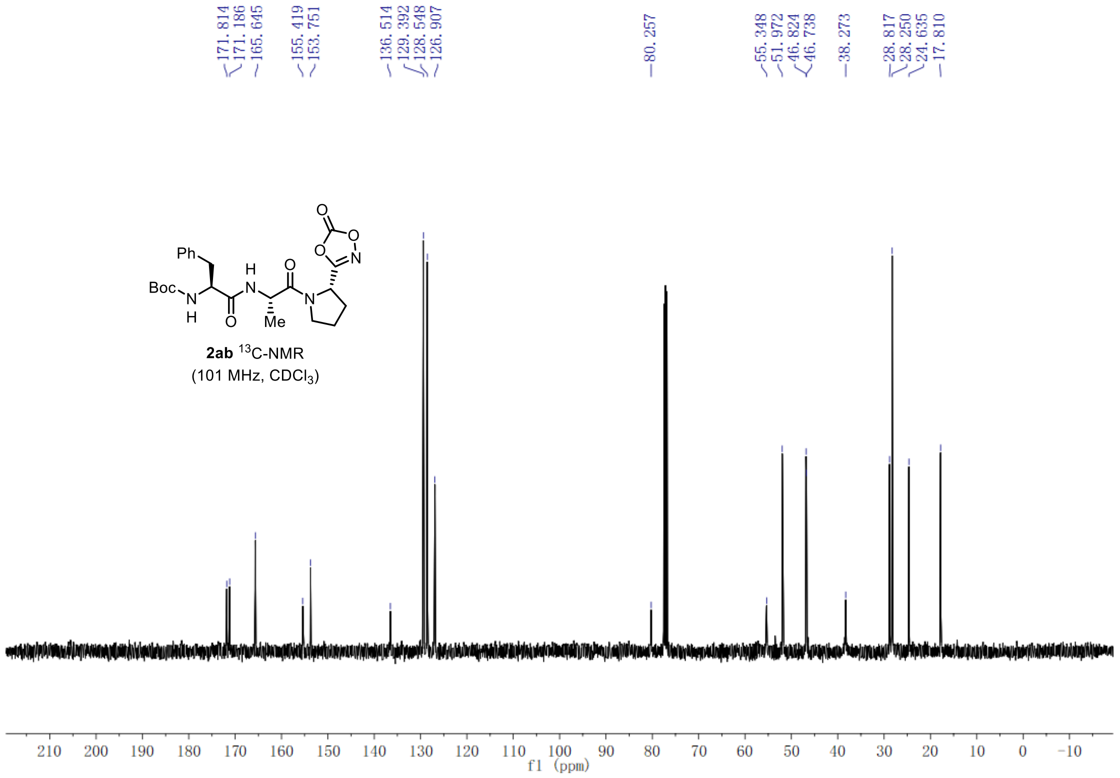
171.814
171.186
165.645
155.419
153.751
136.514
129.392
128.548
126.907
80.257
55.348
51.972
46.824
46.738
38.273
28.817
28.250
24.635
17.810
Ph
Boc
N
H
O
N
H
O
Me
N
2ab ¹³C-NMR
(101 MHz, CDCl3)
f1 (ppm)

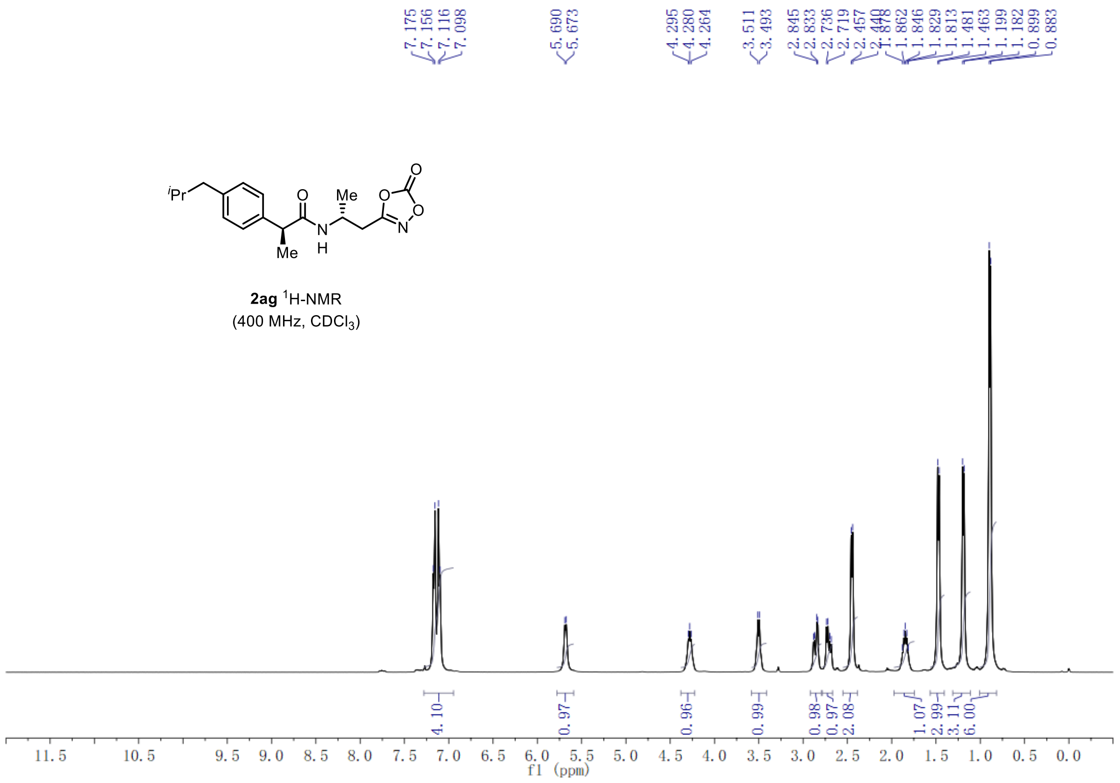
7.175
7.156
7.116
7.098
5.690
5.673
4.295
4.280
4.264
3.511
3.493
2.845
2.833
2.736
2.719
2.457
2.440
1.878
1.862
1.846
1.829
1.813
1.481
1.463
1.199
1.182
0.899
0.883
iPr
O
Me
O
O
O
N
N
H
Me
2ag 1H-NMR
(400 MHz, CDCl3)
4.10
0.97
0.96
0.99
0.98
0.97
2.08
1.07
2.99
3.11
6.00
f1 (ppm)

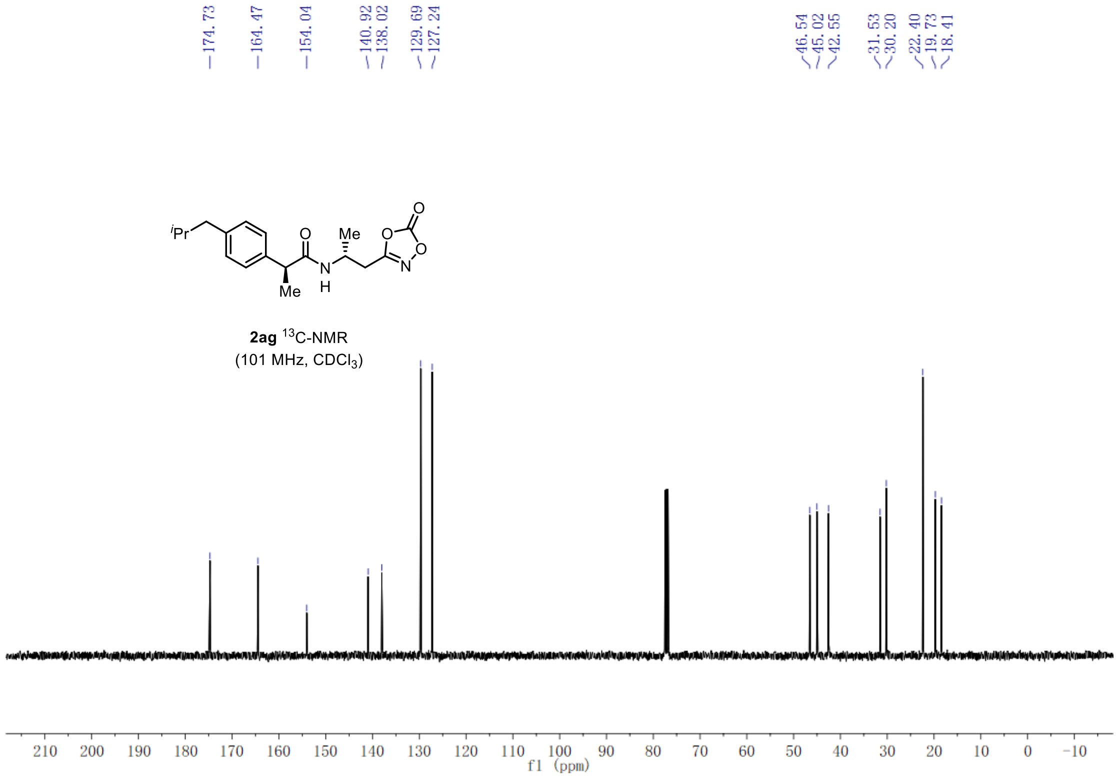
174.73
164.47
154.04
140.92
138.02
129.69
127.24
46.54
45.02
42.55
31.53
30.20
22.40
19.73
18.41
iPr
O
Me
O
O
O
N
N
H
Me
2ag 13C-NMR
(101 MHz, CDCl3)
f1 (ppm)

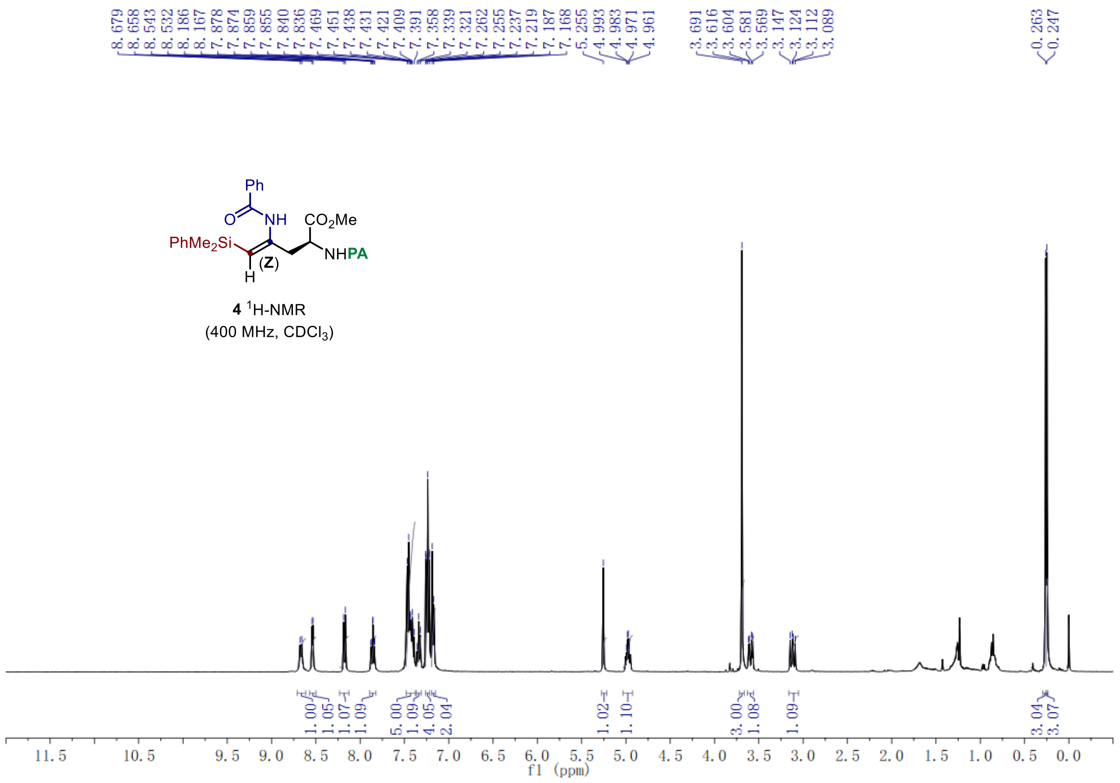
4 1H-NMR
(400 MHz, CDCl3)
PhMe2Si
(Z)
H
NH
O
Ph
CO2Me
NHPA
f1 (ppm)

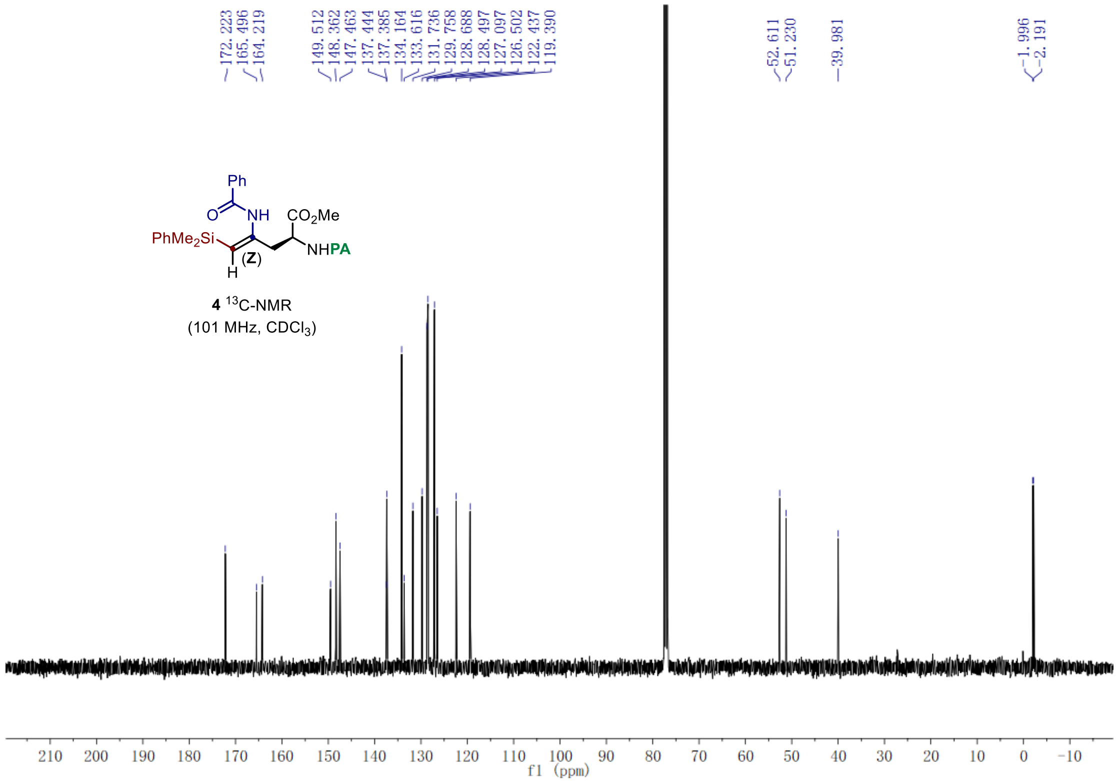
4 13C-NMR
(101 MHz, CDCl3)
PhMe2Si
(Z)
H
NH
O
Ph
CO2Me
NHPA
f1 (ppm)

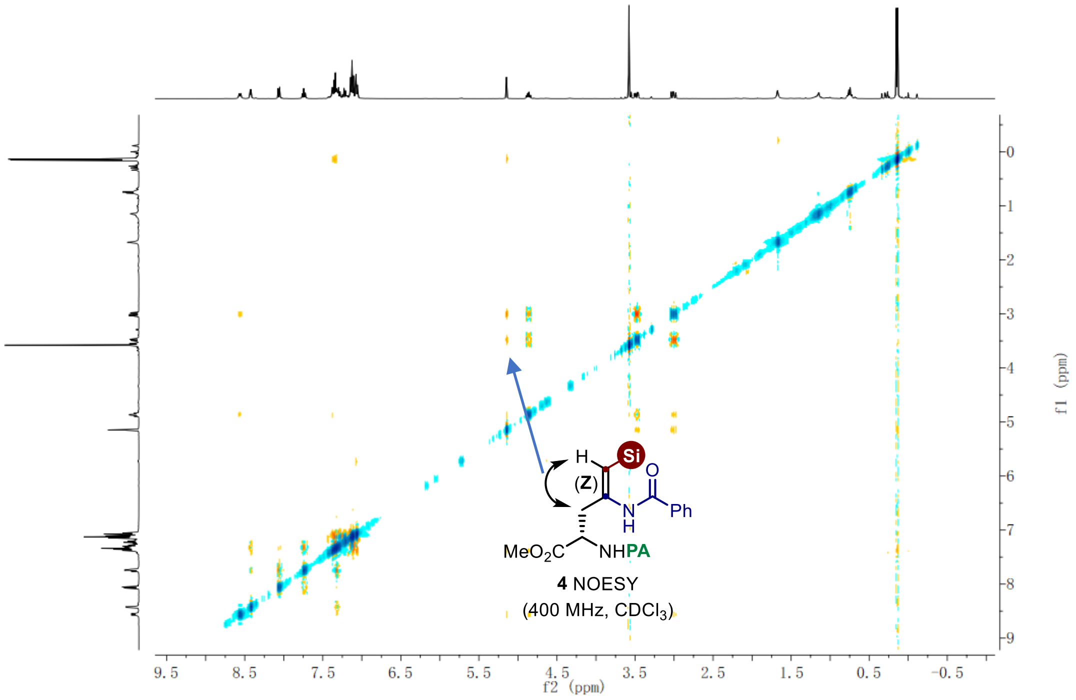
H
Si
(Z)
O
N
H
Ph
MeO2C
NHPA
4 NOESY
(400 MHz, CDCl3)
f2 (ppm)
f1 (ppm)
9.5
8.5
7.5
6.5
5.5
4.5
3.5
2.5
1.5
0.5
-0.5
0
1
2
3
4
5
6
7
8
9

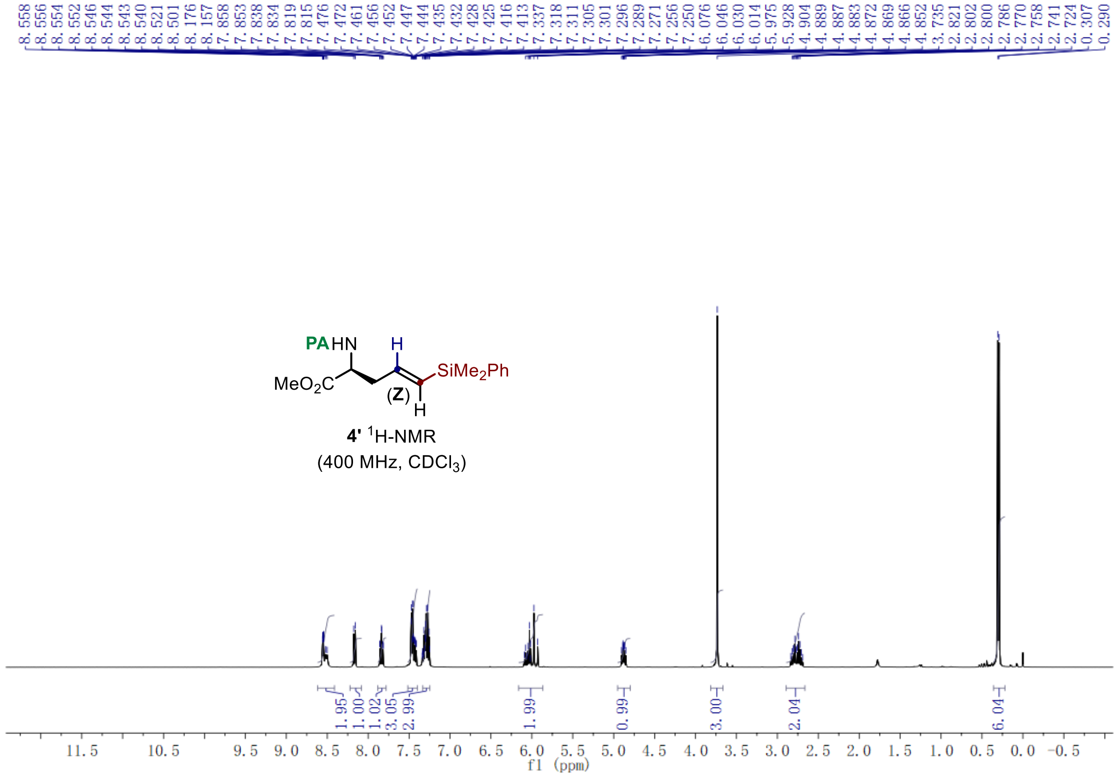

PA HN
MeO2C
(Z)
SiMe2Ph
4' 1H-NMR
(400 MHz, CDCl3)
1.95
1.00
1.02
3.05
2.99
1.99
0.99
3.00
2.04
6.04
f1 (ppm)

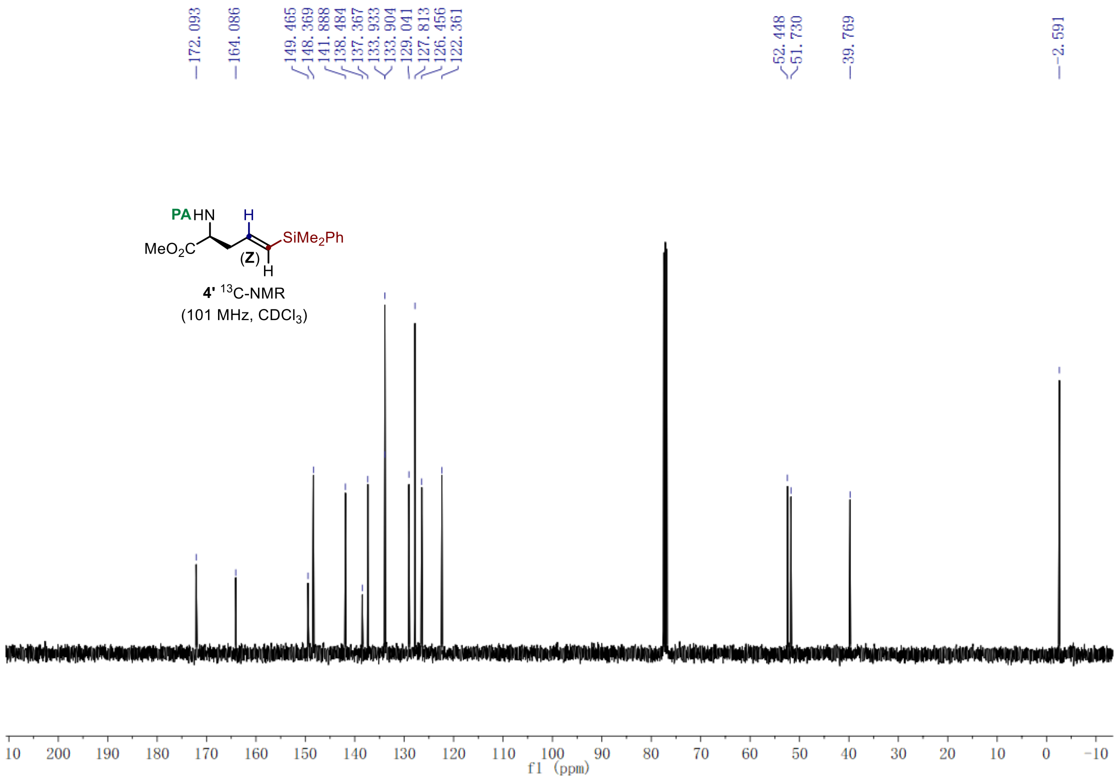

172.093
164.086
149.465
148.369
141.888
138.484
137.367
133.933
133.904
129.041
127.813
126.456
122.361
52.448
51.730
39.769
-2.591
PA HN
MeO2C
(Z)
SiMe2Ph
4' 13C-NMR
(101 MHz, CDCl3)
f1 (ppm)

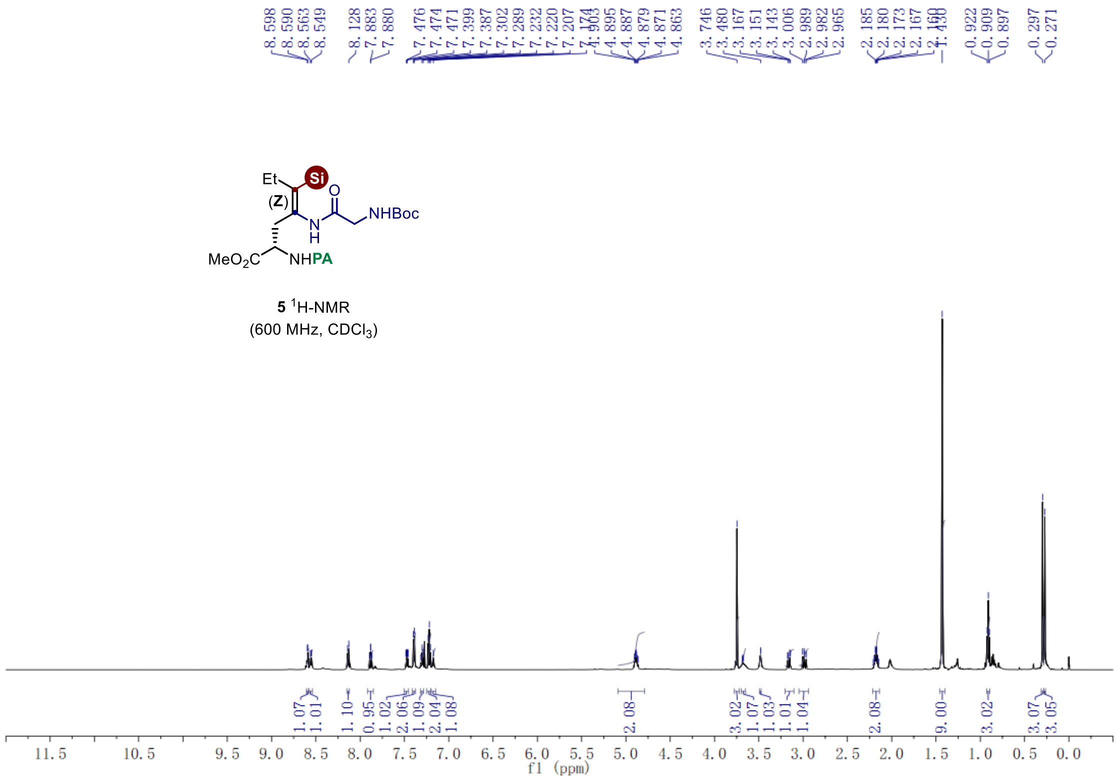

8.598
8.590
8.563
8.549
8.128
7.883
7.880
7.476
7.474
7.471
7.399
7.387
7.302
7.289
7.232
7.220
7.207
4.895
4.887
4.879
4.871
4.863
3.746
3.480
3.167
3.151
3.143
3.006
2.989
2.982
2.965
2.185
2.180
2.173
2.167
1.430
0.922
0.909
0.897
0.297
0.271
Et
Si
(Z)
O
N
H
NHBoc
MeO2C
NHPA
5 1H-NMR
(600 MHz, CDCl3)
1.07
1.01
1.10
0.95
1.02
2.06
1.09
2.04
1.08
2.08
3.02
1.07
1.03
1.01
1.04
2.08
9.00
3.02
3.07
3.05
f1 (ppm)

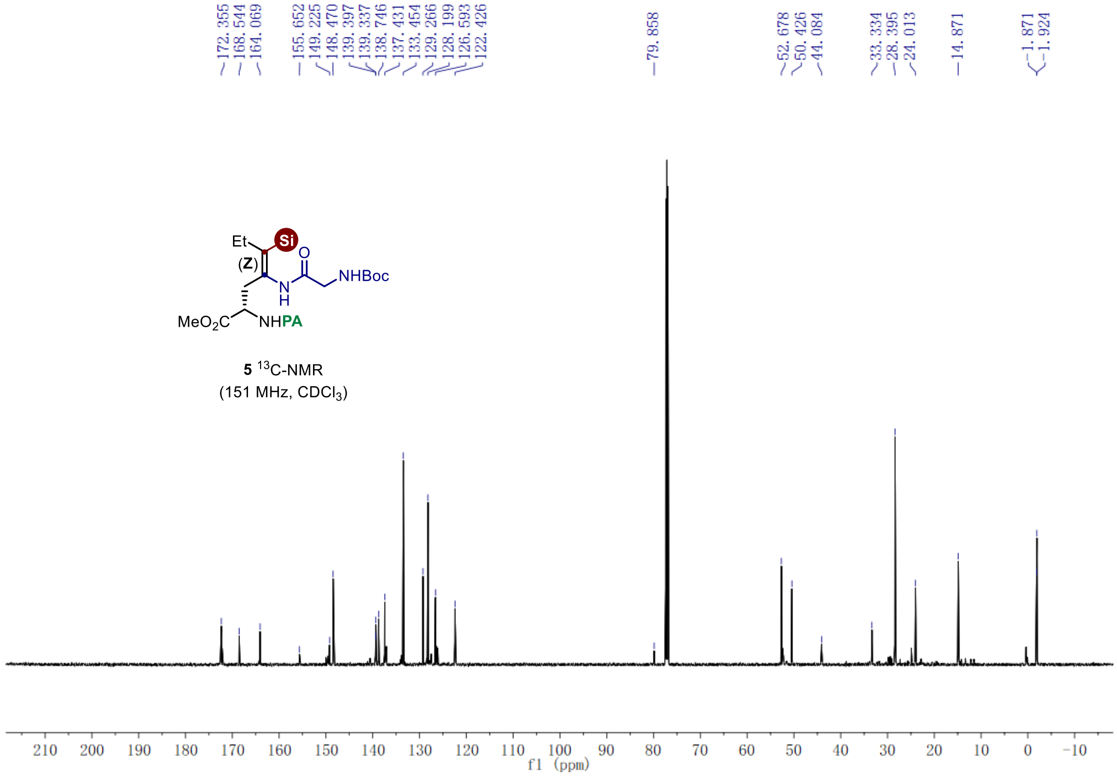

172.355
168.544
164.069
155.652
149.225
148.470
139.397
139.337
138.746
137.431
133.454
129.266
128.199
126.593
122.426
79.858
52.678
50.426
44.084
33.334
28.395
24.013
14.871
-1.871
-1.924
Et
Si
(Z)
O
N
H
NHBoc
MeO2C
NHPA
5 13C-NMR
(151 MHz, CDCl3)
f1 (ppm)

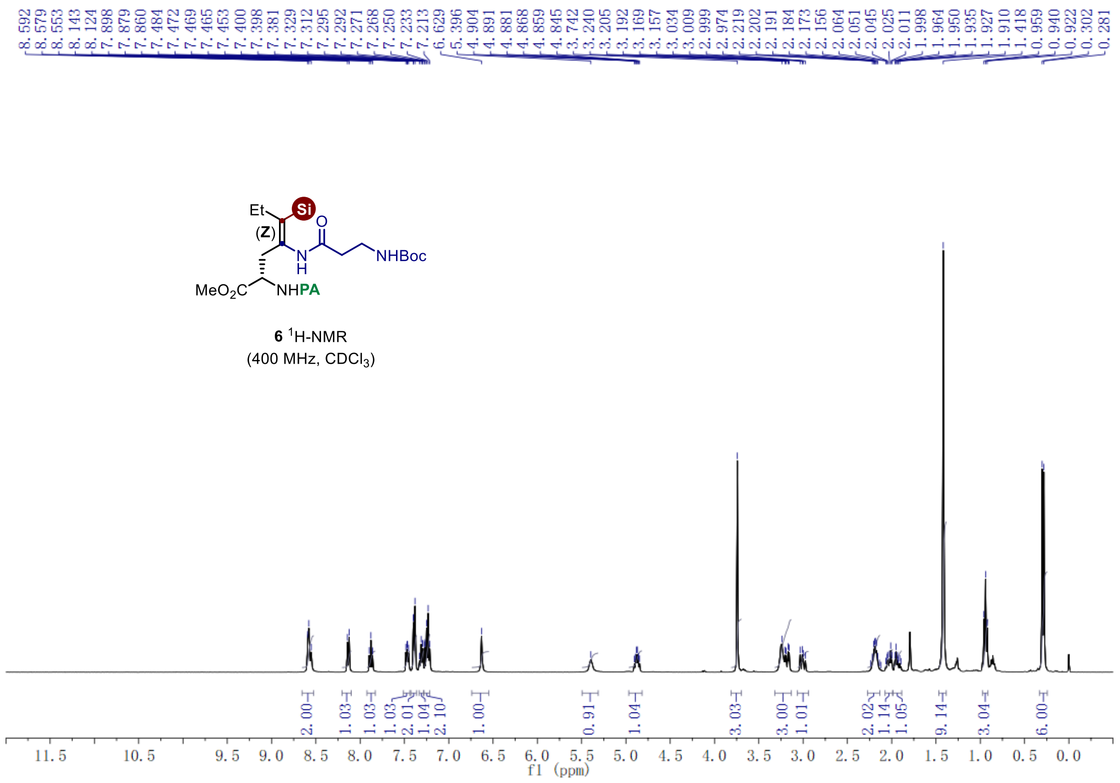
Et
Si
(Z)
O
N
H
NHBoc
MeO2C
NHPA
6 1H-NMR
(400 MHz, CDCl3)
f1 (ppm)

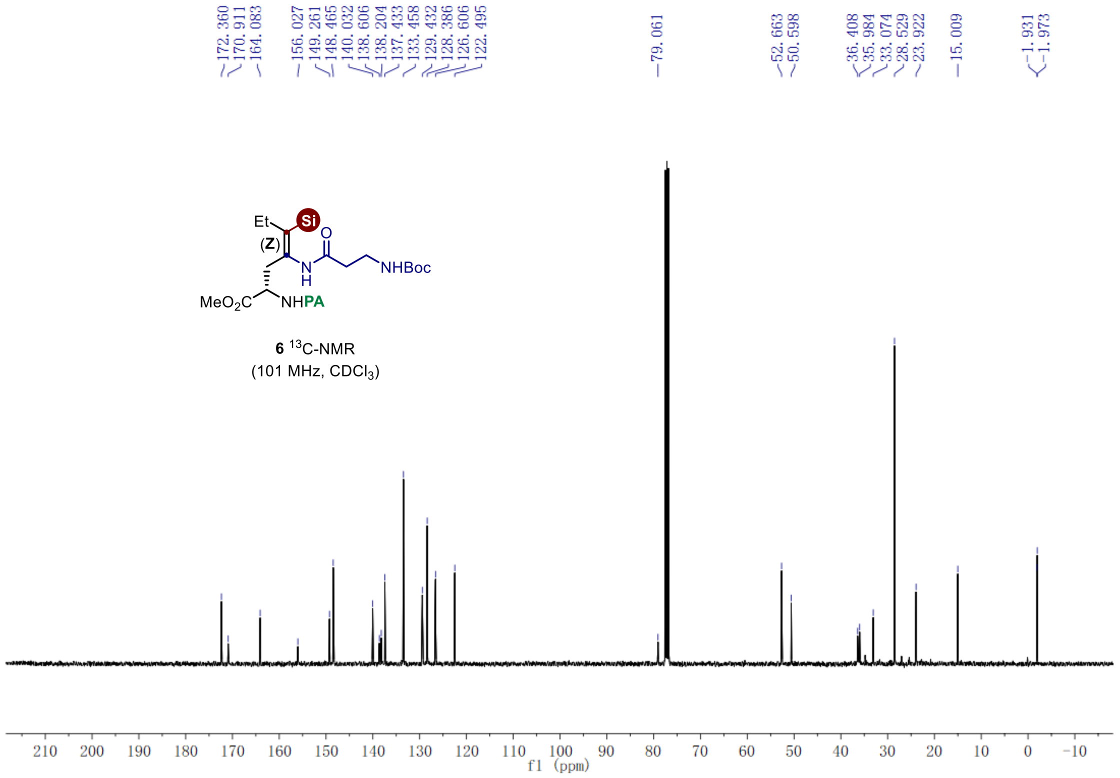
Et
Si
(Z)
O
N
H
NHBoc
MeO2C
NHPA
6 13C-NMR
(101 MHz, CDCl3)
f1 (ppm)

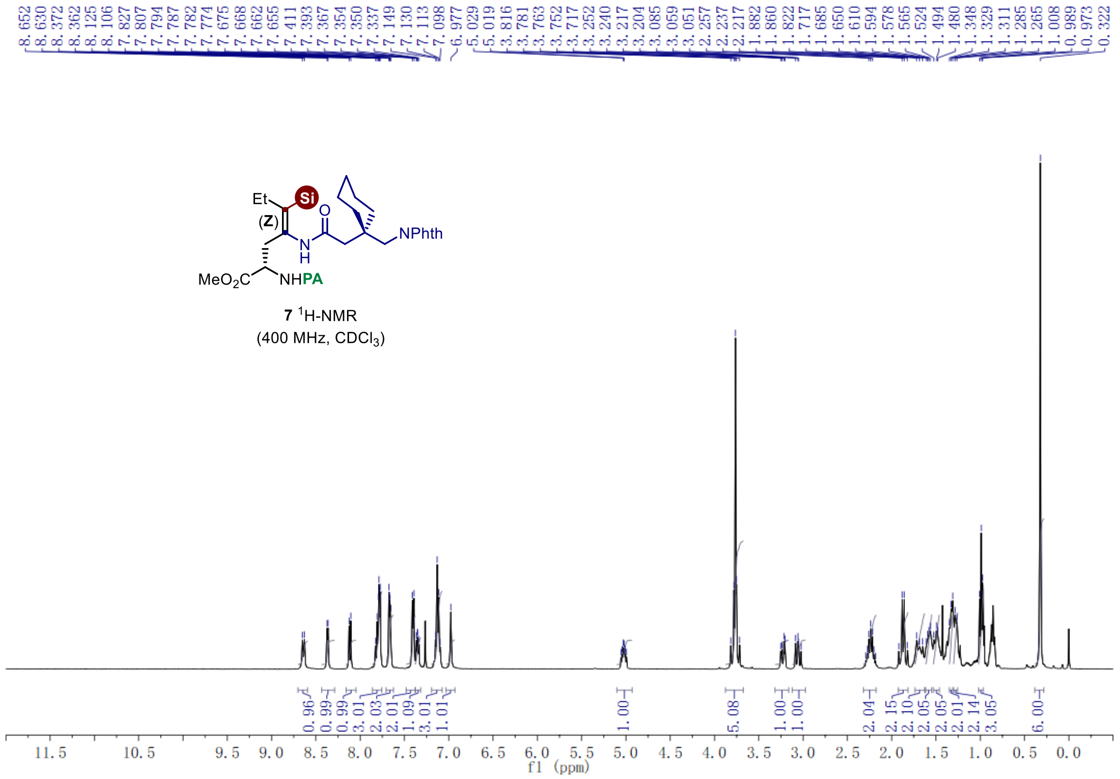
Et
Si
(Z)
O
N
H
NPhth
MeO2C
NHPA
7 1H-NMR
(400 MHz, CDCl3)
f1 (ppm)

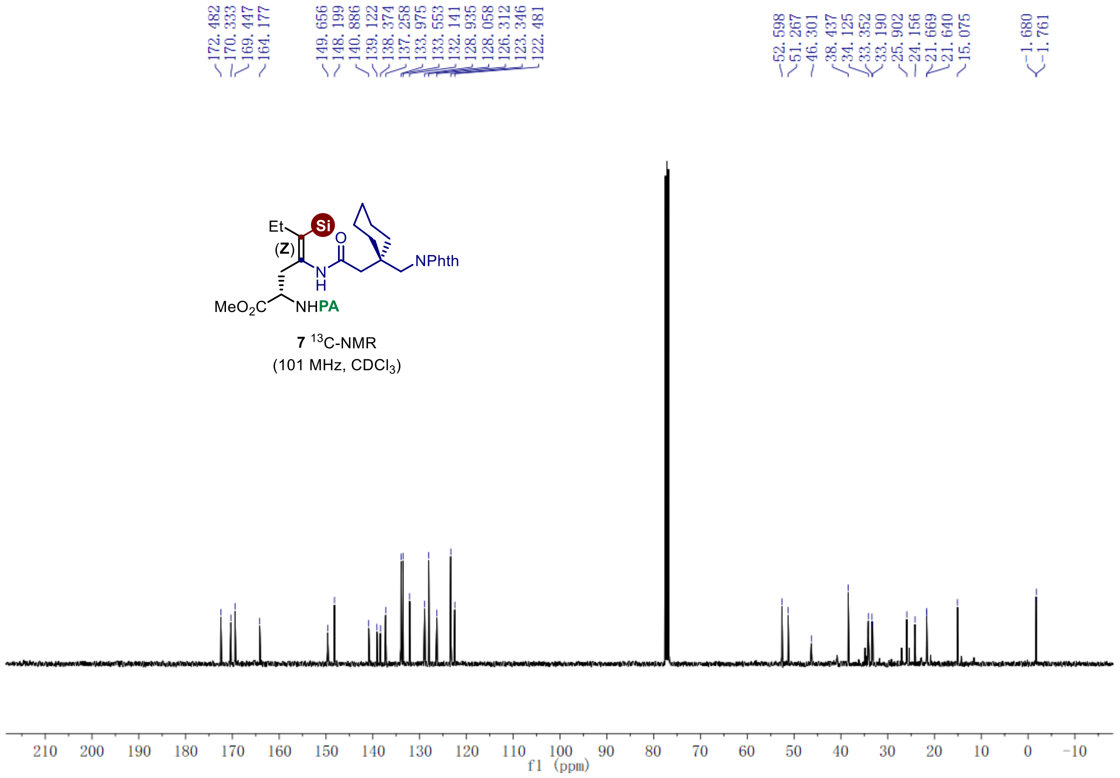
Et
Si
(Z)
O
N
H
NPhth
MeO2C
NHPA
7 13C-NMR
(101 MHz, CDCl3)
f1 (ppm)

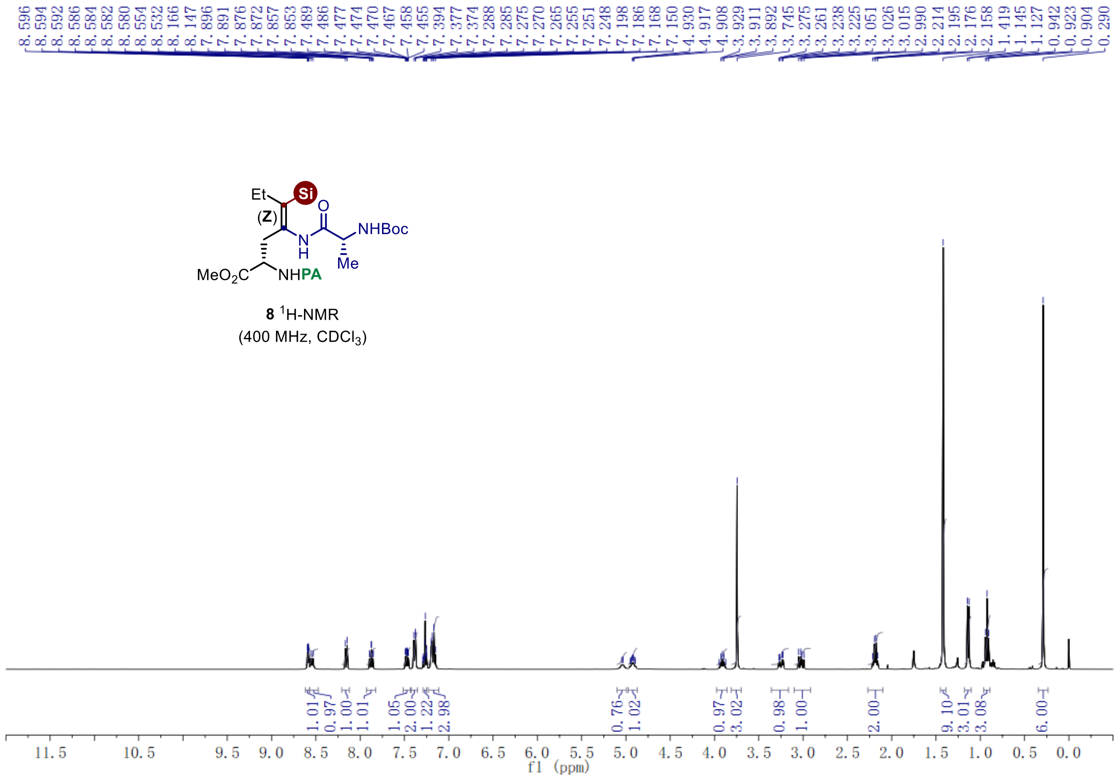
8 1H-NMR
(400 MHz, CDCl3)
f1 (ppm)

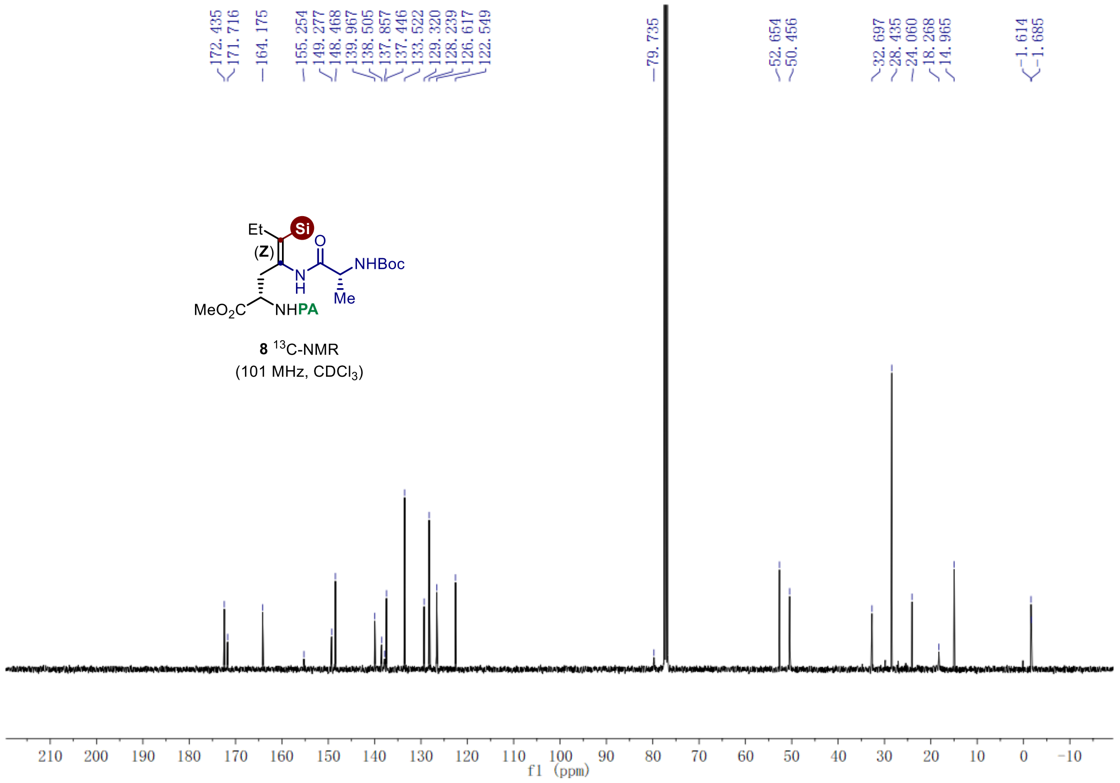
8 13C-NMR
(101 MHz, CDCl3)
f1 (ppm)

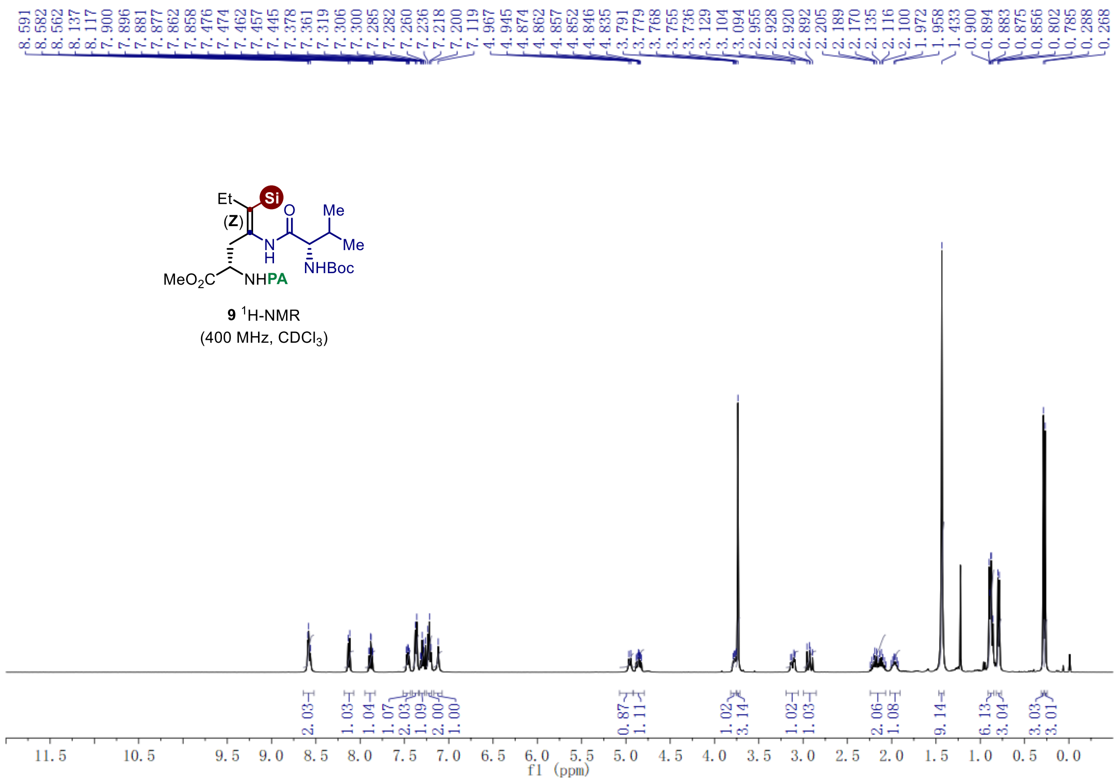
9 1H-NMR
(400 MHz, CDCl3)
f1 (ppm)

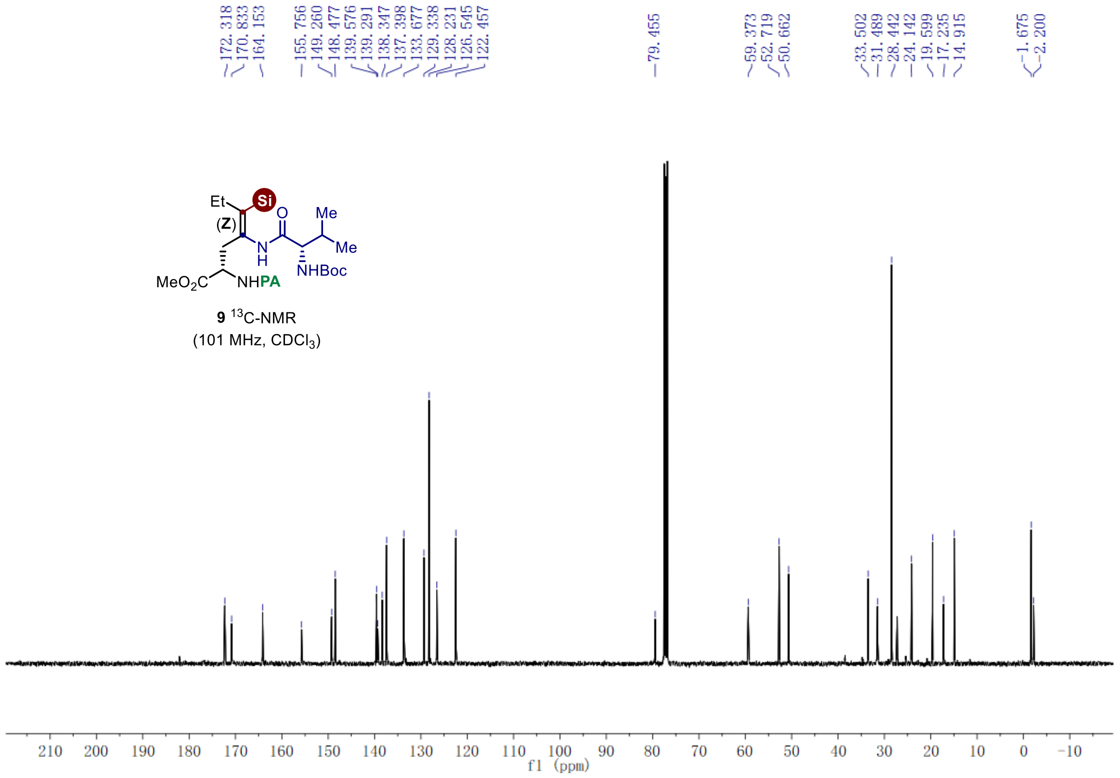
9 13C-NMR
(101 MHz, CDCl3)
f1 (ppm)

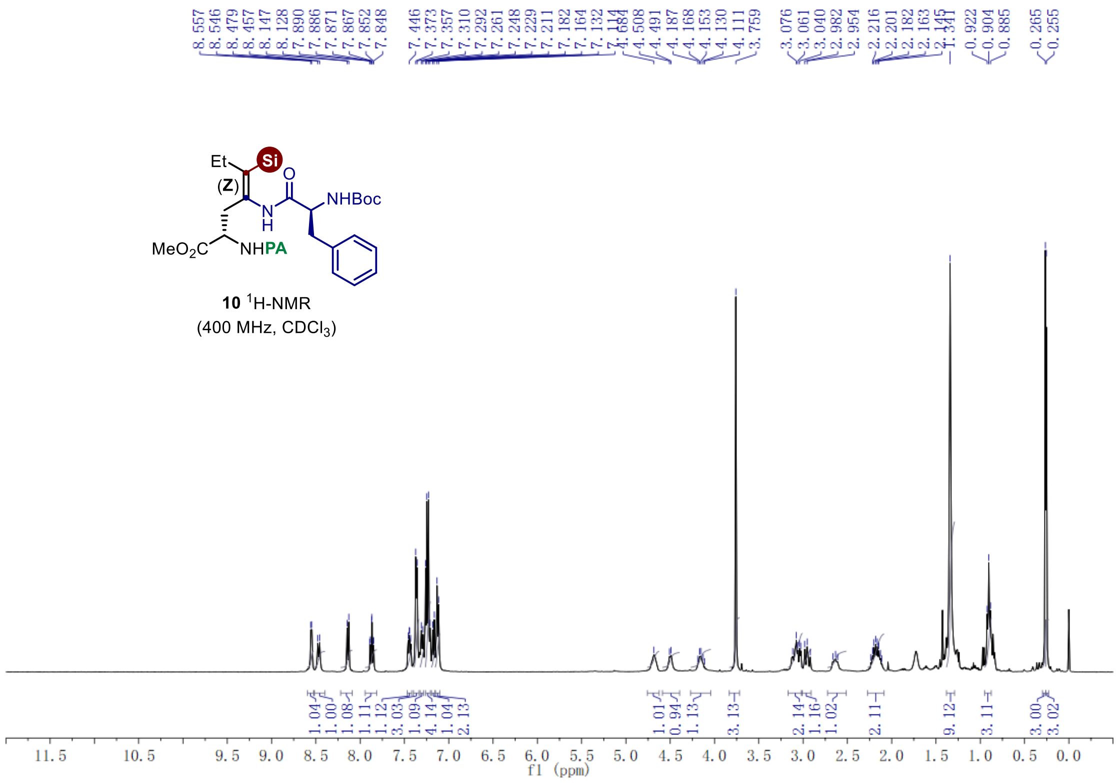
Et
Si
(Z)
O
N
H
NHBoc
MeO2C
NHPA
10 1H-NMR
(400 MHz, CDCl3)
f1 (ppm)

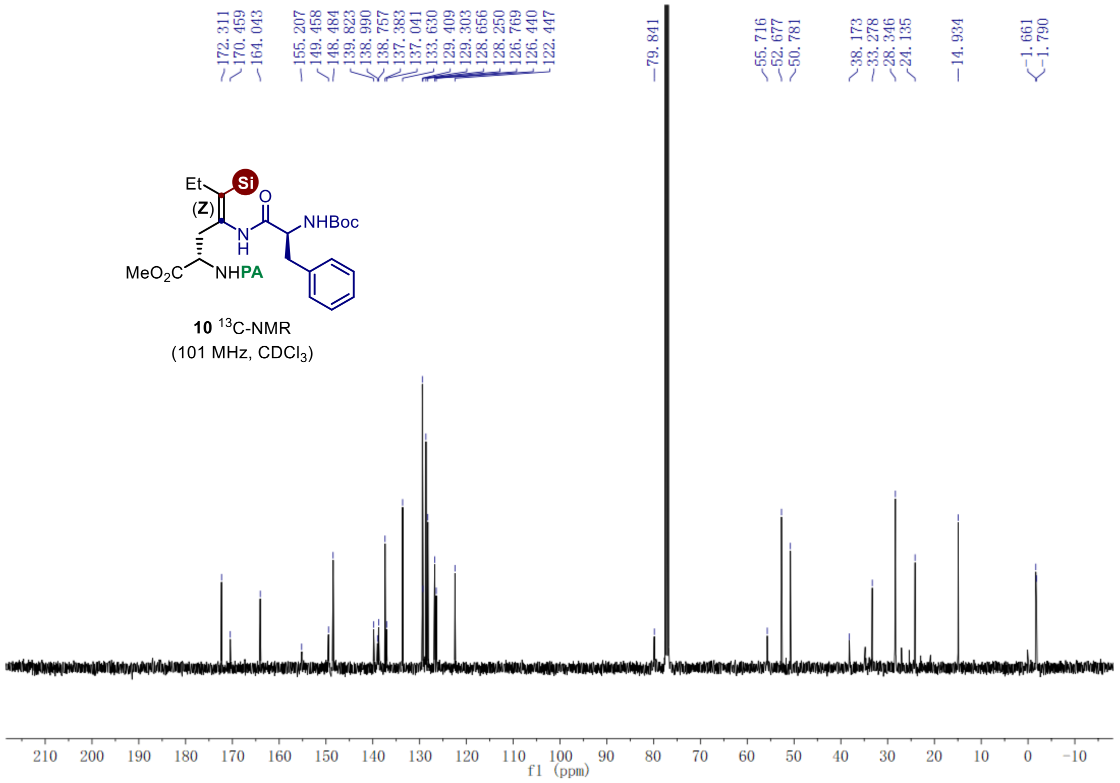
Et
Si
(Z)
O
N
H
NHBoc
MeO2C
NHPA
10 13C-NMR
(101 MHz, CDCl3)
f1 (ppm)

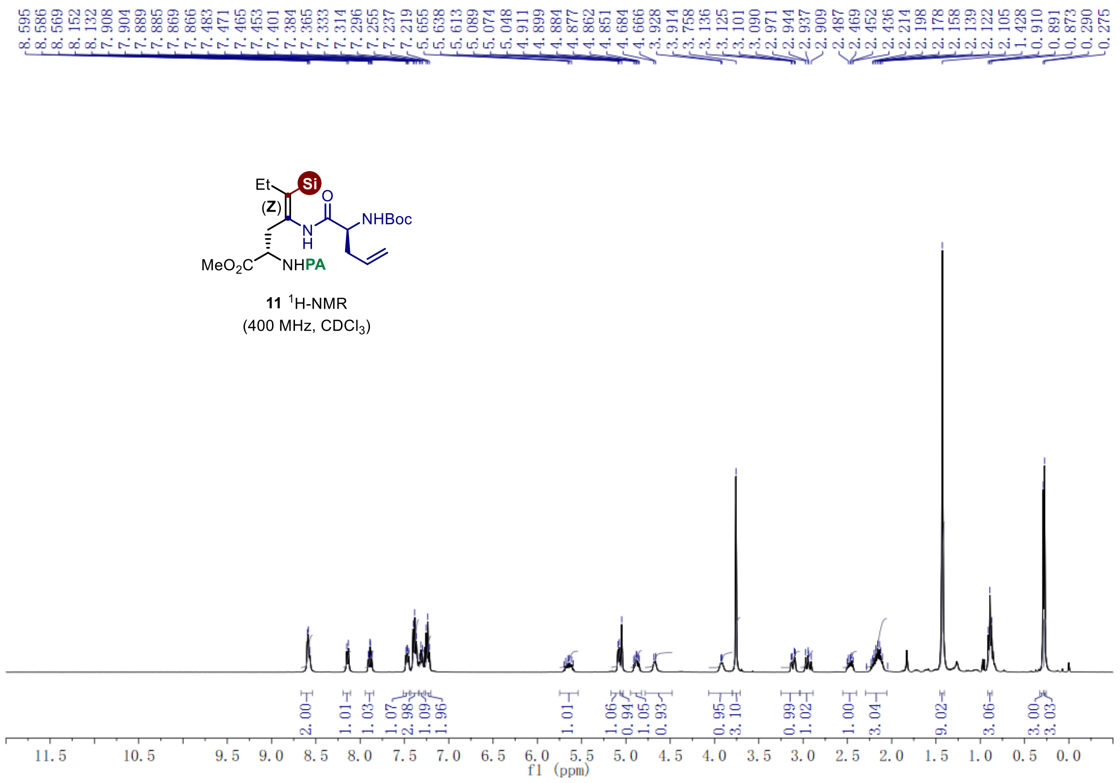

11 ^{1}H-NMR
(400 MHz, $CDCl_3$)

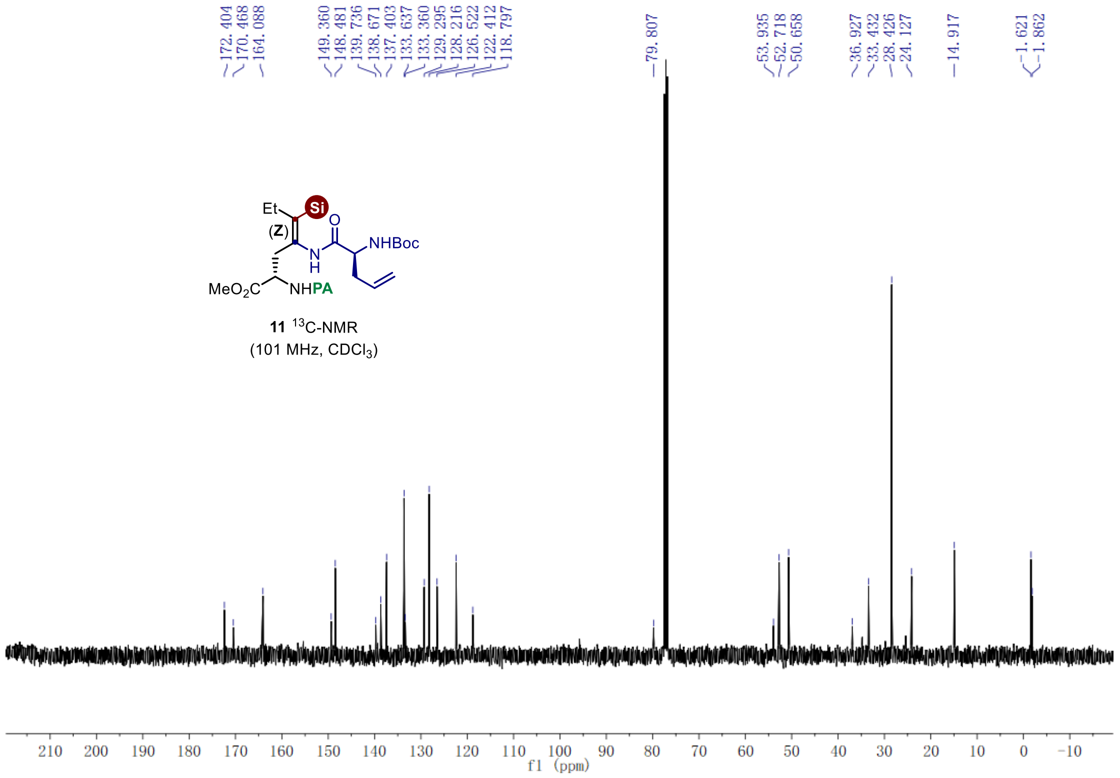

11 ^{13}C-NMR
(101 MHz, $CDCl_3$)

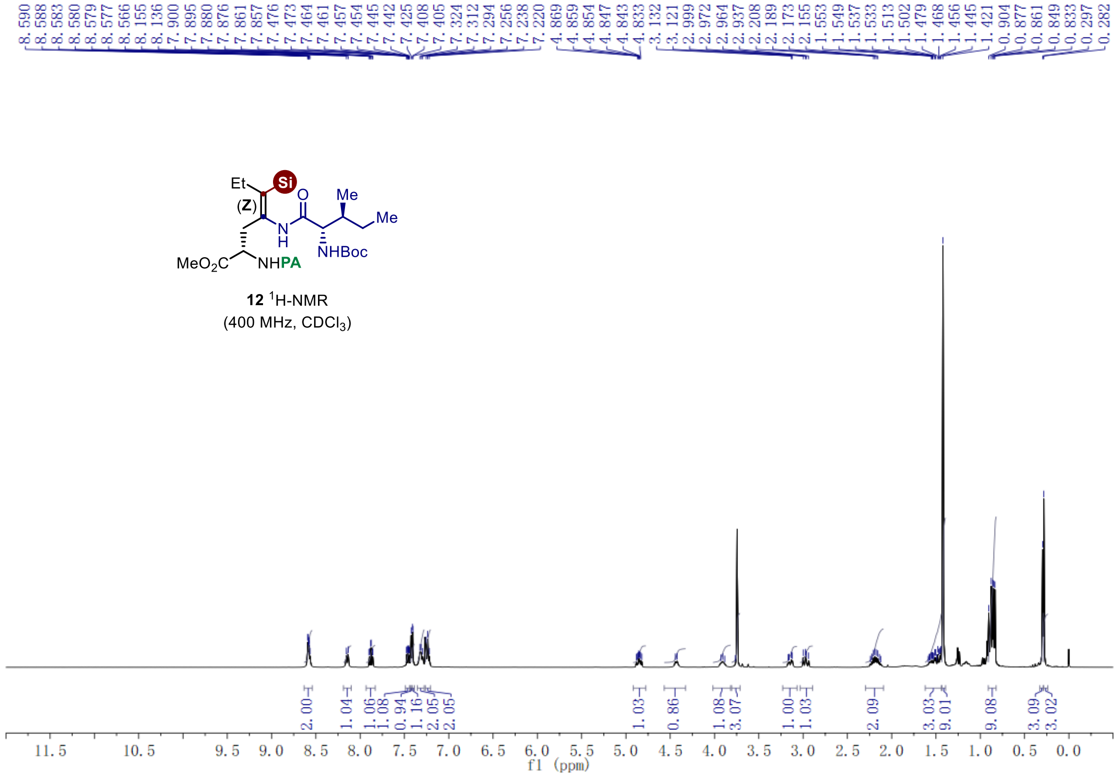

12 ¹H-NMR
(400 MHz, $CDCl_3$)

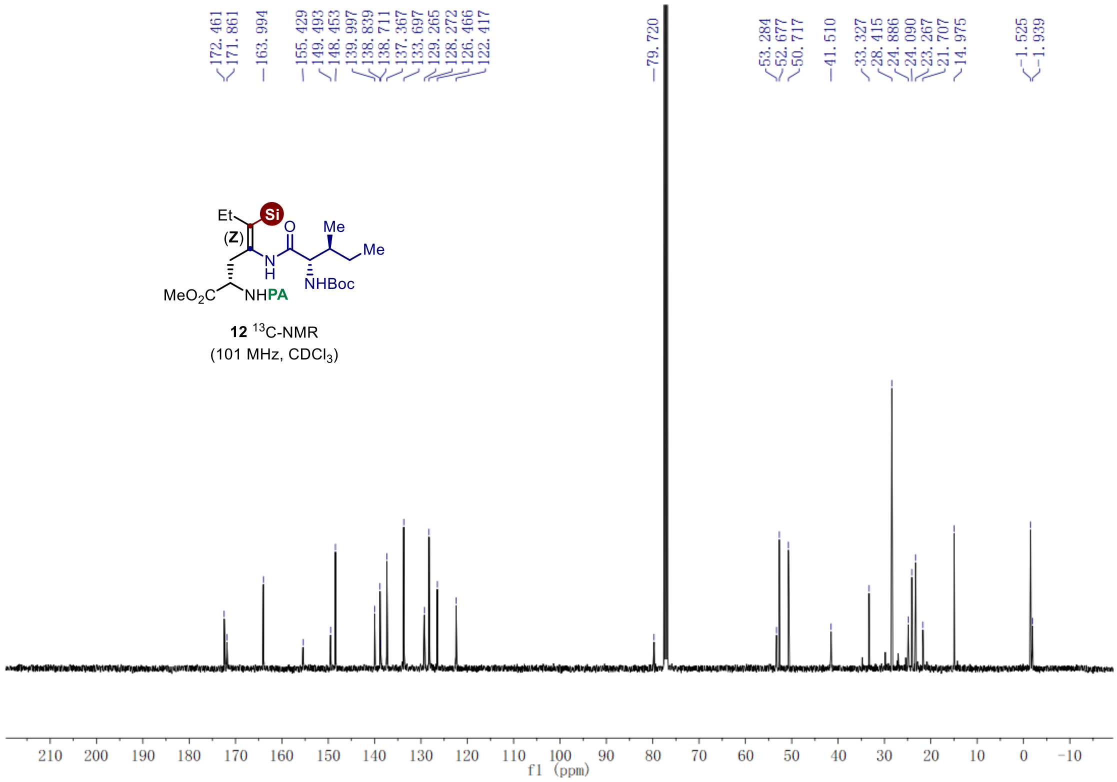

12 ¹³C-NMR
(101 MHz, $CDCl_3$)

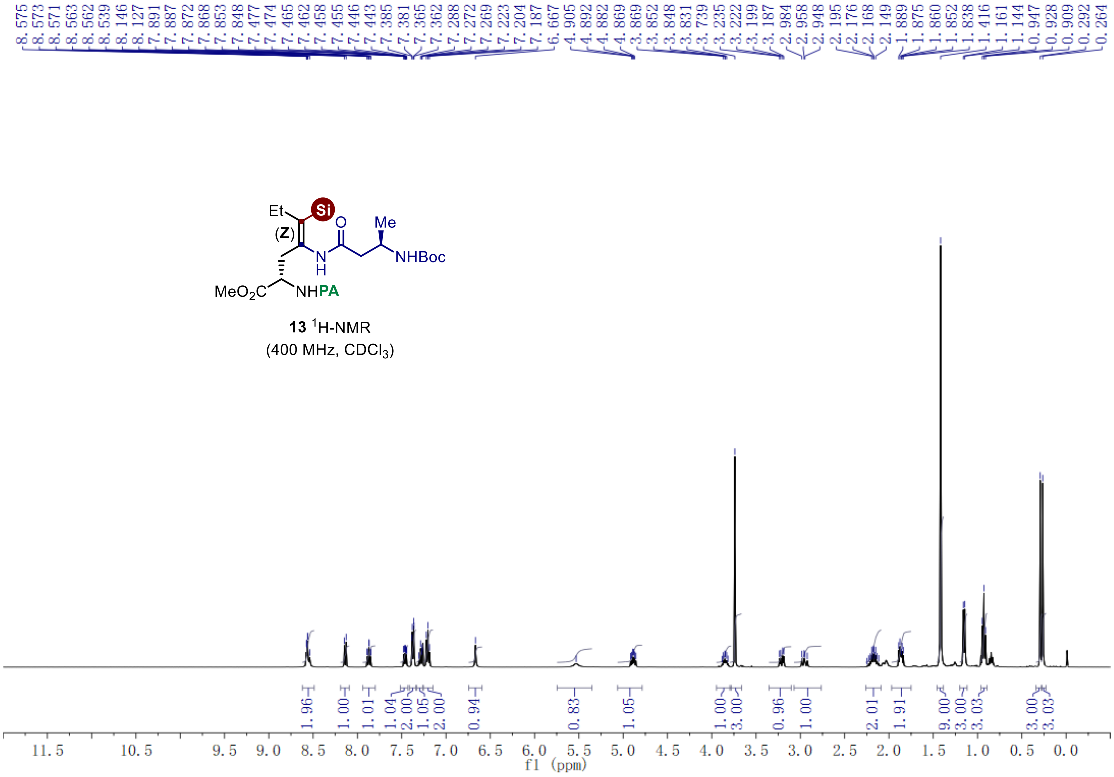
8.575
8.573
8.571
8.563
8.562
8.539
8.146
8.127
7.891
7.887
7.872
7.868
7.853
7.848
7.477
7.474
7.465
7.462
7.458
7.455
7.446
7.443
7.385
7.381
7.365
7.362
7.288
7.272
7.269
7.223
7.204
7.187
6.667
4.905
4.892
4.882
4.869
3.869
3.852
3.848
3.831
3.739
3.235
3.222
3.199
3.187
2.984
2.958
2.948
2.195
2.176
2.168
2.149
1.889
1.875
1.860
1.852
1.838
1.416
1.161
1.144
0.947
0.928
0.909
0.292
0.264
Et
Si
(Z)
O
Me
N
H
NHBoc
MeO2C
NHPA
13 1H-NMR
(400 MHz, CDCl3)
1.96
1.00
1.01
1.04
2.00
1.05
2.00
0.94
0.83
1.05
1.00
3.00
0.96
1.00
2.01
1.91
9.00
3.00
3.03
3.00
3.03
11.5
10.5
9.5
9.0
8.5
8.0
7.5
7.0
6.5
6.0
5.5
5.0
4.5
4.0
3.5
3.0
2.5
2.0
1.5
1.0
0.5
0.0
f1 (ppm)

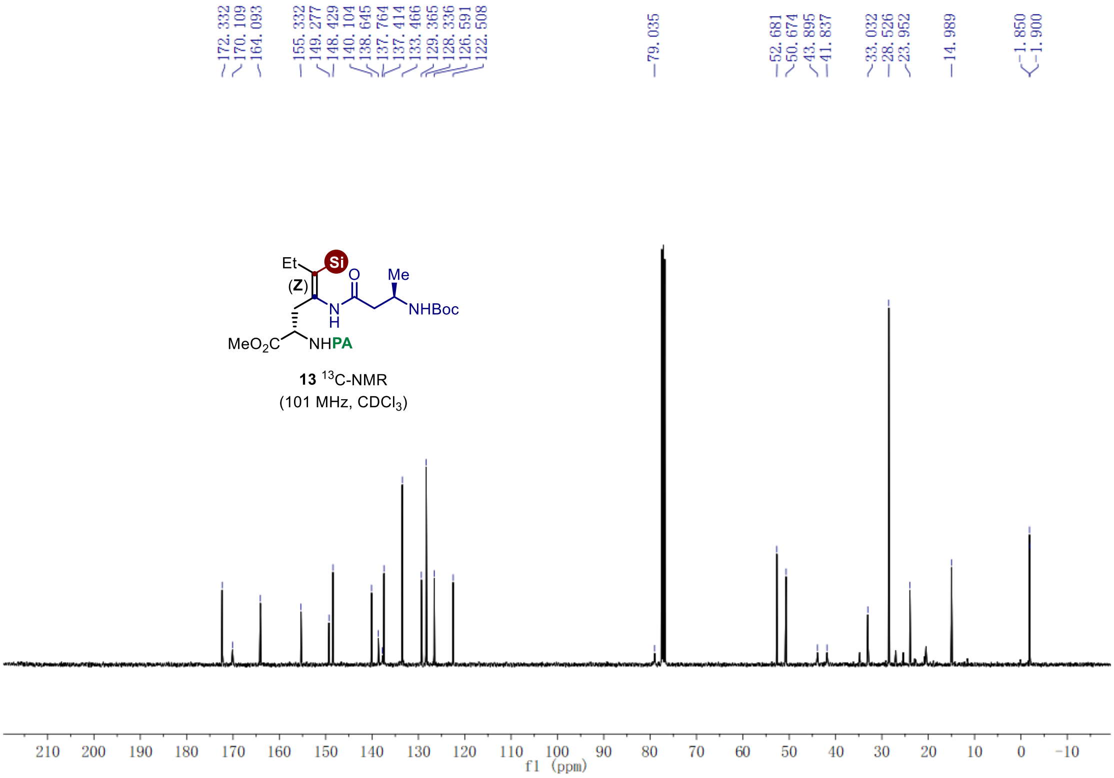
172.332
170.109
164.093
155.332
149.277
148.429
140.104
138.645
137.764
137.414
133.466
129.365
128.336
126.591
122.508
79.035
52.681
50.674
43.895
41.837
33.032
28.526
23.952
14.989
-1.850
-1.900
Et
Si
(Z)
O
Me
N
H
NHBoc
MeO2C
NHPA
13 13C-NMR
(101 MHz, CDCl3)
210
200
190
180
170
160
150
140
130
120
110
100
90
80
70
60
50
40
30
20
10
0
-10
f1 (ppm)

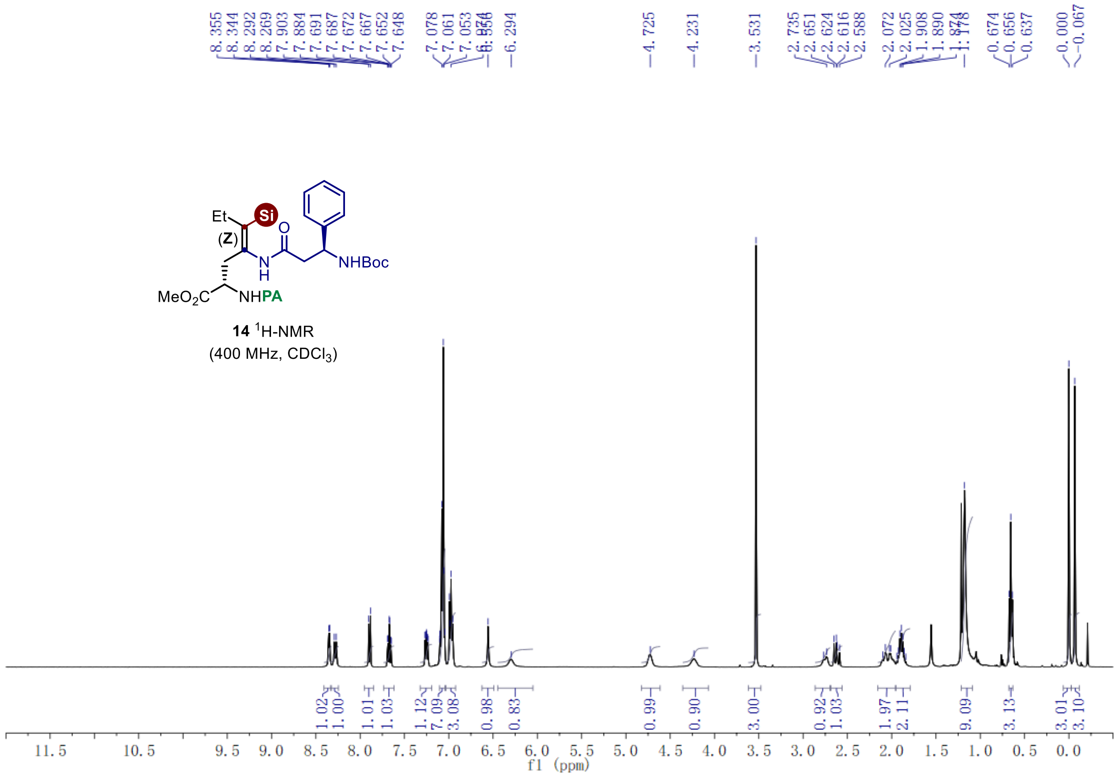

14 ^{1}H-NMR
(400 MHz, $CDCl_3$)

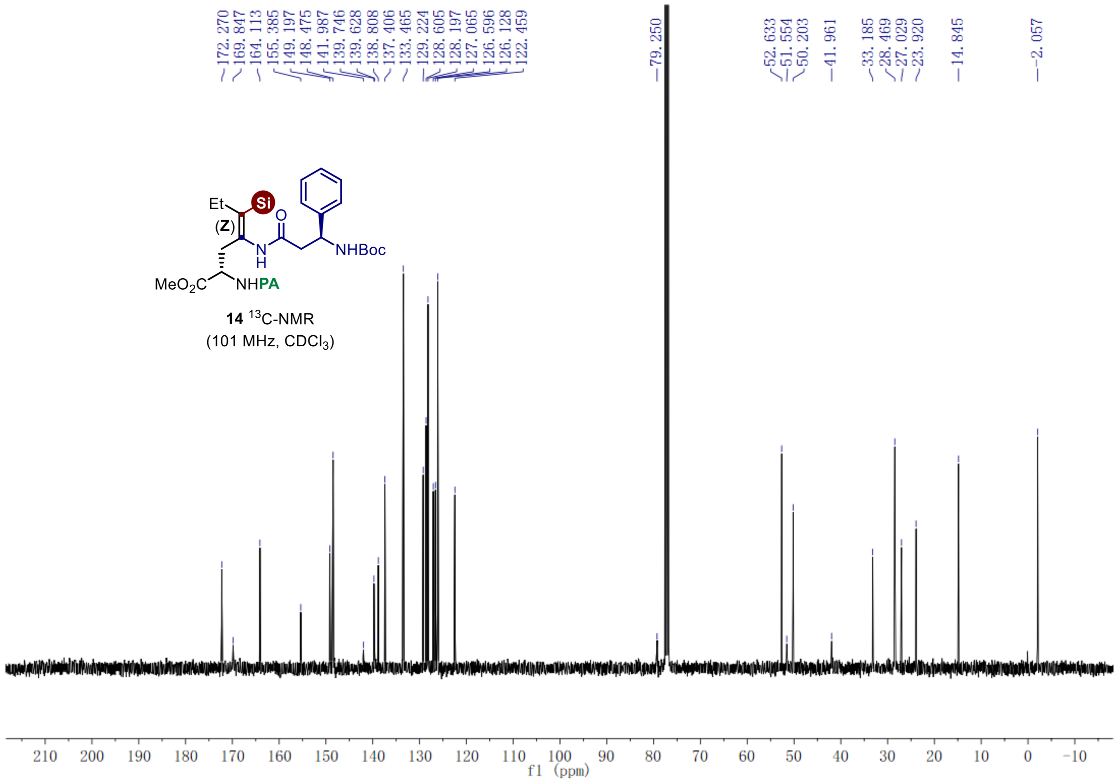

14 ^{13}C-NMR
(101 MHz, $CDCl_3$)

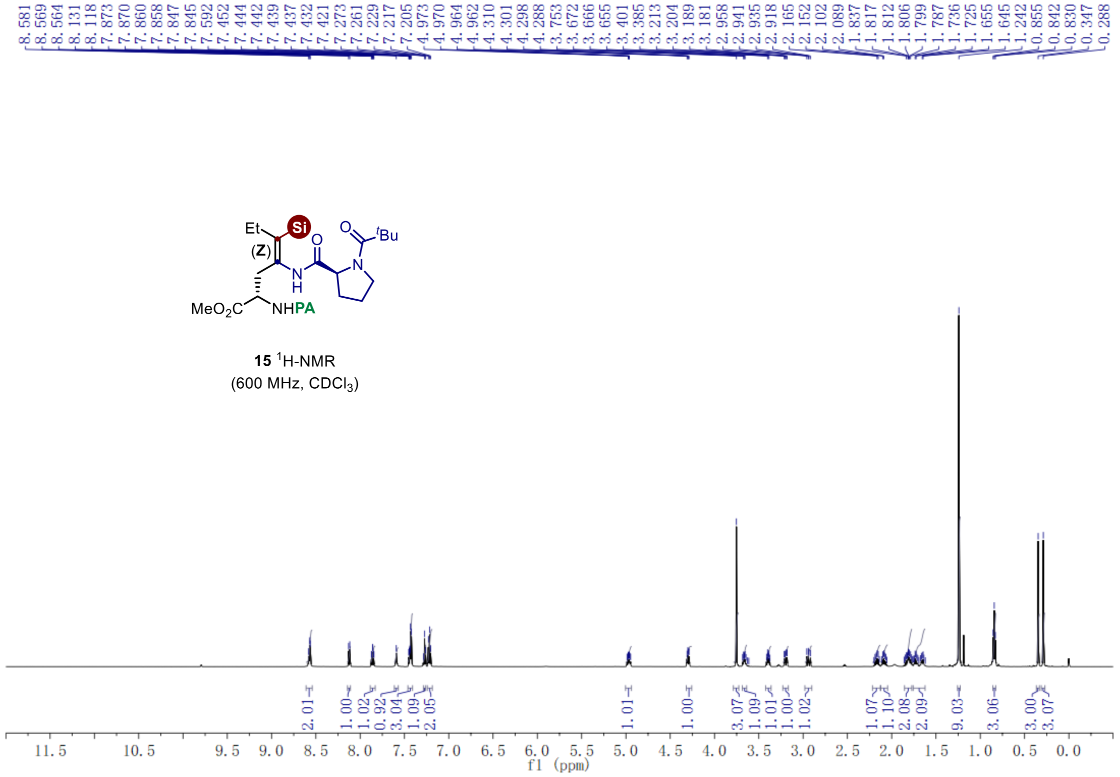
8.581 8.569 8.564 8.131 8.118 7.873 7.870 7.860 7.858 7.847 7.845 7.592 7.452 7.444 7.442 7.439 7.437 7.432 7.421 7.273 7.261 7.229 7.217 7.205 4.973 4.970 4.964 4.962 4.310 4.301 4.298 4.288 3.753 3.672 3.666 3.655 3.401 3.385 3.213 3.204 3.189 3.181 2.958 2.941 2.935 2.918 2.165 2.152 2.102 2.089 1.837 1.817 1.812 1.806 1.799 1.787 1.736 1.725 1.655 1.645 1.242 0.855 0.842 0.830 0.347 0.288
Et
Si
(Z)
O
O
tBu
N
N
H
MeO2C
NHPA
15 1H-NMR
(600 MHz, CDCl3)
2.01 1.00 1.02 0.92 3.04 1.09 2.05 1.01 1.00 3.07 1.09 1.01 1.00 1.02 1.07 1.10 2.08 2.09 9.03 3.06 3.00 3.07
f1 (ppm)

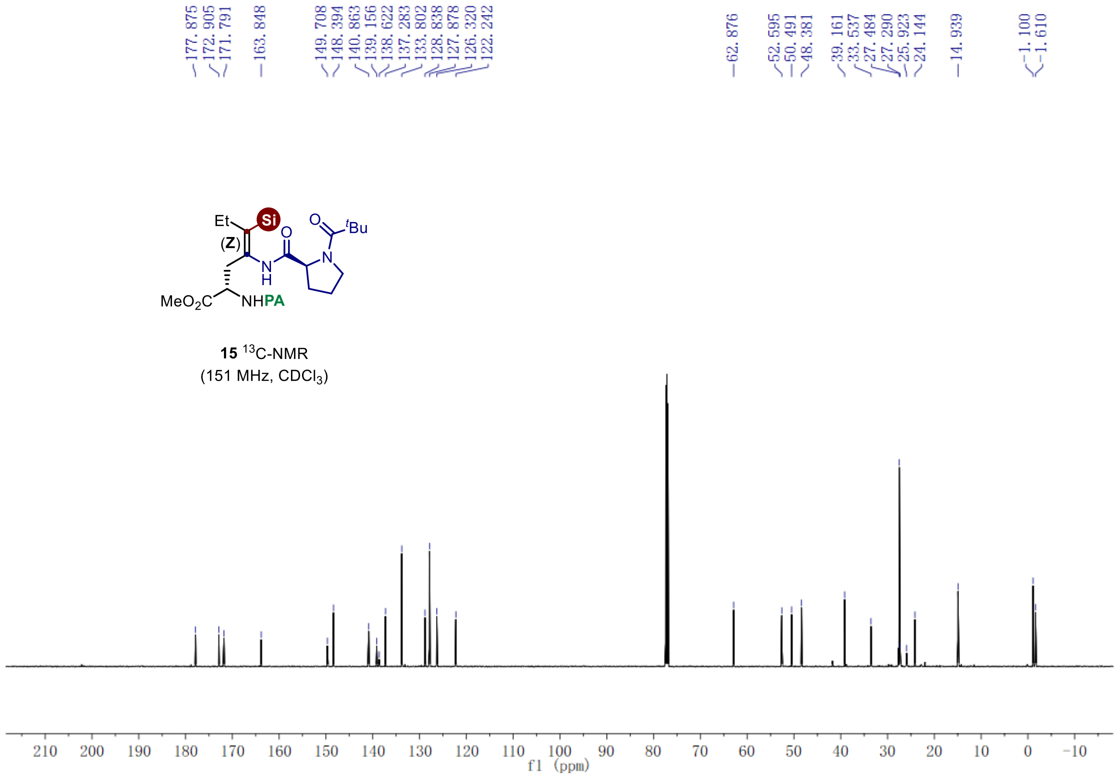
177.875 172.905 171.791 163.848 149.708 148.394 140.863 139.156 138.622 137.283 133.802 128.838 127.878 126.320 122.242 62.876 52.595 50.491 48.381 39.161 33.537 27.484 27.290 25.923 24.144 14.939 -1.100 -1.610
Et
Si
(Z)
O
O
tBu
N
N
H
MeO2C
NHPA
15 13C-NMR
(151 MHz, CDCl3)
f1 (ppm)

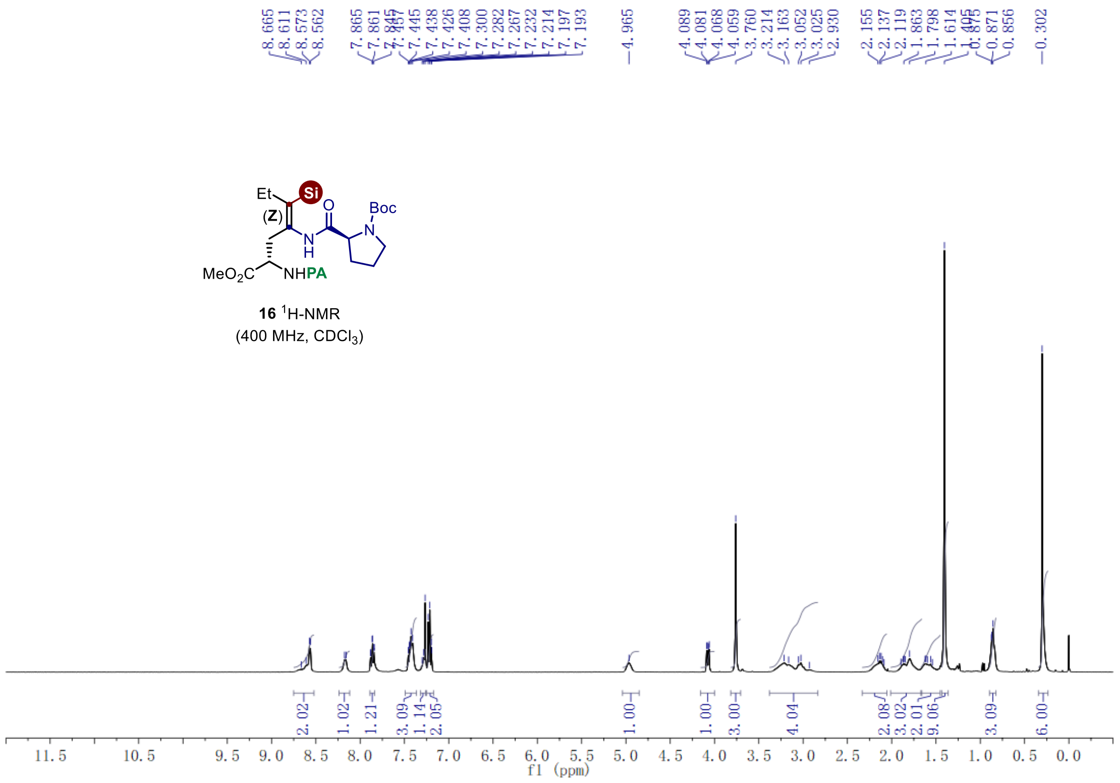
16 1H-NMR
(400 MHz, CDCl3)

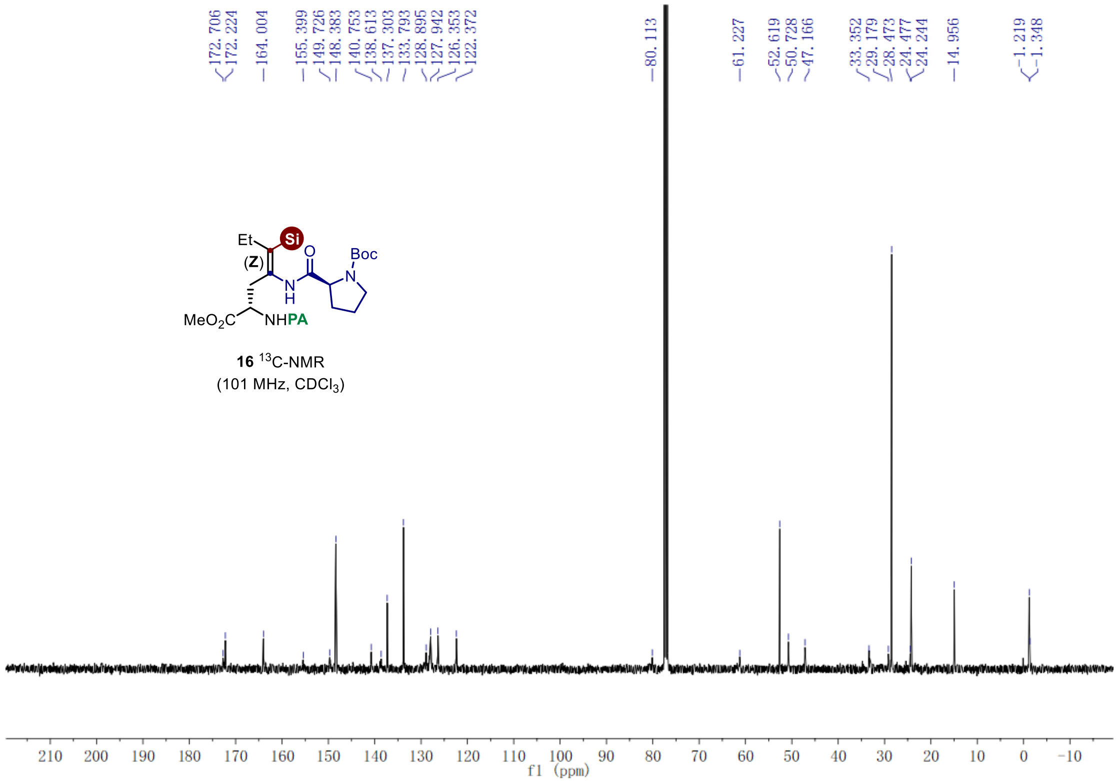
16 13C-NMR
(101 MHz, CDCl3)

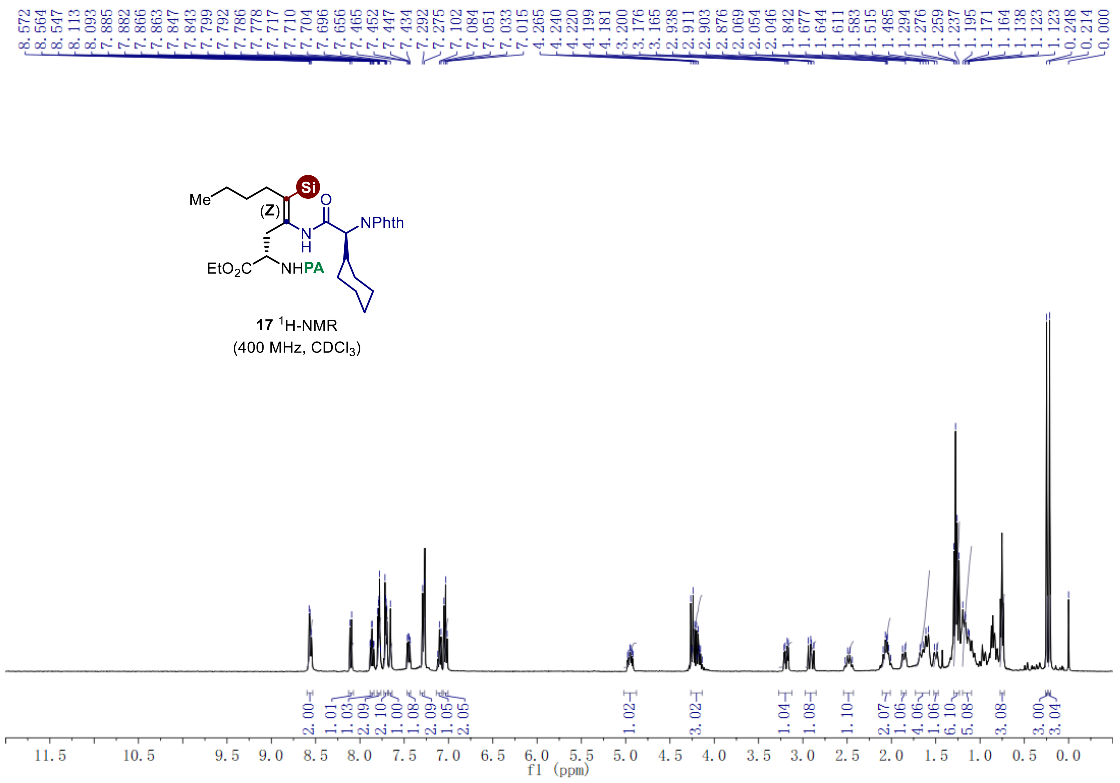

17 ^{1}H-NMR
(400 MHz, $CDCl_3$)

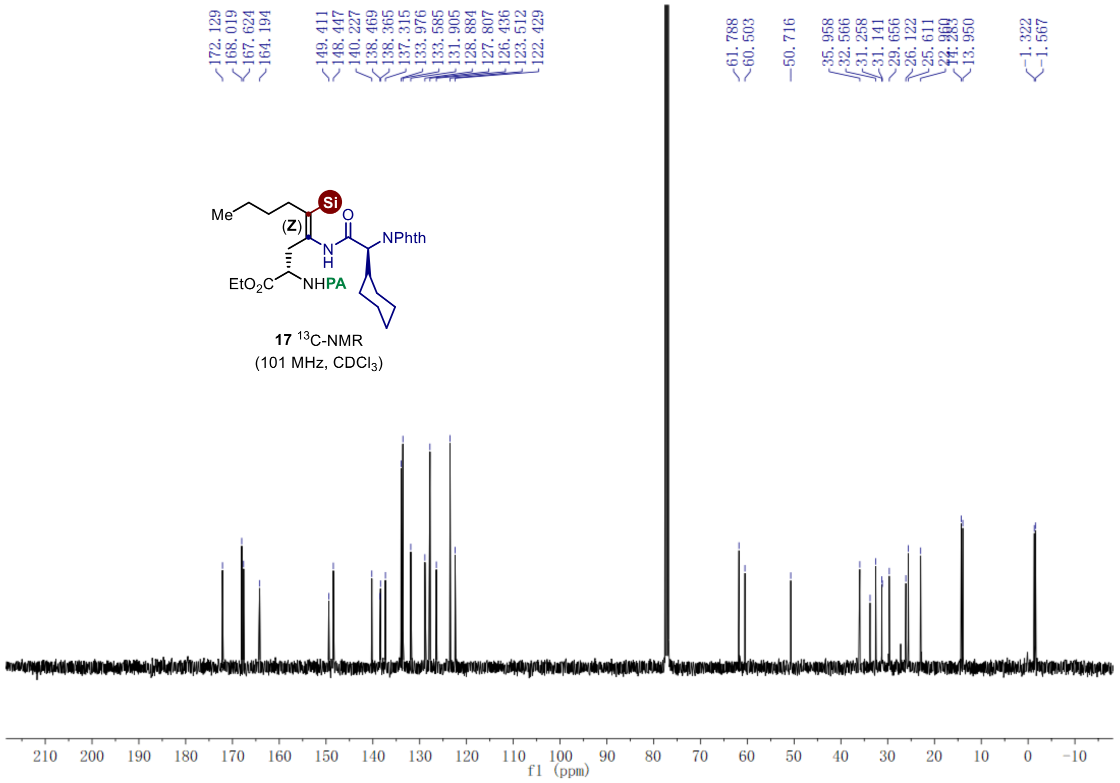

17 ^{13}C-NMR
(101 MHz, $CDCl_3$)

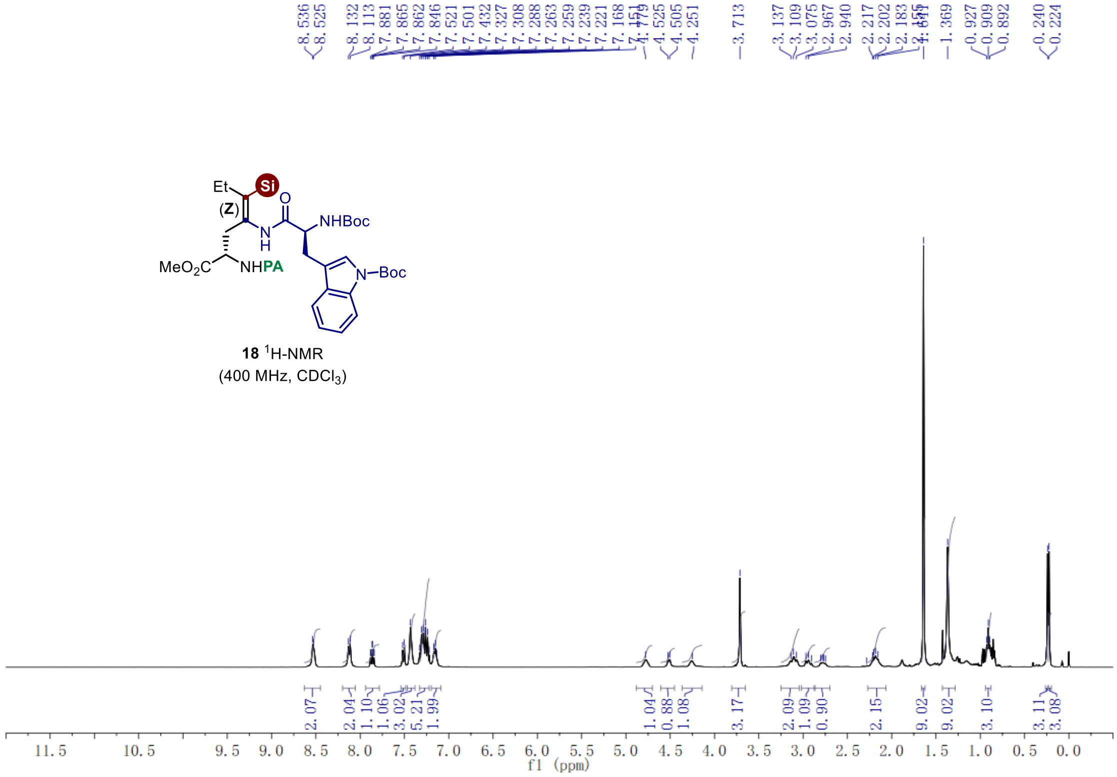

Et
Si
(Z)
O
NHBoc
N
H
MeO2C
NHPA
N–Boc
18 1H-NMR
(400 MHz, CDCl3)
8.536
8.525
8.132
8.113
7.881
7.865
7.862
7.846
7.521
7.501
7.432
7.327
7.308
7.288
7.263
7.259
7.239
7.221
7.168
4.525
4.505
4.251
3.713
3.137
3.109
3.075
2.967
2.940
2.217
2.202
2.183
1.369
0.927
0.909
0.892
0.240
0.224
f1 (ppm)

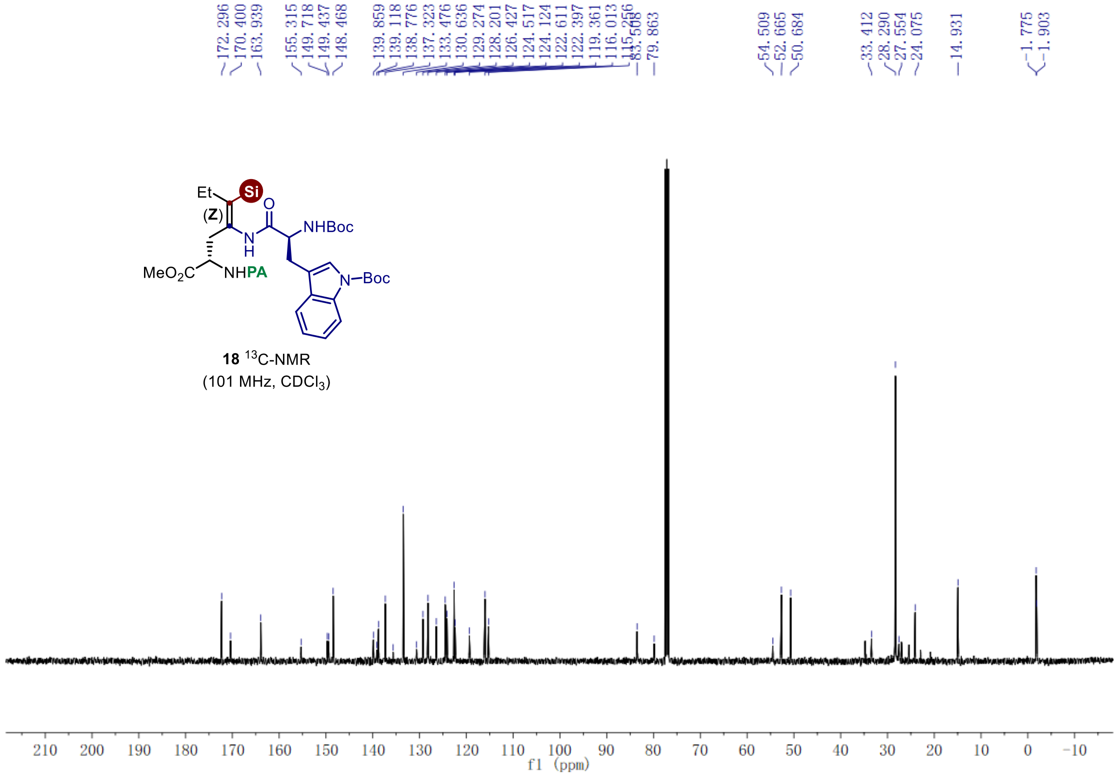

Et
Si
(Z)
O
NHBoc
N
H
MeO2C
NHPA
N–Boc
18 13C-NMR
(101 MHz, CDCl3)
172.296
170.400
163.939
155.315
149.718
149.437
148.468
139.859
139.118
138.776
137.323
133.476
130.636
129.274
128.201
126.427
124.517
124.124
122.611
122.397
119.361
116.013
79.863
54.509
52.665
50.684
33.412
28.290
27.554
24.075
14.931
-1.775
-1.903
f1 (ppm)

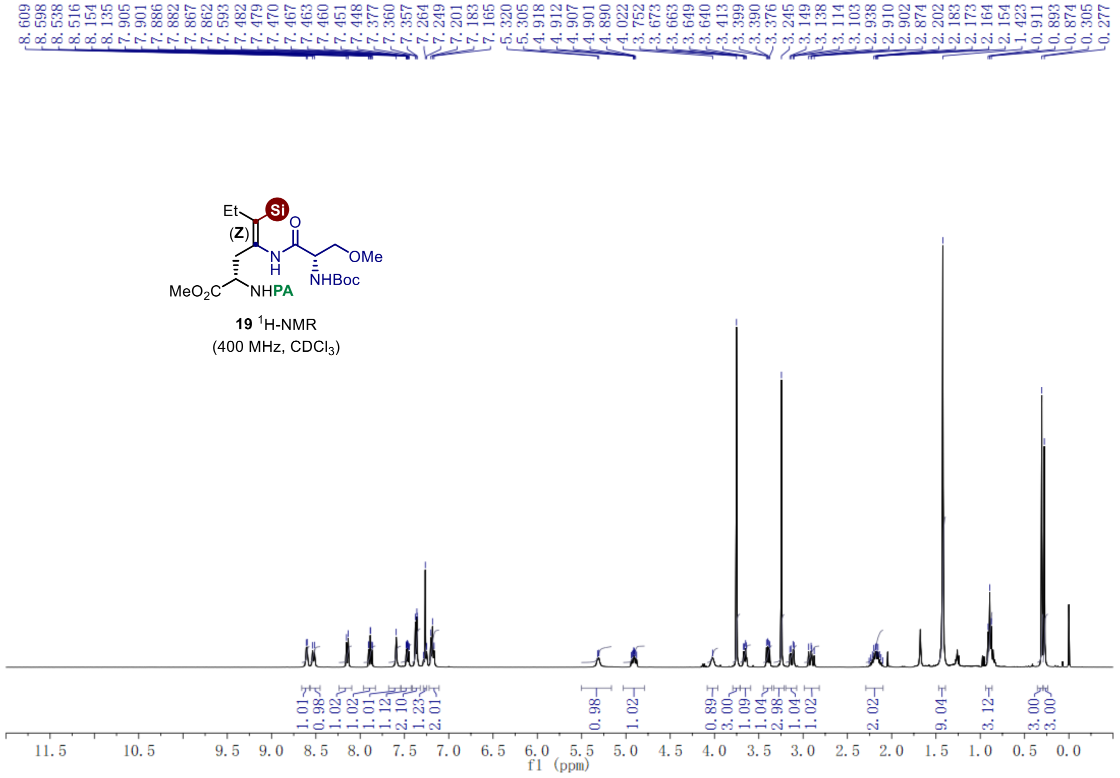

19 ^{1}H-NMR
(400 MHz, $CDCl_3$)

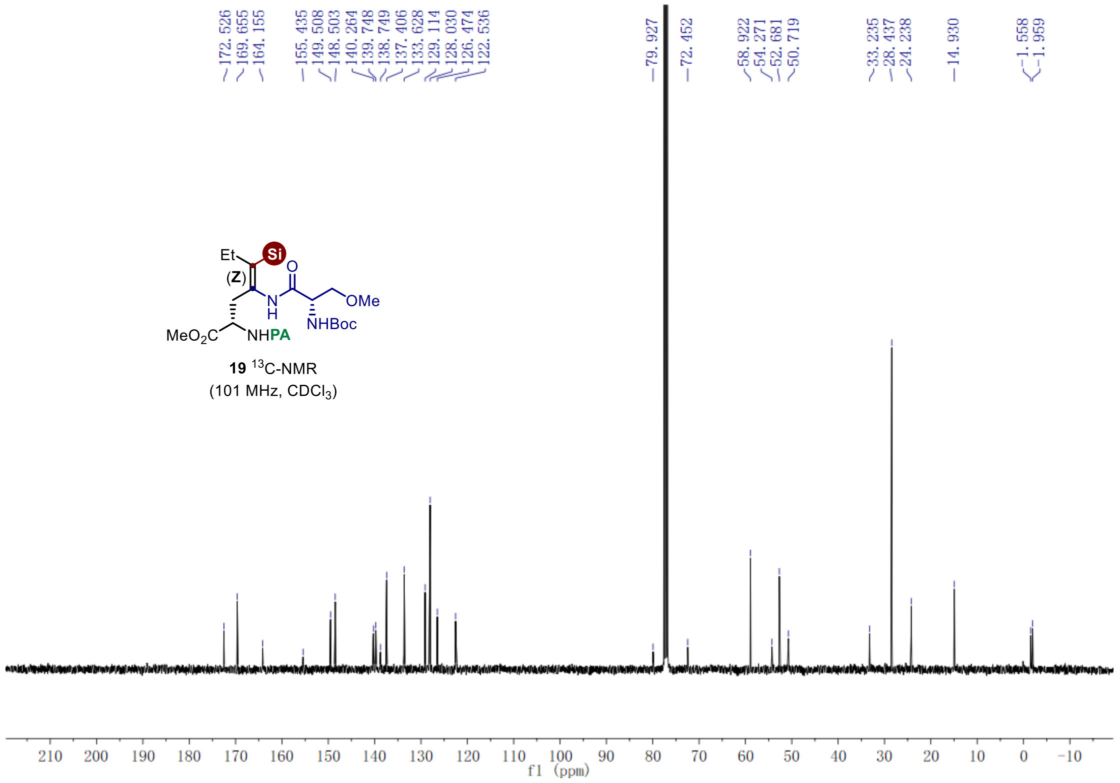

19 ^{13}C-NMR
(101 MHz, $CDCl_3$)

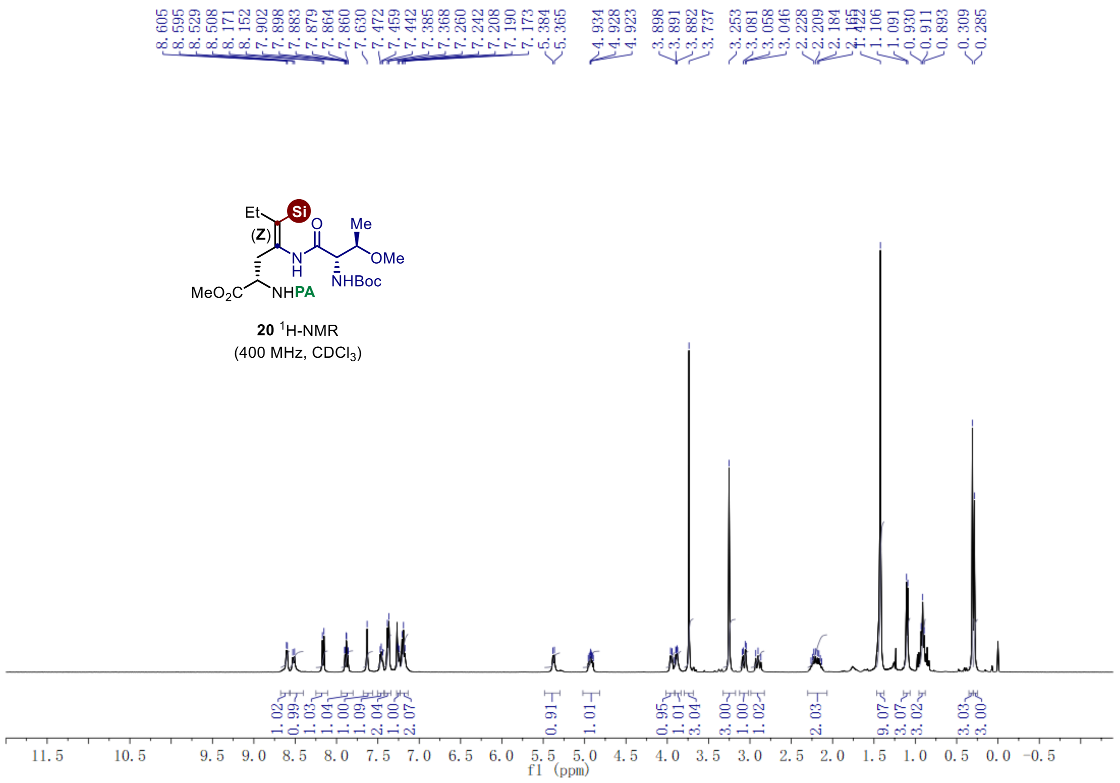
Et
Si
(Z)
O
Me
N
H
OMe
NHBoc
MeO2C
NHPA
20 1H-NMR
(400 MHz, CDCl3)
8.605 8.595 8.529 8.508 8.171 8.152 7.902 7.898 7.883 7.879 7.864 7.860 7.630 7.472 7.459 7.442 7.385 7.368 7.260 7.242 7.208 7.190 7.173 5.384 5.365 4.934 4.928 4.923 3.898 3.891 3.882 3.737 3.253 3.081 3.058 3.046 2.228 2.209 2.184 2.165 2.122 1.106 1.091 0.930 0.911 0.893 0.309 0.285
1.02 0.99 1.03 1.04 1.00 1.09 2.04 1.00 2.07 0.91 1.01 0.95 1.01 3.04 3.00 1.00 1.02 2.03 9.07 3.07 3.02 3.03 3.00
11.5 10.5 9.5 9.0 8.5 8.0 7.5 7.0 6.5 6.0 5.5 5.0 4.5 4.0 3.5 3.0 2.5 2.0 1.5 1.0 0.5 0.0 -0.5
f1 (ppm)

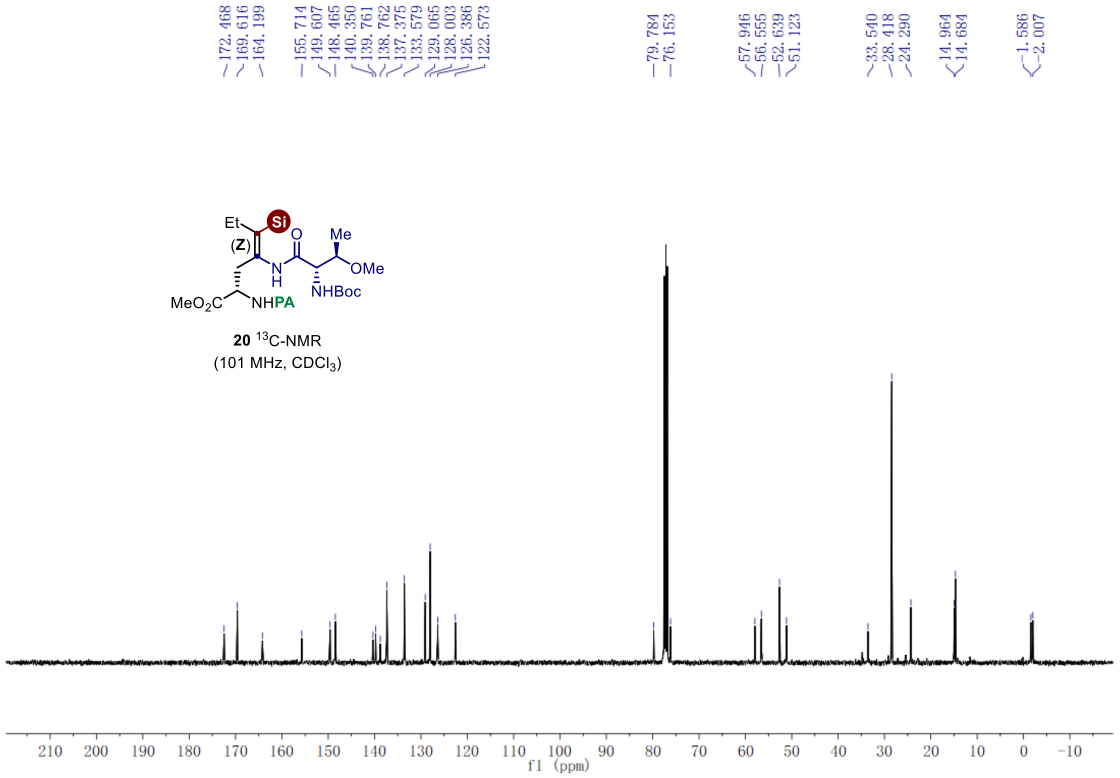
172.468 169.616 164.199 155.714 149.607 148.465 140.350 139.761 138.762 137.375 133.579 129.065 128.003 126.386 122.573 79.784 76.153 57.946 56.555 52.639 51.123 33.540 28.418 24.290 14.964 14.684 -1.586 -2.007
Et
Si
(Z)
O
Me
N
H
OMe
NHBoc
MeO2C
NHPA
20 13C-NMR
(101 MHz, CDCl3)
210 200 190 180 170 160 150 140 130 120 110 100 90 80 70 60 50 40 30 20 10 0 -10
f1 (ppm)

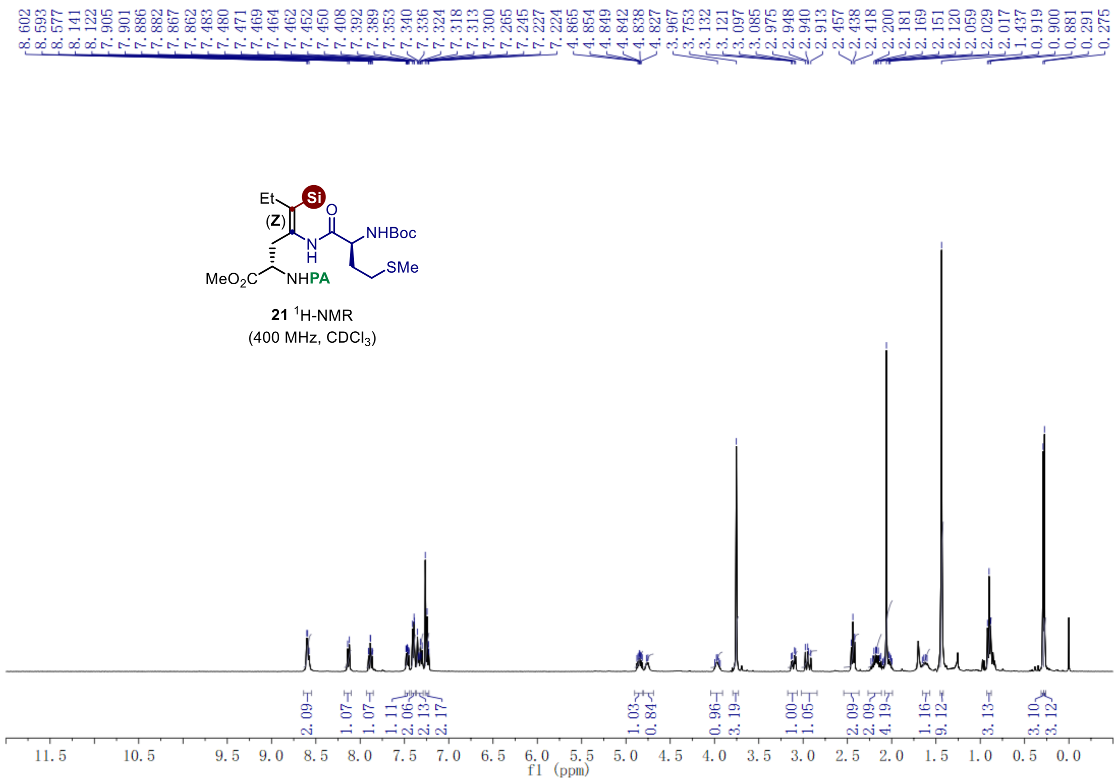
8.602
8.593
8.577
8.141
8.122
7.905
7.901
7.886
7.882
7.867
7.862
7.483
7.480
7.471
7.469
7.464
7.462
7.452
7.450
7.408
7.392
7.389
7.353
7.340
7.336
7.324
7.318
7.313
7.300
7.265
7.245
7.227
7.224
4.865
4.854
4.849
4.842
4.838
4.827
3.967
3.753
3.132
3.121
3.097
3.085
2.975
2.948
2.940
2.913
2.457
2.438
2.418
2.200
2.181
2.169
2.151
2.120
2.059
2.029
2.017
1.437
0.919
0.900
0.881
0.291
0.275
Et
Si
(Z)
O
NHBoc
N
H
MeO2C
NHPA
SMe
21 1H-NMR
(400 MHz, CDCl3)
2.09
1.07
1.07
1.11
2.06
2.13
2.17
1.03
0.84
0.96
3.19
1.00
1.05
2.09
2.09
4.19
1.16
9.12
3.13
3.10
3.12
f1 (ppm)

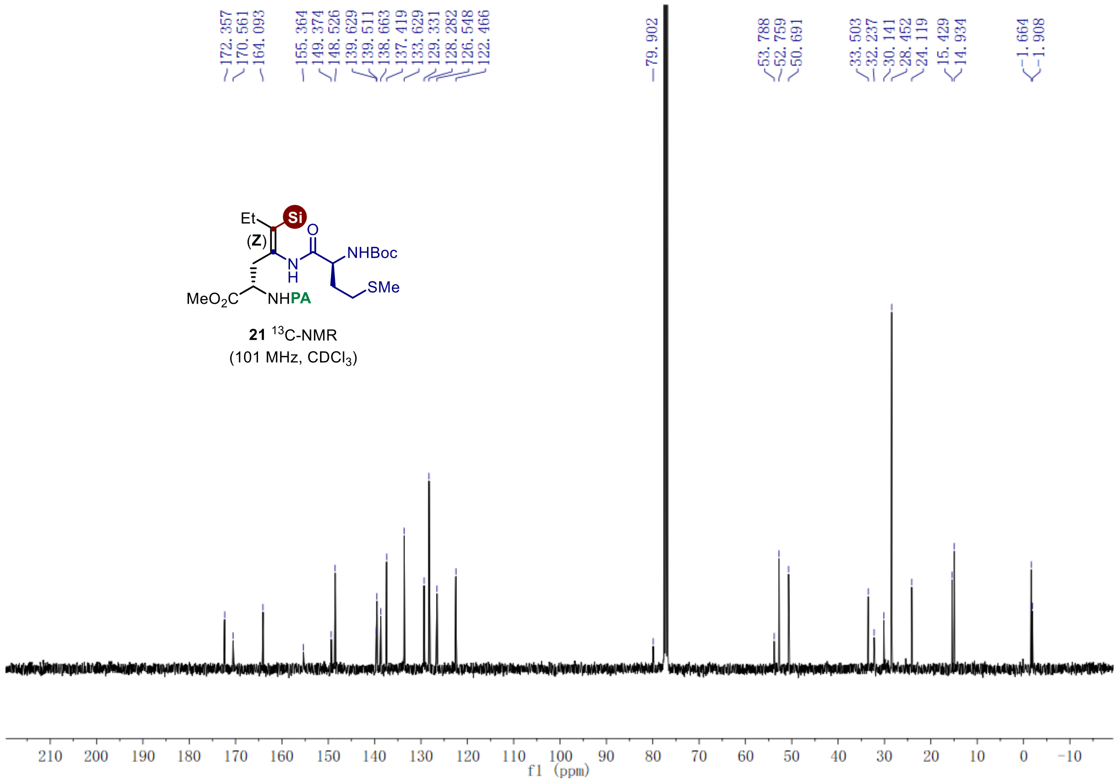
172.357
170.561
164.093
155.364
149.374
148.526
139.629
139.511
138.663
137.419
133.629
129.331
128.282
126.548
122.466
79.902
53.788
52.759
50.691
33.503
32.237
30.141
28.452
24.119
15.429
14.934
-1.664
-1.908
Et
Si
(Z)
O
NHBoc
N
H
MeO2C
NHPA
SMe
21 13C-NMR
(101 MHz, CDCl3)
f1 (ppm)

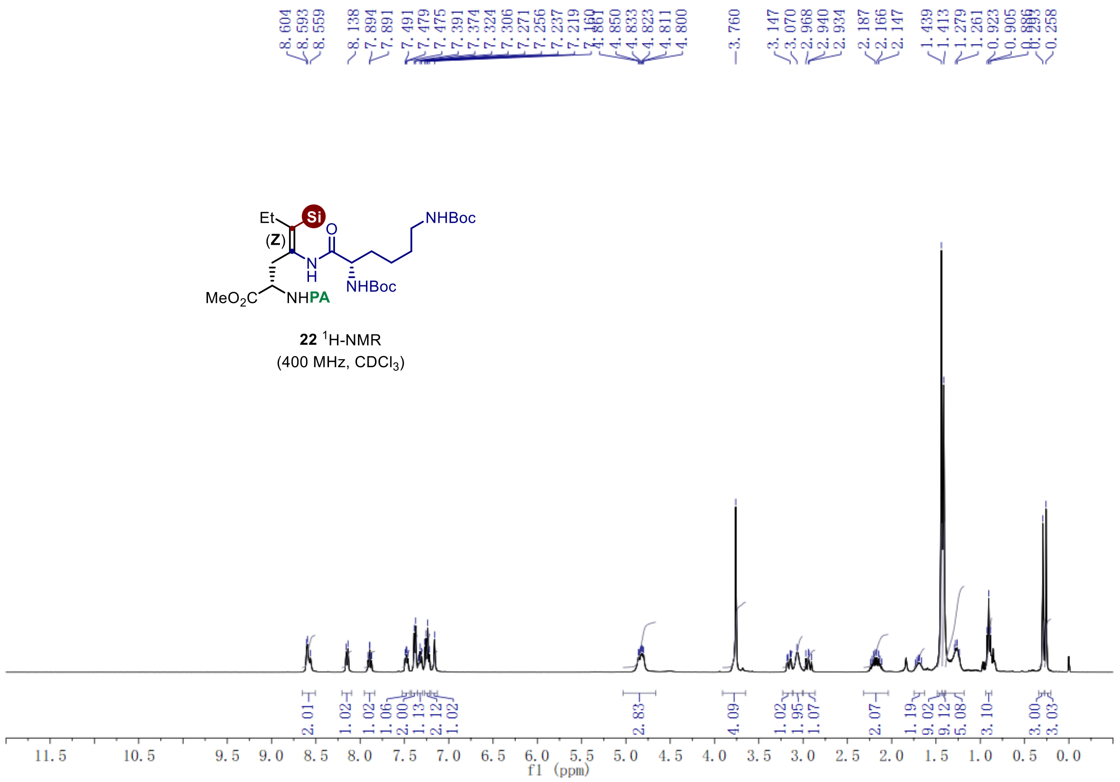

22 ^{1}H-NMR
(400 MHz, $CDCl_3$)

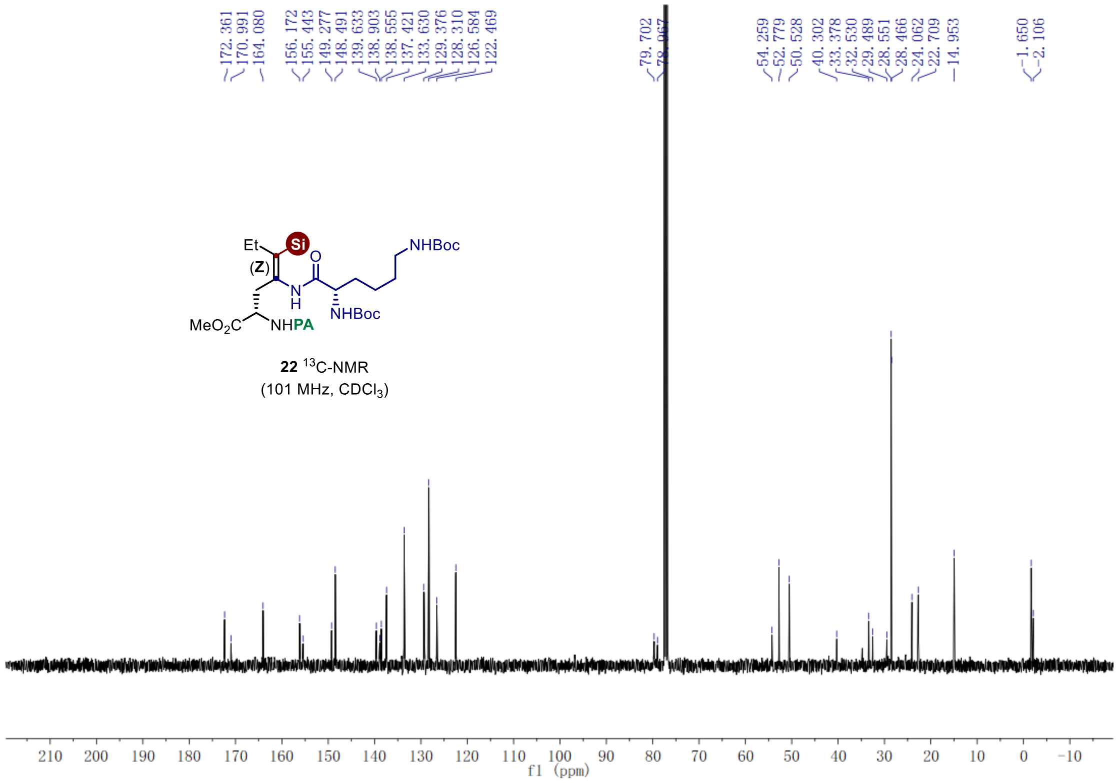

22 ^{13}C-NMR
(101 MHz, $CDCl_3$)

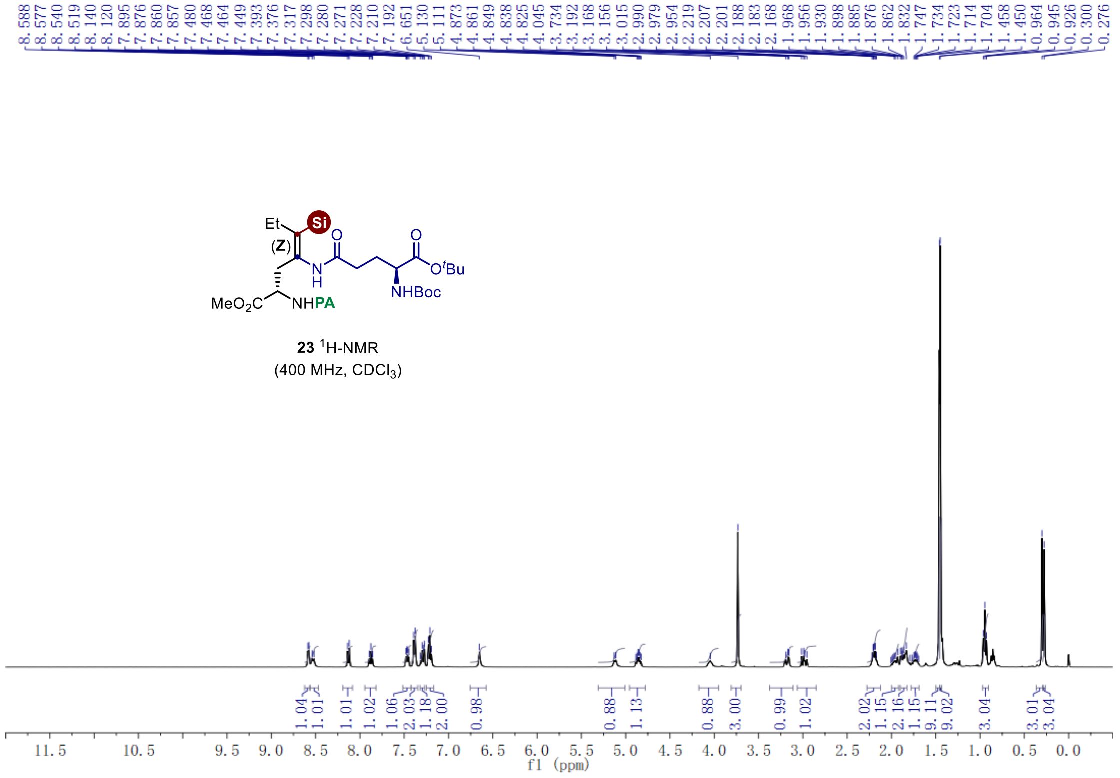
8.588 8.577 8.540 8.519 8.140 8.120 7.895 7.876 7.860 7.857 7.480 7.468 7.464 7.449 7.393 7.376 7.317 7.298 7.280 7.271 7.228 7.210 7.192 6.651 5.130 5.111 4.873 4.861 4.849 4.838 4.825 4.045 3.734 3.192 3.168 3.156 3.015 2.990 2.979 2.954 2.219 2.207 2.201 2.188 2.183 2.168 1.968 1.956 1.930 1.898 1.885 1.876 1.862 1.832 1.747 1.734 1.723 1.714 1.704 1.458 1.450 0.964 0.945 0.926 0.300 0.276
Et
Si
(Z)
O
N
H
O
O^{t}Bu
MeO$_2$C
NHPA
NHBoc
23 ^{1}H-NMR
(400 MHz, CDCl$_3$)
1.04 1.01 1.01 1.02 1.06 2.03 1.18 2.00 0.98 0.88 1.13 0.88 3.00 0.99 1.02 2.02 1.15 2.16 1.15 9.11 9.02 3.04 3.01 3.04
11.5 10.5 9.5 9.0 8.5 8.0 7.5 7.0 6.5 6.0 5.5 5.0 4.5 4.0 3.5 3.0 2.5 2.0 1.5 1.0 0.5 0.0
f1 (ppm)

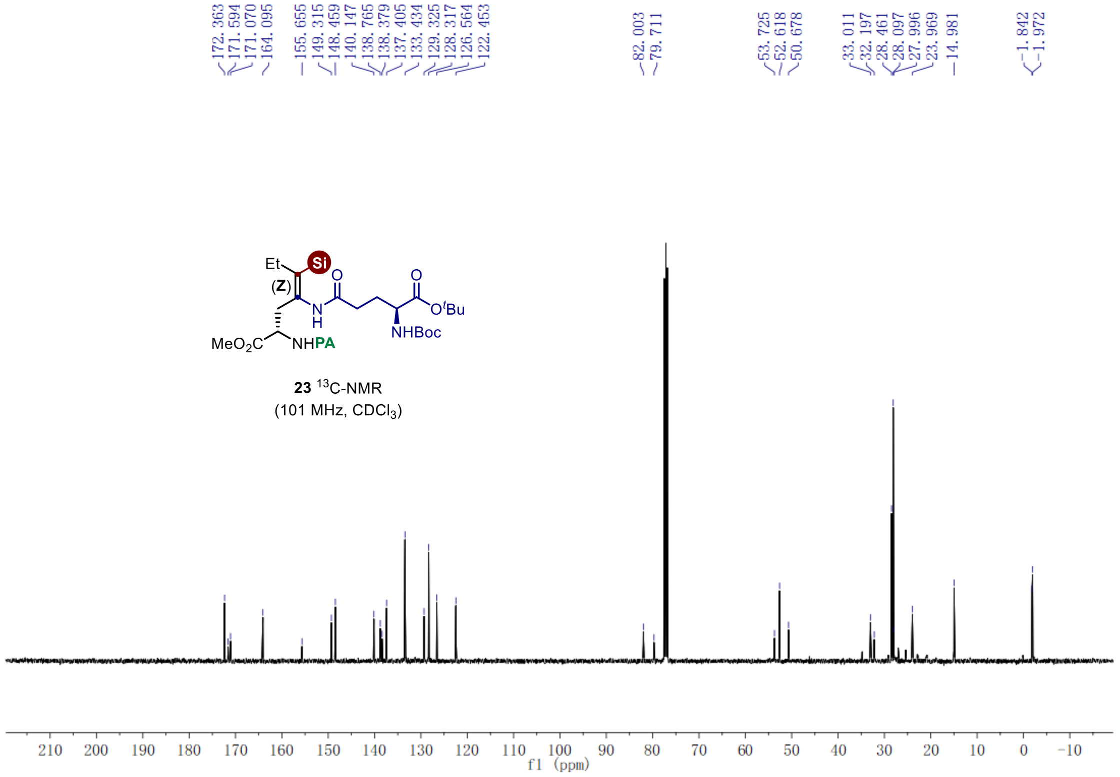
172.363 171.594 171.070 164.095 155.655 149.315 148.459 140.147 138.765 138.379 137.405 133.434 129.325 128.317 126.564 122.453 82.003 79.711 53.725 52.618 50.678 33.011 32.197 28.461 28.097 27.996 23.969 14.981 -1.842 -1.972
Et
Si
(Z)
O
N
H
O
O^{t}Bu
MeO$_2$C
NHPA
NHBoc
23 ^{13}C-NMR
(101 MHz, CDCl$_3$)
210 200 190 180 170 160 150 140 130 120 110 100 90 80 70 60 50 40 30 20 10 0 -10
f1 (ppm)

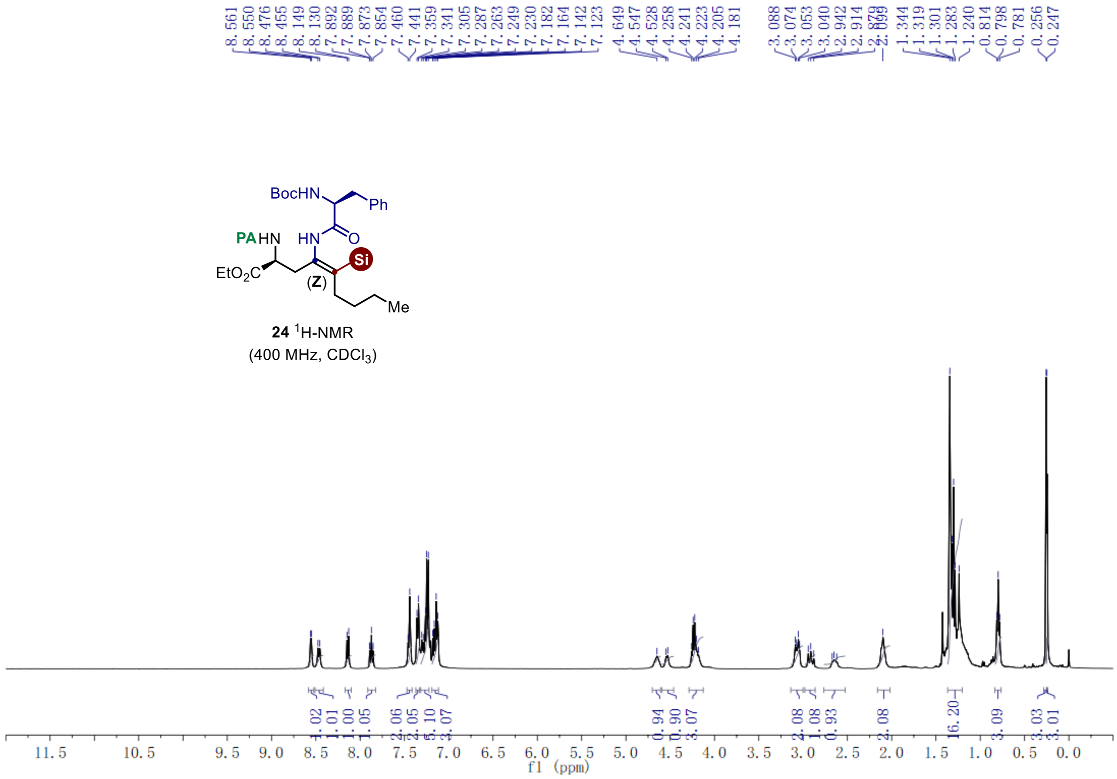
8.561 8.550 8.476 8.455 8.149 8.130 7.892 7.889 7.873 7.854 7.460 7.441 7.359 7.341 7.305 7.287 7.263 7.249 7.230 7.182 7.164 7.142 7.123
4.649 4.547 4.528 4.258 4.241 4.223 4.205 4.181
3.088 3.074 3.053 3.040 2.942 2.914
1.344 1.319 1.301 1.283 1.240 0.814 0.798 0.781 0.256 0.247
BocHN
Ph
PA HN
HN
O
Si
EtO$_2$C
(Z)
Me
24 ^{1}H-NMR
(400 MHz, $CDCl_3$)
1.02 1.01 1.00 1.05 2.06 2.05 5.10 3.07 0.94 0.90 3.07 2.08 1.08 0.93 2.08 16.20 3.09 3.03 3.01
11.5 10.5 9.5 9.0 8.5 8.0 7.5 7.0 6.5 6.0 5.5 5.0 4.5 4.0 3.5 3.0 2.5 2.0 1.5 1.0 0.5 0.0
f1 (ppm)

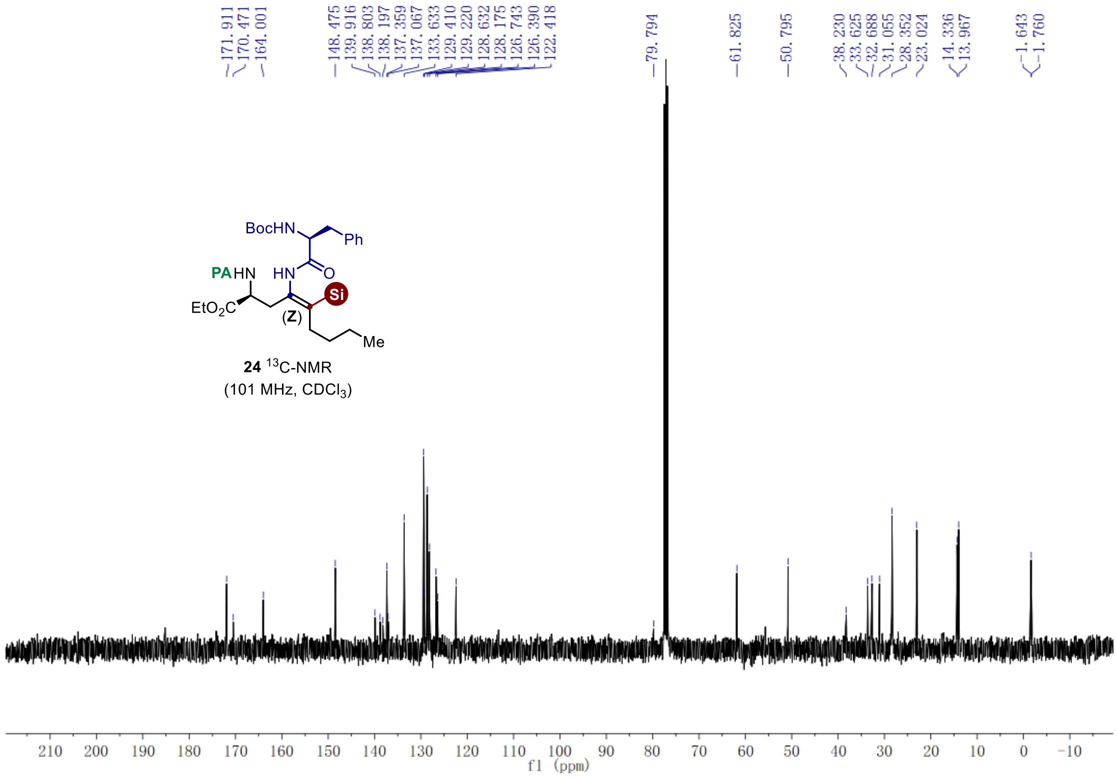
171.911 170.471 164.001
148.475 139.916 138.803 138.197 137.359 137.067 133.633 129.410 129.220 128.632 128.175 126.743 126.390 122.418
79.794
61.825
50.795
38.230 33.625 32.688 31.055 28.352 23.024 14.336 13.967
1.643 -1.760
BocHN
Ph
PA HN
HN
O
Si
EtO$_2$C
(Z)
Me
24 ^{13}C-NMR
(101 MHz, $CDCl_3$)
210 200 190 180 170 160 150 140 130 120 110 100 90 80 70 60 50 40 30 20 10 0 -10
f1 (ppm)

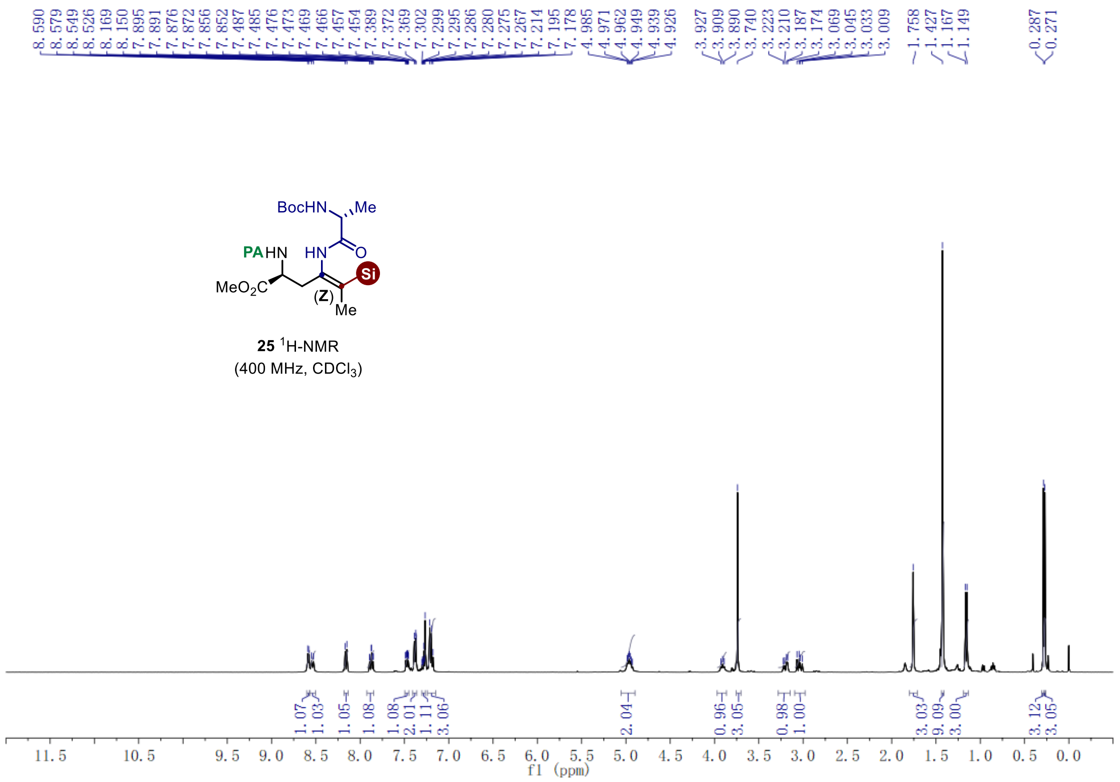
BocHN
Me
PAHN
HN
O
Si
MeO2C
(Z)
Me
25 1H-NMR
(400 MHz, CDCl3)
f1 (ppm)

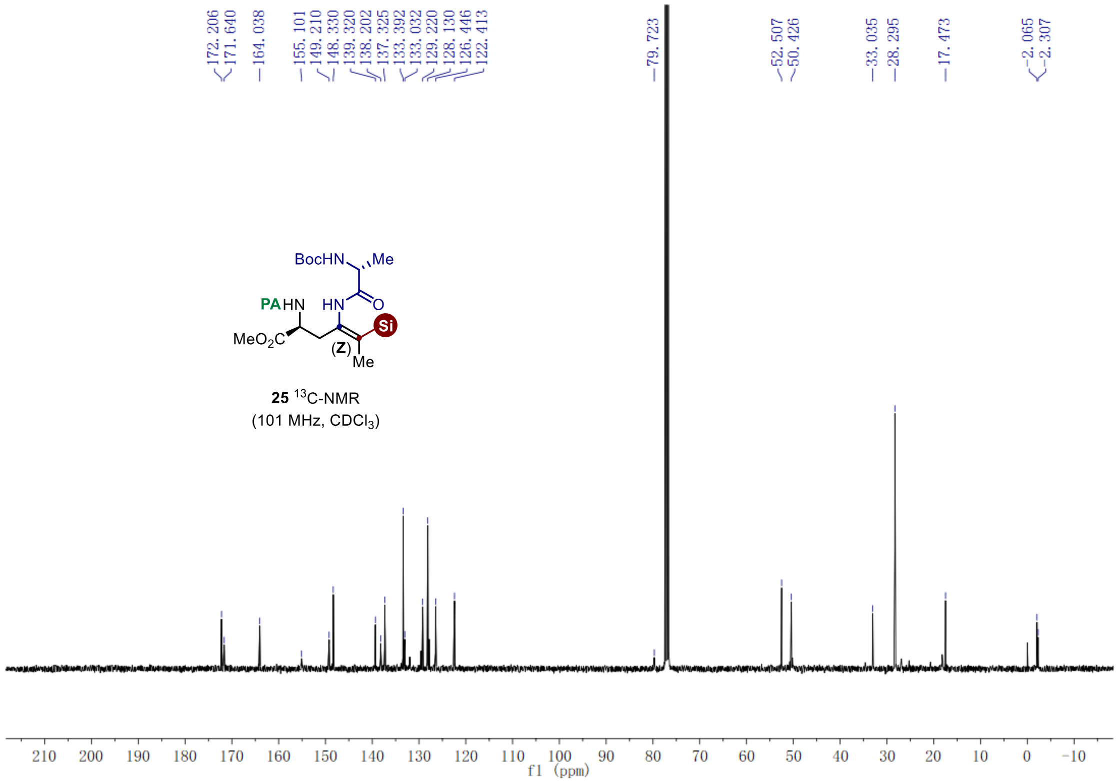
BocHN
Me
PAHN
HN
O
Si
MeO2C
(Z)
Me
25 13C-NMR
(101 MHz, CDCl3)
f1 (ppm)

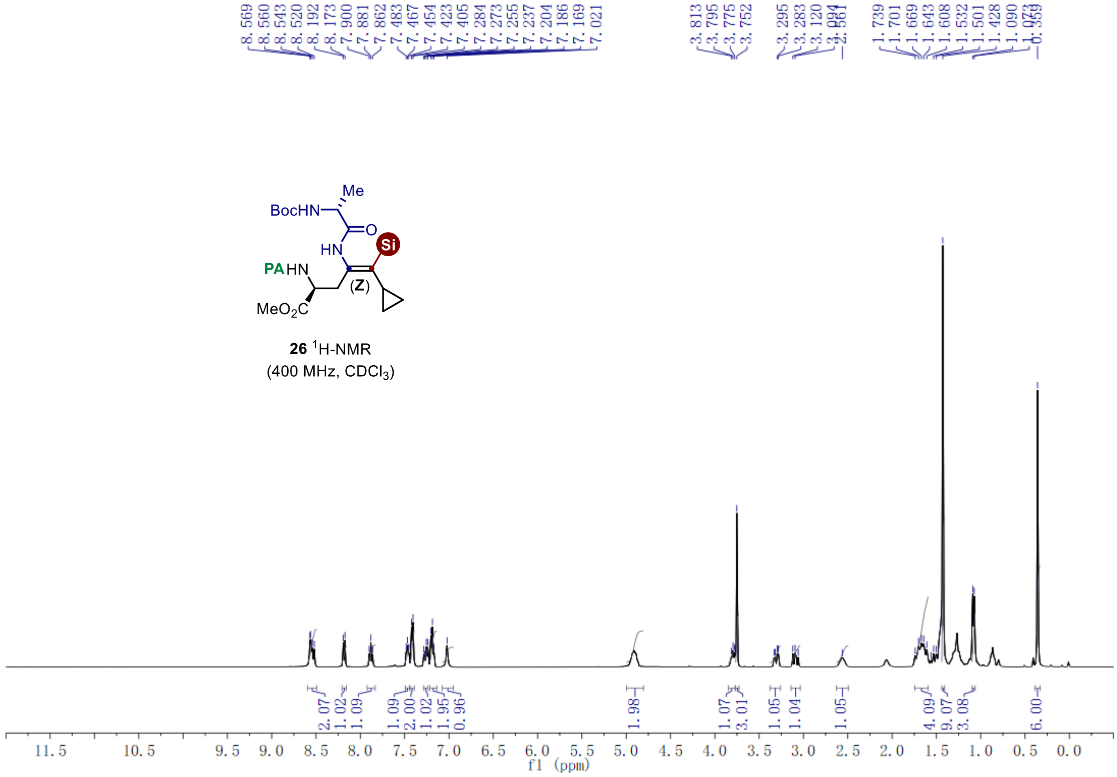
8.569 8.560 8.543 8.520 8.192 8.173 7.900 7.881 7.862 7.483 7.467 7.454 7.423 7.405 7.284 7.273 7.255 7.237 7.204 7.186 7.169 7.021
3.813 3.795 3.775 3.752 3.295 3.283 3.120 2.561
1.739 1.701 1.669 1.643 1.608 1.532 1.501 1.428 1.090
Me
BocHN
O
HN
Si
PAHN
(Z)
MeO2C
26 1H-NMR
(400 MHz, CDCl3)
2.07 1.02 1.09 1.09 2.00 1.02 1.95 0.96 1.98 1.07 3.01 1.05 1.04 1.05 4.09 9.07 3.08 6.00
11.5 10.5 9.5 9.0 8.5 8.0 7.5 7.0 6.5 6.0 5.5 5.0 4.5 4.0 3.5 3.0 2.5 2.0 1.5 1.0 0.5 0.0
f1 (ppm)

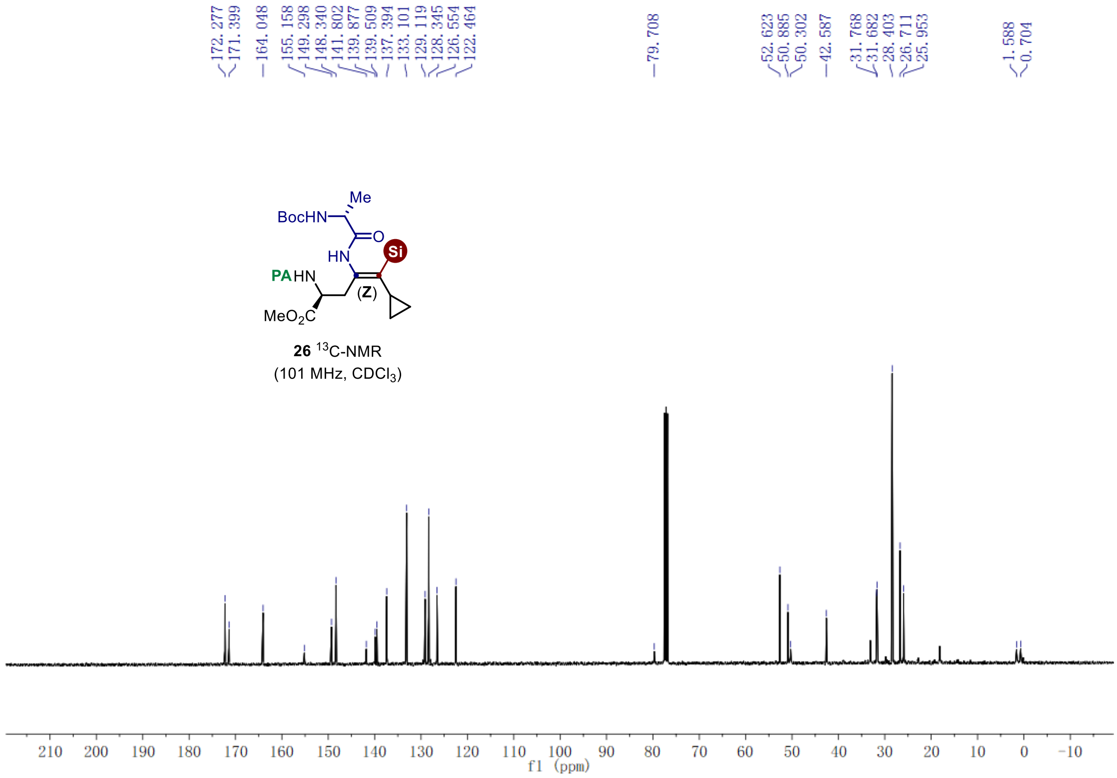
172.277 171.399 164.048 155.158 149.298 148.340 141.802 139.877 139.509 137.394 133.101 129.119 128.345 126.554 122.464
79.708
52.623 50.885 50.302 42.587 31.768 31.682 28.403 26.711 25.953
1.588 0.704
Me
BocHN
O
HN
Si
PAHN
(Z)
MeO2C
26 13C-NMR
(101 MHz, CDCl3)
210 200 190 180 170 160 150 140 130 120 110 100 90 80 70 60 50 40 30 20 10 0 -10
f1 (ppm)

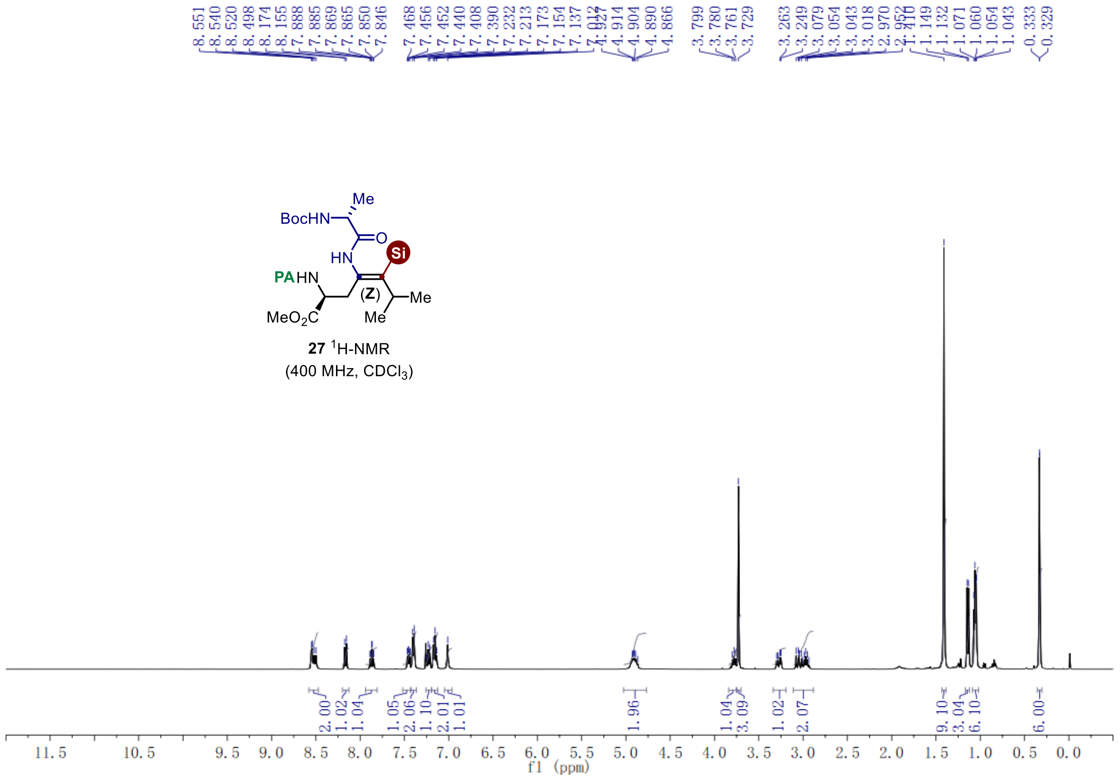
8.551 8.540 8.520 8.498 8.174 8.155 7.888 7.885 7.869 7.865 7.850 7.846 7.468 7.456 7.452 7.440 7.408 7.390 7.232 7.213 7.173 7.154 7.137 4.914 4.904 4.890 4.866 3.799 3.780 3.761 3.729 3.263 3.249 3.079 3.054 3.043 3.018 2.970 1.149 1.132 1.071 1.060 1.054 1.043 0.333 0.329
BocHN
Me
O
HN
Si
PA HN
(Z)
Me
MeO2C
Me
27 1H-NMR
(400 MHz, CDCl3)
2.00 1.02 1.04 1.05 2.06 1.10 2.01 1.01 1.96 1.04 3.09 1.02 2.07 9.10 3.04 6.10 6.00
11.5 10.5 9.5 9.0 8.5 8.0 7.5 7.0 6.5 6.0 5.5 5.0 4.5 4.0 3.5 3.0 2.5 2.0 1.5 1.0 0.5 0.0
f1 (ppm)

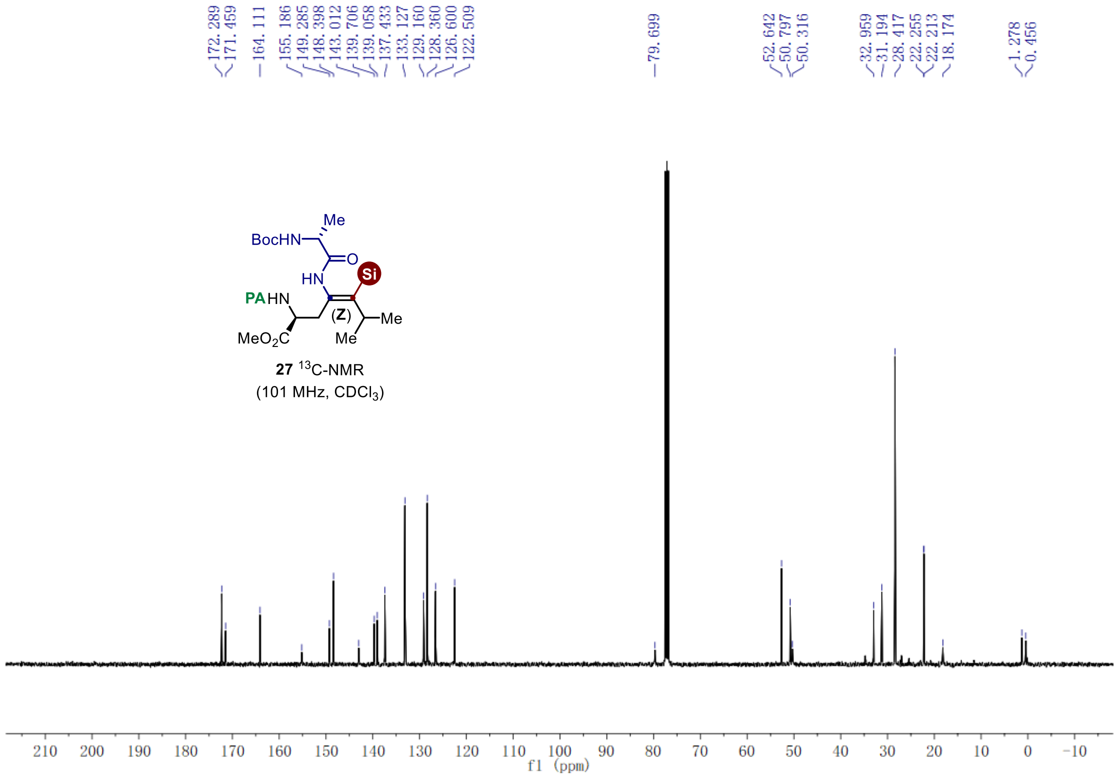
172.289 171.459 164.111 155.186 149.285 148.398 143.012 139.706 139.058 137.433 133.127 129.160 128.360 126.600 122.509 79.699 52.642 50.797 50.316 32.959 31.194 28.417 22.255 22.213 18.174 1.278 0.456
BocHN
Me
O
HN
Si
PA HN
(Z)
Me
MeO2C
Me
27 13C-NMR
(101 MHz, CDCl3)
210 200 190 180 170 160 150 140 130 120 110 100 90 80 70 60 50 40 30 20 10 0 -10
f1 (ppm)

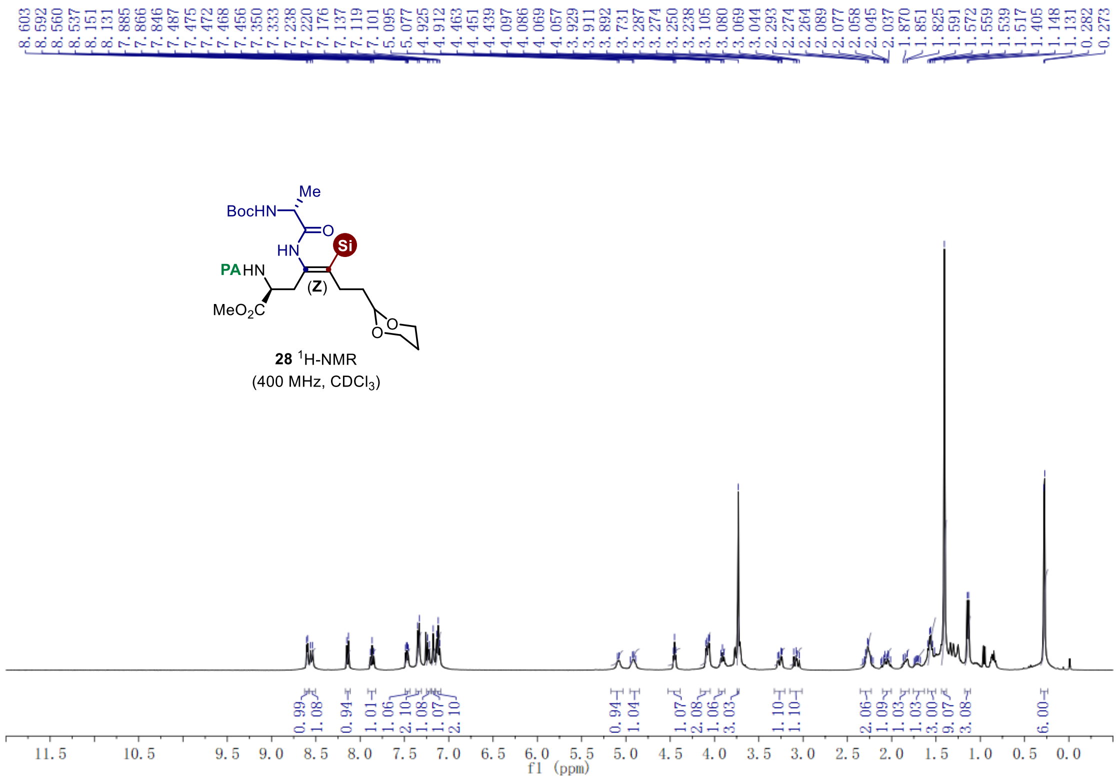

BocHN
Me
O
HN
Si
PAHN
(Z)
MeO2C
O
O
28 1H-NMR
(400 MHz, CDCl3)
f1 (ppm)

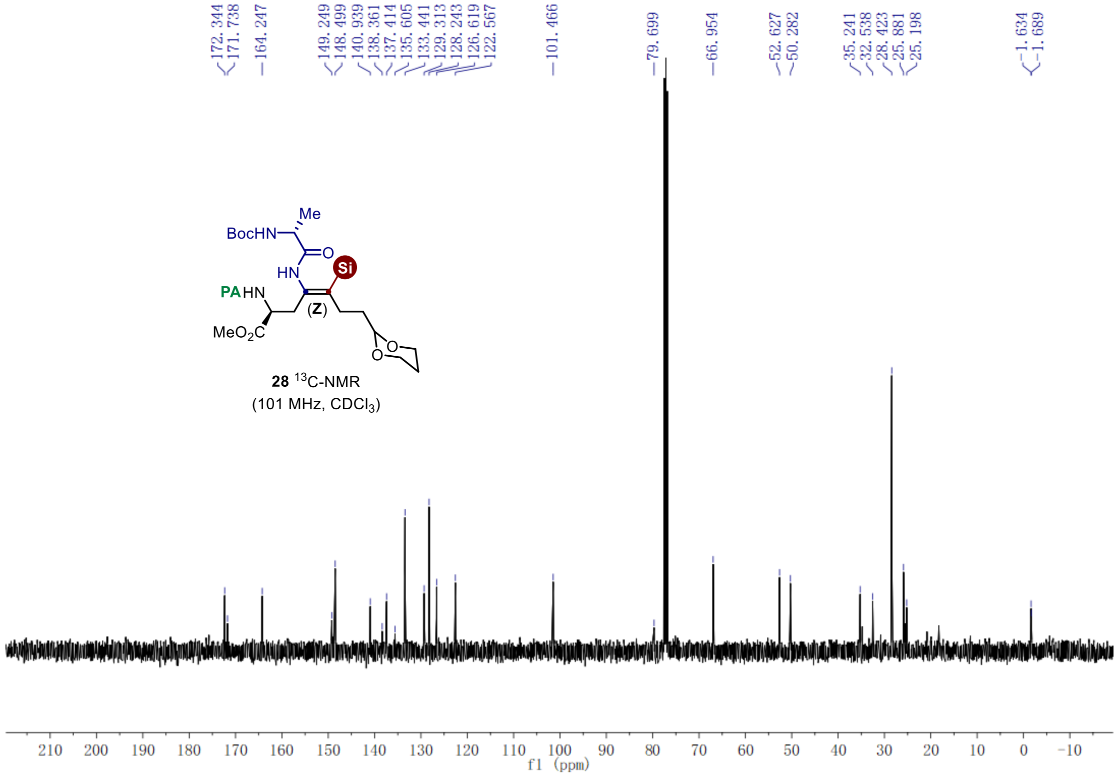

BocHN
Me
O
HN
Si
PAHN
(Z)
MeO2C
O
O
28 13C-NMR
(101 MHz, CDCl3)
f1 (ppm)

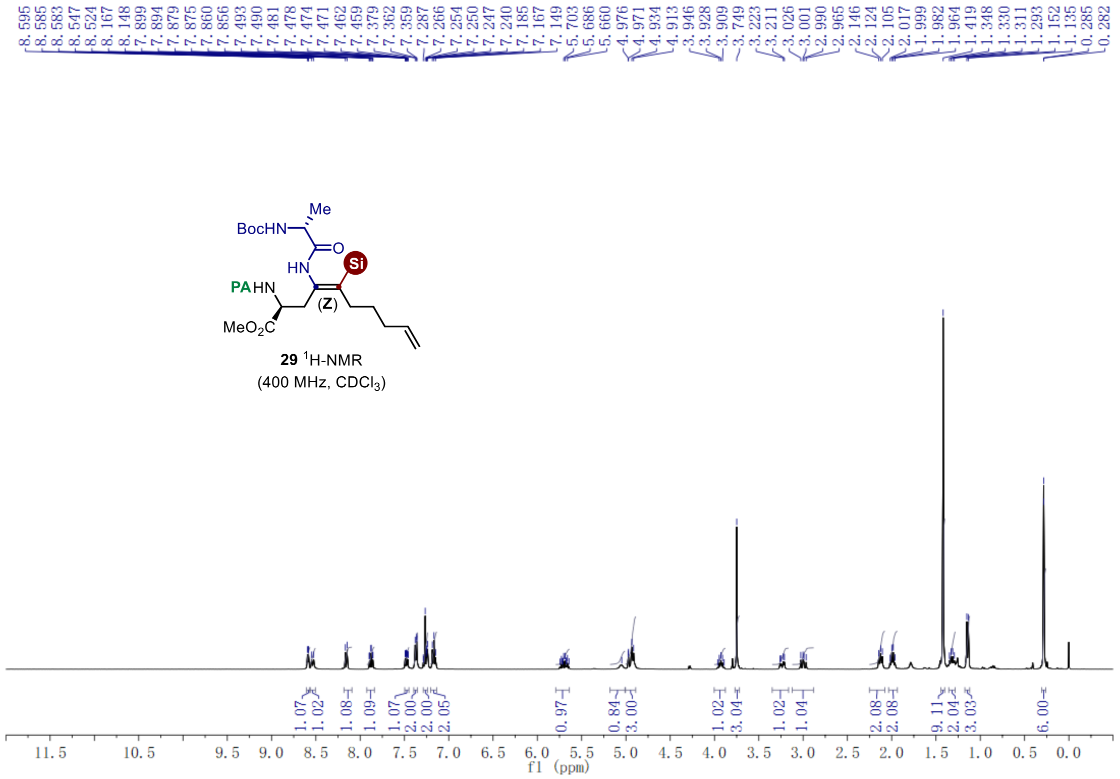
BocHN
Me
O
HN
Si
PAHN
(Z)
MeO2C
29 1H-NMR
(400 MHz, CDCl3)
f1 (ppm)

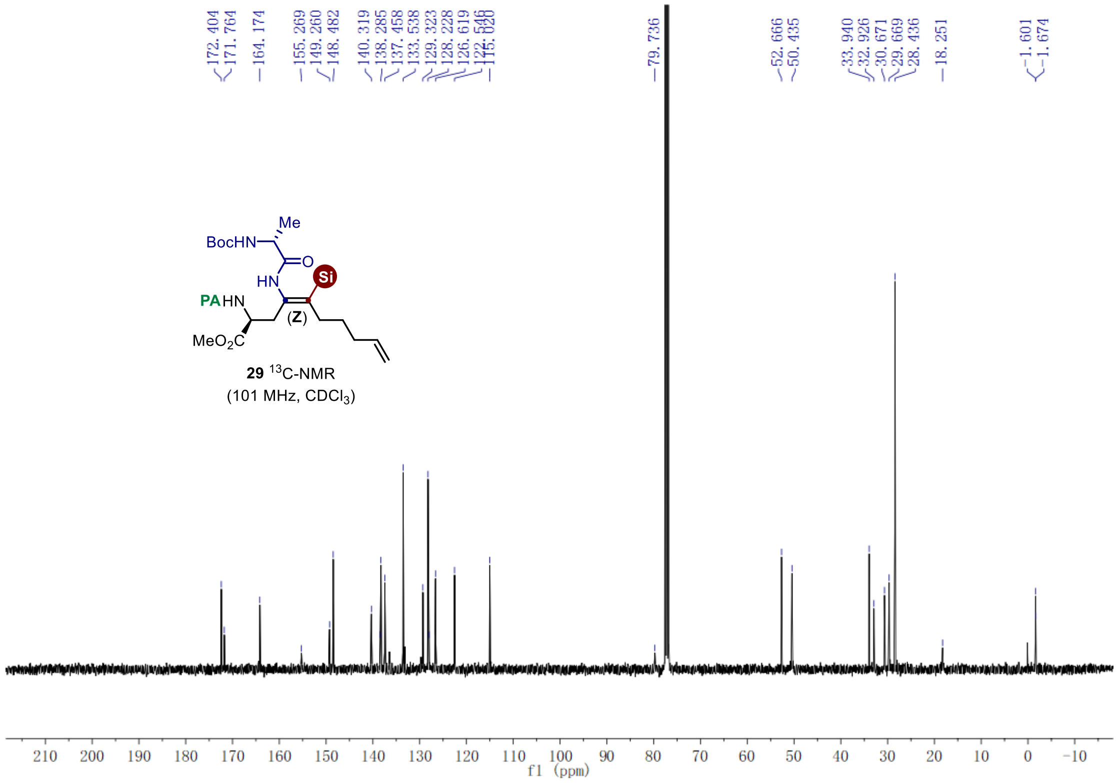
BocHN
Me
O
HN
Si
PAHN
(Z)
MeO2C
29 13C-NMR
(101 MHz, CDCl3)
f1 (ppm)

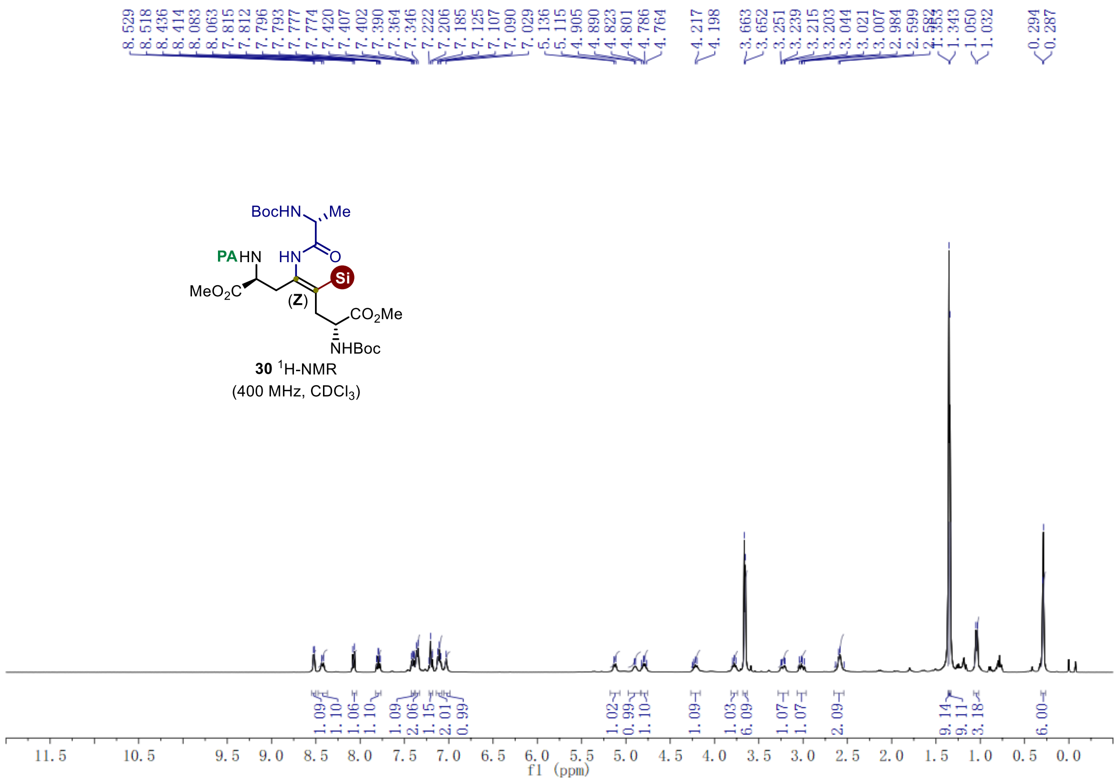
BocHN
Me
PA HN
HN
O
Si
MeO2C
(Z)
CO2Me
NHBoc
30 1H-NMR
(400 MHz, CDCl3)
8.529 8.518 8.436 8.414 8.083 8.063 7.815 7.812 7.796 7.793 7.777 7.774 7.420 7.407 7.402 7.390 7.364 7.346 7.222 7.206 7.185 7.125 7.107 7.090 7.029 5.136 5.115 4.905 4.890 4.823 4.801 4.786 4.764 4.217 4.198 3.663 3.652 3.251 3.239 3.215 3.203 3.044 3.021 3.007 2.984 2.599 2.582 1.343 1.050 1.032 0.294 0.287
1.09 1.10 1.06 1.10 1.09 2.06 1.15 2.01 0.99 1.02 0.99 1.10 1.09 1.03 6.09 1.07 1.07 2.09 9.14 9.11 3.18 6.00
f1 (ppm)

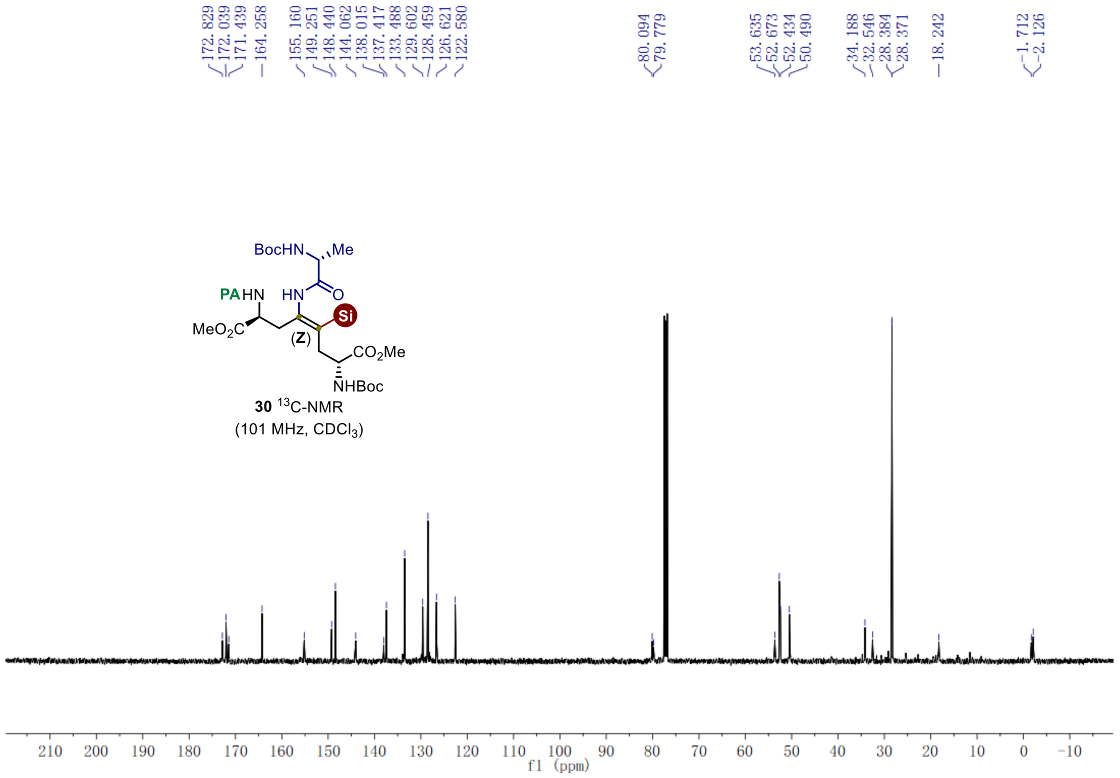
BocHN
Me
PA HN
HN
O
Si
MeO2C
(Z)
CO2Me
NHBoc
30 13C-NMR
(101 MHz, CDCl3)
172.829 172.039 171.439 164.258 155.160 149.251 148.440 144.062 138.015 137.417 133.488 129.602 128.459 126.621 122.580 80.094 79.779 53.635 52.673 52.434 50.490 34.188 32.546 28.384 28.371 18.242 -1.712 -2.126
f1 (ppm)

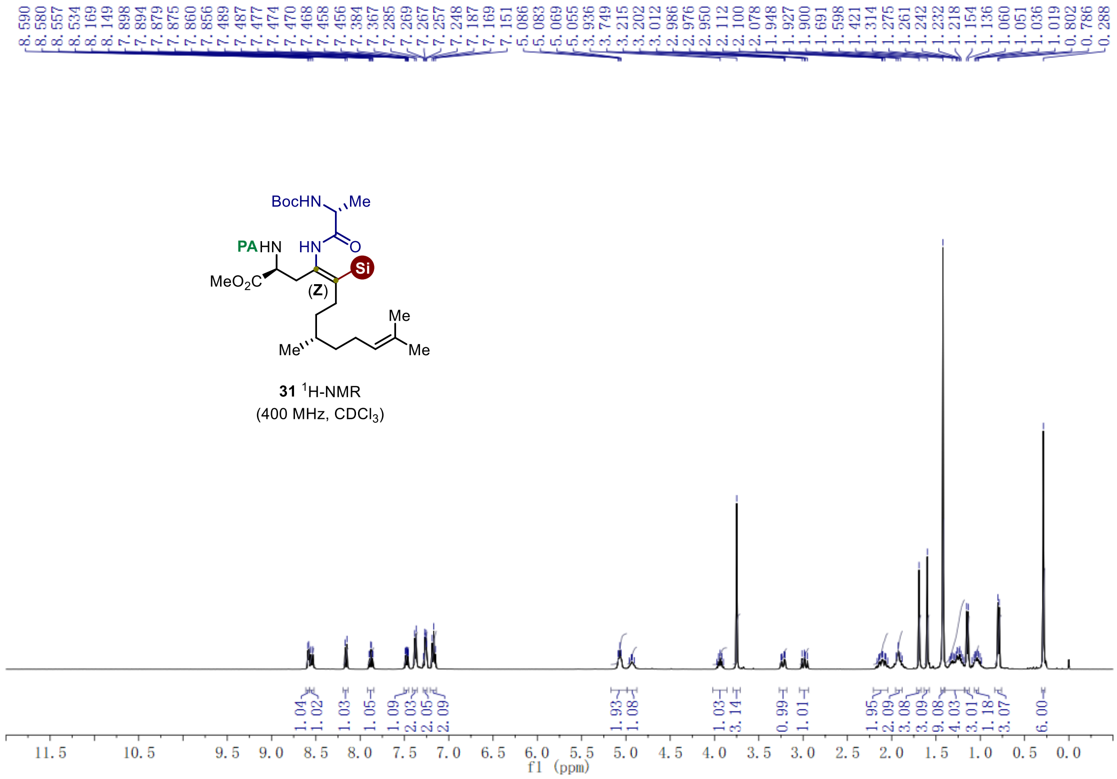
8.590
8.580
8.557
8.534
8.169
8.149
7.898
7.894
7.879
7.875
7.860
7.856
7.489
7.487
7.477
7.474
7.470
7.468
7.458
7.456
7.384
7.367
7.285
7.269
7.267
7.257
7.248
7.187
7.169
7.151
5.086
5.083
5.069
5.055
3.936
3.749
3.215
3.202
3.012
2.986
2.976
2.950
2.112
2.100
2.078
1.948
1.927
1.900
1.691
1.598
1.421
1.314
1.275
1.261
1.242
1.232
1.218
1.154
1.136
1.060
1.051
1.036
1.019
0.802
0.786
0.288
BocHN
Me
PAHN
HN
O
Si
MeO2C
(Z)
Me
Me
Me
31 1H-NMR
(400 MHz, CDCl3)
1.04
1.02
1.03
1.05
1.09
2.03
2.05
2.09
1.93
1.08
1.03
3.14
0.99
1.01
1.95
2.09
3.08
3.09
9.08
4.03
3.01
1.18
3.07
6.00
f1 (ppm)

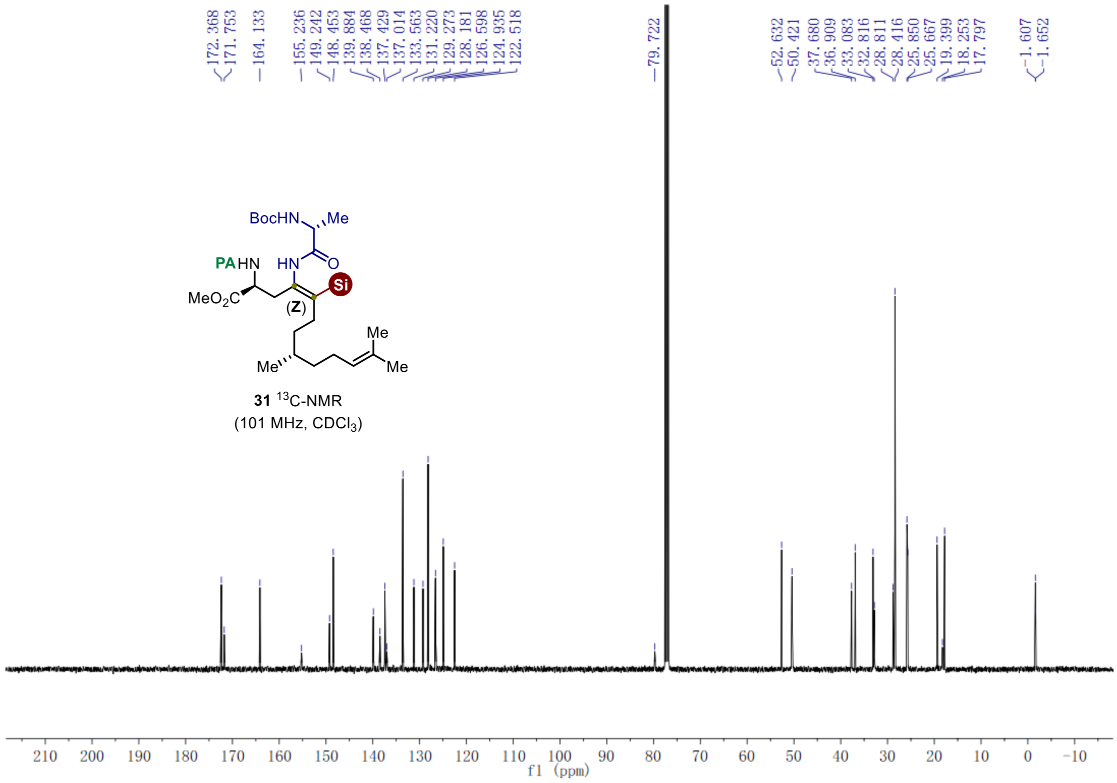
172.368
171.753
164.133
155.236
149.242
148.453
139.884
138.468
137.429
137.014
133.563
131.220
129.273
128.181
126.598
124.935
122.518
79.722
52.632
50.421
37.680
36.909
33.083
32.816
28.811
28.416
25.850
25.667
19.399
18.253
17.797
1.607
-1.652
BocHN
Me
PAHN
HN
O
Si
MeO2C
(Z)
Me
Me
Me
31 13C-NMR
(101 MHz, CDCl3)
f1 (ppm)

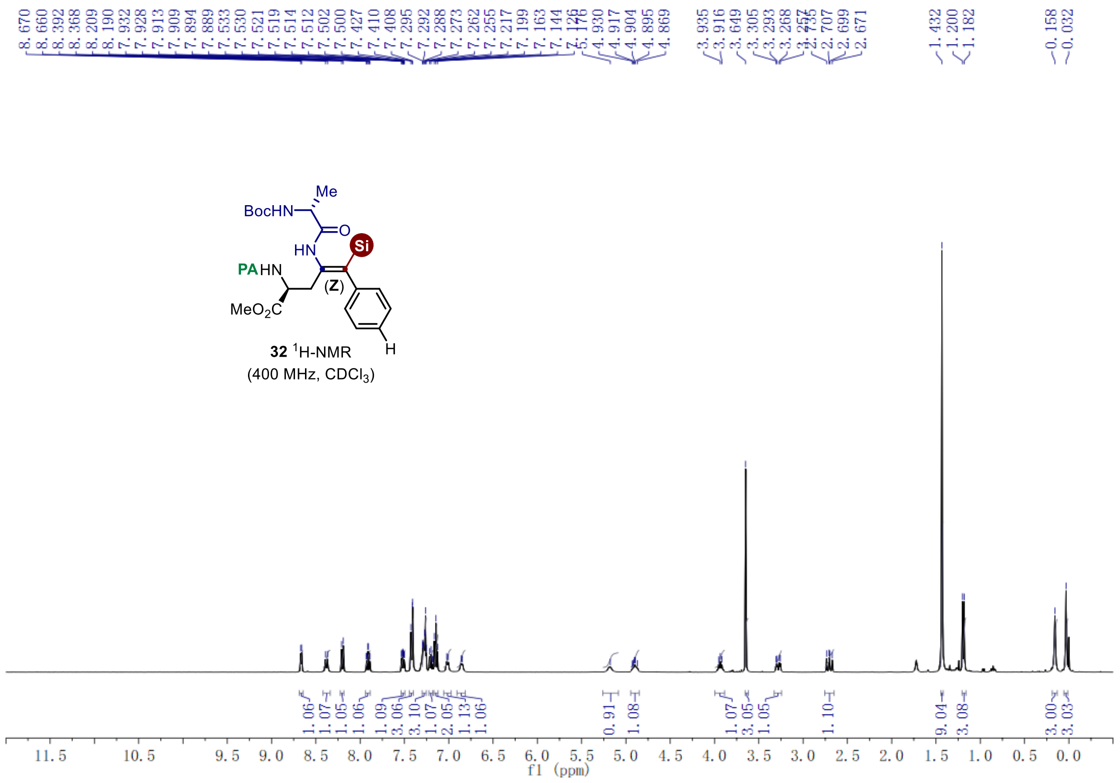
8.670
8.660
8.392
8.368
8.209
8.190
7.932
7.928
7.913
7.909
7.894
7.889
7.533
7.530
7.521
7.519
7.514
7.512
7.502
7.500
7.427
7.410
7.408
7.295
7.292
7.288
7.273
7.262
7.255
7.217
7.199
7.163
7.144
4.930
4.917
4.904
4.895
4.869
3.935
3.916
3.649
3.305
3.293
3.268
2.707
2.699
2.671
1.432
1.200
1.182
0.158
0.032
Me
BocHN
O
HN
Si
PA HN
(Z)
MeO2C
H
32 1H-NMR
(400 MHz, CDCl3)
1.06
1.07
1.05
1.06
1.09
3.06
3.10
1.07
2.05
1.13
1.06
0.91
1.08
1.07
3.05
1.05
1.10
9.04
3.08
3.00
3.03
f1 (ppm)

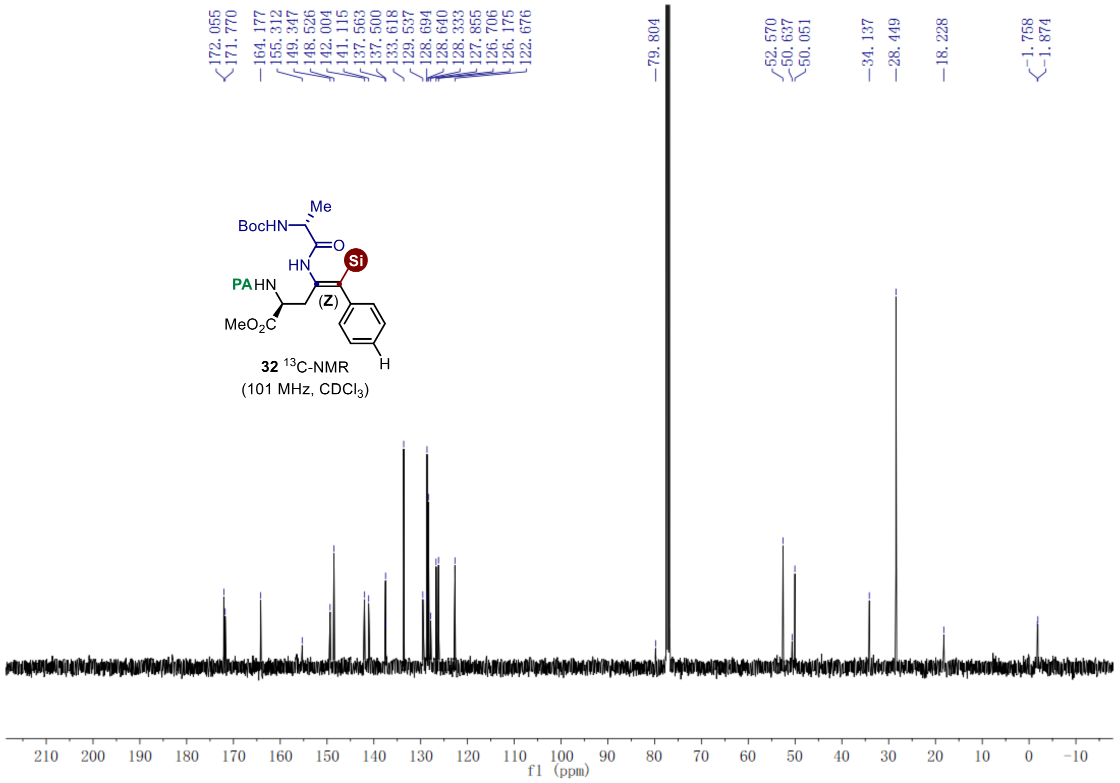
172.055
171.770
164.177
155.312
149.347
148.526
142.004
141.115
137.563
137.500
133.618
129.537
128.694
128.640
128.333
127.855
126.706
126.175
122.676
79.804
52.570
50.637
50.051
34.137
28.449
18.228
-1.758
-1.874
Me
BocHN
O
HN
Si
PA HN
(Z)
MeO2C
H
32 13C-NMR
(101 MHz, CDCl3)
f1 (ppm)

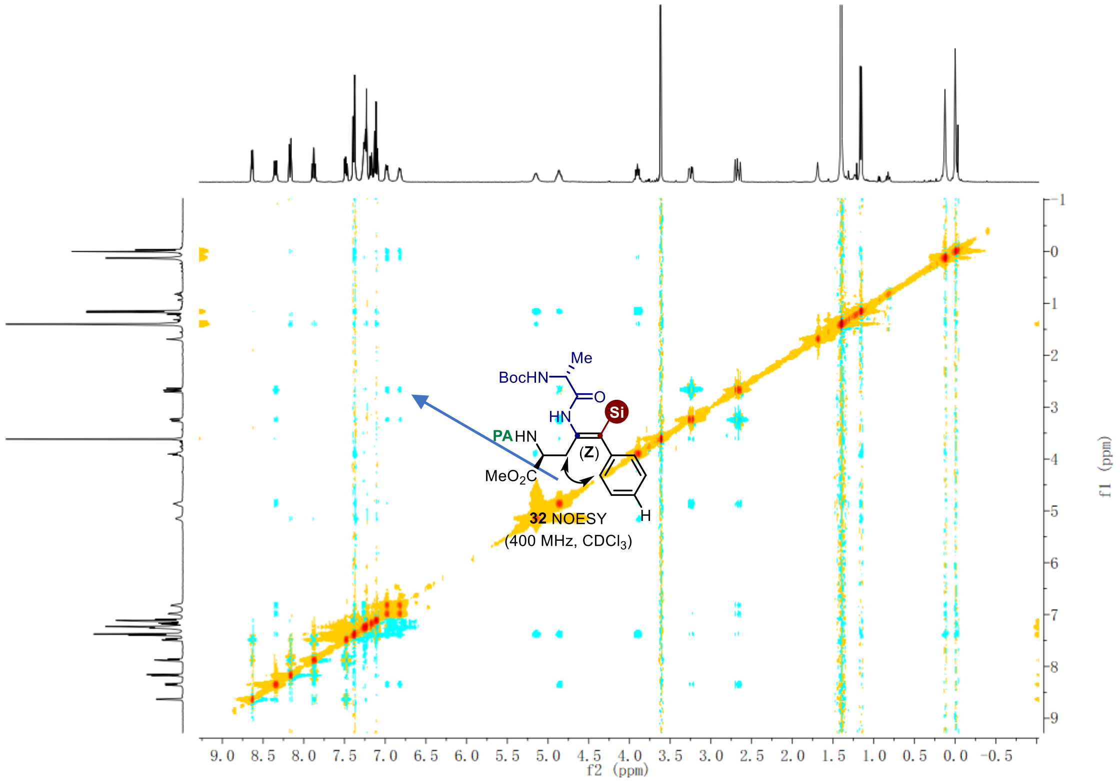
BocHN
Me
O
HN
Si
PA HN
(Z)
MeO2C
H
32 NOESY
(400 MHz, CDCl3)
f1 (ppm)
f2 (ppm)

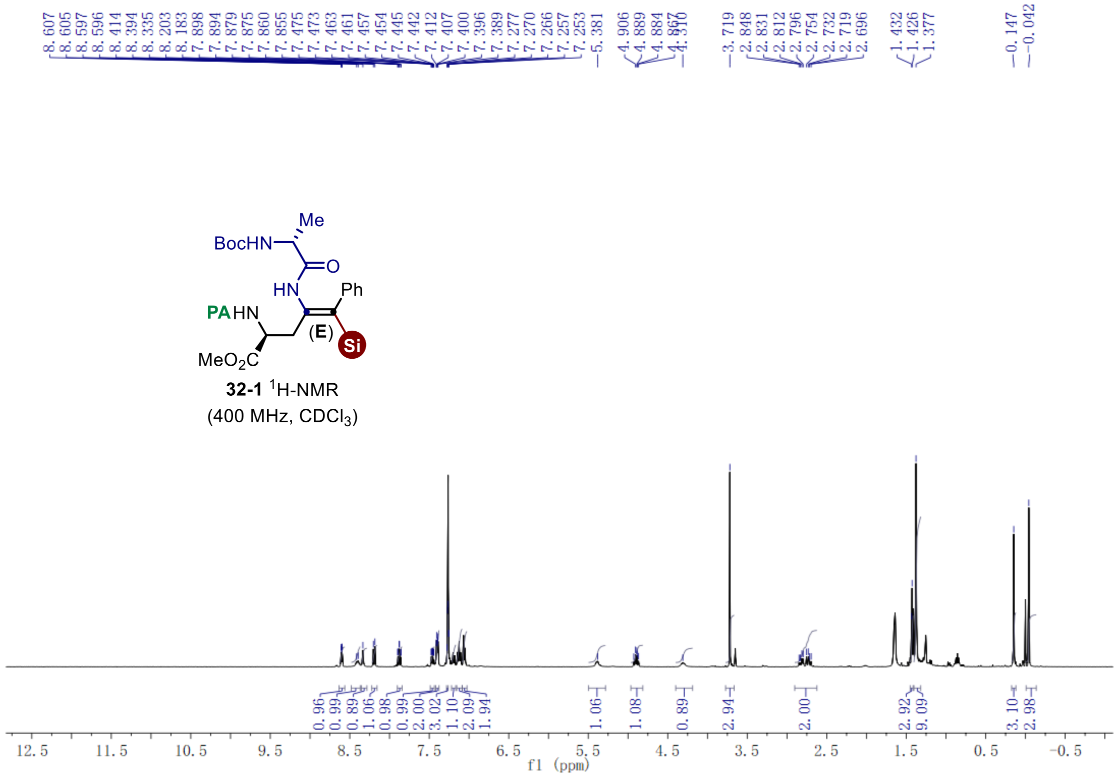
BocHN
Me
O
HN
Ph
PA HN
(E)
Si
MeO2C
32-1 1H-NMR
(400 MHz, CDCl3)
f1 (ppm)

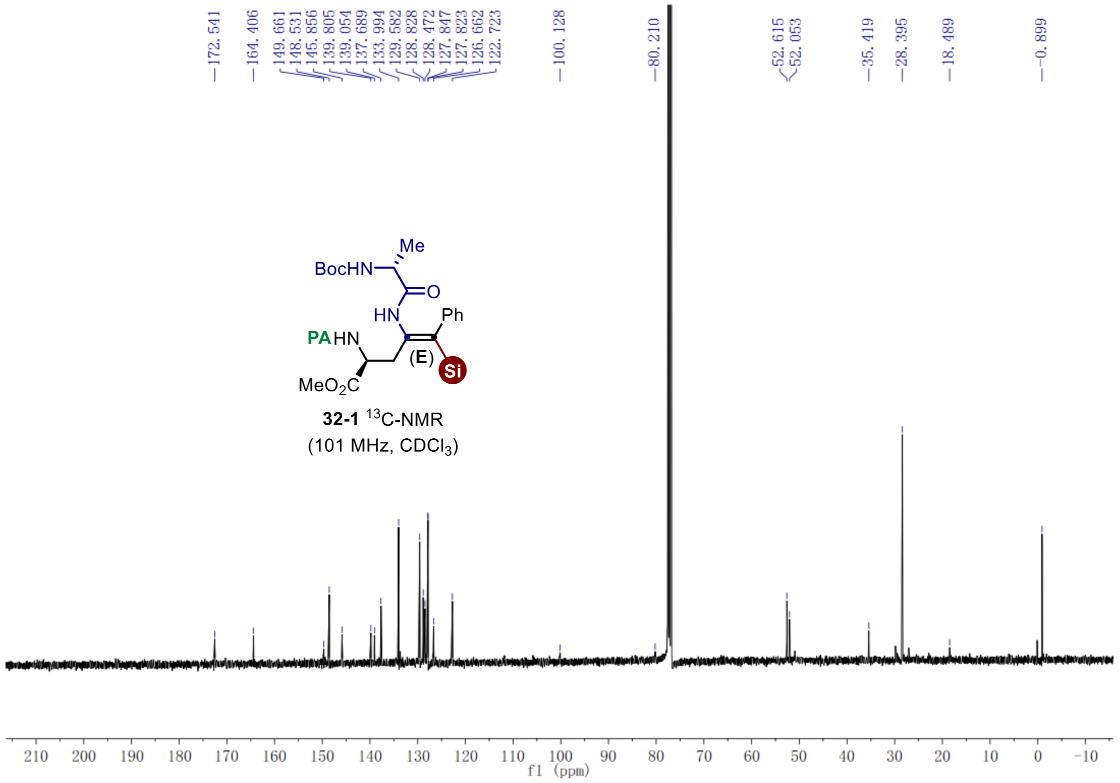
BocHN
Me
O
HN
Ph
PA HN
(E)
Si
MeO2C
32-1 13C-NMR
(101 MHz, CDCl3)
f1 (ppm)

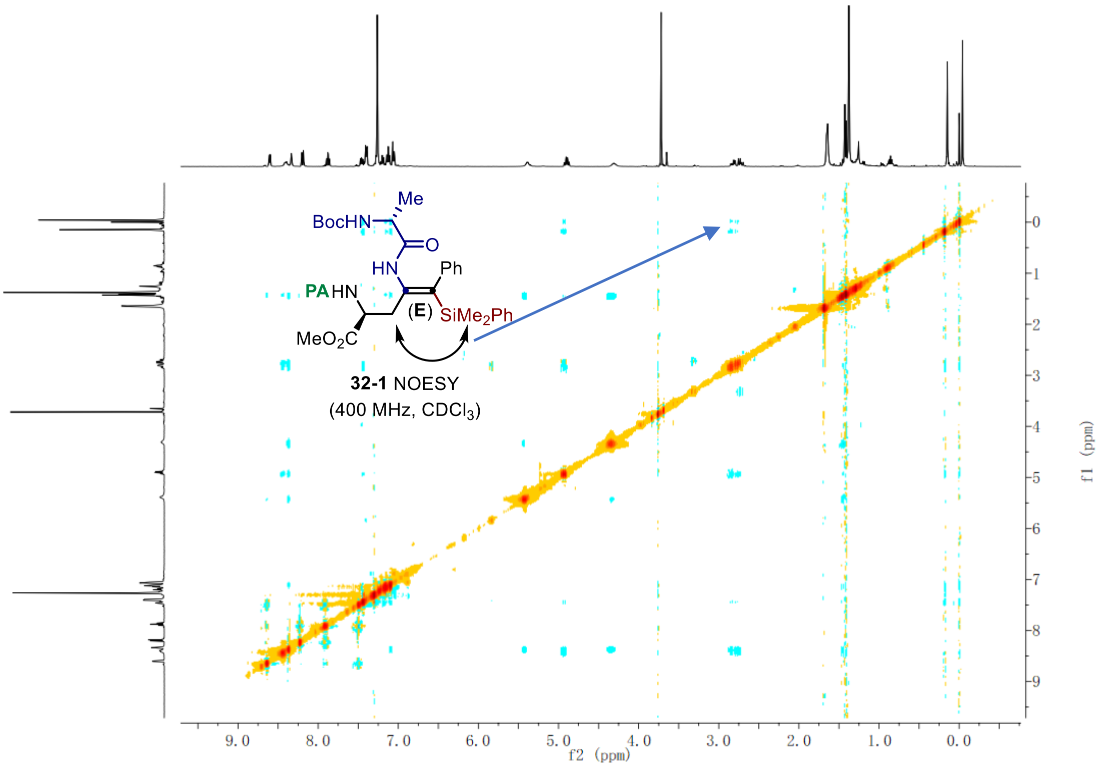
Me
BocHN
O
HN
Ph
PA HN
(E)
SiMe2Ph
MeO2C
32-1 NOESY
(400 MHz, CDCl3)
f2 (ppm)
f1 (ppm)

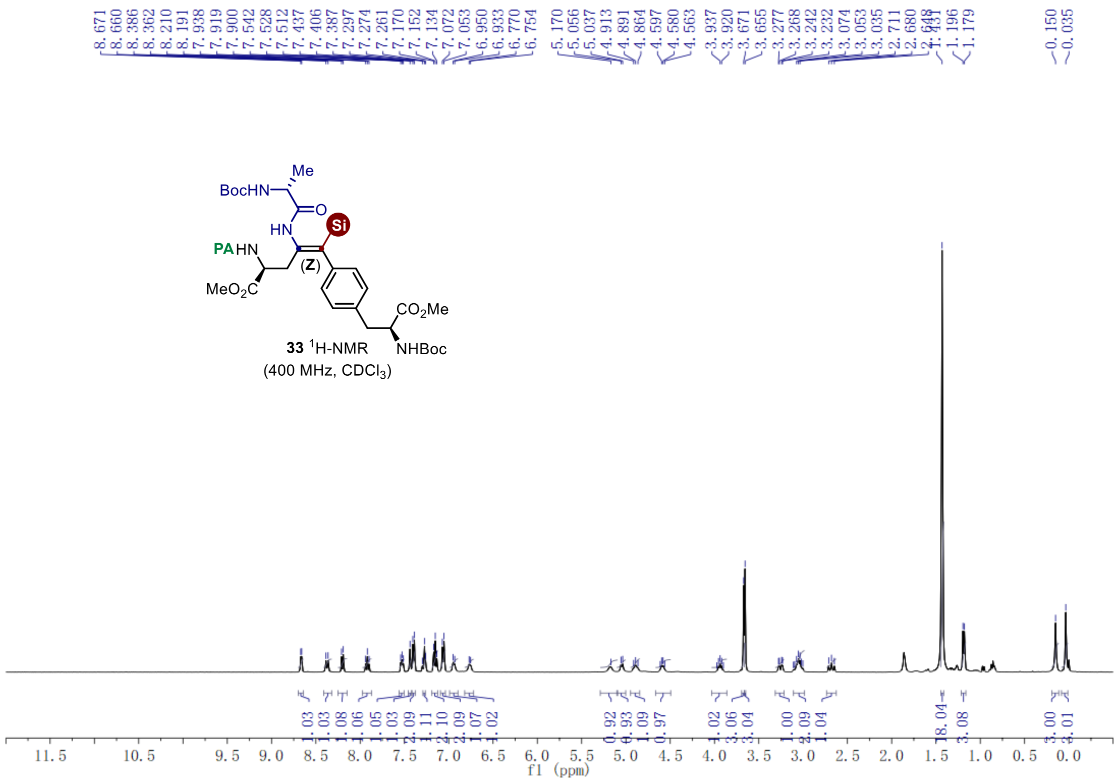
BocHN
Me
O
HN
Si
PAHN
(Z)
MeO2C
CO2Me
33 1H-NMR
NHBoc
(400 MHz, CDCl3)
f1 (ppm)

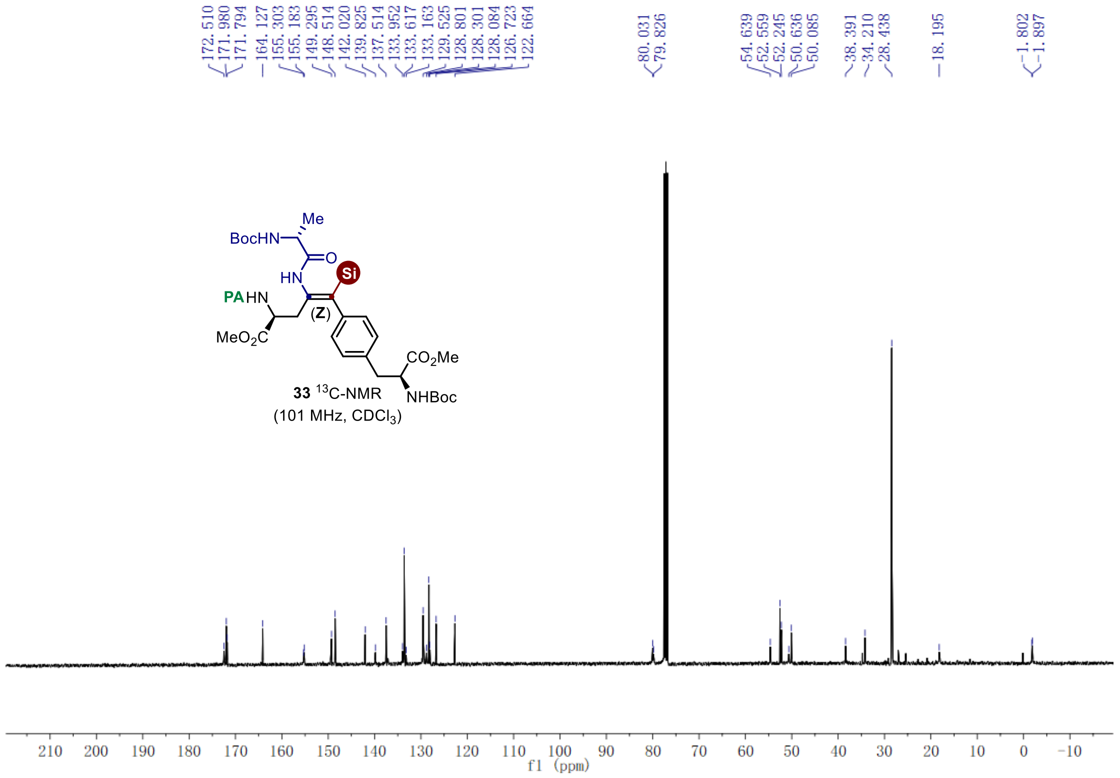
BocHN
Me
O
HN
Si
PAHN
(Z)
MeO2C
CO2Me
33 13C-NMR
NHBoc
(101 MHz, CDCl3)
f1 (ppm)

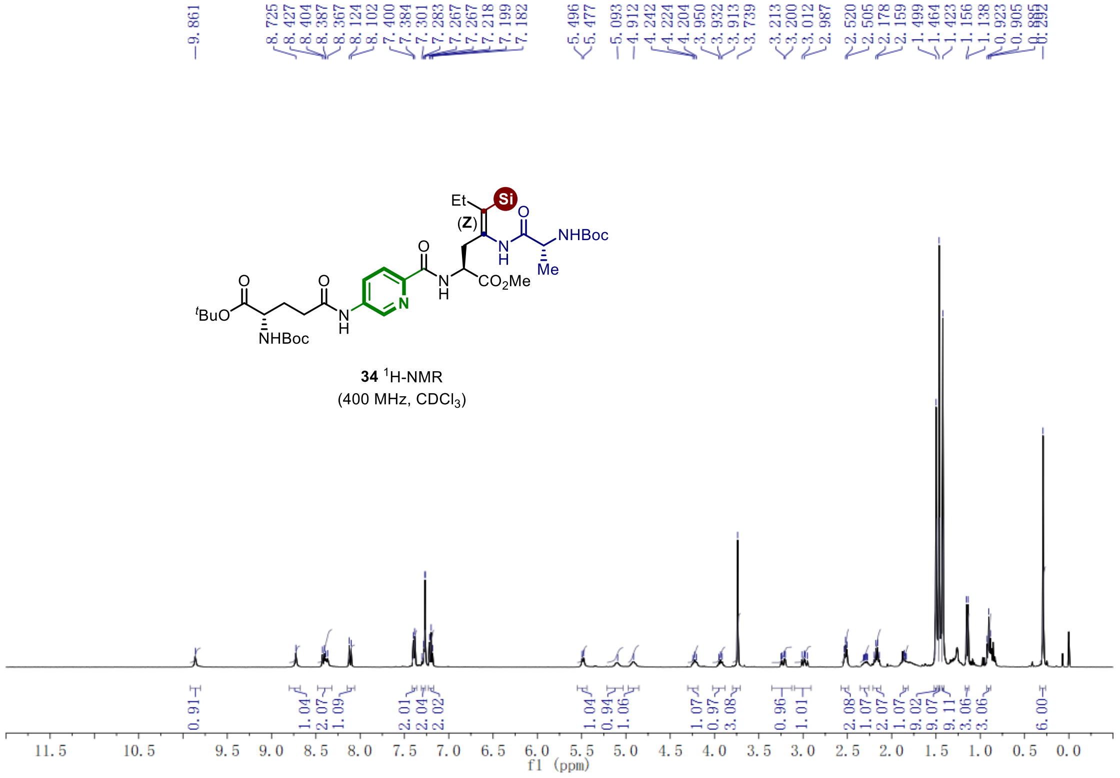
Et
Si
(Z)
O
NHBoc
N
H
Me
O
N
H
CO2Me
O
O
tBuO
NHBoc
N
H
N
34 1H-NMR
(400 MHz, CDCl3)
f1 (ppm)

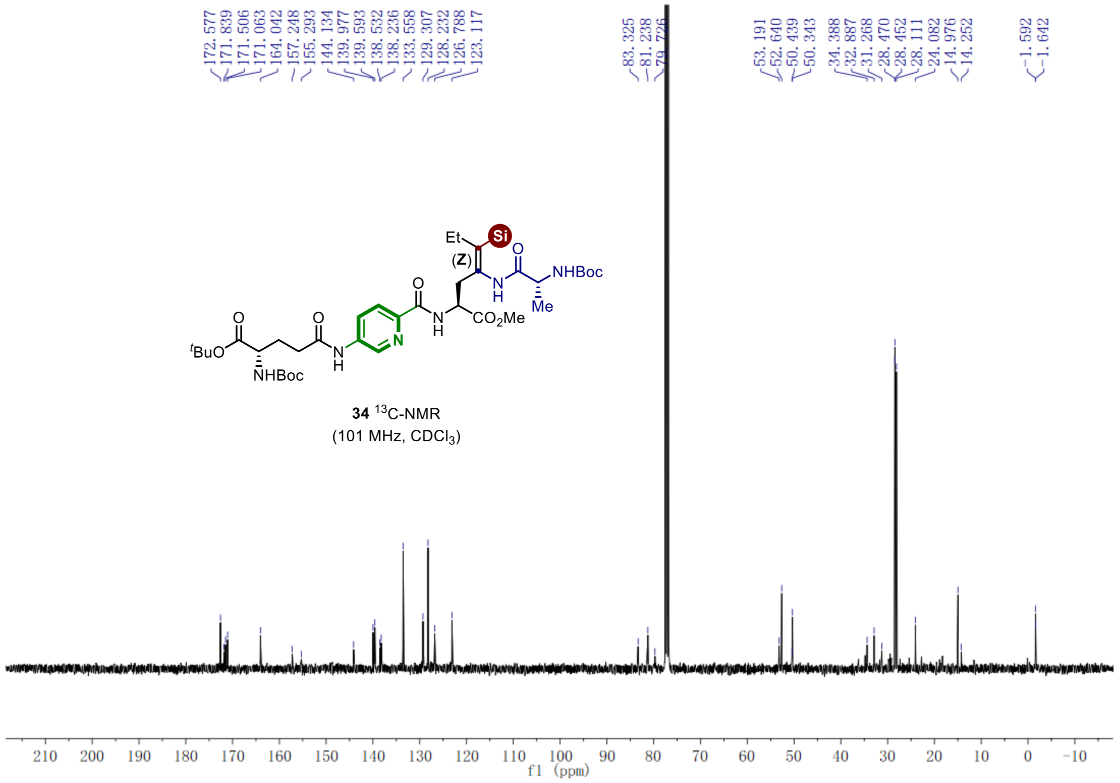
Et
Si
(Z)
O
NHBoc
N
H
Me
O
N
H
CO2Me
O
O
tBuO
NHBoc
N
H
N
34 13C-NMR
(101 MHz, CDCl3)
f1 (ppm)

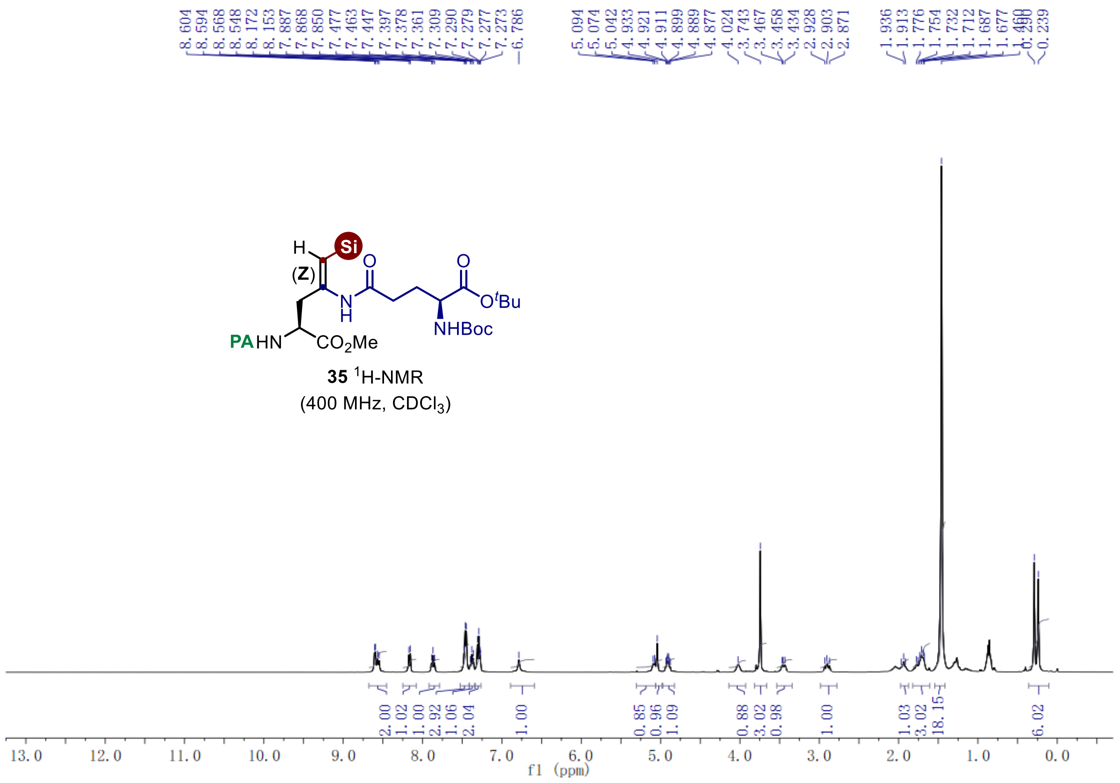
35 1H-NMR
(400 MHz, CDCl3)

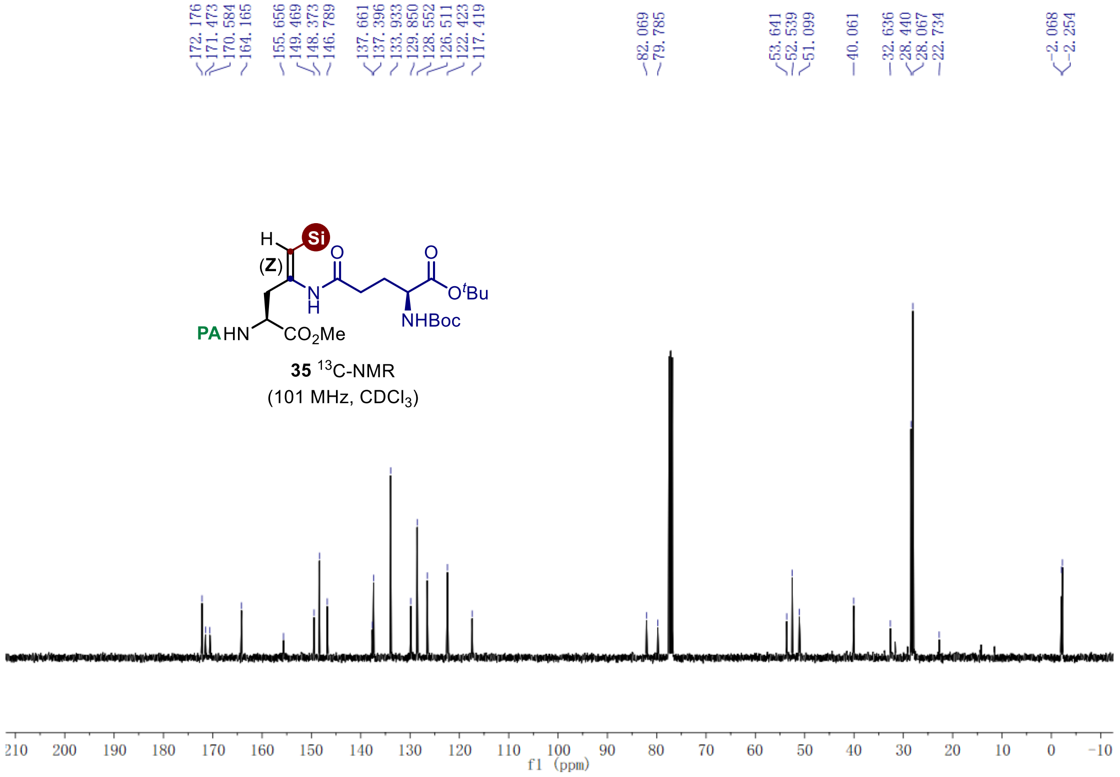
35 13C-NMR
(101 MHz, CDCl3)

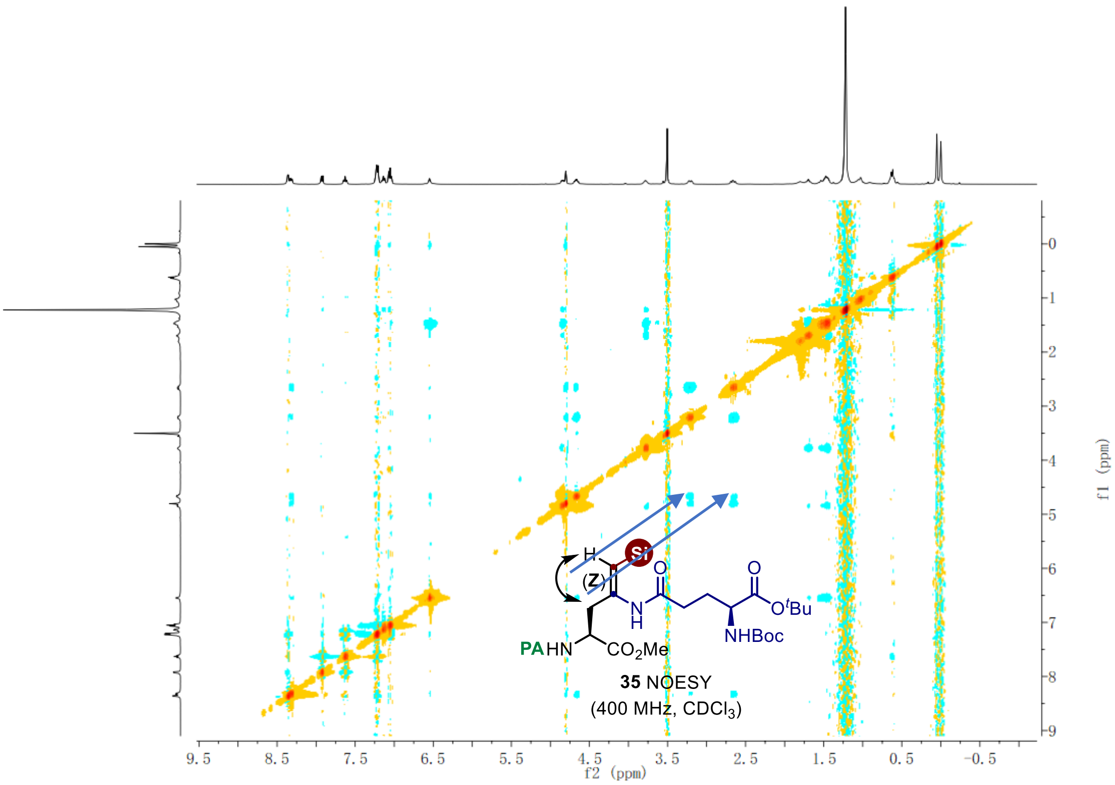

H
Si
(Z)
N
H
O
O
O^{t}Bu
PA HN
CO_2Me
NHBoc
35 NOESY
(400 MHz, $CDCl_3$)
f2 (ppm)
f1 (ppm)

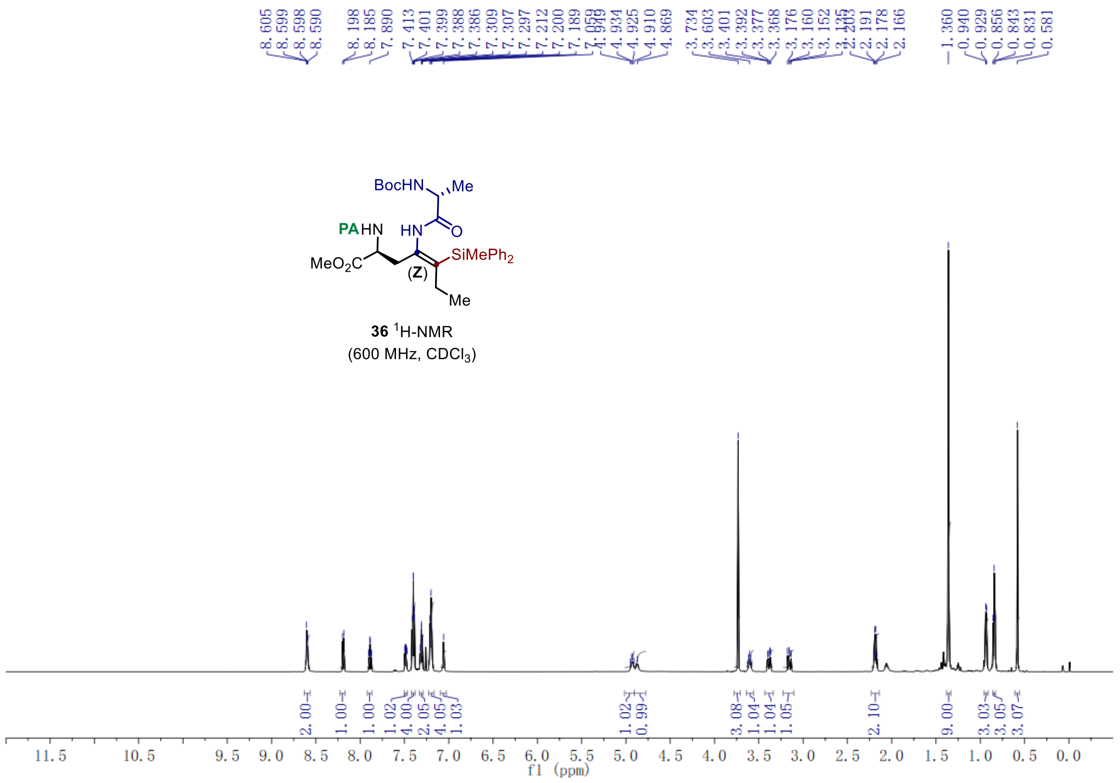
BocHN
Me
PA HN
HN
O
SiMePh2
MeO2C
(Z)
Me
36 1H-NMR
(600 MHz, CDCl3)
f1 (ppm)

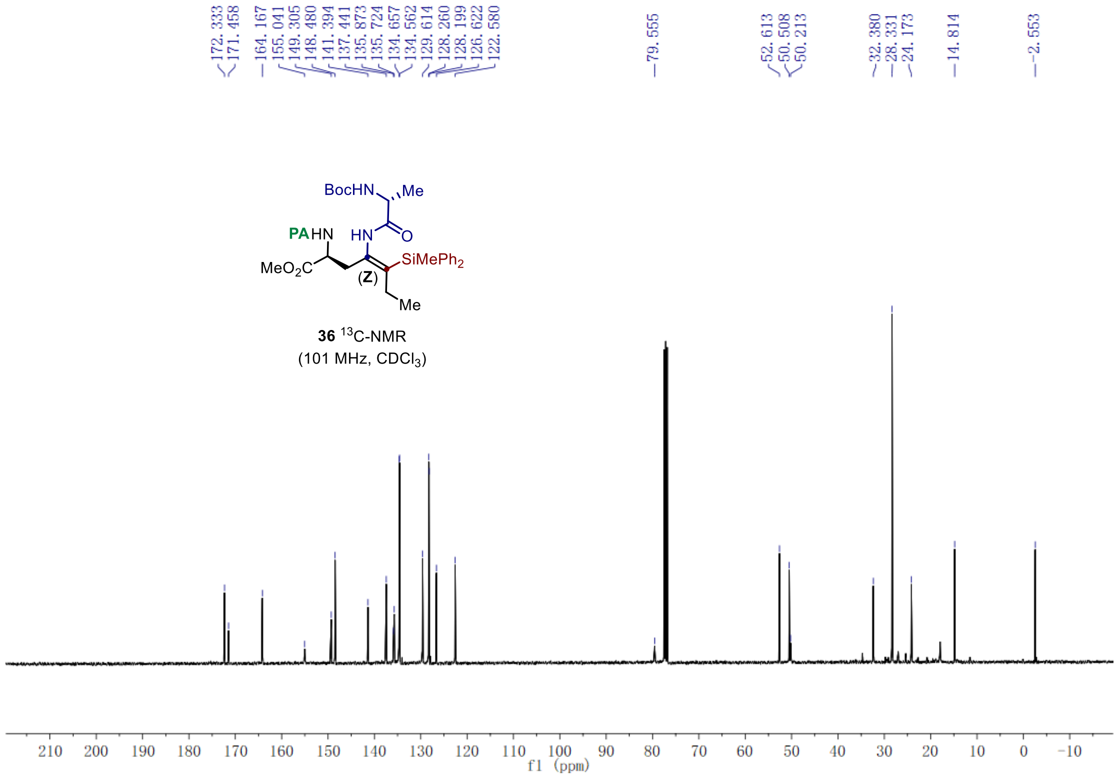
BocHN
Me
PA HN
HN
O
SiMePh2
MeO2C
(Z)
Me
36 13C-NMR
(101 MHz, CDCl3)
f1 (ppm)

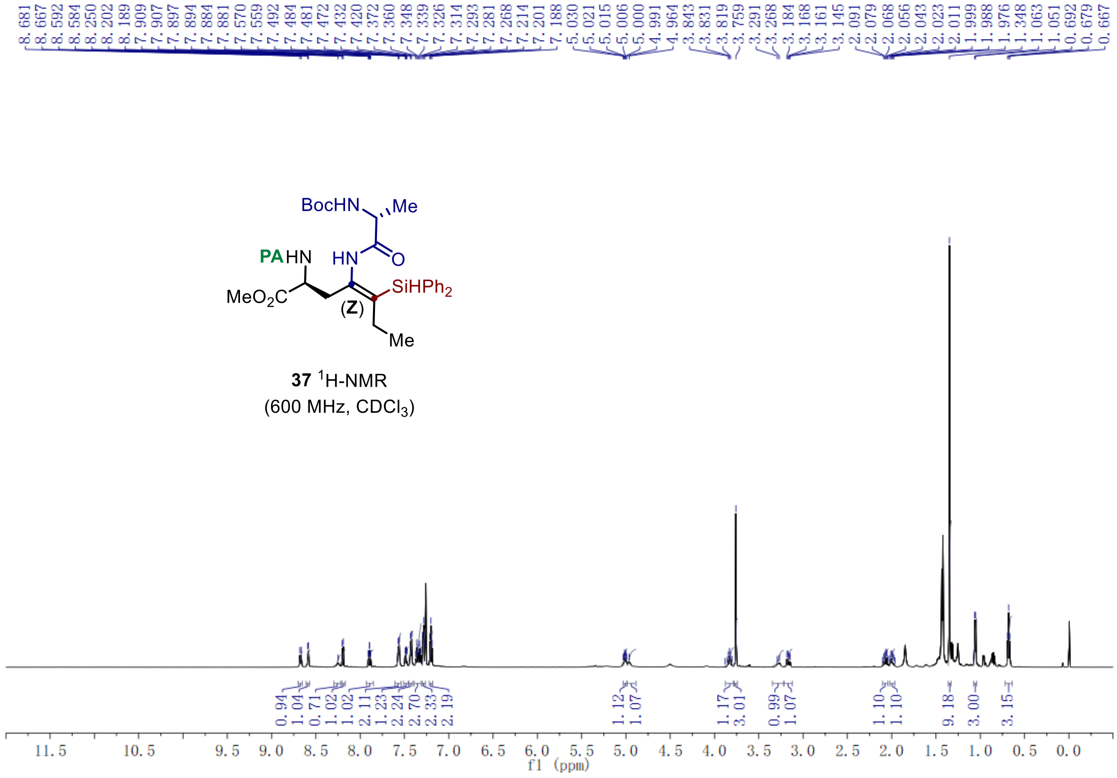

37 ^{1}H-NMR
(600 MHz, $CDCl_3$)

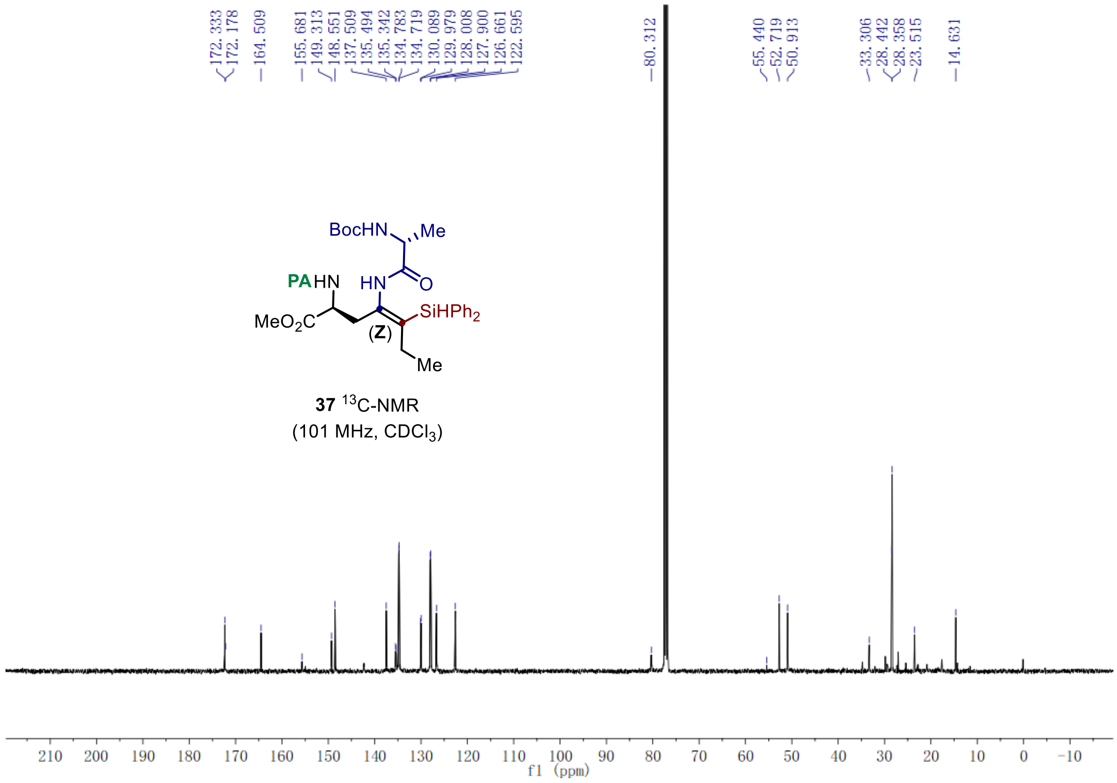

37 ^{13}C-NMR
(101 MHz, $CDCl_3$)

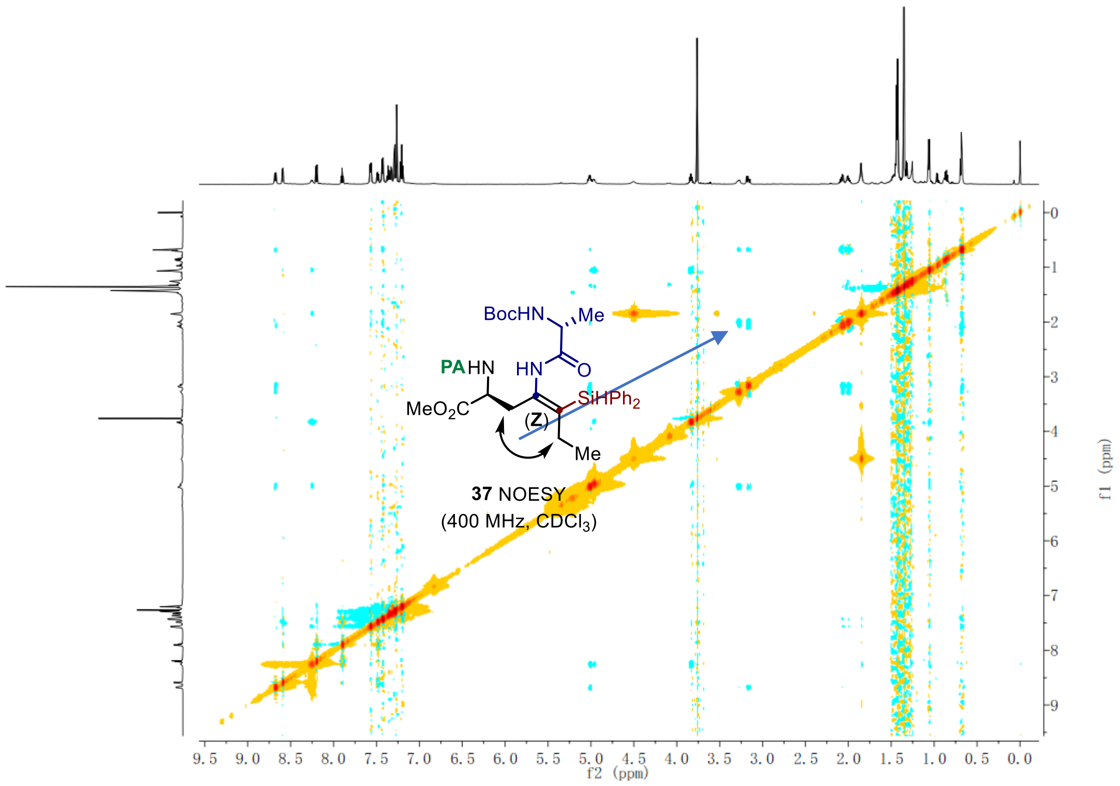

BocHN
Me
PA HN
HN
O
MeO2C
SiHPh2
(Z)
Me
37 NOESY
(400 MHz, CDCl3)
f2 (ppm)
f1 (ppm)

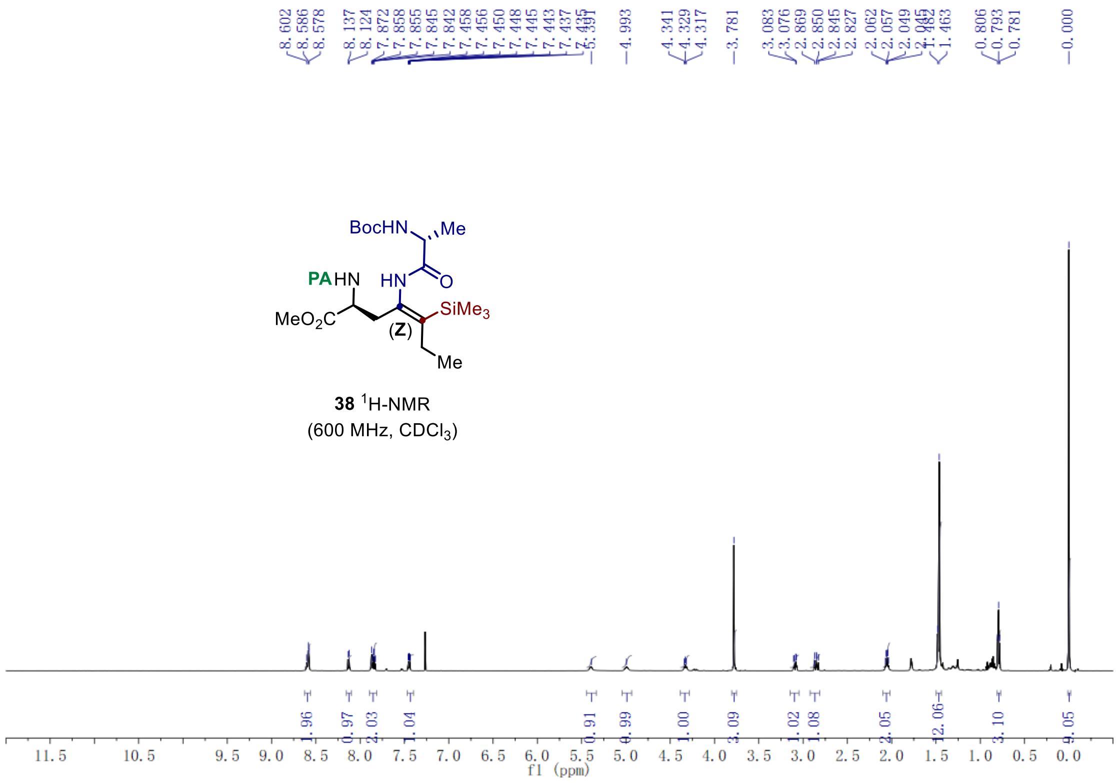
8.602
8.586
8.578
8.137
8.124
7.872
7.858
7.855
7.845
7.842
7.458
7.456
7.450
7.448
7.445
7.443
7.437
4.993
4.341
4.329
4.317
3.781
3.083
3.076
2.869
2.850
2.845
2.827
2.062
2.057
2.049
1.463
0.806
0.793
0.781
0.000
BocHN
Me
PA HN
HN
O
MeO2C
SiMe3
(Z)
Me
38 1H-NMR
(600 MHz, CDCl3)
1.96
0.97
2.03
1.04
0.91
0.99
1.00
3.09
1.02
1.08
2.05
12.06
3.10
9.05
11.5
10.5
9.5
9.0
8.5
8.0
7.5
7.0
6.5
6.0
5.5
5.0
4.5
4.0
3.5
3.0
2.5
2.0
1.5
1.0
0.5
0.0
f1 (ppm)

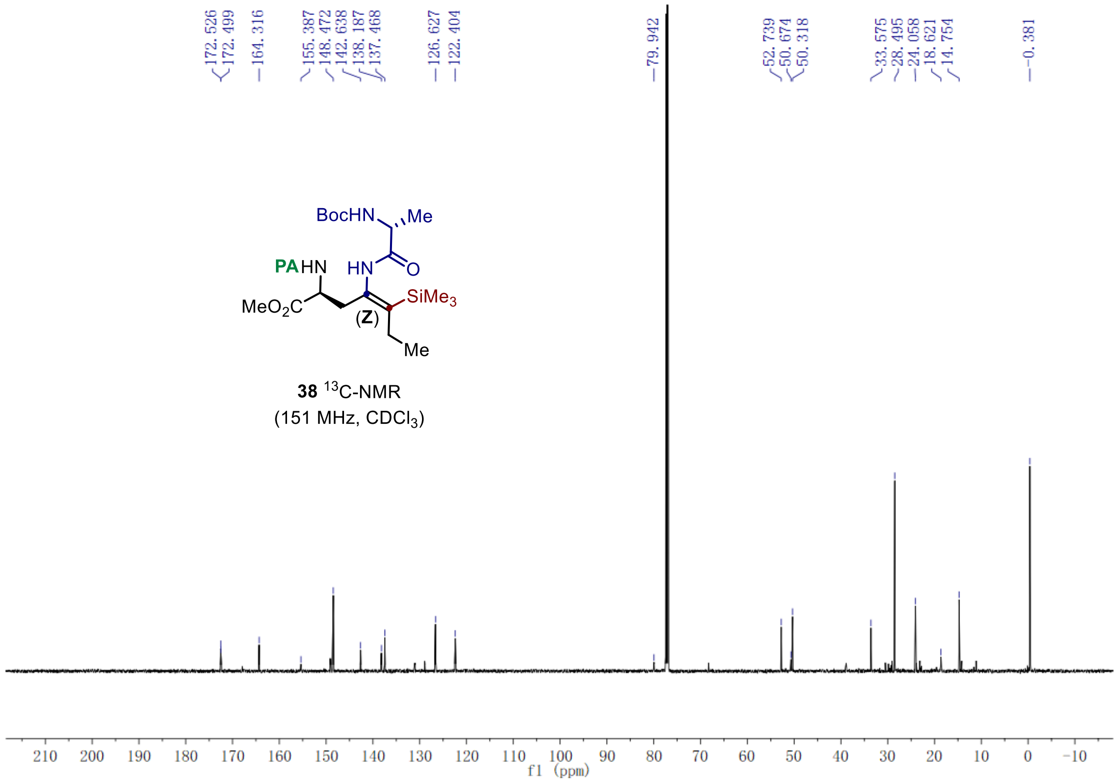
172.526
172.499
164.316
155.387
148.472
142.638
138.187
137.468
126.627
122.404
79.942
52.739
50.674
50.318
33.575
28.495
24.058
18.621
14.754
-0.381
BocHN
Me
PA HN
HN
O
MeO2C
SiMe3
(Z)
Me
38 13C-NMR
(151 MHz, CDCl3)
210
200
190
180
170
160
150
140
130
120
110
100
90
80
70
60
50
40
30
20
10
0
-10
f1 (ppm)

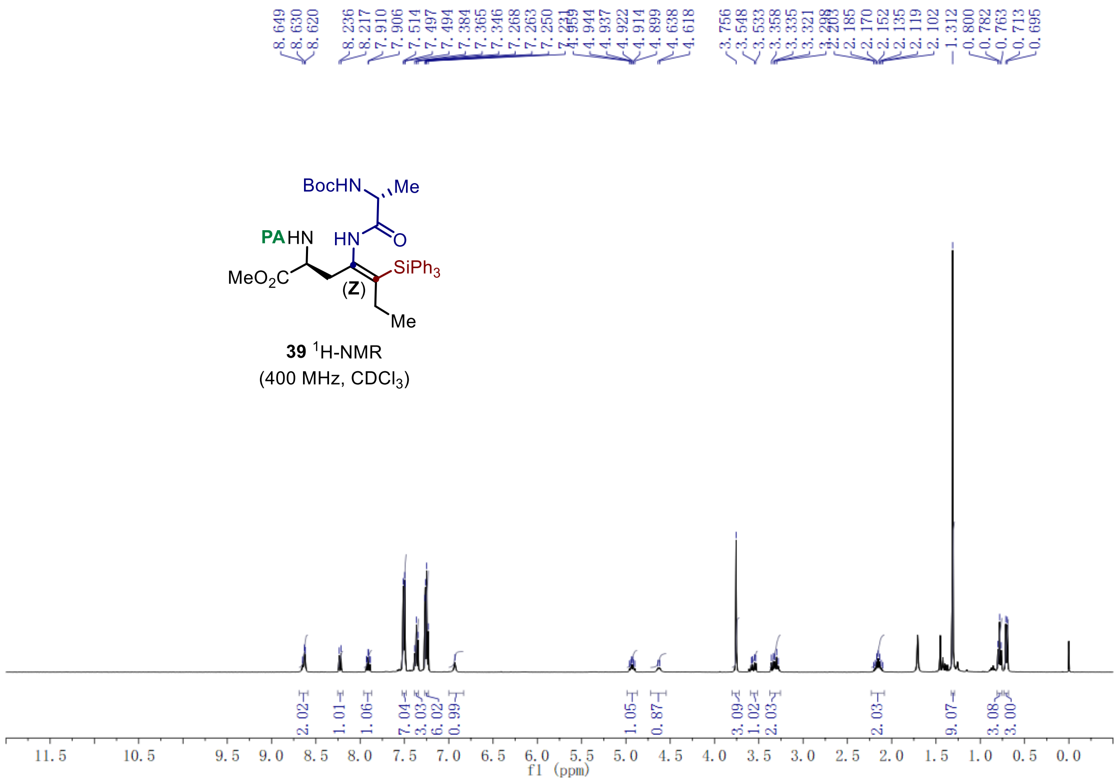

39 1H-NMR
(400 MHz, CDCl3)

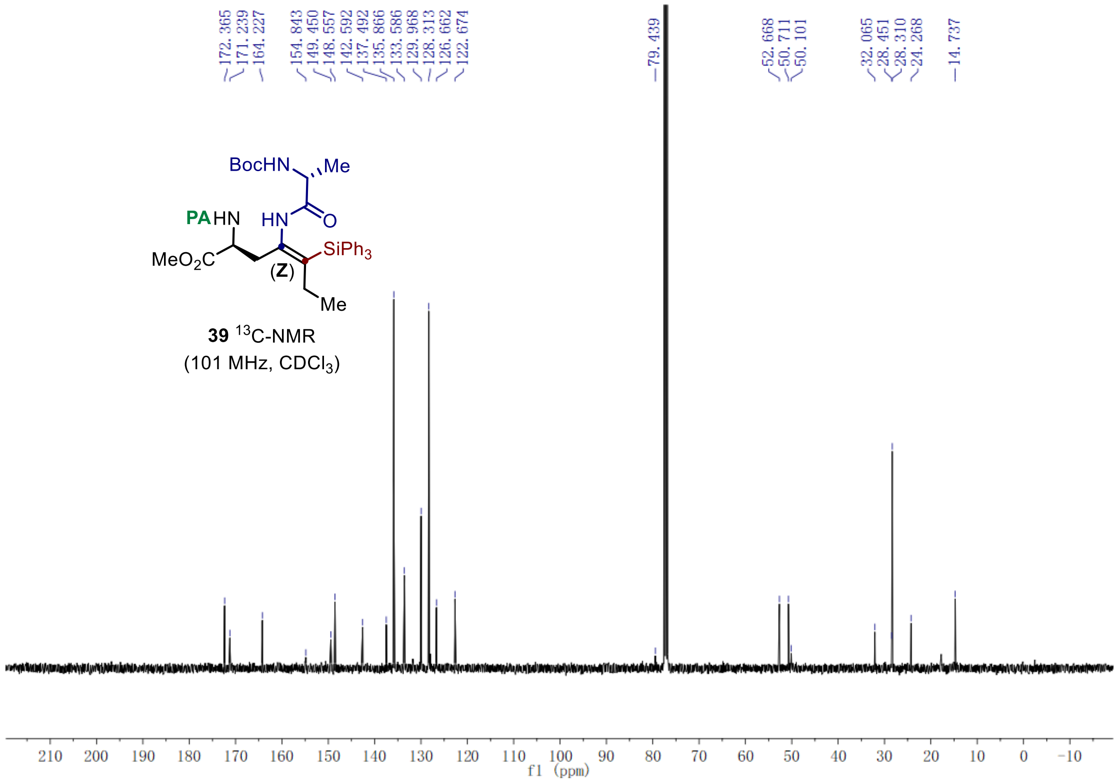

39 13C-NMR
(101 MHz, CDCl3)

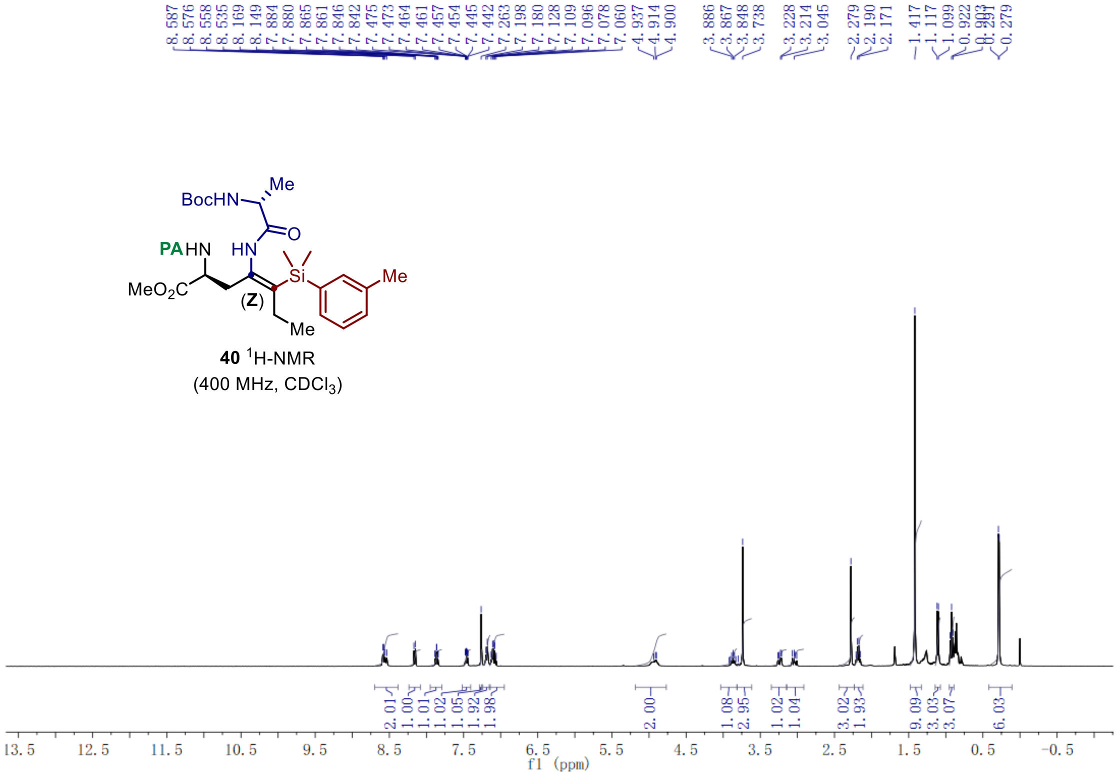
BocHN
Me
O
PAHN
HN
MeO2C
(Z)
Si
Me
Me
40 1H-NMR
(400 MHz, CDCl3)
f1 (ppm)

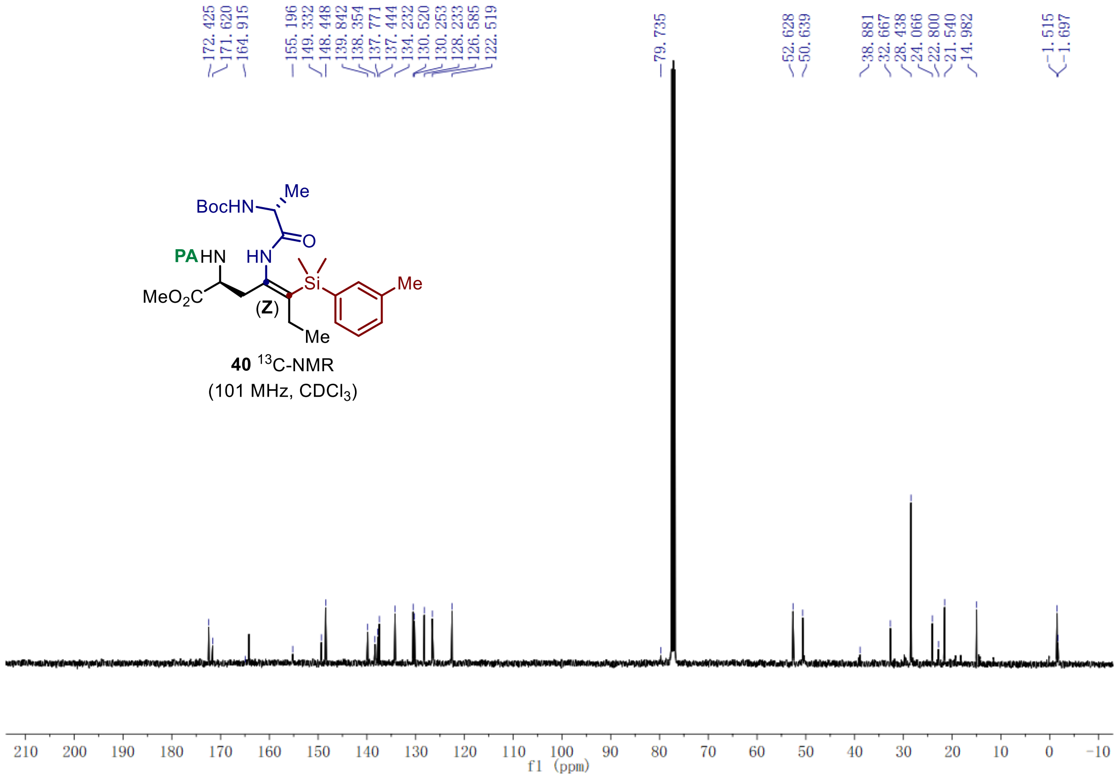
BocHN
Me
O
PAHN
HN
MeO2C
(Z)
Si
Me
Me
40 13C-NMR
(101 MHz, CDCl3)
f1 (ppm)

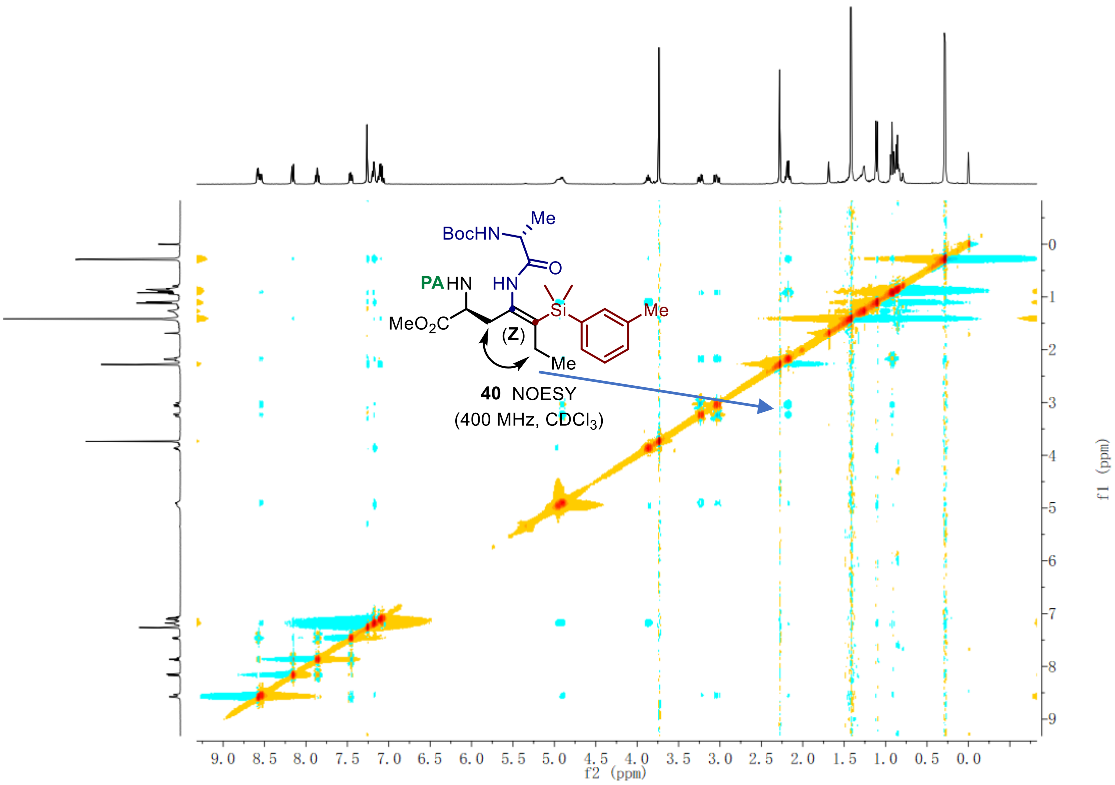

BocHN
Me
O
PA HN
HN
Si
Me
MeO2C
(Z)
Me
40 NOESY
(400 MHz, CDCl3)
f2 (ppm)
f1 (ppm)

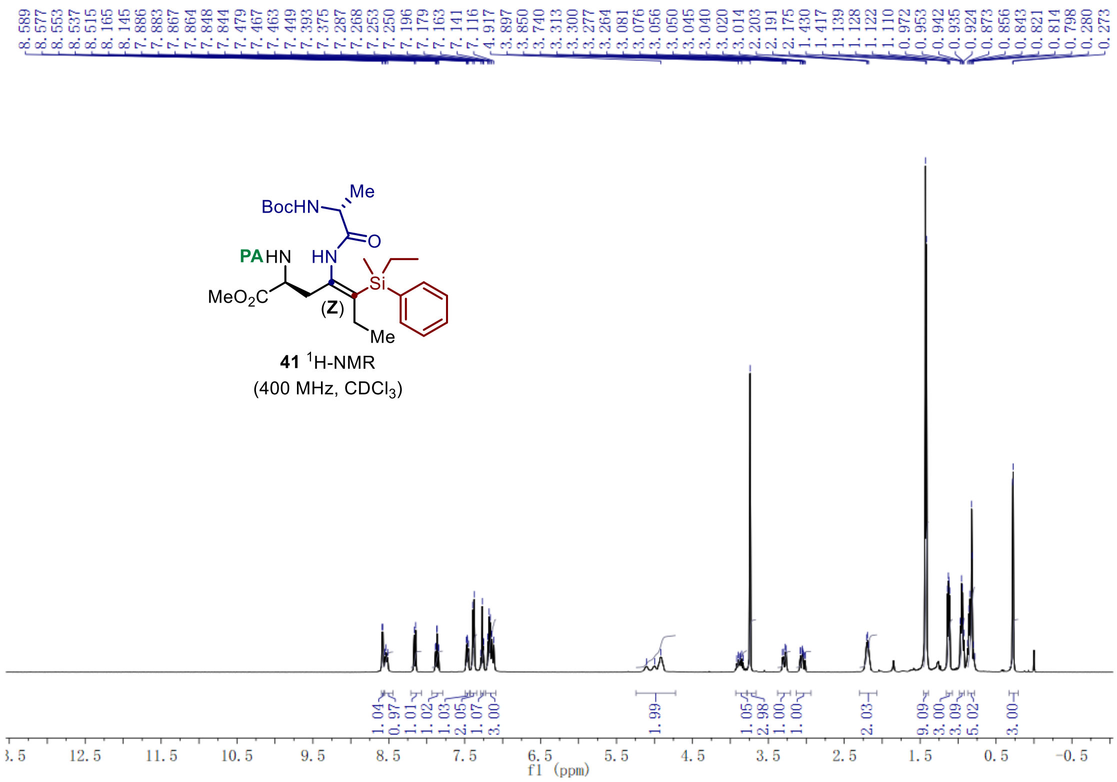

8.589
8.577
8.553
8.537
8.515
8.165
8.145
7.886
7.883
7.867
7.864
7.848
7.844
7.479
7.467
7.463
7.449
7.393
7.375
7.287
7.268
7.253
7.250
7.196
7.179
7.163
7.141
7.116
4.917
3.897
3.850
3.740
3.313
3.300
3.277
3.264
3.081
3.076
3.056
3.050
3.045
3.040
3.020
3.014
2.203
2.191
2.175
1.430
1.417
1.139
1.128
1.122
1.110
0.972
0.953
0.942
0.935
0.924
0.873
0.856
0.843
0.821
0.814
0.798
0.280
0.273
BocHN
Me
O
PA HN
HN
Si
MeO2C
(Z)
Me
41 1H-NMR
(400 MHz, CDCl3)
f1 (ppm)

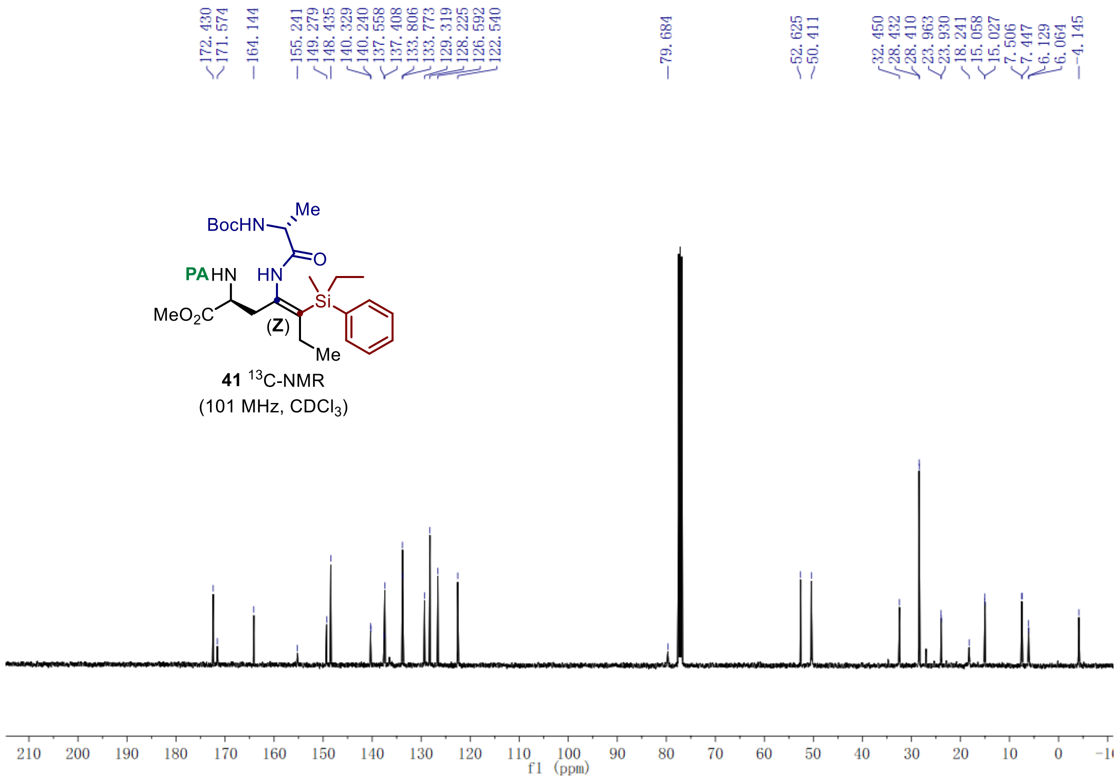

172.430
171.574
164.144
155.241
149.279
148.435
140.329
140.240
137.558
137.408
133.806
133.773
129.319
128.225
126.592
122.540
79.684
52.625
50.411
32.450
28.432
28.410
23.963
23.930
18.241
15.058
15.027
7.506
7.447
6.129
6.064
-4.145
BocHN
Me
O
PA HN
HN
Si
MeO2C
(Z)
Me
41 13C-NMR
(101 MHz, CDCl3)
f1 (ppm)

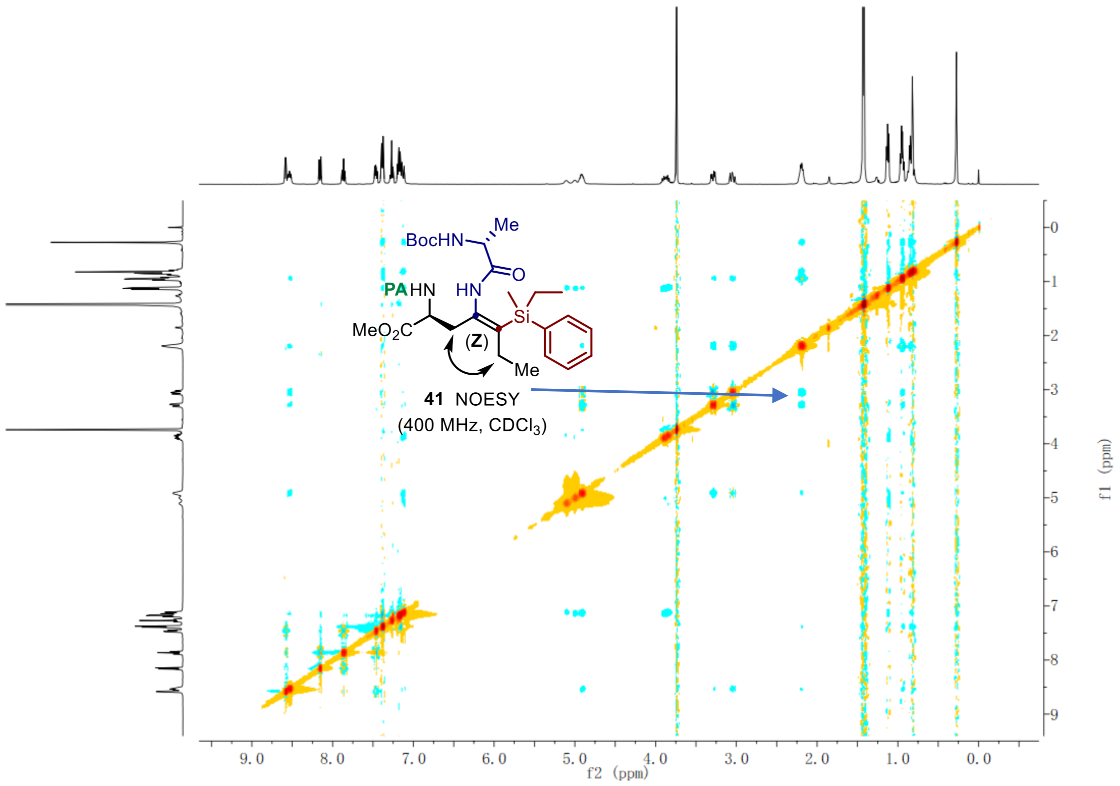
BocHN
Me
O
PA HN
HN
Si
MeO2C
(Z)
Me
41 NOESY
(400 MHz, CDCl3)
f2 (ppm)
f1 (ppm)

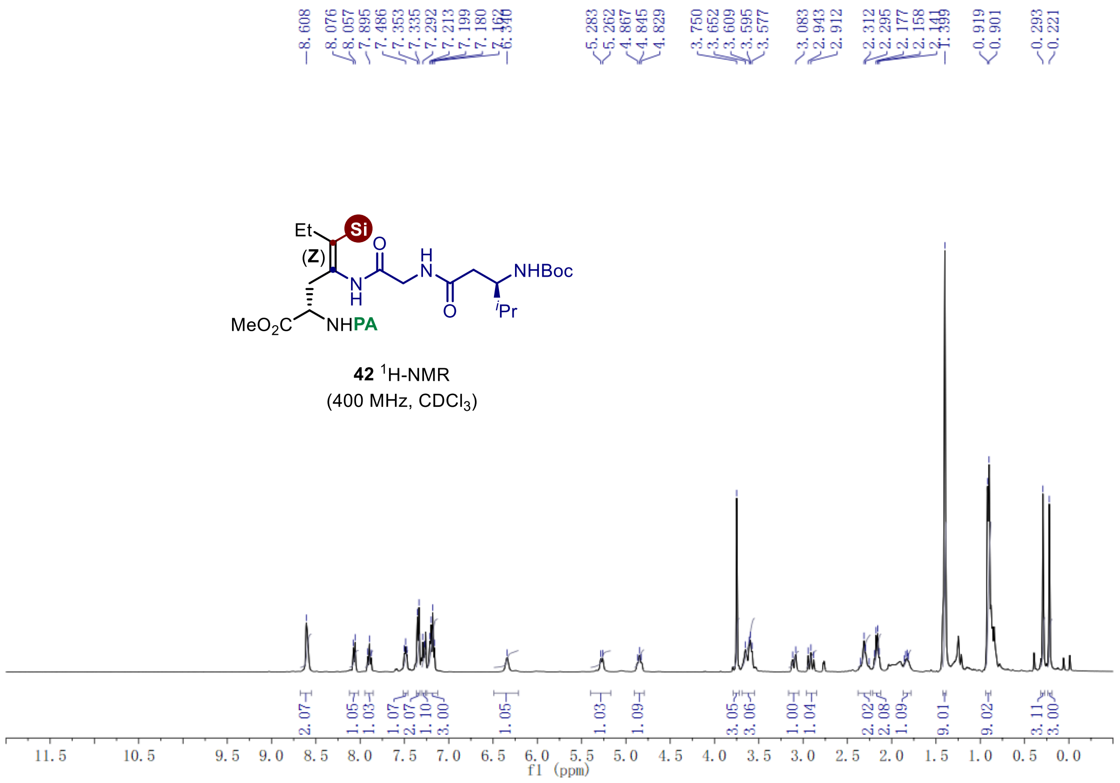

42 ^{1}H-NMR
(400 MHz, $CDCl_3$)

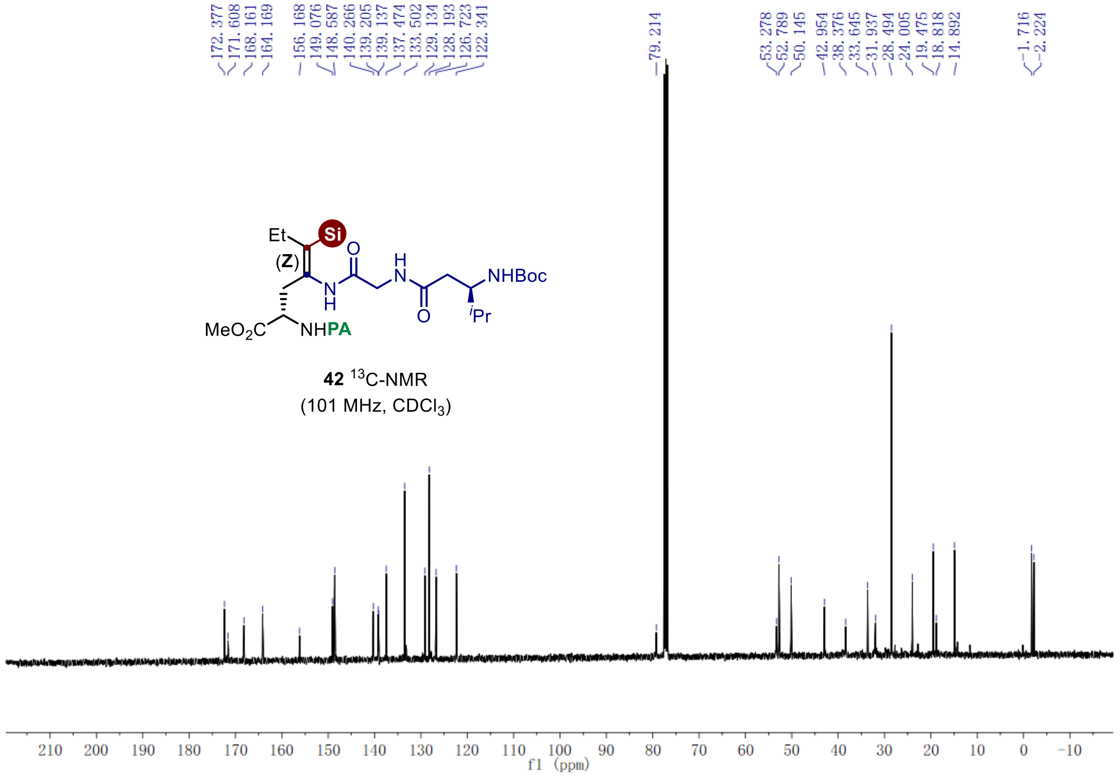

42 ^{13}C-NMR
(101 MHz, $CDCl_3$)

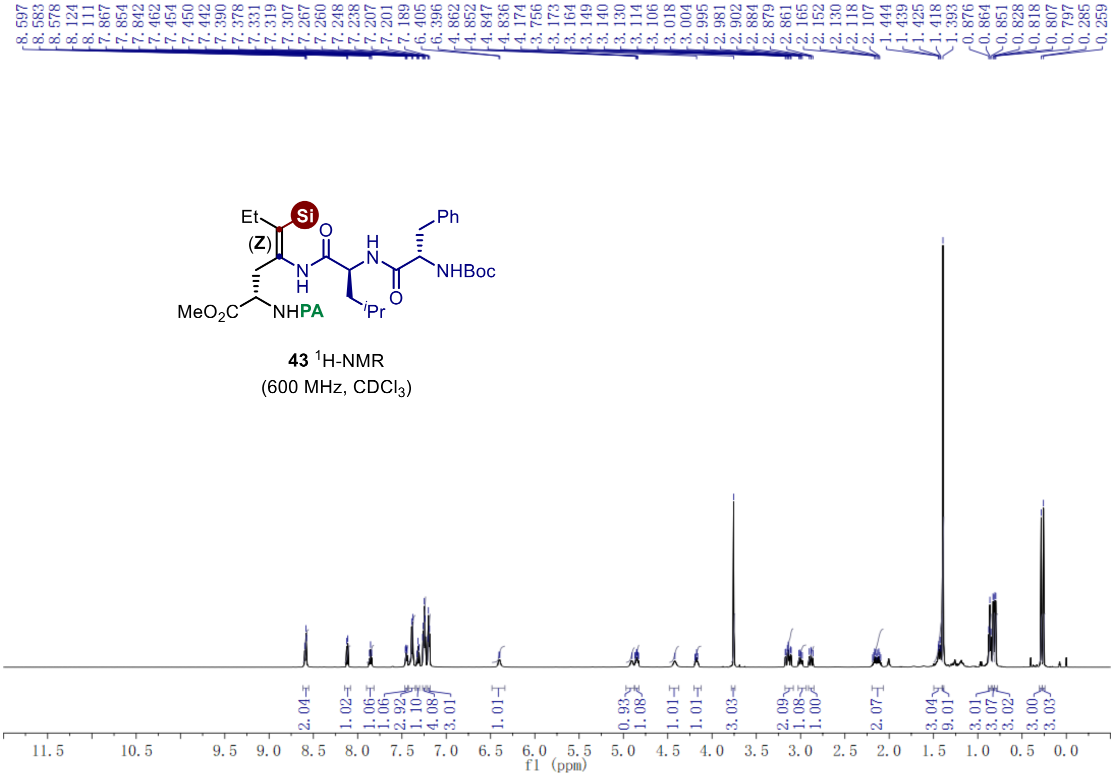

43 ^{1}H-NMR
(600 MHz, $CDCl_3$)

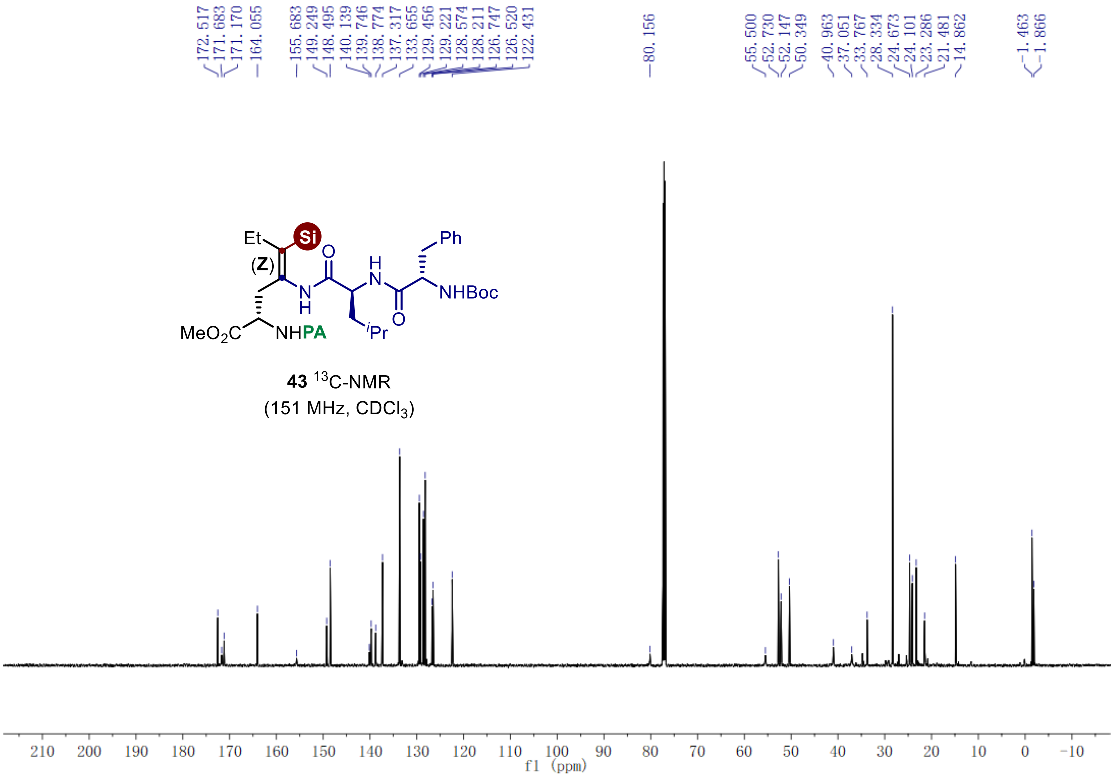

43 ^{13}C-NMR
(151 MHz, $CDCl_3$)

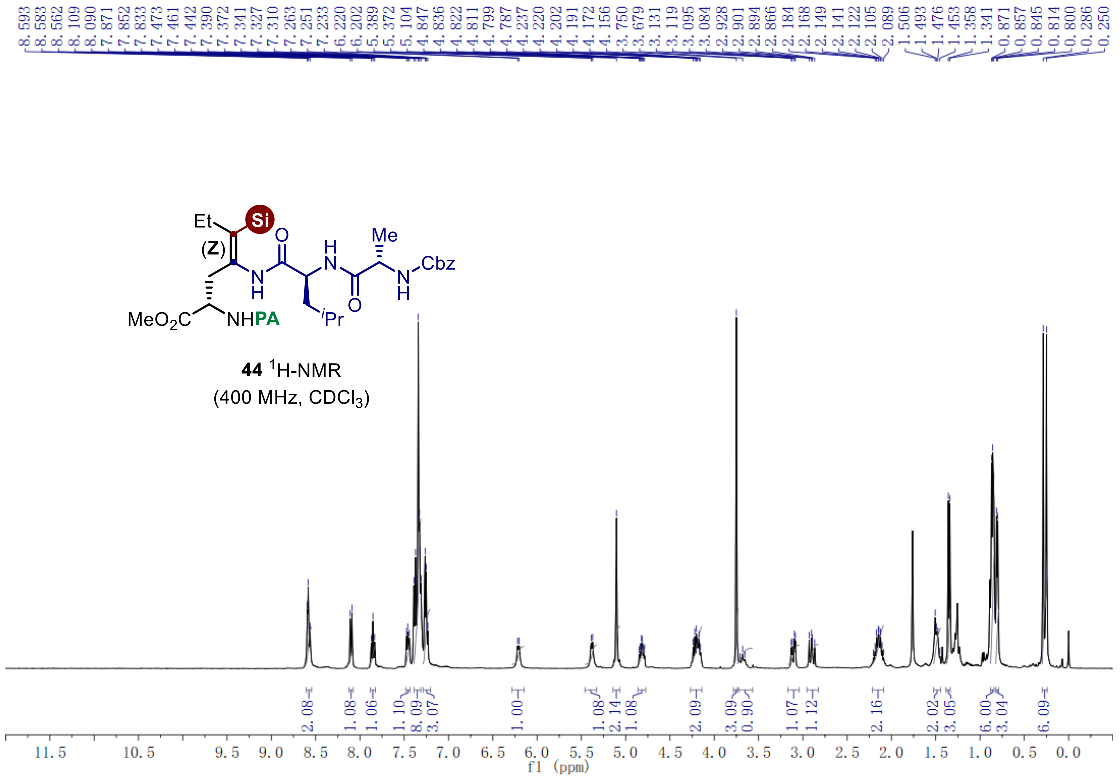
8.593 8.583 8.562 8.109 8.090 7.871 7.852 7.833 7.473 7.461 7.442 7.390 7.372 7.341 7.327 7.310 7.263 7.251 7.233 6.220 6.202 5.389 5.372 5.104 4.847 4.836 4.822 4.811 4.799 4.787 4.237 4.220 4.202 4.191 4.172 4.156 3.750 3.679 3.131 3.119 3.095 3.084 2.928 2.901 2.894 2.866 2.184 2.168 2.149 2.141 2.122 2.105 2.089 1.506 1.493 1.476 1.453 1.358 1.341 0.871 0.857 0.845 0.814 0.800 0.286 0.250
Et
Si
(Z)
O
H
N
Me
N
Cbz
H
H
N
O
MeO2C
NHPA
iPr
44 1H-NMR
(400 MHz, CDCl3)
2.08 1.08 1.06 1.10 8.09 3.07 1.00 1.08 2.14 1.08 2.09 3.09 0.90 1.07 1.12 2.16 2.02 3.05 6.00 3.04 6.09
11.5 10.5 9.5 9.0 8.5 8.0 7.5 7.0 6.5 6.0 5.5 5.0 4.5 4.0 3.5 3.0 2.5 2.0 1.5 1.0 0.5 0.0
f1 (ppm)

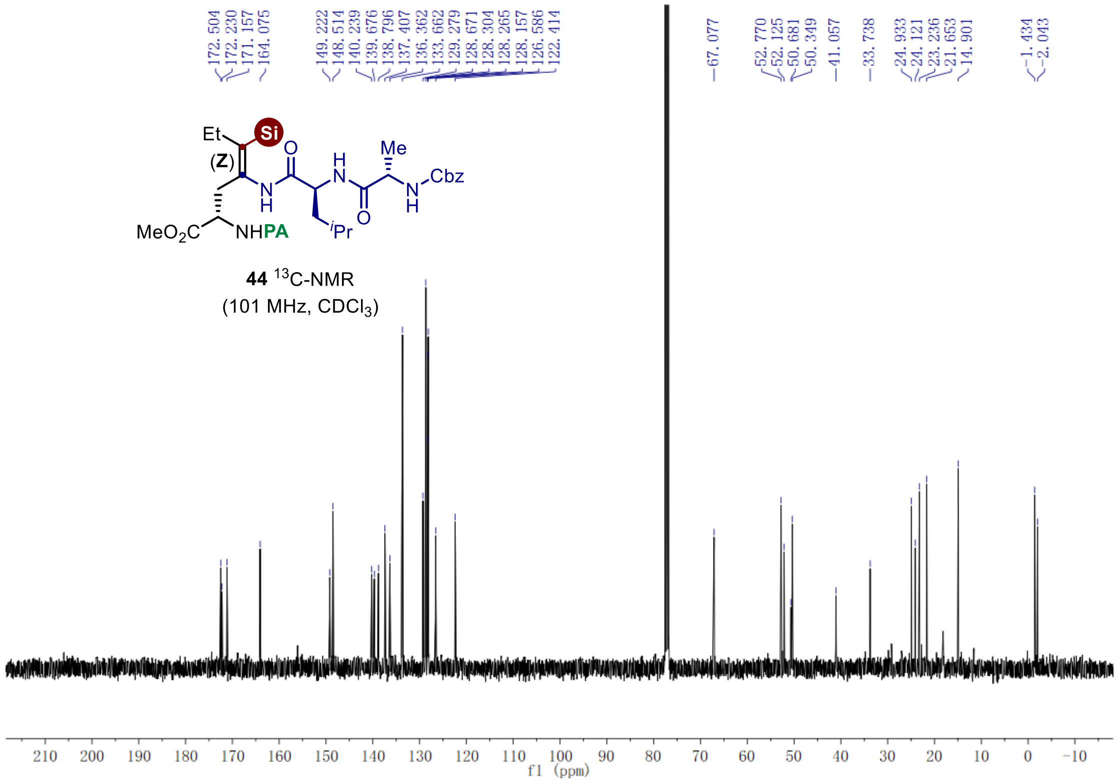
172.504 172.230 171.157 164.075 149.222 148.514 140.239 139.676 138.796 137.407 136.362 133.662 129.279 128.671 128.304 128.265 128.157 126.586 122.414 67.077 52.770 52.125 50.681 50.349 41.057 33.738 24.933 24.121 23.236 21.653 14.901 -1.434 -2.043
Et
Si
(Z)
O
H
N
Me
N
Cbz
H
H
N
O
MeO2C
NHPA
iPr
44 13C-NMR
(101 MHz, CDCl3)
210 200 190 180 170 160 150 140 130 120 110 100 90 80 70 60 50 40 30 20 10 0 -10
f1 (ppm)

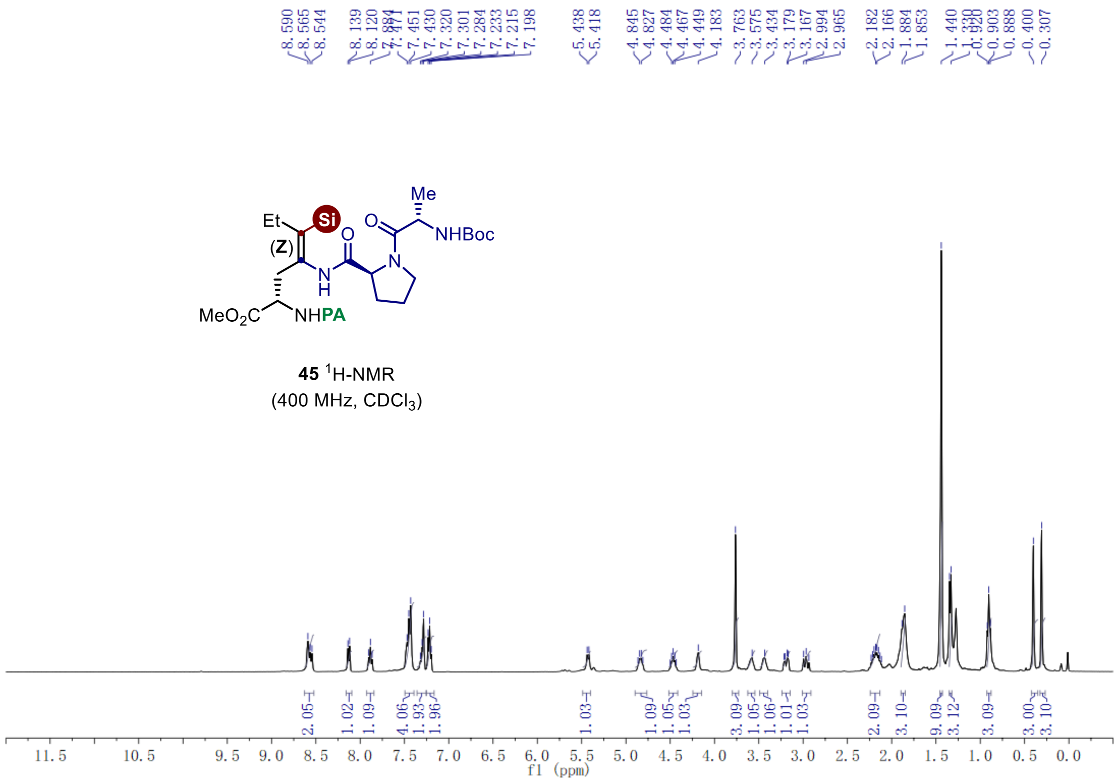
Et
Si
(Z)
O
O
Me
NHBoc
N
N
H
MeO2C
NHPA
45 1H-NMR
(400 MHz, CDCl3)
f1 (ppm)

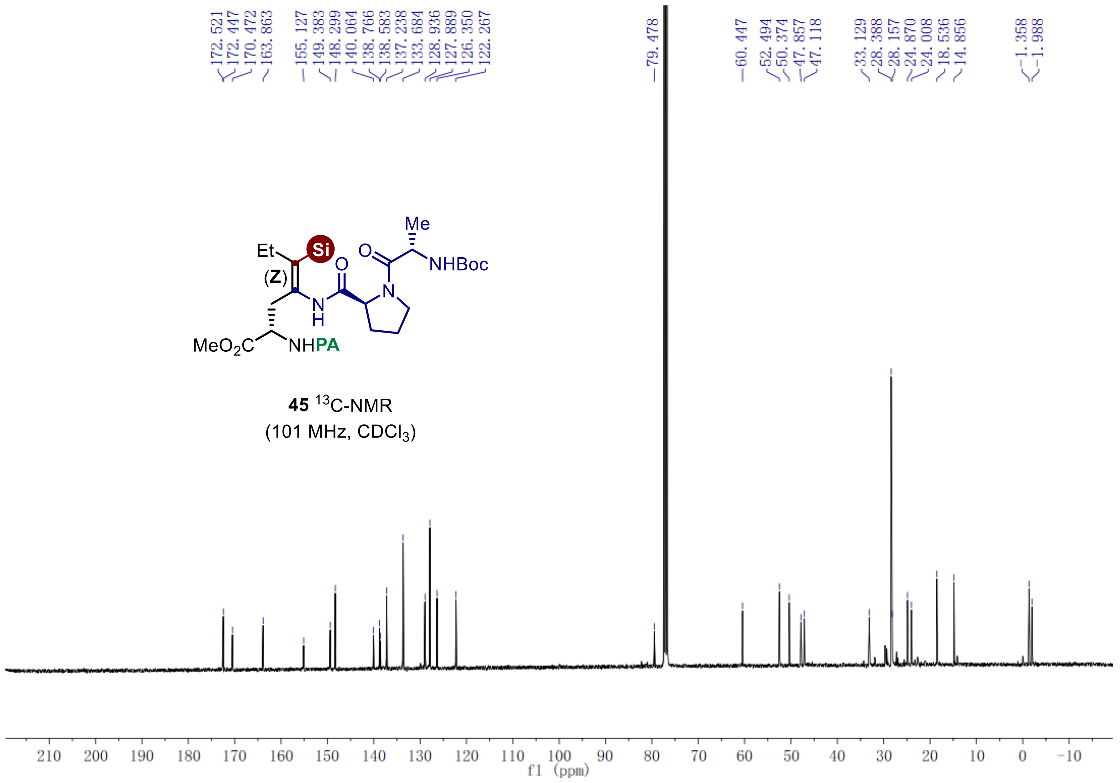
Et
Si
(Z)
O
O
Me
NHBoc
N
N
H
MeO2C
NHPA
45 13C-NMR
(101 MHz, CDCl3)
f1 (ppm)

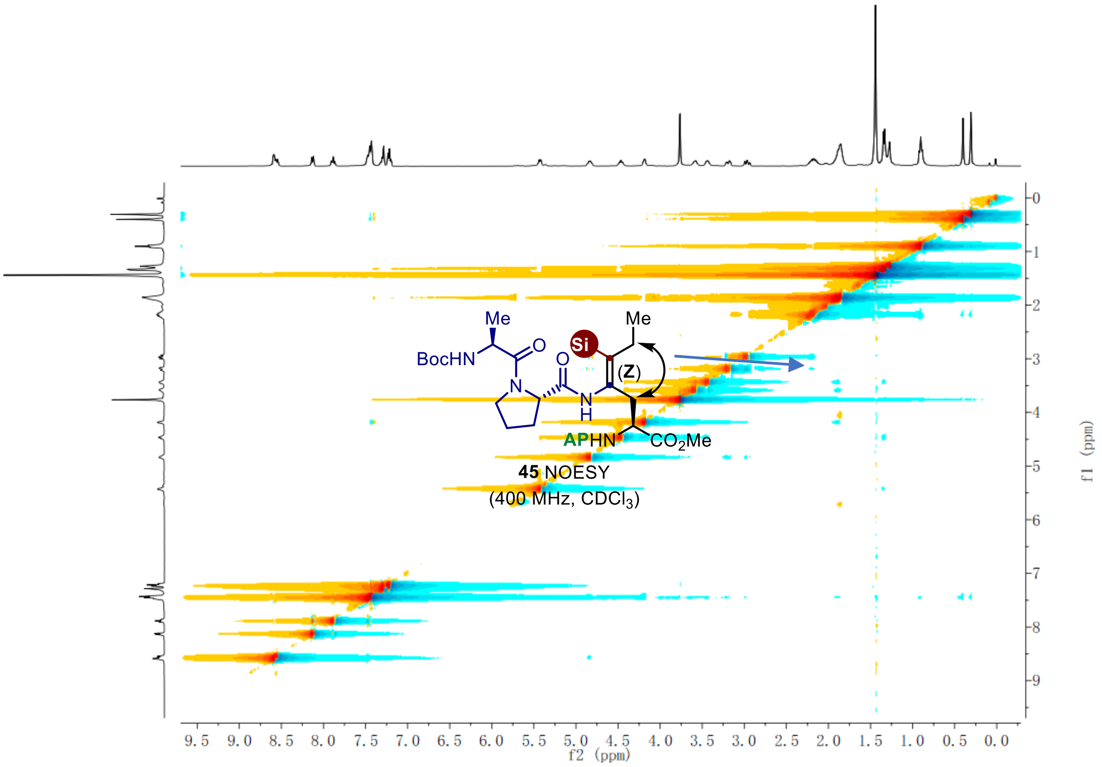
Me
BocHN
O
N
O
N
H
Si
Me
(Z)
APHN
CO2Me
45 NOESY
(400 MHz, CDCl3)
9.5 9.0 8.5 8.0 7.5 7.0 6.5 6.0 5.5 5.0 4.5 4.0 3.5 3.0 2.5 2.0 1.5 1.0 0.5 0.0
f2 (ppm)
0 1 2 3 4 5 6 7 8 9
f1 (ppm)

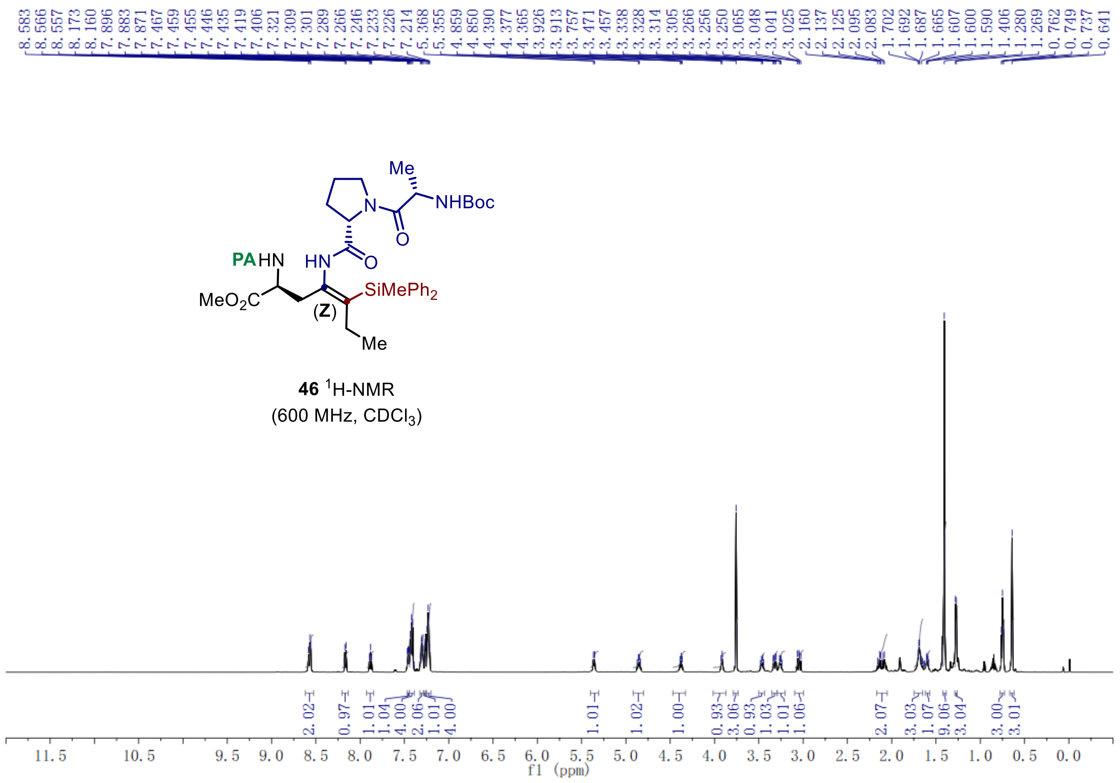
46 ^{1}H-NMR
(600 MHz, $CDCl_3$)
f1 (ppm)

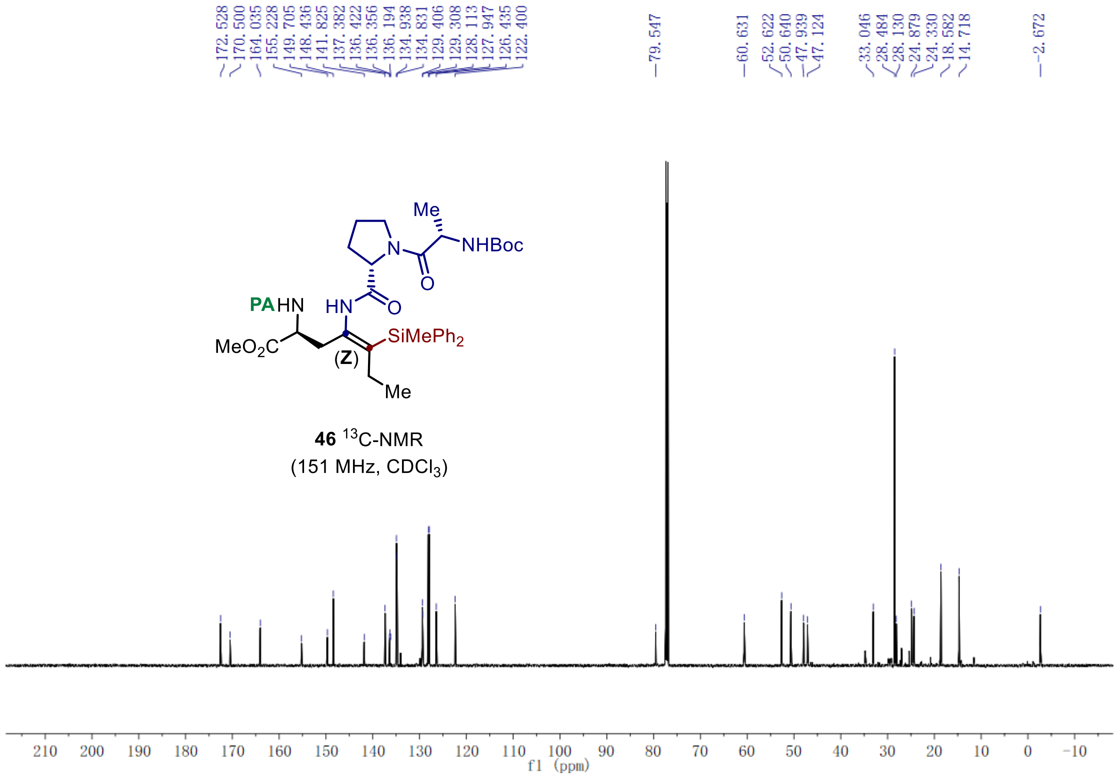
46 ^{13}C-NMR
(151 MHz, $CDCl_3$)
f1 (ppm)

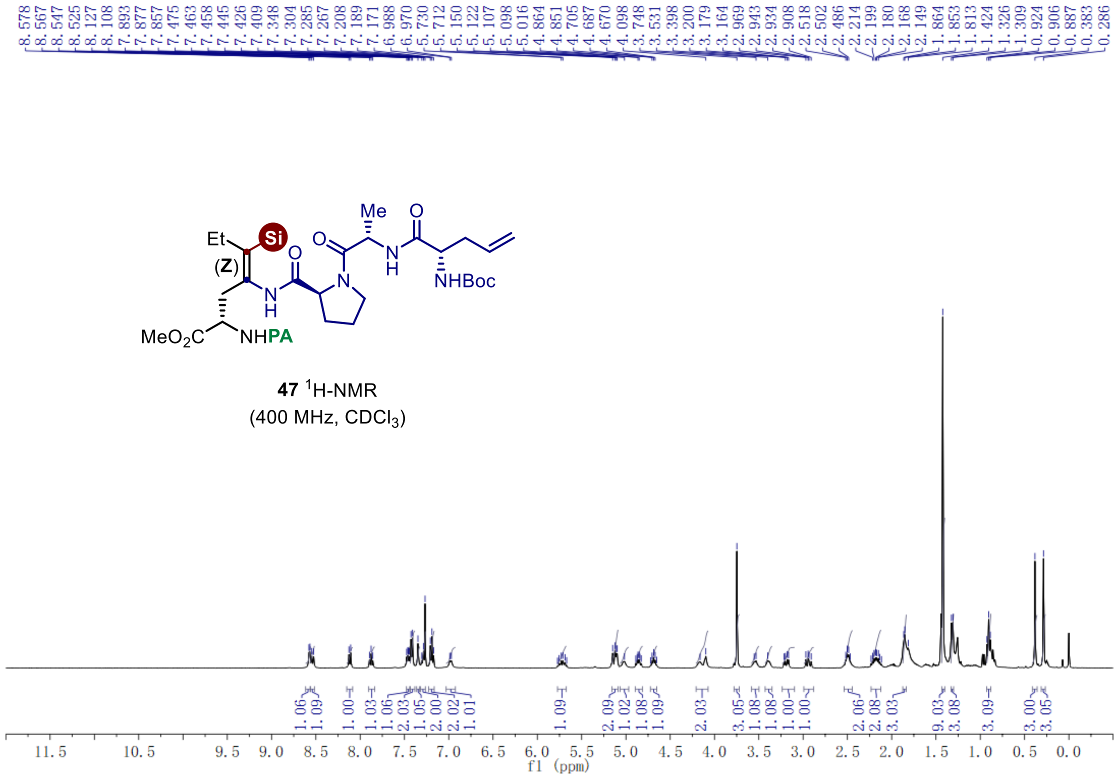

47 ^{1}H-NMR
(400 MHz, $CDCl_3$)

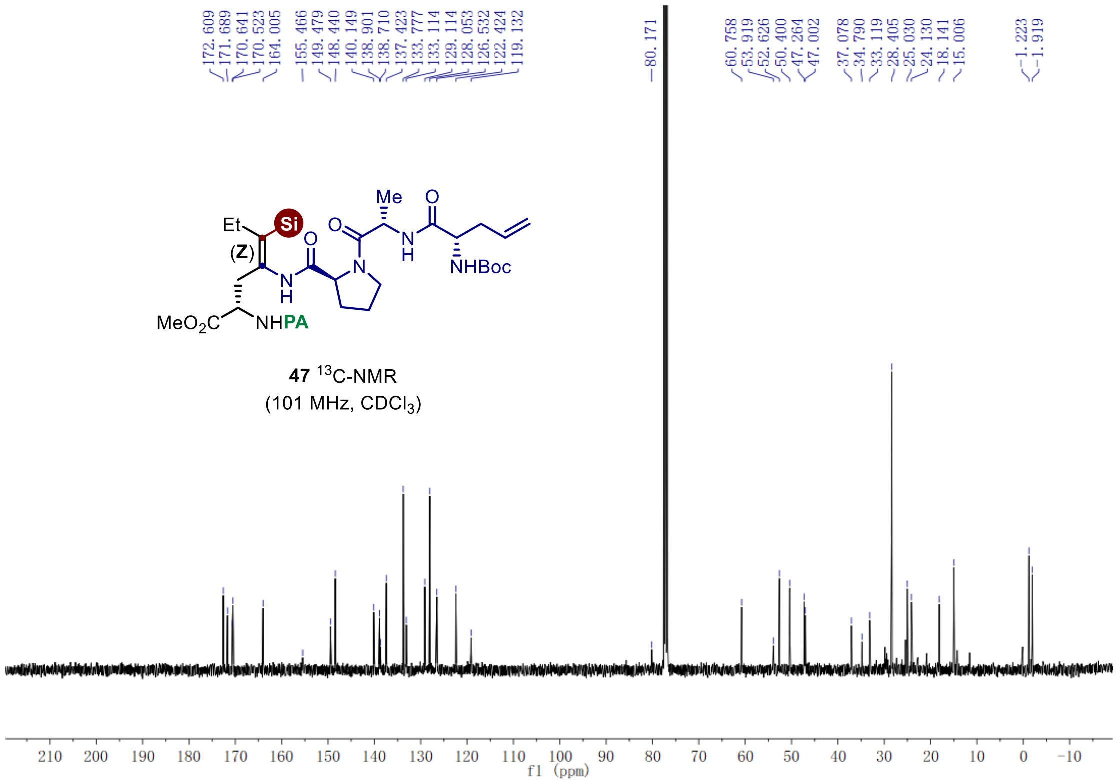

47 ^{13}C-NMR
(101 MHz, $CDCl_3$)

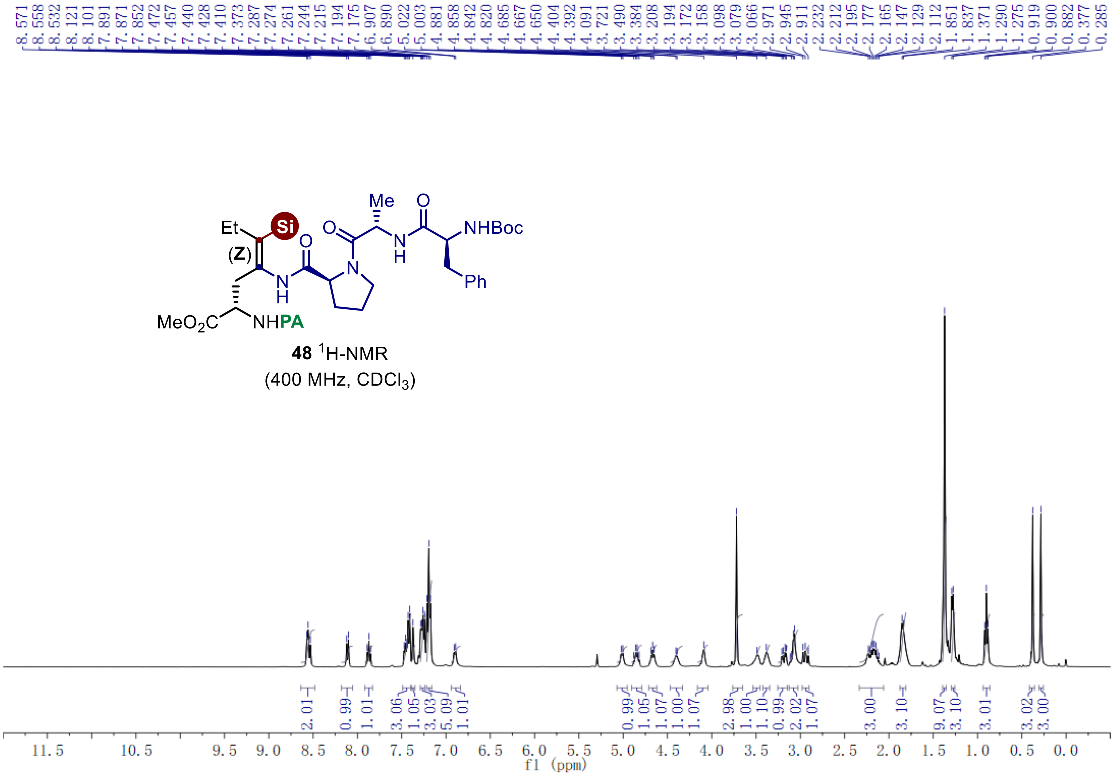
8.571 8.558 8.532 8.121 8.101 7.891 7.871 7.852 7.472 7.457 7.440 7.428 7.410 7.373 7.287 7.274 7.261 7.244 7.215 7.194 7.175 6.907 6.890 5.022 5.003 4.881 4.858 4.842 4.820 4.685 4.667 4.650 4.404 4.392 4.091 3.721 3.490 3.384 3.208 3.194 3.172 3.158 3.098 3.079 3.066 2.971 2.945 2.911 2.232 2.212 2.195 2.177 2.165 2.147 2.129 2.112 1.851 1.837 1.371 1.290 1.275 0.919 0.900 0.882 0.377 0.285
Et
Si
(Z)
O
Me
O
NHBoc
N
H
N
N
H
Ph
MeO2C
NHPA
48 1H-NMR
(400 MHz, CDCl3)
f1 (ppm)

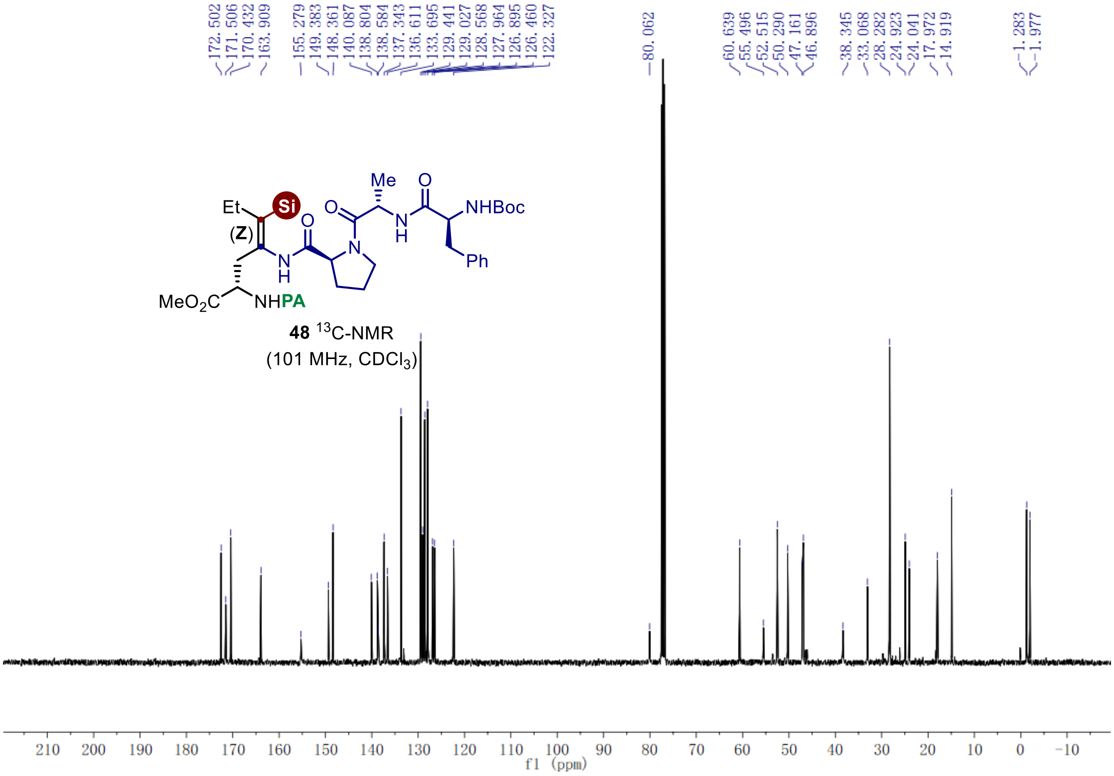
172.502 171.506 170.432 163.909 155.279 149.383 148.361 140.087 138.804 138.584 137.343 136.611 133.695 129.441 129.027 128.568 127.964 126.895 126.460 122.327 80.062 60.639 55.496 52.515 50.290 47.161 46.896 38.345 33.068 28.282 24.923 24.041 17.972 14.919 -1.283 -1.977
Et
Si
(Z)
O
Me
O
NHBoc
N
H
N
N
H
Ph
MeO2C
NHPA
48 13C-NMR
(101 MHz, CDCl3)
f1 (ppm)

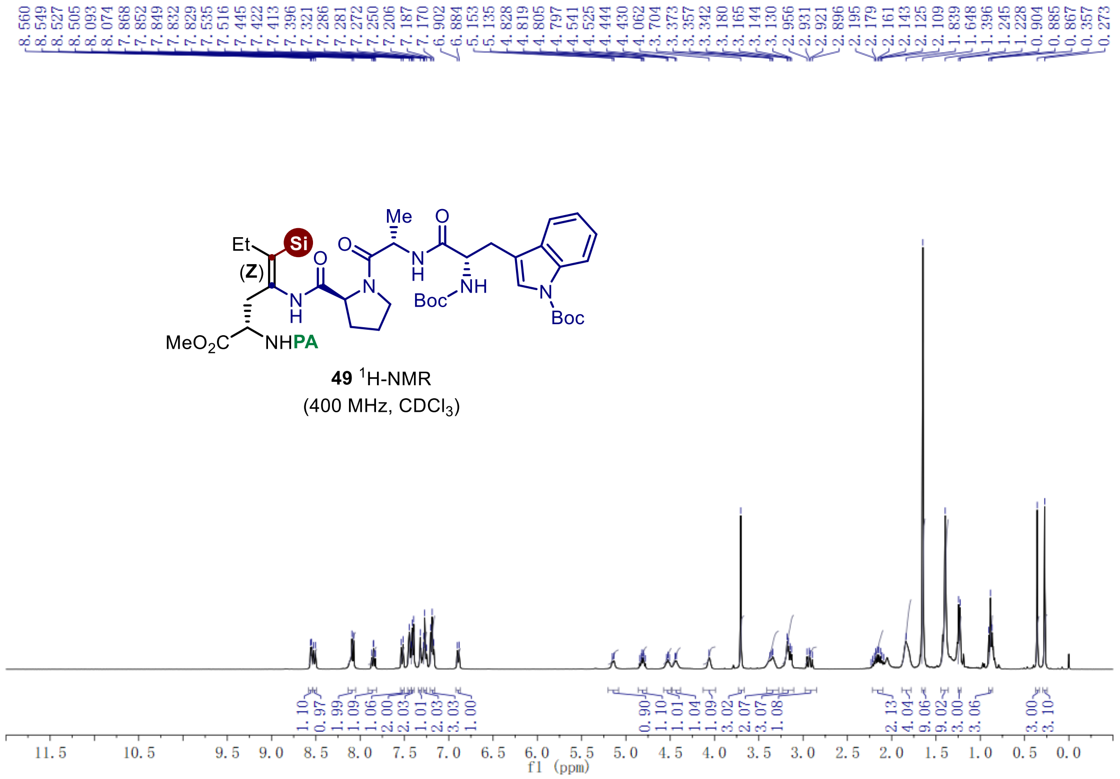
8.560 8.549 8.527 8.505 8.093 8.074 7.868 7.852 7.849 7.832 7.829 7.535 7.516 7.445 7.422 7.413 7.396 7.321 7.286 7.281 7.272 7.250 7.206 7.187 7.170 6.902 6.884 5.153 5.135 4.828 4.819 4.805 4.797 4.541 4.525 4.444 4.430 4.062 3.704 3.373 3.357 3.342 3.180 3.165 3.144 3.130 2.956 2.931 2.921 2.896 2.195 2.179 2.161 2.143 2.125 2.109 1.839 1.648 1.396 1.245 1.228 0.904 0.885 0.867 0.357 0.273
Et
Si
(Z)
O
N
H
MeO2C
NHPA
Me
O
O
N
N
H
Boc
NH
N
Boc
49 1H-NMR
(400 MHz, CDCl3)
1.10 0.97 1.99 1.09 1.06 2.00 2.03 1.01 2.03 3.03 1.00 0.90 1.10 1.01 1.04 1.09 3.02 2.07 3.07 1.08 2.13 4.04 9.06 9.02 3.00 3.06 3.00 3.10
11.5 10.5 9.5 9.0 8.5 8.0 7.5 7.0 6.5 6.0 5.5 5.0 4.5 4.0 3.5 3.0 2.5 2.0 1.5 1.0 0.5 0.0
f1 (ppm)

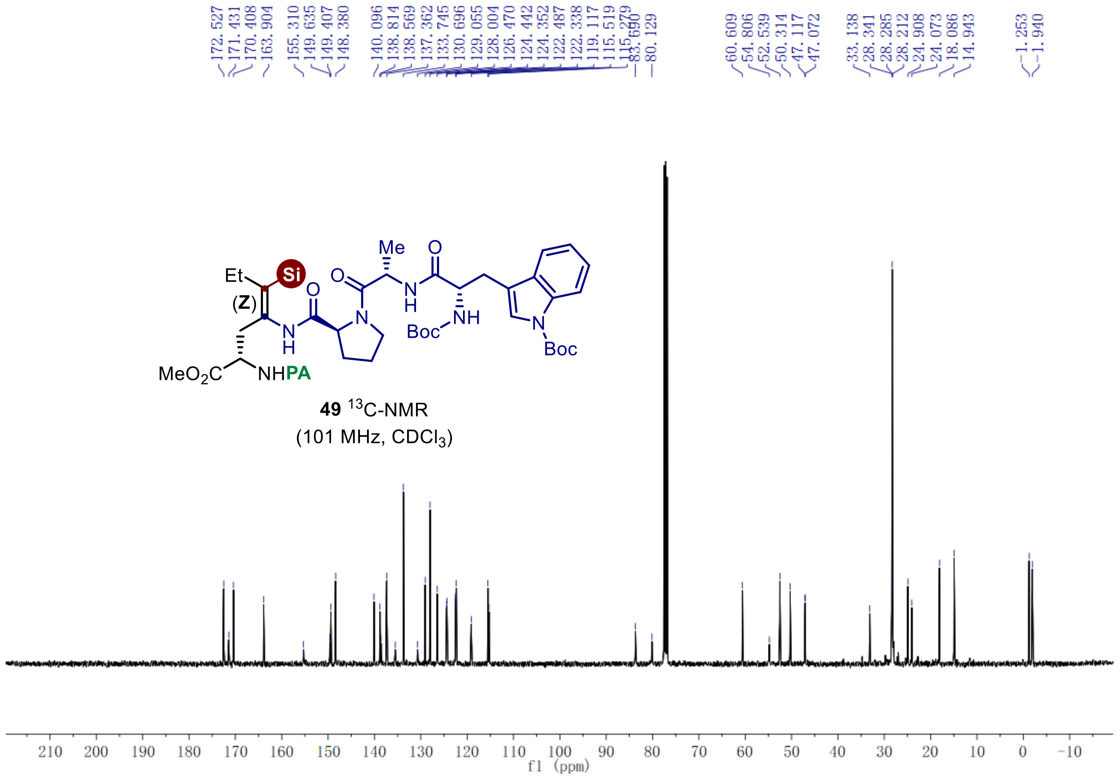
172.527 171.431 170.408 163.904 155.310 149.635 149.407 148.380 140.096 138.814 138.569 137.362 133.745 130.696 129.055 128.004 126.470 124.442 124.352 122.487 122.338 119.117 115.519 115.279 83.690 80.129 60.609 54.806 52.539 50.314 47.117 47.072 33.138 28.341 28.285 28.212 24.908 24.073 18.086 14.943 -1.253 -1.940
Et
Si
(Z)
O
N
H
MeO2C
NHPA
Me
O
O
N
N
H
Boc
NH
N
Boc
49 13C-NMR
(101 MHz, CDCl3)
210 200 190 180 170 160 150 140 130 120 110 100 90 80 70 60 50 40 30 20 10 0 -10
f1 (ppm)

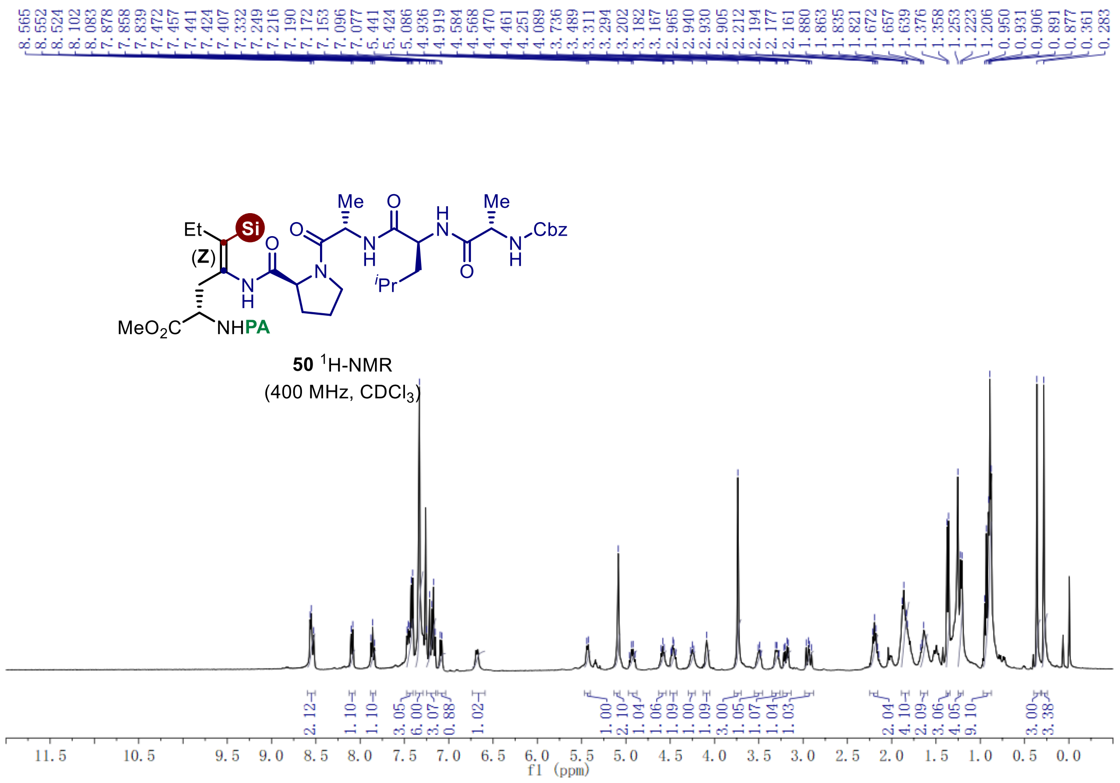
8.565 8.552 8.524 8.102 8.083 7.878 7.858 7.839 7.472 7.457 7.441 7.424 7.407 7.332 7.249 7.216 7.190 7.172 7.153 7.096 7.077 5.441 5.424 5.086 4.936 4.919 4.584 4.568 4.470 4.461 4.251 4.089 3.736 3.489 3.311 3.294 3.202 3.182 3.167 2.965 2.940 2.930 2.905 2.212 2.194 2.177 2.161 1.880 1.863 1.835 1.821 1.672 1.657 1.639 1.376 1.358 1.253 1.223 1.206 0.950 0.931 0.906 0.891 0.877 0.361 0.283
Et
Si
(Z)
Me
O
N
H
NHPA
MeO2C
iPr
Cbz
50 1H-NMR
(400 MHz, CDCl3)
2.12 1.10 1.10 3.05 6.00 3.07 0.88 1.02 1.00 2.10 1.04 1.06 1.09 1.00 1.09 3.00 1.05 1.07 1.04 1.03 2.04 4.10 2.09 3.06 4.05 9.10 3.00 3.38
11.5 10.5 9.5 9.0 8.5 8.0 7.5 7.0 6.5 6.0 5.5 5.0 4.5 4.0 3.5 3.0 2.5 2.0 1.5 1.0 0.5 0.0
f1 (ppm)

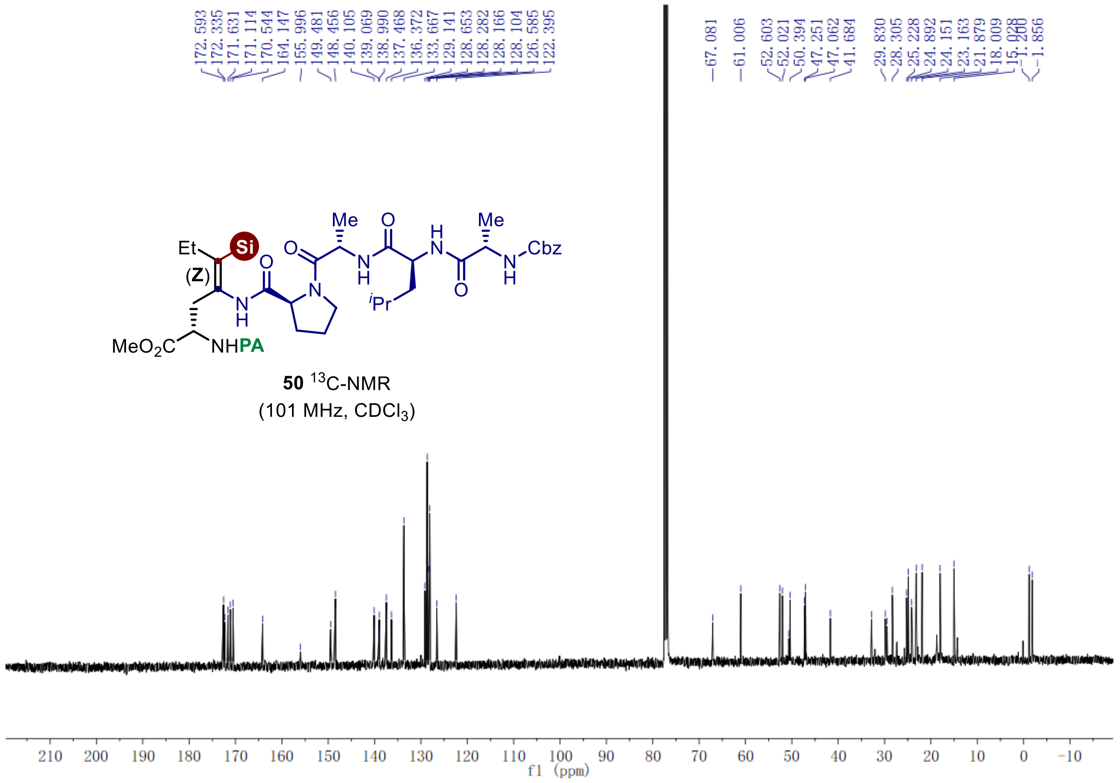
172.593 172.335 171.631 171.114 170.544 164.147 155.996 149.481 148.456 140.105 139.069 138.990 137.468 136.372 133.667 129.141 128.653 128.282 128.166 128.104 126.585 122.395
67.081 61.006 52.603 52.021 50.394 47.251 47.062 41.684 29.830 28.305 25.228 24.892 24.151 23.163 21.879 18.009 -1.856
Et
Si
(Z)
Me
O
N
H
NHPA
MeO2C
iPr
Cbz
50 13C-NMR
(101 MHz, CDCl3)
210 200 190 180 170 160 150 140 130 120 110 100 90 80 70 60 50 40 30 20 10 0 -10
f1 (ppm)

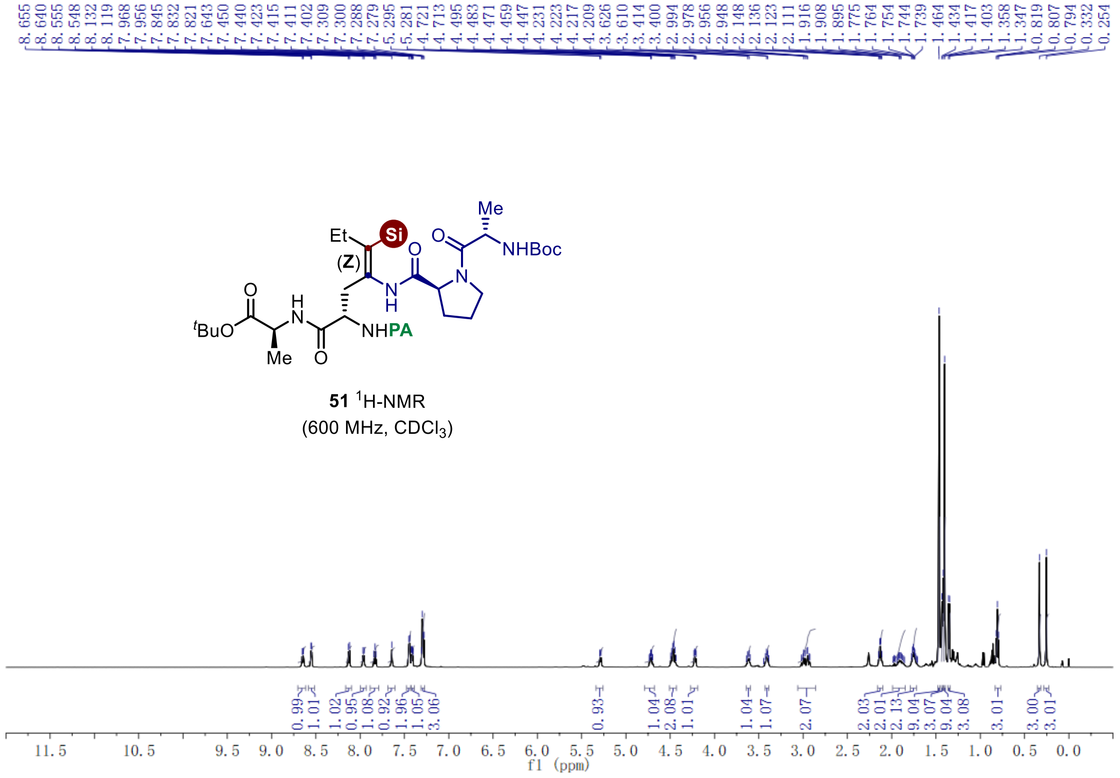
8.655 8.640 8.555 8.548 8.132 8.119 7.968 7.956 7.845 7.832 7.821 7.643 7.450 7.440 7.423 7.415 7.411 7.402 7.309 7.300 7.288 7.279 5.295 5.281 4.721 4.713 4.495 4.483 4.471 4.459 4.447 4.231 4.223 4.217 4.209 3.626 3.610 3.414 3.400 2.994 2.978 2.956 2.948 2.148 2.136 2.123 2.111 1.916 1.908 1.895 1.775 1.764 1.754 1.744 1.739 1.464 1.434 1.417 1.403 1.358 1.347 0.819 0.807 0.794 0.332 0.254
Et
Si
(Z)
Me
NHBoc
N
H
O
tBuO
NHPA
51 1H-NMR
(600 MHz, CDCl3)
0.99 1.01 1.02 0.95 1.08 0.92 1.96 1.05 3.06 0.93 1.04 2.08 1.01 1.04 1.07 2.07 2.03 2.01 2.13 9.04 3.07 9.04 3.08 3.01 3.00 3.01
11.5 10.5 9.5 9.0 8.5 8.0 7.5 7.0 6.5 6.0 5.5 5.0 4.5 4.0 3.5 3.0 2.5 2.0 1.5 1.0 0.5 0.0
f1 (ppm)

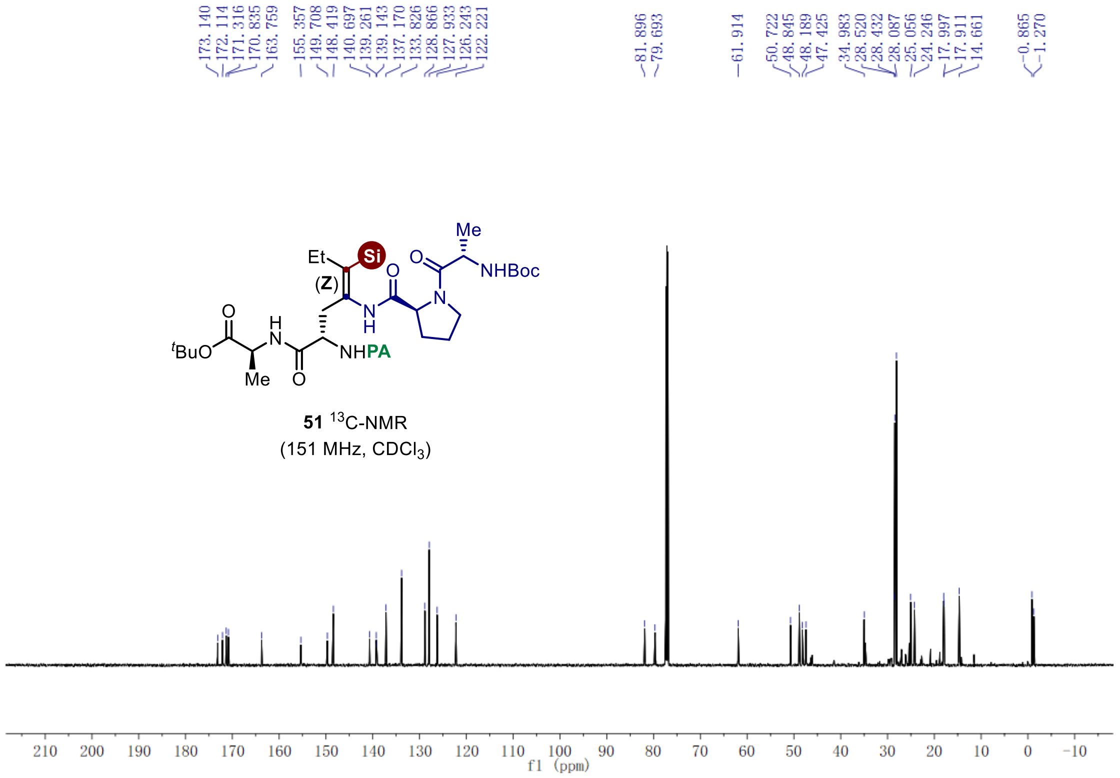
173.140 172.114 171.316 170.835 163.759 155.357 149.708 148.419 140.697 139.261 139.143 137.170 133.826 128.866 127.933 126.243 122.221 81.896 79.693 61.914 50.722 48.845 48.189 47.425 34.983 28.520 28.432 28.087 25.056 24.246 17.997 17.911 14.661 -0.865 -1.270
Et
Si
(Z)
Me
NHBoc
N
H
O
tBuO
NHPA
51 13C-NMR
(151 MHz, CDCl3)
210 200 190 180 170 160 150 140 130 120 110 100 90 80 70 60 50 40 30 20 10 0 -10
f1 (ppm)

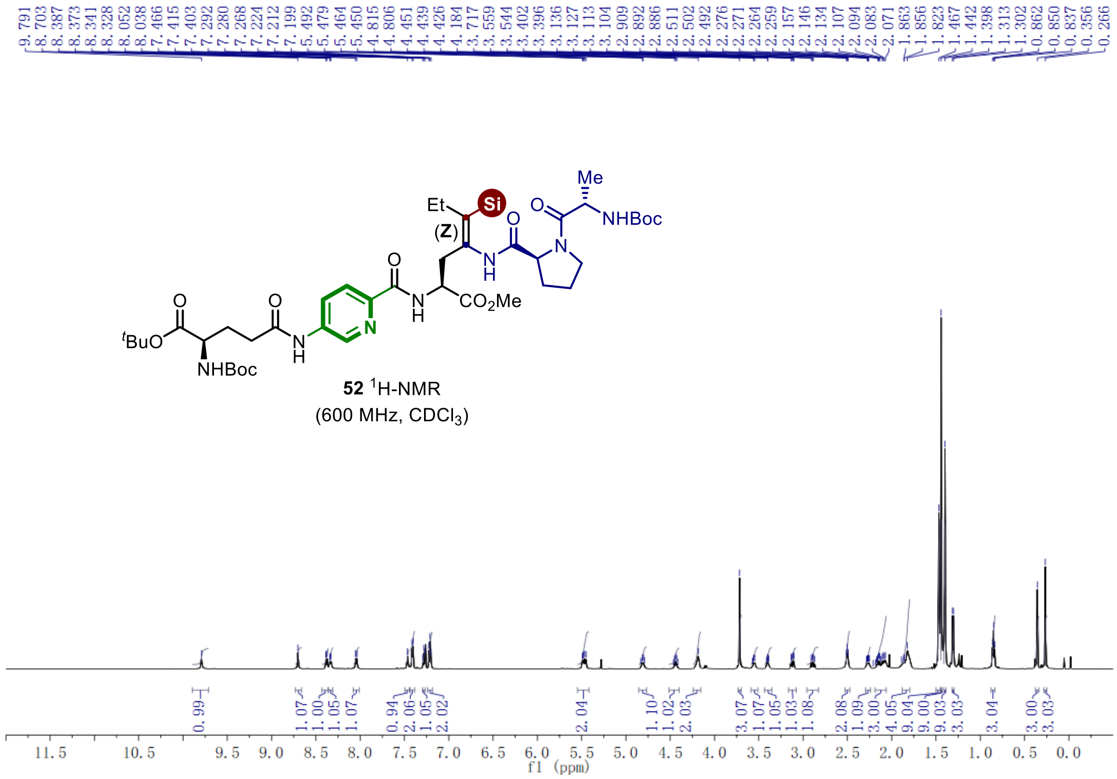
52 1H-NMR
(600 MHz, CDCl3)
f1 (ppm)

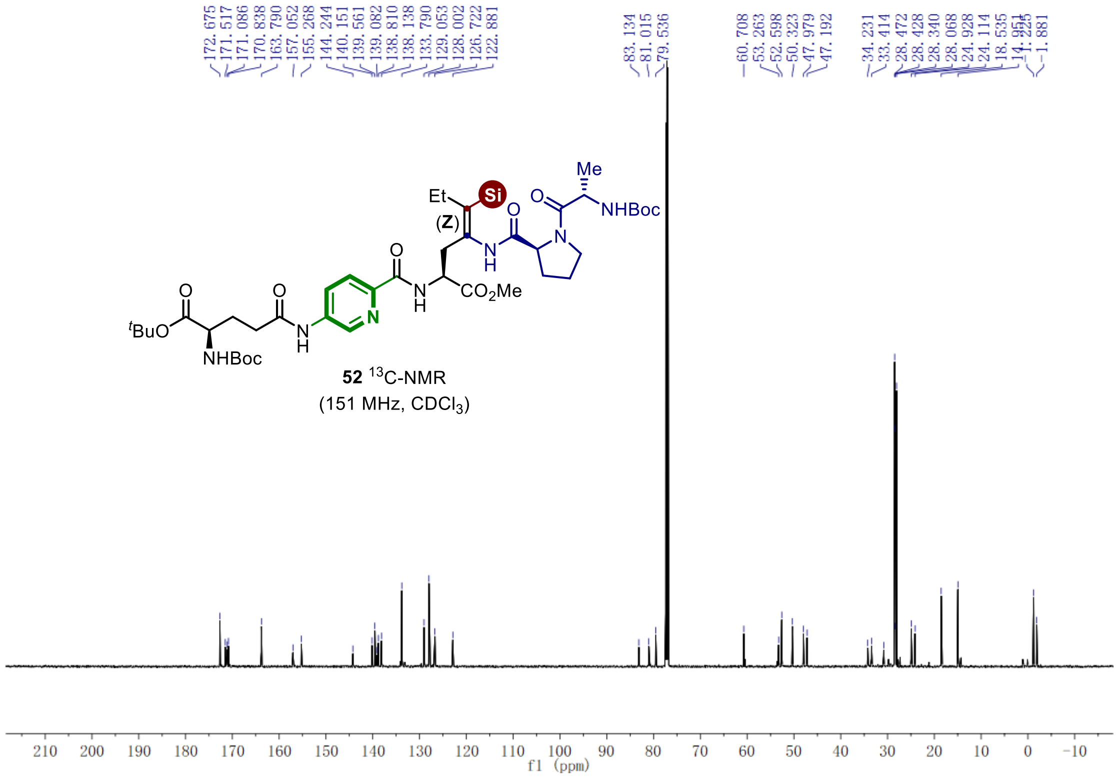
52 13C-NMR
(151 MHz, CDCl3)
f1 (ppm)

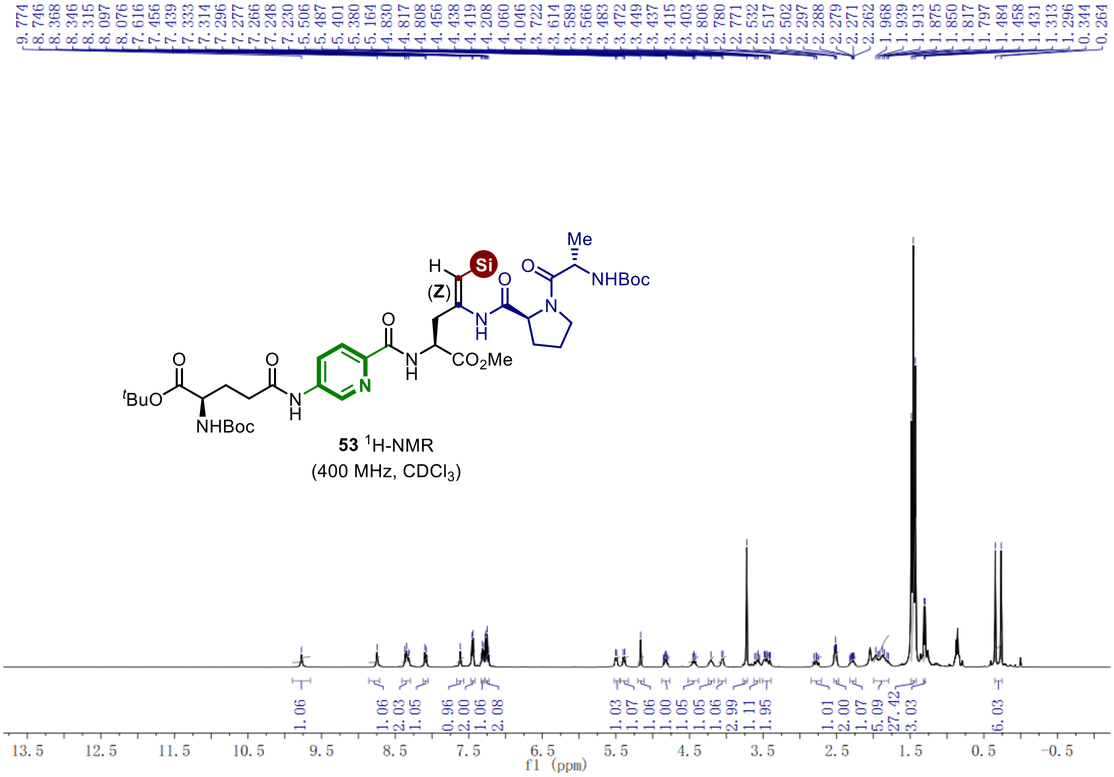

53 ^{1}H-NMR
(400 MHz, $CDCl_3$)

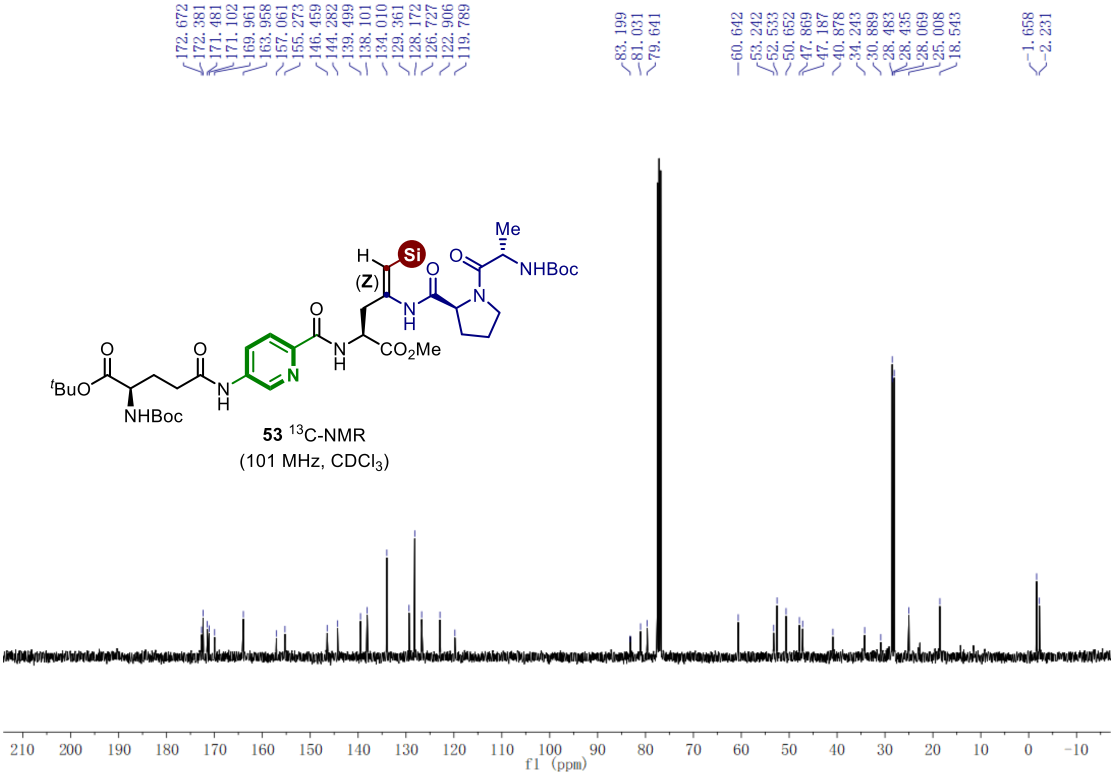

53 ^{13}C-NMR
(101 MHz, $CDCl_3$)

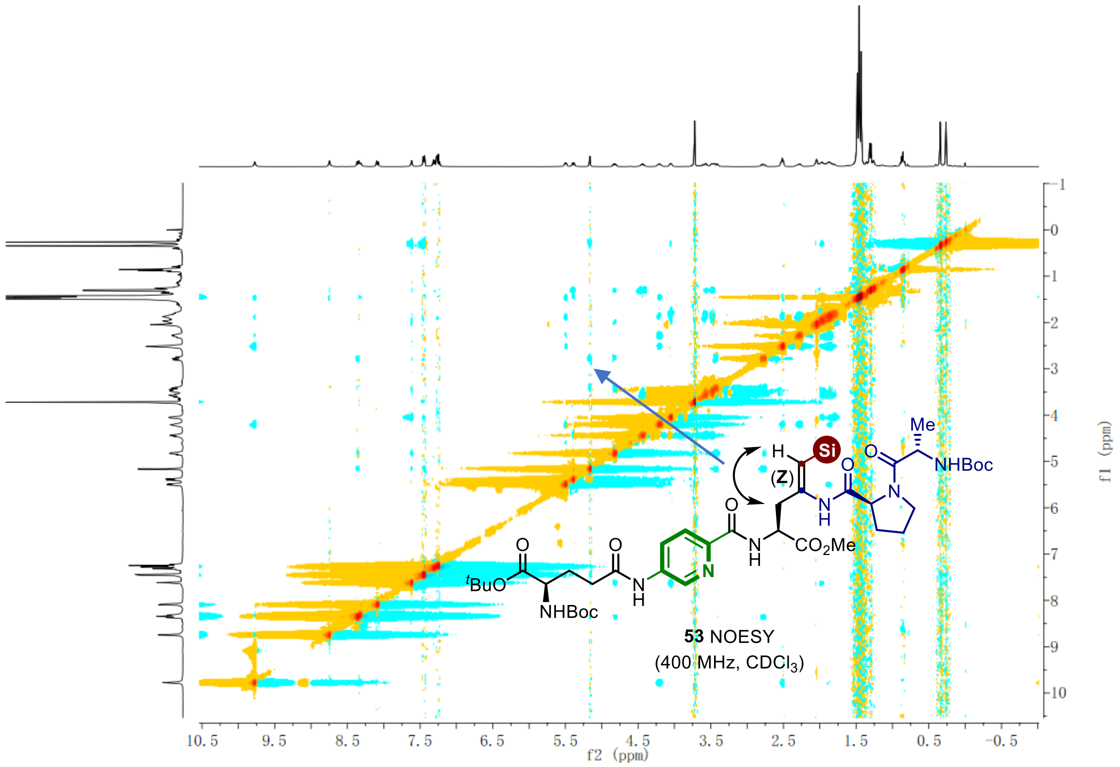

f2 (ppm)
f1 (ppm)
H
Si
(Z)
O
NH
O
N
Me
NHBoc
O
N
H
CO2Me
N
O
tBuO
O
NHBoc
N
H
53 NOESY
(400 MHz, CDCl3)

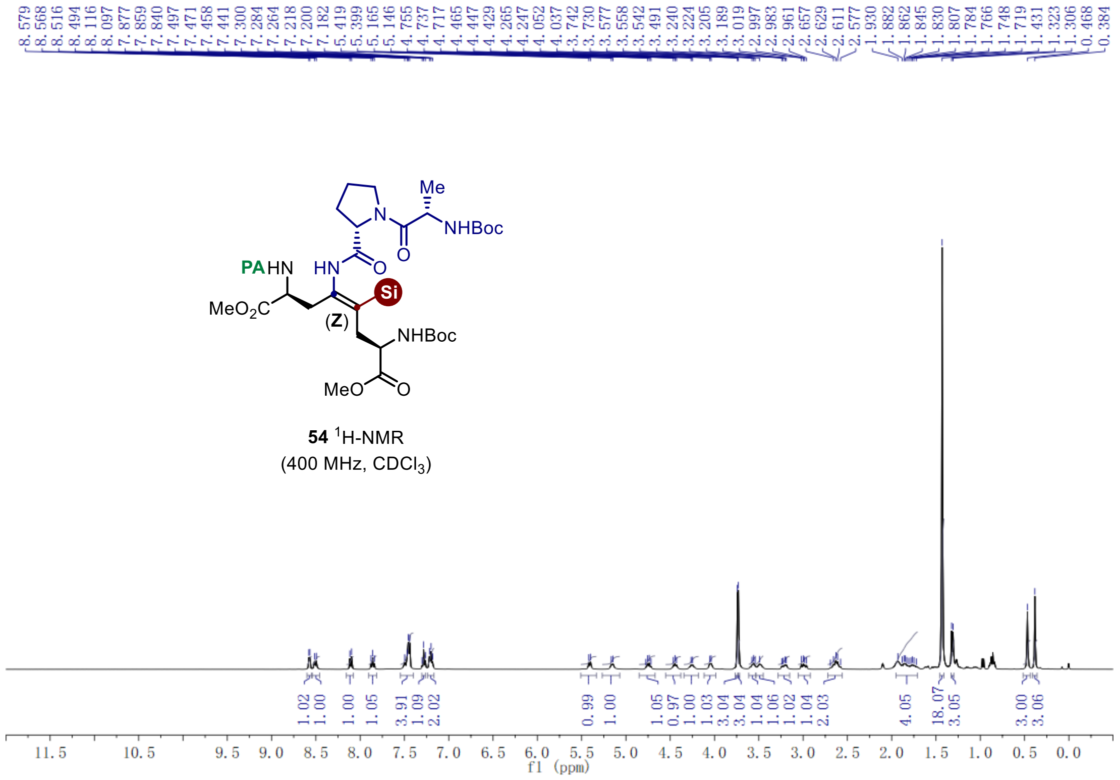
8.579 8.568 8.516 8.494 8.116 8.097 7.877 7.859 7.840 7.497 7.471 7.458 7.441 7.300 7.284 7.264 7.218 7.200 7.182 5.419 5.399 5.165 5.146 4.755 4.737 4.717 4.465 4.447 4.429 4.265 4.247 4.052 4.037 3.742 3.730 3.577 3.558 3.542 3.491 3.240 3.224 3.205 3.189 3.019 2.997 2.983 2.961 2.657 2.629 2.611 2.577 1.930 1.882 1.862 1.845 1.830 1.807 1.784 1.766 1.748 1.719 1.431 1.323 1.306 0.468 0.384
Me
NHBoc
N
O
PA HN
HN
O
Si
MeO2C
(Z)
NHBoc
MeO
O
54 1H-NMR
(400 MHz, CDCl3)
1.02 1.00 1.00 1.05 3.91 1.09 2.02 0.99 1.00 1.05 0.97 1.00 1.03 3.04 3.04 1.04 1.06 1.02 1.04 2.03 4.05 18.07 3.05 3.00 3.06
11.5 10.5 9.5 9.0 8.5 8.0 7.5 7.0 6.5 6.0 5.5 5.0 4.5 4.0 3.5 3.0 2.5 2.0 1.5 1.0 0.5 0.0
f1 (ppm)

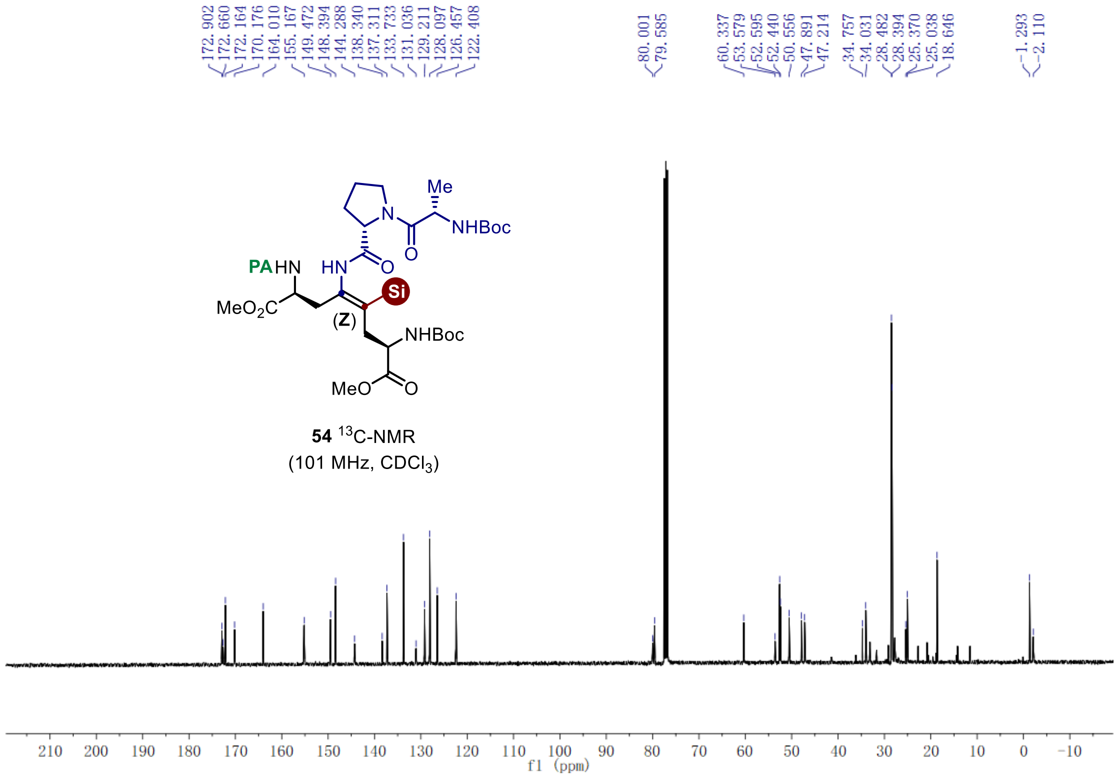
172.902 172.660 172.164 170.176 164.010 155.167 149.472 148.394 144.288 138.340 137.311 133.733 131.036 129.211 128.097 126.457 122.408 80.001 79.585 60.337 53.579 52.595 52.440 50.556 47.891 47.214 34.757 34.031 28.482 28.394 25.370 25.038 18.646 -1.293 -2.110
Me
NHBoc
N
O
PA HN
HN
O
Si
MeO2C
(Z)
NHBoc
MeO
O
54 13C-NMR
(101 MHz, CDCl3)
210 200 190 180 170 160 150 140 130 120 110 100 90 80 70 60 50 40 30 20 10 0 -10
f1 (ppm)

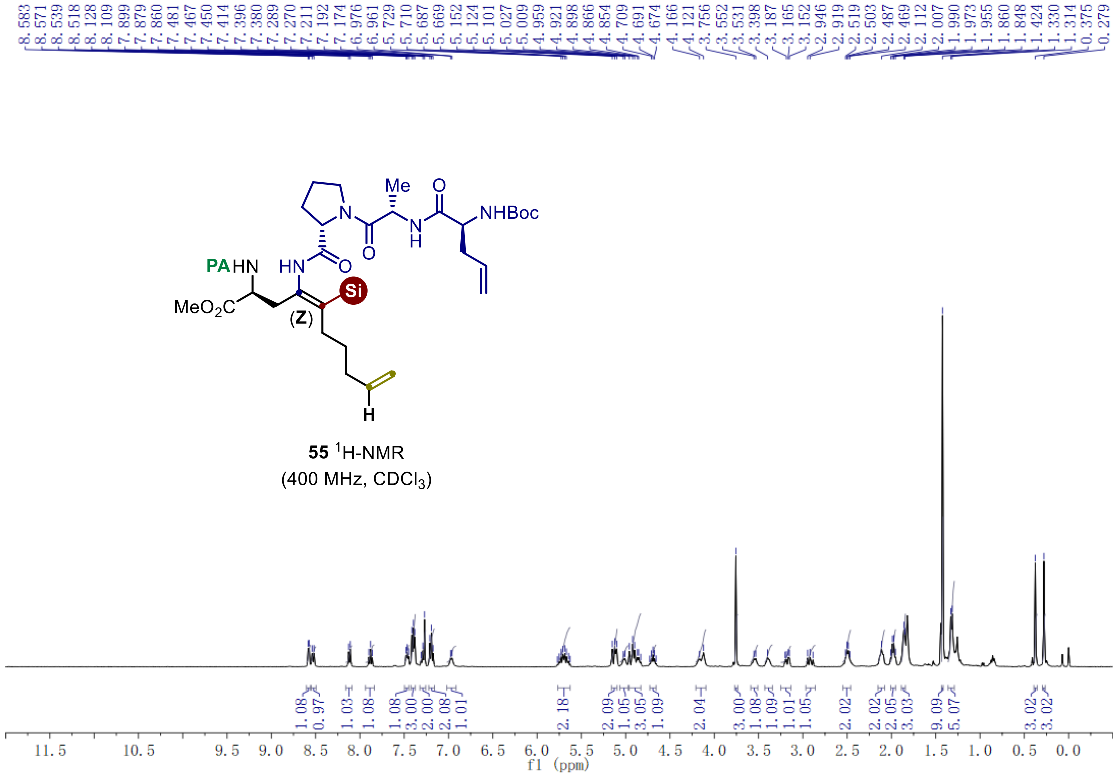

55 ^{1}H-NMR
(400 MHz, $CDCl_3$)

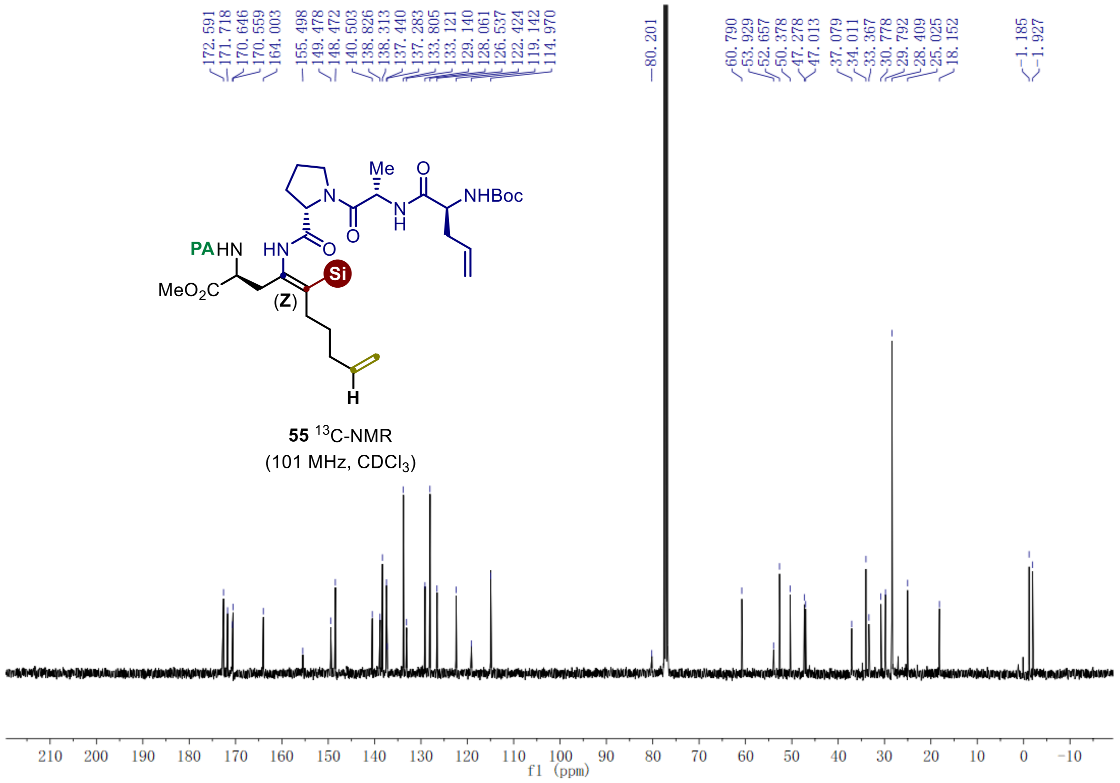

55 ^{13}C-NMR
(101 MHz, $CDCl_3$)

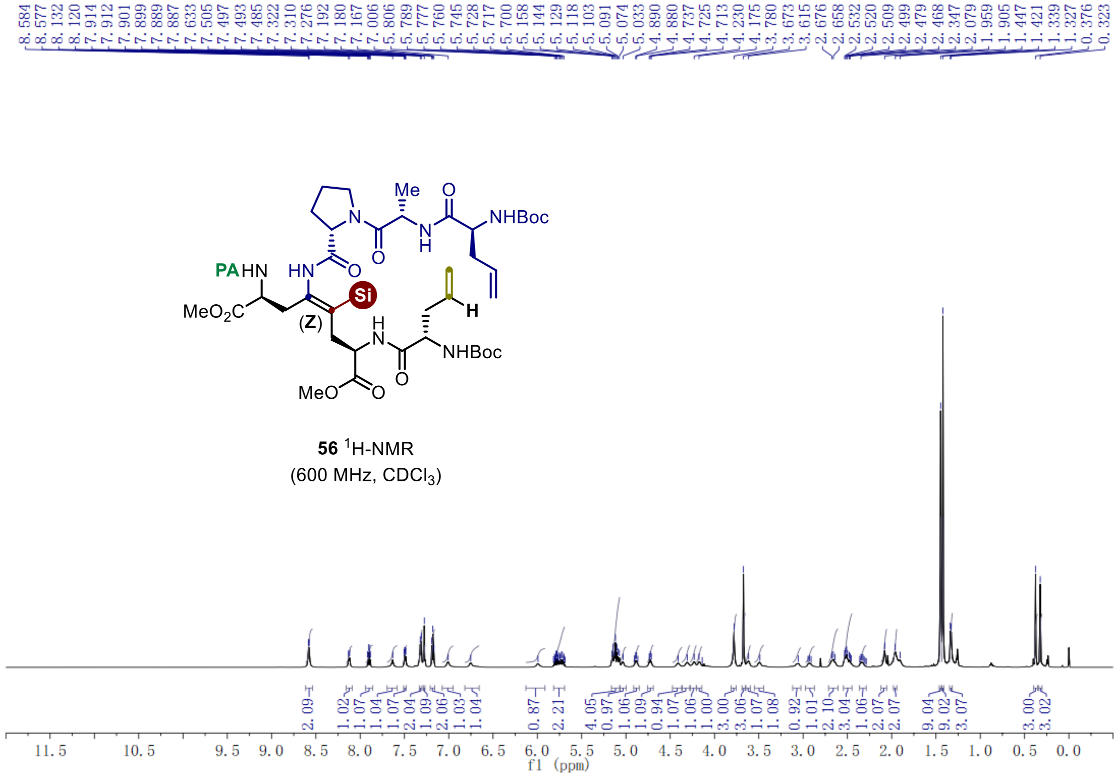

56 ^{1}H-NMR
(600 MHz, $CDCl_3$)

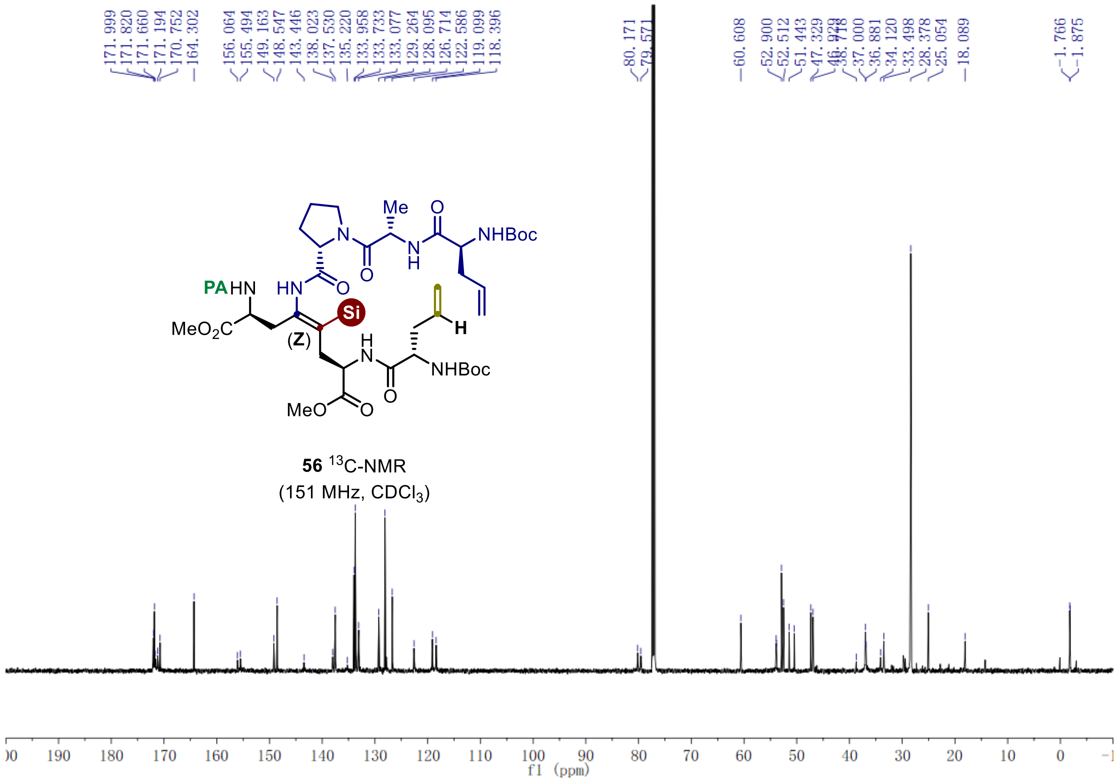

56 ^{13}C-NMR
(151 MHz, $CDCl_3$)

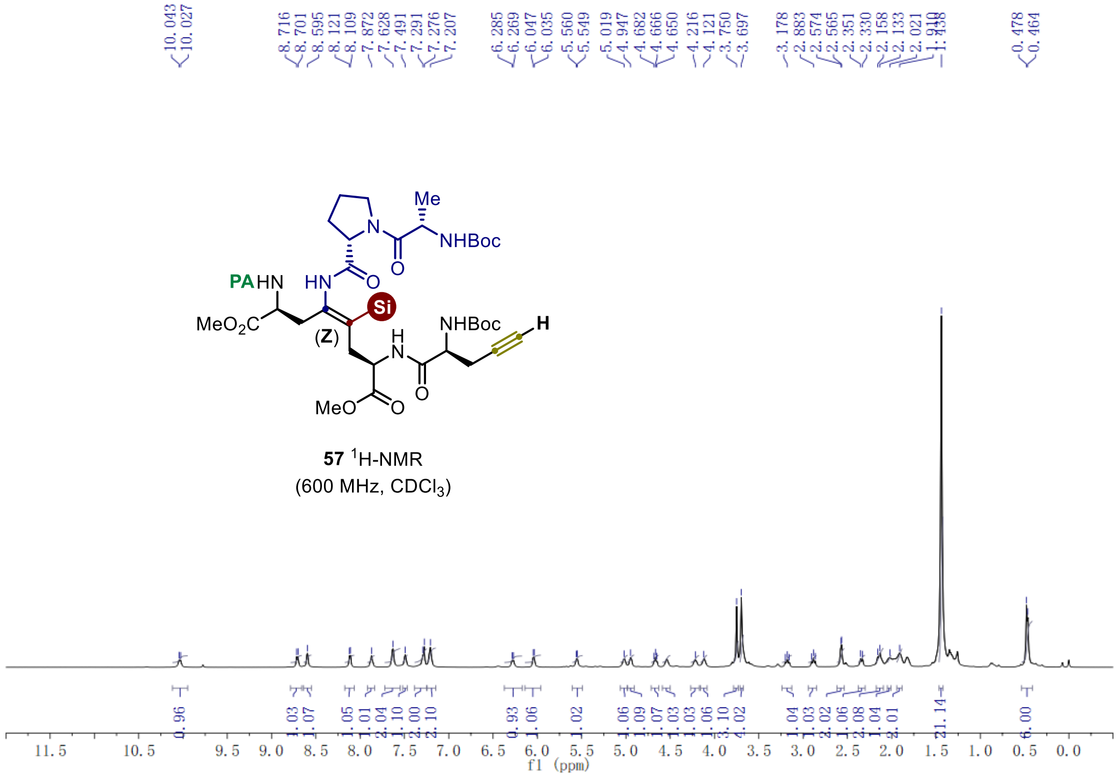
57 ¹H-NMR
(600 MHz, CDCl3)
PAHN
HN
MeO2C
Si
(Z)
NHBoc
Me
H
MeO
f1 (ppm)

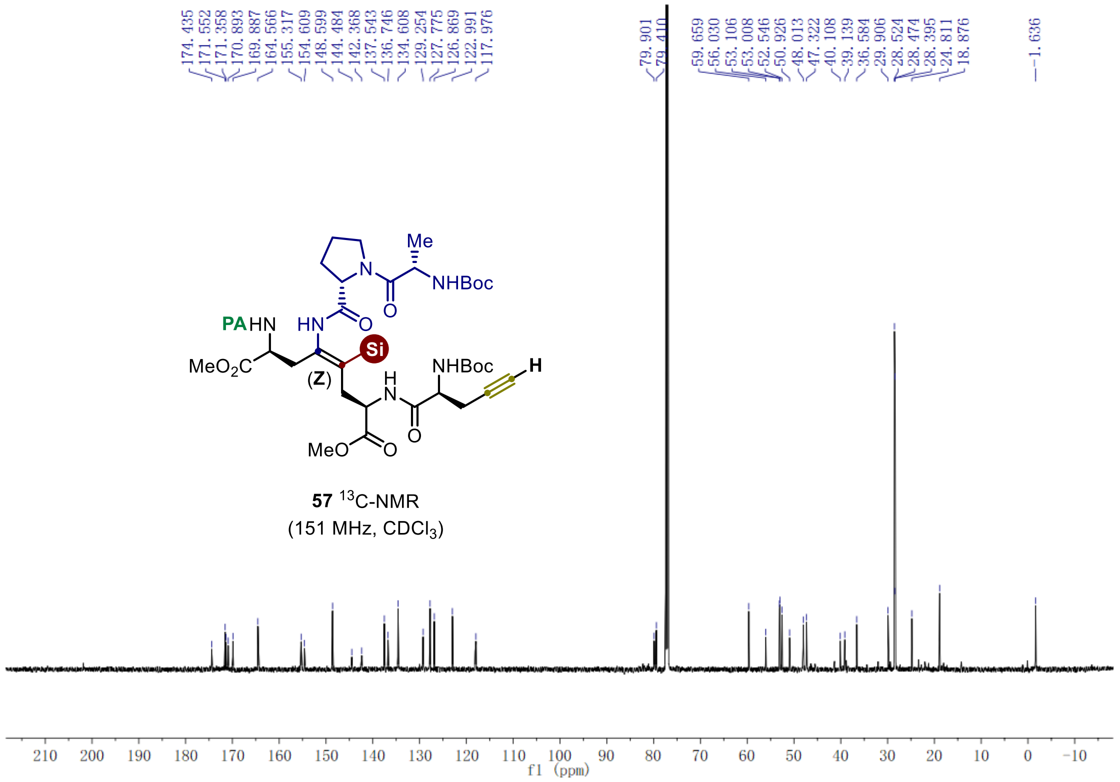
57 ¹³C-NMR
(151 MHz, CDCl3)
PAHN
HN
MeO2C
Si
(Z)
NHBoc
Me
H
MeO
f1 (ppm)

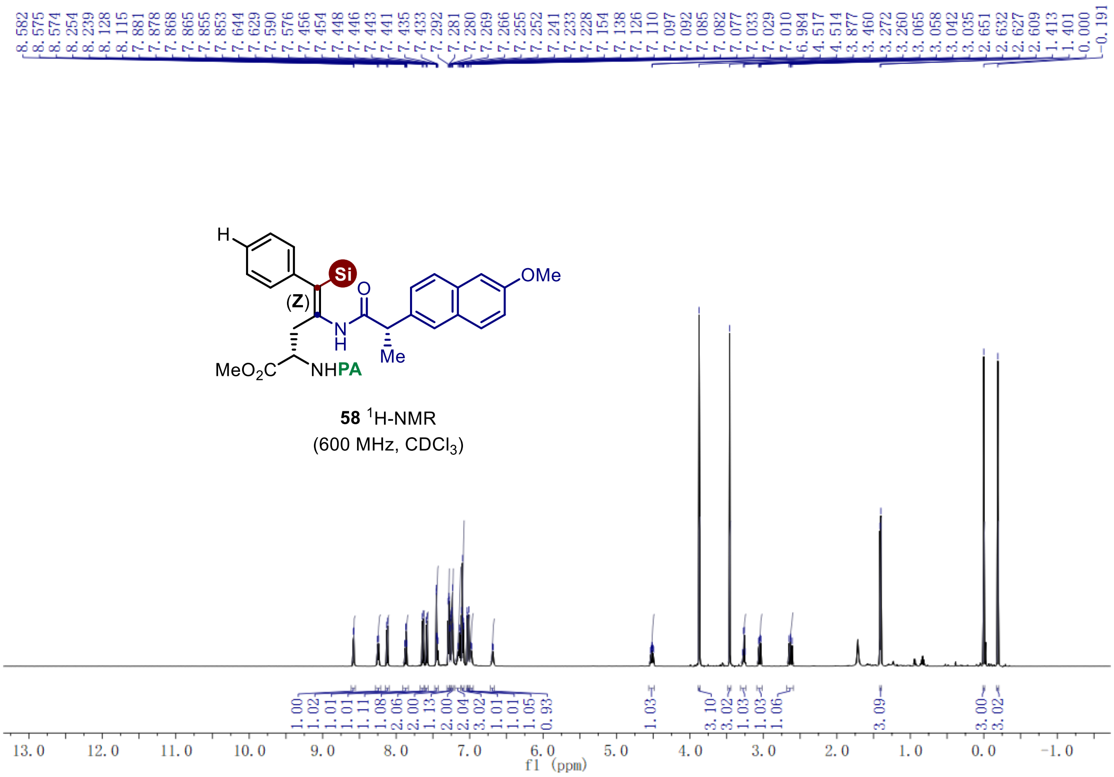

58 ^{1}H-NMR
(600 MHz, $CDCl_3$)

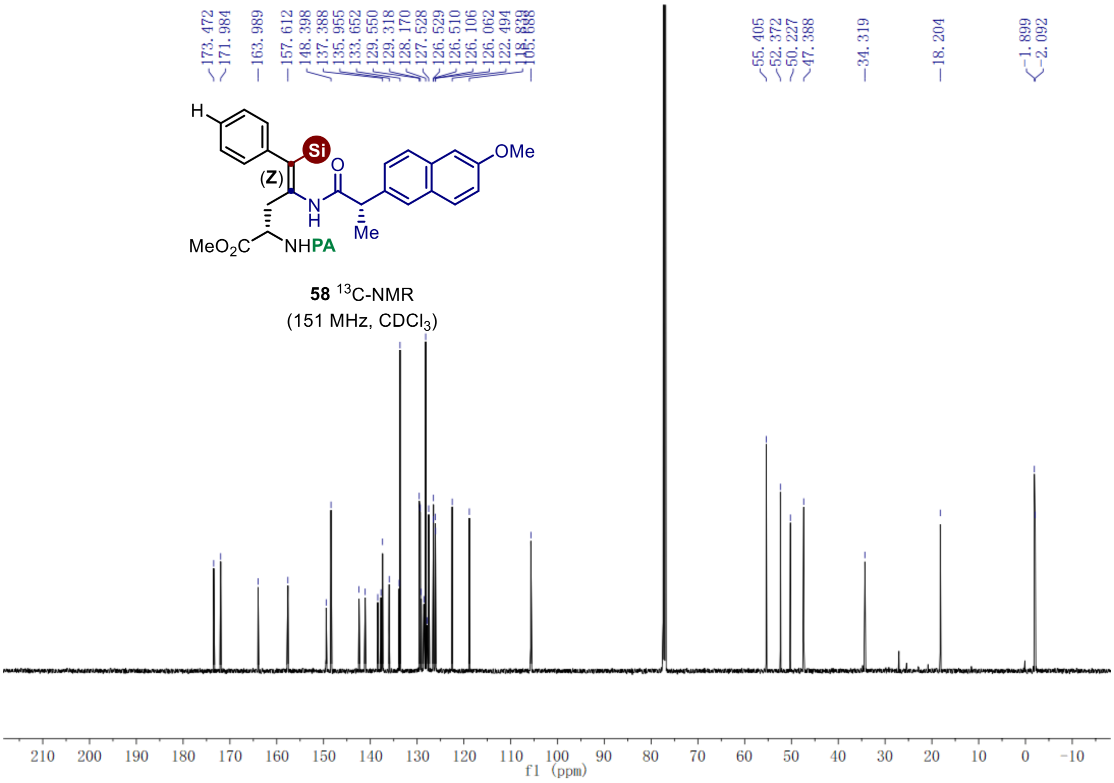

58 ^{13}C-NMR
(151 MHz, $CDCl_3$)

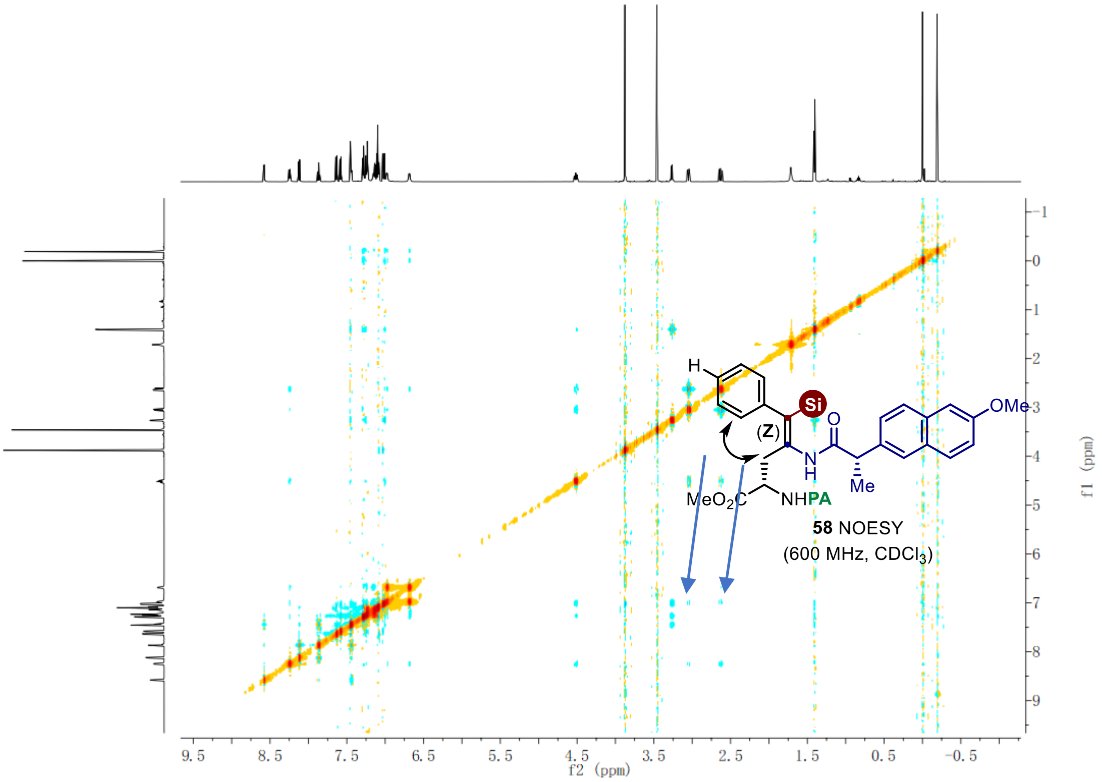

H
Si
(Z)
O
OMe
N
H
Me
MeO2C
NHPA
58 NOESY
(600 MHz, CDCl3)
f2 (ppm)
f1 (ppm)

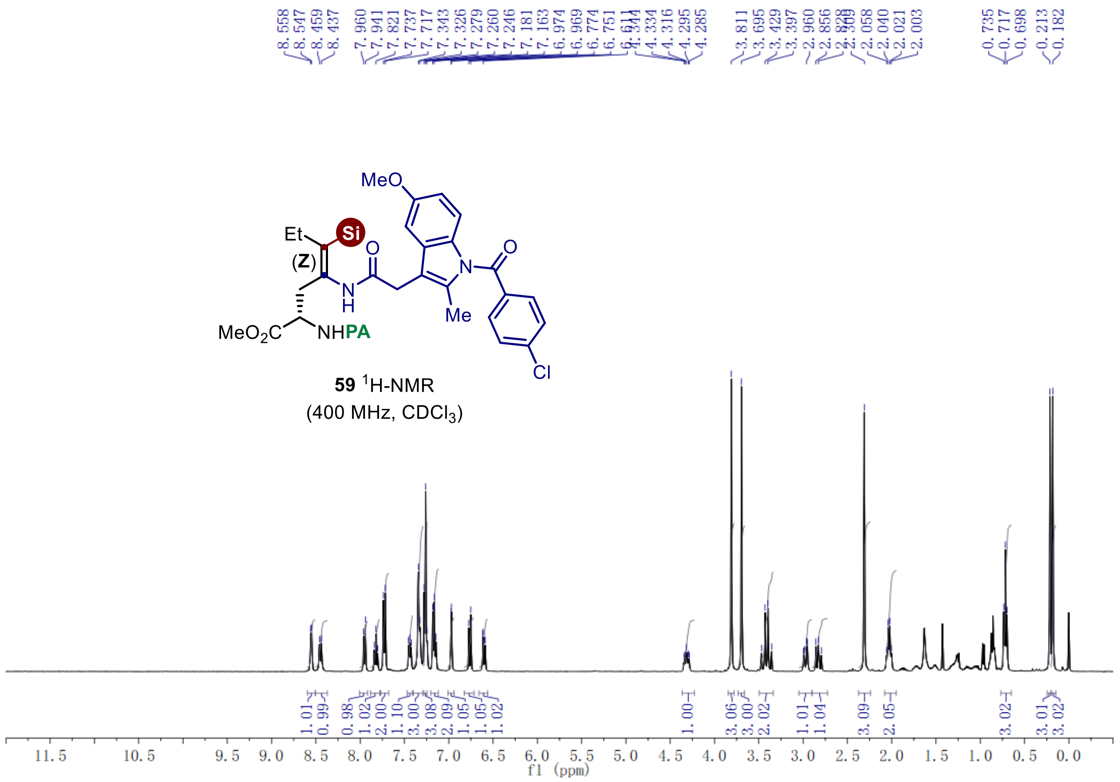
MeO
Et
Si
(Z)
O
N
H
N
O
Me
MeO2C
NHPA
Cl
59 1H-NMR
(400 MHz, CDCl3)
f1 (ppm)

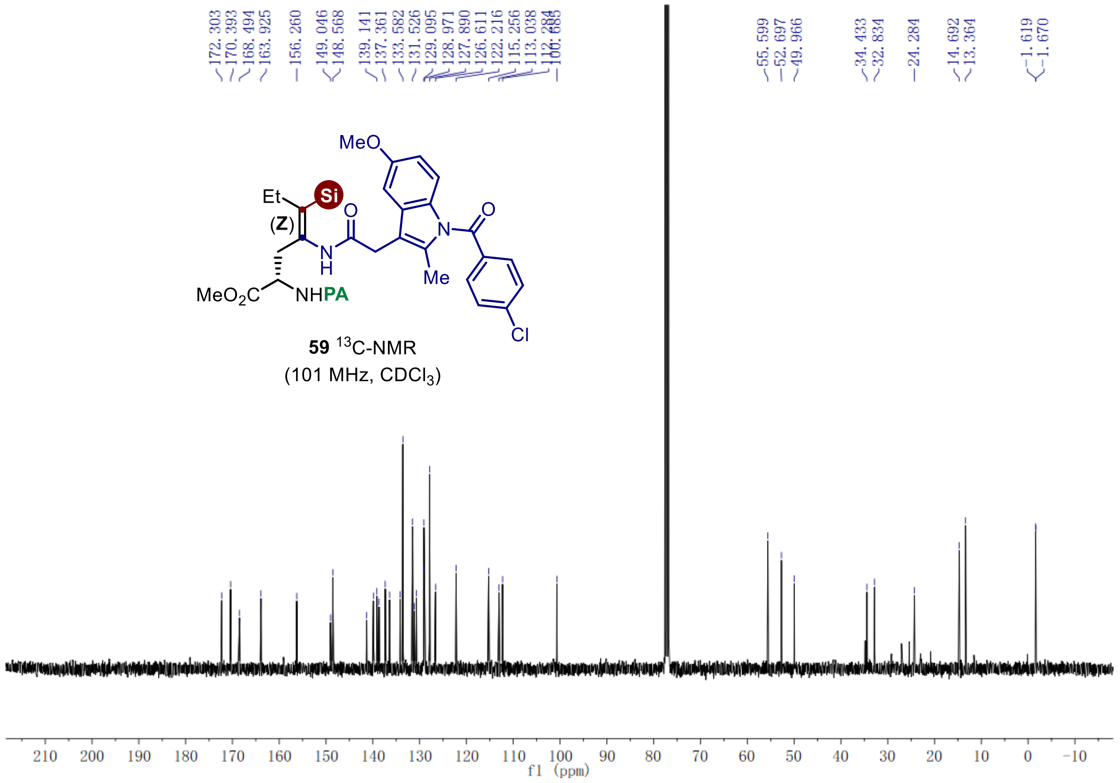
MeO
Et
Si
(Z)
O
N
H
N
O
Me
MeO2C
NHPA
Cl
59 13C-NMR
(101 MHz, CDCl3)
f1 (ppm)

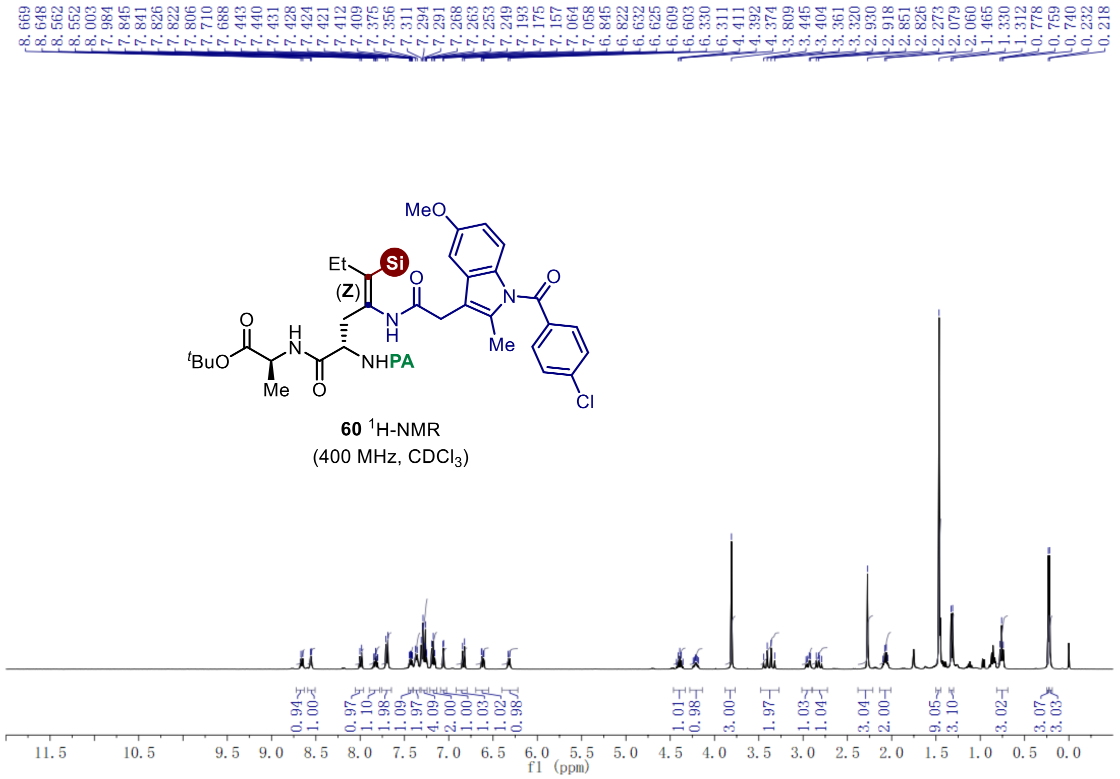
8.669 8.648 8.562 8.552 8.003 7.984 7.845 7.841 7.826 7.822 7.806 7.710 7.688 7.443 7.440 7.431 7.428 7.424 7.421 7.412 7.409 7.375 7.356 7.311 7.294 7.291 7.268 7.263 7.253 7.249 7.193 7.175 7.157 7.064 7.058 6.845 6.822 6.632 6.625 6.609 6.603 6.330 6.311 4.411 4.392 4.374 3.809 3.445 3.404 3.361 3.320 2.930 2.918 2.851 2.826 2.273 2.079 2.060 1.465 1.330 1.312 0.778 0.759 0.740 0.232 0.218
MeO
Et
Si
(Z)
O
N
H
tBuO
Me
NHPA
Cl
60 1H-NMR
(400 MHz, CDCl3)
0.94 1.00 0.97 1.10 1.98 1.09 1.97 4.09 2.00 1.00 1.03 1.02 0.98 1.01 0.98 3.00 1.97 1.03 1.04 3.04 2.00 9.05 3.10 3.02 3.07 3.03
11.5 10.5 9.5 9.0 8.5 8.0 7.5 7.0 6.5 6.0 5.5 5.0 4.5 4.0 3.5 3.0 2.5 2.0 1.5 1.0 0.5 0.0
f1 (ppm)

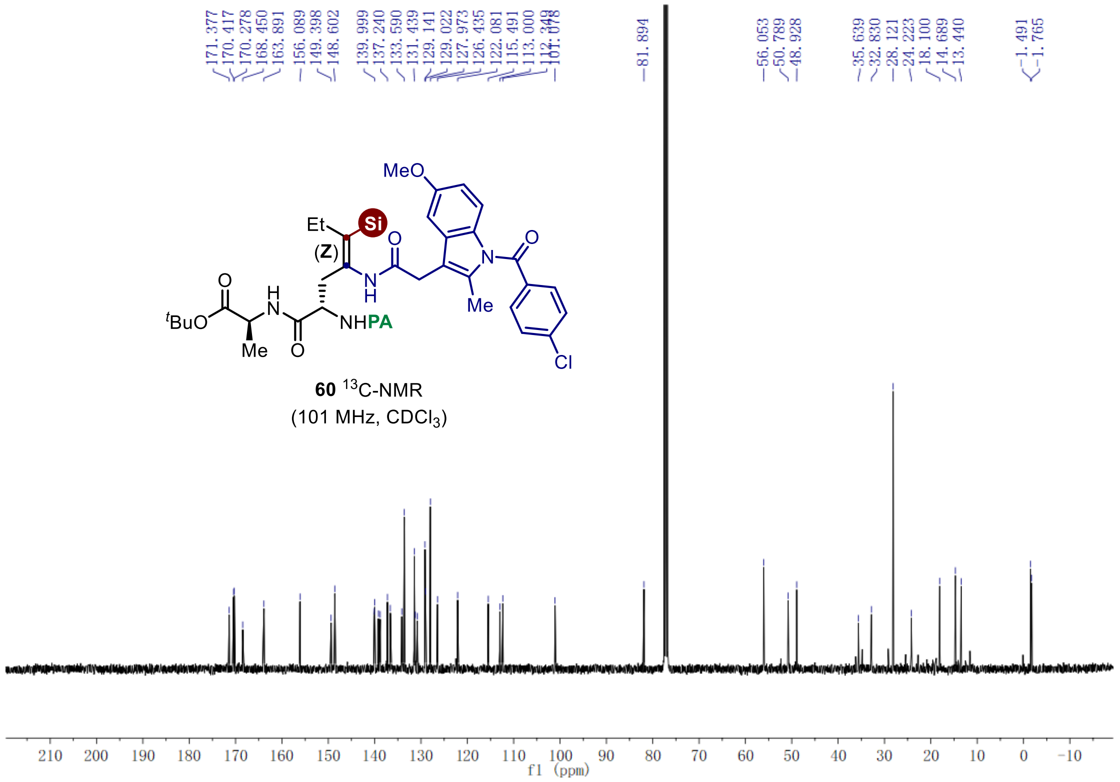
171.377 170.417 170.278 168.450 163.891 156.089 149.398 148.602 139.999 137.240 133.590 131.439 129.141 129.022 127.973 126.435 122.081 115.491 113.000
81.894
56.053 50.789 48.928
35.639 32.830 28.121 24.223 18.100 14.689 13.440
-1.491 -1.765
MeO
Et
Si
(Z)
O
N
H
tBuO
Me
NHPA
Cl
60 13C-NMR
(101 MHz, CDCl3)
210 200 190 180 170 160 150 140 130 120 110 100 90 80 70 60 50 40 30 20 10 0 -10
f1 (ppm)

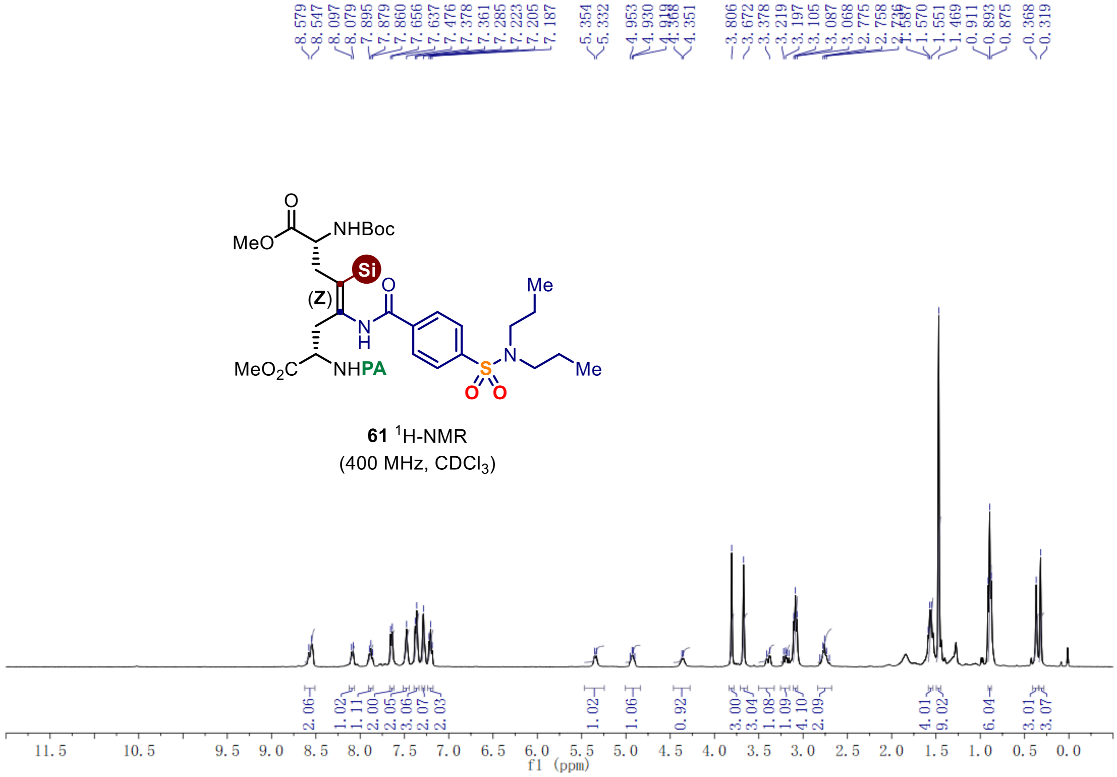

61 ^{1}H-NMR
(400 MHz, $CDCl_3$)

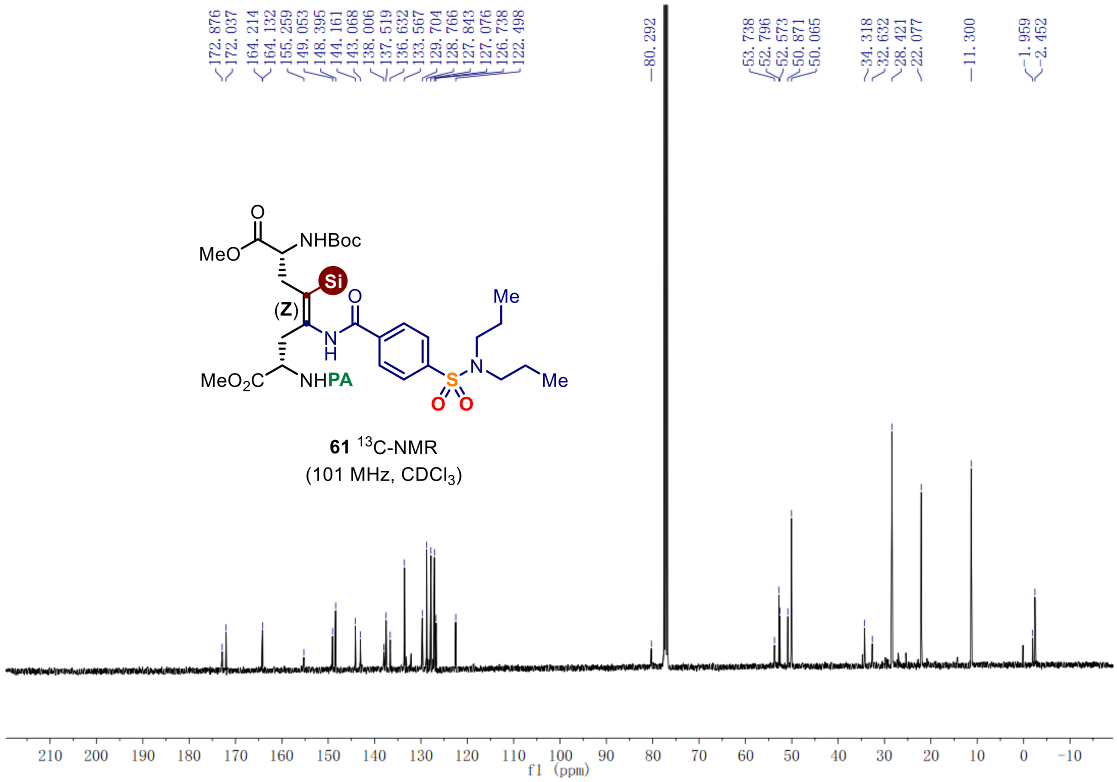

61 ^{13}C-NMR
(101 MHz, $CDCl_3$)

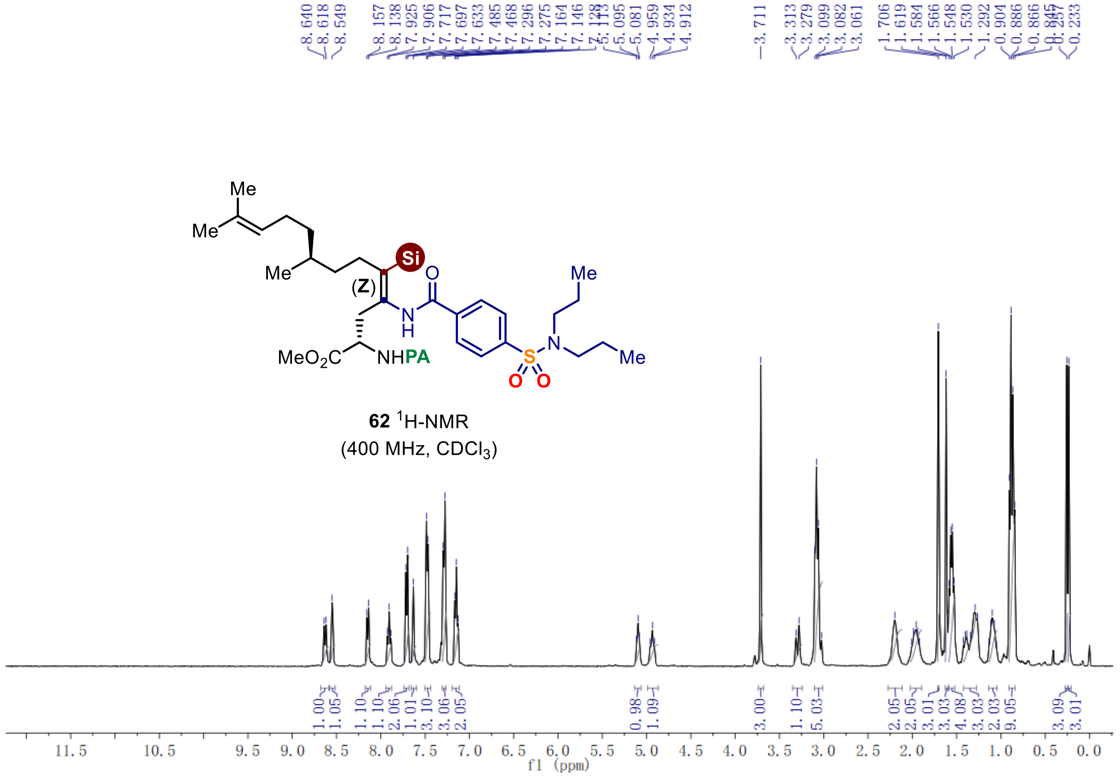

62 ^{1}H-NMR (400 MHz, $CDCl_3$)

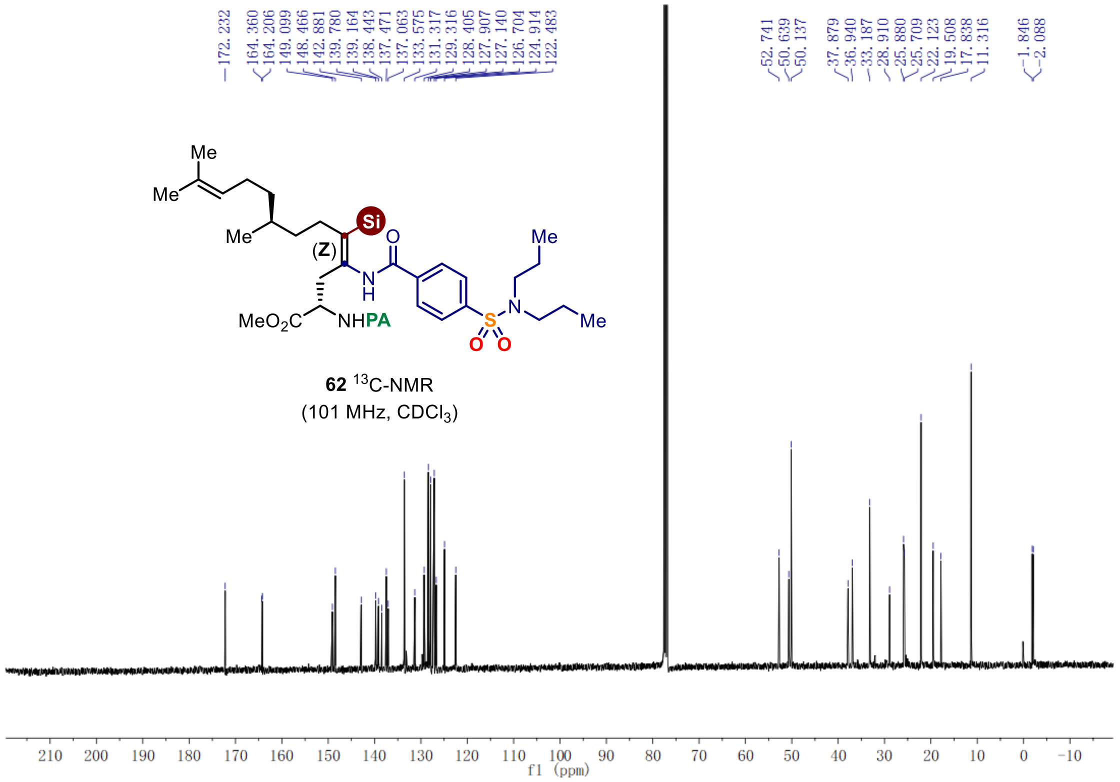

62 ^{13}C-NMR (101 MHz, $CDCl_3$)

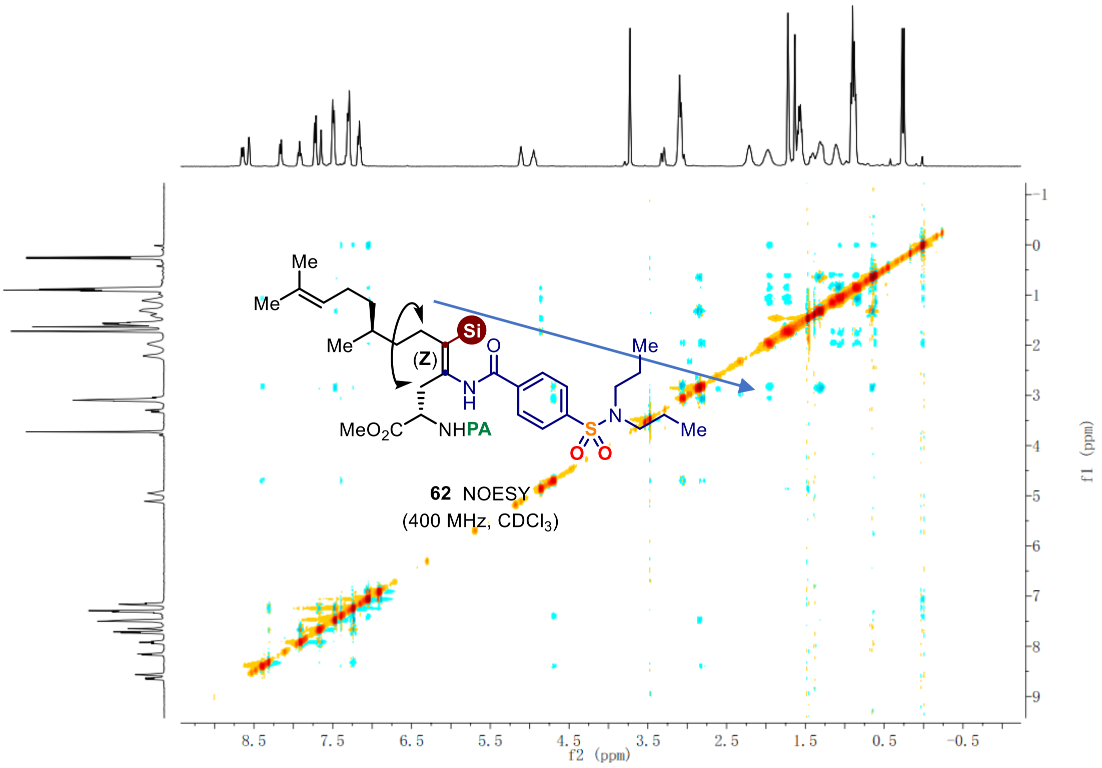

Me
Me
Me
Si
O
(Z)
N
H
MeO2C
NHPA
Me
N
S
O
O
Me
62 NOESY
(400 MHz, CDCl3)
f2 (ppm)
f1 (ppm)

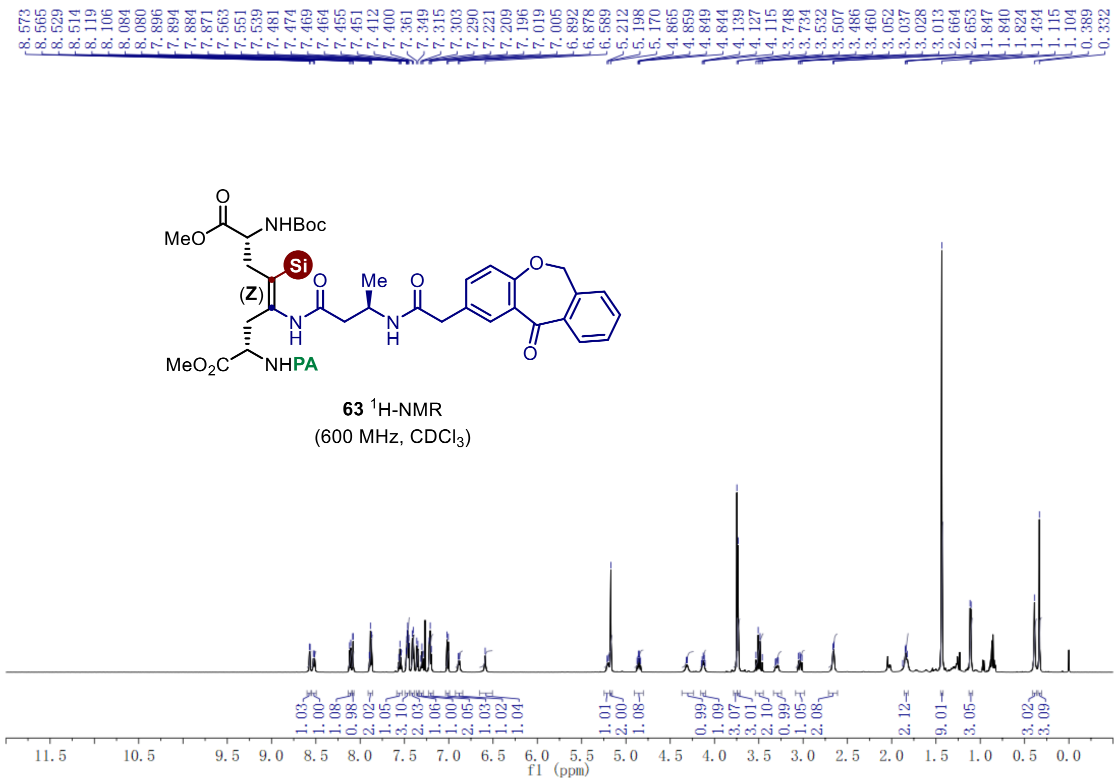

63 ¹H-NMR
(600 MHz, $CDCl_3$)

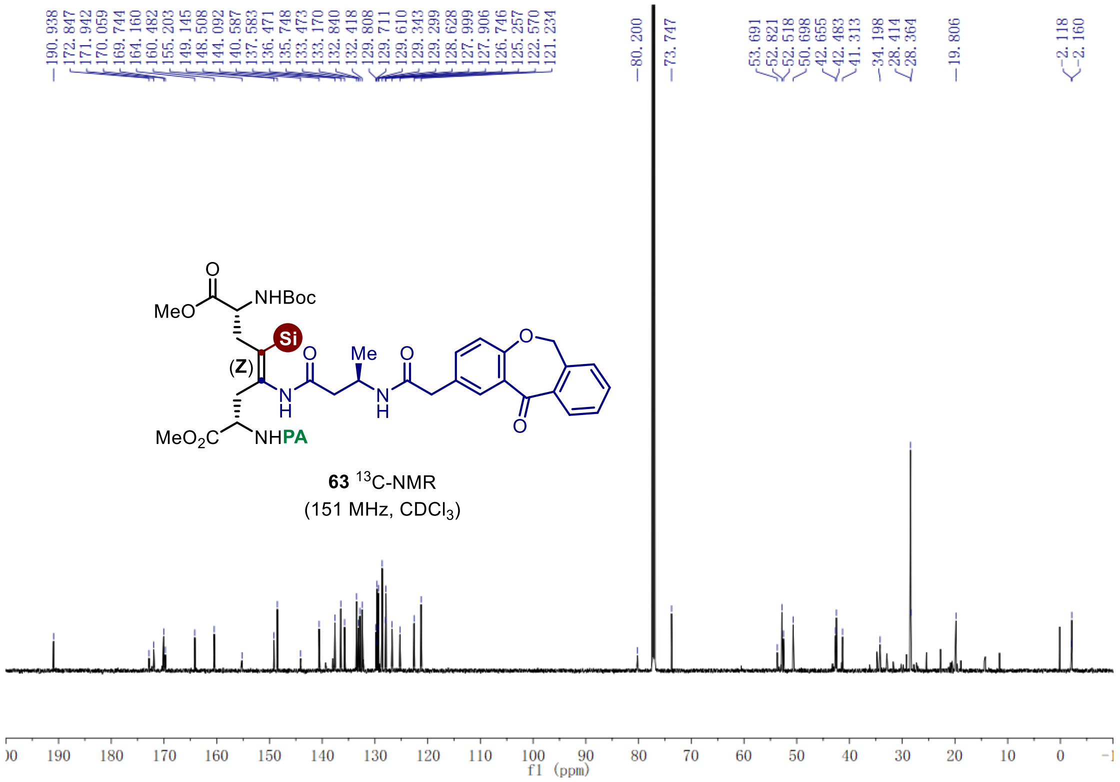

63 ¹³C-NMR
(151 MHz, $CDCl_3$)

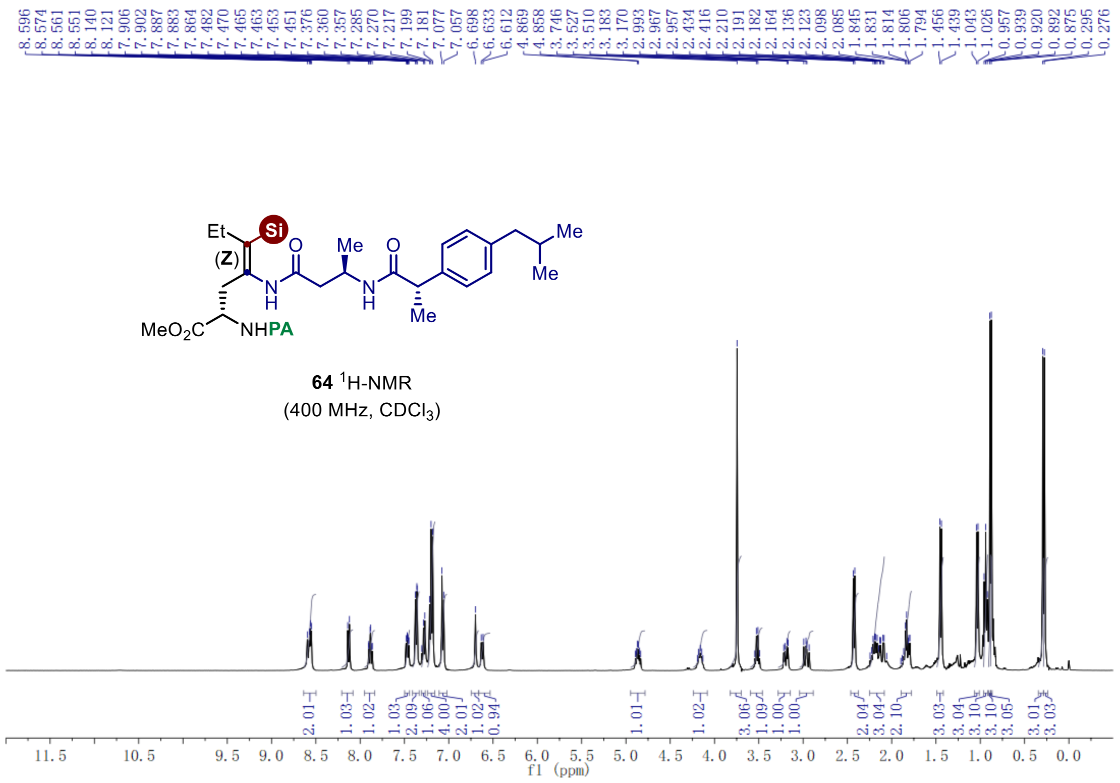

64 ^{1}H-NMR
(400 MHz, $CDCl_3$)

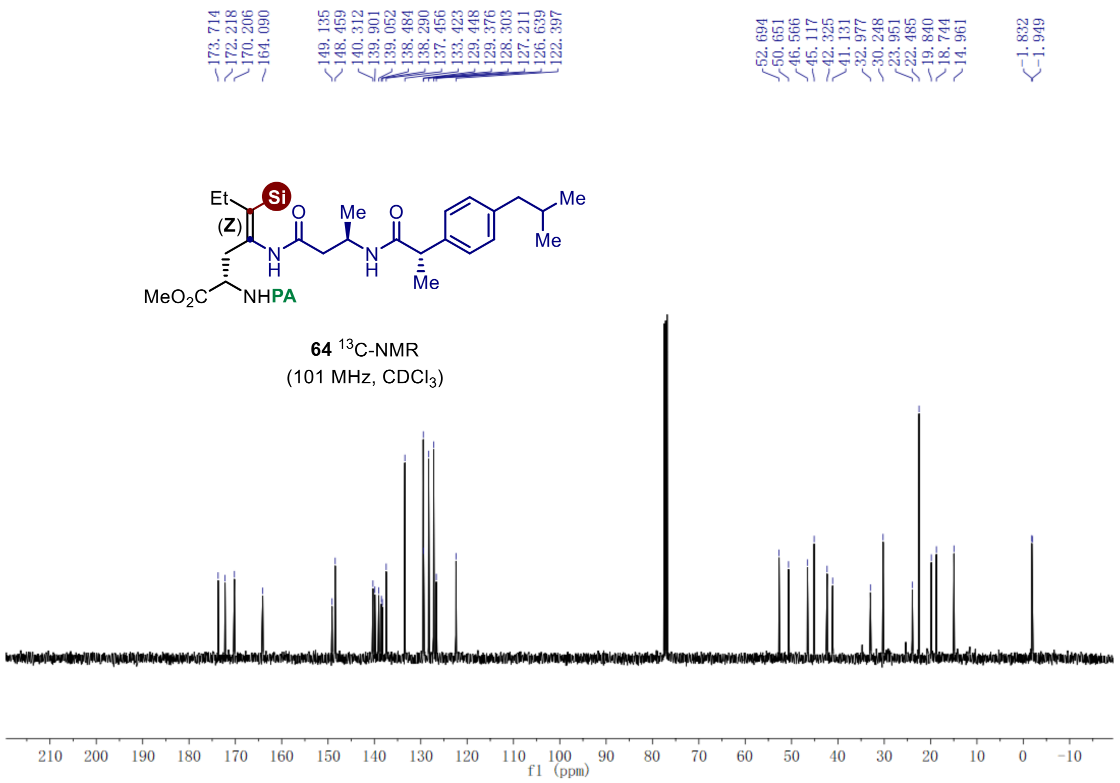

64 ^{13}C-NMR
(101 MHz, $CDCl_3$)

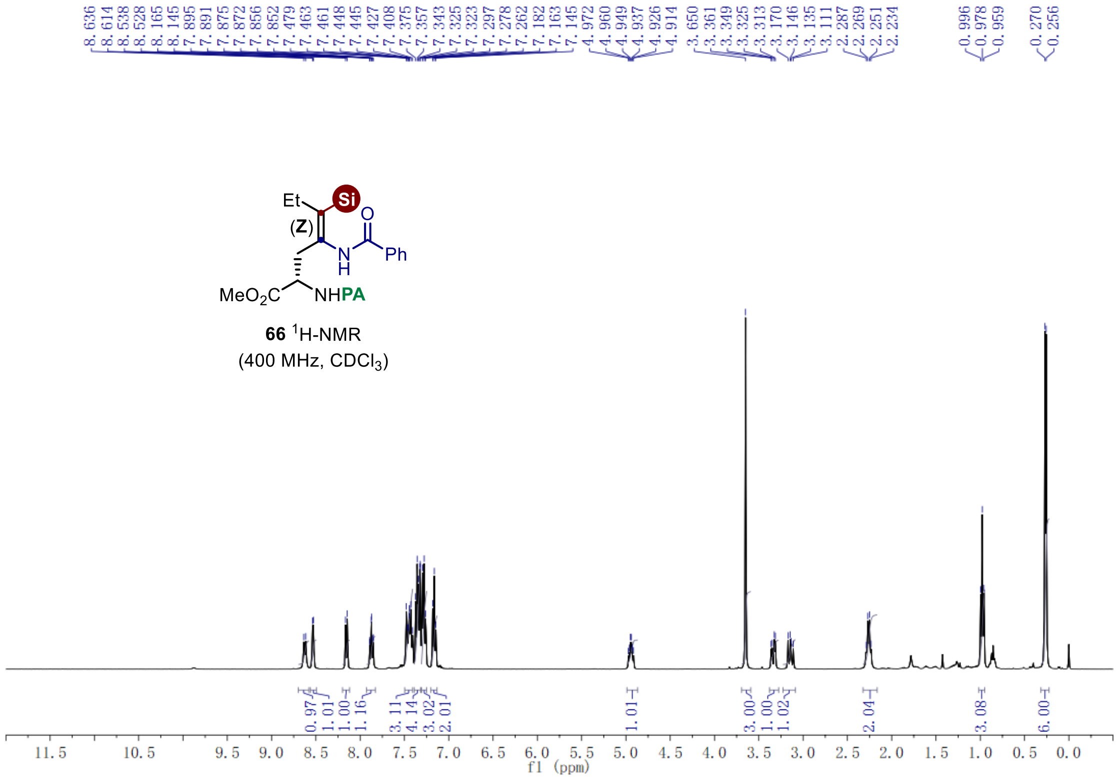
8.636 8.614 8.538 8.528 8.165 8.145 7.895 7.891 7.875 7.872 7.856 7.852 7.479 7.463 7.461 7.448 7.445 7.427 7.408 7.375 7.357 7.343 7.325 7.323 7.297 7.278 7.262 7.182 7.163 7.145 4.972 4.960 4.949 4.937 4.926 4.914 3.650 3.361 3.349 3.325 3.313 3.170 3.146 3.135 3.111 2.287 2.269 2.251 2.234 0.996 0.978 0.959 0.270 0.256
Et
Si
(Z)
O
N
H
Ph
MeO2C
NHPA
66 1H-NMR
(400 MHz, CDCl3)
0.97 1.01 1.00 1.16 3.11 4.14 3.02 2.01 1.01 3.00 1.00 1.02 2.04 3.08 6.00
11.5 10.5 9.5 9.0 8.5 8.0 7.5 7.0 6.5 6.0 5.5 5.0 4.5 4.0 3.5 3.0 2.5 2.0 1.5 1.0 0.5 0.0
f1 (ppm)

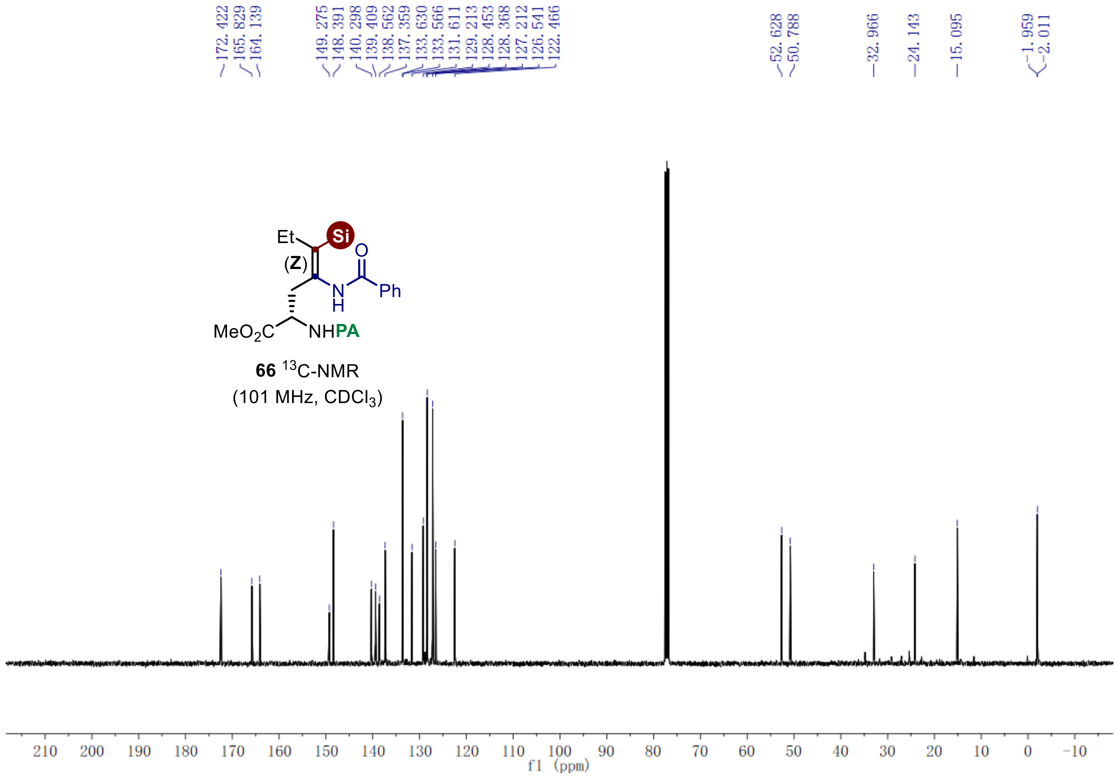
172.422 165.829 164.139 149.275 148.391 140.298 139.409 138.562 137.359 133.630 133.566 131.611 129.213 128.453 128.368 127.212 126.541 122.466 52.628 50.788 32.966 24.143 15.095 -1.959 -2.011
Et
Si
(Z)
O
N
H
Ph
MeO2C
NHPA
66 13C-NMR
(101 MHz, CDCl3)
210 200 190 180 170 160 150 140 130 120 110 100 90 80 70 60 50 40 30 20 10 0 -10
f1 (ppm)

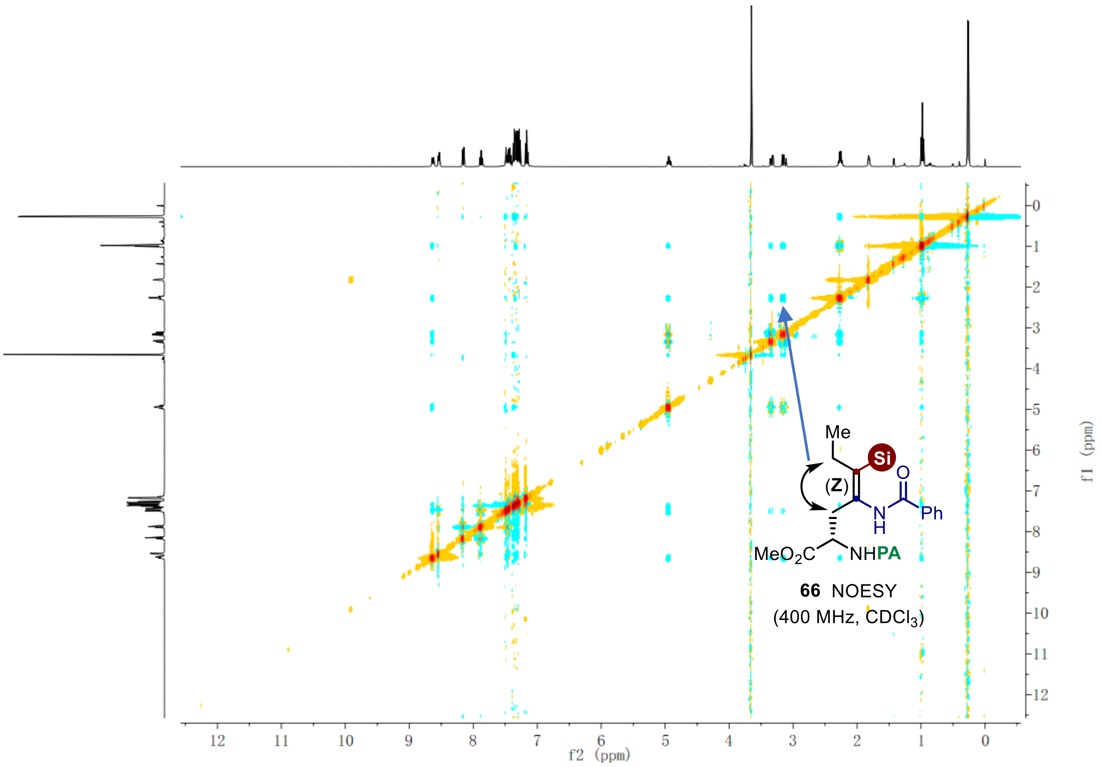

Me
Si
(Z)
O
N
H
Ph
MeO2C
NHPA
66 NOESY
(400 MHz, CDCl3)
f2 (ppm)
f1 (ppm)

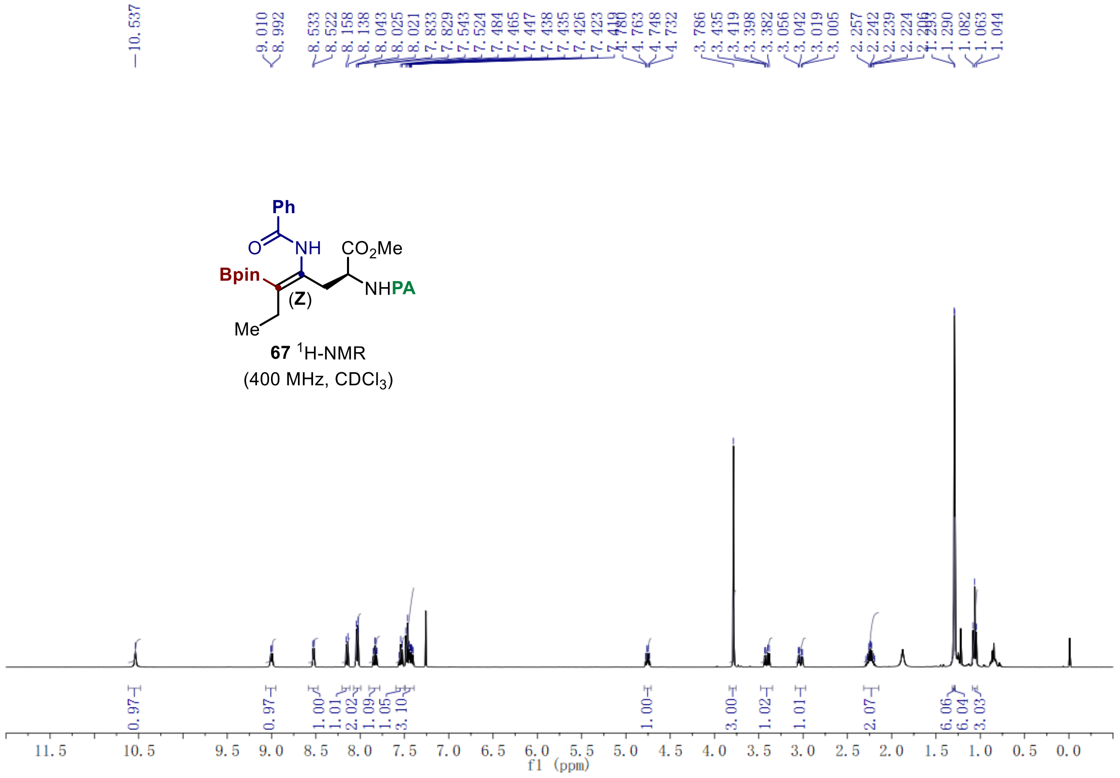
67 ^{1}H-NMR
(400 MHz, $CDCl_3$)

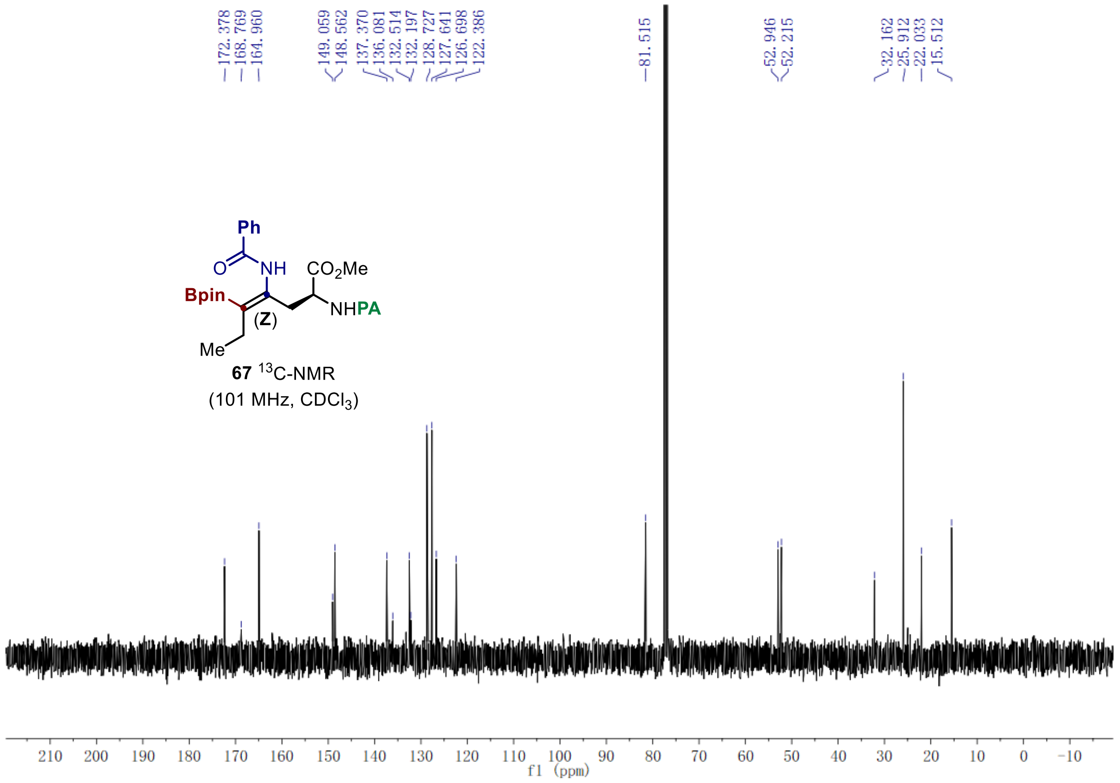
67 ^{13}C-NMR
(101 MHz, $CDCl_3$)

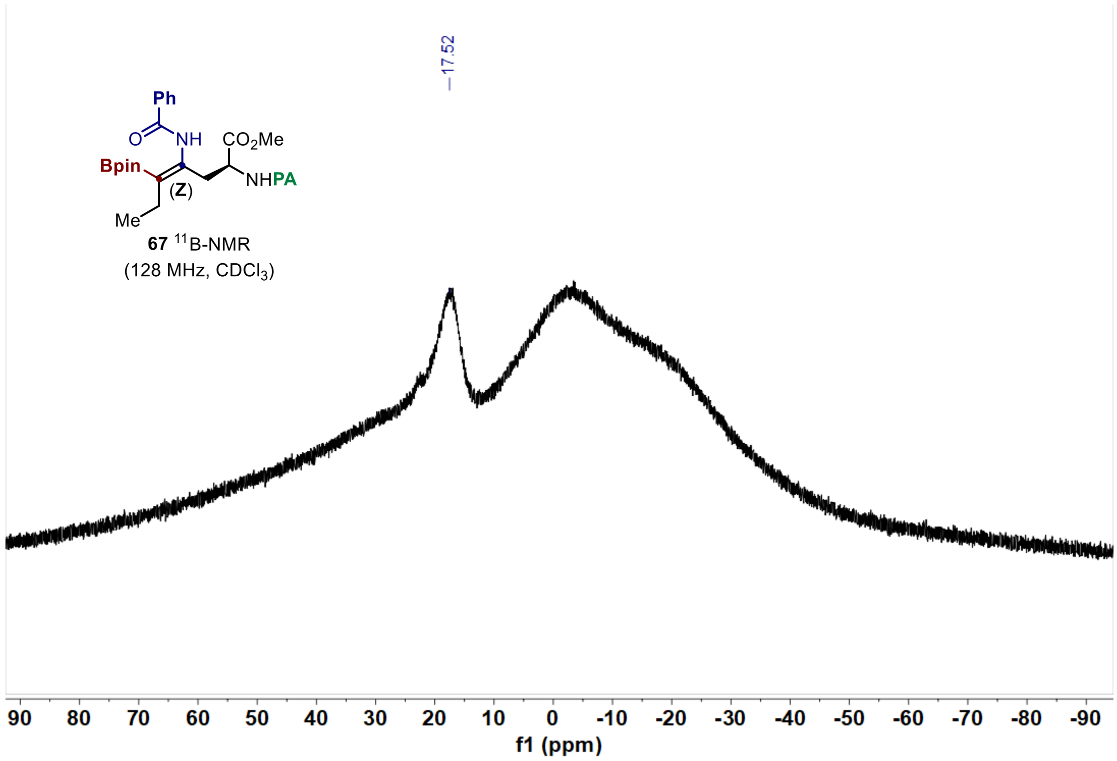

17.52
Ph
O
NH
CO2Me
Bpin
(Z)
NHPA
Me
67 11B-NMR
(128 MHz, CDCl3)
90 80 70 60 50 40 30 20 10 0 -10 -20 -30 -40 -50 -60 -70 -80 -90
f1 (ppm)

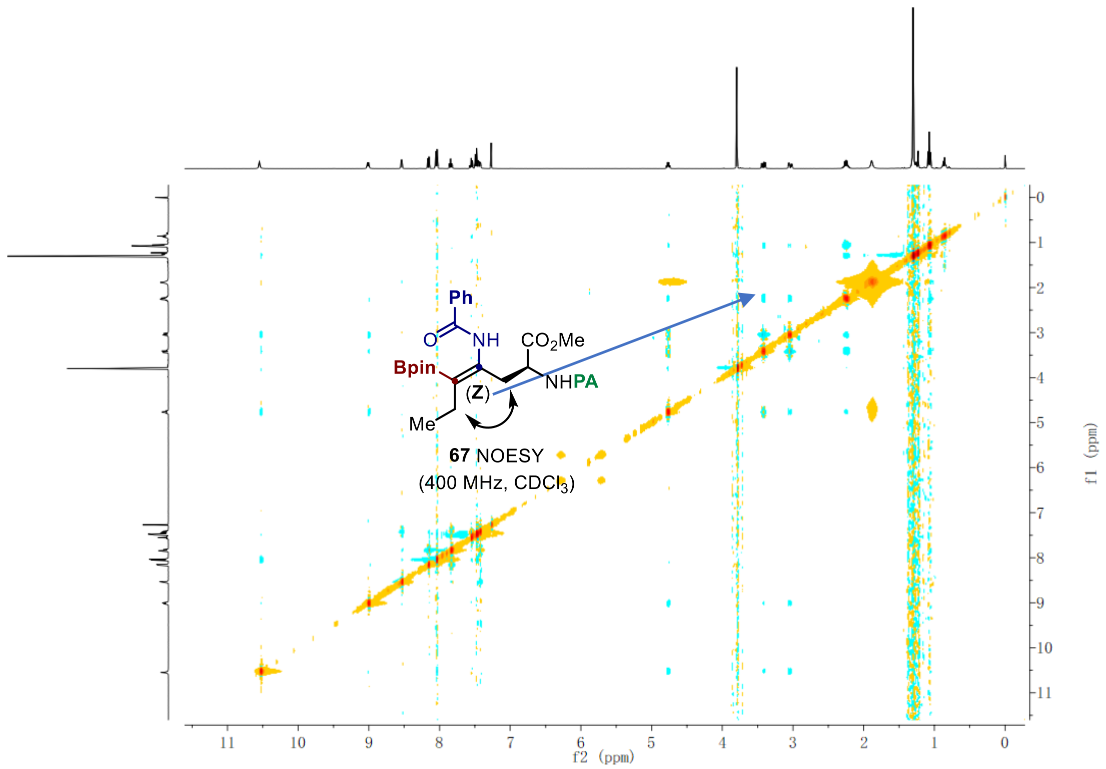

Ph
O
NH
CO2Me
Bpin
(Z)
NHPA
Me
67 NOESY
(400 MHz, CDCl3)
11 10 9 8 7 6 5 4 3 2 1 0
f2 (ppm)
f1 (ppm)

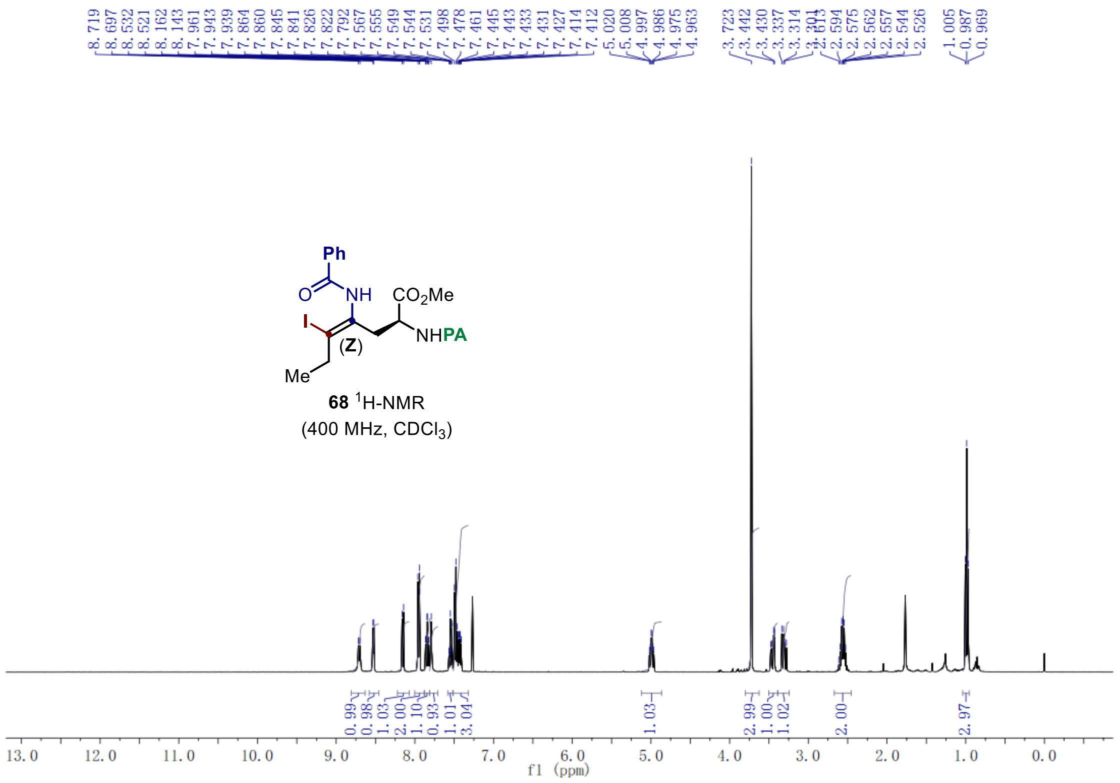
68 1H-NMR
(400 MHz, CDCl3)
f1 (ppm)

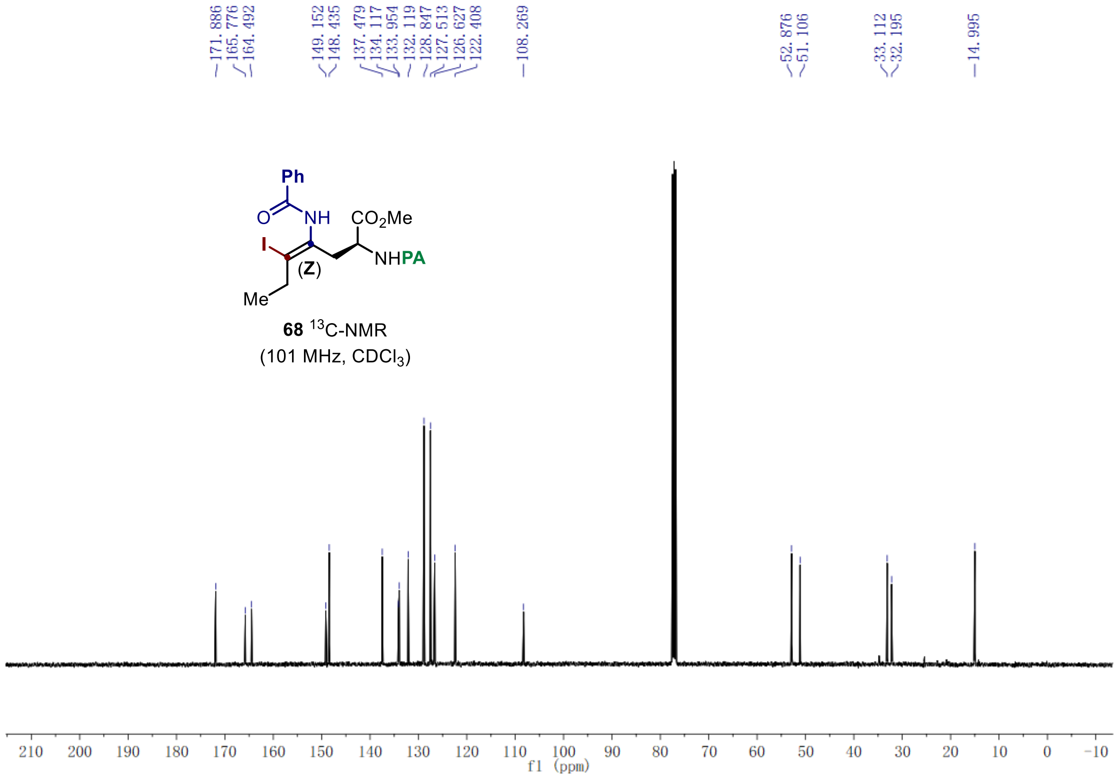
68 13C-NMR
(101 MHz, CDCl3)
f1 (ppm)

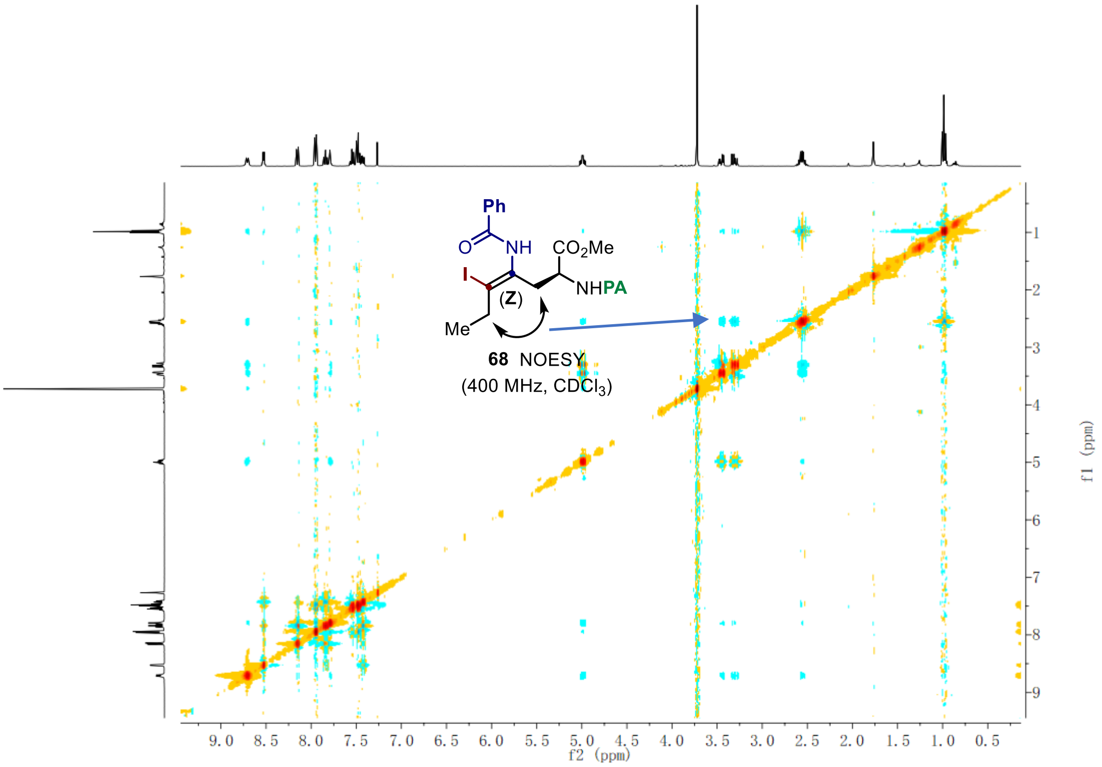
Ph
O
NH
CO2Me
I
(Z)
NHPA
Me
68 NOESY
(400 MHz, CDCl3)
f2 (ppm)
f1 (ppm)

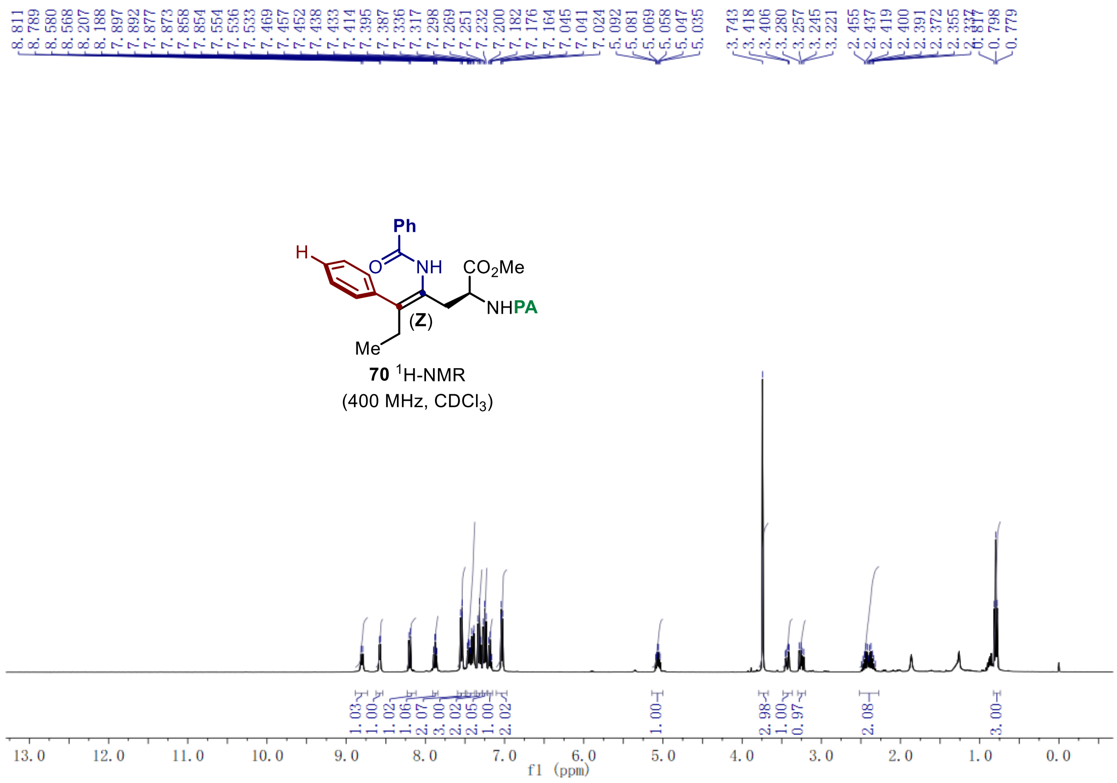
8.811 8.789 8.580 8.568 8.207 8.188 7.897 7.892 7.877 7.873 7.858 7.854 7.554 7.536 7.533 7.469 7.457 7.452 7.438 7.433 7.414 7.395 7.387 7.336 7.317 7.298 7.269 7.251 7.232 7.200 7.182 7.176 7.164 7.045 7.041 7.024 5.092 5.081 5.069 5.058 5.047 5.035 3.743 3.418 3.406 3.280 3.257 3.245 3.221 2.455 2.437 2.419 2.400 2.391 2.372 2.355 2.337 0.817 0.798 0.779
Ph
H
O
NH
CO2Me
NHPA
(Z)
Me
70 1H-NMR
(400 MHz, CDCl3)
1.03 1.00 1.02 1.06 2.07 3.00 2.02 2.05 1.00 2.02 1.00 2.98 1.00 0.97 2.08 3.00
f1 (ppm)

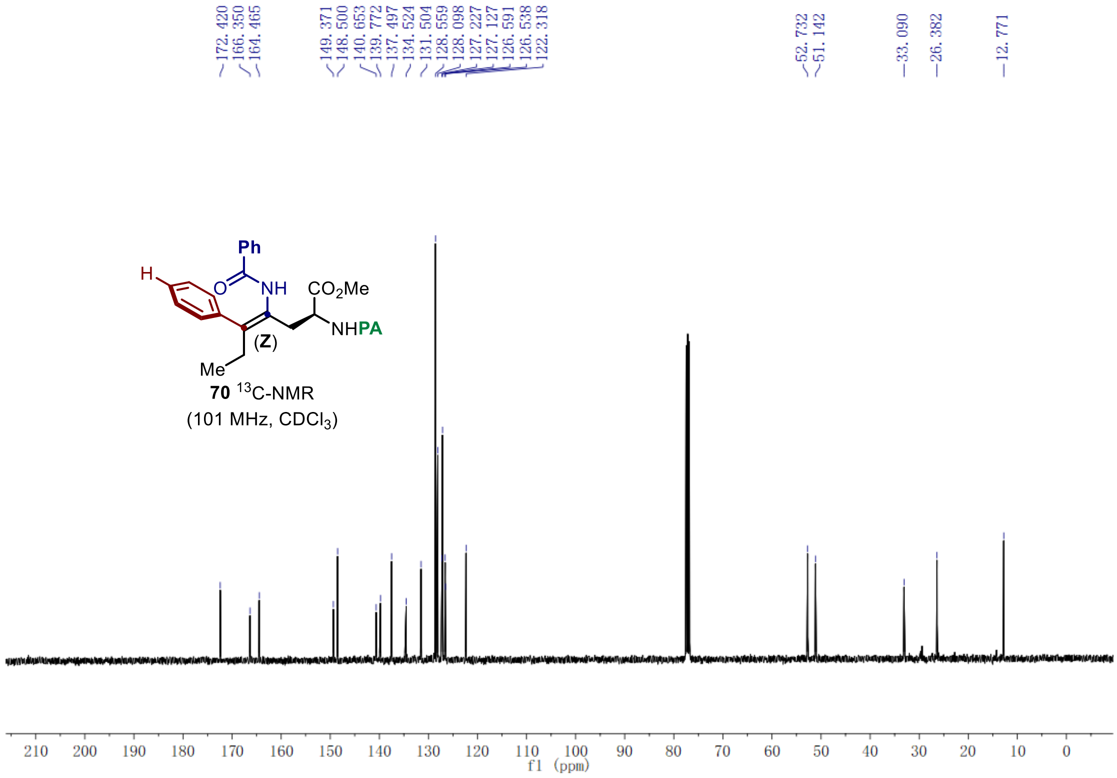
172.420 166.350 164.465 149.371 148.500 140.653 139.772 137.497 134.524 131.504 128.559 128.098 127.227 127.127 126.591 126.538 122.318 52.732 51.142 33.090 26.382 12.771
Ph
H
O
NH
CO2Me
NHPA
(Z)
Me
70 13C-NMR
(101 MHz, CDCl3)
f1 (ppm)

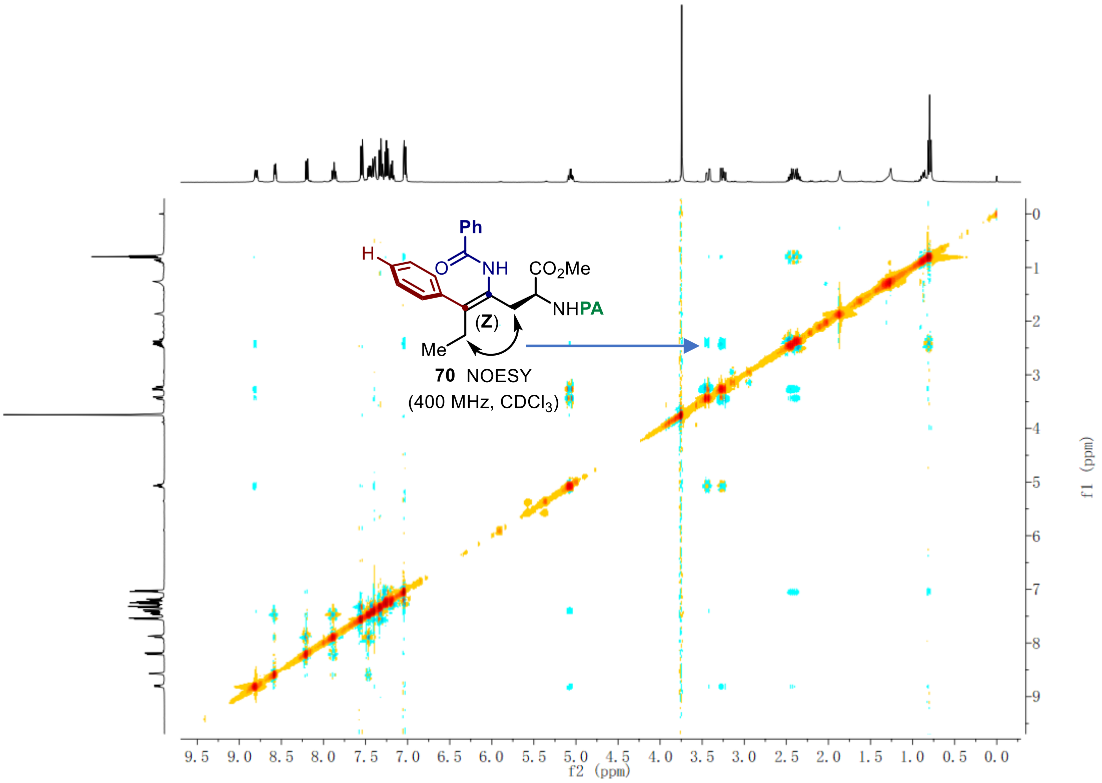

Ph
H
O
NH
CO2Me
NHPA
(Z)
Me
70 NOESY
(400 MHz, CDCl3)
f2 (ppm)
f1 (ppm)

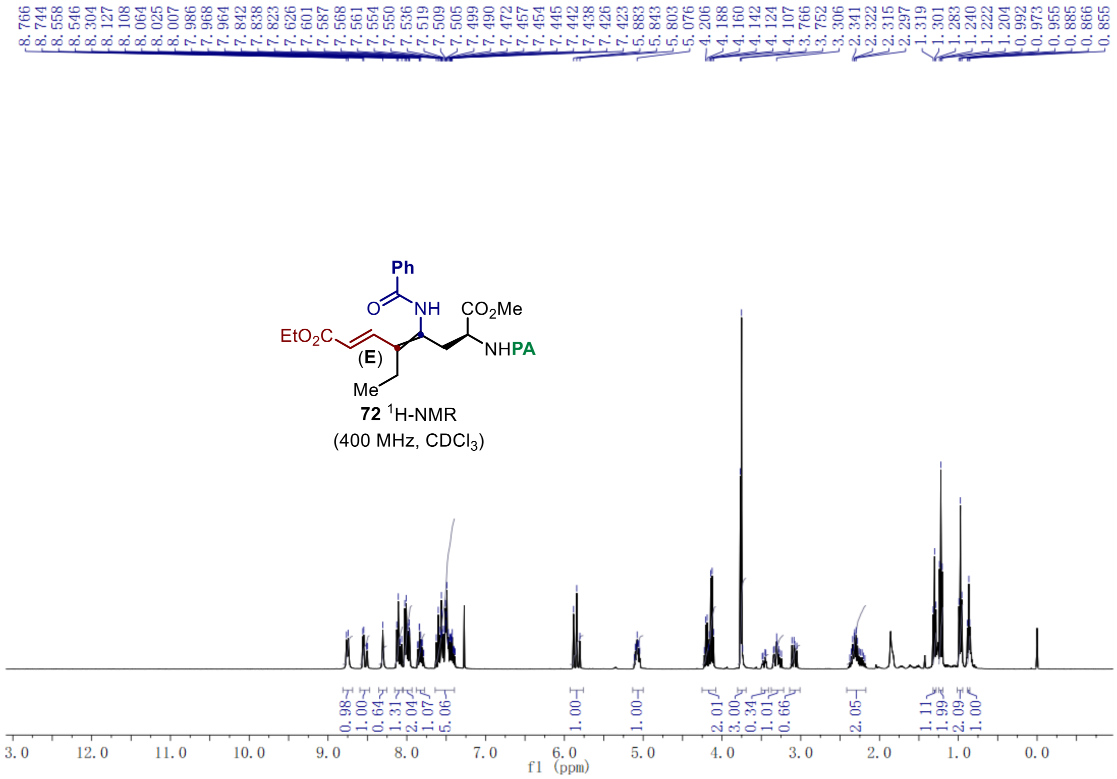

8.766
8.744
8.558
8.546
8.304
8.127
8.108
8.064
8.025
8.007
7.986
7.968
7.964
7.842
7.838
7.823
7.626
7.601
7.587
7.568
7.561
7.554
7.550
7.536
7.519
7.509
7.505
7.499
7.490
7.472
7.457
7.454
7.445
7.442
7.438
7.426
7.423
5.883
5.843
5.803
5.076
4.206
4.188
4.160
4.142
4.124
4.107
3.766
3.752
3.306
2.341
2.322
2.315
2.297
1.319
1.301
1.283
1.240
1.222
1.204
0.992
0.973
0.955
0.885
0.866
0.855
Ph
O
NH
CO2Me
EtO2C
(E)
NHPA
Me
72 1H-NMR
(400 MHz, CDCl3)
0.98
1.00
0.64
1.31
2.04
1.07
5.06
1.00
1.00
2.01
3.00
0.34
1.01
0.66
2.05
1.11
1.99
2.09
1.00
3.0
12.0
11.0
10.0
9.0
8.0
7.0
6.0
5.0
4.0
3.0
2.0
1.0
0.0
f1 (ppm)

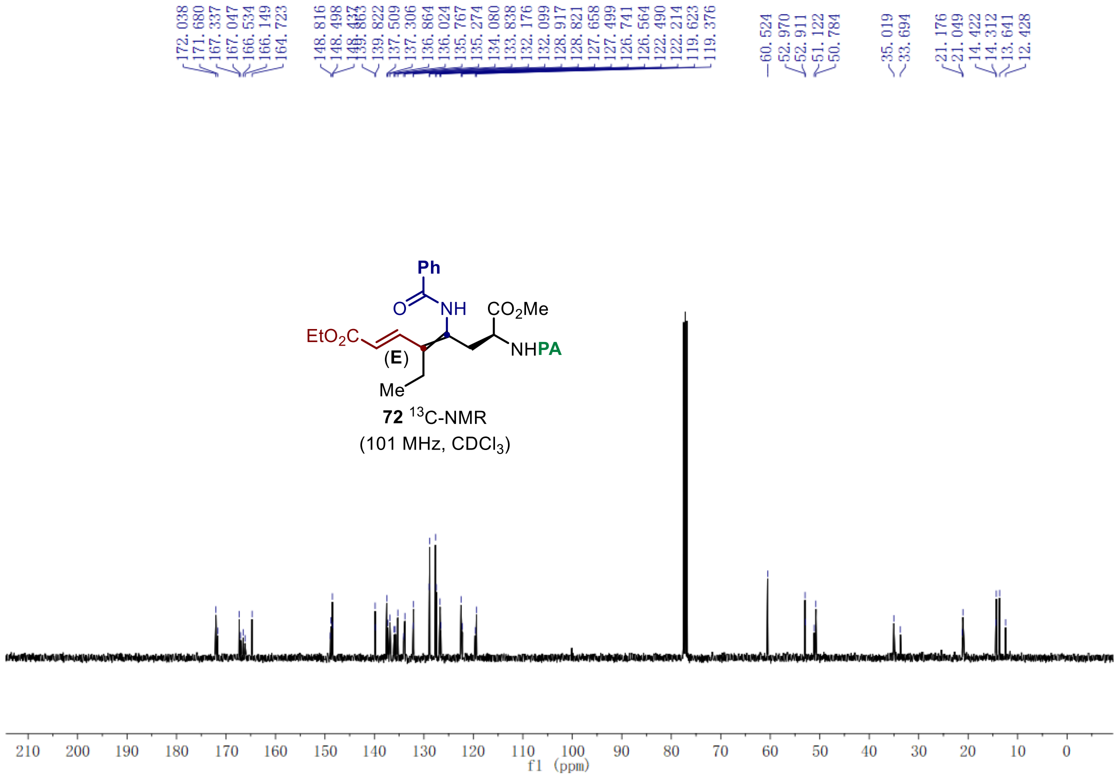

172.038
171.680
167.337
167.047
166.534
166.149
164.723
148.816
148.498
139.822
137.509
137.306
136.864
136.024
135.767
135.274
134.080
133.838
132.176
132.099
128.917
128.821
127.658
127.499
126.741
126.564
122.490
122.214
119.623
119.376
60.524
52.970
52.911
51.122
50.784
35.019
33.694
21.176
21.049
14.422
14.312
13.641
12.428
Ph
O
NH
CO2Me
EtO2C
(E)
NHPA
Me
72 13C-NMR
(101 MHz, CDCl3)
210
200
190
180
170
160
150
140
130
120
110
100
90
80
70
60
50
40
30
20
10
0
f1 (ppm)

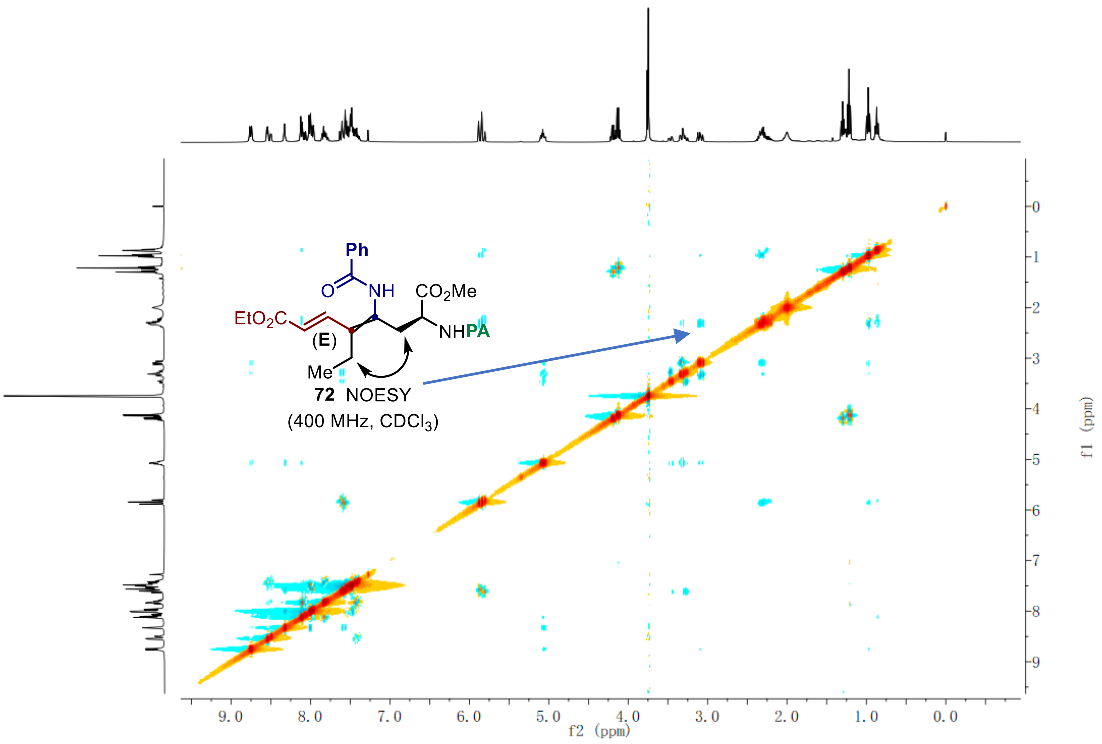
Ph
O
NH
CO2Me
EtO2C
NHPA
(E)
Me
72 NOESY
(400 MHz, CDCl3)
f2 (ppm)
f1 (ppm)

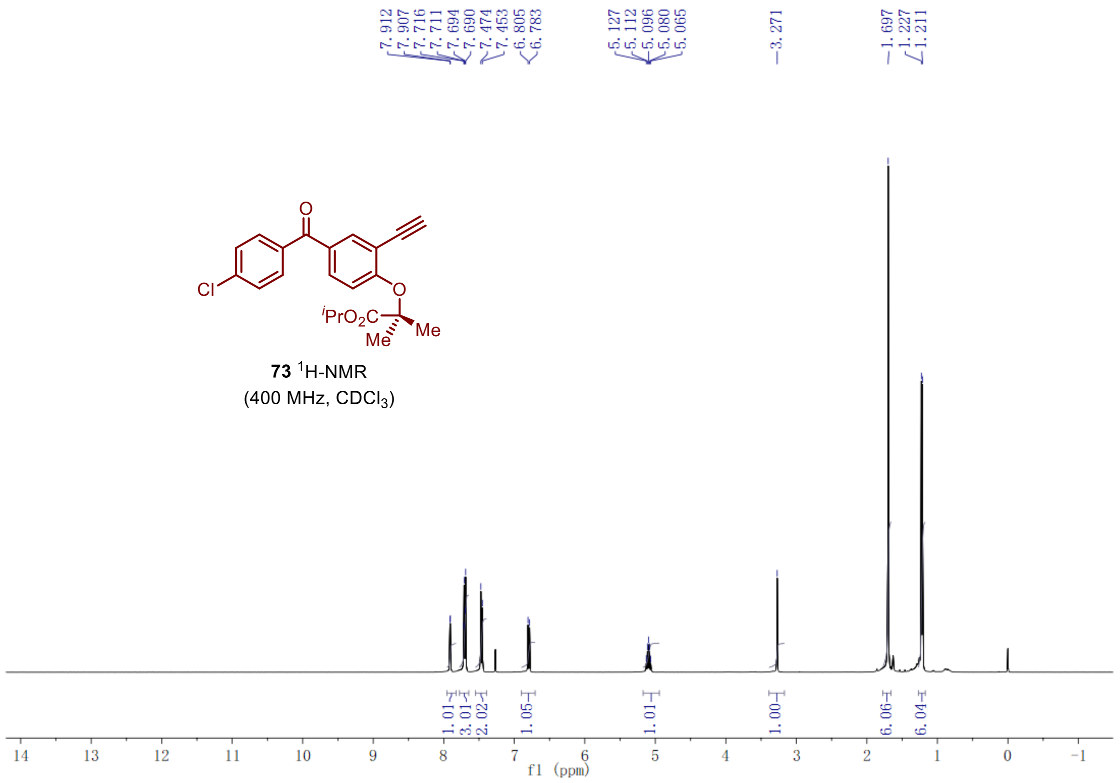
7.912
7.907
7.716
7.711
7.694
7.690
7.474
7.453
6.805
6.783
5.127
5.112
5.096
5.080
5.065
3.271
1.697
1.227
1.211
Cl
O
^{i}PrO$_2$C
Me
Me
73 ^{1}H-NMR
(400 MHz, CDCl$_3$)
1.01
3.01
2.02
1.05
1.01
1.00
6.06
6.04
f1 (ppm)

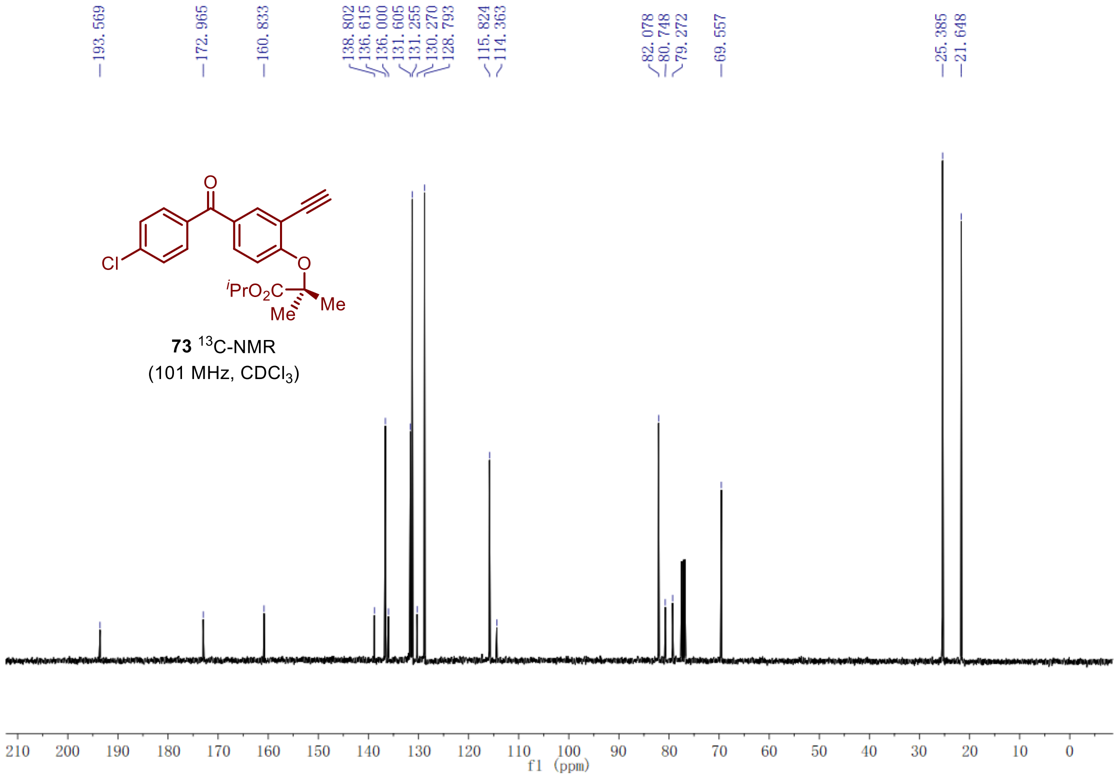
193.569
172.965
160.833
138.802
136.615
136.000
131.605
131.255
130.270
128.793
115.824
114.363
82.078
80.748
79.272
69.557
25.385
21.648
Cl
O
^{i}PrO$_2$C
Me
Me
73 ^{13}C-NMR
(101 MHz, CDCl$_3$)
f1 (ppm)

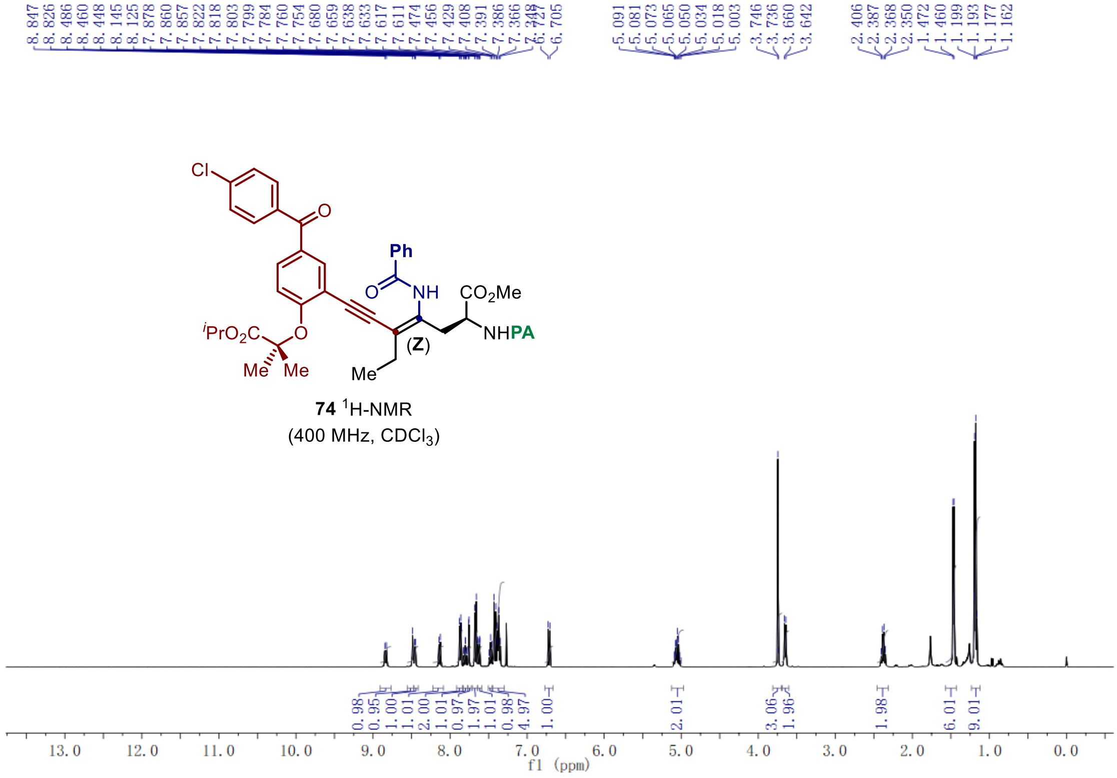

74 ^{1}H-NMR
(400 MHz, $CDCl_3$)

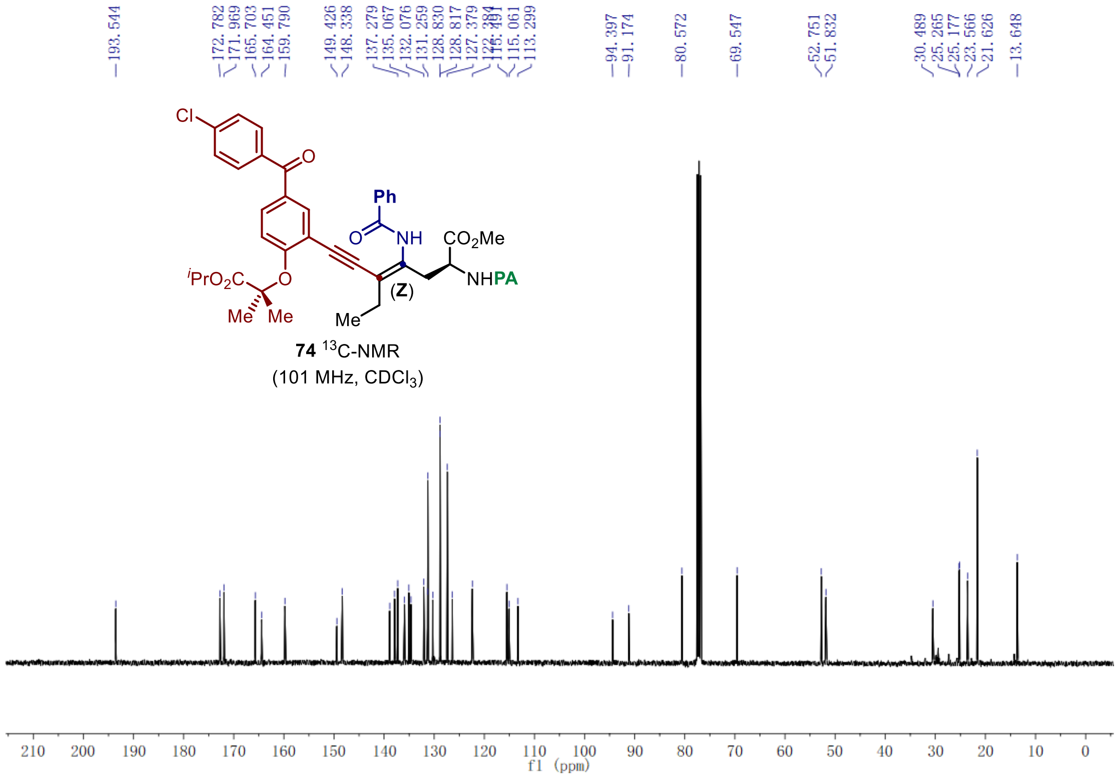

74 ^{13}C-NMR
(101 MHz, $CDCl_3$)

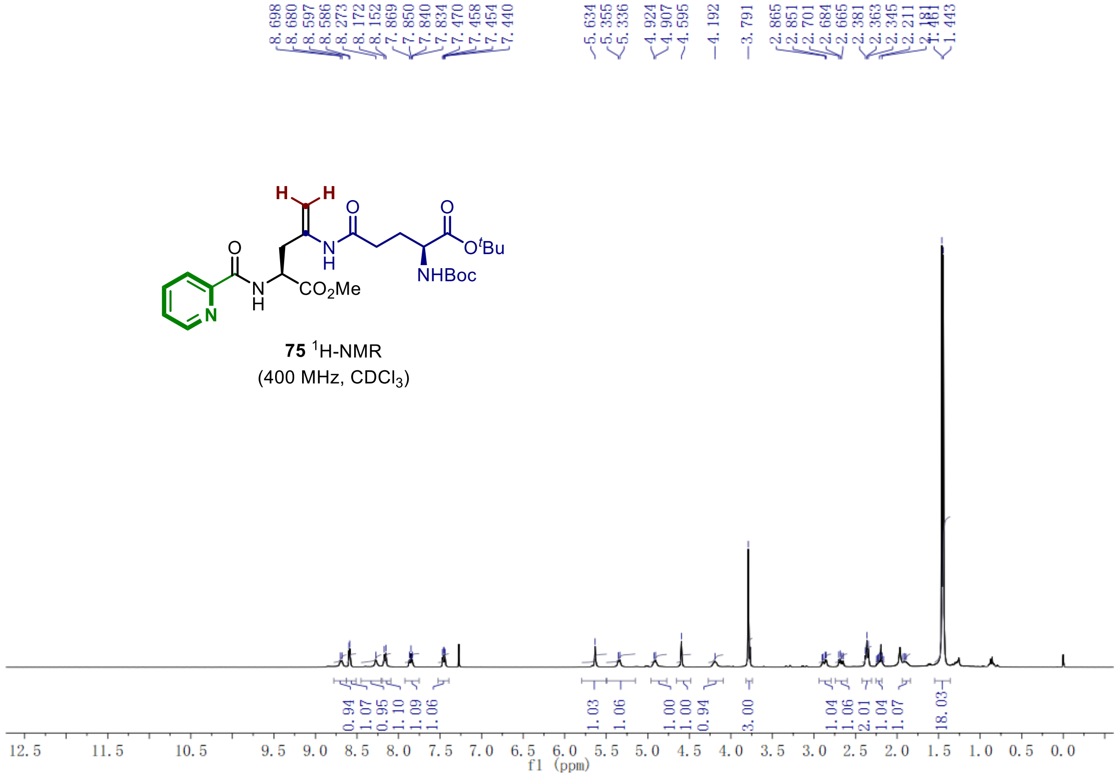

8.698
8.680
8.597
8.586
8.273
8.172
8.152
7.869
7.850
7.840
7.834
7.470
7.458
7.454
7.440
5.634
5.355
5.336
4.924
4.907
4.595
4.192
3.791
2.865
2.851
2.701
2.684
2.665
2.381
2.363
2.345
2.211
1.443
0.94
1.07
0.95
1.10
1.09
1.06
1.03
1.06
1.00
1.00
0.94
3.00
1.04
1.06
2.01
1.04
1.07
18.03
75 ^{1}H-NMR
(400 MHz, $CDCl_3$)
f1 (ppm)

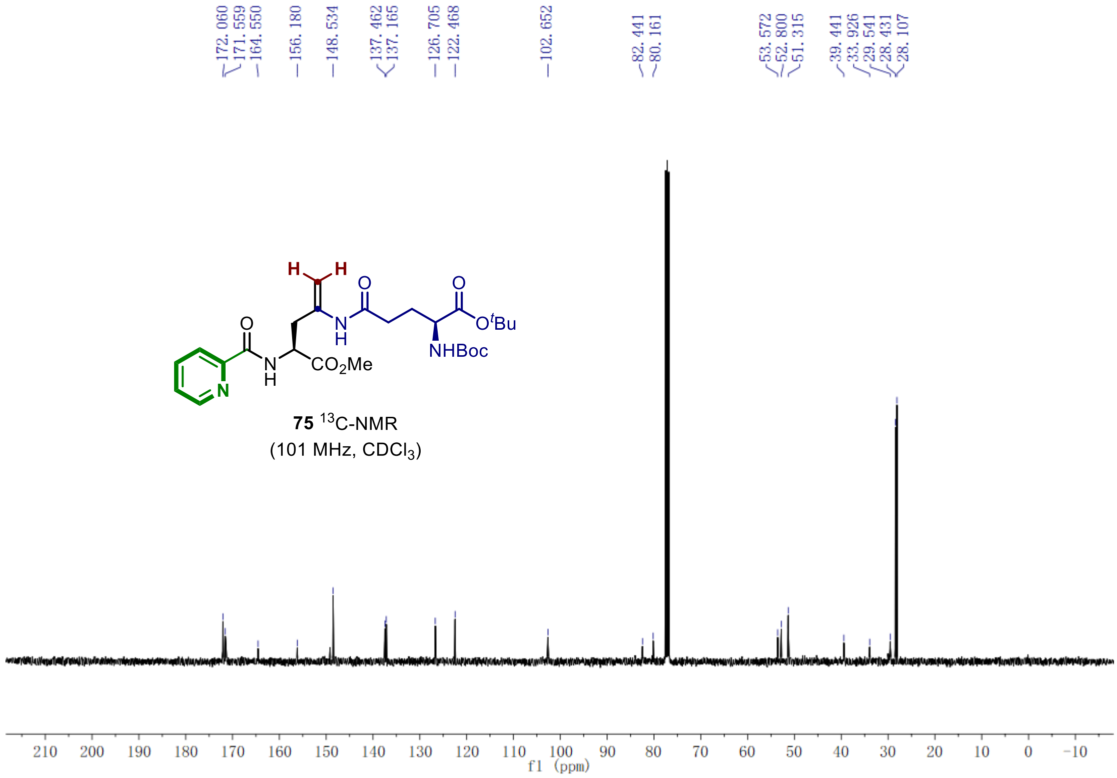

172.060
171.559
164.550
156.180
148.534
137.462
137.165
126.705
122.468
102.652
82.441
80.161
53.572
52.800
51.315
39.441
33.926
29.541
28.431
28.107
75 ^{13}C-NMR
(101 MHz, $CDCl_3$)
f1 (ppm)

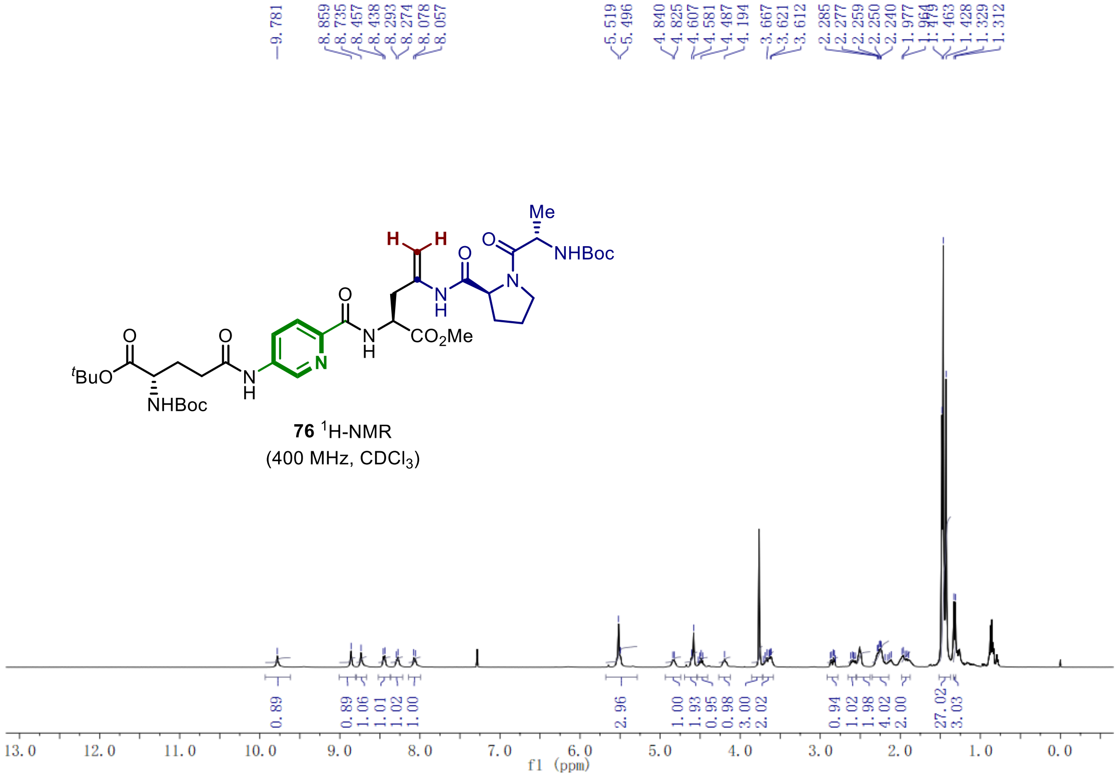
9.781
8.859
8.735
8.457
8.438
8.293
8.274
8.078
8.057
5.519
5.496
4.840
4.825
4.607
4.581
4.487
4.194
3.667
3.621
3.612
2.285
2.277
2.259
2.250
2.240
1.977
1.463
1.428
1.329
1.312
Me
NHBoc
O
H
H
N
CO2Me
tBuO
NHBoc
76 1H-NMR
(400 MHz, CDCl3)
0.89
0.89
1.06
1.01
1.02
1.00
2.96
1.00
1.93
0.95
0.98
3.00
2.02
0.94
1.02
1.98
4.02
2.00
27.02
3.03
13.0
12.0
11.0
10.0
9.0
8.0
7.0
6.0
5.0
4.0
3.0
2.0
1.0
0.0
f1 (ppm)

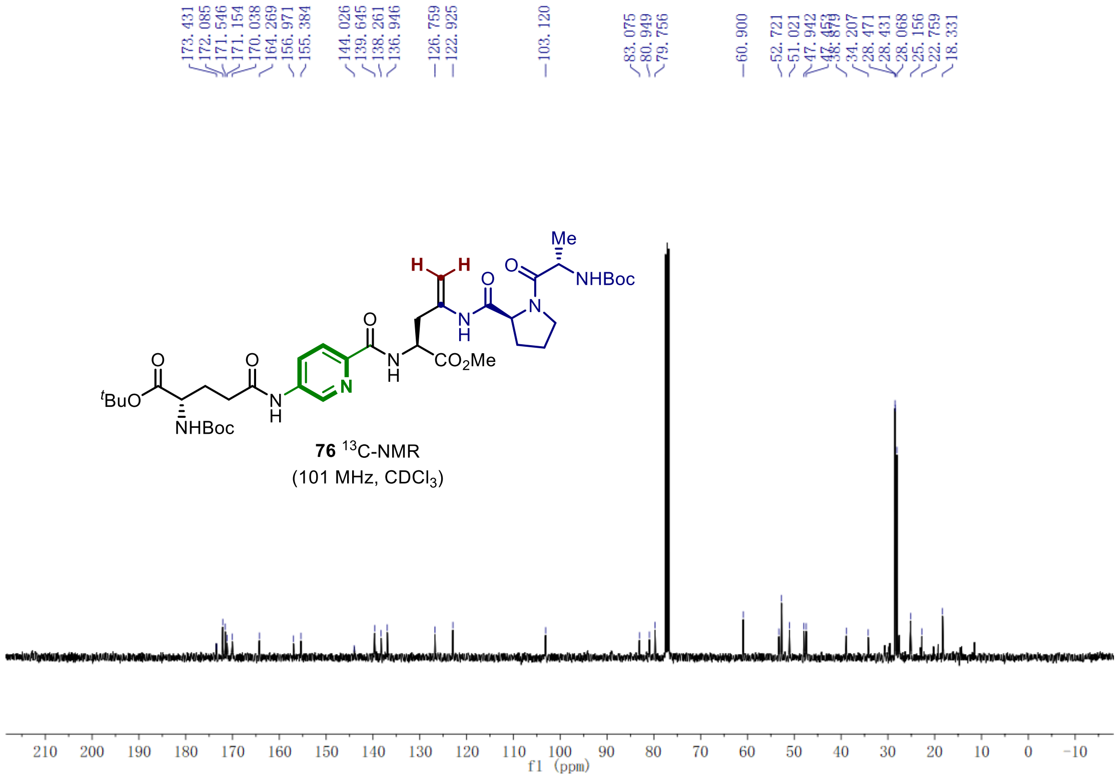
173.431
172.085
171.546
171.154
170.038
164.269
156.971
155.384
144.026
139.645
138.261
136.946
126.759
122.925
103.120
83.075
80.949
79.756
60.900
52.721
51.021
47.942
34.207
28.471
28.431
28.068
25.156
22.759
18.331
Me
NHBoc
O
H
H
N
CO2Me
tBuO
NHBoc
76 13C-NMR
(101 MHz, CDCl3)
210
200
190
180
170
160
150
140
130
120
110
100
90
80
70
60
50
40
30
20
10
0
-10
f1 (ppm)

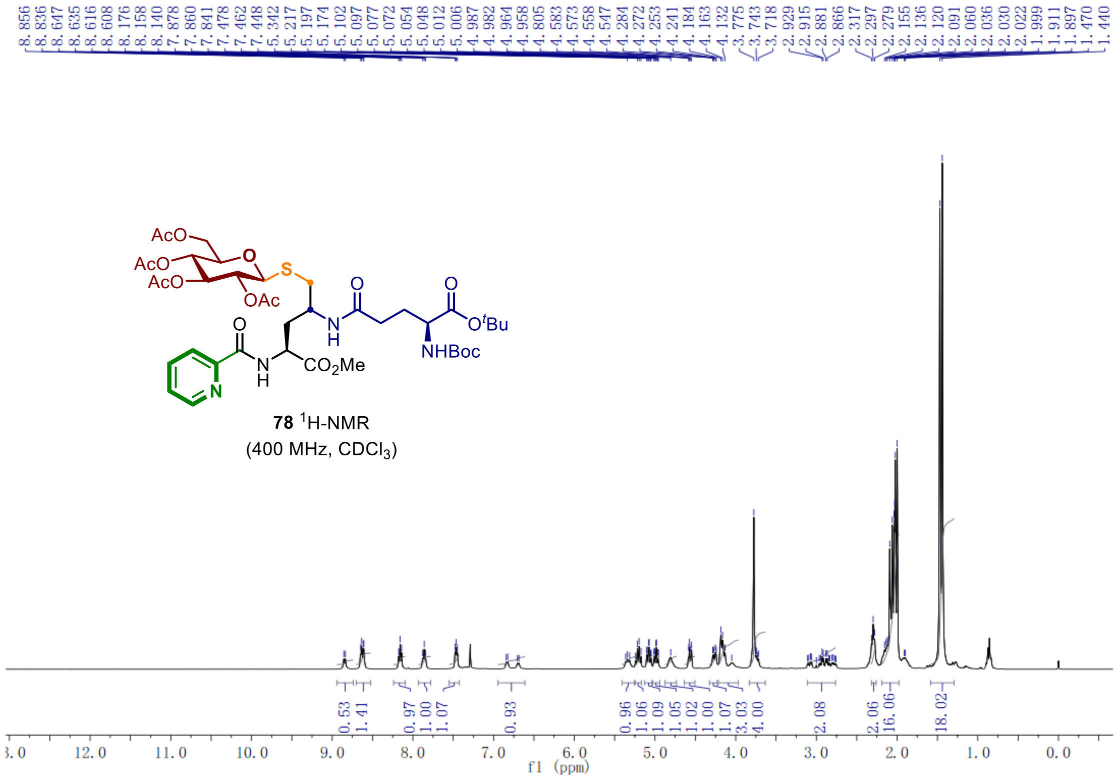
8.856 8.836 8.647 8.635 8.616 8.608 8.176 8.158 8.140 7.878 7.860 7.841 7.478 7.462 7.448 5.342 5.217 5.197 5.174 5.102 5.097 5.077 5.072 5.054 5.048 5.012 5.006 4.987 4.982 4.964 4.958 4.805 4.583 4.573 4.558 4.547 4.284 4.272 4.253 4.241 4.184 4.163 4.132 3.775 3.743 3.718 2.929 2.915 2.881 2.866 2.317 2.297 2.279 2.155 2.136 2.120 2.091 2.060 2.036 2.030 2.022 1.999 1.911 1.897 1.470 1.440
AcO
AcO
AcO
O
S
OAc
O
N
H
O
O^tBu
NHBoc
O
N
H
CO2Me
N
78 1H-NMR
(400 MHz, CDCl3)
0.53 1.41 0.97 1.00 1.07 0.93 0.96 1.06 1.09 1.05 1.02 1.00 1.07 3.03 4.00 2.08 2.06 16.06 18.02
12.0 11.0 10.0 9.0 8.0 7.0 6.0 5.0 4.0 3.0 2.0 1.0 0.0
f1 (ppm)

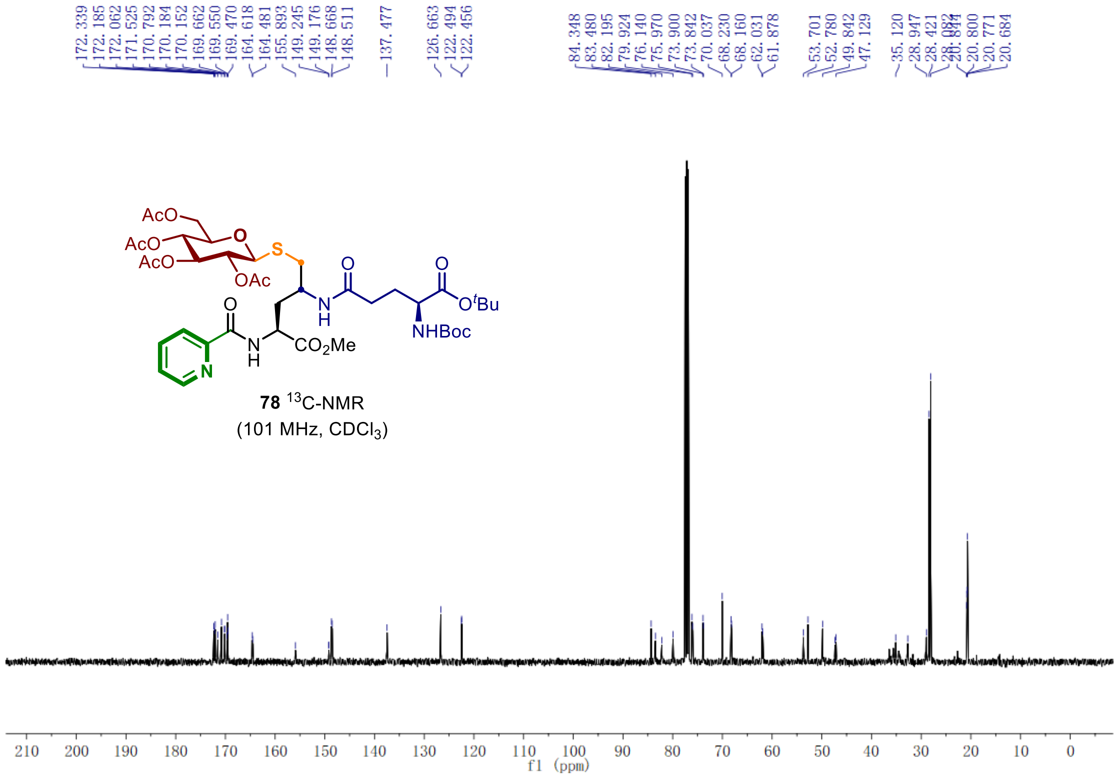
172.339 172.185 172.062 171.525 170.792 170.184 170.152 169.662 169.550 169.470 164.618 164.481 155.893 149.245 149.176 148.668 148.511 137.477 126.663 122.494 122.456
84.348 83.480 82.195 79.924 76.140 75.970 73.900 73.842 70.037 68.230 68.160 62.031 61.878 53.701 52.780 49.842 47.129 35.120 28.947 28.421 20.800 20.771 20.684
AcO
AcO
AcO
O
S
OAc
O
N
H
O
O^tBu
NHBoc
O
N
H
CO2Me
N
78 13C-NMR
(101 MHz, CDCl3)
210 200 190 180 170 160 150 140 130 120 110 100 90 80 70 60 50 40 30 20 10 0
f1 (ppm)

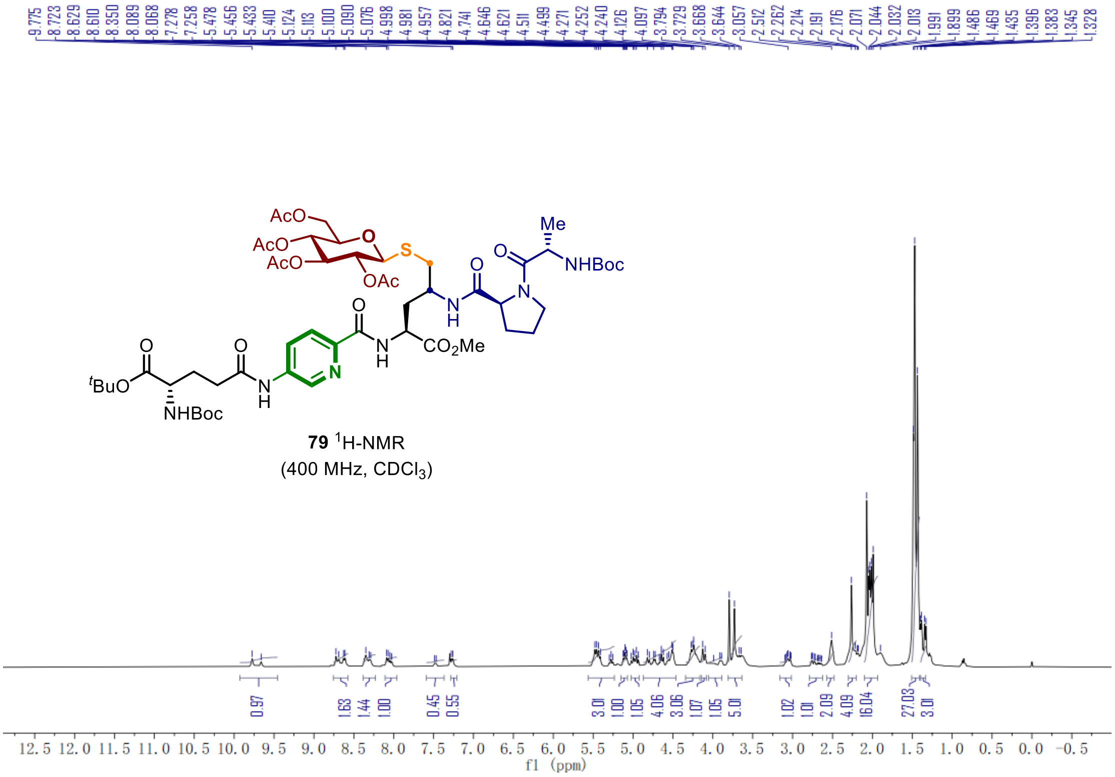

79 ^{1}H-NMR
(400 MHz, $CDCl_3$)

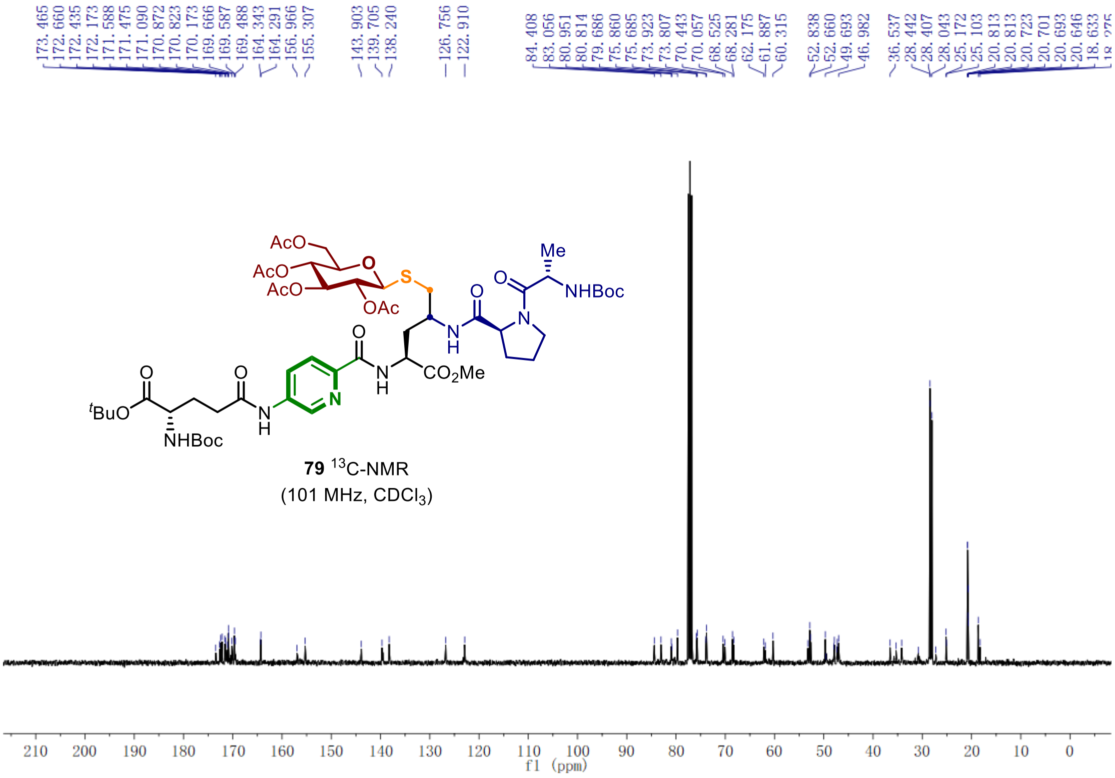

79 ^{13}C-NMR
(101 MHz, $CDCl_3$)

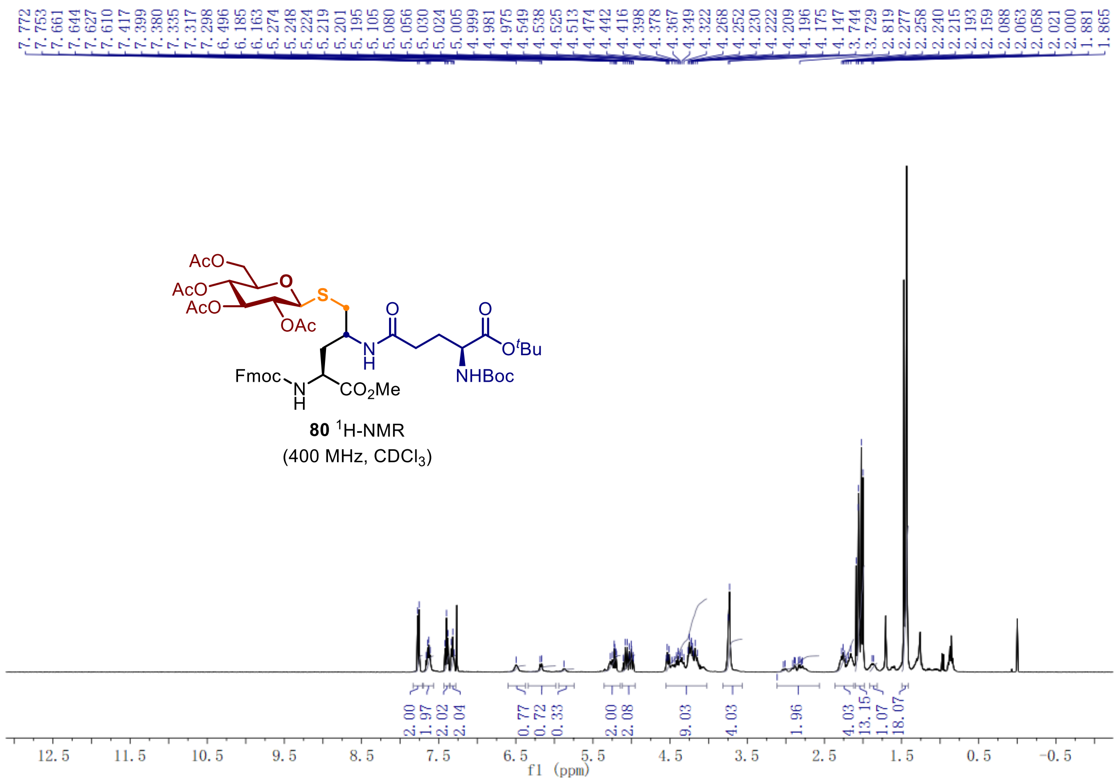
7.772
7.753
7.661
7.644
7.627
7.610
7.417
7.399
7.380
7.335
7.317
7.298
6.496
6.185
6.163
5.274
5.248
5.224
5.219
5.201
5.195
5.105
5.080
5.056
5.030
5.024
5.005
4.999
4.981
4.975
4.549
4.538
4.525
4.513
4.474
4.442
4.416
4.398
4.378
4.367
4.349
4.322
4.268
4.252
4.230
4.222
4.209
4.196
4.175
4.147
3.744
3.729
2.819
2.277
2.258
2.240
2.215
2.193
2.159
2.088
2.063
2.058
2.021
2.000
1.881
1.865
AcO
AcO
AcO
O
S
OAc
O
O
N
H
O^{t}Bu
Fmoc
N
H
CO$_2$Me
NHBoc
80 ^{1}H-NMR
(400 MHz, $CDCl_3$)
2.00
1.97
2.02
2.04
0.77
0.72
0.33
2.00
2.08
9.03
4.03
1.96
4.03
13.15
1.07
18.07
12.5
11.5
10.5
9.5
8.5
7.5
6.5
5.5
4.5
3.5
2.5
1.5
0.5
-0.5
f1 (ppm)

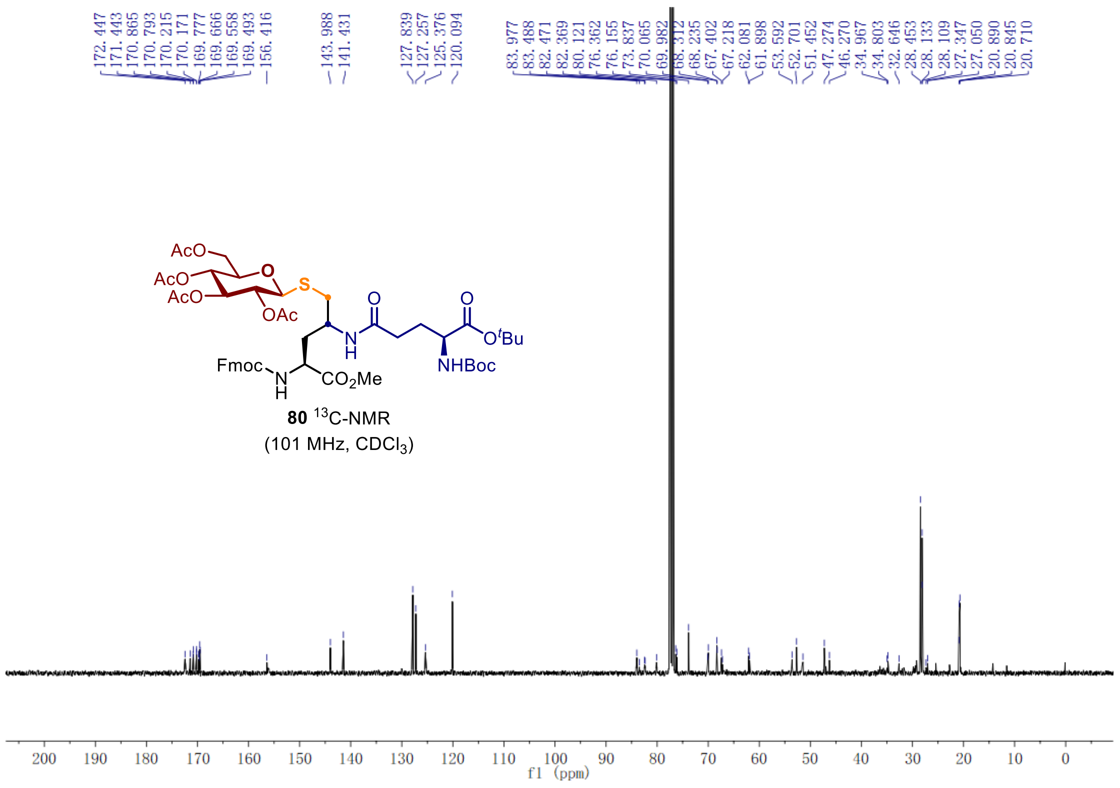
172.447
171.443
170.865
170.793
170.215
170.171
169.777
169.666
169.558
169.493
156.416
143.988
141.431
127.839
127.257
125.376
120.094
83.977
83.488
82.471
82.369
80.121
76.362
76.155
73.837
70.065
69.982
68.312
68.235
67.402
67.218
62.081
61.898
53.592
52.701
51.452
47.274
46.270
34.967
34.803
32.646
28.453
28.133
28.109
27.347
27.050
20.890
20.845
20.710
AcO
AcO
AcO
O
S
OAc
O
O
N
H
O^{t}Bu
Fmoc
N
H
CO$_2$Me
NHBoc
80 ^{13}C-NMR
(101 MHz, $CDCl_3$)
200
190
180
170
160
150
140
130
120
110
100
90
80
70
60
50
40
30
20
10
0
f1 (ppm)
